# Fetal Hepatic Lipidome Is More Greatly Affected by Maternal Rate of Gain Compared with Vitamin and Mineral Supplementation at day 83 of Gestation

**DOI:** 10.3390/metabo13020175

**Published:** 2023-01-25

**Authors:** Ana Clara B. Menezes, Carl R. Dahlen, Kacie L. McCarthy, Cierrah J. Kassetas, Friederike Baumgaertner, James D. Kirsch, Sheri T. Dorsam, Tammi L. Neville, Alison K. Ward, Pawel P. Borowicz, Lawrence P. Reynolds, Kevin K. Sedivec, J. Chris Forcherio, Ronald Scott, Joel S. Caton, Matthew S. Crouse

**Affiliations:** 1 Department of Animal Science, South Dakota State University, Brookings, SD 57006, USA; 2 Center for Nutrition and Pregnancy, Department of Animal Sciences, North Dakota State University, Fargo, ND 58108, USA; 3 Department of Animal Sciences, University of Nebraska-Lincoln, Lincoln, NE 68588, USA; 4 Central Grasslands Research and Extension Center, North Dakota State University, Streeter, ND 58483, USA; 5 Purina Animal Nutrition LLC, Gray Summit, MO 63039, USA; 6 United States Department of Agriculture, Agriculture Research Service, U.S. Meat Animal Research Center, Clay Center, NE 68933, USA

**Keywords:** beef heifer, body weight gain, fetal programming, fetus, lipidomics, fetal liver, vitamin and mineral supplementation

## Abstract

Herein, we evaluated the hepatic lipid metabolic profiles of bovine fetuses in response to maternal vitamin and mineral supplementation (VMSUP; supplemented (VTM) or not (NoVTM)) and two different rates of gain (GAIN; low gain (LG), 0.28 kg/d, or moderate gain (MG), 0.79 kg/d). Crossbred Angus heifers (*n* = 35; initial BW = 359.5 ± 7.1 kg) were randomly assigned to a 2 × 2 factorial arrangement, resulting in the following treatment combinations: NoVTM-LG (*n* = 9), NoVTM-MG (*n* = 9), VTM-LG (*n* = 9), and VTM-MG (*n* = 8). Heifers received their treatments until d 83 of gestation, when they were ovariohysterectomized. Fetuses were harvested and liver samples were analyzed via ultrahigh-performance liquid chromatography–tandem mass spectroscopy to characterize lipid profiles and abundances. We identified 374 biochemicals/metabolites belonging to 57 sub-pathways of the lipid metabolism super-pathway. The majority of the biochemicals/metabolites (*n* = 152) were significantly affected by the main effect of GAIN. Maternal moderate rates of gain resulted in greater abundances (*p* ≤ 0.0001) of ω-3 fatty acids (eicosapentaenoate, docosapentaenoate, and docosahexaenoate) and lower abundances (*p* ≤ 0.0001) of ω-6 fatty acids. Further, MG resulted in the accumulation of several diacylglycerols and depletion of the majority of the monoacylglycerols. Concentrations of nearly all acylcarnitines (*p* ≤ 0.03) were decreased in VTM-LG fetal livers compared to all other treatment combinations, indicating a greater rate of complete oxidation of fatty acids. Levels of secondary bile acids were impacted by VMSUP, being greater (*p* ≤ 0.0048) in NoVTM than in VTM fetal livers. Moreover, NoVTM combined with lower rate of gain resulted in greater concentrations of most secondary bile acid biochemicals/metabolites. These data indicate that maternal diet influenced and altered fetal hepatic lipid composition in the first trimester of gestation. Maternal body weight gain exerted a greater influence on fetal lipid profiles than vitamin and mineral supplementation. Specifically, lower rate of gain (0.28 kg/d) resulted in an increased abundance of the majority of the biochemicals/metabolites identified in this study.

## 1. Introduction

Lipids play a crucial role in fetal development. These molecules are the main constituents of cellular membranes; are essential for energy metabolism, storage, and homeostasis; are required for central nervous system and brain development; and act as key messenger molecules involved in signal transduction and molecular recognition processes [1,2,3,4]. During fetal life, maternal circulation (via placental transfer) is the main source of lipids to the fetus [5]; thus, fetal hepatic lipid profile is influenced by maternal diet and maternal metabolic status [2,5]. Evidence shows [1,6] that maternal nutrition during gestation can permanently affect the metabolism of offspring through epigenetic modifications. Genes related to lipid metabolism are more likely to be altered during embryogenesis and early gestation [1]; thus, maternal nutrient intake during these critical windows may exert a significant influence on the expression of genes involved in lipid metabolism, consequently affecting lipid metabolic pathways in offspring [1].

Our group has developed a research model [7] examining the effects of maternal vitamin and mineral supplementation (VTM (supplemented) vs. NoVTM (not supplemented)) and two different rates of gain (LG (low gain) vs. MG (moderate gain)) during the first 83 days of gestation on maternal and fetal outcomes in beef cattle. We have previously explored amino acid, carbohydrate, and energy profiles in fetal livers at d 83 of gestation [8]. A metabolomic analysis revealed that metabolites in the oxidative phosphorylation pathway were more abundant in the livers of fetuses from VTM than NoVTM dams, suggesting that a greater supply of micronutrients during the pre-conceptional and first trimester of pregnancy may positively modulate mitochondrial energy metabolism in offspring. These changes in the abundance of metabolites suggest physiological adaptations to meet fetal metabolic needs. It is unknown whether the greater supply of vitamins and minerals to dams increases fetal expression of enzymes in the electron transport chain or allows for greater efficiency of the electron transport chain. Either way, energy metabolism and lipid metabolism are interdependent metabolic pathways; thus, exploring fetal lipidomics will expand our knowledge of the energetic and potential metabolic effects of our dietary treatments.

Supporting our theory that the fetal lipidome may be affected by maternal diet, Diniz et al. [9] conducted a differential gene expression analysis for placenta samples from this study. The results revealed the upregulation of *SREBF2* (sterol regulatory element-binding protein 2) and *FADS1* (metabolism of and degradation of polyunsaturated fatty acids) in VTM-LG vs. NoVTM-MG and the upregulation of *HMGCS1*, *FDFT1*, *MSMO1*, and *SQLE* (cholesterol-biosynthesis-related genes) in VTM-LG vs. VTM-MG, suggesting a greater uptake of fatty acids and cholesterol by VTM-LG fetuses. Further, preliminary results [10] showed the upregulation of *ABCA1* and *ABCA6* (cholesterol and metal ion transport) and *PPARG* and *SDR16C5* (lipoprotein transport and metabolism) in VTM vs. NoVTM fetal livers. Thus, a characterization of the lipidomic profiles of fetuses from this project would provide insights into maternal–fetal lipid transport and uptake, allowing a more comprehensive interpretation of the effects of our treatments in fetal lipid metabolism. Therefore, the objective of this study was to analyze the hepatic lipid metabolomes of 83-day-old fetuses in response to maternal vitamin/mineral supplementation and rate of gain. We hypothesized that maternal VTM supplementation combined with low rates of gain would result in a greater abundance of cholesterol metabolites and lower abundances of acylcarnitines and β-hydroxybutyrate, indicating greater efficiency of energy utilization in fetuses from VTM-LG dams.

## 2. Materials and Methods

### 2.1. Ethics Statement

All animal procedures were approved by the North Dakota State University Institutional Animal Care and Use Committee (#A19012). This study is part of a larger study in which heifers were subjected to vitamin and mineral supplementation (from pre-breeding to day 83 post-breeding) and two rates of gain (from breeding to d 83 of gestation). Data reported herein were collected from 35 heifers that were ovariohysterectomized on d 83 ± 0.27 of gestation [7,11].

### 2.2. Animals, Experimental Design, and Dietary Treatments

Treatments, housing, and diets were previously described by Menezes et al. (2022). Briefly, crossbred Angus heifers gestating female fetuses (*n* = 35; initial BW = 359.5 ± 7.1 kg) were randomly assigned to one of four treatments in a 2 × 2 factorial arrangement with main effects of vitamin and mineral supplementation (VMSUP; supplemented (VTM) vs. unsupplemented (NoVTM)) and rate of gain (GAIN; low gain (LG) 0.28 kd/d or moderate gain (MG) 0.79 kg/d), resulting in the following treatment combinations: (1) no vitamin and mineral supplementation, low gain (NoVTM-LG; *n* = 9); (2) no vitamin and mineral supplementation, moderate gain (NoVTM-MG; *n* = 9); (3) vitamin and mineral supplementation, low gain (VTM-LG; *n* = 9); (4) vitamin and mineral supplementation, moderate gain (VTM-MG; *n* = 8).

The VMSUP factor was initiated pre-breeding, allowing time for heifers to alter their mineral status prior to breeding (d 0 of the study). The durations that VTM and NoVTM heifers received their treatments varied according to the breeding groups to which they were assigned. Treatments were initiated on the same calendar day, but breeding occurred over seven breeding-group timepoints due to logistical constraints. (The effect of breeding group was previously tested, and no significant effects were observed). Therefore, the VMSUP factor was initiated 71 to 148 d before artificial insemination (AI). The vitamin and mineral supplement provided vitamins A, D, and E and macro- and trace minerals to meet 110% of the requirements specified by the NASEM [12] and consisted of ground corn carrier and a loose vitamin and mineral premix (Purina Wind & Rain Storm All-Season 7.5 Complete; Land O’Lakes, Inc., Arden Hills, MN, USA) fed at 0.45 kg/heifer/day (337 g of carrier and 113 g of premix). Heifers in the NoVTM treatment received the ground corn carrier at 0.45 kg/heifer/day with no addition of vitamins and minerals. To complete the factorial arrangement of treatment, heifers were randomly assigned to either LG or MG treatments within their respective VMSUP factor at the time of AI. To achieve LG, heifers were maintained on the basal diet and targeted to gain 0.28 kg/d. To achieve MG (0.79 kg/d), heifers were fed the basal diet with the addition of a protein/energy supplement (a blend of ground corn, dried distillers’ grains plus solubles, wheat midds, fish oil, urea, and ethoxyquin) fed at a rate of 0.58% of BW as-fed daily. Supplements were top-dressed over a basal diet consisting of prairie grass hay, corn silage, and dried distillers’ grains plus solubles. Heifers were individually fed, once daily, in an electronic head gate facility (American Calan, Northwood, NH, USA) and received treatments until the experiment endpoint of d 83 ± 0.27 after AI, when they were ovariohysterectomized [13].

### 2.3. Sample Collection

Following ovariohysterectomy, the fetus was removed from the gravid uterus and dissected. Fetal liver was collected, and 50 mg samples were weighed, placed in 2 mL cryotubes, snap-frozen on dry ice, and stored at −80 °C until further analysis. Samples were shipped to Metabolon, Inc. (Morrisville, NC, USA), where their lipid metabolic profiles were determined.

Sample preparations and analyses in this study and in Crouse et al. [8] were performed by Metabolon, Inc. (Morrisville, NC, USA). Therefore, the descriptions of all procedures herein and in the aforementioned paper are similar.

### 2.4. Sample Preparation

Liver samples were prepared using the automated MicroLab STAR^®^ system (Hamilton Company, Reno, NV, USA). Briefly, proteins were precipitated with methanol under vigorous shaking for 2 min (Glen Mills GenoGrinder 200, Clifton, NJ, USA) followed by centrifugation. The resulting extract was divided into five fractions: two for analysis by two separate reverse-phase (RP) ultrahigh-performance liquid chromatography (UPLC)–tandem mass spectrometers (MS/MS) with positive-ion-mode electrospray ionization (ESI), one for analysis by RP/UPLC-MS/MS with negative-ion-mode ESI, one for analysis by hydrophilic interaction liquid chromatography (HILIC)/UPLC-MS/MS with negative-ion-mode ESI, and one sample was reserved for backup. Samples were placed briefly on a TurboVap^®^ (Zymark, Hopkinton, MA, USA) to remove the organic solvent. The sample extracts were then stored overnight under nitrogen before preparation for analysis.

### 2.5. Sample Analysis

The lipidomic analyses presented herein and the metabolomic analyses [8] were performed by Metabolon, Inc. (Morrisville, NC, USA). Thus, the methodologies described in both studies are similar.

#### 2.5.1. Quality Control

For quality control [8], the following controls were analyzed in concert with the experimental samples: (1) a pooled matrix sample generated by taking a small volume of each experimental sample, which served as a technical replicate throughout the data set; (2) extracted water samples, which served as process blanks; and (3) a recovery standard and an internal standard, which were spiked into every analyzed sample, allowing monitoring of instrument performance and chromatographic alignment. Instrument variability was determined by calculating the median relative standard deviations (RSDs) for the standards that were added to each sample prior to injection into the mass spectrometers. Overall process variability was determined by calculating the median RSDs for all endogenous metabolites (i.e., non-instrument standards) present in 100% of the pooled matrix samples. Experimental samples were randomized across the platform run with QC samples spaced evenly among the injections. The total mean instrument variability for internal standards was 3%, and the total process variability for endogenous biochemicals/metabolites was 7%.

#### 2.5.2. Ultrahigh-Performance Liquid Chromatography–Tandem Mass Spectroscopy (UPLC-MS/MS)

All methods [8] utilized Waters ACQUITY ultrahigh-performance liquid chromatography and a Thermo Scientific Q-Exactive high-resolution/accurate mass spectrometer interfaced with a heated electrospray ionization (HESI-II) source and an Orbitrap mass analyzer operated at 35,000 mass resolution. The sample extracts were dried and then reconstituted in solvents compatible with each of the four methods. Each reconstitution solvent contained a series of standards at fixed concentrations to ensure injection and chromatographic consistency. One aliquot was analyzed using acidic positive-ion conditions chromatographically optimized for more hydrophilic compounds. In this method, the extract was gradient-eluted from a C18 column (Waters UPLC BEH C18-2.1 × 100 mm, 1.7 µm) using water and methanol, containing 0.05% perfluoropentanoic acid (PFPA) and 0.1% formic acid (FA). Another aliquot was also analyzed using acidic positive-ion conditions; however, it was chromatographically optimized for more hydrophobic compounds. In this method, the extract was gradient-eluted from the same aforementioned C18 column using methanol, acetonitrile, water, 0.05% PFPA, and 0.01% FA and was operated at an overall higher organic content. Another aliquot was analyzed using basic negative-ion optimized conditions using a separate, dedicated C18 column. The basic extracts were gradient-eluted from the column using methanol and water with 6.5 mM ammonium bicarbonate at pH 8. The fourth aliquot was analyzed via negative ionization following elution from a HILIC column (Waters UPLC BEH Amide 2.1 × 150 mm, 1.7 µm) using a gradient consisting of water and acetonitrile with 10 mM ammonium formate, pH 10.8. The MS analysis alternated between MS and data-dependent MSn scans using dynamic exclusion. The scan range varied slightly between methods but covered 70–1000 m/z.

#### 2.5.3. Data Extraction and Compound Identification

Raw data were extracted, peak-identified, and QC-processed using Metabolon’s hardware and software [8]. The informatics system consisted of four major components, the Laboratory Information Management System (LIMS), the data-extraction and peak-identification software, data-processing tools for QC and compound identification, and a collection of information-interpretation and -visualization tools for use by data analysts. Compounds were identified by comparison with library entries of purified standards or recurrent unknown entities. Metabolon maintains a library based on authenticated standards that contains the retention time/indexes (RIs), mass-to-charge (m/z) ratios, and chromatographic data (including MS/MS spectral data) for each molecule present in the library. Further, biochemical identifications were based on three criteria: (1) retention index within a narrow RI window of the proposed identification; (2) accurate mass match to the library +/− 10 ppm; and (3) the MS/MS forward and reverse scores for the experimental data and authentic standards. The MS/MS scores are based on comparisons of the ions present in the experimental spectra with the ions present in the library spectra.

### 2.6. Statistical Analysis

Biochemical/metabolite data were log-transformed and analyzed by two-way ANOVA for main effects of VMSUP, GAIN, and their interaction. Contrasts between treatments were conducted by two-way ANOVA contrasts, and *p*-values ≤ 0.05 were considered significant. Tendencies will not be discussed. Pathway enrichment was calculated with the MetaboLync Pathway Analysis software (Morrisville, NC, USA). using the following formula: (k/m)/(n/N), where k = the number of significant metabolites per pathway, m = the total number of detected metabolites per pathway, n = the number of significant metabolites in the study, and N = the total number of detected metabolites in the study, as previously described by Simintiras et al. [14]. Pathways with enrichment scores > 1 have more metabolites with statistically significant fold changes compared to all other pathways within the study.

## 3. Results

### 3.1. Enrichment Score Analysis

A total of 57 sub-pathways belonging to the lipid metabolism super-pathway were identified. An enrichment analysis (Table 1) revealed that 12 sub-pathways were enriched for VMSUP × GAIN interaction: dicarboxylate fatty acids (0.88), branched-chain amino acid (BCAA) metabolism (5.62), short-chain acylcarnitine (5.35), medium-chain acylcarnitine (8.32), monounsaturated acylcarnitine (14.02), polyunsaturated acylcarnitine (7.52), hydroxy acylcarnitine (11.76), carnitine metabolism (8.04), inositol metabolism (8.04), phospholipid metabolism (1.59), glycerolipid metabolism (5.35), and secondary bile acid metabolism (3.61). The remaining pathways had enrichment scores of 0 for VMSUP × GAIN interactions.

Seven sub-pathways were enriched for the main effect of VMSUP, namely, dicarboxylate fatty acids (1.04), amino fatty acids (9.5), polyunsaturated acylcarnitine metabolism (2.09), phospholipid metabolism (1.88), lysoplasmalogens (3.79), ceramides (5.91), dihydrosphingomyelins (11.9), sphingomyelins (0.74), primary bile acid metabolism (3.15), and secondary bile acid metabolism (11.51).

The majority of the sub-pathways, 32 out of the 57, were enriched for the main effect of GAIN. Among them, phosphatidylglycerol (4.28), phosphatidylinositol (4.33), and phosphatidylserine (4.33) were considered the three most significantly enriched significant ones.

### 3.2. Statistical Heat Map—Specific Interactive and Main Effects

All of the following statistical heat map data are presented in Table 2. Box plots of all metabolites presented in Table 2 are included in Supplementary Materials Figure S1. For added visualization of two-way ANOVA and two-way ANOVA contrasts please refer to Supplementary Materials Table S1.

The following biochemical pathways did not have any biochemicals/metabolites affected by a VMSUP × GAIN interaction (*p* ≥ 0.1583) or main effects of VMSUP (*p* ≥ 0.0607) and GAIN (*p* ≥ 0.0611): fatty acid synthesis, fatty acid metabolism, short-chain fatty acids, long-chain saturated fatty acids, branched fatty acids, glycosyl PE, ketone bodies, sphingolipid synthesis, dihydroceramides, lactosylceramides, sphingosines, mevalonate metabolism, sterols, pregnenolone steroids, progestin steroids, corticosteroids, and androgenic steroids.

The medium-chain fatty acid pathway did not have any biochemicals/metabolites affected by a VMSUP × GAIN interaction (*p* ≥ 0.1356) or by the main effect of VMSUP (*p* ≥ 0.4936). However, pelargonate (*p* = 0.0264) and 2- and 3-decenoate (*p* = 0.0038) were affected by the main effect of GAIN; there was a greater accumulation of both metabolites in LG compared to MG.

Levels of long-chain monounsaturated fatty acids were not affected by a VMSUP × GAIN interaction (*p* ≥ 0.3901) or by VMSUP (*p* ≥ 0.0852). However, oleate/vaccenate, 10-nonadecenoate, and eicosenoate were affected by GAIN (*p* ≥ 0.0160), their levels being greater in LG than in MG.

In the long-chain polyunsaturated fatty acid (PUFA) pathway, none of the biochemicals/metabolites were affected by a VMSUP × GAIN interaction (*p* ≥ 0.1825) or by the main effect of VMSUP (*p* ≥ 0.1603). Most of the biochemicals/metabolites in this pathway were affected by GAIN: levels of stearidonate, eicosapentaenoate, heneicosapentaenoate, docosapentaenoate, docosahexaenoate, and nisinate were greater in MG than in LG (*p* ≤ 0.0001), while dihomo-linoleate, docosatrienoate, docosapentaenoate, docosadienoate, and mead acid levels were greater in LG than in MG (*p* ≤ 0.0001).

In the dicarboxylate fatty acid pathway, the only biochemical/metabolite affected by a VMSUP × GAIN interaction was 3-hydroxyadipate (*p* = 0.0427): the concentration in NoVTM-LG was 0.54-fold greater than for all other treatments. None of the biochemicals/metabolites in this pathway were affected by VMSUP (*p* ≥ 0.0648); however, heptenedioate, hexadecanedioate, and hexadecenedioate levels were affected by GAIN (*p* ≤ 0.0048), being greater in LG than in MG.

Only two biochemicals were identified in the amino fatty acid pathway: 2-aminoheptanoate and 2-aminooctanoate. The former was affected by the main effects of VMSUP (greater in VTM than in NoVTM; *p* = 0.0014) and GAIN (greater in LG than in MG; *p* = 0.0026), while the latter was not affected by VMSUP (*p* = 0.1428), GAIN (*p* = 0.0774), or their interaction (*p* = 0.1983).

In the BCAA pathway, propionyl CoA (*p* = 0.0349) and propionylcarnitine C3 (*p* = 0.0364) were affected by a VMSUP × GAIN interaction, whereas butyrylglycine was affected by GAIN (*p* = 0.0264), levels being greater in LG than in MG. None of the biochemicals/metabolites in this pathway were affected by VMSUP (*p* ≥ 0.2451).

The biochemicals/metabolites of the acylglycine pathway were not affected by a VMSUP × GAIN interaction (*p* ≥ 0.8391) nor by VMSUP (*p* ≥ 0.2440). 3-hydroxybutyroylglycine was the only biochemical affected by GAIN (*p* = 0.0247), its levels being greater in LG than in MG.

Levels of biochemicals/metabolites in the short- and medium-chain acylcarnitine pathways, as well as in the monounsaturated acylcarnitine pathway, were not affected by VMSUP or GAIN, but significant VMSUP × GAIN interactions were observed. In the short-chain acylcarnitine pathway, valerylcarnitine (*p* = 0.0198) was 0.77-fold greater in NoVTM-LG than in all other treatments. Levels of the following medium-chain acylcarnitines: cis-3,4-methyleneheptanoylcarnitine (*p* = 0.02), decanoylcarnitine (*p* = 0.03), and laurylcarnitine (*p* = 0.03) were nearly 1.47-fold lower in VTM-LG compared to all other treatments, while in the monounsaturated acylcarnitine pathway, the abundances of cis-4-decenoylcarnitine, 5-dodecenoylcarnitine, myristoleoylcarnitine, palmitoleoylcarnitine, oleoylcarnitine, erucoylcarnitine, and nervonoylcarnitine were affected (*p* ≥ 0.0029) by VMSUP × GAIN interactions.

The significant biochemicals/metabolites in the polyunsaturated acylcarnitine pathway influenced by a VMSUP × GAIN interaction (*p* ≥ 0.0031) were linoleoylcarnitine, dihomo-linoleoylcarnitine, docosadienoylcarnitine, and docosatrienoylcarnitine. Linolenoylcarnitine was affected by VMSUP (*p* = 0.0381), its levels being greater in NoVTM than in VTM. Abundances of meadoylcarnitine (*p* ≤ 0.0001) and docosahexaenoylcarnitine (*p* = 0.0005) were affected by GAIN, levels of the former being greater in LG than in MG, and levels of the latter being greater in MG than in LG.

In the dicarboxylate acylcarnitine pathway, none of the three biochemicals identified, adipoylcarnitine, pimeloylcarnitine/3-methyladipoylcarnitine, or suberoylcarnitine, were affected by a VMSUP × GAIN interaction (*p* ≥ 0.0975) or by the main effect of VMSUP (*p* ≥ 0.7234). However, suberoylcarnitine was affected by GAIN (*p* = 0.0363), its levels being greater in LG than in MG.

In the hydroxy acylcarnitine pathway, the following biochemicals were affected by a VMSUP × GAIN interaction (*p* ≥ 0.0001): (S)-3-hydroxybutyrylcarnitine, 3-hydroxyhexanoylcarnitine, 3-hydroxyhexanoylcarnitine, 3-hydroxyoctanoylcarnitine, 3-hydroxyoctanoylcarnitine, and 3-hydroxypalmitoylcarnitine. Further, none of the biochemicals identified in this pathway were affected by either VMSUP (*p* ≥ 0.1519) or GAIN (*p* ≥ 0.0652).

Of the two biochemicals identified in the carnitine metabolism pathway, carnitine and deoxycarnitine, only carnitine was affected by a VMSUP × GAIN interaction (*p* = 0.0204): in NoVTM-LG, the concentration was 0.76-fold greater than for all other treatments. Neither carnitine nor deoxycarnitine were affected by the main effects of VMSUP (*p* ≥ 0.0909) or GAIN (*p* ≥ 0.2280).

Biochemicals/metabolites identified in the acylcholine, monohydroxy fatty acid, dihydroxy fatty acid, eicosanoid, and endocannabinoid pathways were affected only by GAIN: palmitoylcholine and oleoylcholine (acylcholine pathway; *p* = 0.0326 and 0.0086, respectively) were greater in MG than in LG. Levels of the monohydroxy fatty acids 2-hydroxyoctanoate (*p* = 0.0120), 2-hydroxydecanoate (*p* = 0.0048), and 3-hydroxylauratewere (*p* = 0.0099) were greater in LG than in MG. This same pattern was observed for 3,4-dihydroxybutyrate (dihydroxy fatty acid pathway; *p* = 0.0384) and prostaglandin F2alpha and 12-HHTrE (eicosanoid pathway; *p* ≥ 0.0030). In the endocannabinoid pathway, linoleoyl ethanolamide (*p* = 0.0131) and N-oleoylserine (*p* = 0.0137) were more abundant in MG than in LG and in LG than in MG, respectively.

A significant VMSUP × GAIN interaction (*p* = 0.0230) was observed for myo-inositol (inositol metabolism pathway): in NoVTM-LG, its concentration was 1.10-fold lower than for all other treatments. The other biochemical identified in this pathway, inositol 1-phosphate, was not affected by VMSUP (*p* = 0.1155), GAIN (*p* = 0.2348), or their interaction (*p* = 0.1737).

Out of all biochemicals/metabolites identified in the phospholipid metabolism pathway, choline phosphate was the only one affected by a VMSUP × GAIN interaction (*p* = 0.0179). Glycerophosphoinositol and trimethylamine N-oxide were affected by GAIN (*p* = 0.0455 and 0.0008, respectively), their levels being greater in MG than in LG. In addition, the abundance of trimethylamine N-oxide was affected by VMSUP (*p* = 0.0037), its levels being greater in NoVTM than in VTM.

Seventeen out of the 20 biochemicals/metabolites identified in the phosphatidylcoline pathway were affected by GAIN (*p* ≤ 0.0457). Of these biochemicals/metabolites, levels of 1-myristoyl-2-arachidonoyl-GPC, 1-palmitoyl-2-palmitoleoyl-GPC, 1-palmitoyl-2-oleoyl-GPC, 1-palmitoyl-2-gamma-linolenoyl-GPC, 1-palmitoyl-2-arachidonoyl-GPC, 1-stearoyl-2-oleoyl-GPC, and 1,2-dioleoyl-GPC were greater in LG than in MG. Conversely, the abundances of 1,2-dipalmitoyl-GPC, 1-palmitoyl-2-stearoyl-GPC, 1-palmitoyl-2-docosahexaenoyl-GPC, 1-palmitoleoyl-2-linoleoyl-GPC, 1,2-distearoyl-GPC, 1-stearoyl-2-linoleoyl-GPC, 1-stearoyl-2-docosahexaenoyl-GPC, 1-oleoyl-2-linoleoyl-GPC, 1-oleoyl-2-docosahexaenoyl-GPC, and 1,2-dilinoleoyl-GPC were greater in MG than in LG.

In the phosphatidylethanolamine pathway, only 1,2-dipalmitoyl-GPE (*p* = 0.8622) and 1-oleoyl-2-arachidonoyl-GPE (*p* = 0.0972) were not affected by GAIN. All other biochemicals/metabolites were significantly affected by GAIN (*p* ≤ 0.0300). Of these biochemicals/metabolites, MG resulted in greater accumulation of 1-palmitoyl-2-linoleoyl-GPE, 1-palmitoyl-2-docosahexaenoyl-GPE, 1-stearoyl-2-docosahexaenoyl-GPE, 1-oleoyl-2-linoleoyl-GPE, and 1-oleoyl-2-docosahexaenoyl-GPE than LG, whereas the opposite was observed for 1-palmitoyl-2-oleoyl-GPE, 1-palmitoyl-2-arachidonoyl-GPE, 1-palmitoleoyl-2-oleoyl-GPE, 1-stearoyl-2-oleoyl-GPE, 1-stearoyl-2-arachidonoyl-GPE, and 1,2-dioleoyl-GPE.

All biochemicals/metabolites in the phosphatidylserine, phosphatidylglycerol, and phosphatidylinositol pathways were affected by GAIN (*p* ≤ 0.0115). With the exception of 1-stearoyl-2-linoleoyl-GPS, 1-palmitoyl-2-arachidonoyl-GPI, 1-stearoyl-2-arachidonoyl-GPI, and 1-oleoyl-2-arachidonoyl-GPI, all other biochemical/metabolite concentrations were greater in LG than in MG.

A significant effect of GAIN (*p* ≤ 0.0461) was also observed in the lysophospholipid pathway: levels of nine biochemicals were greater in LG than in MG (1-palmitoleoyl-GPC, 1-oleoyl-GPC, 1-arachidonoyl-GPC, 1-oleoyl-GPE, 1-arachidonoyl-GPE, 1-oleoyl-GPS, 1-palmitoyl-GPG, 1-stearoyl-GPG, 1-oleoyl-GPG, and 1-linoleoyl-GPG), and levels of three biochemicals (1-linoleoyl-GPC, 1-lignoceroyl-GPC, and 1-arachidonoyl-GPI) were greater in MG than in LG.

As for the last six pathways mentioned before, the plasmalogen pathway was only affected by GAIN (*p* ≤ 0.0401): levels of 1-(1-enyl-palmitoyl)-2-oleoyl-GPE, 1-(1-enyl-palmitoyl)-2-palmitoleoyl-GPC, 1-(1-enyl-palmitoyl)-2-arachidonoyl-GPE, and 1-(1-enyl-stearoyl)-2-oleoyl-GPE were greater in LG than in MG, but the abundance of 1-(1-enyl-palmitoyl)-2-linoleoyl-GPC was greater in MG than in LG.

In the lysoplasmalogen pathway, none of the identified biochemicals/metabolites were affected by a VMSUP × GAIN interaction (*p* ≥ 0.4377); however, the abundance of 1-(1-enyl-oleoyl)-GPE was affected by the main effects of GAIN (*p* = 0.0002; greater in LG than in MG) and VMSUP (*p* = 0.0370; greater in VTM than in NoVTM). A significant effect of GAIN was also observed for 1-(1-enyl-stearoyl)-GPE (*p* = 0.0406) and 1-(1-enyl-oleoyl)-2-oleoyl-GPE (*p* = 0.0001): levels of the first were greater in MG than in LG, and levels of the second were greater in LG than in MG.

In the glycerolipid metabolism pathway, glycerol 3-phosphate was the only biochemical influenced by a VMSUP × GAIN interaction (*p* = 0.0184): in NoVTM-LG, the concentration was 1.10-fold lower than for all other treatments. None of the biochemicals/metabolites in this pathway were affected by main effects of VMSUP (*p* ≥ 0.2183) or GAIN (*p* ≥ 0.2120).

Monoacyl- and diacylglycerols were only affected by GAIN (*p* ≤ 0.0314). The moderate rate of gain resulted in the accumulation of several diacylglycerols (with the exception of palmitoyl-arachidonoyl-glycerol, which was not affected by VMSUP, GAIN, or their interaction; *p* ≥ 0.2181) and the depletion of the majority of the monoacylglycerols (except 1-eicosapentaenoylglycerol and 2-eicosapentaenoylglycero, levels of which were greater in MG than in LG).

None of the ceramides were affected by a VMSUP × GAIN interaction (*p* ≥ 0.2937). The VTM supplementation resulted in lower abundances (*p* ≤ 0.0459) of N-palmitoyl-sphingadienine, N-stearoyl-sphingadienine, and N-behenoyl-sphingadienine compared to NoVTM. A significant effect of GAIN (*p* ≤ 0.0299) was observed for five biochemicals in the ceramide pathway; with the exception of ceramide (d18:1/17:0, d17:1/18:0), levels of all the others were greater in LG than in MG.

The two biochemicals identified in the hexosylceramide pathway were not affected by either a VMSUP × GAIN interaction (*p* ≥ 0.7008) or by VMSUP (*p* ≥ 0.4394). However, glycosyl-N-palmitoyl-sphingosine was affected by GAIN (*p* = 0.0150), being greater in LG than in MG.

The only glycosphingolipid sulfate identified, 3-sulfo-palmitoyl-galactosylceramide, was affected by GAIN (*p* = 0.0210), its abundance being greater in MG than in LG. No significant effect was observed for VMSUP (*p* = 0.4461) or VMSUP × GAIN interaction (*p* = 0.9192).

In the dihydrosphingomyelin pathway, behenoyl dihydrosphingomyelin, sphingomyelin (d18:0/20:0, d16:0/22:0), and sphingomyelin (d18:0/18:0, d19:0/17:0) were affected by VMSUP (*p* ≤ 0.0368), their levels being greater in VTM than in NoVTM. Further, the latter biochemical was affected by GAIN (*p* = 0.0013), its levels being greater in LG than in MG. A GAIN effect (*p* = 0.0004) was also observed for palmitoyl dihydrosphingomyelin, its levels being greater in MG than in LG.

The sphingomyelin pathway did not have any biochemicals/metabolites affected by a VMSUP × GAIN interaction (*p* ≥ 0.0570). However, behenoyl, tricosanoyl, and 11 other sphingomyelin biochemicals were affected by GAIN (*p* ≤ 0.0001). Lastly, a VMSUP effect (*p* = 0.0227) was observed for sphingomyelin (d17:2/16:0, d18:2/15:0), its abundance being greater in NoVTM than in VTM.

None of the primary bile acids identified were affected by a VMSUP × GAIN interaction (*p* ≥ 0.1563) or by GAIN (*p* ≥ 0.0827). Further, taurochenodeoxycholate was the only primary bile acid affected by VMSUP, being more abundant in VTM than in NoVTM.

For the secondary bile acid metabolism pathway, glycoursodeoxycholate (*p* = 0.0434) and tauroursodeoxycholate (*p* = 0.0366) were more abundant in the livers of the NoVTM-LG group compared to all other treatments. Maternal VTM supplementation (*p* ≤ 0.0428) resulted in lower concentrations of deoxycholate, glycodeoxycholate, taurodeoxycholate, and taurocholenate sulfate and greater concentrations of taurolithocholate. Further, the abundances of glycodeoxycholate, taurolithocholate, and taurocholenate sulfate were also affected by GAIN (*p* ≤ 0.0351): MG resulted in lower concentrations of glycodeoxycholate and higher concentrations of taurolithocholate and taurocholenate sulfate.

## 4. Discussion

In this comprehensive lipidomic study, we expanded our previous investigation [8], showing that the fetal metabolome is affected by maternal vitamin/mineral supplementation (from pre-breeding to d 83 of gestation) and rates of gain (from breeding to d 83 of gestation). This study revealed further details, providing quantitative measurements of 374 biochemicals/metabolites belonging to 57 sub-pathways of the lipid metabolism super-pathway. The majority of the metabolites (*n* = 152) were significantly affected by the main effect of GAIN, and, interestingly, greater abundances were observed in LG fetal livers compared to MG livers. Herein, we demonstrated that moderate rates of gain resulted in greater concentrations of PUFAs and diacylglycerols and lower concentrations of monoacylglycerols in fetal livers. Further, our data demonstrate that vitamin and mineral supplementation combined with low rates of gain resulted in lower abundances of acylcarnitines in fetal livers, which may indicate greater efficiency of energy utilization. Even though we previously reported (Diniz et al., 2021) an upregulation of genes related to cholesterol metabolism in maternal placental caruncles of VTM-LG heifers, in the present study none of the metabolites of the sterol sub-pathway were affected by VTM-LG. Lastly, secondary bile acid metabolites were significantly affected by VMSUP, with greater concentrations in NoVTM fetal livers compared to VTM livers. The data generated in this study are unique and help elucidate metabolic programming adaptations in fetal lipid metabolism in response to maternal diet and rate of gain during the first trimester of pregnancy. A detailed discussion of the main findings of this study is presented below, and the complete data set can be found in the Supplementary Materials.

Concentrations of long-chain polyunsaturated fatty acids (PUFAs) in maternal circulation are positively correlated with concentrations of these fatty acids in the diet [5,15]. As maternal circulation is the main source of PUFAs to the fetus [1,16], the profile as well as the concentrations of PUFAs in fetal liver are directly related to maternal dietary intake of PUFAs. Heifers fed to achieve moderate rates of gain were supplemented with a supplement containing fish oil, which is a rich source of ω-3 PUFAs; thus, as expected, greater abundances of ω-3 PUFAs, especially eicosapentaenoate (EPA), docosapentaenoate (DPA), and docosahexaenoate (DHA), were observed in fetal livers in the MG group compared to the LG group. Eicosapentaenoate and DHA are involved in retinal and nervous system maturation and neurotransmitter metabolism, which are crucial for fetal development [17]. In a recent review, Roque-Jimenez et al. [5] highlighted that the placentas of ruminants favor the transport of ω-3 PUFAs to fetuses, especially in the first trimester of gestation. As organogenesis occurs primarily in the first trimester of gestation, supplementing pregnant heifers with sources of ω-3 PUFAs during this window may have beneficial effects in terms of supporting fetal growth and development and consequently neonatal performance. In addition, ω-3 PUFAs are well-known for their anti-inflammatory effects, playing a key role in reproductive performance and pregnancy maintenance [18]. Omega 3 PUFAs, especially EPA and DHA, have inhibitory effects on PGF2α, which is a pro-inflammatory molecule and exerts a luteolytic effect; thus, inhibition of PGF2α by ω-3 PUFAs may prevent regression of the corpus luteum (CL) and consequently prevent a decrease in the synthesis of progesterone (P4) and promote pregnancy maintenance. We have reported [11] that moderate rates of gain during the first 83 d of gestation resulted in larger and heavier CLs and greater concentrations of P4 compared to low rates of gain. Altogether, the findings of this and of our previous study [11] suggest a positive effect of ω-3 PUFAs in pregnancy maintenance. Whether or not fish oil supplementation affected ovary size and numbers of follicles in female fetuses in this study deserves future investigation.

Long-chain PUFAs are critical constituents of membrane phospholipids, including phosphatidylcholine (PC), phosphatidylethanolamine (PE), phosphatidylinositol (PI), phosphatidylglycerol (PG), and phosphatidylserine (PS) [19]. Abott et al. [20] demonstrated a steady-state relationship between dietary PUFAs and membrane phospholipid compositions in muscle, heart, liver, brain, and red blood cells in rats. Thus, the abundance of ω-3 and ω-6 PUFAs strongly influences their relative abundance in membrane phospholipids [20]. In the current study, GAIN affected the abundance of ω-3 and ω-6 PUFAs, levels of the former being greater in MG (as previously described) and levels of the latter being greater in LG. Consequently, the abundances of ω-3 and ω-6 PUFAs reflected the abundances and compositions of PC, PE, PI, PG, and PS in fetal livers. For instance, MG resulted in greater concentrations of DHA in fetal livers. DHA is a component of 1-palmitoyl-2-docosahexaenoyl-GPC (a phospholipid belonging to the PC class), concentrations of which were also greater in MG fetuses. This same pattern was observed for the various types of PC, PE, PI, PG, and PS identified in this study. It is important to highlight that even though the ratio of omega 3 to omega 6 is usually valuable, we were unable to use it here because it is a measure of relative abundances and not absolute concentrations.

Moderate rates of gain during the first 83 days of gestation also resulted in the accumulation of several diacylglycerols (DAGs) and the depletion of the majority of monoacylglycerols (MAGs). According to Xia and Coleman [21], the synthesis of DAGs is more complex in neonatal livers than in adult livers and other tissues. Diacylglycerols can be synthesized from the acylation of glycerol 3-phosphate; however, in the liver, the monoacylglycerol pathway is an additional pathway for diacylglycerol synthesis [22]. In fetal and neonatal livers, the activity of the enzyme monoacylglycerol acyltransferase is 700-fold greater than in adult livers. This enzyme utilizes MAGs to synthesize DAGs, ensuring the de novo synthesis of DAGs for membrane phospholipid biogenesis, and to synthesize triacylglycerols for VLDL production [21,22]. Thus, our results suggest that maternal moderate rates of gain during the first trimester of gestation alter lipid metabolism in fetal livers, most likely by increasing the hydrolysis of triacylglycerols to generate monoacylglycerols, which are then used as substrates for monoacylglycerol transferase, ultimately resulting in a greater accumulation of diacylglycerols.

The majority of acylcarnitines were affected by VMSUP × GAIN interactions, their concentrations being lower in VTM-LG than for all other combinations. This indicates a greater rate of complete oxidation of fatty acids. Fatty acid oxidation occurs mainly in the mitochondria, where, after a series of enzymatic reactions and translocations, acylcarnitine is transported into the mitochondrial matrix [23]. Once in the mitochondrial matrix, acylcarnitine is converted to acyl-CoA and free carnitine. Acyl-CoA is then available for β-oxidation, producing acetyl-CoA, which enters the TCA cycle. The TCA cycle generates electron donors, such as NADH and FADH2, supporting the transport of electrons into the electron transport chain, ultimately resulting in ATP production [23]. Thus, data from this study suggest that a combination of VTM supplementation and lower rates of gain during the first trimester of gestation may improve energy utilization in fetal livers. In a follow-up study, pregnant heifers targeted to gain 0.45 kg/d received (*n* = 7) or did not receive (*n* = 7) a VTM supplement throughout gestation. Calves were harvested 30 h after birth and samples of livers and small intestines were collected and evaluated for mitochondrial oxygen consumption [24]. Contrary to what we expected, we did not observe differences in energy metabolism between the livers of calves from control and VTM dams. Interestingly, greater respiratory rates in all mitochondrial respiratory states and a greater efficiency of energy utilization were observed in the small intestines of the VTM group compared to the control samples. When interpreting data from the current study, the lack of differences in mitochondrial energy metabolism between neonatal livers in the NoVTM and VTM groups may be explained by the fact that levels of the ketone body BHBA, which often serve as markers of changes in lipid oxidation, remained relatively constant across treatments, suggesting that even if there is a change in lipid oxidation rates, hepatocyte energy levels remain relatively stable, which is also supported by the lack of meaningful differences in glycolysis and TCA cycles [8]. Overall, these changes support the argument that supplementation may alter lipid metabolism in the liver and possibly other organs to a degree such that the liver can adapt and thus avoid major changes in energy metabolism.

Even in early gestation, fetal liver has the ability to synthesize primary bile acids [25]. It has been suggested that at this stage of fetal development bile acids act as signaling molecules with endocrine and paracrine functions, instead of serving as digestive surfactants [25]. Data from this study show that the pool of primary bile acids identified in fetal livers were not affected by maternal treatments. Primary bile acids are synthesized from cholesterol; thus, as expected, the abundances of cholesterol were also similar between the four treatments. Interestingly, levels of secondary bile acids were affected by VMSUP and to a lesser degree by GAIN. Secondary bile acids are produced by the action of gut bacteria on primary bile acids. The fetal intestine is considered sterile; thus, the primary source of secondary bile acids to the fetus is probably via placental transfer [25,26]. Concentrations of secondary bile acids are greater in adult livers, followed by fetal livers and neonatal livers, in sheep [26]. Data reported by Harvey et al. [26] suggest that after parturition, due to the cessation of placental transfer of secondary bile acids, concentrations of these metabolites decrease in the livers of neonates. Using the same experimental units from the current experiment (i.e., the same fetuses), we characterized the intestinal microbiome and, indeed, found that it was colonized at 83 d of gestation. We conducted 16S sequencing on frozen samples of allantoic and amniotic fluids, cotyledons, and fetal intestines [27]. Future work should expand on the 16S platform to include both aerobic and anaerobic culturing and shotgun metagenomic techniques for dam and fetus samples at different stages of gestation and for different diets to enhance our understanding of the establishment of the fetal microbiome. As the animal grows, the maturation of the gastrointestinal tract and its microbiota results in a greater production of secondary bile acids, especially deoxycholic acid. Thus, our results suggest that pregnant heifers not supplemented with VTM, as well as heifers fed to achieve low rates of gain (0.29 kg/d) or a combination of both (NoVTM-LG), undergo greater production of secondary bile acids, which in turn are transferred to the fetus. This may explain why we observed greater concentrations of glycoursodeoxycholate and tauroursodeoxycholate in NoVTM-LG fetuses as well as greater concentrations of deoxycholate, glycodeoxycholate, and taurodeoxycholate in fetal livers in response to NoVTM and a greater abundance of glycodeoxycholate in response to LG.

## 5. Conclusions

Our lipidomic fingerprint approach demonstrates that maternal vitamin and mineral supplementation and rate of gain in the first trimester of gestation influenced and altered lipid composition and lipid metabolism in fetal livers. Thirty-two lipid sub-pathways out of the 57 identified were affected by rates of gain; concentrations of the majority of the metabolites were greater in response to low gain (0.28 kg/d) than to moderate gain (0.79 kg/d), especially concentrations of monoacylglycerols. Further, a combination of VTM supplementation and low rates of gain resulted in lower concentrations of acylcarnitines, indicating a greater rate of complete oxidation of fatty acids and suggesting more efficient energy use in VTM-LG fetuses.

## Figures and Tables

**Table 1:** Enrichment scores for each sub-pathway identified. Sub-pathways with enrichment scores > 1 have more biochemicals/metabolites with statistically significant fold changes compared to all other sub-pathways within the study ^1^.

Sub-Pathway	VMSUP	GAIN	VMSUP × GAIN
Fatty acid synthesis	0	0	0
Fatty acid metabolism	0	0	0
Short-chain fatty acids	0	0	0
Medium-chain fatty acids	0	1.06	0
Long-chain saturated fatty acids	0	0	0
Long-chain monounsaturated fatty acids	0	1.6	0
Long-chain polyunsaturated fatty acids (ω3 and ω6)	0	2.84	0
Fatty acids, branched	0	0	0
Fatty acids, dicarboxylate	1.04	0.7	0.88
Fatty acids, amino	9.5	2.12	0
Fatty acid metabolism (also BCAA metabolism)	0	0.7	5.62
Fatty acid metabolism (acylglycine)	0	1.41	0
Fatty acid metabolism (acylcarnitine, short-chain)	0	0	5.35
Fatty acid metabolism (acylcarnitine, medium-chain)	0	0	8.32
Fatty acid metabolism (acylcarnitine, long-chain saturated)	0	0.6	0
Fatty acid metabolism (acylcarnitine, monounsaturated)	0	0	14.02
Fatty acid metabolism (acylcarnitine, polyunsaturated)	2.09	1.9	7.52
Fatty acid metabolism (acylcarnitine, dicarboxylate)	0	1.41	0
Fatty acid metabolism (acylcarnitine, hydroxy)	0	0	11.76
Carnitine metabolism	0	0	8.04
Ketone bodies	0	0	0
Fatty acid metabolism (acylcholine)	0	4.27	0
Fatty acids, monohydroxy	0	0.79	0
Fatty acids, dihydroxy	0	1.06	0
Eicosanoid	0	4.27	0
Endocannabinoid	0	1.06	0
Inositol metabolism	0	0	8.04
Phospholipid metabolism	1.88	0.84	1.59
Phosphatidylcholine (PC)	0	3.84	0
Phosphatidylethanolamine (PE)	0	3.8	0
Glycosyl PE	0	0	0
Phosphatidylserine (PS)	0	4.33	0
Phosphatidylglycerol (PG)	0	4.28	0
Phosphatidylinositol (PI)	0	4.33	0
Lysophospholipids	0	2.03	0
Plasmalogens	0	2.15	0
Lysoplasmalogens	3.79	2.56	0
Glycerolipid metabolism	0	0	5.35
Monoacylglycerols	0	3.19	0
Diacylglycerols	0	3.59	0
Sphingolipid synthesis	0	0	0
Dihydroceramides	0	0	0
Ceramides	5.91	2.15	0
Hexosylceramides (HCERs)	0	2.12	0
Lactosylceramides (LCERs)	0	0	0
Glycosphingolipid sulfates	0	4.25	0
Dihydrosphingomyelins	11.9	1.7	0
Sphingomyelins	0.74	2.29	0
Sphingosines	0	0	0
Mevalonate metabolism	0	0	0
Sterols	0	0	0
Pregnenolone steroids	0	0	0
Progestin steroids	0	0	0
Corticosteroids	0	0	0
Androgenic steroids	0	0	0
Primary bile acid metabolism	3.15	0	0
Secondary bile acid metabolism	11.51	1.42	3.61

^1^ Pathway enrichment was calculated with the MetaboLync Pathway Analysis software using the following formula: (km)/(nN), where k = the number of significant metabolites per pathway, m = the total number of detected metabolites per pathway, n = the number of significant metabolites in the study, and N = the total number of detected metabolites in the study. VMSUP = main effect of vitamin and mineral supplementation; GAIN = main effect of rate of gain.

**Table 2:** Statistical heat map of biochemicals/metabolites involved in lipid metabolism sub-pathways.

Sub-Pathway	Biochemical/Metabolite	Two-Way ANOVA Main Effects			Two-Way ANOVA Contrasts					
		VMSUP	GAIN	VMSUP × GAIN	NOVTM-MG	VTM-LG	NOVTM-MG	VTM-MG	VTM-MG	VTM-MG
					NOVTM-LG	NOVTM-LG	VTM-LG	NOVTM-LG	VTM-LG	NOVTM-MG
Fatty acid synthesis	Malonylcarnitine	0.6004	0.8518	0.5273	0.90	1.02	0.88	1.58	1.55	1.75
	Malonate	0.5443	0.7489	0.3604	0.92	0.88	1.05	0.94	1.07	1.02
Fatty acid metabolism	Acetyl CoA	0.9768	0.1678	0.3412	1.00	0.74	1.36	1.09	1.47	1.09
	Arachidonoyl CoA	0.9156	0.2020	0.7691	0.89	0.98	0.91	0.89	0.91	1.01
Short-chain fatty acids	Butyrate/isobutyrate (4:0)	0.5887	0.8982	0.7815	0.98	1.07	0.92	1.05	0.98	1.07
Medium-chain fatty acids	Caproate (6:0)	0.9770	0.7233	0.4823	1.00	1.06	0.95	0.90	0.85	0.90
	Heptanoate (7:0)	0.6258	0.1832	0.1356	**0.75**	0.90	0.84	0.91	1.02	1.22
	Caprylate (8:0)	0.6044	0.9963	0.7821	1.00	0.97	1.02	0.94	0.97	0.94
	Pelargonate (9:0)	0.6416	0.0264	0.7231	0.89	1.00	0.88	**0.81**	**0.81**	0.92
	Caprate (10:0)	0.4936	0.4288	0.8032	0.95	1.02	0.94	1.00	0.98	1.05
	(2 or 3)-decenoate (10:1n7 or n8)	0.6474	0.0038	0.2416	0.75	1.32	**0.57**	**0.67**	**0.51**	0.90
	10-undecenoate (11:1n1)	0.6329	0.4096	0.9492	0.91	1.13	0.81	0.94	0.83	1.03
	5-dodecenoate (12:1n7)	0.9263	0.6330	0.5255	0.90	0.93	0.97	0.93	1.00	1.04
Long-chain saturated fatty acids	Myristate (14:0)	0.5000	0.1252	0.9073	0.70	0.85	0.82	0.61	0.71	0.87
	Palmitate (16:0)	0.6729	0.0861	0.8515	0.68	0.90	0.76	0.63	0.70	0.92
	Margarate (17:0)	0.5368	0.9661	0.6397	0.98	0.95	1.03	0.85	0.90	0.87
	Stearate (18:0)	0.8565	0.2467	0.7560	0.83	0.96	0.86	0.79	0.82	0.95
	Nonadecanoate (19:0)	0.5739	0.4009	0.7070	1.07	0.96	1.12	0.96	1.00	0.90
	Arachidate (20:0)	0.8201	0.2809	0.7673	0.87	0.95	0.91	0.82	0.86	0.95
	Behenate (22:0) *	0.4666	0.3024	0.9745	0.83	0.98	0.85	0.90	0.92	1.08
Long-chain monounsaturated fatty acids	Myristoleate (14:1n5)	0.5608	0.1867	0.6784	0.79	0.90	0.87	0.64	0.71	0.82
	Palmitoleate (16:1n7)	0.4932	0.1151	0.9115	0.55	0.74	0.74	0.45	0.60	0.81
	10-heptadecenoate (17:1n7)	0.5256	0.1449	0.7139	0.64	0.84	0.76	0.52	0.62	0.82
	Oleate/vaccenate (18:1)	0.6802	0.0160	0.7475	0.51	0.84	0.61	**0.44**	**0.53**	0.87
	10-nonadecenoate (19:1n9)	0.4236	0.0375	0.5913	0.67	0.90	0.75	**0.55**	**0.62**	0.82
	Eicosenoate (20:1)	0.5678	0.0160	0.7268	0.56	0.84	0.67	**0.48**	**0.57**	0.86
	Erucate (22:1n9)	0.7258	0.0680	0.9463	0.76	0.97	0.79	0.78	0.80	1.02
	Nervonate (24:1n9) *	0.0852	0.7309	0.3901	0.71	1.05	0.67	1.07	1.02	**1.52**
Long-chain polyunsaturated fatty acids (n3 and n6)	Tetradecadienoate (14:2) *	0.3803	0.2895	0.3216	1.31	1.06	1.24	1.02	0.96	0.77
	Stearidonate (18:4n3)	0.3999	0.0000	0.7516	**3.35**	1.14	**2.94**	**2.39**	**2.09**	0.71
	Eicosapentaenoate (EPA; 20:5n3)	0.4090	0.0000	0.8541	**1.78**	0.51	**3.49**	**1.43**	**2.81**	0.81
	Heneicosapentaenoate (21:5n3)	0.5073	0.0009	0.6814	**1.78**	0.89	**2.00**	**1.36**	**1.53**	0.76
	Docosapentaenoate (n3 DPA; 22:5n3)	0.3120	0.0001	0.8582	**1.81**	0.68	**2.67**	**1.38**	**2.03**	0.76
	Docosahexaenoate (DHA; 22:6n3)	0.3888	0.0366	0.8576	**1.05**	0.64	**1.64**	0.86	1.34	0.82
	Nisinate (24:6n3)	0.1603	0.0036	0.7965	**1.39**	0.60	**2.32**	0.92	**1.53**	0.66
	Linoleate (18:2n6)	0.3011	0.6402	0.8975	0.88	0.73	1.19	0.67	0.92	0.77
	Linolenate (alpha or gamma; (18:3n3 or 6))	0.3865	0.2335	0.9315	0.58	0.70	0.83	0.45	0.65	0.78
	Dihomo-linoleate (20:2n6)	0.5078	0.0014	0.8063	**0.45**	0.79	**0.58**	**0.39**	**0.49**	0.86
	Dihomo-linolenate (20:3n3 or n6)	0.5639	0.9193	0.7138	0.86	0.84	1.02	0.71	0.84	0.83
	Arachidonate (20:4n6)	0.6282	0.2515	0.6036	0.73	0.85	0.86	0.59	0.70	0.81
	Docosatrienoate (22:3n6) *	0.7710	0.0000	0.7496	**0.24**	0.83	**0.29**	**0.21**	**0.26**	0.89
	Adrenate (22:4n6)	0.4252	0.0928	0.1825	0.79	1.09	0.73	**0.55**	**0.51**	0.70
	Docosapentaenoate (n6 DPA; 22:5n6)	0.9570	0.0000	0.2542	**0.27**	1.06	**0.26**	**0.22**	**0.21**	0.80
	Docosadienoate (22:2n6)	0.5684	0.0002	0.8519	**0.39**	0.77	**0.50**	**0.34**	**0.44**	0.88
	Mead acid (20:3n9)	0.9696	0.0000	0.9078	**0.17**	0.71	**0.24**	**0.18**	**0.25**	1.03
Fatty acids, branched	(14 or 15)-methylpalmitate (a17:0 or i17:0)	0.4007	0.1144	0.4969	0.79	0.93	0.84	**0.65**	0.69	0.82
	(16 or 17)-methylstearate (a19:0 or i19:0)	0.2502	0.0606	0.3566	0.86	0.97	0.90	**0.67**	**0.70**	0.78
Fatty acids, dicarboxylate	Dimethylmalonic acid	0.3117	0.0669	0.6302	**0.84**	0.91	0.92	**0.81**	0.89	0.96
	Glutarate (C5-DC)	0.6326	0.0597	0.1244	0.95	1.20	**0.79**	0.85	**0.71**	0.89
	3-methylglutarate/2-methylglutarate	0.0648	0.1524	0.5368	0.74	**0.73**	1.01	**0.66**	0.90	0.89
	2-hydroxyglutarate	0.1793	0.4479	0.6170	1.02	0.90	1.13	0.96	1.07	0.95
	Adipate (C6-DC)	0.5207	0.0887	0.4395	0.92	1.07	0.86	0.82	**0.76**	0.89
	2-hydroxyadipate	0.1913	0.1858	0.4830	0.96	0.94	1.03	**0.76**	0.81	0.78
	3-hydroxyadipate	0.0239	0.7663	0.0427	**0.77**	**0.54**	1.43	**0.67**	1.25	0.87
	Maleate	0.1617	0.2452	0.7165	0.89	0.87	1.02	**0.81**	0.93	0.91
	Heptenedioate (C7:1-DC) *	0.0966	0.0204	0.9125	**0.62**	0.63	0.98	**0.42**	0.66	0.68
	Azelate (C9-DC)	0.1005	0.9551	0.5058	1.19	0.75	1.59	0.54	0.73	0.46
	Sebacate (C10-DC)	0.0893	0.9199	0.5185	1.28	0.81	1.58	0.76	0.94	0.60
	Dodecanedioate (C12-DC)	0.2229	0.6367	0.7837	0.97	0.88	1.09	0.78	0.89	0.81
	Dodecadienoate (12:2) *	0.6373	0.1975	0.3624	0.98	1.22	0.80	0.74	0.61	0.76
	Tridecenedioate (C13:1-DC) *	0.4095	0.7256	0.7448	1.12	0.92	1.21	0.93	1.00	0.83
	Tetradecanedioate (C14-DC)	0.2515	0.2370	0.9284	0.93	0.93	1.00	0.83	0.89	0.89
	Hexadecanedioate (C16-DC)	0.3369	0.0262	0.3782	0.85	0.99	0.86	**0.73**	**0.75**	0.86
	Hexadecenedioate (C16:1-DC) *	0.6653	0.0048	0.7459	**0.53**	0.91	**0.59**	**0.53**	**0.59**	1.00
	Octadecenedioate (C18:1-DC)	0.7692	0.0951	0.7015	0.75	0.93	0.80	0.75	0.81	1.01
Fatty acids, amino	2-aminoheptanoate	0.0014	0.0026	0.4558	**0.76**	**1.67**	**0.46**	1.03	**0.62**	**1.36**
	2-aminooctanoate	0.1428	0.0774	0.1983	1.15	1.00	1.15	**1.55**	**1.56**	**1.35**
Fatty acid metabolism (also BCAA metabolism)	Butyrylcarnitine (C4)	0.9281	0.4535	0.5029	0.79	0.91	0.87	0.91	1.01	1.16
	Butyrylglycine	0.9088	0.0264	0.9910	0.83	1.05	**0.79**	0.82	0.79	1.00
	Propionyl CoA	0.2451	0.0759	0.0349	0.95	**0.68**	**1.40**	1.05	**1.54**	1.10
	Propionylcarnitine (C3)	0.5091	0.3533	0.0364	0.91	**0.82**	1.11	1.04	**1.28**	1.15
	Propionylglycine	0.5899	0.6840	0.4983	0.96	0.88	1.09	0.99	1.13	1.03
	Methylmalonate (MMA)	0.8604	0.7050	0.9735	1.05	0.99	1.06	1.02	1.03	0.97
Fatty acid metabolism (acylglycine)	Valerylglycine	0.8384	0.5304	0.8391	0.93	0.91	1.02	1.06	1.17	1.15
	N-palmitoylglycine	0.2440	0.1061	0.8956	0.76	0.98	0.78	**0.62**	0.63	0.81
	3-hydroxybutyroylglycine **	0.5337	0.0247	0.9878	0.76	0.91	0.83	**0.70**	0.77	0.93
Fatty acid metabolism (acylcarnitine, short-chain)	Acetylcarnitine (C2)	0.8733	0.3934	0.0740	0.89	0.81	1.09	1.09	**1.34**	1.23
	Valerylcarnitine (C5)	0.5451	0.7227	0.0198	0.79	**0.77**	1.03	0.99	**1.28**	1.25
	Isocaproylcarnitine	0.5431	0.2216	0.8707	0.84	0.90	0.93	0.84	0.94	1.01
Fatty acid metabolism (acylcarnitine, medium-chain)	Hexanoylcarnitine (C6)	0.7046	0.8879	0.0633	0.72	0.79	0.91	1.01	1.28	1.40
	Octanoylcarnitine (C8)	0.1911	0.7010	0.2878	0.84	1.37	0.62	1.31	0.96	1.55
	Cis-3,4-methyleneheptanoylcarnitine	0.5777	0.2007	0.0165	0.88	**0.76**	1.16	1.09	**1.44**	1.24
	Nonanoylcarnitine (C9)	0.4635	0.2712	0.0868	**0.54**	**0.59**	0.91	0.73	1.23	1.36
	Decanoylcarnitine (C10)	0.7974	0.9531	0.0339	0.79	0.79	1.01	1.04	1.32	**1.30**
	Laurylcarnitine (C12)	0.6370	0.2442	0.0297	0.87	**0.72**	1.21	1.07	**1.49**	1.23
Fatty acid metabolism (acylcarnitine, long-chain saturated)	Myristoylcarnitine (C14)	0.7586	0.0928	0.0704	0.95	0.82	1.16	1.26	**1.54**	1.32
	Pentadecanoylcarnitine (C15) *	0.9083	0.1994	0.0925	0.94	0.91	1.03	1.20	**1.32**	1.28
	Palmitoylcarnitine (C16)	0.8264	0.0853	0.0576	0.97	0.81	1.21	1.19	**1.47**	1.22
	Margaroylcarnitine (C17) *	0.7560	0.1333	0.0899	1.02	0.95	1.06	1.29	**1.35**	1.27
	Stearoylcarnitine (C18)	0.9925	0.0205	0.2125	1.17	0.96	**1.22**	**1.37**	**1.43**	1.17
	Arachidoylcarnitine (C20) *	0.9953	0.1654	0.1041	1.00	0.90	1.12	1.20	**1.34**	1.20
	Behenoylcarnitine (C22) *	0.5401	0.4048	0.0844	0.88	0.87	1.02	1.16	**1.34**	1.31
Fatty acid metabolism (acylcarnitine, monounsaturated)	Butenoylcarnitine (C4:1)	0.0544	0.3286	0.4740	0.78	**0.68**	1.15	**0.69**	1.03	0.89
	Cis-4-decenoylcarnitine (C10:1)	0.4645	0.6165	0.0029	**0.73**	**0.67**	1.10	1.01	**1.51**	**1.38**
	5-dodecenoylcarnitine (C12:1)	0.5969	0.8441	0.0302	**0.76**	**0.72**	1.06	0.92	1.29	1.22
	Myristoleoylcarnitine (C14:1) *	0.9435	0.8234	0.0374	0.75	0.76	0.99	1.01	1.33	1.34
	Palmitoleoylcarnitine (C16:1) *	0.8235	0.5589	0.0184	0.82	**0.77**	1.07	1.07	**1.39**	1.30
	Oleoylcarnitine (C18:1)	0.9007	0.4009	0.0300	0.86	**0.80**	1.07	1.11	**1.39**	1.30
	Eicosenoylcarnitine (C20:1) *	0.7054	0.0576	0.0731	1.00	0.91	1.10	1.39	**1.53**	1.39
	Erucoylcarnitine (C22:1) *	0.9852	0.6879	0.0448	0.84	0.83	1.01	1.09	**1.31**	1.30
	Nervonoylcarnitine (C24:1) *	0.8271	0.5109	0.0230	**0.71**	**0.75**	0.95	0.95	1.28	1.34
Fatty acid metabolism (acylcarnitine, polyunsaturated)	Linoleoylcarnitine (C18:2) *	0.1230	0.0186	0.0031	0.96	**0.67**	**1.43**	1.12	**1.68**	1.17
	Linolenoylcarnitine (C18:3) *	0.0381	0.4242	0.0758	**0.67**	**0.62**	1.09	**0.66**	1.07	0.99
	Dihomo-linoleoylcarnitine (C20:2) *	0.9602	0.9586	0.0067	**0.73**	**0.78**	0.93	1.03	**1.31**	**1.40**
	Arachidonoylcarnitine (C20:4)	0.1203	0.7051	0.3270	0.85	**0.83**	1.03	0.80	0.97	0.94
	Meadoylcarnitine (C20:3n9) *	0.7527	0.0000	0.6679	**0.23**	0.76	**0.30**	**0.29**	**0.39**	1.31
	Docosadienoylcarnitine (C22:2) *	0.9716	0.0496	0.0190	0.95	**0.78**	1.22	1.30	**1.67**	**1.37**
	Docosatrienoylcarnitine (C22:3) *	0.7205	0.2168	0.0480	0.91	**0.79**	1.15	1.15	**1.45**	1.26
	Adrenoylcarnitine (C22:4) *	0.6092	0.7802	0.1040	0.78	0.78	0.99	0.87	1.12	1.12
	Docosahexaenoylcarnitine (C22:6) *	0.0842	0.0005	0.1086	1.26	**0.66**	**1.92**	1.29	**1.96**	1.02
Fatty acid metabolism (acylcarnitine, dicarboxylate)	Adipoylcarnitine (C6-DC)	0.7234	0.0557	0.3213	**0.67**	0.96	0.70	0.88	0.92	1.31
	Pimeloylcarnitine/3-methyladipoylcarnitine (C7-DC)	0.8979	0.4490	0.0975	**0.73**	0.85	0.86	0.88	1.04	1.22
	Suberoylcarnitine (C8-DC)	0.9730	0.0363	0.1095	**0.47**	0.74	0.63	0.72	0.97	1.55
Fatty acid metabolism (acylcarnitine, hydroxy)	(R)-3-hydroxybutyrylcarnitine	0.8627	0.3078	0.0552	**0.68**	0.80	0.86	1.00	1.25	1.46
	(S)-3-hydroxybutyrylcarnitine	0.7181	0.2611	0.0018	0.85	**0.77**	1.11	1.08	**1.41**	**1.27**
	3-hydroxyhexanoylcarnitine (1)	0.9194	0.2380	0.0001	**0.67**	**0.76**	0.89	0.93	**1.23**	**1.38**
	3-hydroxyhexanoylcarnitine (2)	0.1519	0.0652	0.0019	**0.54**	0.74	**0.73**	0.96	1.28	**1.76**
	3-hydroxyoctanoylcarnitine (1)	0.3025	0.1893	0.0014	0.81	**0.82**	1.00	1.27	**1.55**	**1.55**
	3-hydroxyoctanoylcarnitine (2)	0.6963	0.5048	0.0039	**0.81**	**0.81**	1.01	1.14	**1.41**	**1.40**
	3-hydroxydecanoylcarnitine	0.5116	0.9289	0.2127	0.75	0.87	0.86	1.06	1.21	1.40
	3-hydroxypalmitoylcarnitine	0.6775	0.1181	0.0355	0.95	**0.79**	1.20	1.22	**1.54**	1.29
	3-hydroxyoleoylcarnitine	0.5178	0.0515	0.1233	1.07	0.93	1.15	**1.61**	**1.73**	1.50
Carnitine metabolism	Deoxycarnitine	0.1141	0.2280	0.5365	1.08	0.83	**1.30**	0.98	1.18	0.91
	Carnitine	0.0909	0.9439	0.0204	**0.83**	**0.76**	1.10	0.88	1.16	1.06
Ketone bodies	3-hydroxybutyrate (BHBA)	0.6675	0.7372	0.7903	0.97	0.95	1.02	0.94	0.99	0.97
Fatty acid metabolism (acylcholine)	Palmitoylcholine	0.1459	0.0326	0.1197	**1.52**	**1.47**	1.04	**1.53**	1.05	1.01
	Oleoylcholine	0.7233	0.0086	0.7897	**1.31**	1.01	**1.29**	**1.37**	**1.35**	1.05
Fatty acids, monohydroxy	4-hydroxybutyrate (GHB)	0.8116	0.6099	0.1131	0.69	0.74	0.93	0.84	1.13	1.21
	Alpha-hydroxycaproate	0.1075	0.9005	0.9005	1.00	1.05	0.95	1.05	1.00	1.05
	2-hydroxyoctanoate	0.2365	0.0120	0.2924	**0.75**	1.02	**0.74**	0.89	0.87	1.18
	2-hydroxydecanoate	0.2028	0.0048	0.1200	**0.65**	0.98	**0.66**	0.87	0.89	**1.35**
	2-hydroxymyristate	0.4475	0.2611	0.2097	1.03	1.12	0.92	0.72	0.64	0.69
	2-hydroxypalmitate	0.7918	0.1275	0.4104	0.87	1.20	0.73	0.70	0.58	0.80
	2-hydroxystearate	0.7508	0.6297	0.5886	1.08	1.42	0.76	0.99	0.69	0.91
	2-hydroxyarachidate *	0.9633	0.7793	0.6617	1.04	1.16	0.90	0.97	0.84	0.93
	2-hydroxybehenate	0.9160	0.1930	0.5469	0.79	0.91	0.87	0.83	0.91	1.04
	3-hydroxyhexanoate	0.7934	0.9249	0.3906	1.11	1.10	1.01	0.96	0.88	0.86
	3-hydroxyoctanoate	0.7504	0.1593	0.8119	0.86	0.94	0.91	0.83	0.89	0.97
	3-hydroxydecanoate	0.9002	0.4745	0.0864	**0.80**	0.85	0.94	0.92	1.09	1.16
	3-hydroxylaurate	0.1474	0.0099	0.4749	**0.81**	1.05	**0.77**	0.92	0.87	1.13
	5-hydroxyhexanoate	0.3672	0.5997	0.9160	1.10	0.84	1.32	0.89	1.06	0.80
	16-hydroxypalmitate	0.1676	0.7964	0.8239	1.01	0.93	1.09	0.83	0.90	0.83
	13-hode + 9-hode	0.7877	0.0984	0.4126	**1.71**	1.48	1.16	1.39	0.94	0.81
Fatty acids, dihydroxy	2S,3R-dihydroxybutyrate	0.5167	0.8633	0.4873	1.07	0.96	1.12	0.96	1.00	0.89
	2R,3R-dihydroxybutyrate	0.3508	0.2847	0.4082	1.02	1.01	1.01	1.17	1.16	1.15
	2,4-dihydroxybutyrate	0.7690	0.5147	0.4917	1.00	0.96	1.05	1.02	1.07	1.02
	3,4-dihydroxybutyrate	0.1383	0.0384	0.2939	0.93	**1.24**	**0.75**	0.96	**0.77**	1.03
Eicosanoids	Prostaglandin f2alpha	0.6023	0.0030	0.0626	0.72	1.39	**0.52**	**0.45**	**0.33**	**0.63**
	12-hhtre	0.8044	0.0326	0.0895	0.83	1.46	0.57	**0.53**	**0.36**	0.64
Endocannabinoids	Oleoyl ethanolamide	0.9094	0.1466	0.8239	0.78	0.94	0.83	0.78	0.83	1.00
	Palmitoyl ethanolamide	0.7190	0.3233	0.6466	0.69	0.94	0.73	0.93	0.98	1.34
	Stearoyl ethanolamide	0.9709	0.2919	0.7912	0.71	0.97	0.73	0.87	0.90	1.23
	N-oleoyltaurine	0.8165	0.0815	0.5691	**0.63**	0.94	0.68	0.72	0.77	1.14
	N-stearoyltaurine	0.3861	0.4891	0.1655	0.94	0.97	0.97	1.16	1.20	1.23
	N-palmitoyltaurine	0.8423	0.5078	0.4199	0.75	0.90	0.83	0.94	1.04	1.25
	Linoleoyl ethanolamide	0.6780	0.0131	0.6478	**1.52**	1.07	1.42	**1.42**	1.32	0.93
	N-oleoylserine	0.5554	0.0137	0.8414	**0.69**	1.01	0.68	**0.61**	**0.60**	0.88
Inositol metabolism	Myo-inositol	0.1377	0.3032	0.0230	**1.10**	**1.11**	0.99	**1.07**	0.97	0.98
	Inositol 1-phosphate (I1P)	0.1155	0.2348	0.1737	**1.31**	**1.37**	0.96	**1.36**	0.99	1.04
Phospholipid metabolism	Choline	0.9599	0.3619	0.3887	1.08	1.05	1.03	1.04	0.99	0.96
	Choline phosphate	0.1022	0.1581	0.0179	0.95	**0.82**	**1.16**	0.99	**1.20**	1.04
	Cytidine 5’-diphosphocholine	0.9928	0.1589	0.6050	1.04	0.98	1.06	1.06	1.08	1.02
	Glycerophosphorylcholine (GPC)	0.8618	0.1272	0.8789	1.24	1.08	1.15	1.18	1.09	0.95
	Phosphoethanolamine	0.3653	0.1299	0.8833	1.04	1.03	1.01	**1.06**	1.03	1.02
	Cytidine-5’-diphosphoethanolamine	0.5655	0.7751	0.9412	1.02	1.03	0.99	1.03	1.01	1.02
	Glycerophosphoethanolamine	0.9475	0.4546	0.7497	1.08	1.05	1.03	1.05	1.00	0.97
	Glycerophosphoserine *	0.6111	0.4879	0.9035	0.95	1.03	0.92	1.00	0.97	1.05
	Glycerophosphoinositol *	0.2234	0.0455	0.5762	1.06	1.02	1.03	**1.16**	**1.13**	1.09
	Trimethylamine N-oxide	0.0037	0.0008	0.5075	**1.70**	**0.69**	**2.46**	1.01	**1.47**	**0.60**
Phosphatidylcholine (PC)	1-myristoyl-2-palmitoyl-GPC (14:0/16:0)	0.3561	0.5841	0.2342	1.02	1.08	0.94	1.01	0.93	0.99
	1-myristoyl-2-arachidonoyl-GPC (14:0/20:4) *	0.6679	0.0000	0.7972	**0.72**	1.00	**0.72**	**0.75**	**0.75**	1.04
	1,2-dipalmitoyl-GPC (16:0/16:0)	0.7330	0.0000	0.7725	**1.24**	1.02	**1.22**	**1.24**	**1.22**	1.00
	1-palmitoyl-2-palmitoleoyl-GPC (16:0/16:1) *	0.2708	0.0000	0.3753	**0.83**	1.01	**0.82**	**0.87**	**0.87**	1.06
	1-palmitoyl-2-stearoyl-GPC (16:0/18:0)	0.6714	0.0000	0.6388	**1.27**	0.98	**1.30**	**1.35**	**1.38**	1.06
	1-palmitoyl-2-oleoyl-GPC (16:0/18:1)	0.1518	0.0000	0.5300	**0.87**	1.02	**0.85**	**0.91**	**0.89**	1.05
	1-palmitoyl-2-gamma-linolenoyl-GPC (16:0/18:3n6) *	0.3702	0.0000	0.5711	**0.41**	0.95	**0.43**	**0.48**	**0.50**	1.17
	1-palmitoyl-2-dihomo-linolenoyl-GPC (16:0/20:3n3 or 6) *	0.2972	0.0726	0.4292	**0.81**	1.02	**0.80**	0.93	0.91	1.14
	1-palmitoyl-2-arachidonoyl-GPC (16:0/20:4n6)	0.1287	0.0000	0.7434	**0.87**	1.04	**0.83**	**0.89**	**0.85**	1.03
	1-palmitoyl-2-docosahexaenoyl-GPC (16:0/22:6)	0.8906	0.0000	0.5424	**1.35**	0.91	**1.47**	**1.39**	**1.52**	1.03
	1-palmitoleoyl-2-linoleoyl-GPC (16:1/18:2) *	0.0665	0.0000	0.9032	**1.58**	0.89	**1.77**	**1.42**	**1.60**	0.90
	1,2-distearoyl-GPC (18:0/18:0)	0.9876	0.0457	0.4202	1.18	0.88	1.34	1.33	**1.51**	1.13
	1-stearoyl-2-oleoyl-GPC (18:0/18:1)	0.4845	0.0000	0.5561	**0.78**	1.00	**0.78**	**0.82**	**0.82**	1.05
	1-stearoyl-2-linoleoyl-GPC (18:0/18:2) *	0.9411	0.0051	0.3686	1.12	0.96	**1.17**	**1.19**	**1.23**	1.06
	1-stearoyl-2-arachidonoyl-GPC (18:0/20:4)	0.5618	0.2054	0.8176	0.96	1.04	0.92	0.97	0.94	1.02
	1-stearoyl-2-docosahexaenoyl-GPC (18:0/22:6)	0.9066	0.0000	0.8990	**1.60**	0.93	**1.71**	**1.64**	**1.75**	1.02
	1,2-dioleoyl-GPC (18:1/18:1)	0.4207	0.0000	0.8948	**0.76**	1.03	**0.74**	**0.80**	**0.78**	1.05
	1-oleoyl-2-linoleoyl-GPC (18:1/18:2) *	0.1313	0.0000	0.8179	**1.64**	0.92	**1.78**	**1.48**	**1.60**	0.90
	1-oleoyl-2-docosahexaenoyl-GPC (18:1/22:6) *	0.5895	0.0006	0.1961	**1.12**	0.90	**1.23**	**1.16**	**1.28**	1.04
	1,2-dilinoleoyl-GPC (18:2/18:2)	0.7830	0.0000	0.6875	**2.00**	0.96	**2.08**	**1.99**	**2.07**	0.99
Phosphatidylethanolamine (PE)	1,2-dipalmitoyl-GPE (16:0/16:0) *	0.7358	0.8622	0.9129	1.03	1.03	1.00	1.07	1.04	1.04
	1-palmitoyl-2-stearoyl-GPE (16:0/18:0) *	0.9845	0.0300	0.9663	1.14	1.00	1.15	1.16	1.17	1.02
	1-palmitoyl-2-oleoyl-GPE (16:0/18:1)	0.2565	0.0000	0.4176	**0.83**	1.01	**0.82**	**0.88**	**0.87**	1.06
	1-palmitoyl-2-linoleoyl-GPE (16:0/18:2)	0.4468	0.0095	0.2784	1.16	0.87	**1.33**	1.19	**1.36**	1.02
	1-palmitoyl-2-arachidonoyl-GPE (16:0/20:4) *	0.1461	0.0000	0.6211	**0.74**	1.08	**0.68**	**0.77**	**0.71**	1.05
	1-palmitoyl-2-docosahexaenoyl-GPE (16:0/22:6) *	0.9645	0.0000	0.7580	**1.44**	0.92	**1.57**	**1.48**	**1.61**	1.03
	1-palmitoleoyl-2-oleoyl-GPE (16:1/18:1) *	0.2364	0.0001	0.7637	**0.82**	1.08	**0.76**	**0.86**	**0.79**	1.04
	1-stearoyl-2-oleoyl-GPE (18:0/18:1)	0.2978	0.0005	0.9237	**0.88**	1.03	**0.85**	**0.91**	**0.88**	1.04
	1-stearoyl-2-arachidonoyl-GPE (18:0/20:4)	0.1131	0.0002	0.5295	**0.90**	1.07	**0.84**	**0.93**	**0.86**	1.03
	1-stearoyl-2-docosahexaenoyl-GPE (18:0/22:6) *	0.8920	0.0000	0.8308	**1.72**	0.92	**1.86**	**1.67**	**1.81**	0.97
	1,2-dioleoyl-GPE (18:1/18:1)	0.1817	0.0031	0.9417	**0.91**	1.04	**0.88**	0.95	**0.91**	1.04
	1-oleoyl-2-linoleoyl-GPE (18:1/18:2) *	0.8821	0.0000	0.9603	**1.44**	1.02	**1.42**	**1.46**	**1.44**	1.01
	1-oleoyl-2-arachidonoyl-GPE (18:1/20:4) *	0.3065	0.0972	0.3784	0.97	1.07	**0.91**	0.98	**0.92**	1.01
	1-oleoyl-2-docosahexaenoyl-GPE (18:1/22:6) *	0.6848	0.0000	0.6966	**1.37**	0.92	**1.48**	**1.36**	**1.47**	0.99
Glycosyl PE	1-stearoyl-2-arachidonoyl-glycosyl-GPE (18:0/20:4) **	0.4087	0.3792	0.9598	0.82	0.87	0.94	0.66	0.75	0.80
Phosphatidylserine (PS)	1-palmitoyl-2-oleoyl-GPS (16:0/18:1)	0.8683	0.0000	0.2023	**0.77**	0.95	**0.80**	**0.81**	**0.85**	1.05
	1-palmitoyl-2-arachidonoyl-GPS (16:0/20:4)	0.9593	0.0000	0.7717	**0.67**	1.02	**0.66**	**0.67**	**0.65**	0.99
	1-stearoyl-2-oleoyl-GPS (18:0/18:1)	0.1506	0.0000	0.8796	**0.85**	1.04	**0.82**	**0.90**	**0.86**	1.05
	1-stearoyl-2-linoleoyl-GPS (18:0/18:2)	0.2535	0.0000	0.7802	**1.35**	1.06	**1.27**	**1.48**	**1.39**	1.09
	1-stearoyl-2-arachidonoyl-GPS (18:0/20:4)	0.3786	0.0000	0.8797	**0.75**	1.05	**0.71**	**0.77**	**0.74**	1.04
	1,2-dioleoyl-GPS (18:1/18:1)	0.7721	0.0000	0.4442	**0.72**	1.02	**0.70**	**0.67**	**0.66**	0.94
Phosphatidylglycerol (PG)	1-palmitoyl-2-oleoyl-GPG (16:0/18:1)	0.9840	0.0380	0.2289	**0.92**	0.97	0.95	0.95	0.98	1.03
	1,2-dioleoyl-GPG (18:1/18:1)	0.2582	0.0000	0.8505	**0.75**	1.08	**0.69**	**0.79**	**0.73**	1.06
	1-oleoyl-2-linoleoyl-GPG (18:1/18:2) *	0.5862	0.0058	0.9988	**0.83**	0.96	**0.86**	**0.81**	**0.84**	0.98
Phosphatidylinositol (PI)	1-palmitoyl-2-oleoyl-GPI (16:0/18:1) *	0.6275	0.0115	0.4710	**0.80**	0.92	0.87	**0.83**	0.90	1.03
	1-palmitoyl-2-arachidonoyl-GPI (16:0/20:4) *	0.6278	0.0000	0.9948	**1.41**	0.94	**1.49**	**1.37**	**1.45**	0.97
	1-stearoyl-2-oleoyl-GPI (18:0/18:1) *	0.5560	0.0016	0.8030	**0.80**	0.97	**0.83**	**0.77**	**0.79**	0.96
	1,2-dioleoyl-GPI (18:1/18:1)	0.7170	0.0005	0.3205	**0.76**	0.97	**0.78**	**0.82**	**0.85**	1.08
	1-stearoyl-2-arachidonoyl-GPI (18:0/20:4)	0.8770	0.0002	0.7883	**1.34**	1.00	**1.35**	**1.32**	**1.32**	0.98
	1-oleoyl-2-arachidonoyl-GPI (18:1/20:4) *	0.8533	0.0015	0.9906	**1.16**	0.97	**1.19**	**1.16**	**1.19**	1.00
Lysophospholipids	1-palmitoyl-GPC (16:0)	0.9601	0.6973	0.3794	1.09	1.05	1.04	1.02	0.97	0.94
	2-palmitoyl-GPC (16:0) *	0.9705	0.6633	0.9662	1.02	1.01	1.01	1.03	1.03	1.01
	1-palmitoleoyl-GPC (16:1) *	0.9376	0.0000	0.2738	**0.69**	0.93	**0.74**	**0.73**	**0.78**	1.06
	2-palmitoleoyl-GPC (16:1) *	0.2149	0.3072	0.6418	0.92	1.18	0.78	0.98	0.84	1.07
	1-stearoyl-GPC (18:0)	0.7084	0.5430	0.6154	1.12	1.02	1.10	1.02	1.00	0.91
	1-oleoyl-GPC (18:1)	0.7848	0.0017	0.3856	**0.89**	1.07	**0.84**	**0.86**	**0.81**	0.96
	1-linoleoyl-GPC (18:2)	0.8047	0.0199	0.6611	1.13	0.98	**1.15**	1.14	**1.16**	1.01
	1-arachidonoyl-GPC (20:4n6) *	0.5302	0.0136	0.9045	**0.79**	1.10	**0.72**	0.82	**0.74**	1.04
	1-lignoceroyl-GPC (24:0)	0.4129	0.0461	0.8786	1.29	1.12	1.15	**1.37**	1.22	1.06
	1-palmitoyl-GPE (16:0)	0.6554	0.7138	0.3483	1.22	1.06	1.15	1.00	0.94	0.82
	1-stearoyl-GPE (18:0)	0.6009	0.9696	0.1588	1.17	1.10	1.07	0.96	0.87	0.82
	2-stearoyl-GPE (18:0) *	0.6479	0.4031	0.6712	0.95	1.01	0.94	0.86	0.85	0.90
	1-oleoyl-GPE (18:1)	0.8123	0.0021	0.3008	0.87	1.05	**0.83**	**0.80**	**0.76**	0.92
	1-linoleoyl-GPE (18:2) *	0.6995	0.1151	0.7909	1.13	1.00	1.14	1.08	1.08	0.95
	1-arachidonoyl-GPE (20:4n6) *	0.6123	0.0005	0.6641	**0.75**	1.07	**0.71**	**0.75**	**0.70**	0.99
	1-palmitoyl-GPS (16:0) *	0.8295	0.9758	0.7066	1.11	1.08	1.02	0.99	0.91	0.89
	1-stearoyl-GPS (18:0) *	0.7907	0.8350	0.6106	1.17	1.12	1.04	0.99	0.88	0.85
	1-oleoyl-GPS (18:1)	0.9824	0.0085	0.6728	**0.77**	1.04	**0.74**	**0.73**	**0.70**	0.95
	1-linoleoyl-GPS (18:2) *	0.9442	0.9898	0.9252	1.02	1.03	1.00	0.96	0.93	0.94
	1-palmitoyl-GPG (16:0) *	0.8206	0.0003	0.9443	**0.73**	1.02	**0.72**	**0.75**	**0.73**	1.02
	1-stearoyl-GPG (18:0)	0.7228	0.0020	0.9705	**0.70**	1.05	**0.67**	**0.73**	**0.70**	1.04
	1-oleoyl-GPG (18:1) *	0.5069	0.0002	0.9106	**0.68**	1.05	**0.65**	**0.71**	**0.68**	1.05
	1-linoleoyl-GPG (18:2) *	0.3904	0.0243	0.3089	0.89	1.19	**0.75**	0.89	**0.74**	0.99
	1-palmitoyl-GPI (16:0)	0.5653	0.4736	0.8365	1.02	0.91	1.13	0.98	1.08	0.96
	1-stearoyl-GPI (18:0)	0.3707	0.2685	0.9405	1.06	0.89	1.19	0.97	1.08	0.91
	1-oleoyl-GPI (18:1)	0.8753	0.1389	0.9535	0.82	0.94	0.88	0.82	0.87	0.99
	1-linoleoyl-GPI (18:2) *	0.2441	0.5539	0.3441	0.91	0.76	1.19	0.87	1.14	0.96
	1-arachidonoyl-GPI (20:4) *	0.7031	0.0043	0.6038	**1.46**	1.01	**1.45**	**1.30**	**1.29**	0.89
Plasmalogens	1-(1-enyl-palmitoyl)-2-oleoyl-GPE (P-16:0/18:1) *	0.0958	0.0005	0.8008	**0.83**	1.07	**0.78**	0.91	**0.84**	1.09
	1-(1-enyl-palmitoyl)-2-linoleoyl-GPE (P-16:0/18:2) *	0.8964	0.3579	0.8726	0.92	0.98	0.93	0.93	0.95	1.01
	1-(1-enyl-palmitoyl)-2-palmitoyl-GPC (P-16:0/16:0) *	0.1590	0.0757	0.9017	1.08	1.06	1.02	**1.13**	1.07	1.05
	1-(1-enyl-palmitoyl)-2-palmitoleoyl-GPC (P-16:0/16:1) *	0.0683	0.0084	0.7032	**0.92**	1.09	**0.84**	0.97	**0.89**	1.06
	1-(1-enyl-palmitoyl)-2-arachidonoyl-GPE (P-16:0/20:4) *	0.4771	0.0000	0.3667	**0.87**	1.05	**0.83**	**0.87**	**0.83**	1.00
	1-(1-enyl-palmitoyl)-2-oleoyl-GPC (P-16:0/18:1) *	0.1932	0.7206	0.6543	0.97	1.03	0.94	1.03	1.00	1.07
	1-(1-enyl-stearoyl)-2-oleoyl-GPE (P-18:0/18:1)	0.5104	0.0156	0.4498	**0.85**	1.00	**0.85**	0.92	0.91	1.08
	1-(1-enyl-palmitoyl)-2-arachidonoyl-GPC (P-16:0/20:4) *	0.6899	0.0576	0.6443	0.82	1.12	**0.73**	0.81	**0.73**	0.99
	1-(1-enyl-palmitoyl)-2-linoleoyl-GPC (P-16:0/18:2) *	0.9268	0.0401	0.7119	1.12	0.96	1.17	1.22	**1.27**	1.09
	1-(1-enyl-stearoyl)-2-arachidonoyl-GPE (P-18:0/20:4) *	0.5418	0.6522	0.2800	1.02	1.08	0.94	1.00	0.93	0.98
Lysoplasmalogens	1-(1-enyl-palmitoyl)-GPC (P-16:0) *	0.3902	0.0640	0.9207	0.82	1.04	**0.79**	0.87	0.84	1.06
	1-(1-enyl-palmitoyl)-GPE (P-16:0) *	0.0633	0.6736	0.9764	1.02	1.09	0.94	1.11	1.02	1.08
	1-(1-enyl-oleoyl)-GPE (P-18:1) *	0.0370	0.0002	0.6929	**0.86**	**1.11**	**0.77**	0.92	**0.83**	1.07
	1-(1-enyl-stearoyl)-GPE (P-18:0) *	0.1886	0.0406	0.4377	**1.22**	1.14	1.07	**1.25**	1.10	1.02
	1-(1-enyl-oleoyl)-2-oleoyl-GPE (P-18:1/18:1) *	0.0864	0.0001	0.5478	**0.74**	1.09	**0.67**	**0.85**	**0.78**	1.15
Glycerolipid metabolism	Glycerol	0.9808	0.4089	0.3804	1.01	1.07	0.95	0.95	0.89	0.94
	Glycerol 3-phosphate	0.2183	0.4502	0.0184	**1.10**	**1.11**	0.99	1.06	0.95	0.97
	Glycerophosphoglycerol	0.7437	0.2120	0.5403	1.08	1.01	1.07	1.04	1.03	0.96
Monoacylglycerols	1-myristoylglycerol (14:0)	0.6141	0.0134	0.8990	**0.68**	0.89	0.76	**0.61**	**0.69**	0.91
	1-pentadecanoylglycerol (15:0)	0.6497	0.4673	0.8707	0.84	0.91	0.93	0.80	0.88	0.95
	1-palmitoylglycerol (16:0)	0.3573	0.0546	0.7489	0.64	0.81	0.80	**0.59**	0.73	0.92
	1-palmitoleoylglycerol (16:1) *	0.9992	0.0008	0.8053	**0.56**	0.89	**0.63**	**0.56**	**0.63**	1.00
	1-margaroylglycerol (17:0)	0.6545	0.3830	0.7769	0.79	0.91	0.86	0.70	0.77	0.89
	1-oleoylglycerol (18:1)	0.8032	0.0006	0.6163	**0.53**	0.85	**0.62**	**0.54**	**0.64**	1.02
	1-linoleoylglycerol (18:2)	0.6227	0.1609	0.6313	1.09	0.91	1.20	1.06	1.16	0.97
	2-dihomo-linoleoylglycerol (20:2) *	0.2792	0.0000	0.1605	**0.37**	0.86	**0.43**	**0.52**	**0.61**	**1.41**
	1-dihomo-linolenylglycerol (20:3)	0.8299	0.0027	0.3428	**0.59**	0.89	**0.67**	**0.66**	0.74	1.12
	1-arachidonylglycerol (20:4)	0.3055	0.0007	0.6073	**0.76**	1.16	**0.66**	**0.77**	**0.66**	1.01
	1-eicosapentaenoylglycerol (20:5) *	0.5530	0.0000	0.6505	**1.96**	0.93	**2.10**	**2.19**	**2.36**	1.12
	1-docosahexaenoylglycerol (22:6)	0.9954	0.0135	0.9064	**1.21**	0.90	**1.35**	**1.21**	**1.35**	1.00
	2-myristoylglycerol (14:0)	0.1813	0.0091	0.5991	**0.52**	0.68	0.77	**0.44**	0.65	0.84
	2-palmitoylglycerol (16:0)	0.2985	0.0733	0.5215	**0.57**	0.73	0.78	**0.54**	0.73	0.93
	2-palmitoleoylglycerol (16:1) *	0.8137	0.0314	0.9794	0.49	0.79	**0.62**	0.50	0.63	1.03
	2-oleoylglycerol (18:1)	0.6260	0.0008	0.2829	**0.45**	0.72	**0.62**	**0.49**	**0.68**	1.09
	2-linoleoylglycerol (18:2)	0.4619	0.1273	0.7613	1.08	0.85	1.27	1.01	1.19	0.94
	2-arachidonoylglycerol (20:4)	0.5832	0.0028	0.9459	**0.69**	1.01	**0.68**	**0.71**	**0.70**	1.03
	2-eicosapentaenoylglycerol (20:5) *	0.6876	0.0003	0.2144	**1.24**	0.50	**2.51**	**1.37**	**2.76**	1.10
	2-docosahexaenoylglycerol (22:6) *	0.7330	0.1193	0.8152	1.00	0.76	1.31	0.98	1.29	0.98
	1-heptadecenoylglycerol (17:1) *	0.6824	0.0217	0.5708	**0.65**	0.86	0.75	**0.65**	0.75	0.99
	2-heptadecenoylglycerol (17:1) *	0.4905	0.0077	0.6253	**0.62**	0.81	0.77	**0.61**	0.75	0.98
	1-meadoylglycerol (20:3n9) *	0.5873	0.0000	0.3510	**0.19**	0.75	**0.25**	**0.24**	**0.32**	1.29
	1-dihomo-linoleoylglycerol (20:2)	0.0714	0.0012	0.7567	**0.36**	0.99	**0.37**	0.50	**0.51**	1.38
Diacylglycerols	Palmitoyl-arachidonoyl-glycerol (16:0/20:4) [2] *	0.2181	0.9591	0.6301	1.03	0.97	1.07	0.92	0.95	0.89
	Palmitoyl-docosahexaenoyl-glycerol (16:0/22:6) [1] *	0.7665	0.0014	0.9403	**1.77**	0.81	**2.18**	**1.84**	**2.26**	1.04
	Palmitoyl-docosahexaenoyl-glycerol (16:0/22:6) [2] *	0.3139	0.0000	0.7097	**1.70**	0.82	**2.08**	**1.60**	**1.95**	0.94
	Stearoyl-arachidonoyl-glycerol (18:0/20:4) [2] *	0.9263	0.0065	0.6736	**1.52**	1.07	**1.42**	**1.49**	**1.40**	0.98
	Oleoyl-arachidonoyl-glycerol (18:1/20:4) [2] *	0.4042	0.0000	0.5709	**1.48**	0.96	**1.53**	**1.33**	**1.38**	0.90
	Stearoyl-docosahexaenoyl-glycerol (18:0/22:6) [2] *	0.7638	0.0000	0.8013	**1.86**	0.96	**1.94**	**1.87**	**1.95**	1.00
Sphingolipid synthesis	Sphinganine	0.5757	0.0611	0.2360	0.97	1.11	**0.87**	0.93	**0.84**	0.96
	Sphingadienine	0.6137	0.4524	0.3749	1.01	1.04	0.97	0.94	0.90	0.93
	Phytosphingosine	0.9510	0.8231	0.8518	1.02	0.99	1.03	1.07	1.08	1.05
	Hexadecasphinganine (d16:0) *	0.0784	0.0814	0.9933	1.23	1.24	0.99	**1.58**	1.27	1.29
Dihydroceramides	N-palmitoyl-sphinganine (d18:0/16:0)	0.7620	0.1795	0.7904	1.06	1.00	1.05	1.09	1.09	1.03
Ceramides	N-palmitoyl-sphingosine (d18:1/16:0)	0.4692	0.3766	0.8502	0.96	0.98	0.98	0.91	0.93	0.95
	N-stearoyl-sphingosine (d18:1/18:0) *	0.5133	0.0255	0.7833	0.86	1.06	**0.81**	0.87	**0.82**	1.01
	N-palmitoyl-sphingadienine (d18:2/16:0) *	0.0135	0.7735	0.5594	1.05	0.89	**1.19**	0.86	0.97	**0.82**
	N-stearoyl-sphingadienine (d18:2/18:0) *	0.0459	0.6873	0.5790	1.01	0.92	1.09	**0.86**	0.94	**0.86**
	N-behenoyl-sphingadienine (d18:2/22:0) *	0.0295	0.4580	0.2937	0.92	**0.85**	1.07	**0.86**	1.00	0.94
	Ceramide (d18:1/14:0, d16:1/16:0) *	0.0819	0.2754	0.7006	1.07	0.94	**1.14**	0.97	1.03	0.91
	Ceramide (d18:1/17:0, d17:1/18:0) *	0.8903	0.0170	0.8998	**1.19**	1.00	**1.18**	1.19	**1.18**	1.00
	Ceramide (d18:1/20:0, d16:1/22:0, d20:1/18:0) *	0.4868	0.0077	0.9968	**0.78**	1.03	**0.76**	0.80	**0.78**	1.03
	Ceramide (d16:1/24:1, d18:1/22:1) *	0.5997	0.0021	0.4056	**0.74**	0.98	**0.76**	**0.65**	**0.67**	0.88
	Ceramide (d18:2/24:1, d18:1/24:2) *	0.0555	0.0299	0.9471	0.87	0.89	0.98	**0.77**	0.87	0.89
Hexosylceramides (HCERs)	Glycosyl-N-palmitoyl-sphingosine (d18:1/16:0)	0.4394	0.0150	0.8246	0.90	1.05	**0.86**	0.92	**0.88**	1.02
	Glycosyl-N-stearoyl-sphingosine (d18:1/18:0)	0.9589	0.6376	0.7008	1.01	1.03	0.99	0.98	0.95	0.97
Lactosylceramides (LCERs)	Lactosyl-N-palmitoyl-sphingosine (d18:1/16:0)	0.4252	0.3267	0.2246	0.99	0.99	1.00	1.12	1.13	1.13
Glycosphingolipid sulfates	3-sulfo-palmitoyl-galactosylceramide (d18:1/16:0)	0.4461	0.0210	0.9192	1.27	1.05	1.21	**1.40**	**1.33**	1.10
Dihydrosphingomyelins	Myristoyl dihydrosphingomyelin (d18:0/14:0) *	0.2235	0.0593	0.4681	1.05	1.03	1.03	**1.14**	**1.11**	1.08
	Palmitoyl dihydrosphingomyelin (d18:0/16:0) *	0.2268	0.0004	0.6068	**1.13**	1.05	**1.08**	**1.15**	**1.10**	1.02
	Behenoyl dihydrosphingomyelin (d18:0/22:0) *	0.0242	0.6006	0.4730	0.99	1.11	0.89	**1.19**	1.07	**1.20**
	Sphingomyelin (d18:0/18:0, d19:0/17:0) *	0.0123	0.0013	0.9767	**0.82**	**1.16**	**0.71**	0.94	**0.81**	**1.15**
	Sphingomyelin (d18:0/20:0, d16:0/22:0) *	0.0368	0.2445	0.2775	0.87	1.06	**0.82**	1.04	0.98	**1.20**
Sphingomyelins	Palmitoyl sphingomyelin (d18:1/16:0)	0.7313	0.2839	0.9938	0.98	1.01	0.97	0.98	0.98	1.01
	Stearoyl sphingomyelin (d18:1/18:0)	0.1434	0.7954	0.7945	1.00	1.06	0.94	1.04	0.98	1.04
	Behenoyl sphingomyelin (d18:1/22:0) *	0.3287	0.0197	0.8766	**0.89**	1.03	**0.86**	0.93	0.90	1.05
	Tricosanoyl sphingomyelin (d18:1/23:0) *	0.9744	0.0039	0.3964	1.11	0.97	**1.15**	**1.15**	**1.19**	1.03
	Lignoceroyl sphingomyelin (d18:1/24:0)	0.9045	0.8657	0.7088	1.00	0.98	1.02	1.01	1.03	1.01
	Sphingomyelin (d18:2/18:1) *	0.3845	0.0000	0.1672	**1.43**	1.12	**1.28**	**1.40**	**1.25**	0.98
	Sphingomyelin (d18:2/23:1) *	0.3656	0.0004	0.4288	**1.17**	0.91	**1.29**	**1.18**	**1.30**	1.01
	Sphingomyelin (d18:2/24:2) *	0.4569	0.0382	0.8459	1.33	0.87	**1.53**	1.16	1.34	0.88
	Sphingomyelin (d18:1/14:0, d16:1/16:0) *	0.5340	0.0036	0.5367	**0.91**	1.00	**0.91**	**0.94**	**0.94**	1.03
	Sphingomyelin (d18:2/14:0, d18:1/14:1) *	0.1158	0.1365	0.5531	0.91	0.91	1.00	**0.88**	0.96	0.96
	Sphingomyelin (d17:1/16:0, d18:1/15:0, d16:1/17:0) *	0.8171	0.2555	0.1996	1.00	0.96	1.04	1.03	**1.08**	1.03
	Sphingomyelin (d17:2/16:0, d18:2/15:0) *	0.0227	0.3836	0.3667	0.86	**0.77**	1.12	**0.79**	1.02	0.91
	Sphingomyelin (d18:2/16:0, d18:1/16:1) *	0.1738	0.0001	0.9930	**0.89**	0.96	**0.93**	**0.86**	**0.89**	0.96
	Sphingomyelin (d18:1/17:0, d17:1/18:0, d19:1/16:0)	0.6375	0.0000	0.6099	**1.33**	0.99	**1.33**	**1.27**	**1.28**	0.96
	Sphingomyelin (d18:1/18:1, d18:2/18:0)	0.9388	0.1891	0.8325	0.96	1.00	0.95	0.95	0.94	0.99
	Sphingomyelin (d18:1/19:0, d19:1/18:0) *	0.3110	0.0060	0.6235	**1.15**	1.03	1.12	**1.24**	**1.21**	1.08
	Sphingomyelin (d18:1/20:0, d16:1/22:0) *	0.1333	0.0005	0.4335	**0.83**	1.03	**0.80**	0.90	**0.88**	1.09
	Sphingomyelin (d18:1/20:1, d18:2/20:0) *	0.7756	0.0069	0.0570	**0.77**	0.90	**0.85**	**0.84**	0.94	1.09
	Sphingomyelin (d18:1/21:0, d17:1/22:0, d16:1/23:0) *	0.4026	0.2434	0.2278	1.01	0.98	1.02	1.10	1.12	1.10
	Sphingomyelin (d18:2/21:0, d16:2/23:0) *	0.8145	0.2049	0.2621	1.02	0.94	1.08	1.07	**1.14**	1.05
	Sphingomyelin (d18:1/22:1, d18:2/22:0, d16:1/24:1) *	0.6838	0.0029	0.2335	**0.88**	0.98	**0.90**	**0.92**	0.94	1.05
	Sphingomyelin (d18:1/22:2, d18:2/22:1, d16:1/24:2) *	0.5037	0.3716	0.1940	0.88	0.88	1.00	0.91	1.03	1.03
	Sphingomyelin (d18:2/23:0, d18:1/23:1, d17:1/24:1) *	0.8352	0.0545	0.3802	**0.89**	0.96	0.93	0.92	0.96	1.03
	Sphingomyelin (d18:1/24:1, d18:2/24:0) *	0.8445	0.0002	0.9895	**0.79**	1.00	**0.79**	**0.80**	**0.80**	1.01
	Sphingomyelin (d18:2/24:1, d18:1/24:2) *	0.7375	0.1452	0.3010	**0.91**	0.95	0.95	0.94	0.98	1.03
Sphingosines	Sphingosine	0.7159	0.2088	0.1583	1.01	1.11	0.90	0.96	**0.86**	0.95
	Hexadecasphingosine (d16:1) *	0.3551	0.3805	0.2911	1.17	1.16	1.01	1.19	1.02	1.02
	Heptadecasphingosine (d17:1)	0.4797	0.6932	0.4095	1.11	1.17	0.95	1.16	0.99	1.04
Mevalonate metabolism	3-hydroxy-3-methylglutarate	0.0656	0.4551	0.6526	0.94	**0.88**	1.07	**0.85**	0.97	0.90
Sterols	Cholesterol	0.7170	0.2418	0.8114	0.98	1.01	0.96	0.98	0.97	1.00
	Cholesterol sulfate	0.6763	0.1057	0.9951	1.12	1.03	1.09	1.18	1.14	1.05
	4-cholesten-3-one	0.7190	0.1869	0.6391	1.05	0.96	1.10	1.06	1.10	1.00
	7-hydroxycholesterol (alpha or beta)	0.7872	0.6582	0.6135	1.11	1.13	0.98	0.87	0.77	0.79
Pregnenolone steroids	Pregnenolone sulfate	0.3872	0.8758	0.8545	0.99	1.27	0.78	1.12	0.89	1.13
	21-hydroxypregnenolone disulfate	0.0607	0.9632	0.5523	0.96	1.10	0.87	1.23	1.11	**1.28**
	Pregnenetriol sulfate *	0.2229	0.1789	0.7972	1.14	1.15	1.00	**1.31**	1.14	1.15
Progestin steroids	Pregnanolone/allopregnanolone sulfate	0.2288	0.7287	0.7837	1.02	1.21	0.85	1.20	0.99	1.17
Corticosteroids	Cortisol	0.2617	0.3675	0.7489	0.88	1.34	0.66	1.07	0.80	1.21
Androgenic steroids	Dehydroepiandrosterone sulfate (DHEA-S)	0.8401	0.4098	0.3512	1.00	1.33	0.75	0.86	0.64	0.85
	Androsterone sulfate	0.2469	0.9787	0.5246	0.99	1.19	0.83	1.10	0.92	1.10
Primary bile acid metabolism	Cholate	0.2155	0.5108	0.1908	0.86	**0.65**	1.32	0.90	1.37	1.04
	Glycocholate	0.8154	0.3815	0.9794	1.29	1.02	1.26	1.43	1.41	1.12
	Taurocholate	0.6529	0.1564	0.5674	1.24	0.91	1.37	1.52	1.68	1.23
	Chenodeoxycholate	0.5539	0.2660	0.1563	0.96	0.93	1.04	1.21	**1.30**	1.25
	Glycochenodeoxycholate	0.0659	0.3253	0.8968	1.23	1.31	0.94	**1.60**	1.23	1.30
	Taurochenodeoxycholate	0.0413	0.0827	0.7370	1.23	1.25	0.98	**1.70**	1.36	**1.39**
Secondary bile acid metabolism	Deoxycholate	0.0033	0.2298	0.0755	**0.61**	**0.43**	1.43	**0.48**	1.12	0.78
	Glycodeoxycholate	0.0326	0.0344	0.7046	**0.58**	**0.51**	1.15	**0.46**	0.90	0.79
	Taurodeoxycholate	0.0171	0.3697	0.3399	0.66	**0.50**	1.31	**0.56**	1.11	0.85
	Taurolithocholate	0.0126	0.0440	0.8081	1.19	**1.28**	0.93	**1.59**	1.24	**1.33**
	Glycoursodeoxycholate	0.0799	0.0736	0.0434	**0.40**	**0.34**	1.15	**0.43**	1.24	1.08
	Tauroursodeoxycholate	0.2232	0.3622	0.0366	**0.38**	**0.32**	1.17	0.46	1.42	1.22
	Glycocholenate sulfate *	0.2888	0.5286	0.5698	1.38	1.27	1.08	1.32	1.04	0.96
	Taurocholenate sulfate *	0.0428	0.0351	0.5235	**1.81**	**1.47**	1.24	**2.09**	1.43	1.15
	7-ketodeoxycholate	0.2152	0.2591	0.3728	0.59	0.56	1.05	0.55	0.98	0.93

For the ANOVA, blue-shaded cells indicate *p* ≤ 0.05; light-blue-shaded cells indicate 0.05 < *p* < 0.10. Red- and green-shaded cells indicate *p* ≤ 0.05 (red indicates that the mean values are significantly greater for the comparison in question; green indicates significantly lower values). Light-red- and light-green-shaded cells indicate 0.05 < *p* < 0.10 (light red indicates that the mean values trend higher for the comparison in question; light green indicates that values trend lower). Biochemicals/metabolites denoted with * are compounds that have not been confirmed based on a standard but whose identity we are confident about—extracts are of sufficient purity to enable the necessary resolution for accurate identification. Biochemicals/metabolites denoted with ** (3-hydroxybutyroylglycine and 1-stearoyl-2-arachidonoyl-glycosyl-GPE (18:0/20:4)) are compounds for which standards are not available but whose identities were matched with publicly available databases.

## Data Availability

The data presented in this study are available in the main article and in the Supplementary Materials.

## Supplementary Materials

The following supporting information can be downloaded at: https://www.mdpi.com/article/10.3390/metabo13020175/s1, Table S1: The complete data including *p*-values, FDR protected q-values, fold changes, mean values, and percent filled values for all results in this manuscript. Figure S1: Supplementary Figures Box Plots.

## References

[1] Khaire A.A., Kale A.A., Joshi S.R. (2015). Maternal Omega-3 Fatty Acids and Micronutrients Modulate Fetal Lipid Metabolism: A Review. Prostaglandins Leukot. Essent..

[2] Miranda J., Simões R.V., Paules C., Cañueto D., Pardo-Cea M.A., García-Martín M.L., Crovetto F., Fuertes-Martin R., Domenech M., Gómez-Roig M.D. (2018). Metabolic Profiling and Targeted Lipidomics Reveals a Disturbed Lipid Profile in Mothers and Fetuses with Intrauterine Growth Restriction. Sci. Rep..

[3] Rosa Velazquez M., Batistel F., Pinos Rodriguez J.M., Relling A.E. (2020). Effects of Maternal Dietary Omega-3 Polyunsaturated Fatty Acids and Methionine during Late Gestation on Fetal Growth, DNA Methylation, and MRNA Relative Expression of Genes Associated with the Inflammatory Response, Lipid Metabolism and DNA Methylation in Placenta and Offspring’s Liver in Sheep. J. Anim. Sci. Biotechnol..

[4] Sousa B.C., Wakelam M.J.O., Lopez-Clavijo A.F., Ridgway N.D., McLeod R.S. (2021). Chapter 2—Methods of Lipid Analysis. Biochemistry of Lipids, Lipoproteins and Membranes.

[5] Roque-Jiménez J.A., Rosa-Velázquez M., Pinos-Rodríguez J.M., Vicente-Martínez J.G., Mendoza-Cervantes G., Flores-Primo A., Lee-Rangel H.A., Relling A.E. (2021). Role of Long Chain Fatty Acids in Developmental Programming in Ruminants. Animals.

[6] Godfrey K.M., Barker D.J.P. (2000). Fetal nutrition and adult disease. Am. J. Clin. Nutr..

[7] Menezes A.C.B., McCarthy K.L., Kassetas C.J., Baumgaertner F., Kirsch J.D., Dorsam S.T., Neville T.L., Ward A.K., Borowicz P.P., Reynolds L.P. (2022). Vitamin and Mineral Supplementation and Rate of Gain in Beef Heifers I: Effects on Dam Hormonal and Metabolic Status, Fetal Tissue and Organ Mass, and Concentration of Glucose and Fructose in Fetal Fluids at d 83 of Gestation. Animals.

[8] Crouse M.S., McCarthy K.L., Menezes A.C.B., Kassetas C.J., Baumgaertner F., Kirsch J.D., Dorsam S., Neville T.L., Ward A.K., Borowicz P.P. (2022). Vitamin and Mineral Supplementation and Rate of Weight Gain during the First Trimester of Gestation in Beef Heifers Alters the Fetal Liver Amino Acid, Carbohydrate, and Energy Profile at Day 83 of Gestation. Metabolites.

[9] Diniz W.J.S., Reynolds L.P., Borowicz P.P., Ward A.K., Sedivec K.K., McCarthy K.L., Kassetas C.J., Baumgaertner F., Kirsch J.D., Dorsam S.T. (2021). Maternal Vitamin and Mineral Supplementation and Rate of Maternal Weight Gain Affects Placental Expression of Energy Metabolism and Transport-Related Genes. Genes.

[10] Diniz W.J.S., Ward A.K.K., Reynolds L.P.P., Borowicz P.P.P., Sedivec K.K.K., McCarthy K.L.L., Kassetas C.J.J., Baumgaertner F., Kirsch J.D., Dorsam S.T. (2021). PSVIII-22 Maternal Vitamin and Mineral Supplementation Affect Fetal Hepatic Expression of Genes Underlying Mineral Homeostasis and Lipid Metabolism in Early Pregnancy. J. Anim. Sci..

[11] McCarthy K.L., Menezes A.C.B., Kassetas C.J., Baumgaertner F., Kirsch J.D., Dorsam S.T., Neville T.L., Ward A.K., Borowicz P.P., Reynolds L.P. (2022). Vitamin and Mineral Supplementation and Rate of Gain in Beef Heifers II: Effects on tConcentration of Trace Minerals in Maternal Liver and Fetal Liver, Muscle, Allantoic, and Amniotic Fluid at d 83 of gestation. Animals.

[12] National Academy of Sciences Engineering and Medicine (2016). Nutrient Requirements of Beef Cattle: Eighth Revised Edition.

[13] McLean K.J., Dahlen C.R., Borowicz P.P., Reynolds L.P., Crosswhite M.R., Neville B.W., Walden S.D., Caton J.S. (2016). Technical note: A new surgical technique for ovariohysterectomy during early pregnancy in beef heifers. J. Anim. Sci..

[14] Simintiras C.A., Sánchez J.M., McDonald M., Martins T., Binelli M., Lonergan P. (2019). Biochemical Characterization of Progesterone-Induced Alterations in Bovine Uterine Fluid Amino Acid and Carbohydrate Composition during the Conceptus Elongation Window. Biol. Reprod..

[15] Childs S., Hennessy A.A., Sreenan J.M., Wathes D.C., Cheng Z., Stanton C., Diskin M.G., Kenny D.A. (2008). Effect of Level of Dietary N-3 Polyunsaturated Fatty Acid Supplementation on Systemic and Tissue Fatty Acid Concentrations and on Selected Reproductive Variables in Cattle. Theriogenology.

[16] Campbell F.M., Gordon M.J., Dutta-Roy A.K. (1994). Plasma Membrane Fatty Acid-Binding Protein (FABP) of the Sheep Placenta. Biochim. Biophys. Acta.

[17] Brett K.E., Ferraro Z.M., Yockell-Lelievre J., Gruslin A., Adamo K.B. (2014). Maternal–Fetal Nutrient Transport in Pregnancy Pathologies: The Role of the Placenta. Int. J. Mol. Sci..

[18] Hidalgo M.A., Ratto M., Burgos R.A., Waisundara V.Y., Jovandaric M.Z. (2020). Beneficial effect of Omega-3 fatty acids on immune and reproductive endometrial function. Apolipoproteins, Triglycerides, and Cholesterol.

[19] Zhao X., Qiu X. (2019). Very Long Chain Polyunsaturated Fatty Acids Accumulated in Triacylglycerol Are Channeled from Phosphatidylcholine in Thraustochytrium. Front. Microbiol..

[20] Abbott S.K., Else P.L., Atkins T.A., Hulbert A.J. (2012). Fatty Acid Composition of Membrane Bilayers: Importance of Diet Polyunsaturated Fat Balance. Biochim. Biophys. Acta Biomembr..

[21] Xia T., Coleman R.A. (1992). Diacylglycerol Metabolism in Neonatal Rat Liver: Characterization of Cytosolic Diacylglycerol Lipase Activity and Its Activation by Monoalkylglycerols. Biochim. Biophys. Acta.

[22] Coleman R.A., Dennis E.A., Ennis V. (1992). Diacylglycerol Acyltransferase and Monoacylglycerol Acyltransferase from Liver and Intestine. Phospholipid Biosynthesis.

[23] McCann M.R., de la Rosa M.V.G., Rosania G.R., Stringer K.A. (2021). L-Carnitine and Acylcarnitines: Mitochondrial Biomarkers for Precision Medicine. Metabolites.

[24] Menezes A.C.B., Baumgaertner F., Hurlbert J.L., Bochantin K.A., Kirsch J.D., Dorsam S., Sedivec K.K., Swanson K.C., Dahlen C.R. Feeding a Se-Containing Trace Mineral Supplement during Pregnancy: Effects on Mitochondrial Oxygen Consumption in Liver and Small Intestine of Neonatal Calves. Proceedings of the 12th International Symposium on Selenium in Biology and Medicine.

[25] Macias R.I.R., Marin J.J.G., Serrano M.A. (2009). Excretion of Biliary Compounds during Intrauterine Life. World J. Gastroenterol..

[26] Hardy K.J., Hoffman N.E., Mihaly G., Sewell R.B., Smallwood R.A. (1980). Bile acid metabolism in fetal sheep; Perinatal changes in the bile acid pool. J. Physiol..

[27] Amat S., Holman D.B., Schmidt K., McCarthy K.L., Dorsam S.T., Ward A.K., Borowicz P.P., Reynolds L.P., Caton J.S., Sedivec K.K. (2022). Characterization of the Microbiota Associated with 12-Week-Old Bovine Fetuses Exposed to Divergent in Utero Nutrition. Front. Microbiol..

