# Supplementary material for: Fetal Hepatic Lipidome Is More Greatly Affected by Maternal Rate of Gain Compared with Vitamin and Mineral Supplementation at day 83 of Gestation
Source: Metabolites. 2023 Jan 25;13(2):175. doi: 10.3390/metabo13020175 (PMC9961797; doi:10.3390/metabo13020175)
Supplement: Supplementary file 1 [file metabolites-13-00175-s001.zip › Supplementary Figure S1. Lipidomics BOX PLOTS.pptx]

## Slide 1
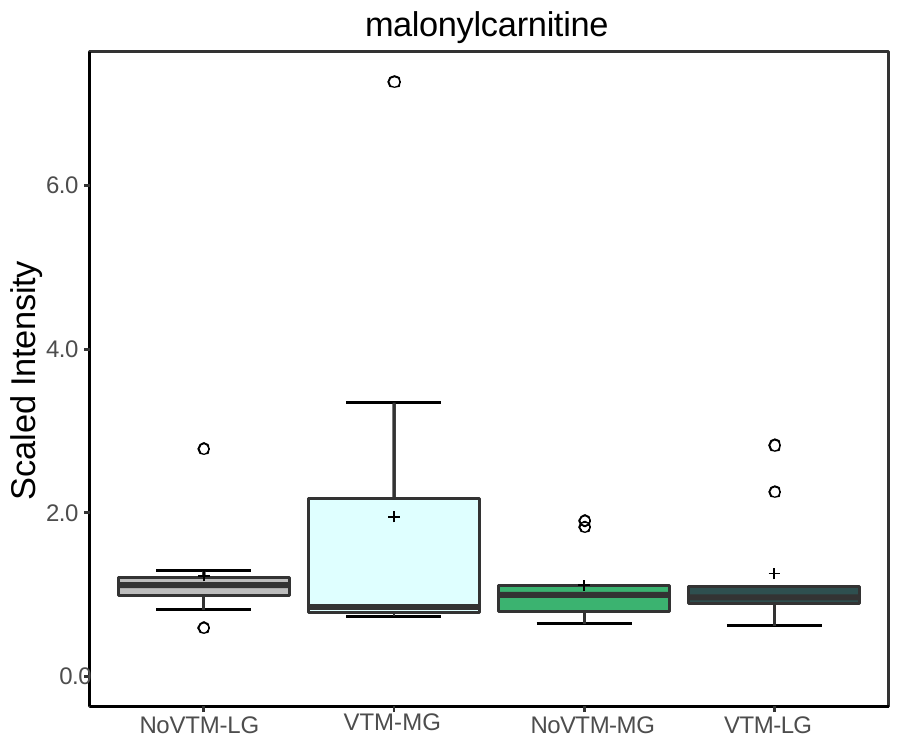

malonylcarnitine
6.0
Scaled Intensity
4.0
2.0
0.0
VTM-MG
NoVTM-LG
NoVTM-MG
VTM-LG

## Slide 2
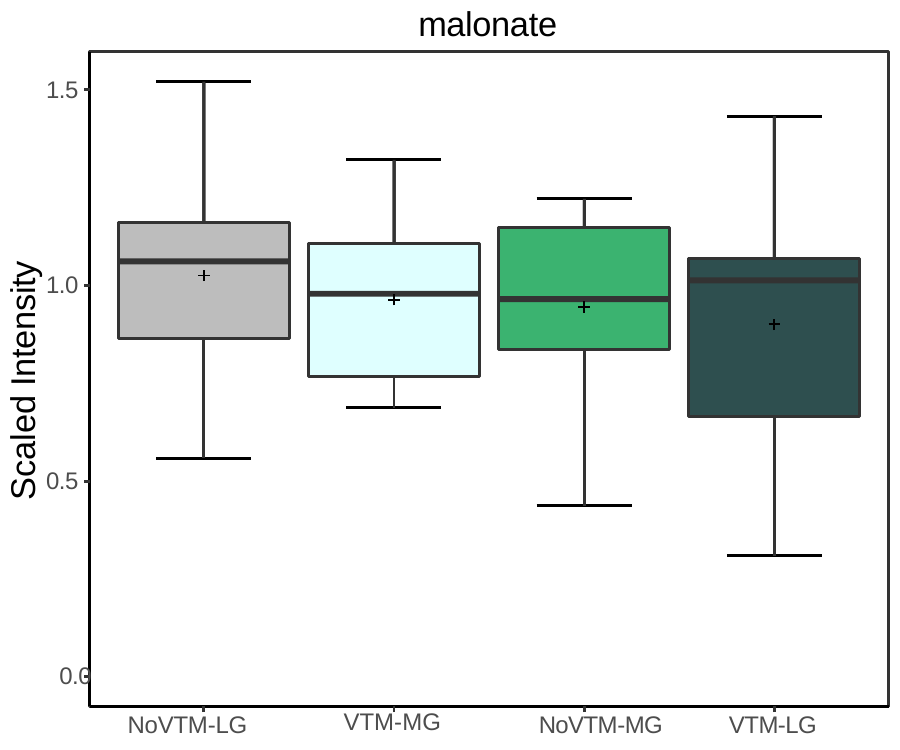

malonate
1.5
Scaled Intensity
1.0
0.5
0.0
VTM-MG
NoVTM-LG
NoVTM-MG
VTM-LG

## Slide 3
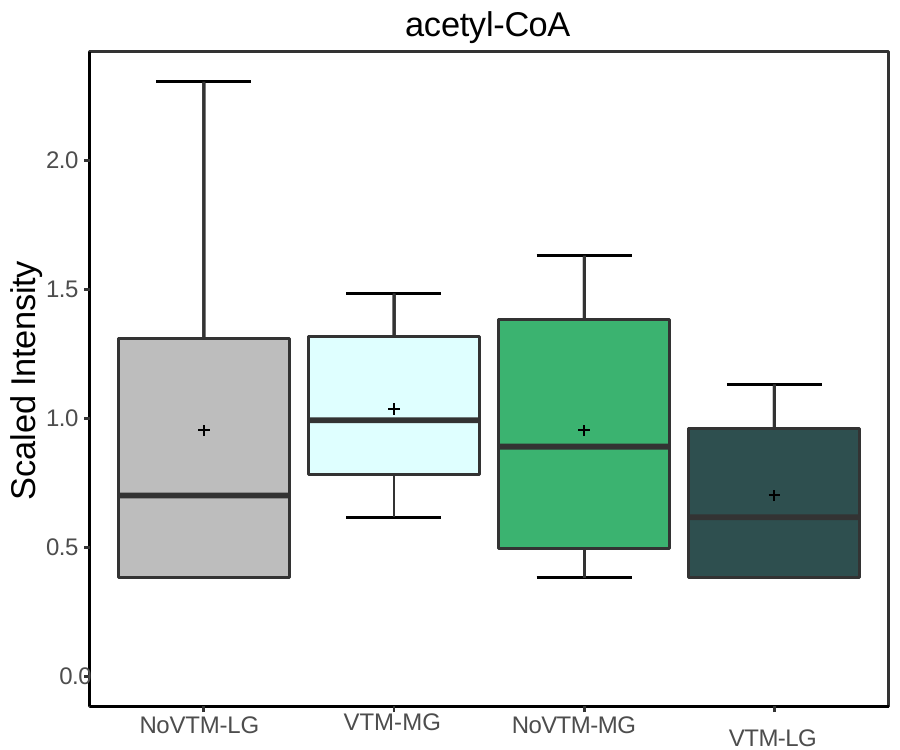

acetyl-CoA
2.0
Scaled Intensity
1.5
1.0
0.5
0.0
VTM-MG
NoVTM-LG
NoVTM-MG
VTM-LG

## Slide 4
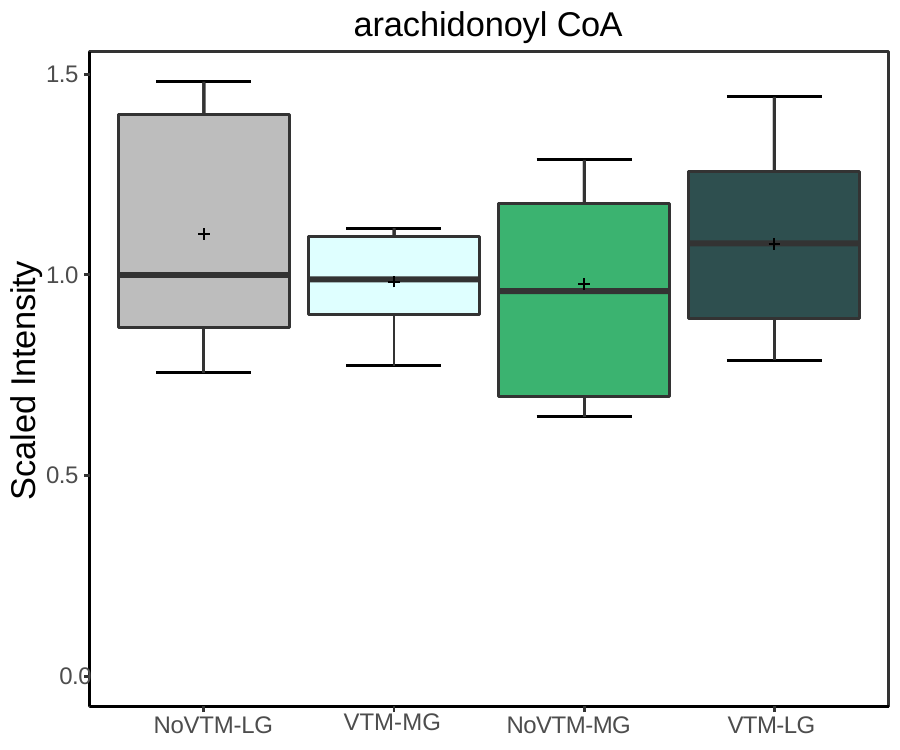

arachidonoyl CoA
1.5
Scaled Intensity
1.0
0.5
0.0
VTM-MG
NoVTM-LG
NoVTM-MG
VTM-LG

## Slide 5
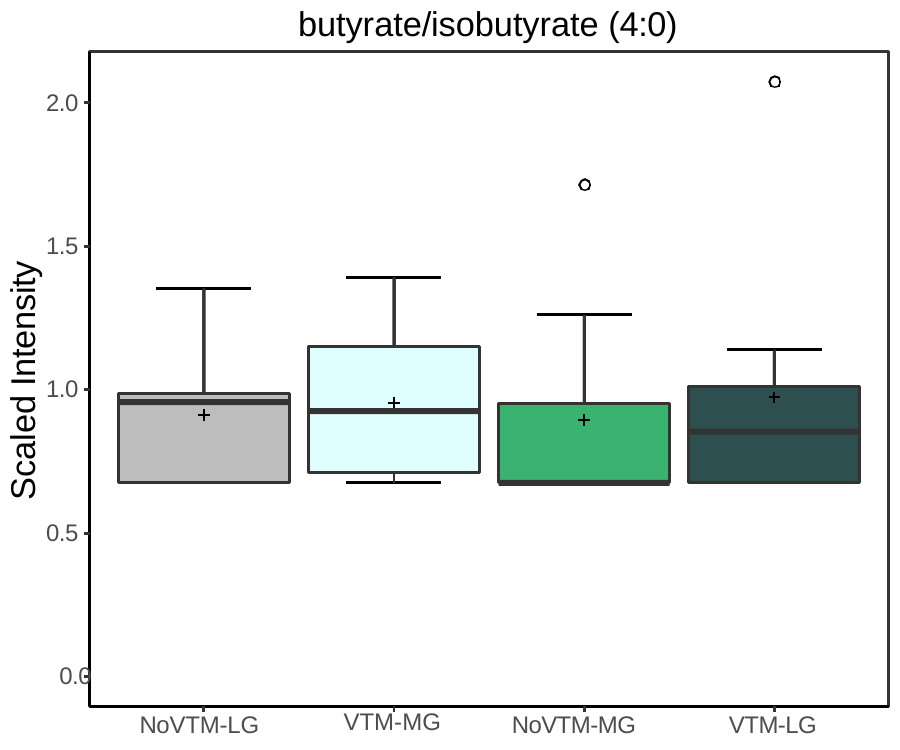

butyrate/isobutyrate (4:0)
2.0
1.5
Scaled Intensity
1.0
0.5
0.0
VTM-MG
NoVTM-LG
NoVTM-MG
VTM-LG

## Slide 6
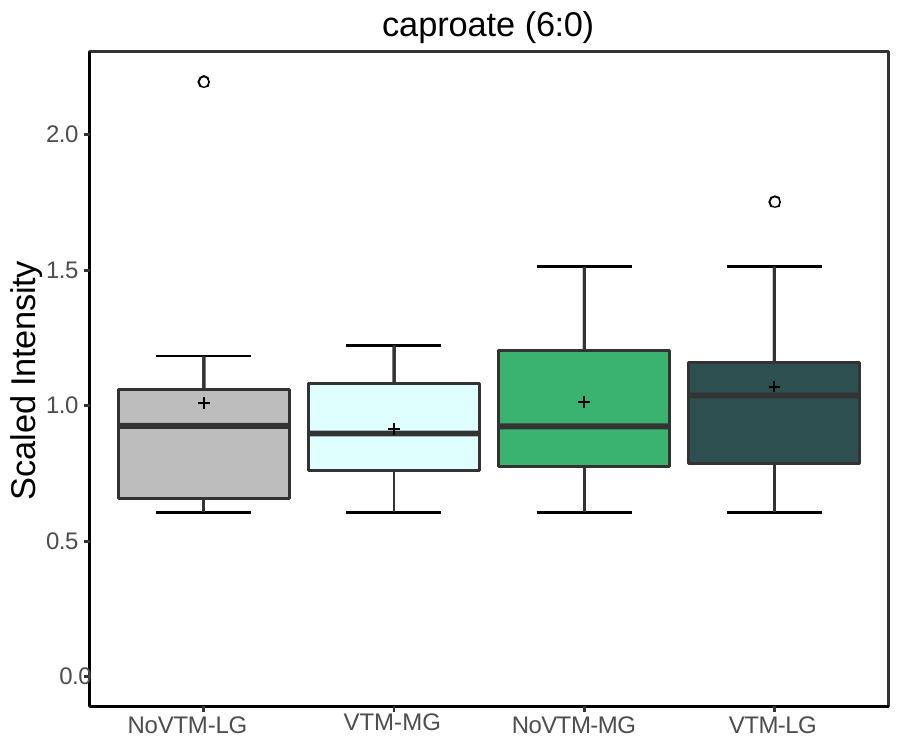

caproate (6:0)
2.0
1.5
Scaled Intensity
1.0
0.5
0.0
VTM-MG
NoVTM-LG
NoVTM-MG
VTM-LG

## Slide 7
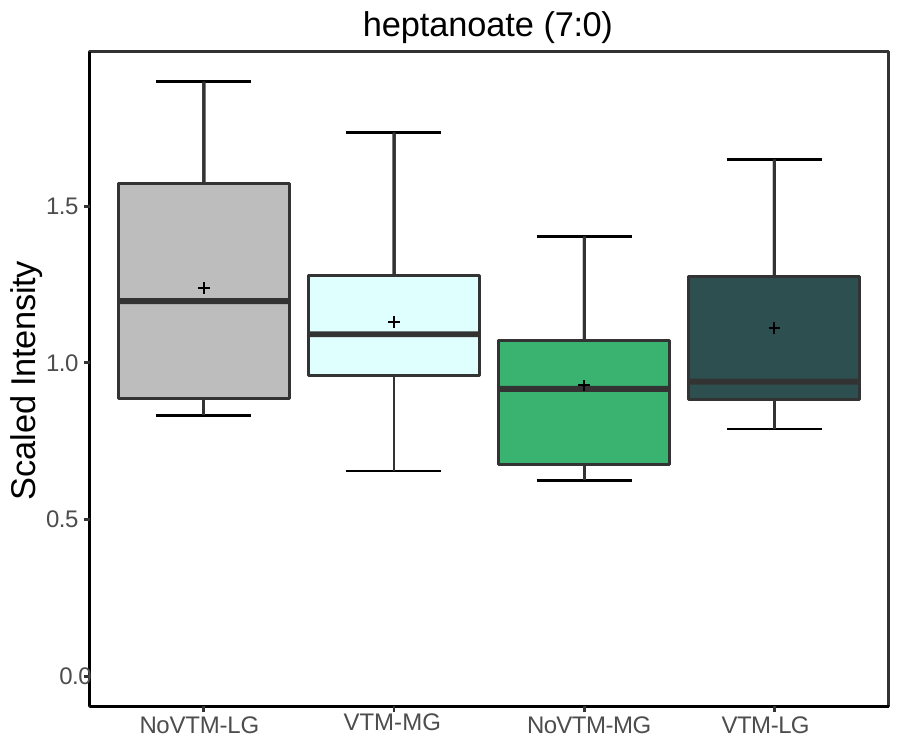

heptanoate (7:0)
1.5
Scaled Intensity
1.0
0.5
0.0
VTM-MG
NoVTM-LG
NoVTM-MG
VTM-LG

## Slide 8
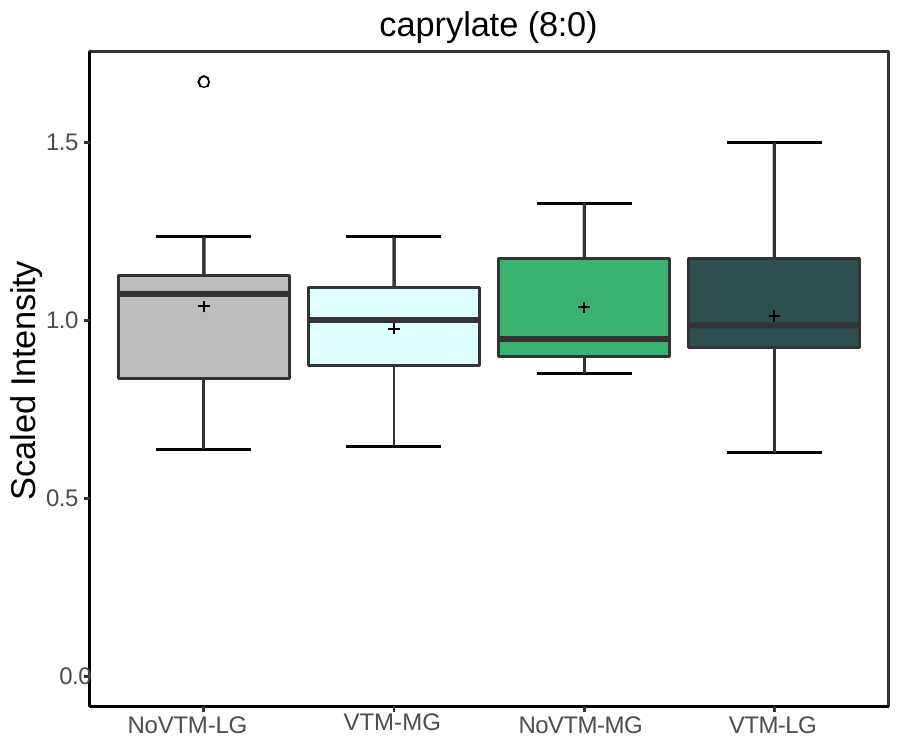

caprylate (8:0)
1.5
Scaled Intensity
1.0
0.5
0.0
VTM-MG
NoVTM-LG
NoVTM-MG
VTM-LG

## Slide 9
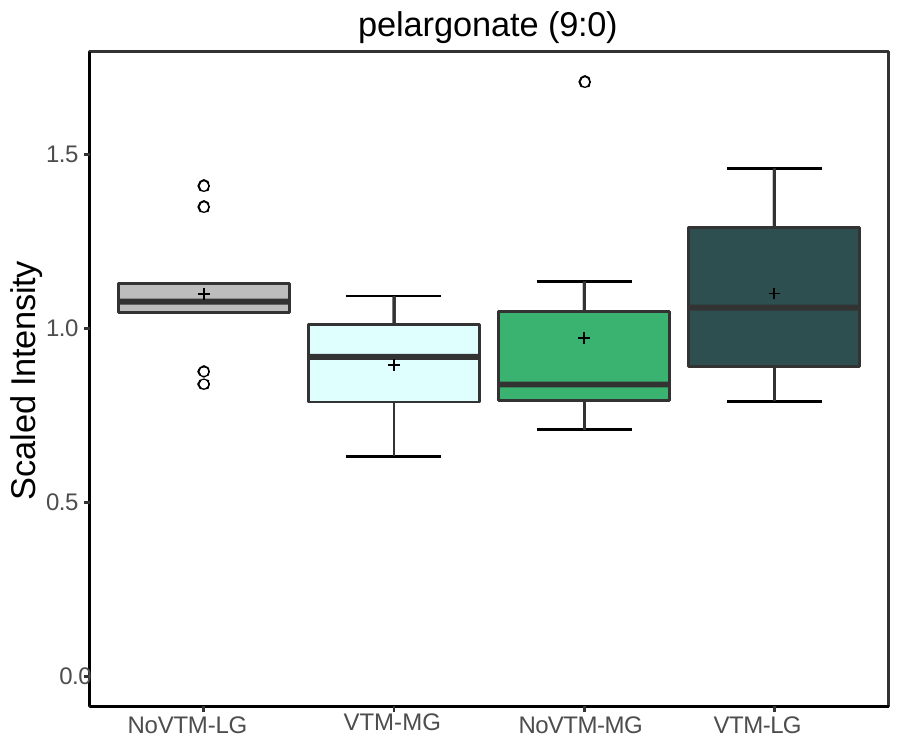

pelargonate (9:0)
1.5
Scaled Intensity
1.0
0.5
0.0
VTM-MG
NoVTM-LG
NoVTM-MG
VTM-LG

## Slide 10
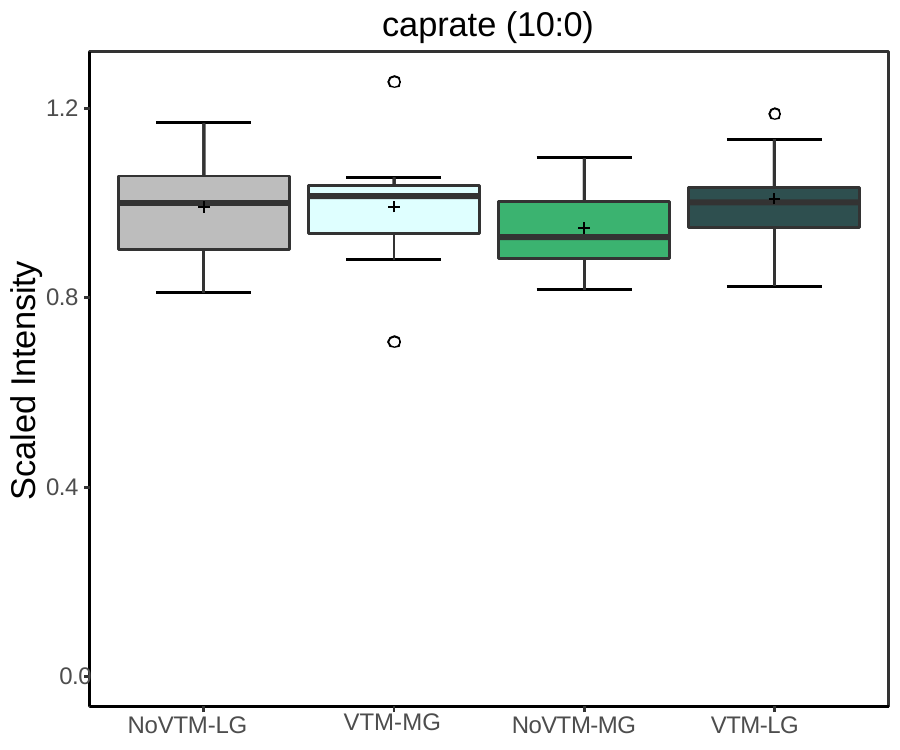

caprate (10:0)
1.2
Scaled Intensity
0.8
0.4
0.0
VTM-MG
NoVTM-LG
NoVTM-MG
VTM-LG

## Slide 11
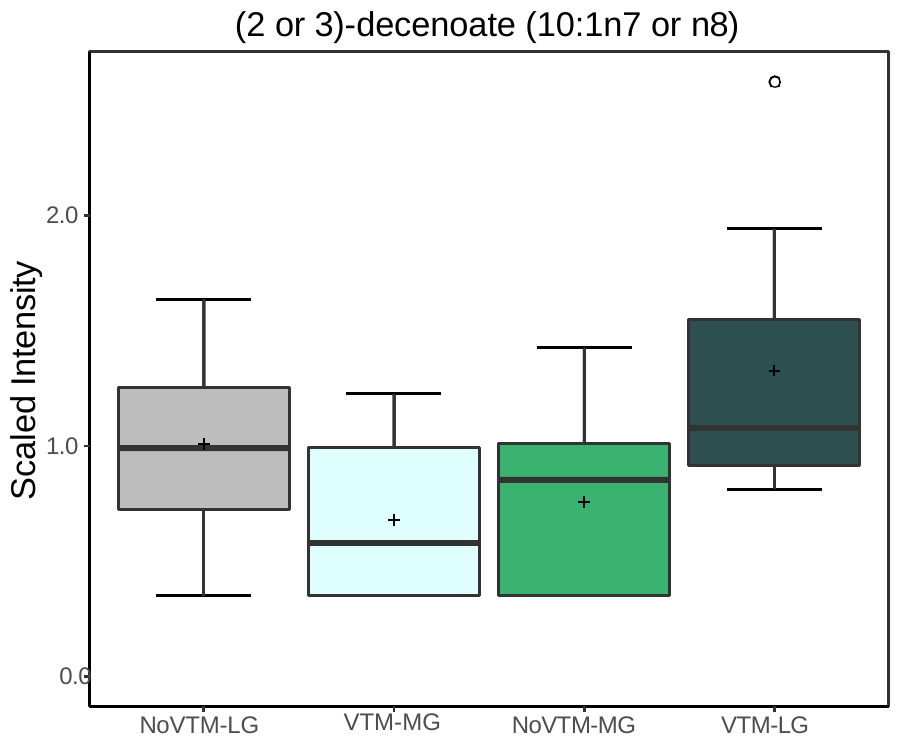

(2 or 3)-decenoate (10:1n7 or n8)
2.0
Scaled Intensity
1.0
0.0
VTM-MG
NoVTM-LG
NoVTM-MG
VTM-LG

## Slide 12
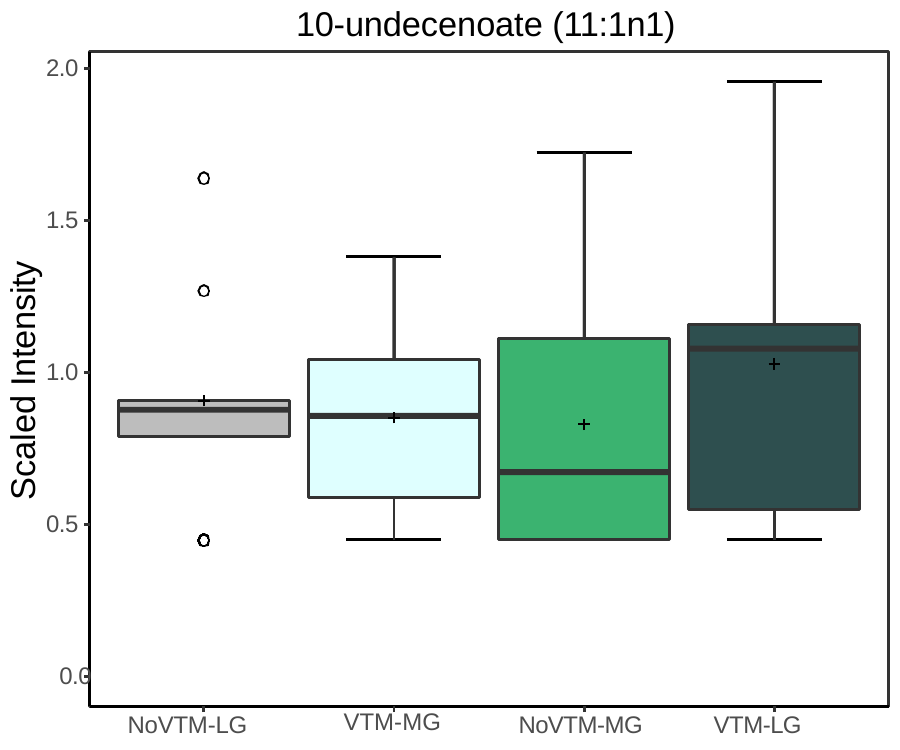

10-undecenoate (11:1n1)
2.0
1.5
Scaled Intensity
1.0
0.5
0.0
VTM-MG
NoVTM-LG
NoVTM-MG
VTM-LG

## Slide 13
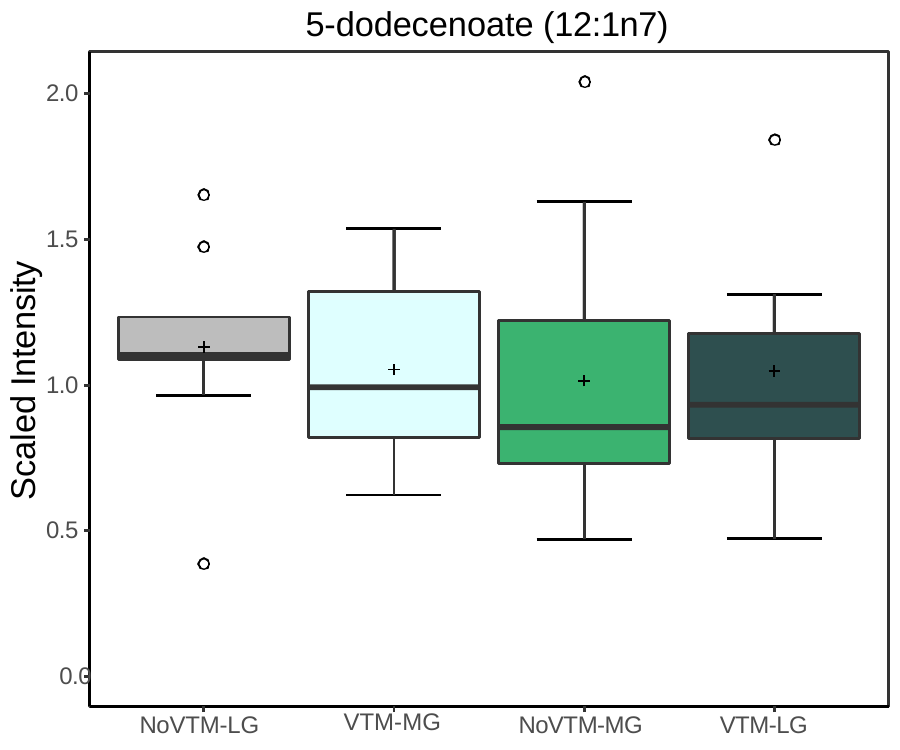

5-dodecenoate (12:1n7)
2.0
1.5
Scaled Intensity
1.0
0.5
0.0
VTM-MG
NoVTM-LG
NoVTM-MG
VTM-LG

## Slide 14
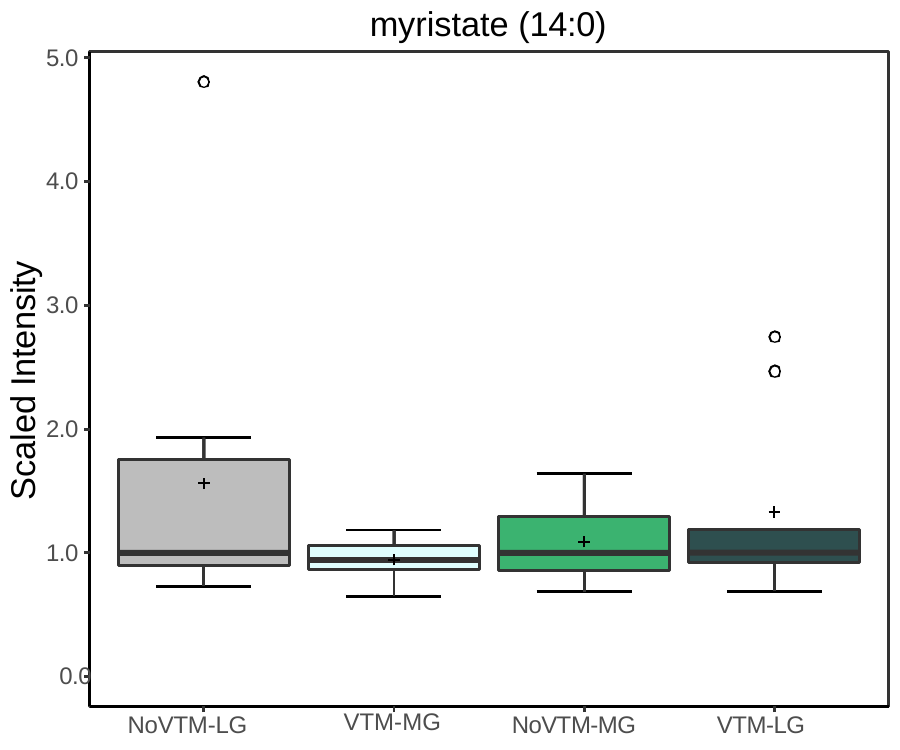

myristate (14:0)
5.0
4.0
Scaled Intensity
3.0
2.0
1.0
0.0
VTM-MG
NoVTM-LG
NoVTM-MG
VTM-LG

## Slide 15
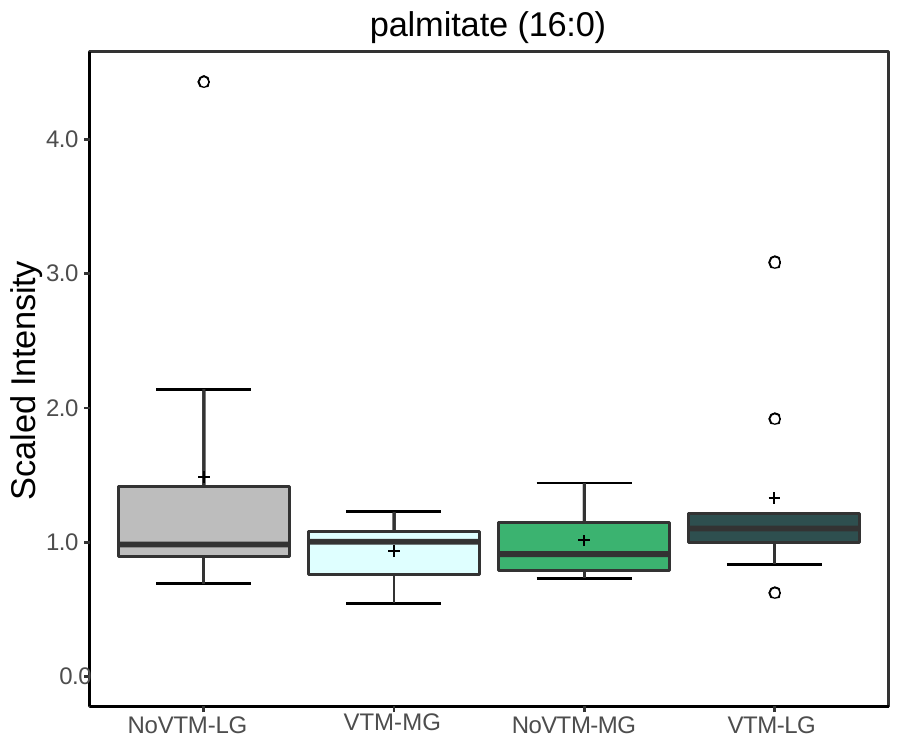

palmitate (16:0)
4.0
3.0
Scaled Intensity
2.0
1.0
0.0
VTM-MG
NoVTM-LG
NoVTM-MG
VTM-LG

## Slide 16
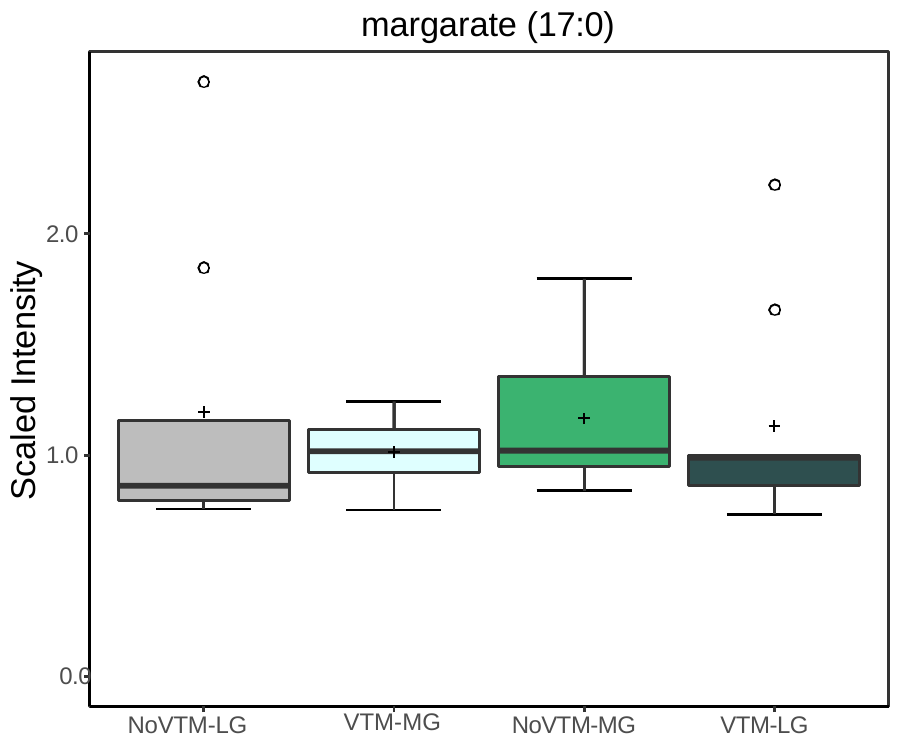

margarate (17:0)
2.0
Scaled Intensity
1.0
0.0
VTM-MG
NoVTM-LG
NoVTM-MG
VTM-LG

## Slide 17
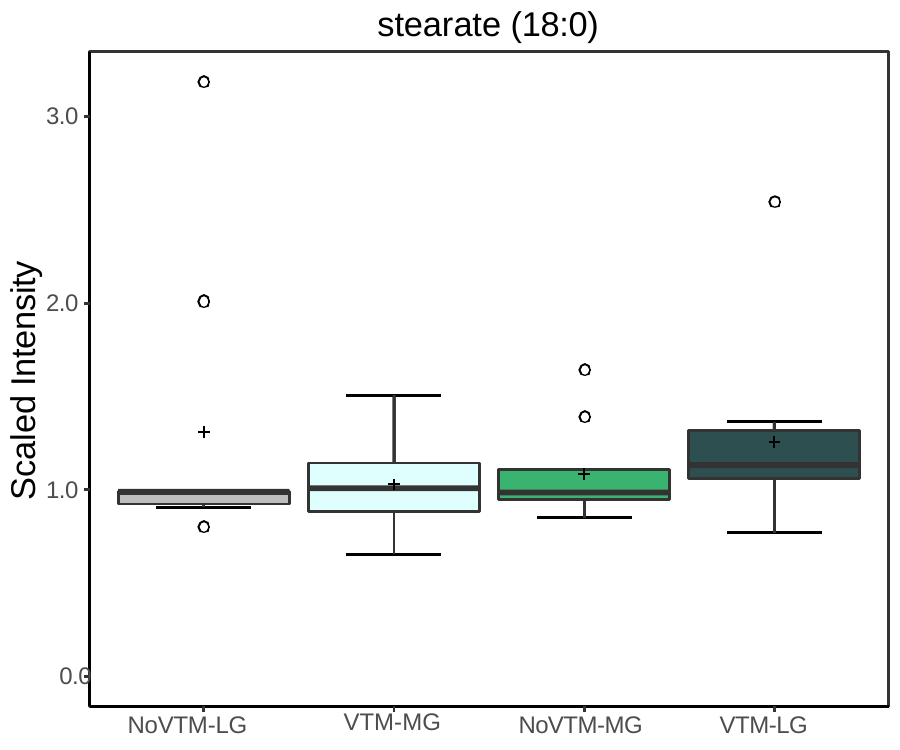

stearate (18:0)
3.0
Scaled Intensity
2.0
1.0
0.0
VTM-MG
NoVTM-LG
NoVTM-MG
VTM-LG

## Slide 18
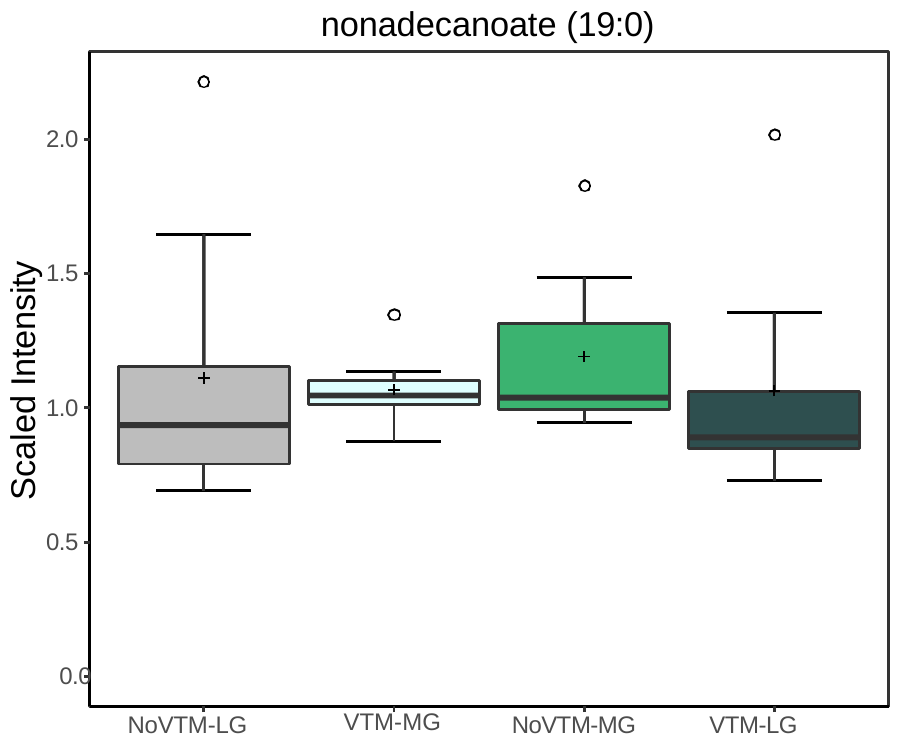

nonadecanoate (19:0)
2.0
1.5
Scaled Intensity
1.0
0.5
0.0
VTM-MG
NoVTM-LG
NoVTM-MG
VTM-LG

## Slide 19
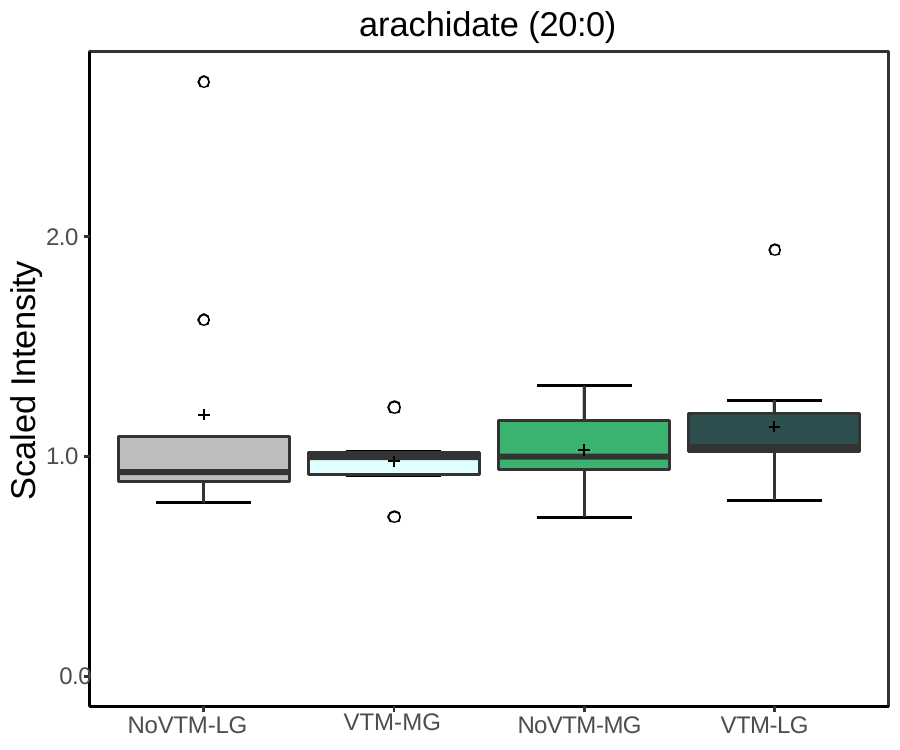

arachidate (20:0)
2.0
Scaled Intensity
1.0
0.0
VTM-MG
NoVTM-LG
NoVTM-MG
VTM-LG

## Slide 20
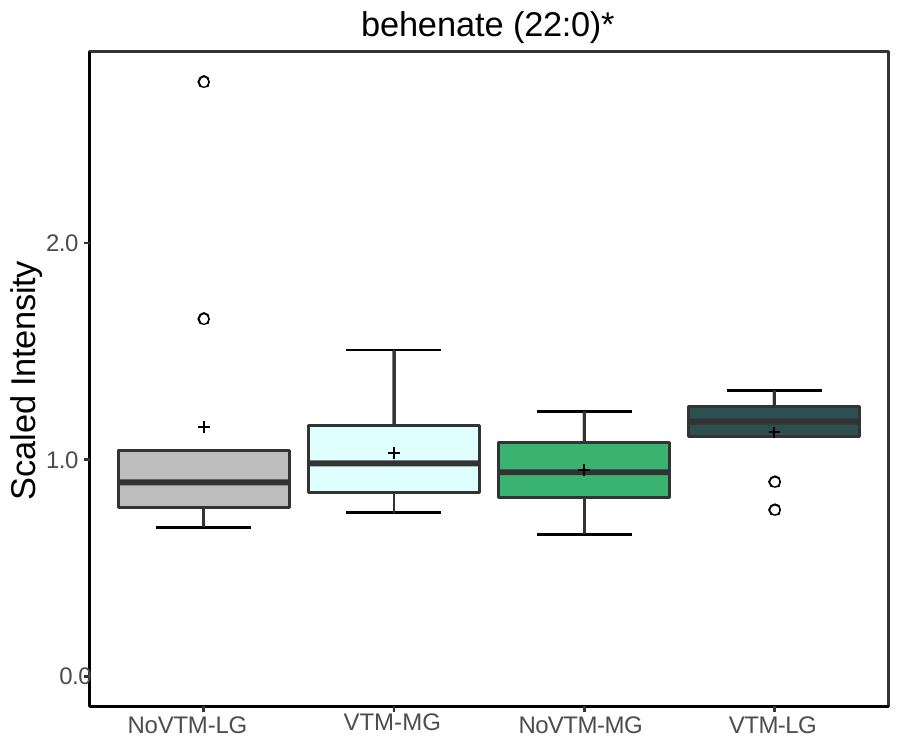

behenate (22:0)*
2.0
Scaled Intensity
1.0
0.0
VTM-MG
NoVTM-LG
NoVTM-MG
VTM-LG

## Slide 21
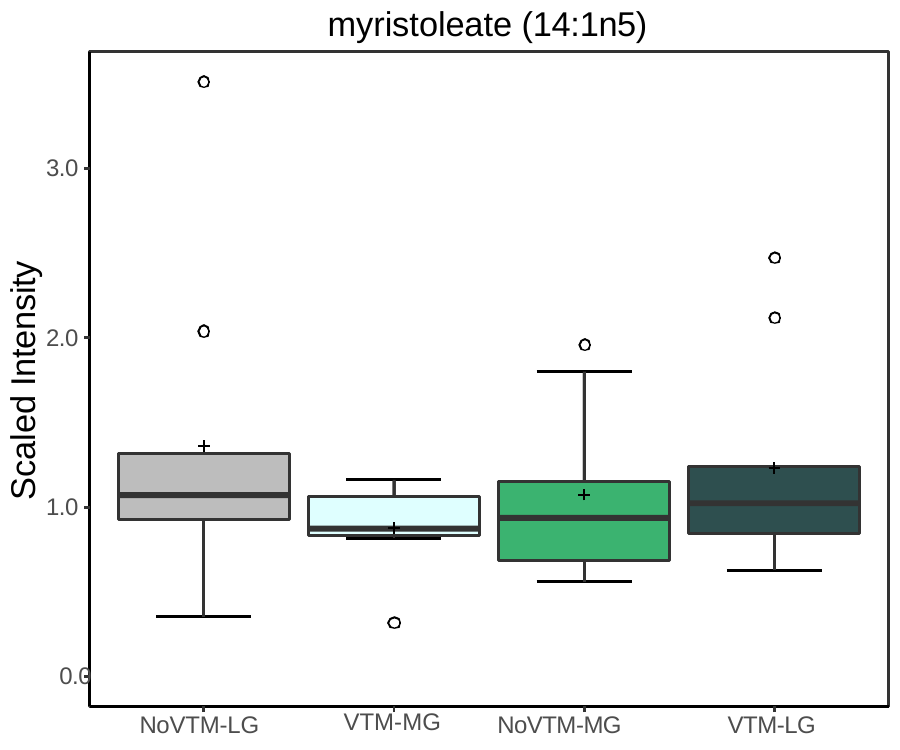

myristoleate (14:1n5)
3.0
Scaled Intensity
2.0
1.0
0.0
VTM-MG
NoVTM-LG
NoVTM-MG
VTM-LG

## Slide 22
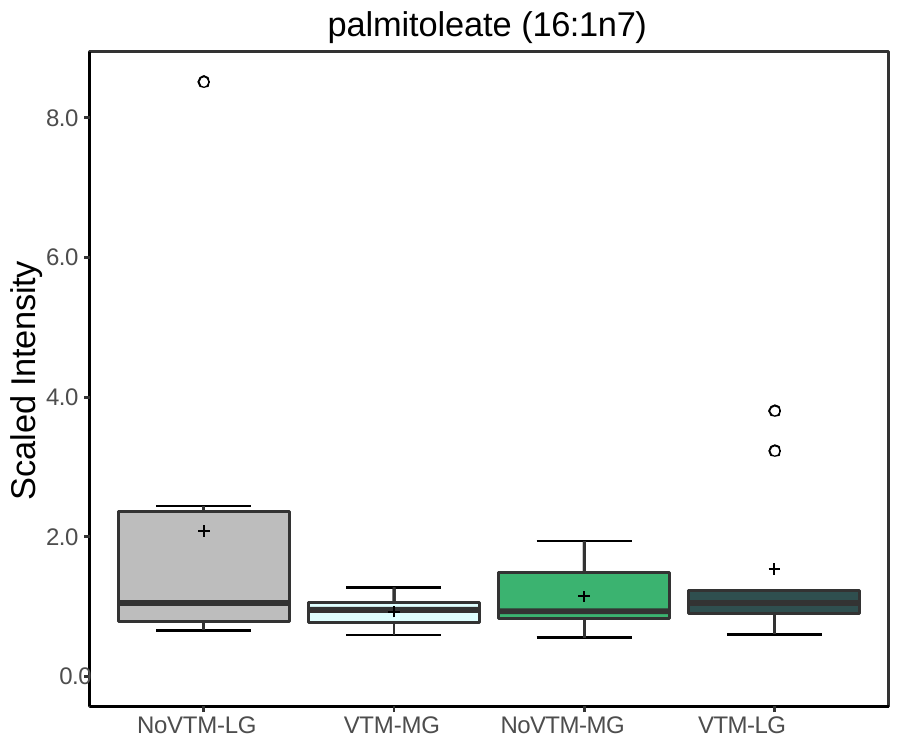

palmitoleate (16:1n7)
8.0
6.0
Scaled Intensity
4.0
2.0
0.0
NoVTM-LG
VTM-MG
NoVTM-MG
VTM-LG

## Slide 23
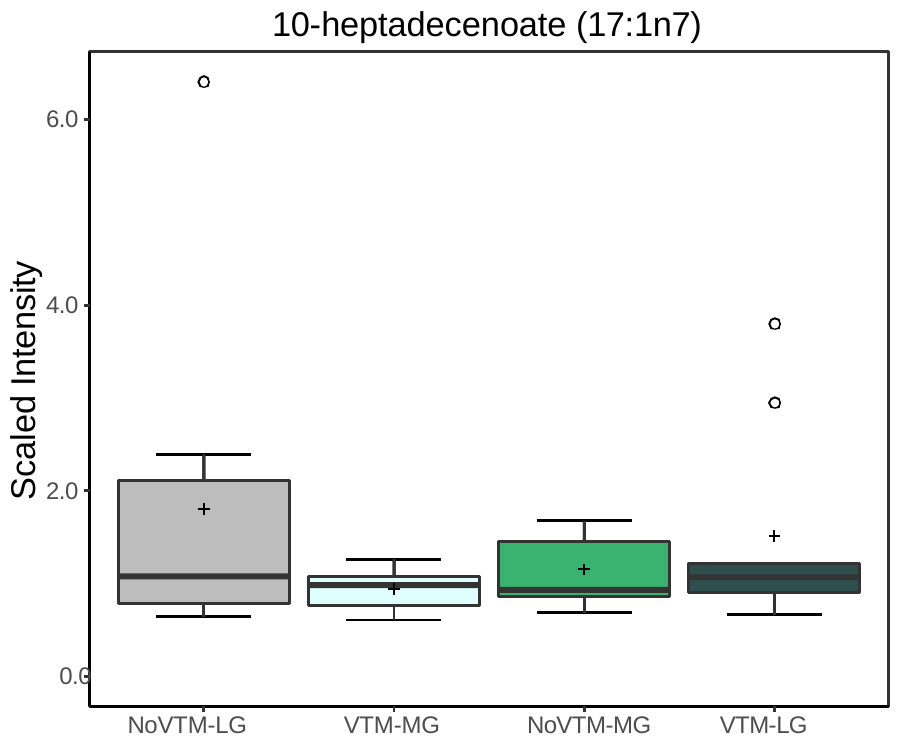

10-heptadecenoate (17:1n7)
6.0
Scaled Intensity
4.0
2.0
0.0
NoVTM-LG
VTM-MG
NoVTM-MG
VTM-LG

## Slide 24
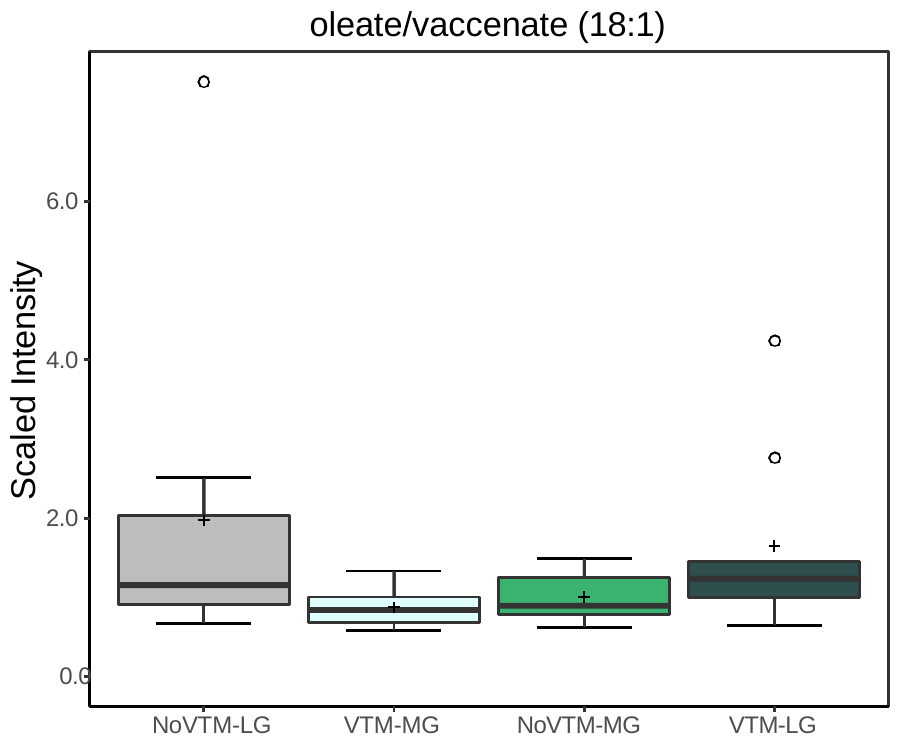

oleate/vaccenate (18:1)
6.0
Scaled Intensity
4.0
2.0
0.0
NoVTM-LG
VTM-MG
NoVTM-MG
VTM-LG

## Slide 25
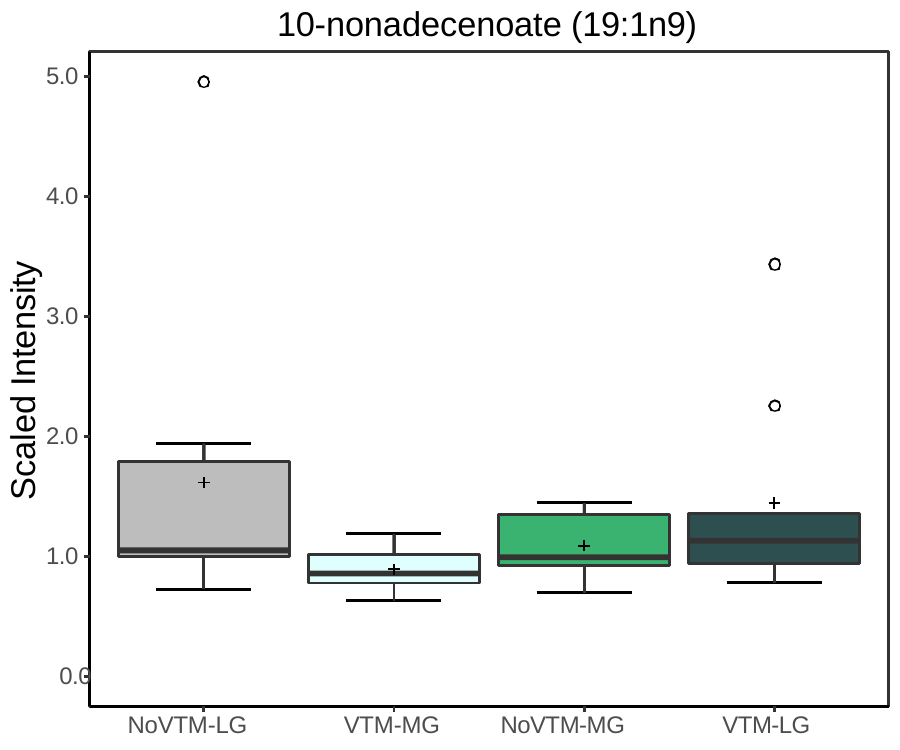

10-nonadecenoate (19:1n9)
5.0
4.0
Scaled Intensity
3.0
2.0
1.0
0.0
NoVTM-LG
VTM-MG
NoVTM-MG
VTM-LG

## Slide 26
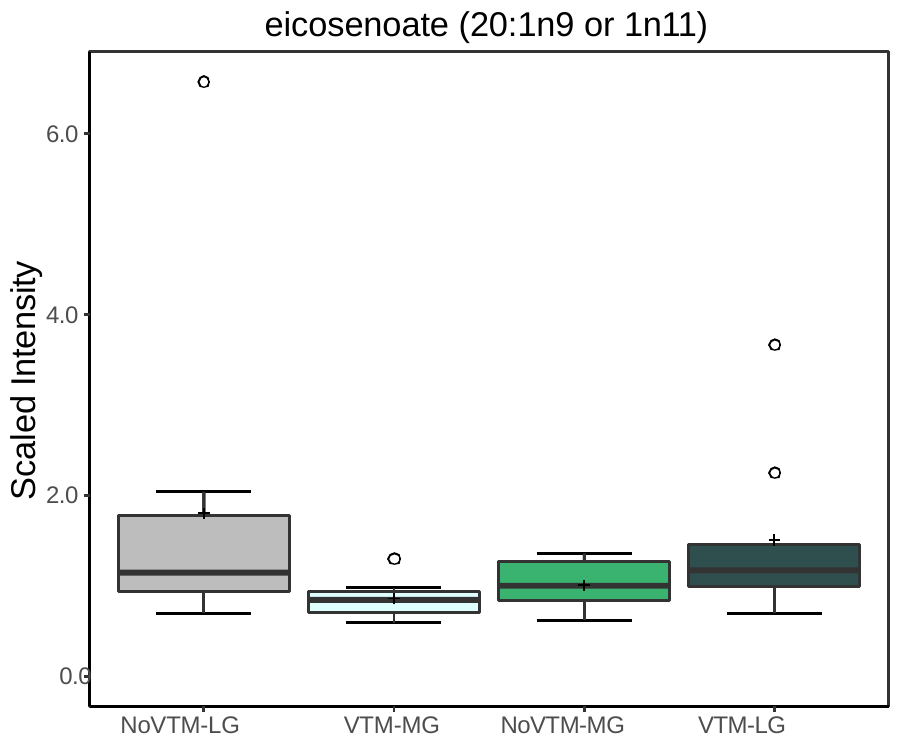

eicosenoate (20:1n9 or 1n11)
6.0
Scaled Intensity
4.0
2.0
0.0
NoVTM-LG
VTM-MG
NoVTM-MG
VTM-LG

## Slide 27
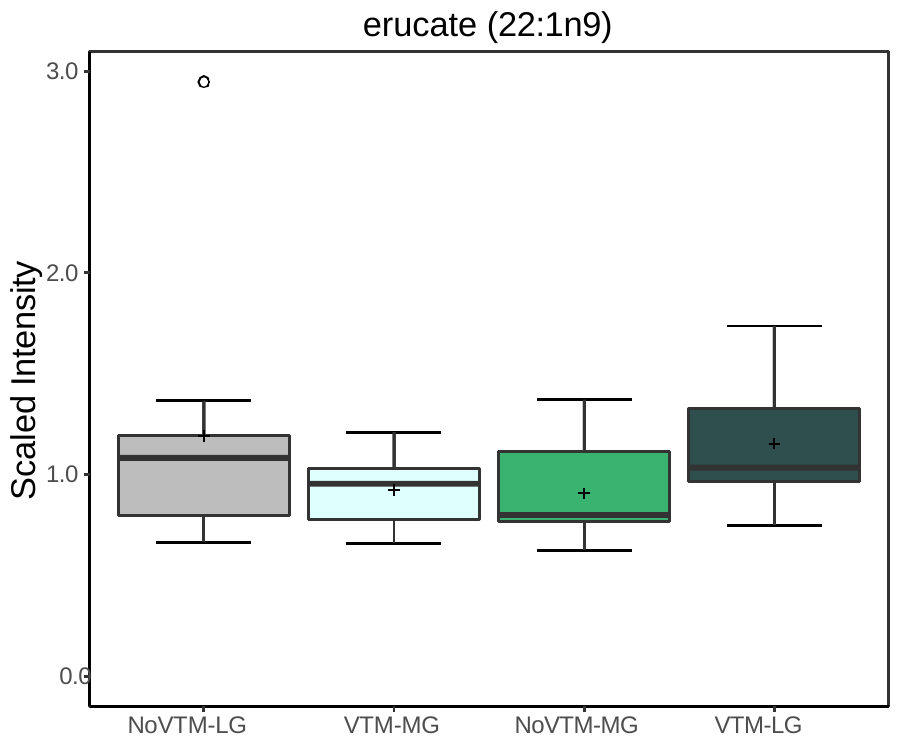

erucate (22:1n9)
3.0
2.0
Scaled Intensity
1.0
0.0
NoVTM-LG
VTM-MG
NoVTM-MG
VTM-LG

## Slide 28
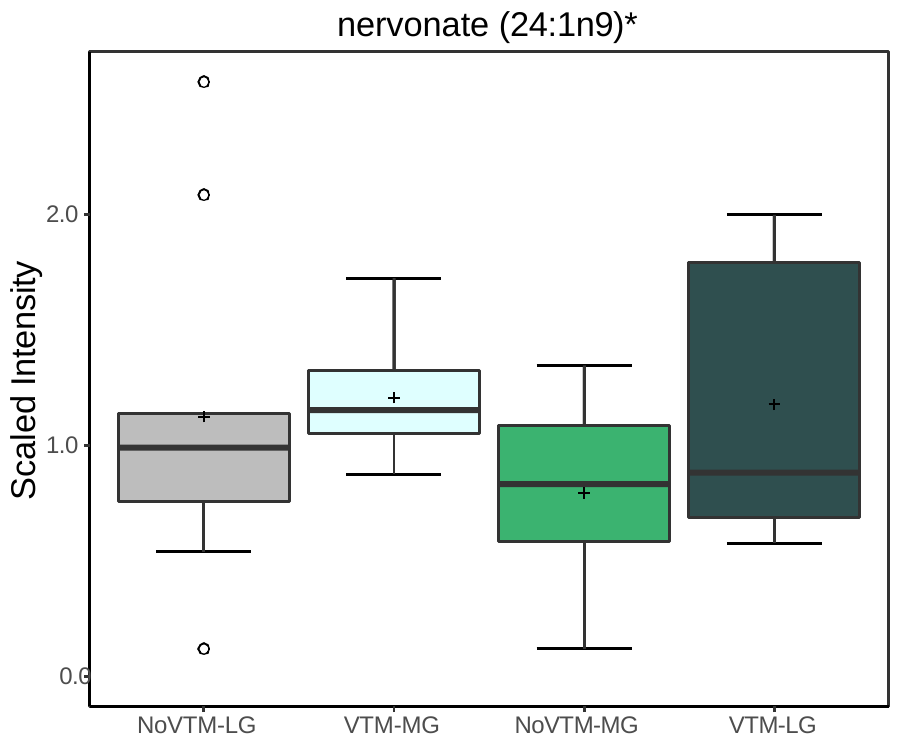

nervonate (24:1n9)*
2.0
Scaled Intensity
1.0
0.0
NoVTM-LG
VTM-MG
NoVTM-MG
VTM-LG

## Slide 29
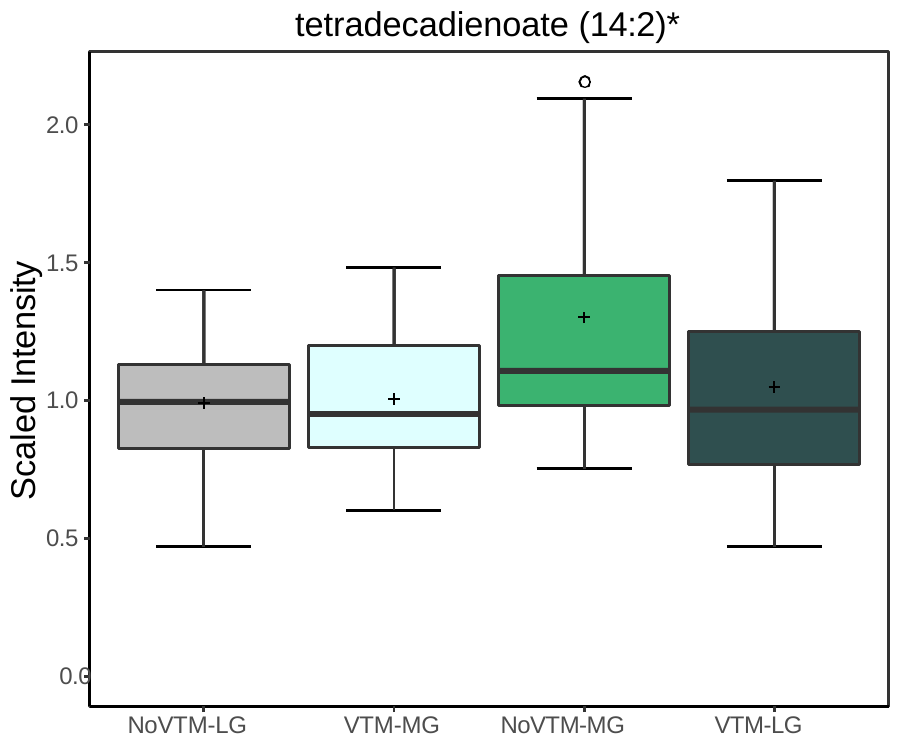

tetradecadienoate (14:2)*
2.0
1.5
Scaled Intensity
1.0
0.5
0.0
NoVTM-LG
VTM-MG
NoVTM-MG
VTM-LG

## Slide 30
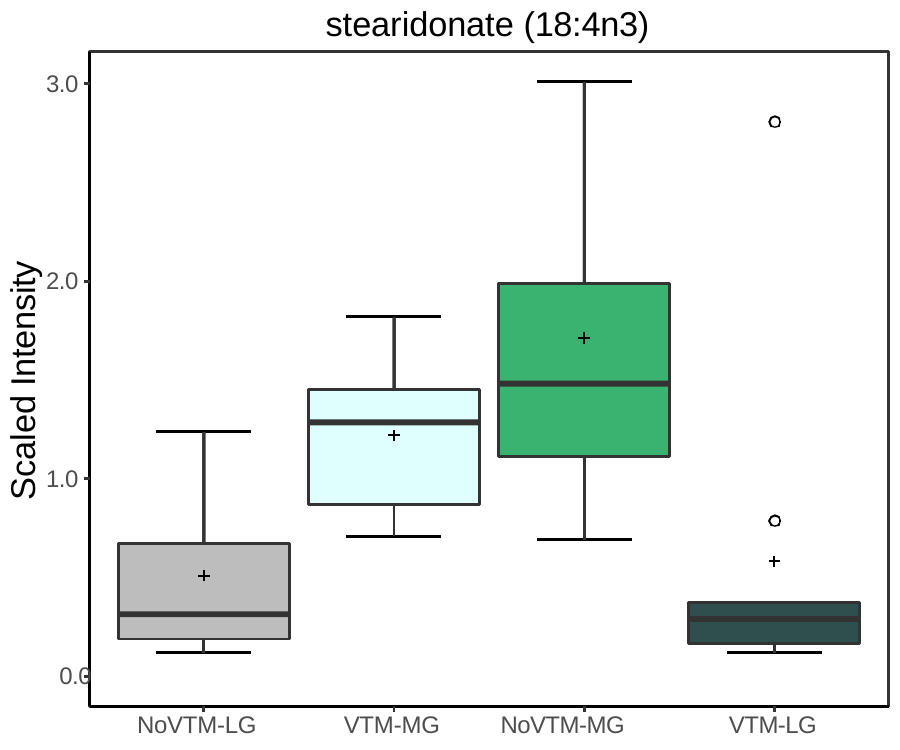

stearidonate (18:4n3)
3.0
Scaled Intensity
2.0
1.0
0.0
NoVTM-LG
VTM-MG
NoVTM-MG
VTM-LG

## Slide 31
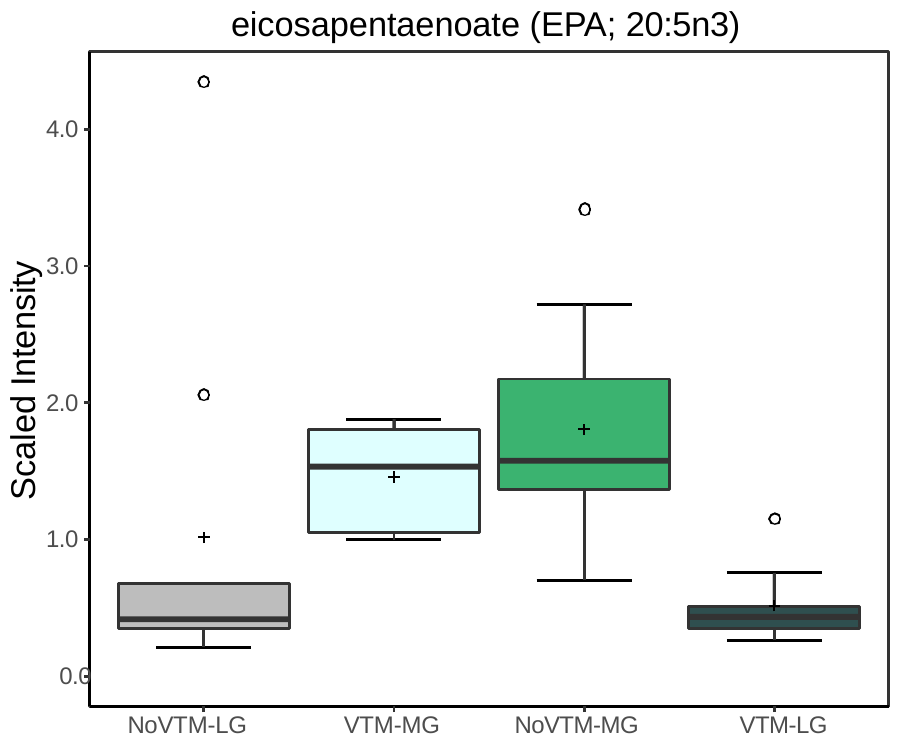

eicosapentaenoate (EPA; 20:5n3)
4.0
3.0
Scaled Intensity
2.0
1.0
0.0
NoVTM-LG
VTM-MG
NoVTM-MG
VTM-LG

## Slide 32
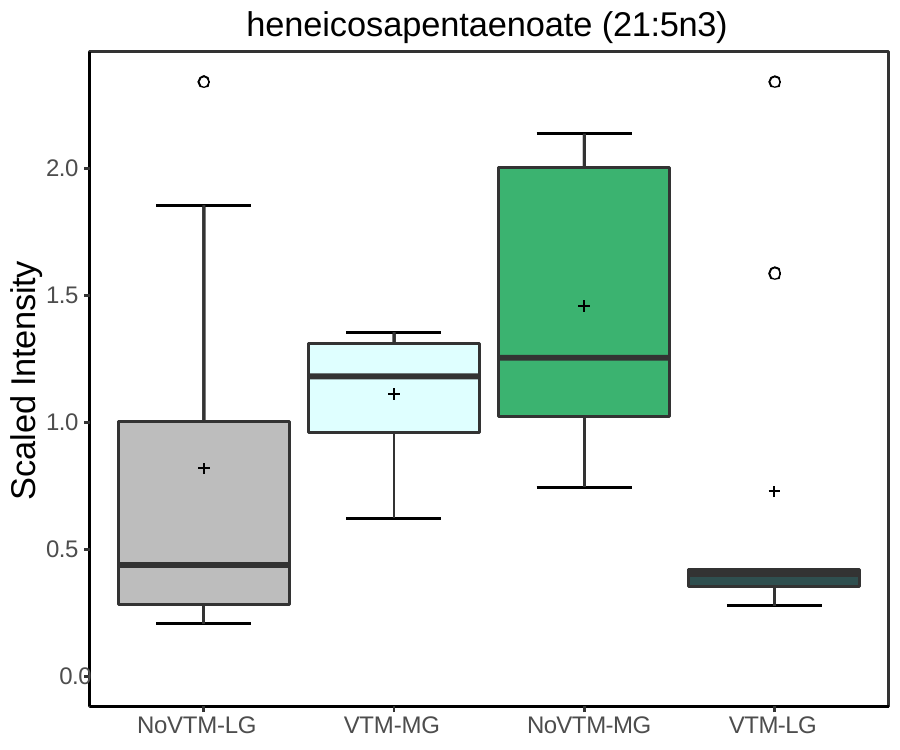

heneicosapentaenoate (21:5n3)
2.0
Scaled Intensity
1.5
1.0
0.5
0.0
NoVTM-LG
VTM-MG
NoVTM-MG
VTM-LG

## Slide 33
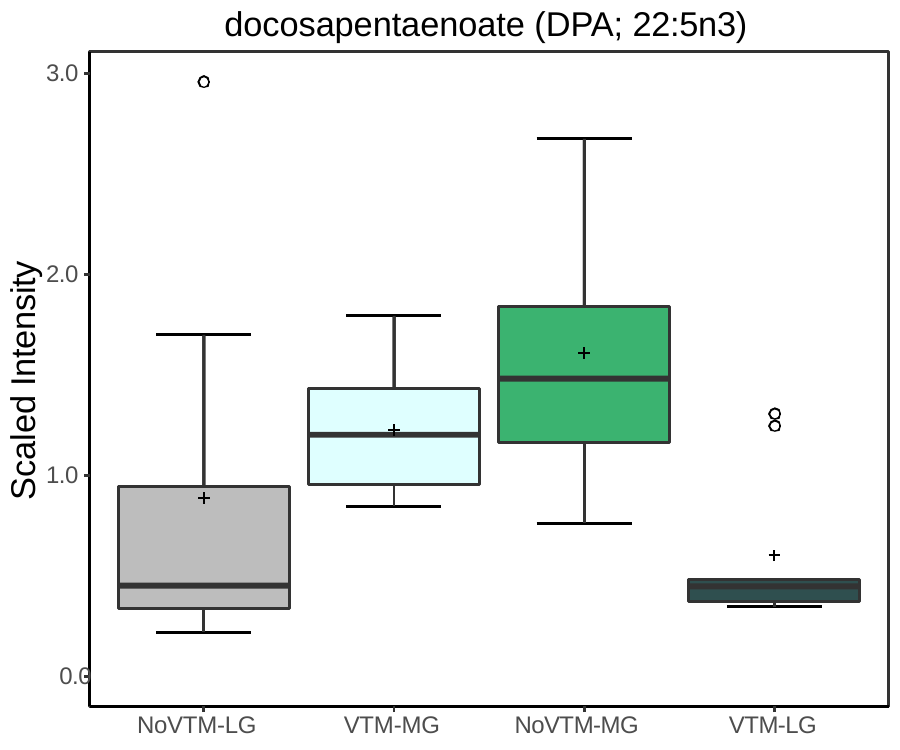

docosapentaenoate (DPA; 22:5n3)
3.0
Scaled Intensity
2.0
1.0
0.0
NoVTM-LG
VTM-MG
NoVTM-MG
VTM-LG

## Slide 34
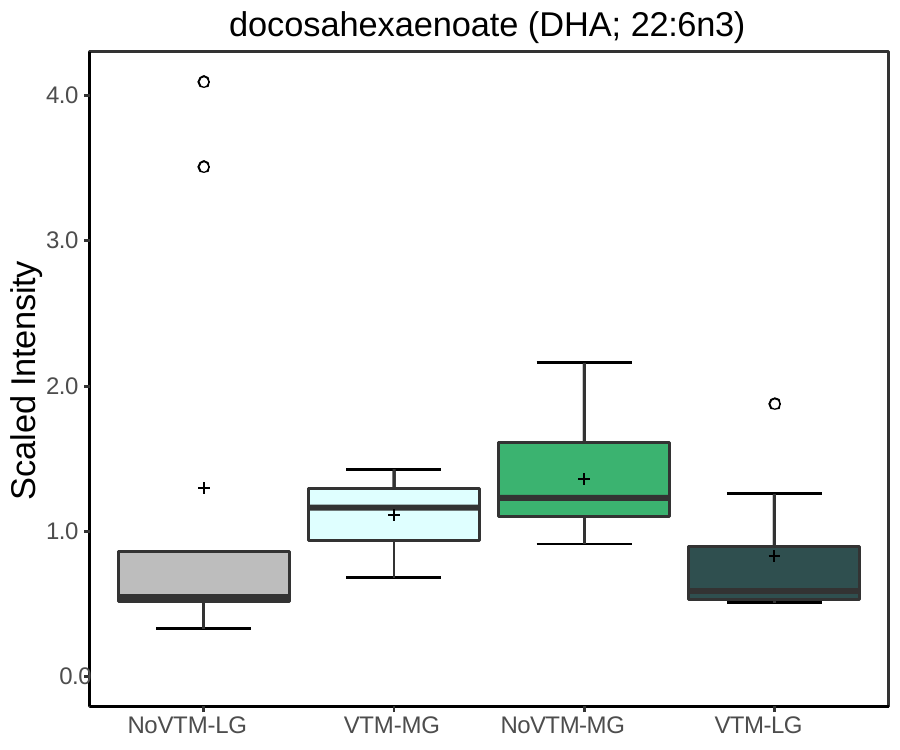

docosahexaenoate (DHA; 22:6n3)
4.0
3.0
Scaled Intensity
2.0
1.0
0.0
NoVTM-LG
VTM-MG
NoVTM-MG
VTM-LG

## Slide 35
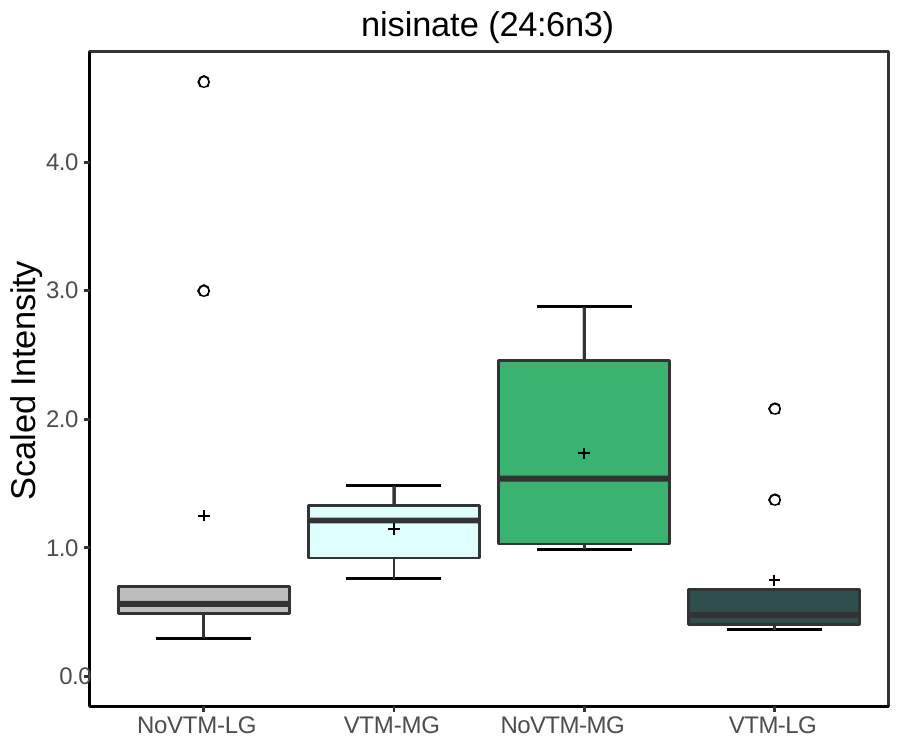

nisinate (24:6n3)
4.0
Scaled Intensity
3.0
2.0
1.0
0.0
NoVTM-LG
VTM-MG
NoVTM-MG
VTM-LG

## Slide 36
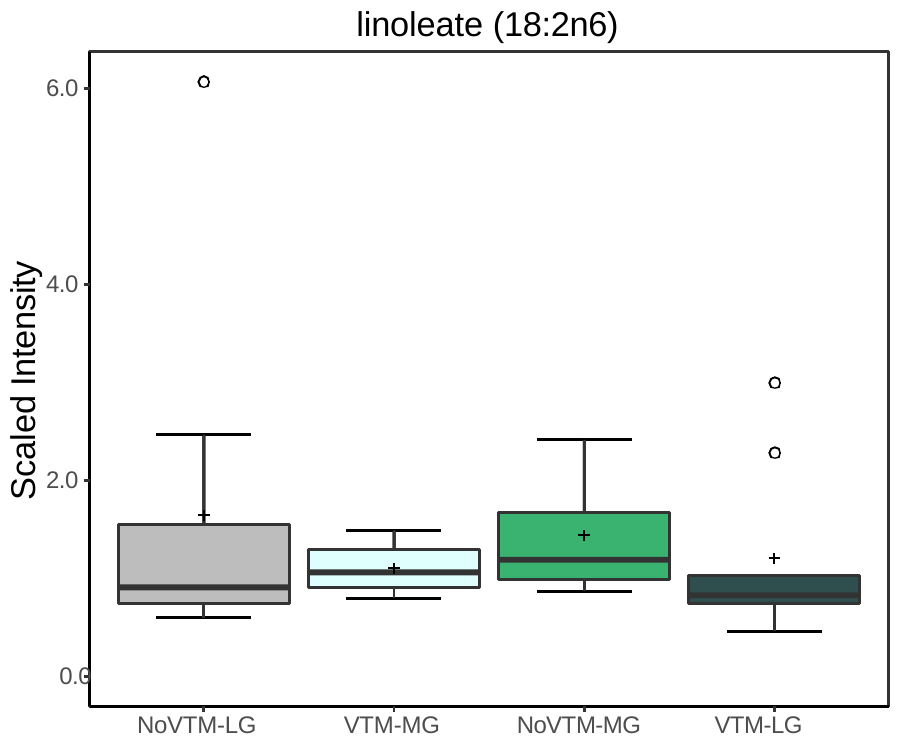

linoleate (18:2n6)
6.0
Scaled Intensity
4.0
2.0
0.0
NoVTM-LG
VTM-MG
NoVTM-MG
VTM-LG

## Slide 37
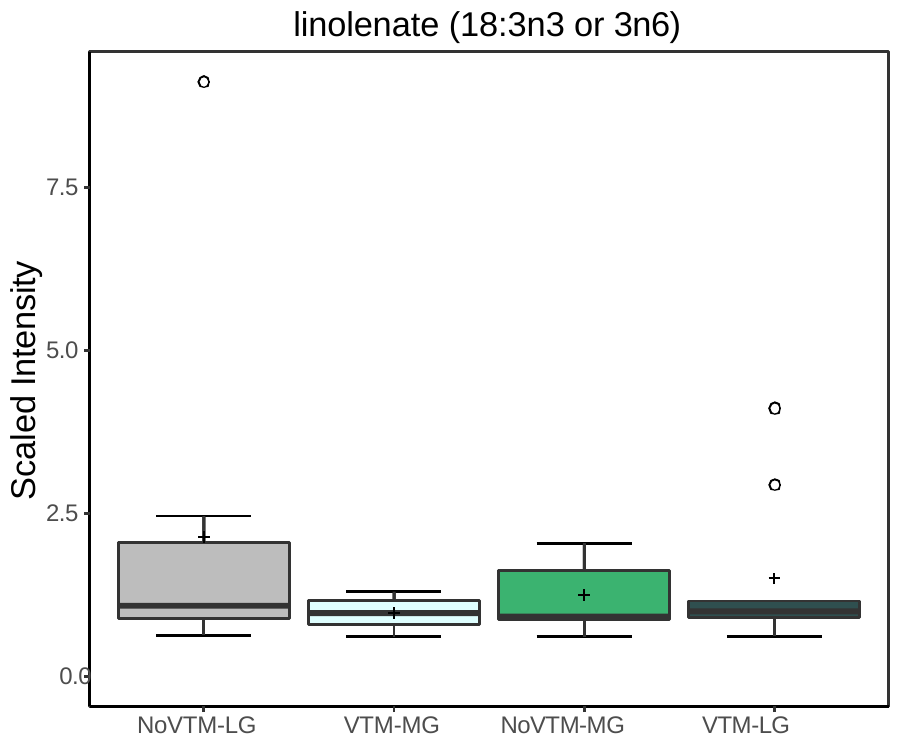

linolenate (18:3n3 or 3n6)
7.5
Scaled Intensity
5.0
2.5
0.0
NoVTM-LG
VTM-MG
NoVTM-MG
VTM-LG

## Slide 38
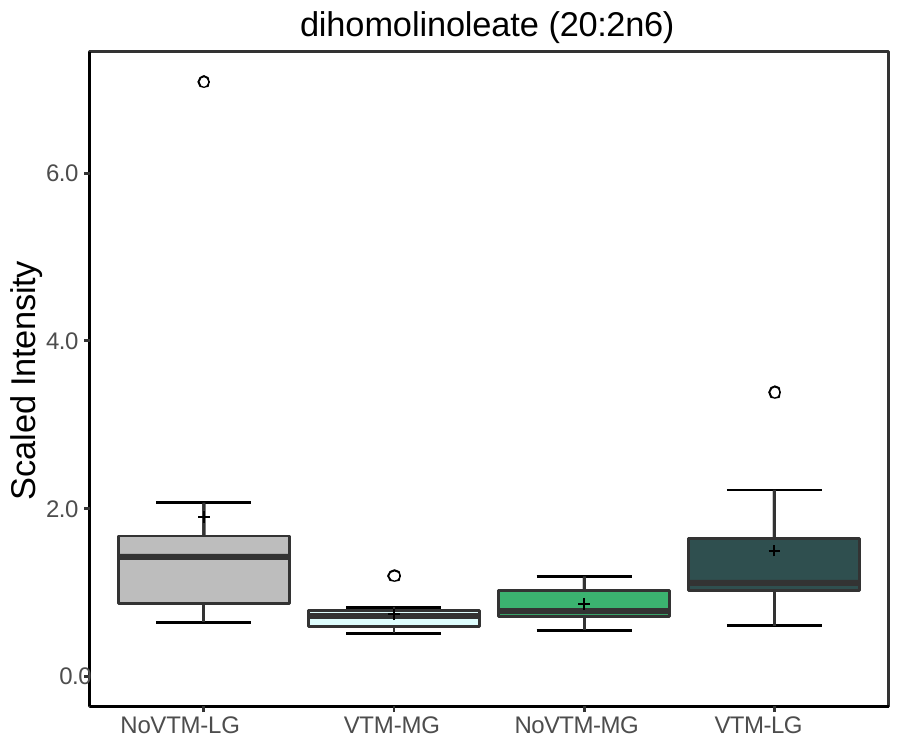

dihomolinoleate (20:2n6)
6.0
Scaled Intensity
4.0
2.0
0.0
NoVTM-LG
VTM-MG
NoVTM-MG
VTM-LG

## Slide 39
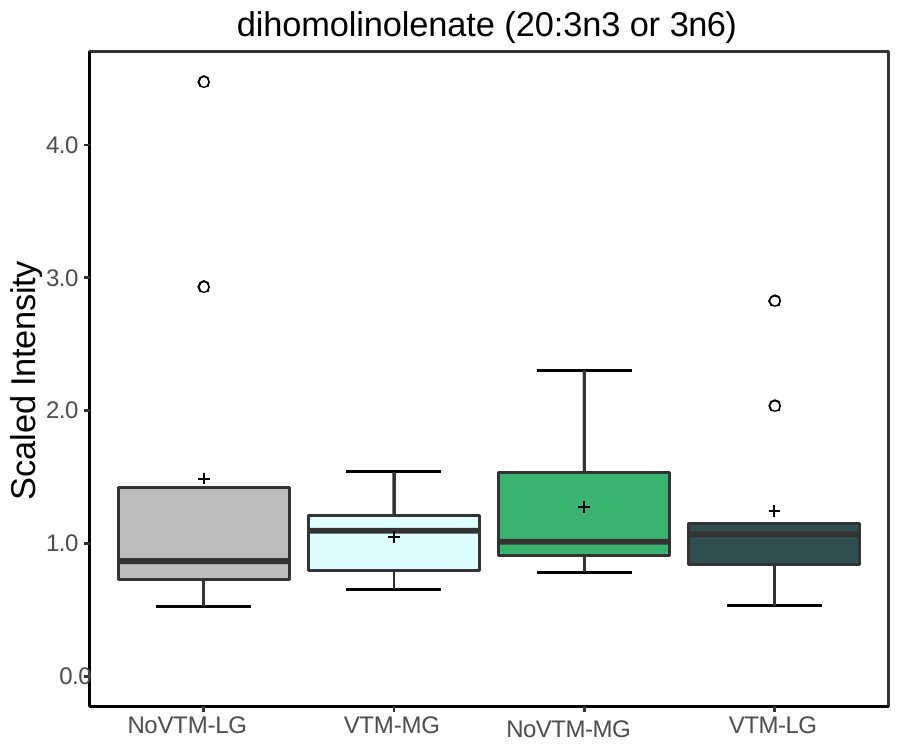

dihomolinolenate (20:3n3 or 3n6)
4.0
Scaled Intensity
3.0
2.0
1.0
0.0
NoVTM-LG
VTM-MG
VTM-LG
NoVTM-MG

## Slide 40
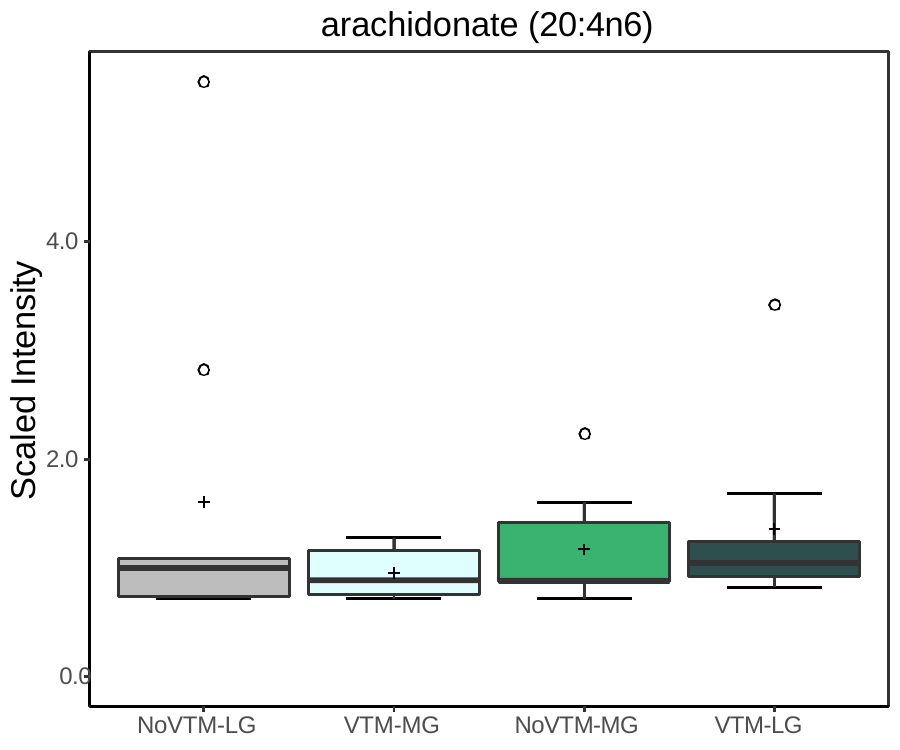

arachidonate (20:4n6)
4.0
Scaled Intensity
2.0
0.0
NoVTM-LG
VTM-MG
NoVTM-MG
VTM-LG

## Slide 41
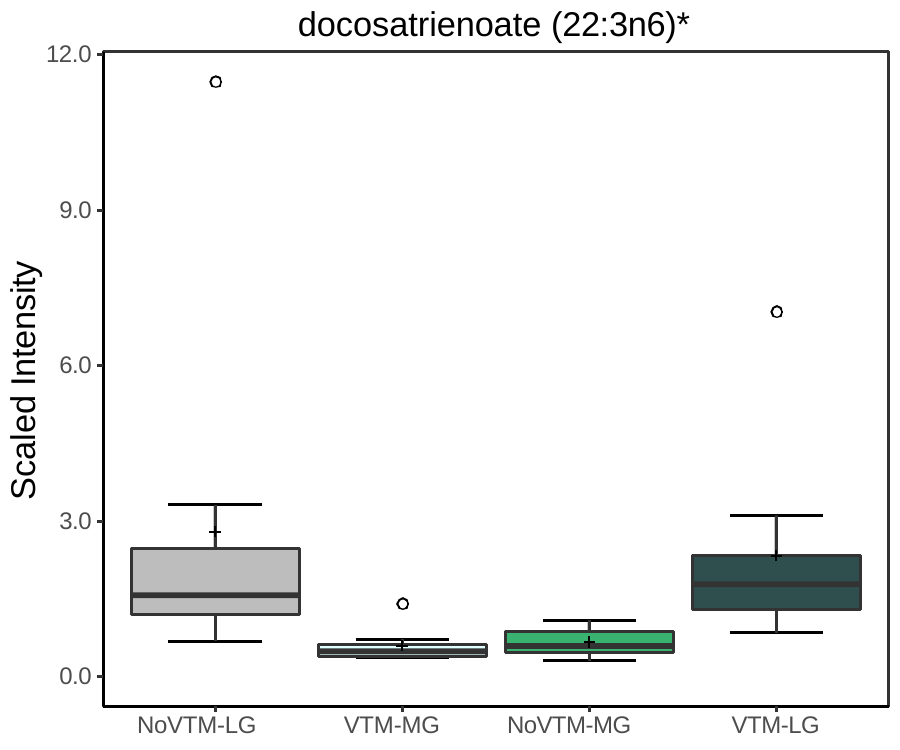

docosatrienoate (22:3n6)*
12.0
9.0
Scaled Intensity
6.0
3.0
0.0
NoVTM-LG
VTM-MG
NoVTM-MG
VTM-LG

## Slide 42
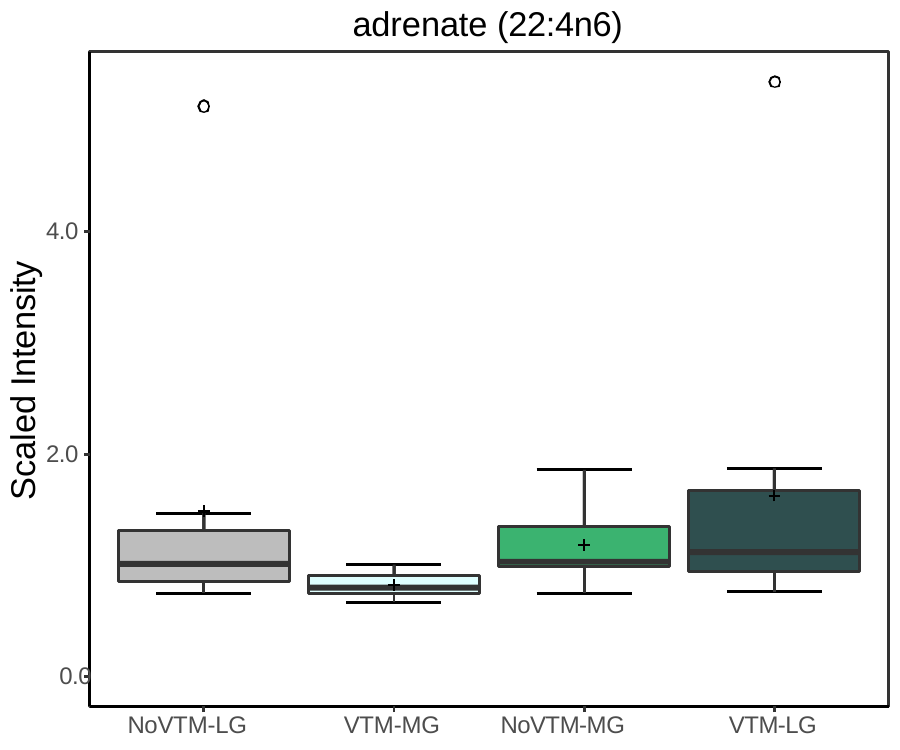

adrenate (22:4n6)
4.0
Scaled Intensity
2.0
0.0
NoVTM-LG
VTM-MG
NoVTM-MG
VTM-LG

## Slide 43
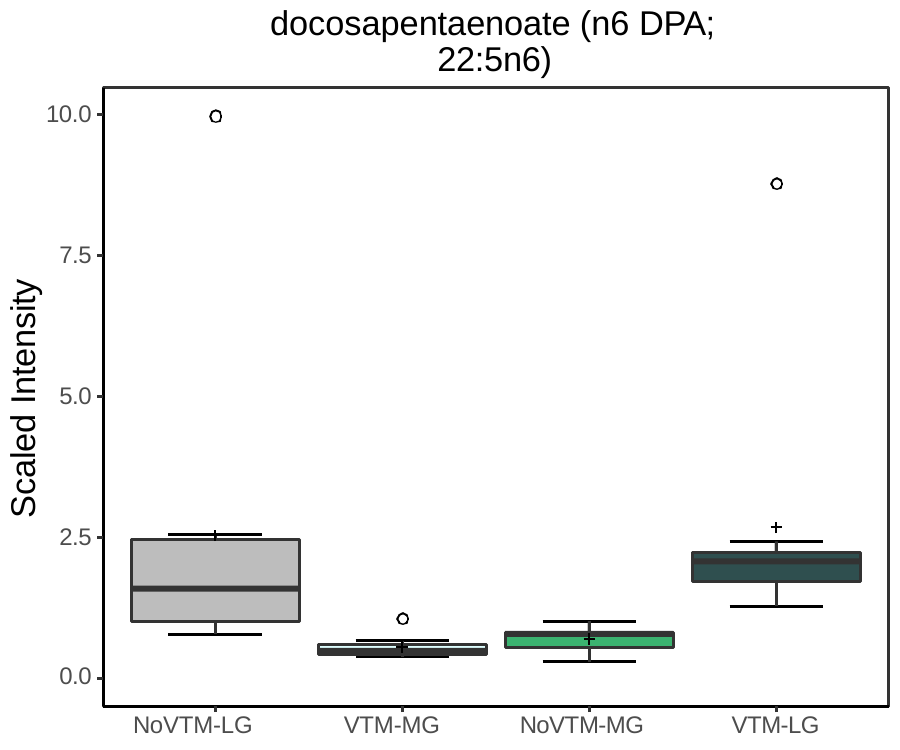

# docosapentaenoate (n6 DPA; 22:5n6)
10.0
7.5
Scaled Intensity
5.0
2.5
0.0
NoVTM-LG
VTM-MG
NoVTM-MG
VTM-LG

## Slide 44
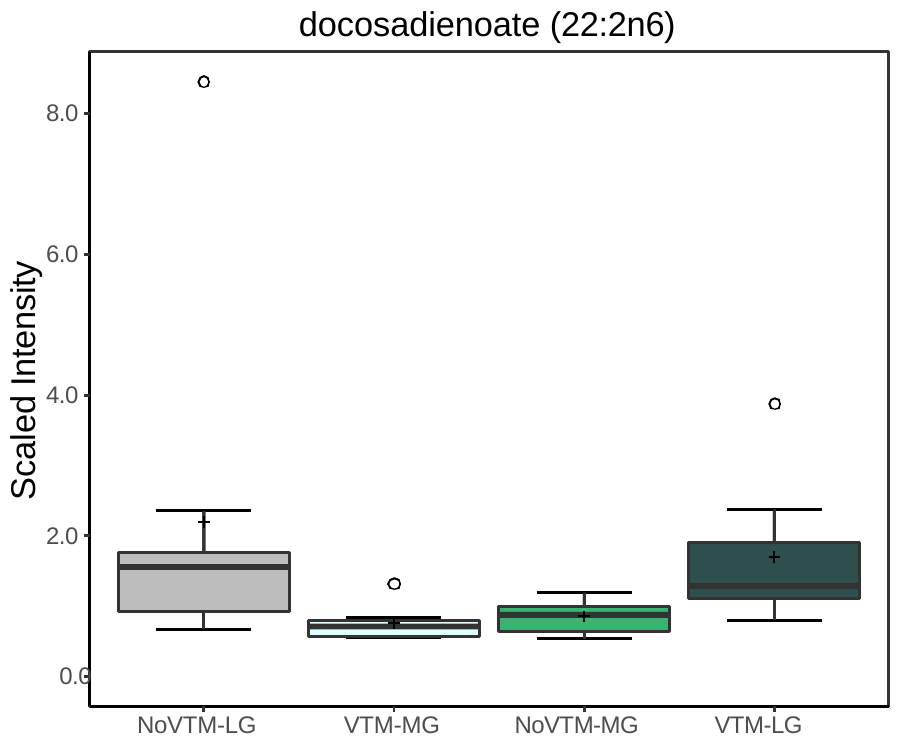

docosadienoate (22:2n6)
8.0
6.0
Scaled Intensity
4.0
2.0
0.0
NoVTM-LG
VTM-MG
NoVTM-MG
VTM-LG

## Slide 45
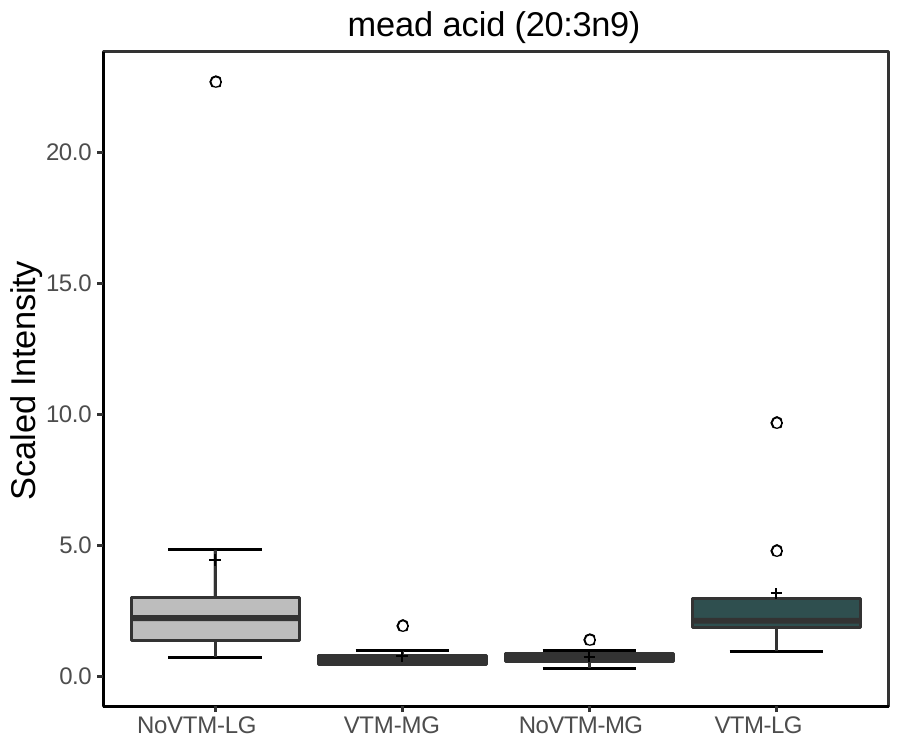

mead acid (20:3n9)
20.0
Scaled Intensity
15.0
10.0
5.0
0.0
NoVTM-LG
VTM-MG
NoVTM-MG
VTM-LG

## Slide 46
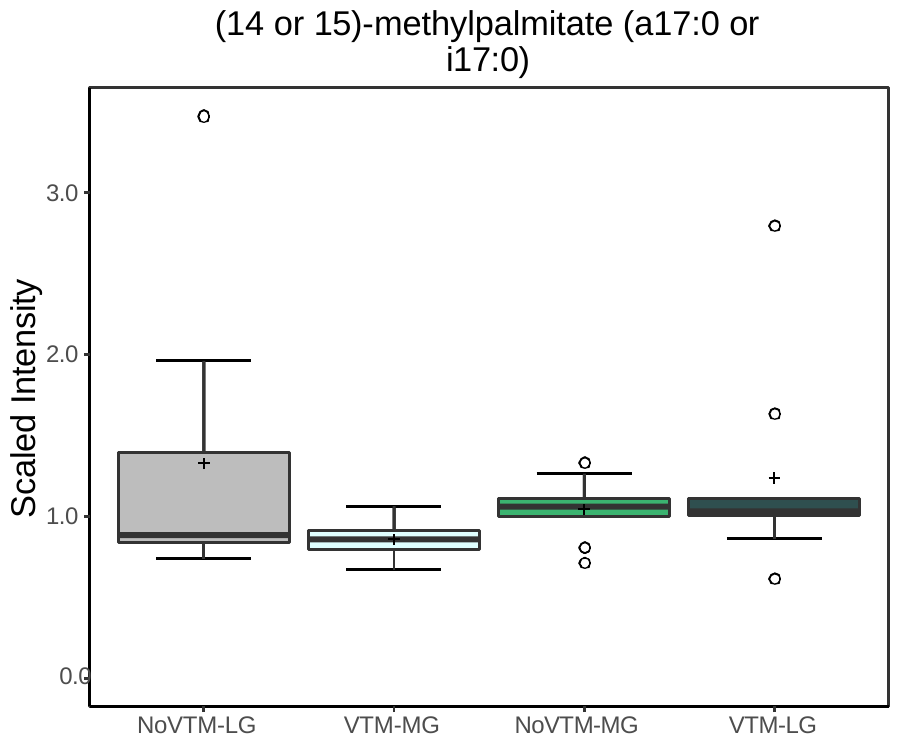

# (14 or 15)-methylpalmitate (a17:0 or i17:0)
3.0
Scaled Intensity
2.0
1.0
0.0
NoVTM-LG
VTM-MG
NoVTM-MG
VTM-LG

## Slide 47
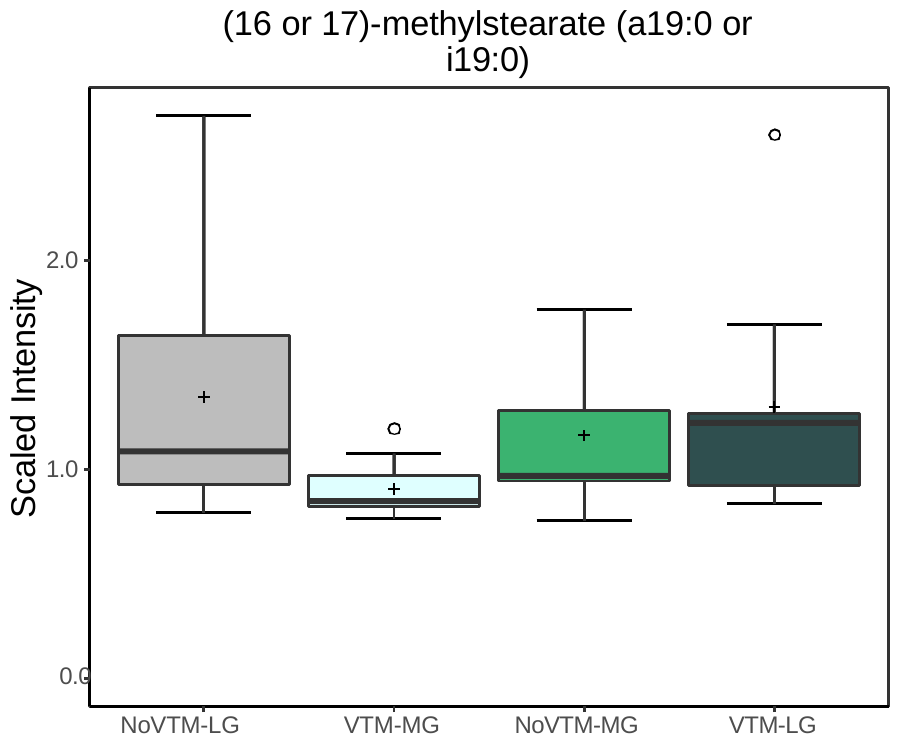

# (16 or 17)-methylstearate (a19:0 or i19:0)
2.0
Scaled Intensity
1.0
0.0
NoVTM-LG
VTM-MG
NoVTM-MG
VTM-LG

## Slide 48
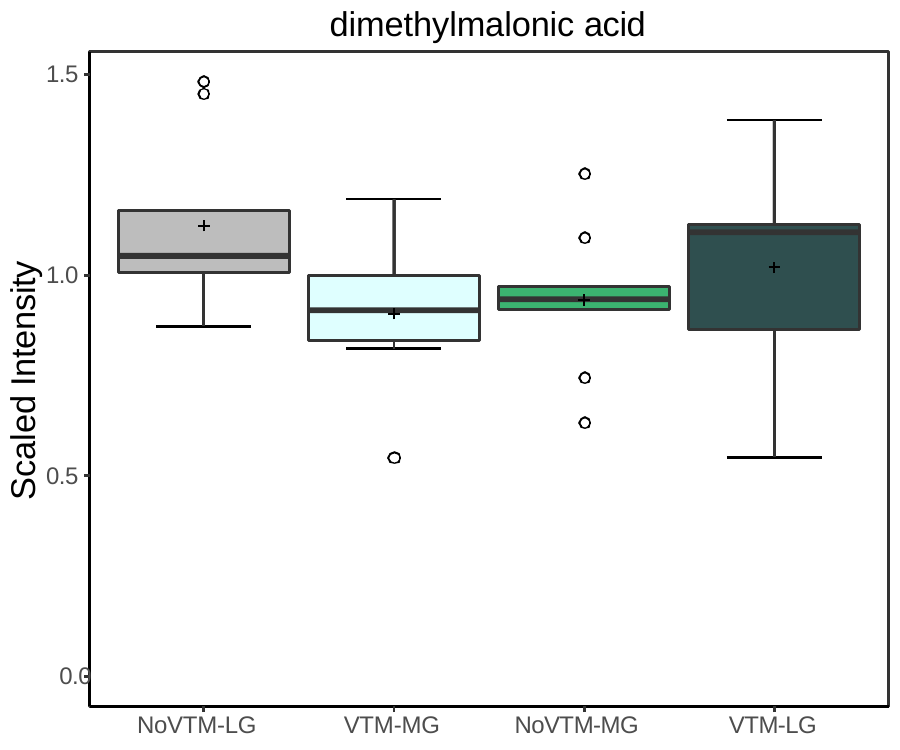

dimethylmalonic acid
1.5
Scaled Intensity
1.0
0.5
0.0
NoVTM-LG
VTM-MG
NoVTM-MG
VTM-LG

## Slide 49
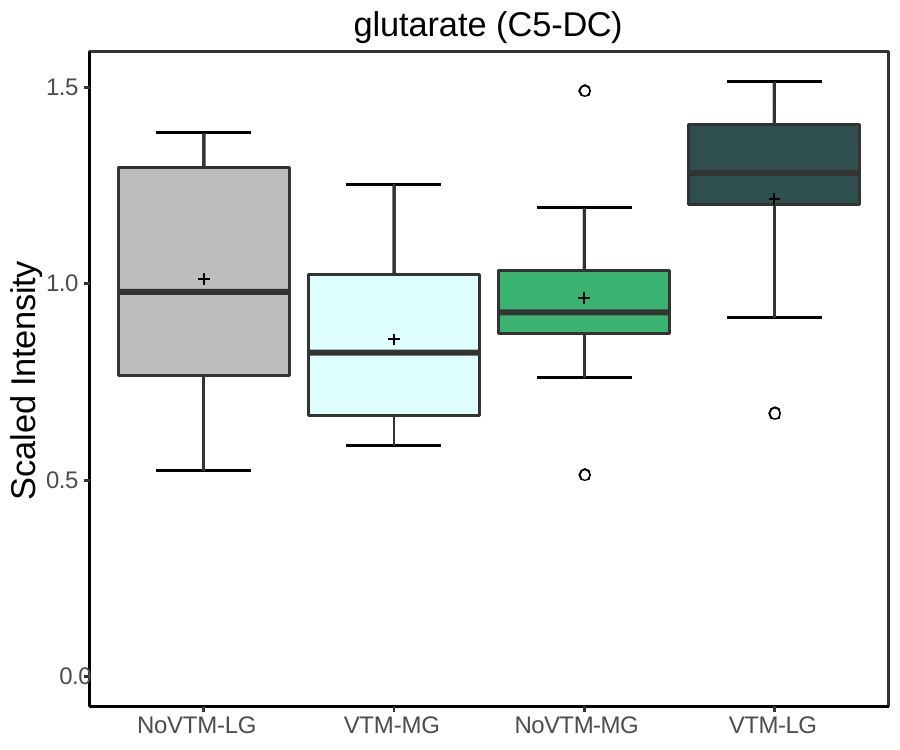

glutarate (C5-DC)
1.5
Scaled Intensity
1.0
0.5
0.0
NoVTM-LG
VTM-MG
NoVTM-MG
VTM-LG

## Slide 50
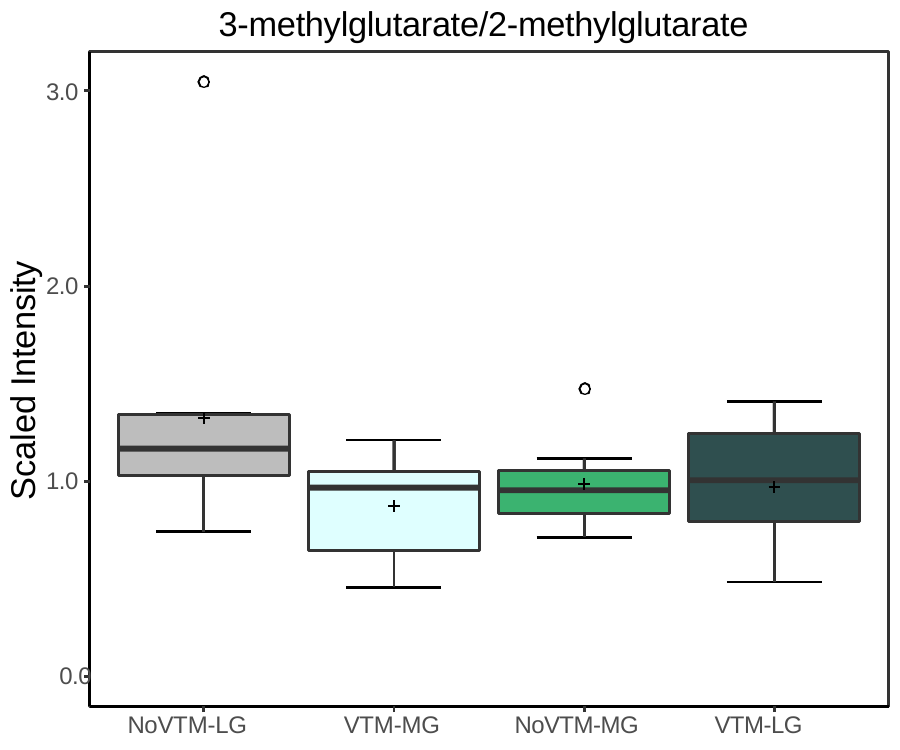

3-methylglutarate/2-methylglutarate
3.0
Scaled Intensity
2.0
1.0
0.0
NoVTM-LG
VTM-MG
NoVTM-MG
VTM-LG

## Slide 51
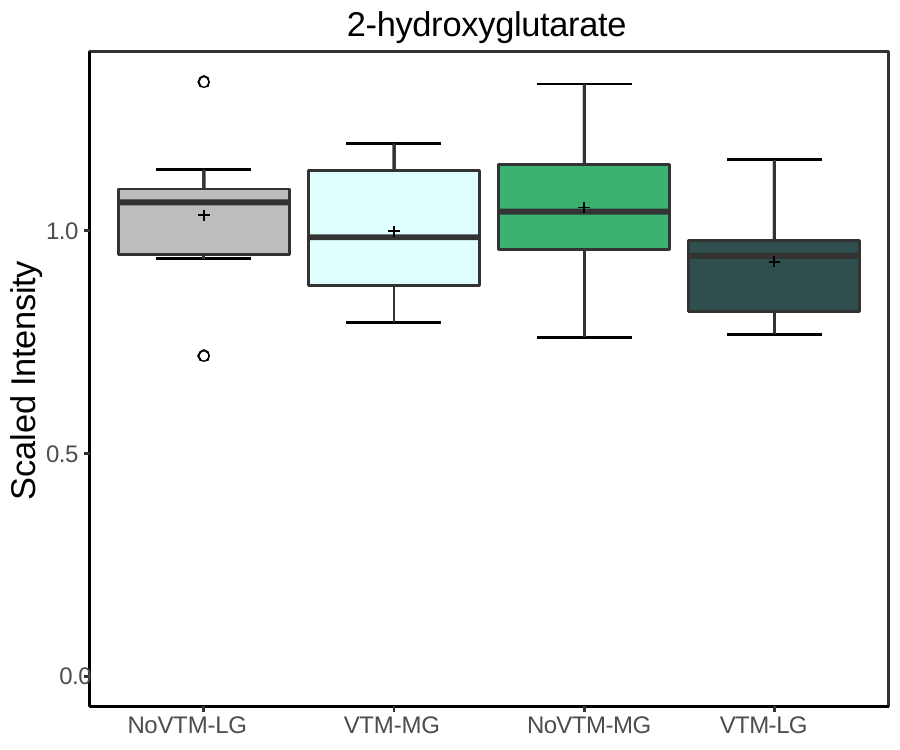

2-hydroxyglutarate
1.0
Scaled Intensity
0.5
0.0
NoVTM-LG
VTM-MG
NoVTM-MG
VTM-LG

## Slide 52
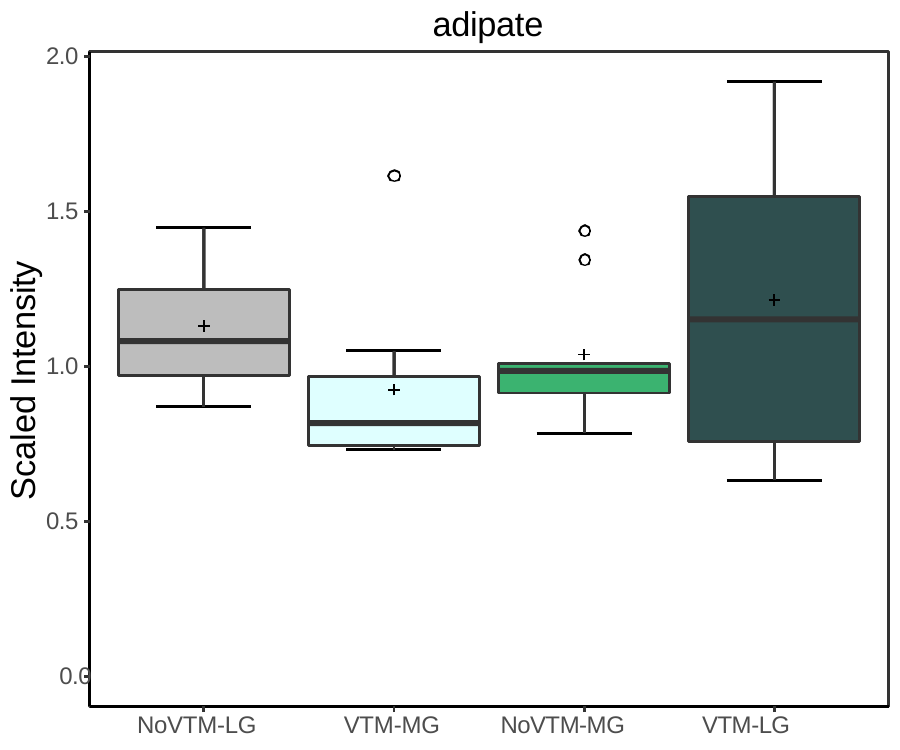

adipate
2.0
1.5
Scaled Intensity
1.0
0.5
0.0
NoVTM-LG
VTM-MG
NoVTM-MG
VTM-LG

## Slide 53
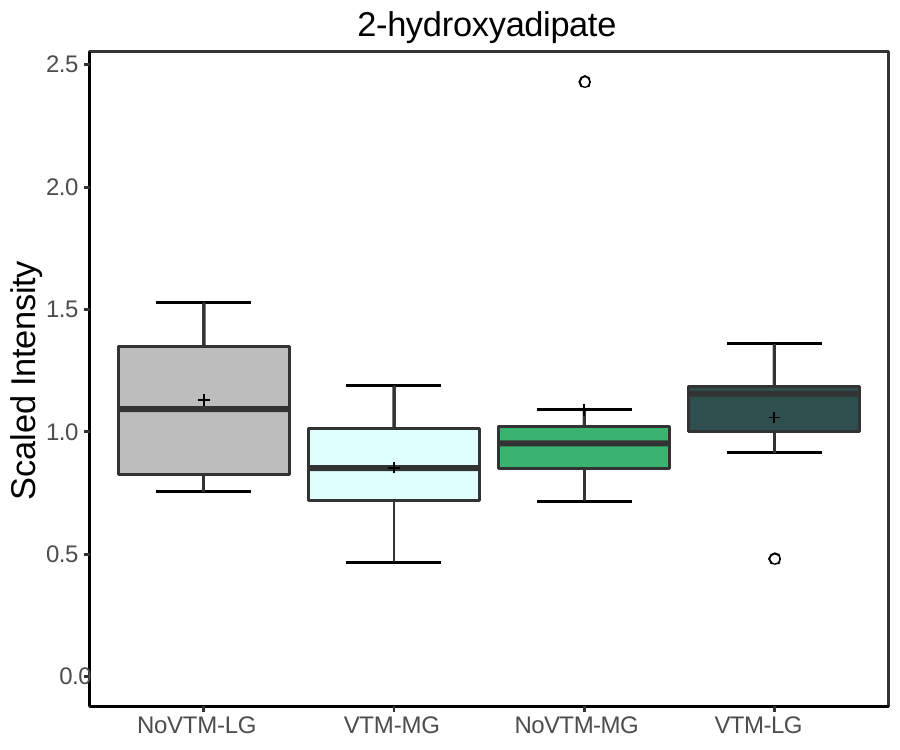

2-hydroxyadipate
2.5
2.0
Scaled Intensity
1.5
1.0
0.5
0.0
NoVTM-LG
VTM-MG
NoVTM-MG
VTM-LG

## Slide 54
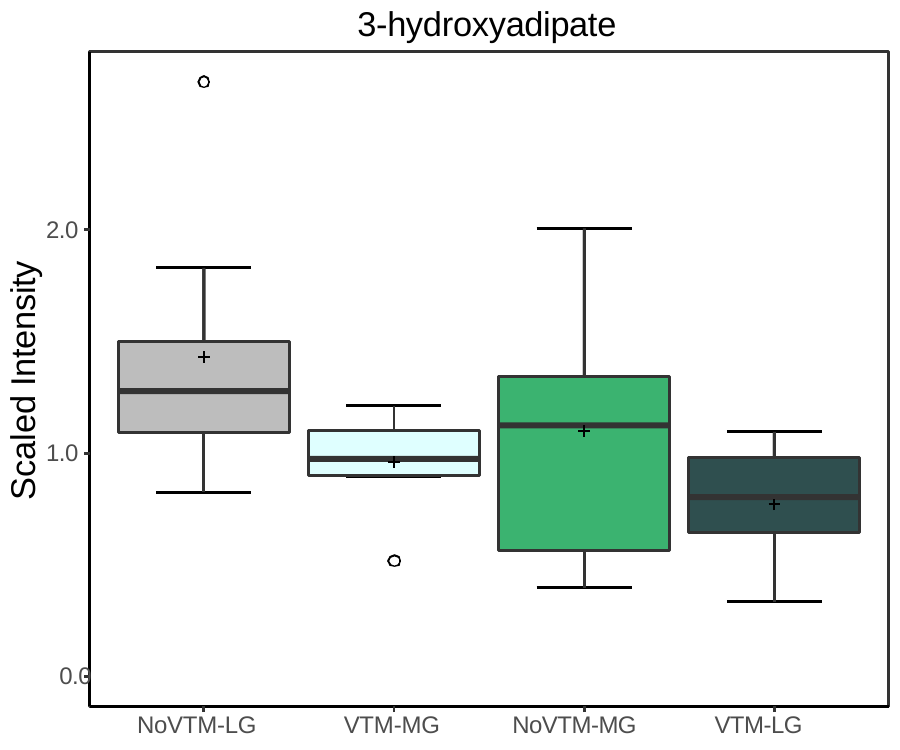

3-hydroxyadipate
2.0
Scaled Intensity
1.0
0.0
NoVTM-LG
VTM-MG
NoVTM-MG
VTM-LG

## Slide 55
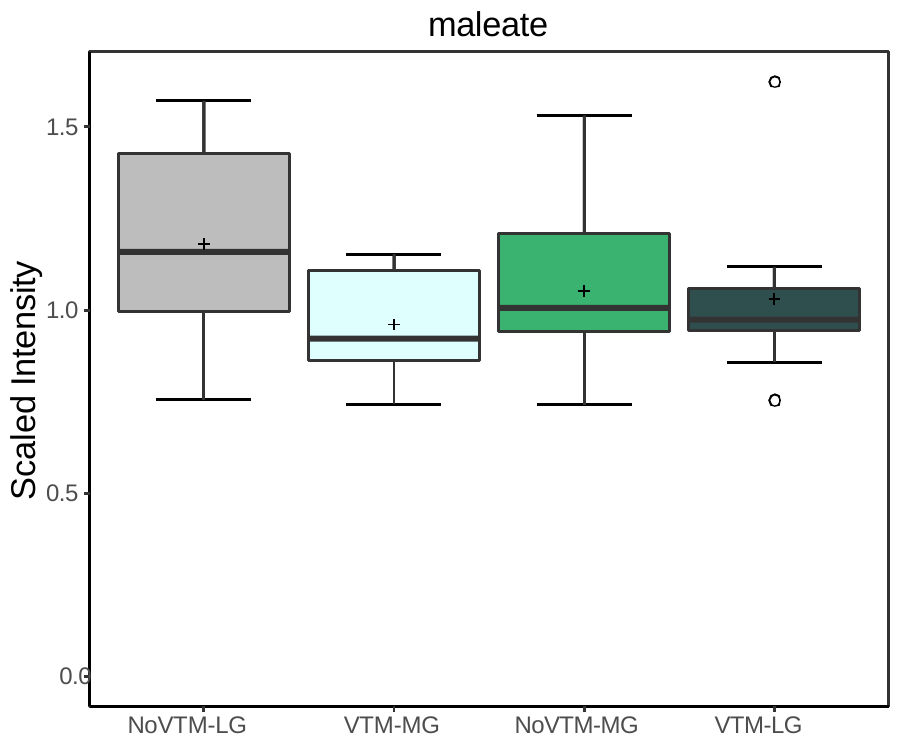

maleate
1.5
Scaled Intensity
1.0
0.5
0.0
NoVTM-LG
VTM-MG
NoVTM-MG
VTM-LG

## Slide 56
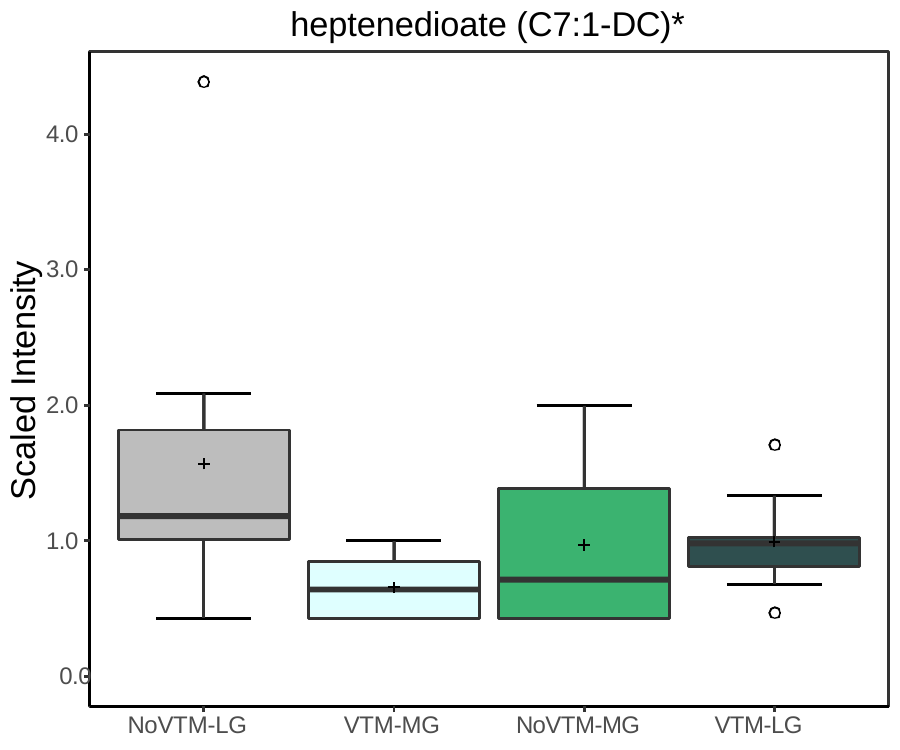

heptenedioate (C7:1-DC)*
4.0
3.0
Scaled Intensity
2.0
1.0
0.0
NoVTM-LG
VTM-MG
NoVTM-MG
VTM-LG

## Slide 57
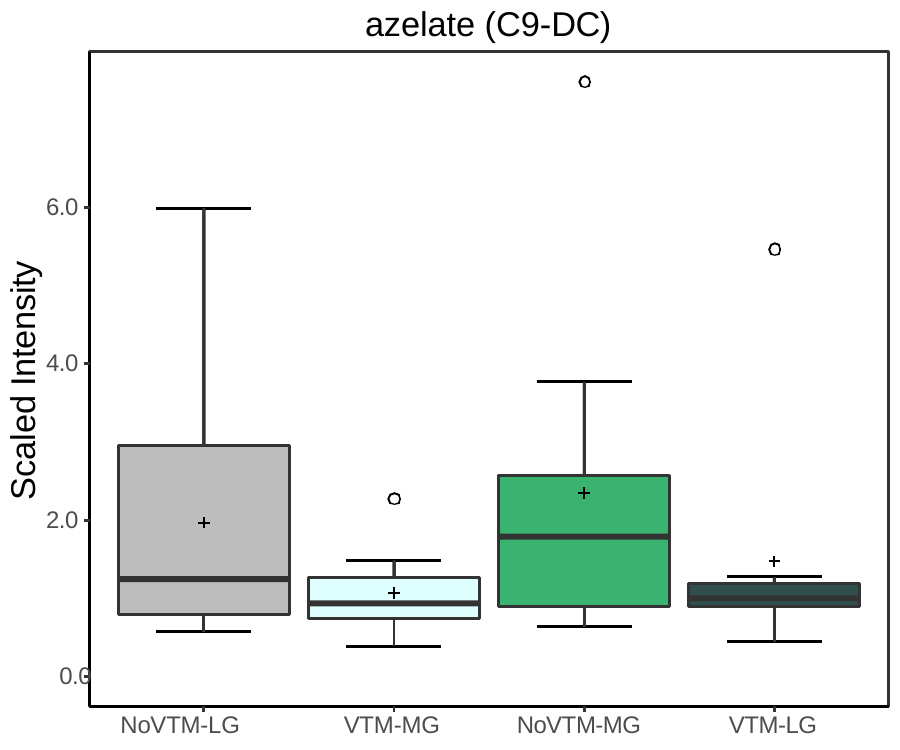

azelate (C9-DC)
6.0
Scaled Intensity
4.0
2.0
0.0
NoVTM-LG
VTM-MG
NoVTM-MG
VTM-LG

## Slide 58
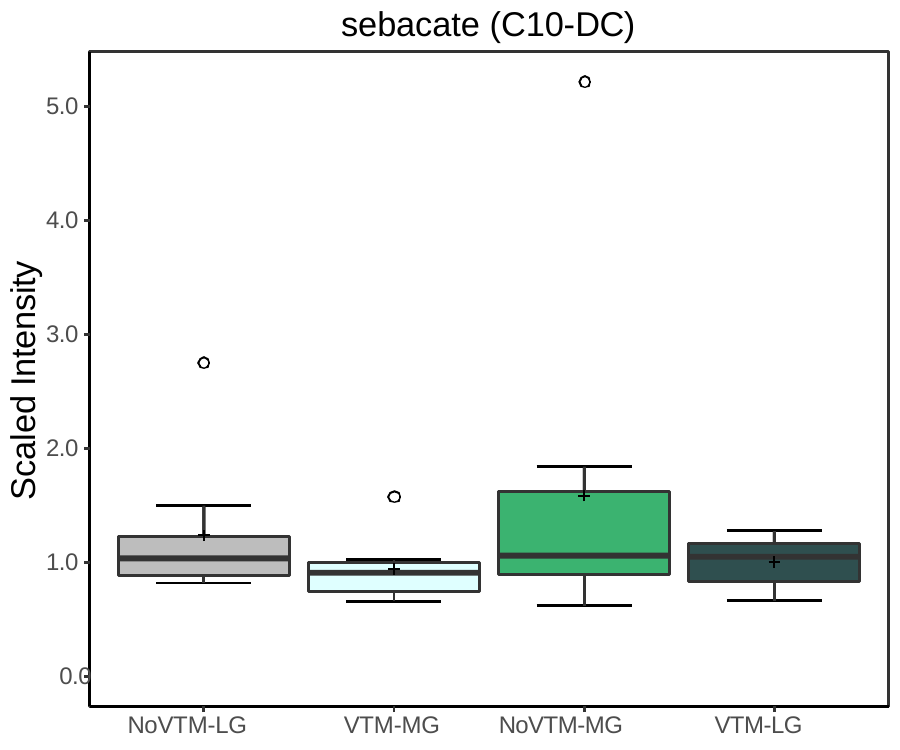

sebacate (C10-DC)
5.0
4.0
Scaled Intensity
3.0
2.0
1.0
0.0
NoVTM-LG
VTM-MG
NoVTM-MG
VTM-LG

## Slide 59
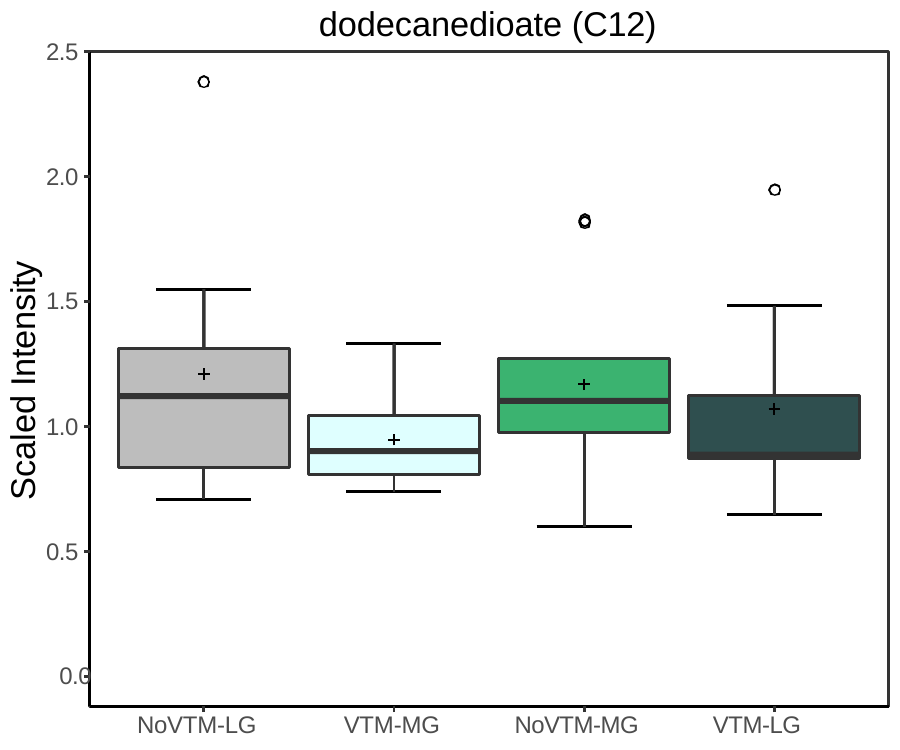

dodecanedioate (C12)
2.5
2.0
Scaled Intensity
1.5
1.0
0.5
0.0
NoVTM-LG
VTM-MG
NoVTM-MG
VTM-LG

## Slide 60
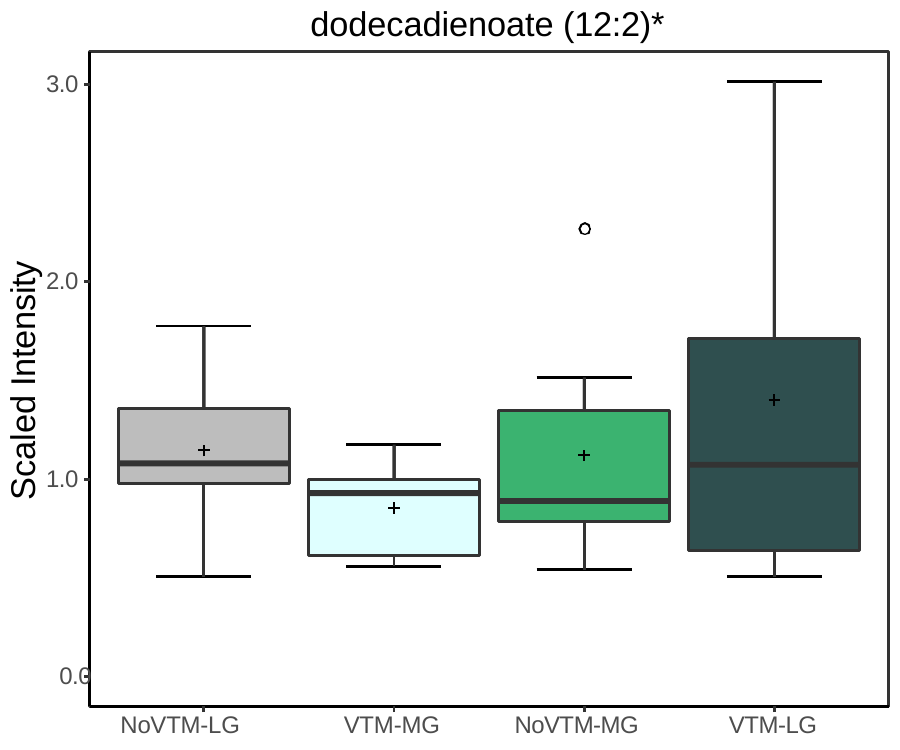

dodecadienoate (12:2)*
3.0
Scaled Intensity
2.0
1.0
0.0
NoVTM-LG
VTM-MG
NoVTM-MG
VTM-LG

## Slide 61
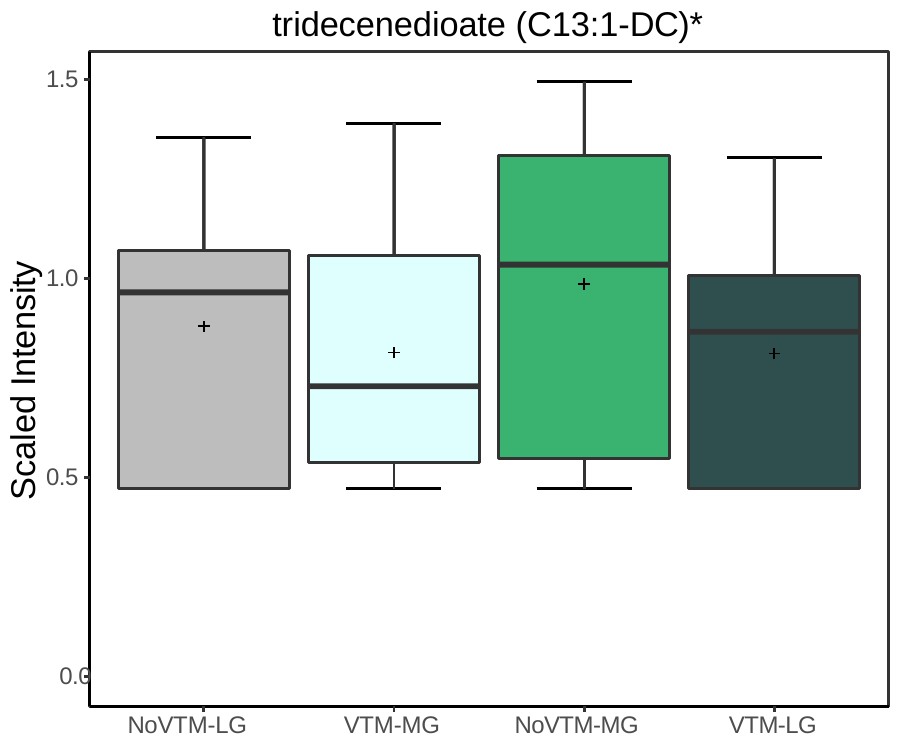

tridecenedioate (C13:1-DC)*
1.5
Scaled Intensity
1.0
0.5
0.0
NoVTM-LG
VTM-MG
NoVTM-MG
VTM-LG

## Slide 62
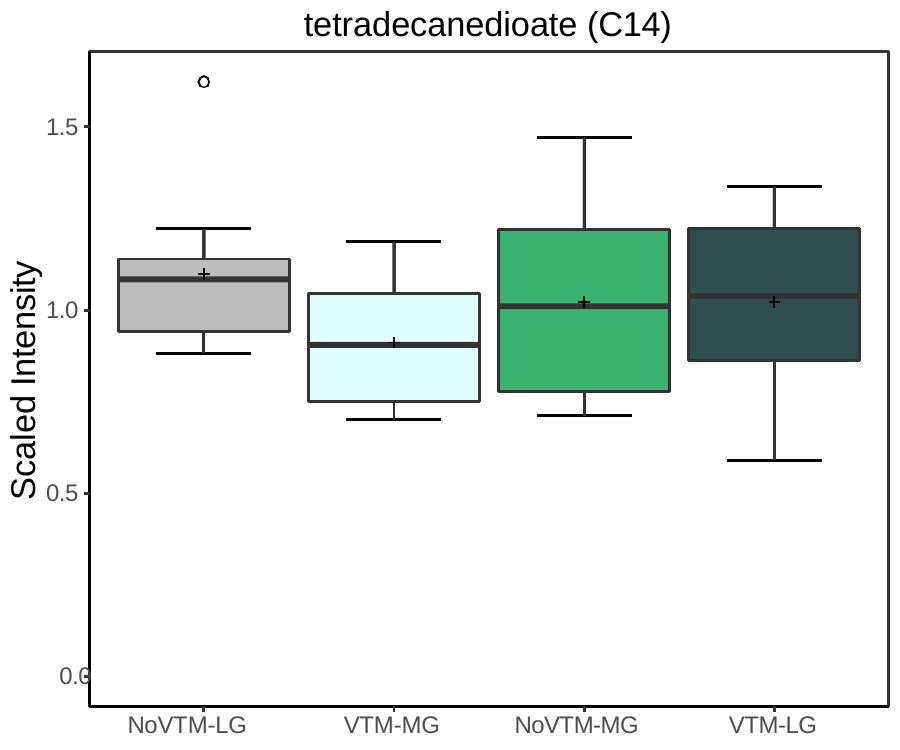

tetradecanedioate (C14)
1.5
Scaled Intensity
1.0
0.5
0.0
NoVTM-LG
VTM-MG
NoVTM-MG
VTM-LG

## Slide 63
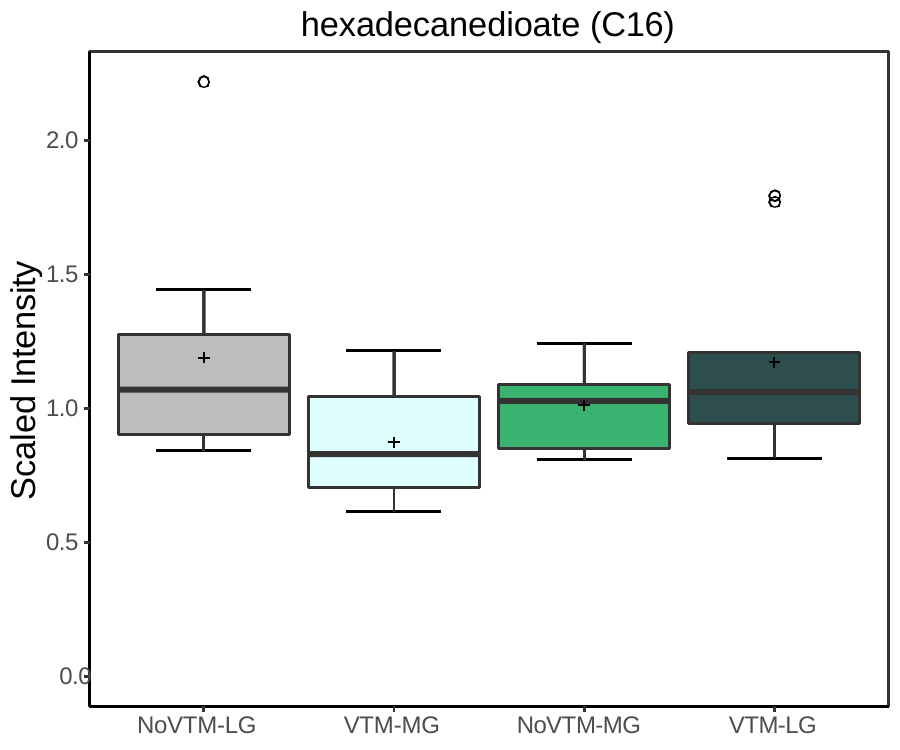

hexadecanedioate (C16)
2.0
Scaled Intensity
1.5
1.0
0.5
0.0
NoVTM-LG
VTM-MG
NoVTM-MG
VTM-LG

## Slide 64
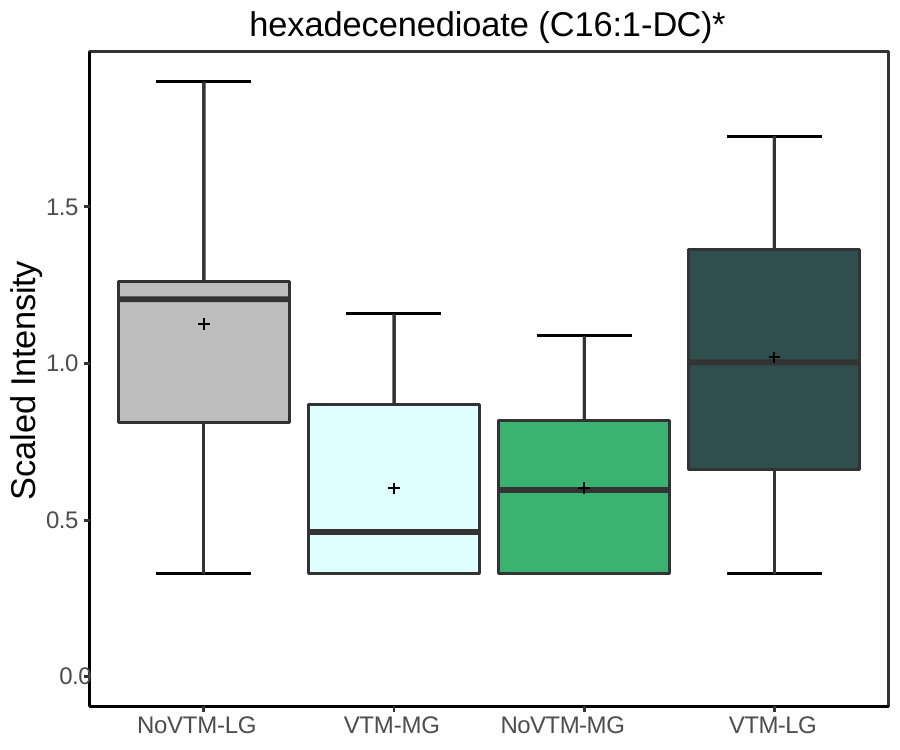

hexadecenedioate (C16:1-DC)*
1.5
Scaled Intensity
1.0
0.5
0.0
NoVTM-LG
VTM-MG
NoVTM-MG
VTM-LG

## Slide 65
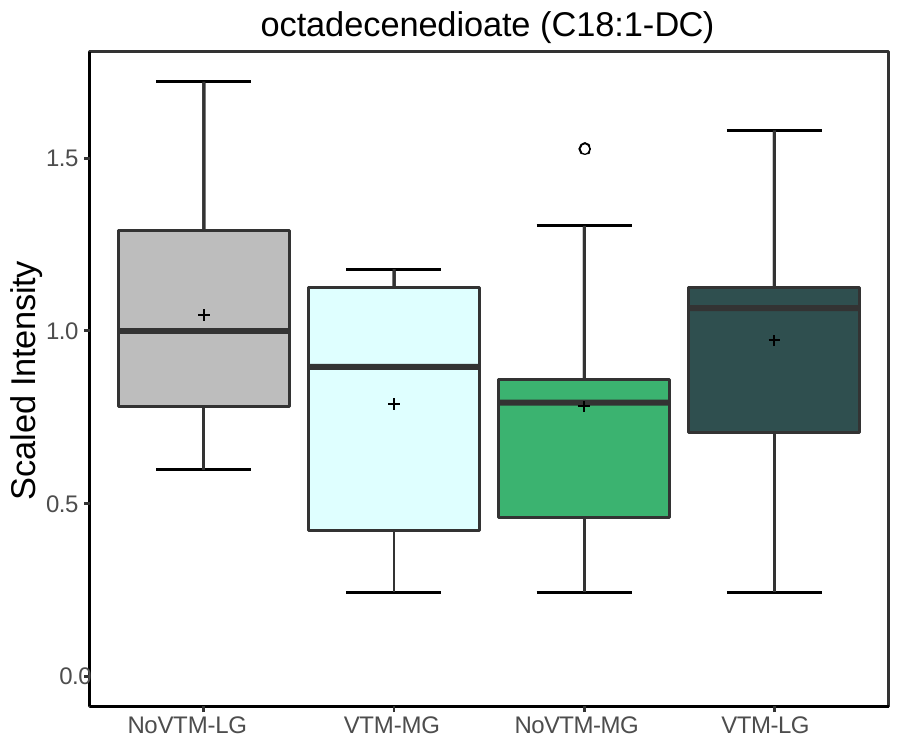

octadecenedioate (C18:1-DC)
1.5
Scaled Intensity
1.0
0.5
0.0
NoVTM-LG
VTM-MG
NoVTM-MG
VTM-LG

## Slide 66
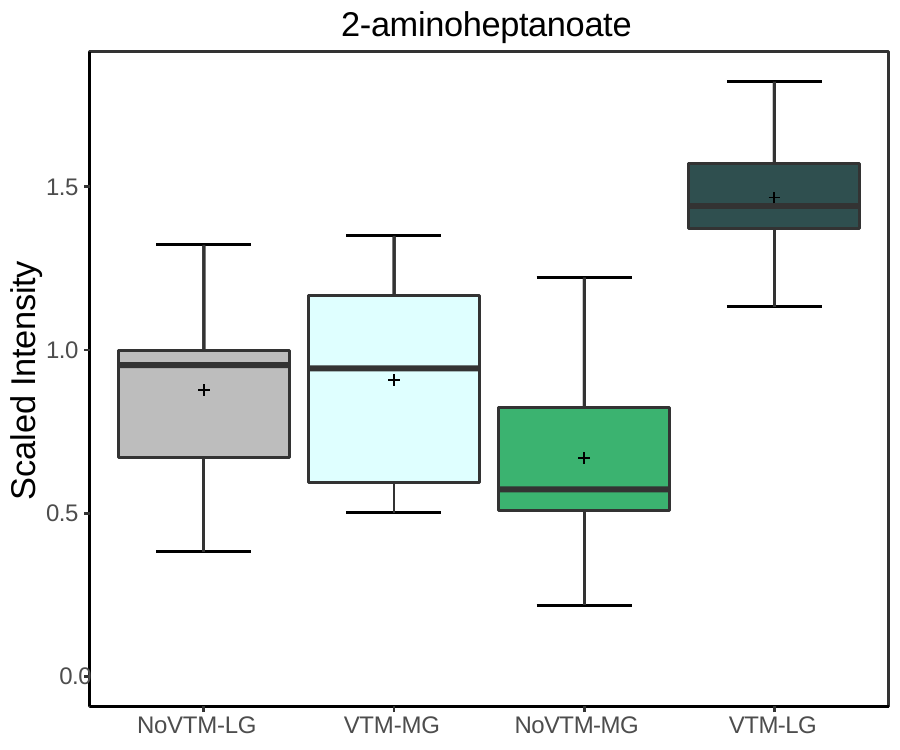

2-aminoheptanoate
1.5
Scaled Intensity
1.0
0.5
0.0
NoVTM-LG
VTM-MG
NoVTM-MG
VTM-LG

## Slide 67
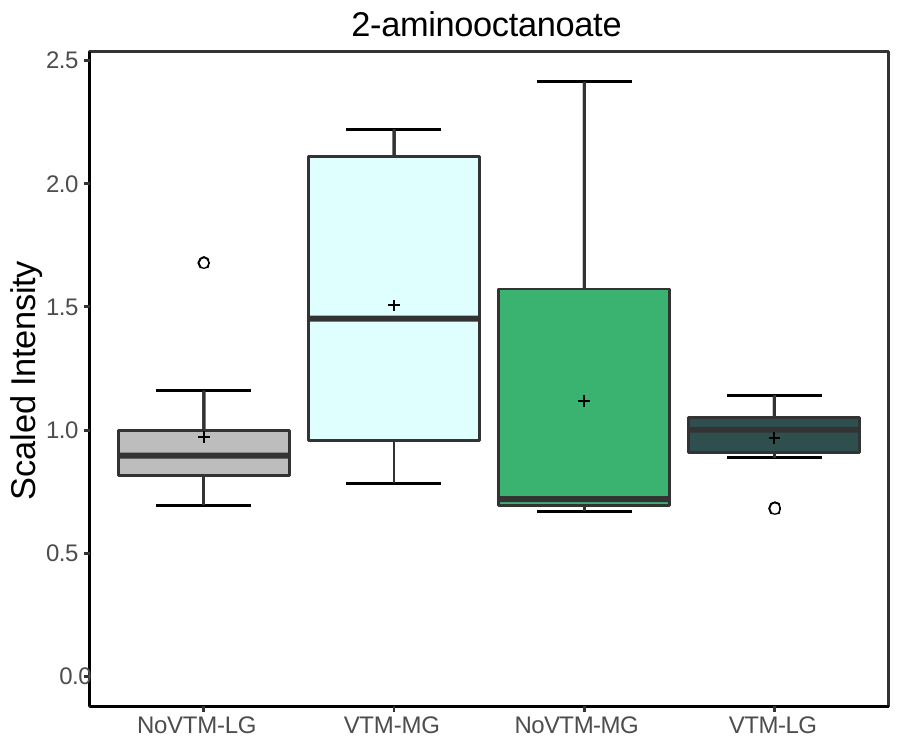

2-aminooctanoate
2.5
2.0
Scaled Intensity
1.5
1.0
0.5
0.0
NoVTM-LG
VTM-MG
NoVTM-MG
VTM-LG

## Slide 68
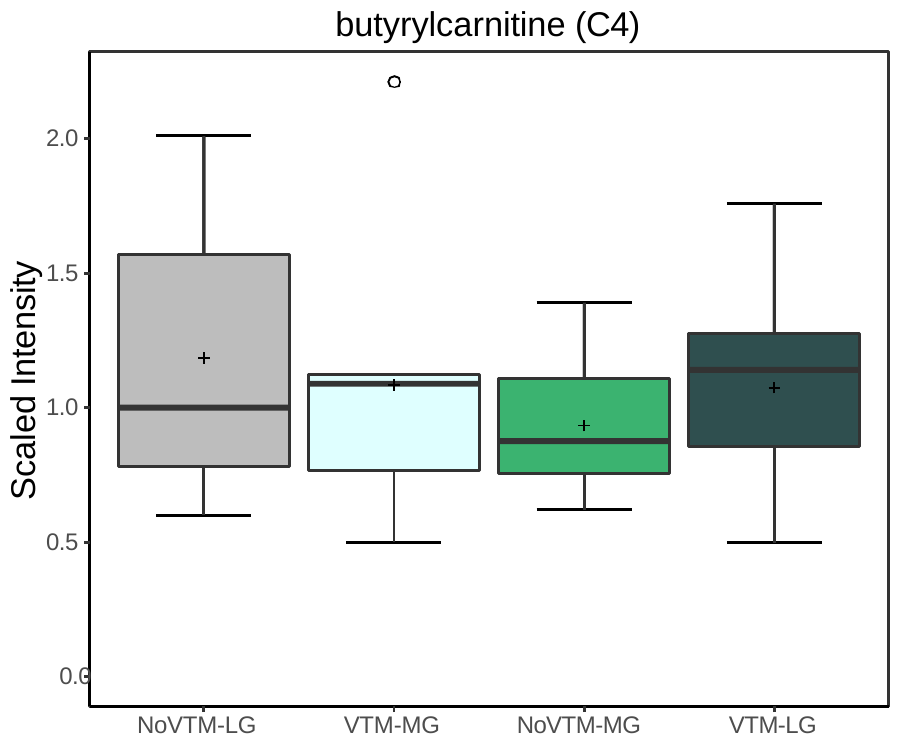

butyrylcarnitine (C4)
2.0
1.5
Scaled Intensity
1.0
0.5
0.0
NoVTM-LG
VTM-MG
NoVTM-MG
VTM-LG

## Slide 69
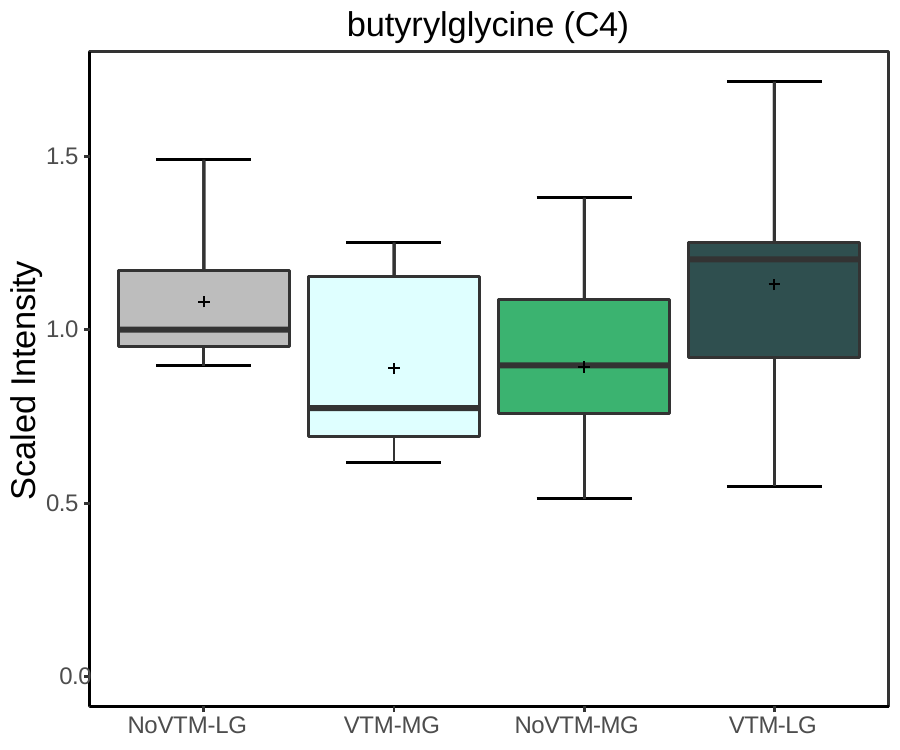

butyrylglycine (C4)
1.5
Scaled Intensity
1.0
0.5
0.0
NoVTM-LG
VTM-MG
NoVTM-MG
VTM-LG

## Slide 70
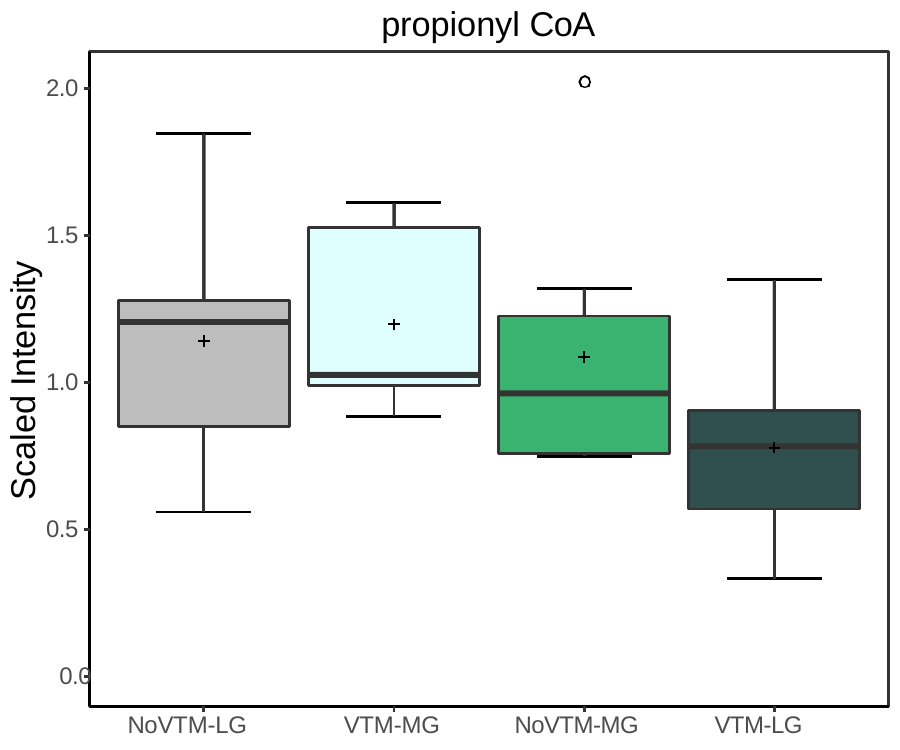

propionyl CoA
2.0
1.5
Scaled Intensity
1.0
0.5
0.0
NoVTM-LG
VTM-MG
NoVTM-MG
VTM-LG

## Slide 71
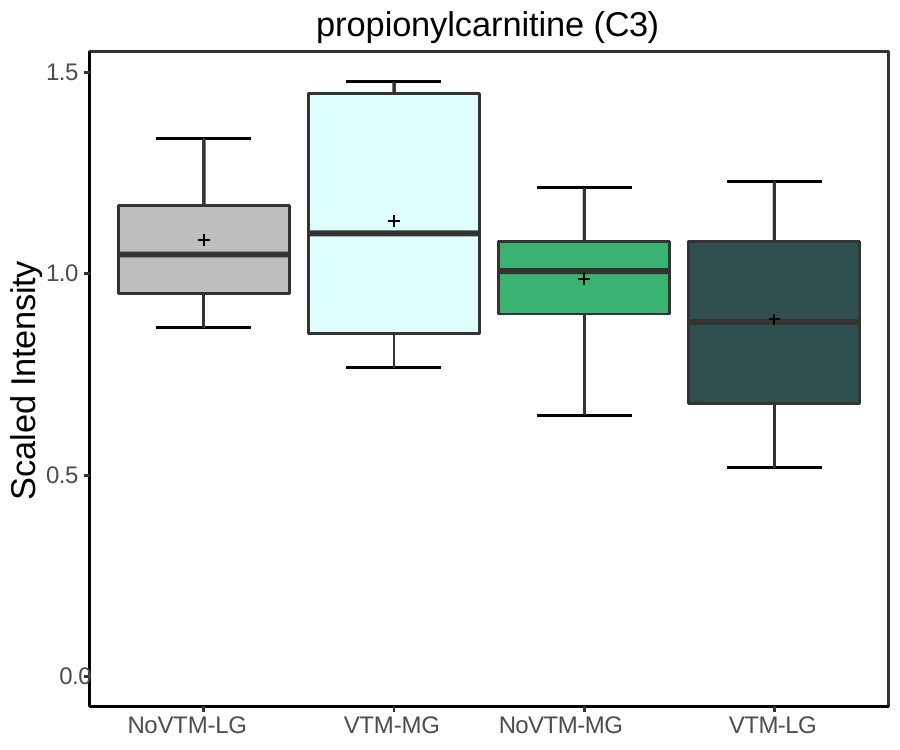

propionylcarnitine (C3)
1.5
1.0
Scaled Intensity
0.5
0.0
NoVTM-LG
VTM-MG
NoVTM-MG
VTM-LG

## Slide 72
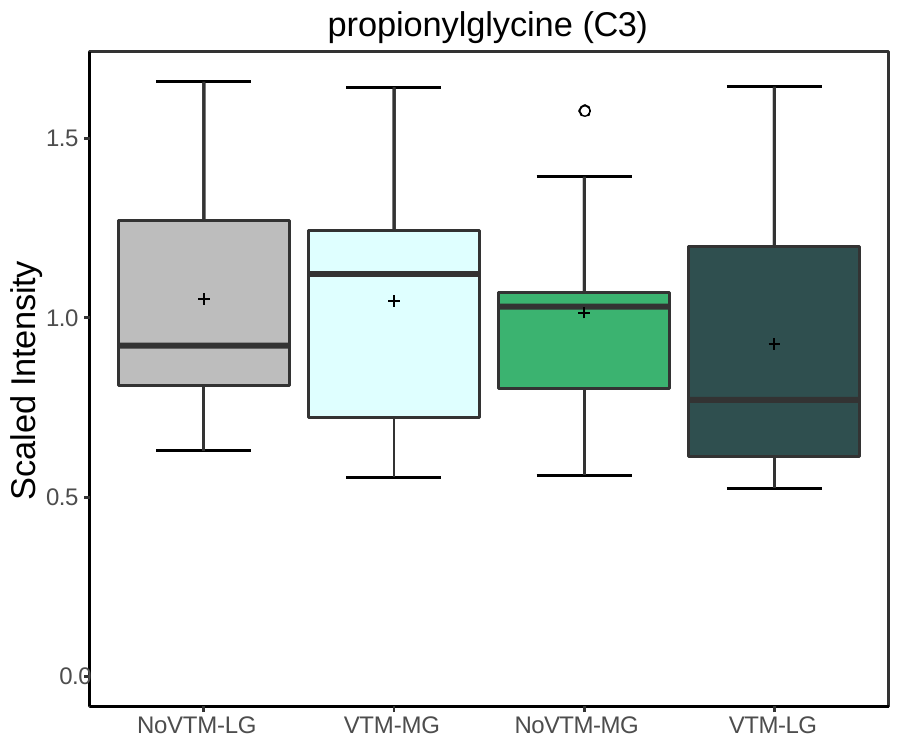

propionylglycine (C3)
1.5
Scaled Intensity
1.0
0.5
0.0
NoVTM-LG
VTM-MG
NoVTM-MG
VTM-LG

## Slide 73
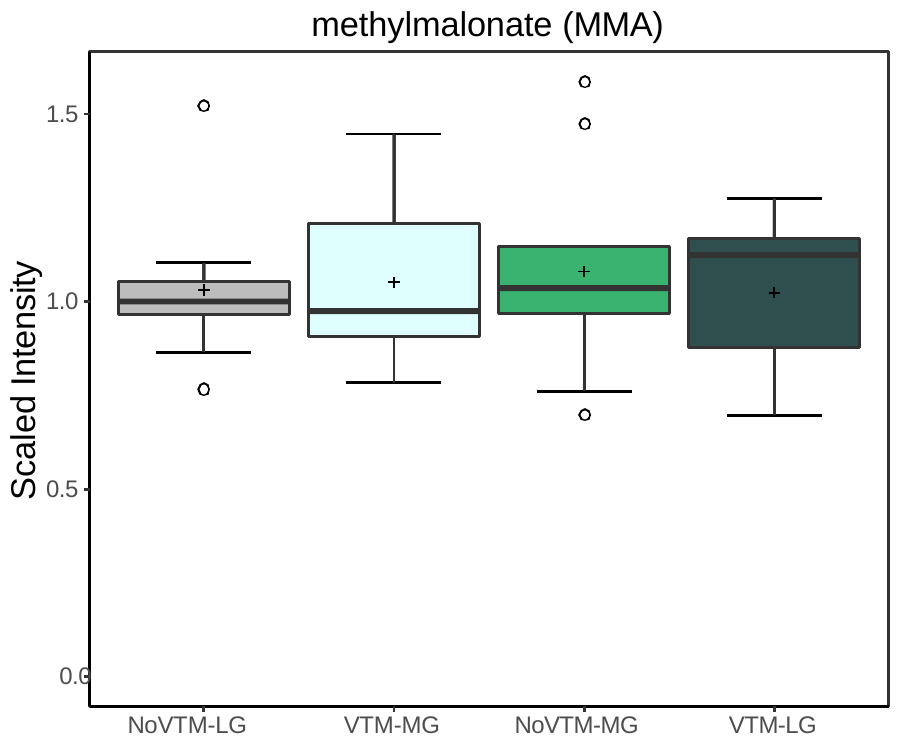

methylmalonate (MMA)
1.5
Scaled Intensity
1.0
0.5
0.0
NoVTM-LG
VTM-MG
NoVTM-MG
VTM-LG

## Slide 74
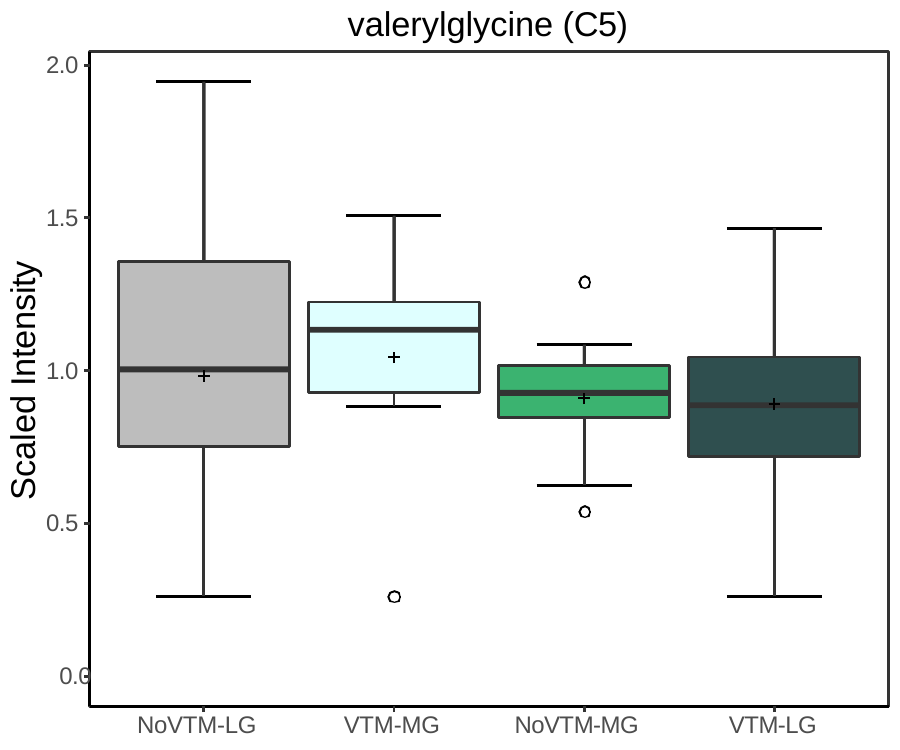

valerylglycine (C5)
2.0
1.5
Scaled Intensity
1.0
0.5
0.0
NoVTM-LG
VTM-MG
NoVTM-MG
VTM-LG

## Slide 75
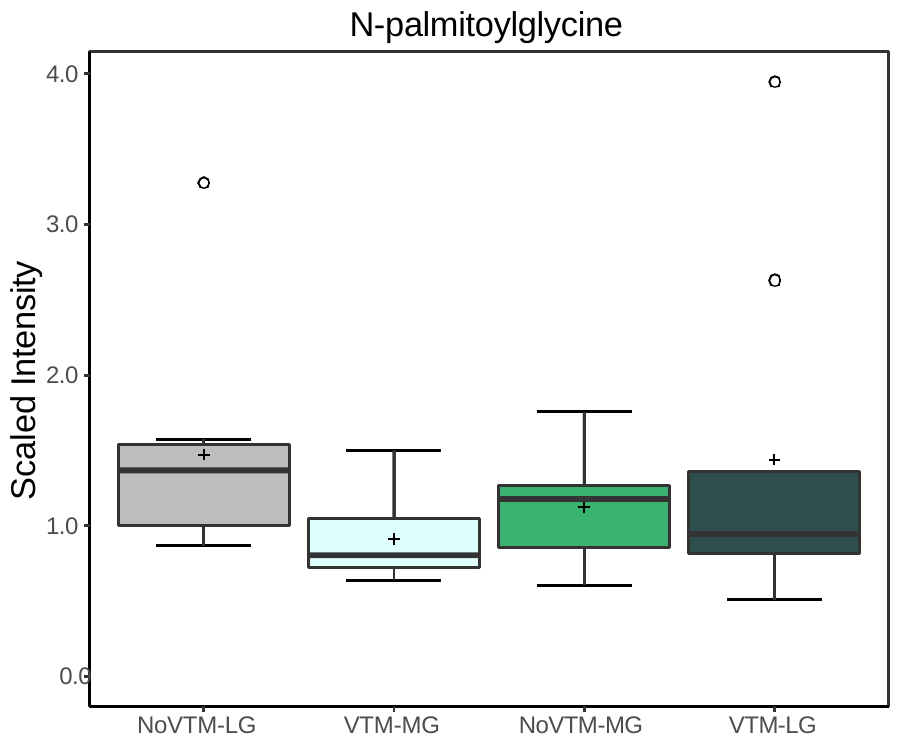

N-palmitoylglycine
4.0
3.0
Scaled Intensity
2.0
1.0
0.0
NoVTM-LG
VTM-MG
NoVTM-MG
VTM-LG

## Slide 76
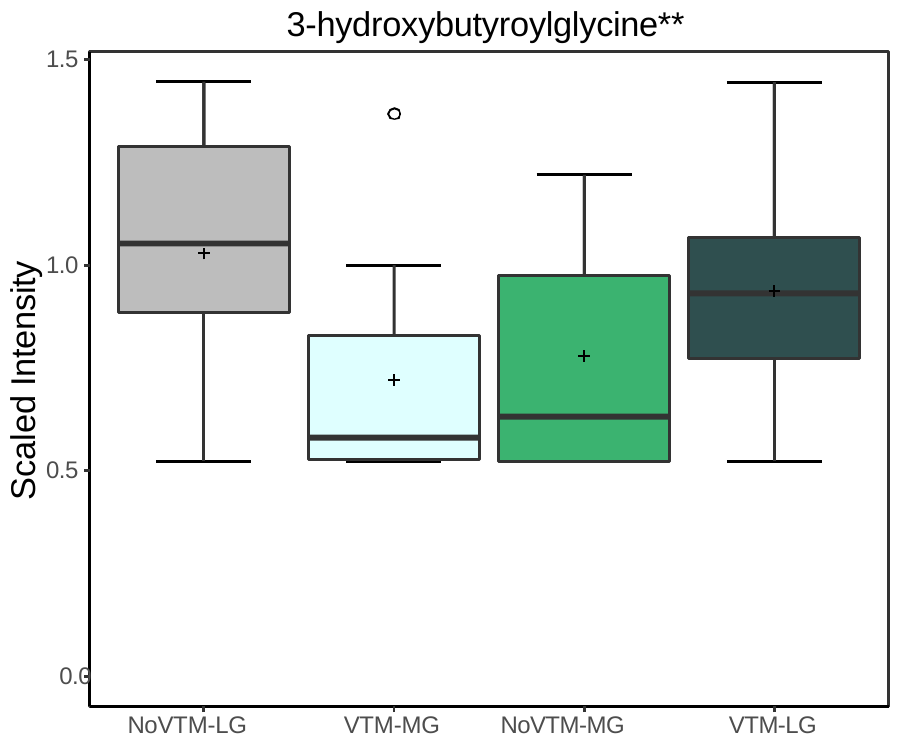

3-hydroxybutyroylglycine**
1.5
1.0
Scaled Intensity
0.5
0.0
NoVTM-LG
VTM-MG
NoVTM-MG
VTM-LG

## Slide 77
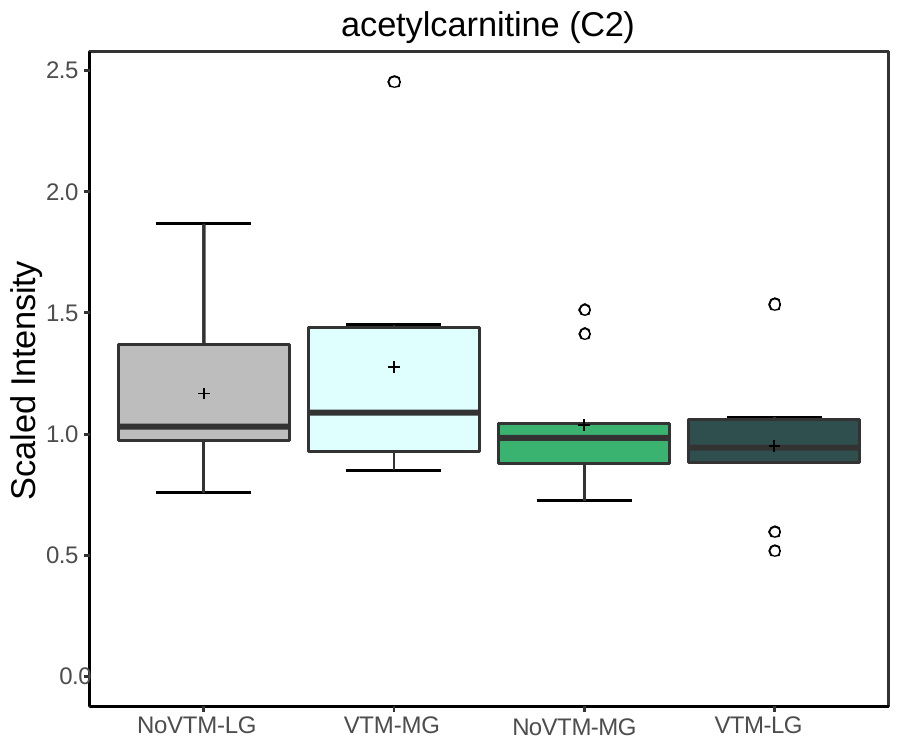

acetylcarnitine (C2)
2.5
2.0
Scaled Intensity
1.5
1.0
0.5
0.0
NoVTM-LG
VTM-MG
VTM-LG
NoVTM-MG

## Slide 78
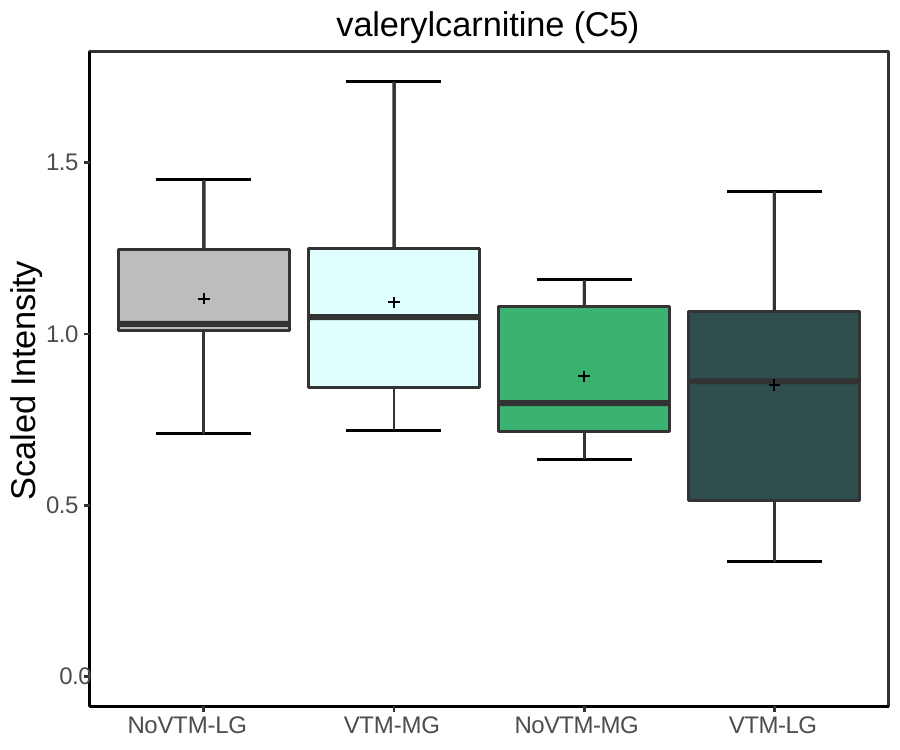

valerylcarnitine (C5)
1.5
Scaled Intensity
1.0
0.5
0.0
NoVTM-LG
VTM-MG
NoVTM-MG
VTM-LG

## Slide 79
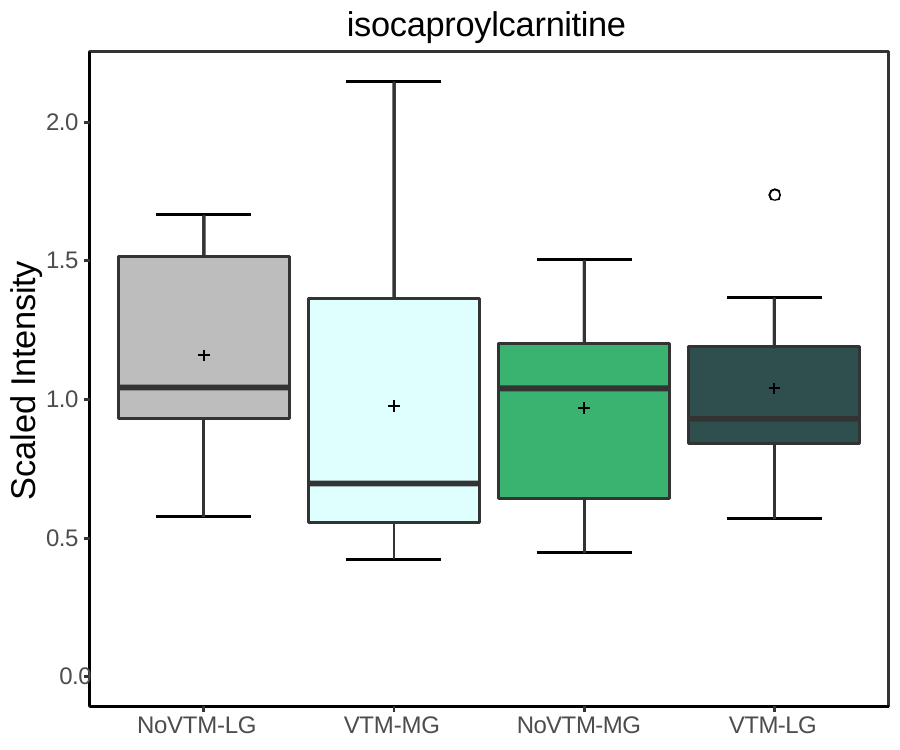

isocaproylcarnitine
2.0
1.5
Scaled Intensity
1.0
0.5
0.0
NoVTM-LG
VTM-MG
NoVTM-MG
VTM-LG

## Slide 80
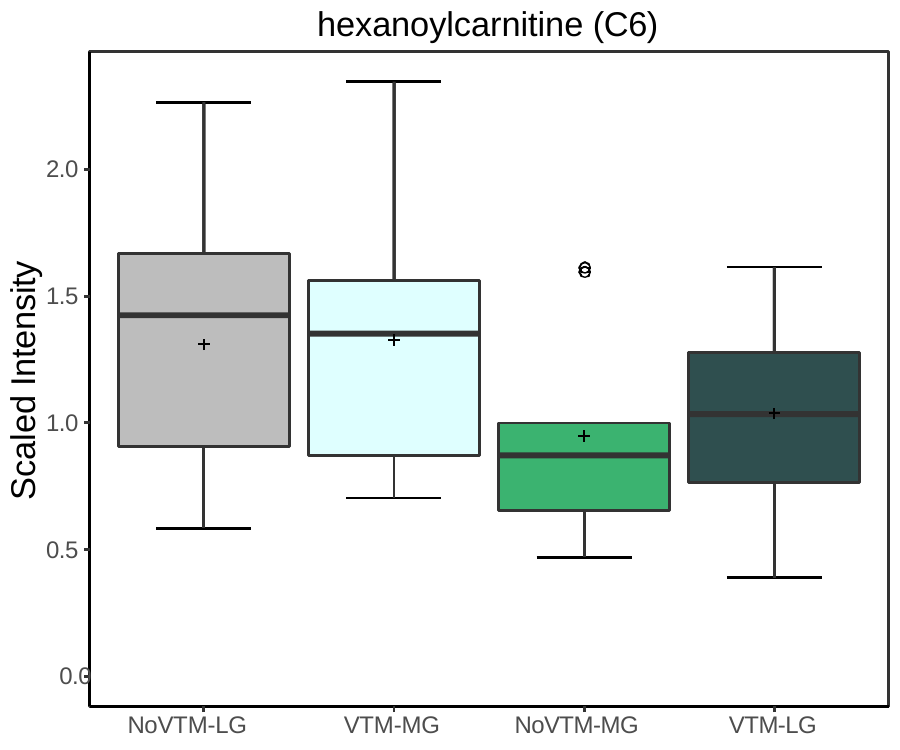

hexanoylcarnitine (C6)
2.0
Scaled Intensity
1.5
1.0
0.5
0.0
NoVTM-LG
VTM-MG
NoVTM-MG
VTM-LG

## Slide 81
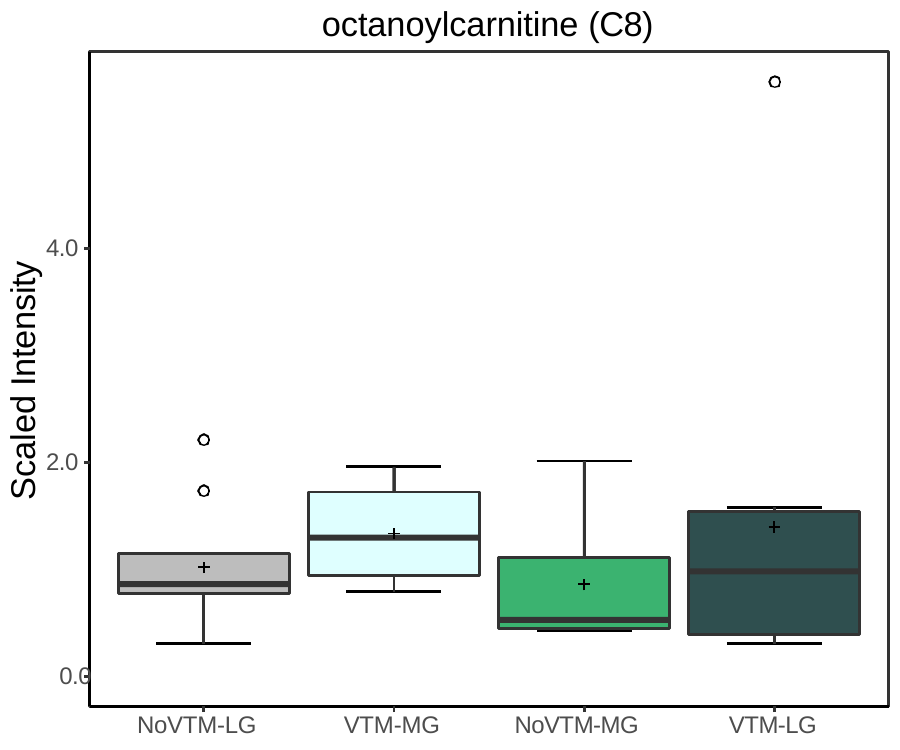

octanoylcarnitine (C8)
4.0
Scaled Intensity
2.0
0.0
NoVTM-LG
VTM-MG
NoVTM-MG
VTM-LG

## Slide 82
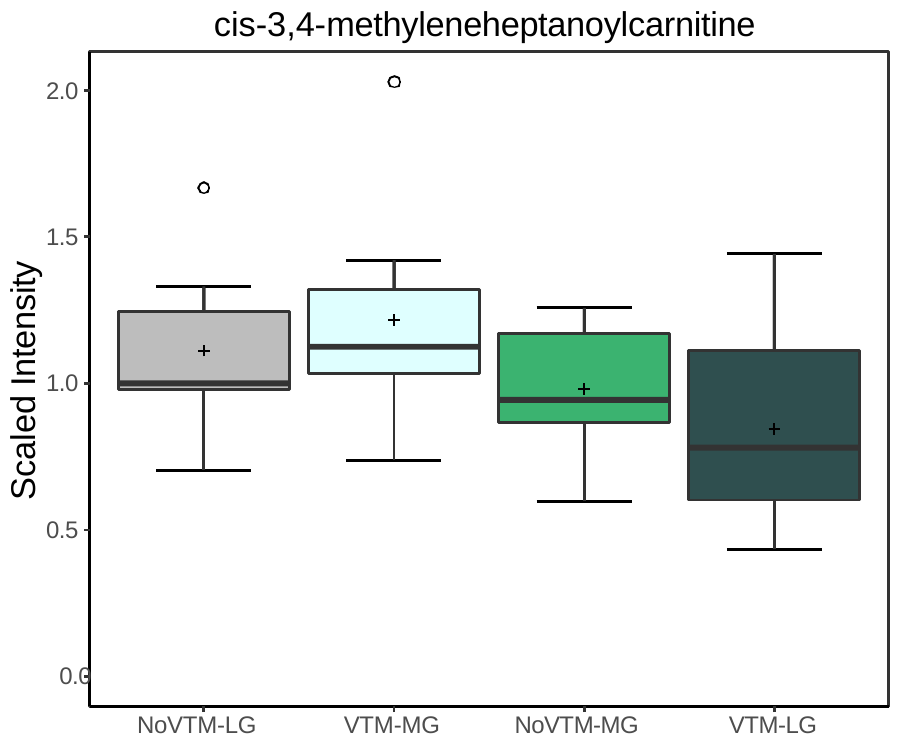

cis-3,4-methyleneheptanoylcarnitine
2.0
1.5
Scaled Intensity
1.0
0.5
0.0
NoVTM-LG
VTM-MG
NoVTM-MG
VTM-LG

## Slide 83
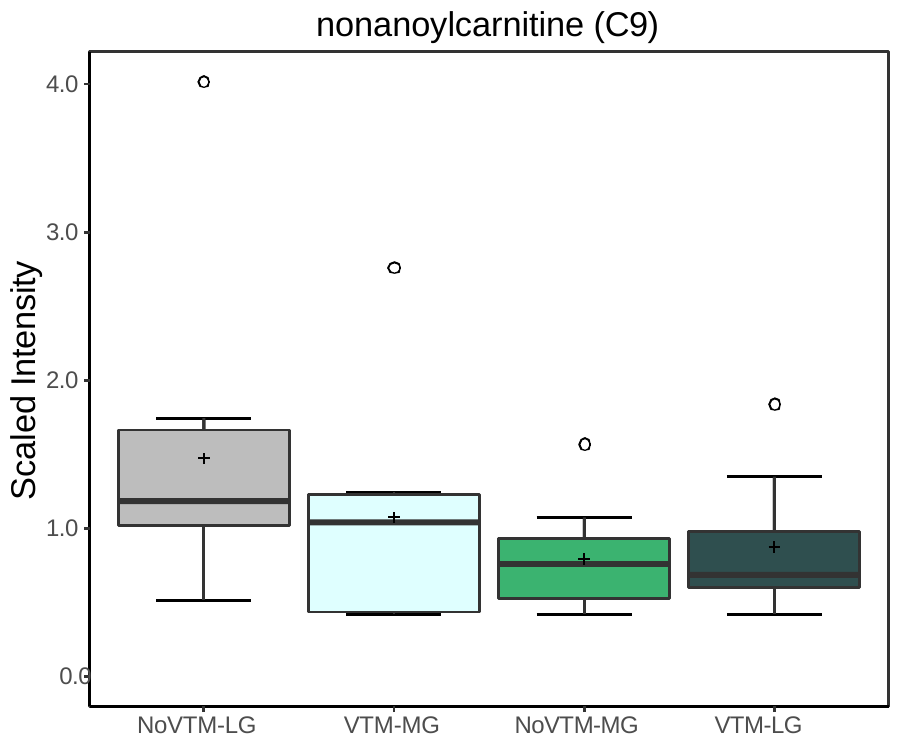

nonanoylcarnitine (C9)
4.0
3.0
Scaled Intensity
2.0
1.0
0.0
NoVTM-LG
VTM-MG
NoVTM-MG
VTM-LG

## Slide 84
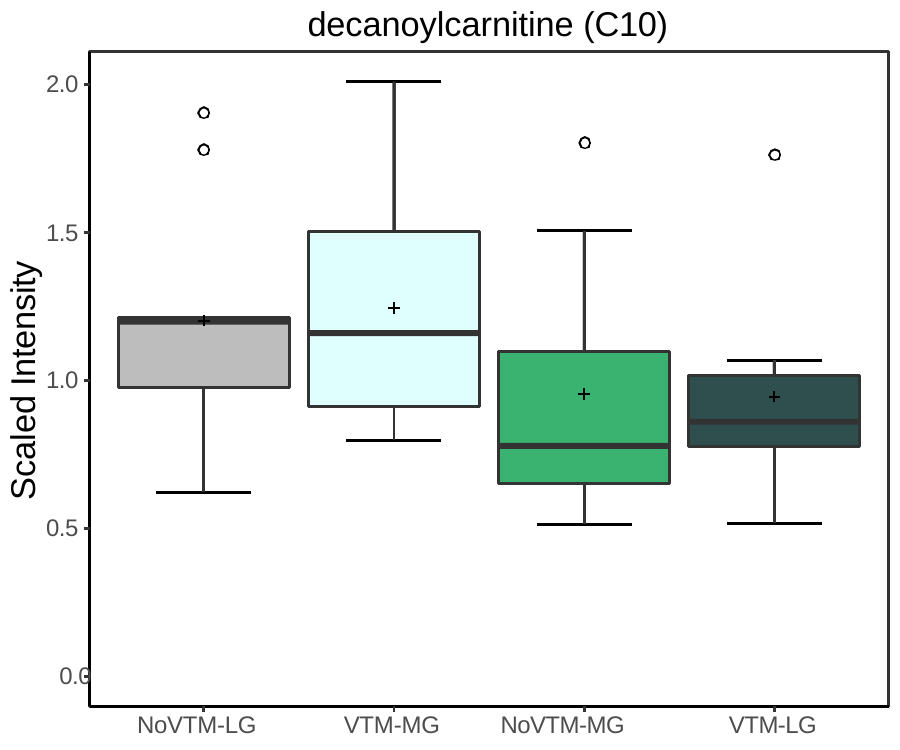

decanoylcarnitine (C10)
2.0
1.5
Scaled Intensity
1.0
0.5
0.0
NoVTM-LG
VTM-MG
NoVTM-MG
VTM-LG

## Slide 85
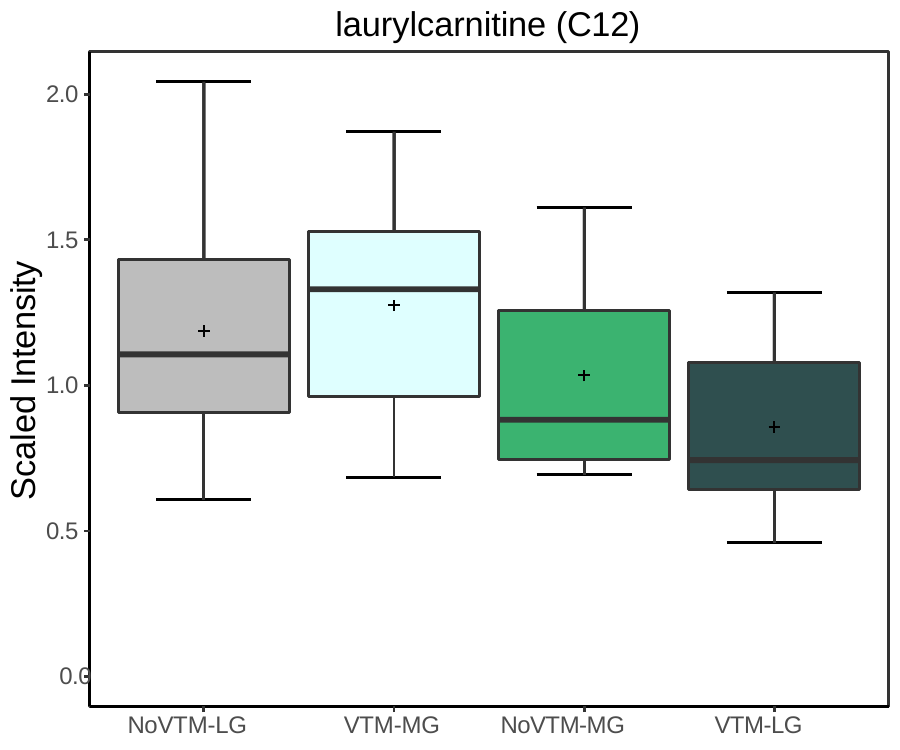

laurylcarnitine (C12)
2.0
1.5
Scaled Intensity
1.0
0.5
0.0
NoVTM-LG
VTM-MG
NoVTM-MG
VTM-LG

## Slide 86
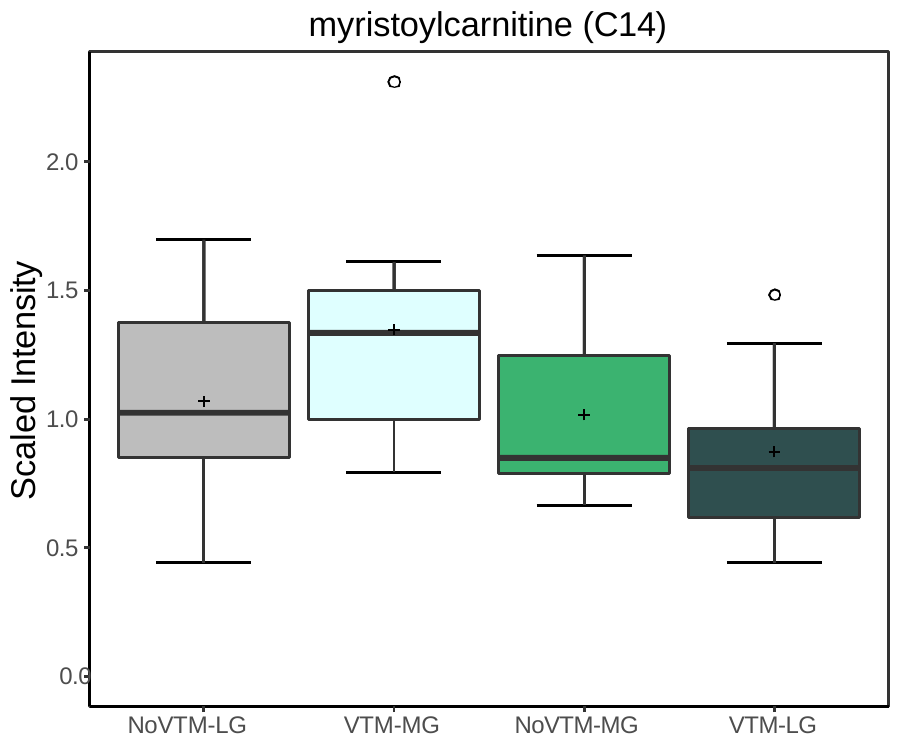

myristoylcarnitine (C14)
2.0
Scaled Intensity
1.5
1.0
0.5
0.0
NoVTM-LG
VTM-MG
NoVTM-MG
VTM-LG

## Slide 87
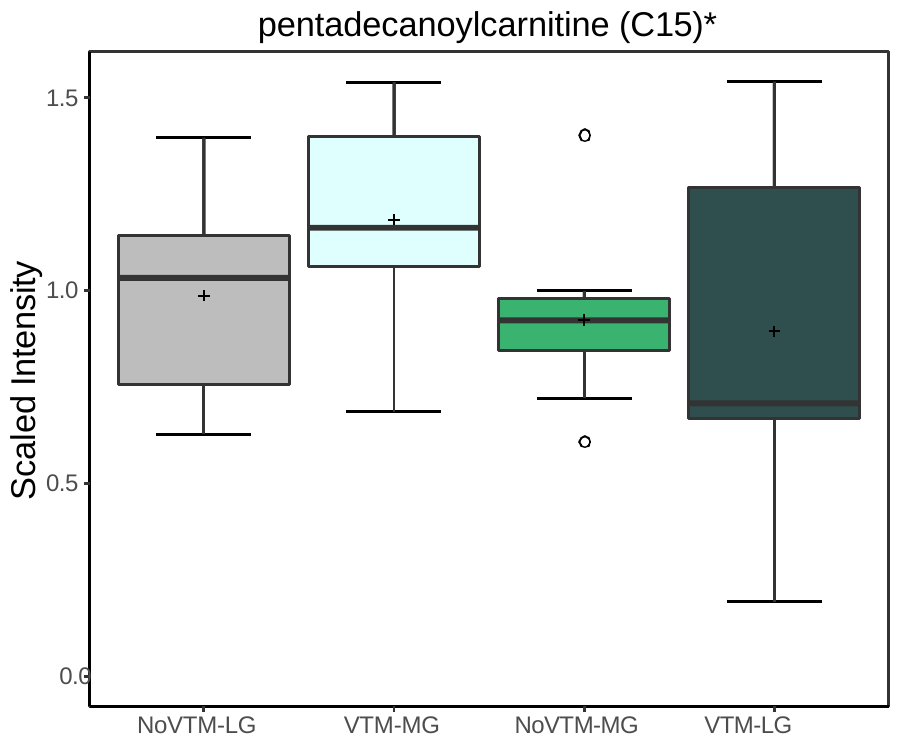

pentadecanoylcarnitine (C15)*
1.5
Scaled Intensity
1.0
0.5
0.0
NoVTM-LG
VTM-MG
NoVTM-MG
VTM-LG

## Slide 88
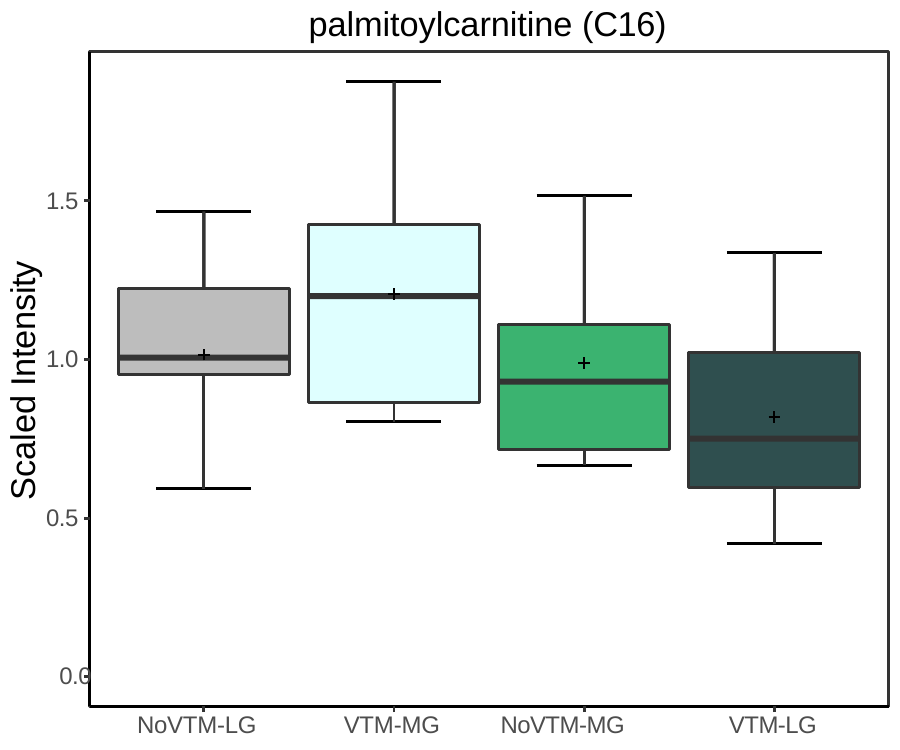

palmitoylcarnitine (C16)
1.5
Scaled Intensity
1.0
0.5
0.0
NoVTM-LG
VTM-MG
NoVTM-MG
VTM-LG

## Slide 89
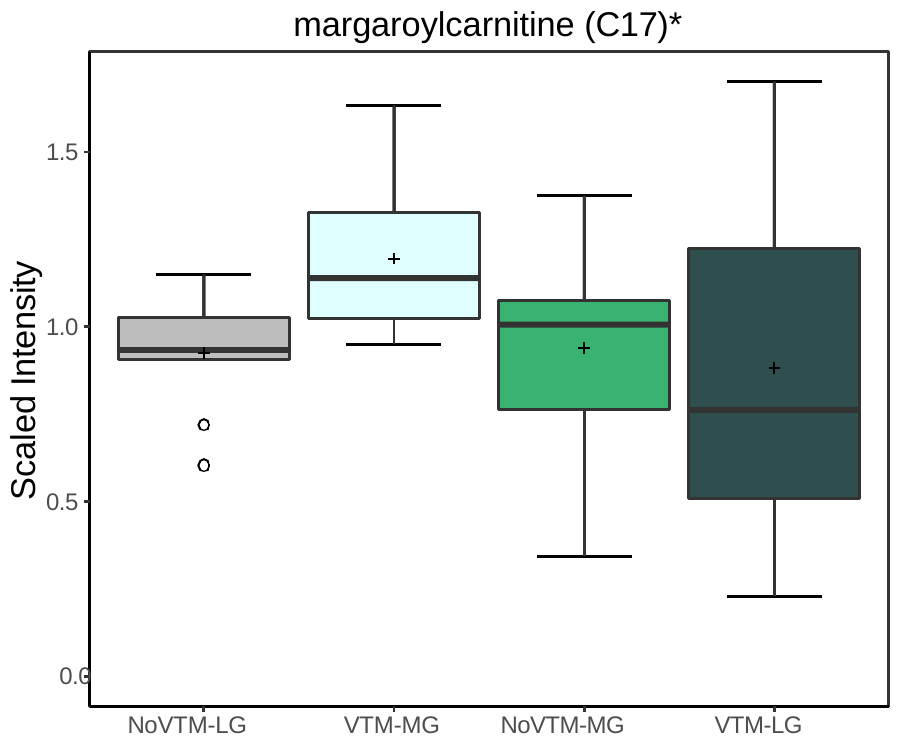

margaroylcarnitine (C17)*
1.5
Scaled Intensity
1.0
0.5
0.0
NoVTM-LG
VTM-MG
NoVTM-MG
VTM-LG

## Slide 90
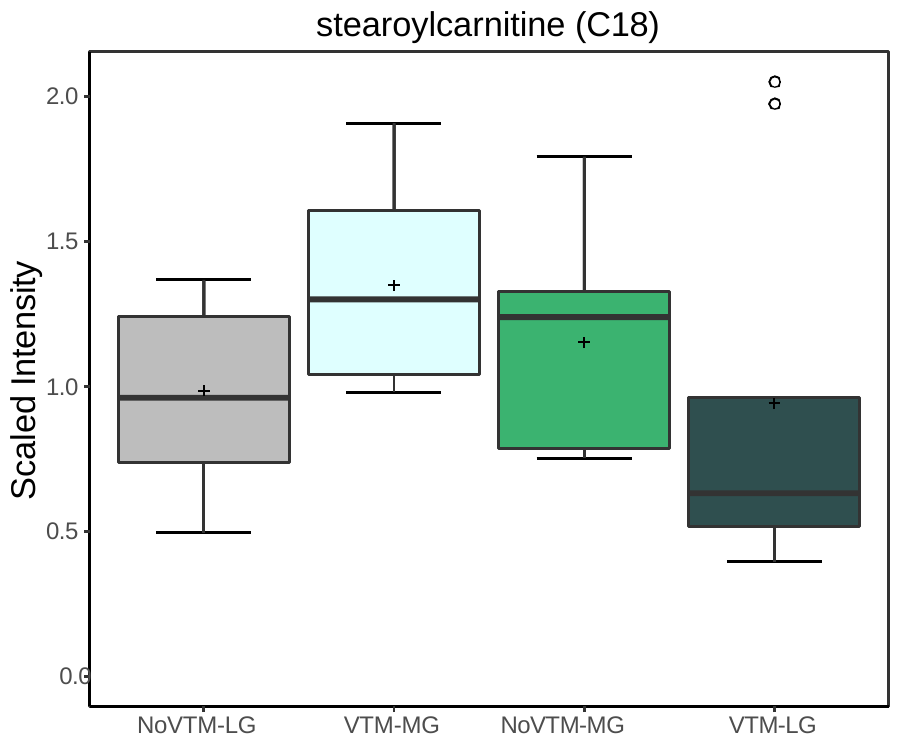

stearoylcarnitine (C18)
2.0
1.5
Scaled Intensity
1.0
0.5
0.0
NoVTM-LG
VTM-MG
NoVTM-MG
VTM-LG

## Slide 91
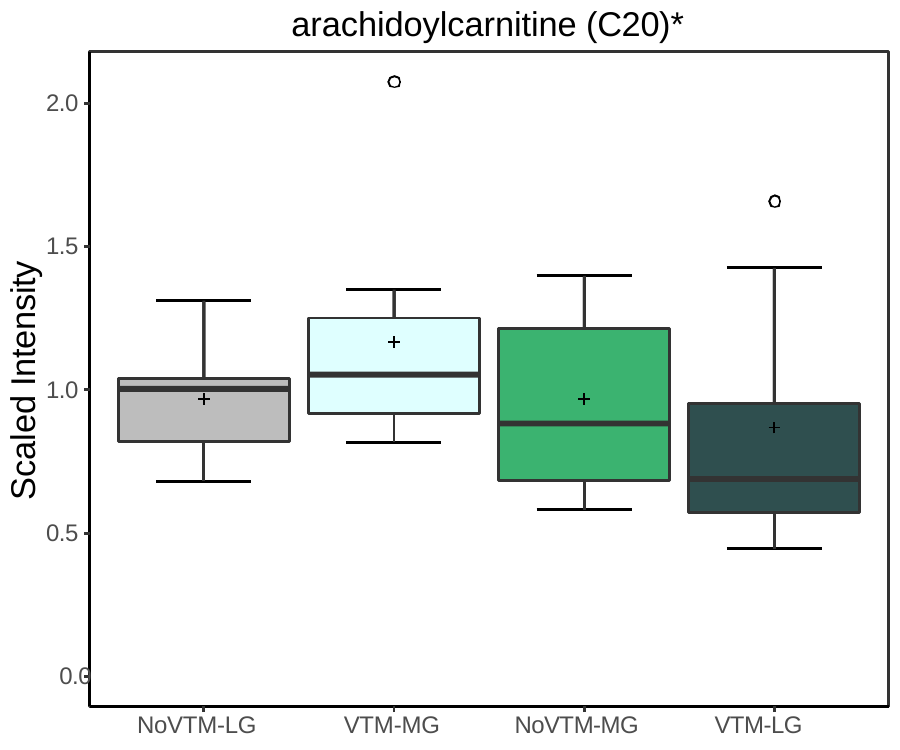

arachidoylcarnitine (C20)*
2.0
1.5
Scaled Intensity
1.0
0.5
0.0
NoVTM-LG
VTM-MG
NoVTM-MG
VTM-LG

## Slide 92
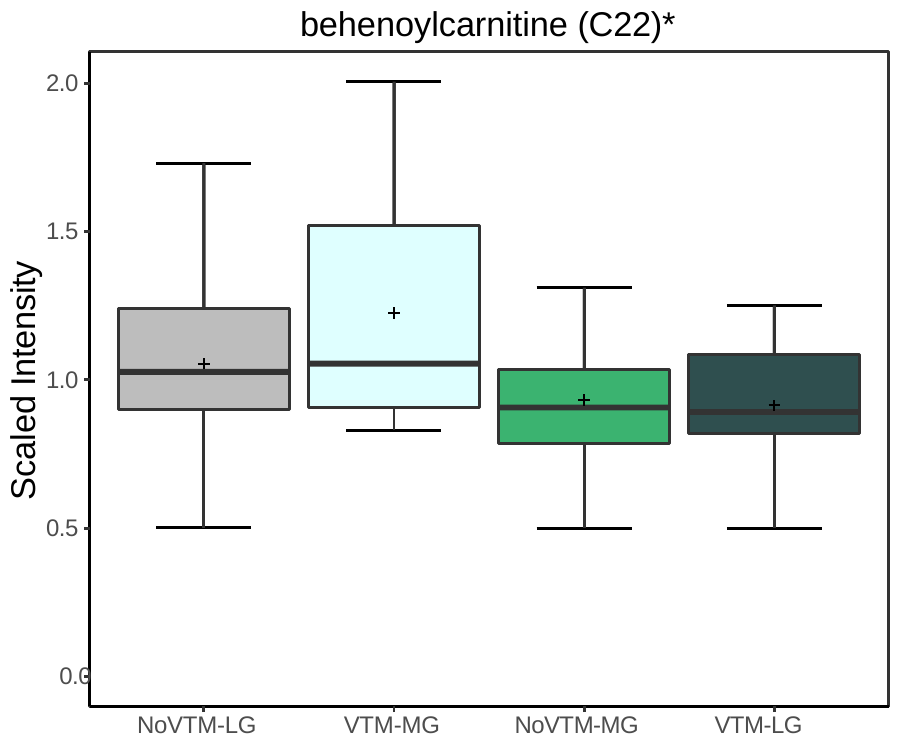

behenoylcarnitine (C22)*
2.0
1.5
Scaled Intensity
1.0
0.5
0.0
NoVTM-LG
VTM-MG
NoVTM-MG
VTM-LG

## Slide 93
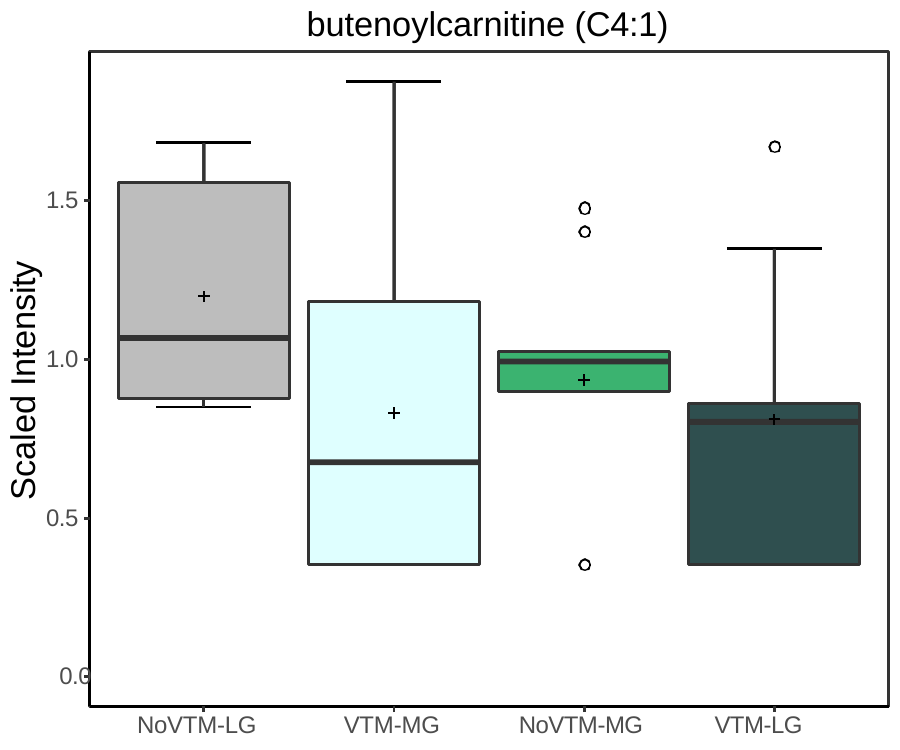

butenoylcarnitine (C4:1)
1.5
Scaled Intensity
1.0
0.5
0.0
NoVTM-LG
VTM-MG
NoVTM-MG
VTM-LG

## Slide 94
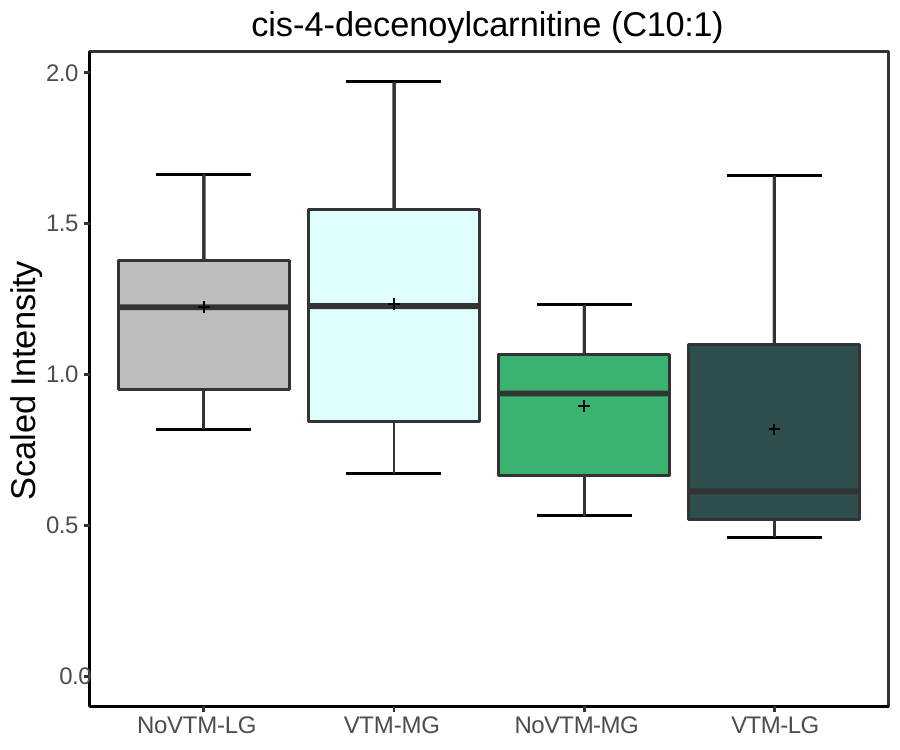

cis-4-decenoylcarnitine (C10:1)
2.0
1.5
Scaled Intensity
1.0
0.5
0.0
NoVTM-LG
VTM-MG
NoVTM-MG
VTM-LG

## Slide 95
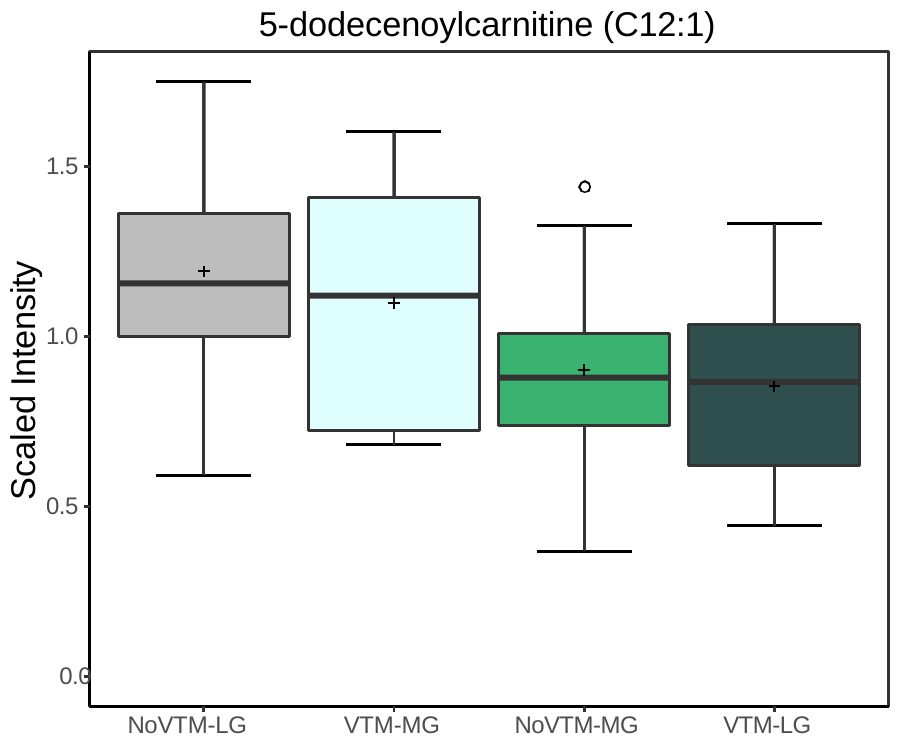

5-dodecenoylcarnitine (C12:1)
1.5
Scaled Intensity
1.0
0.5
0.0
NoVTM-LG
VTM-MG
NoVTM-MG
VTM-LG

## Slide 96
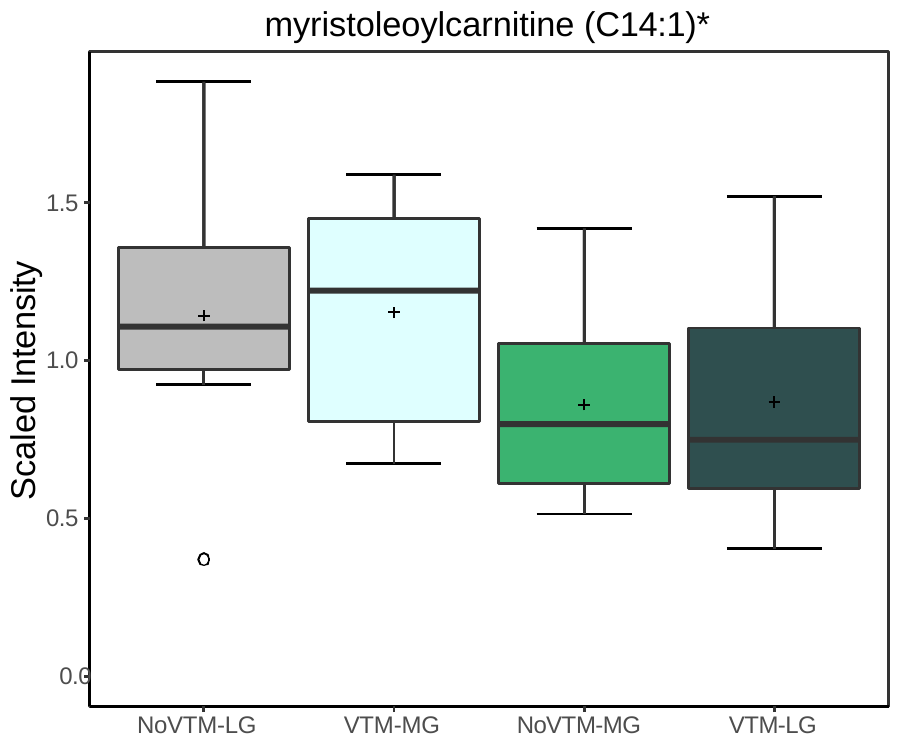

myristoleoylcarnitine (C14:1)*
1.5
Scaled Intensity
1.0
0.5
0.0
NoVTM-LG
VTM-MG
NoVTM-MG
VTM-LG

## Slide 97
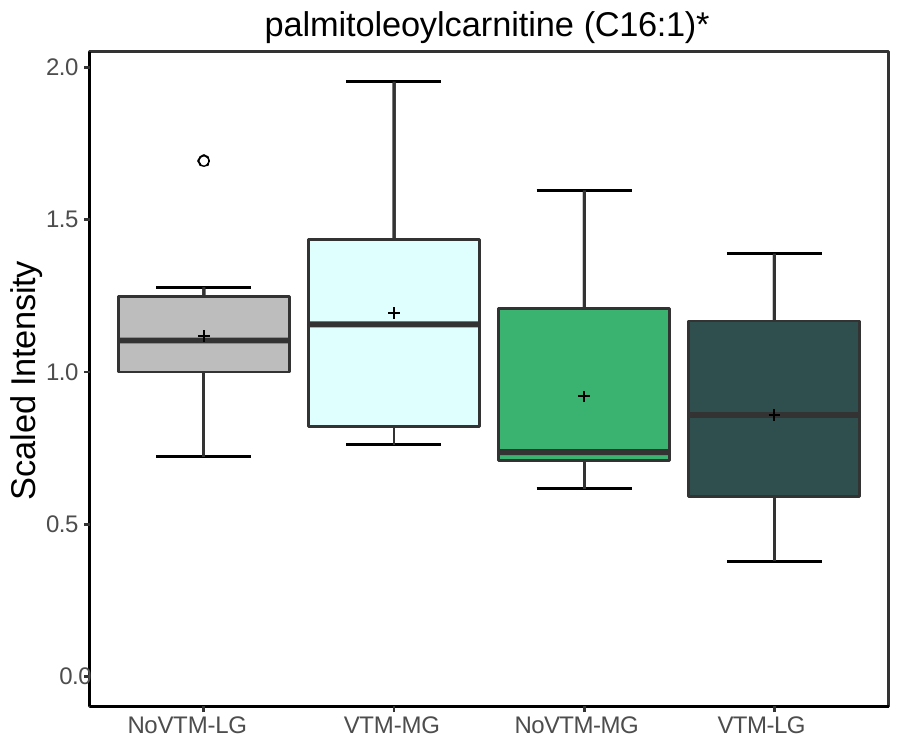

palmitoleoylcarnitine (C16:1)*
2.0
1.5
Scaled Intensity
1.0
0.5
0.0
NoVTM-LG
VTM-MG
NoVTM-MG
VTM-LG

## Slide 98
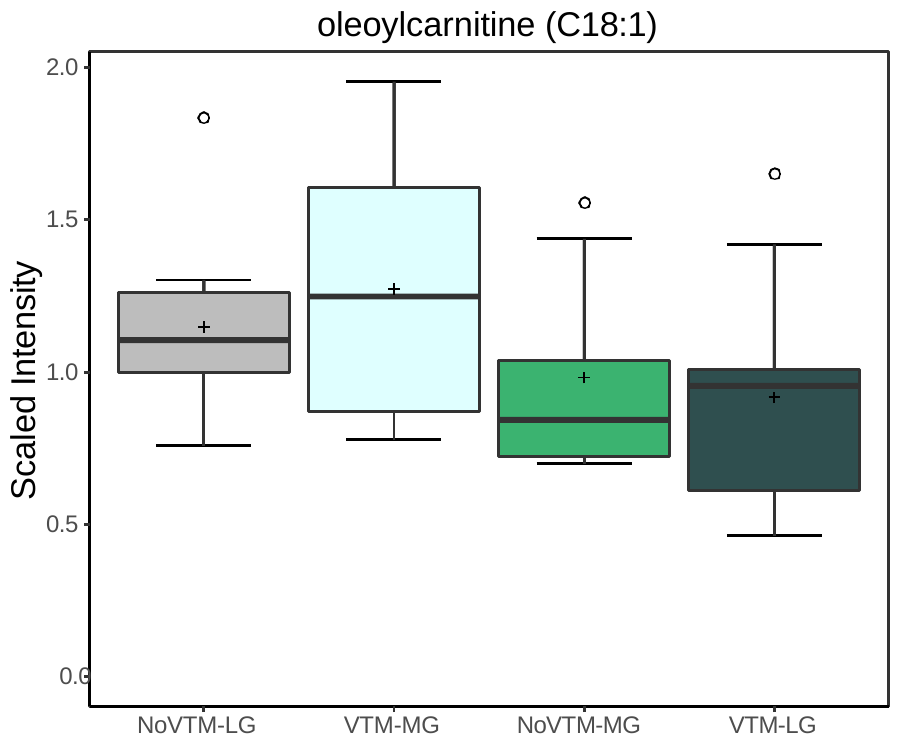

oleoylcarnitine (C18:1)
2.0
1.5
Scaled Intensity
1.0
0.5
0.0
NoVTM-LG
VTM-MG
NoVTM-MG
VTM-LG

## Slide 99
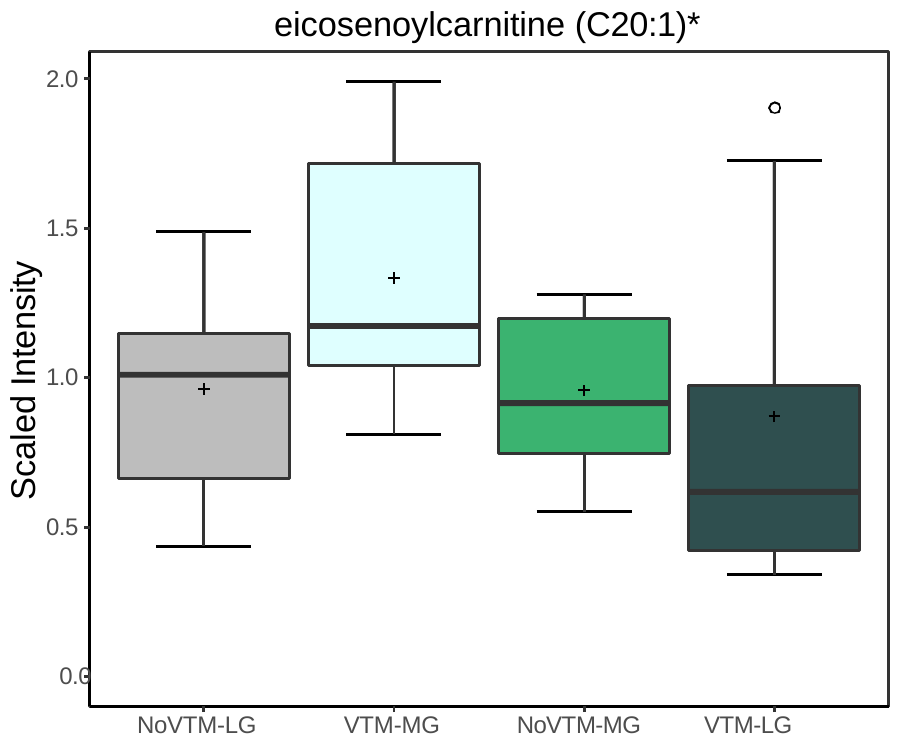

eicosenoylcarnitine (C20:1)*
2.0
1.5
Scaled Intensity
1.0
0.5
0.0
NoVTM-LG
VTM-MG
NoVTM-MG
VTM-LG

## Slide 100
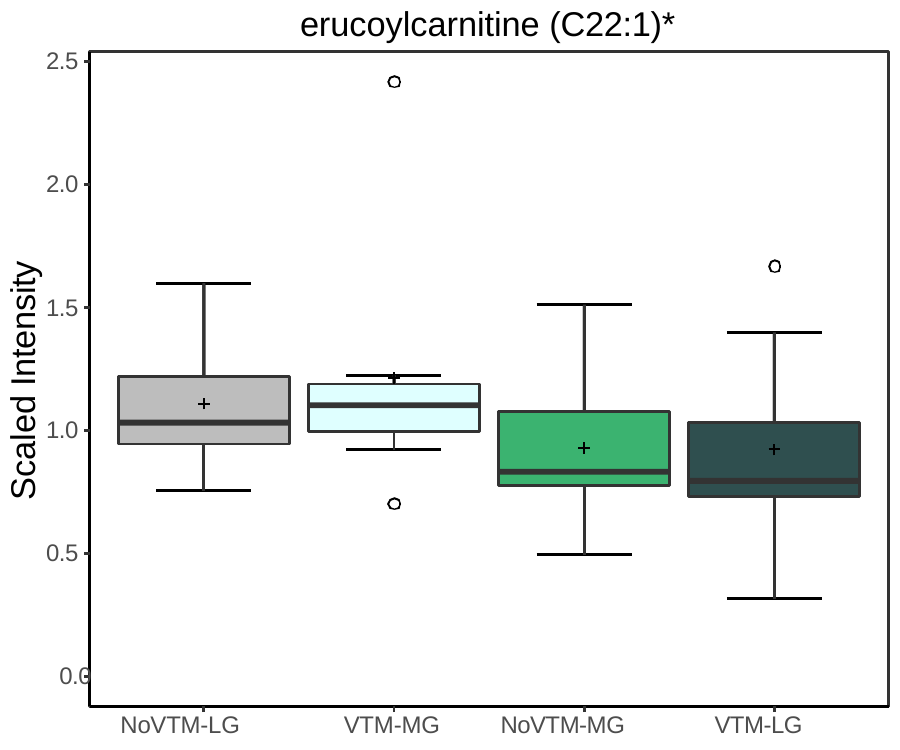

erucoylcarnitine (C22:1)*
2.5
2.0
Scaled Intensity
1.5
1.0
0.5
0.0
NoVTM-LG
VTM-MG
NoVTM-MG
VTM-LG

## Slide 101
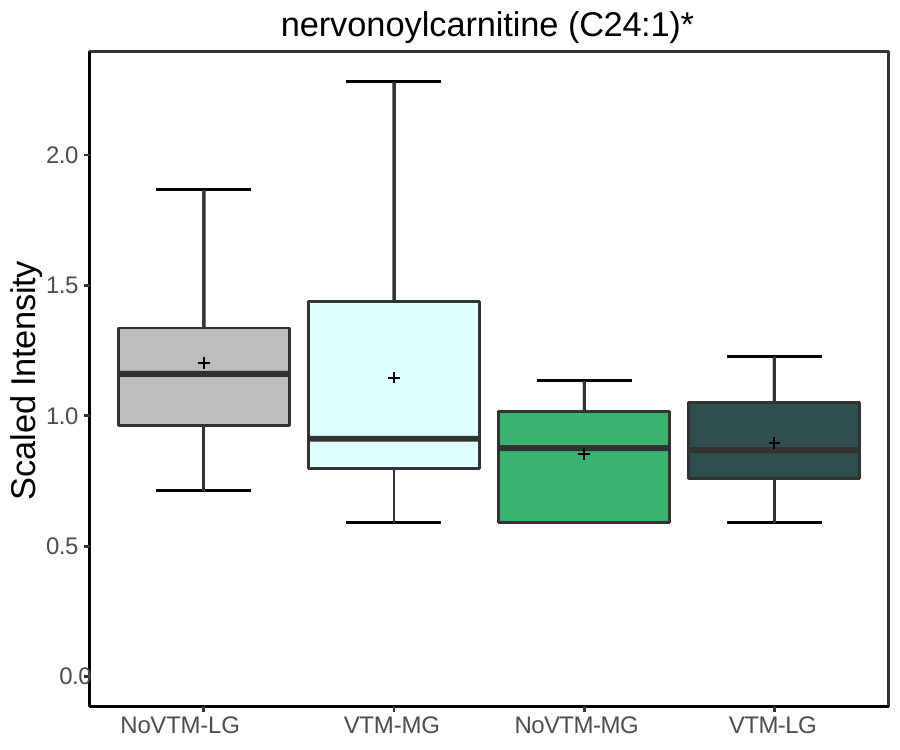

nervonoylcarnitine (C24:1)*
2.0
Scaled Intensity
1.5
1.0
0.5
0.0
NoVTM-LG
VTM-MG
NoVTM-MG
VTM-LG

## Slide 102
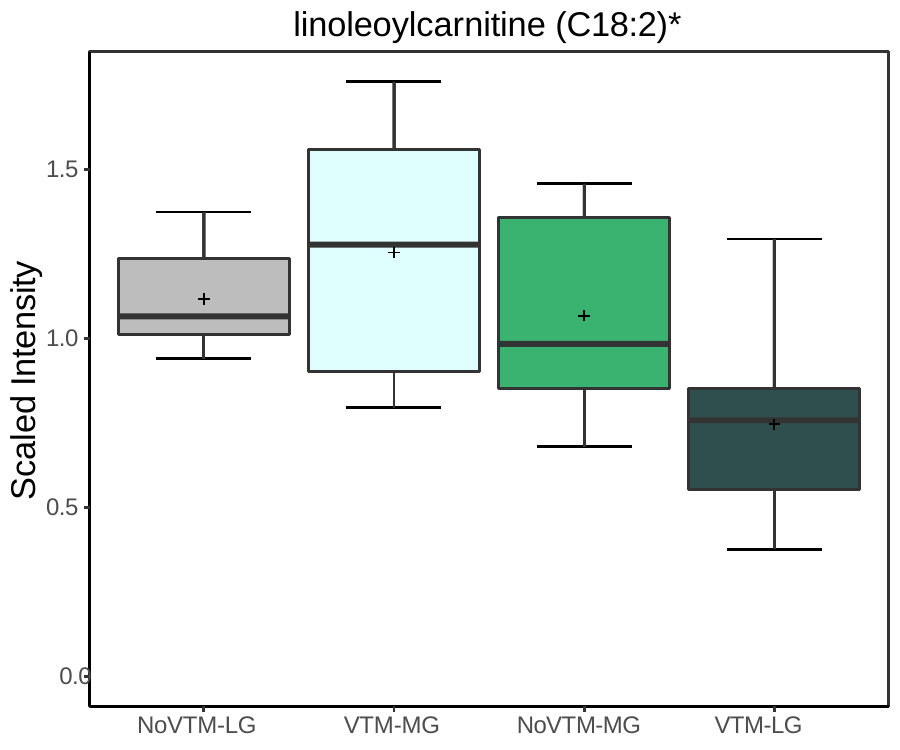

linoleoylcarnitine (C18:2)*
1.5
Scaled Intensity
1.0
0.5
0.0
NoVTM-LG
VTM-MG
NoVTM-MG
VTM-LG

## Slide 103
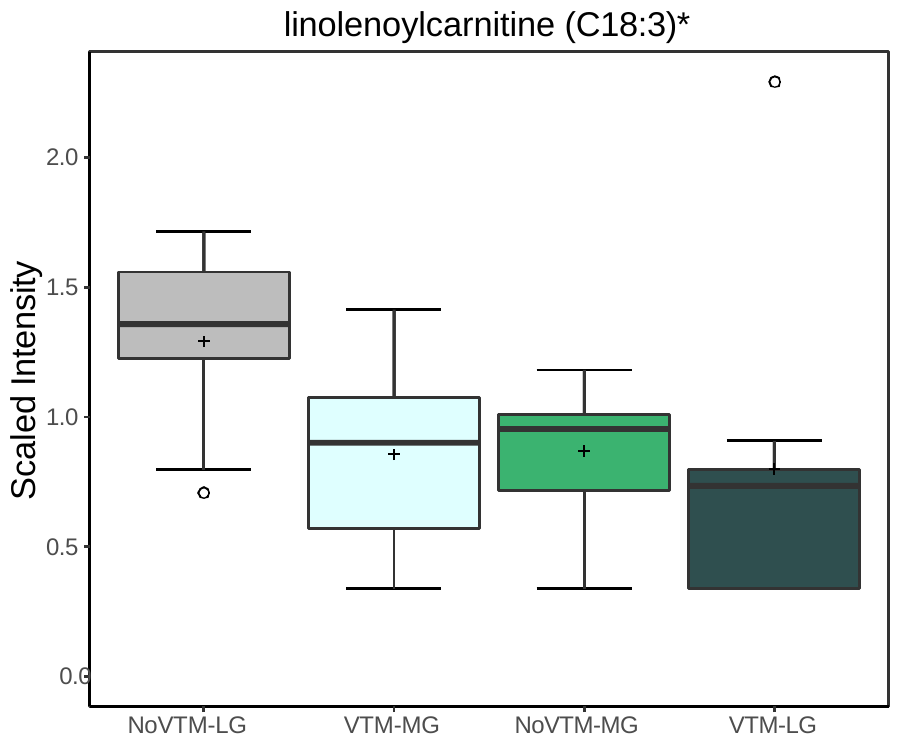

linolenoylcarnitine (C18:3)*
2.0
Scaled Intensity
1.5
1.0
0.5
0.0
NoVTM-LG
VTM-MG
NoVTM-MG
VTM-LG

## Slide 104
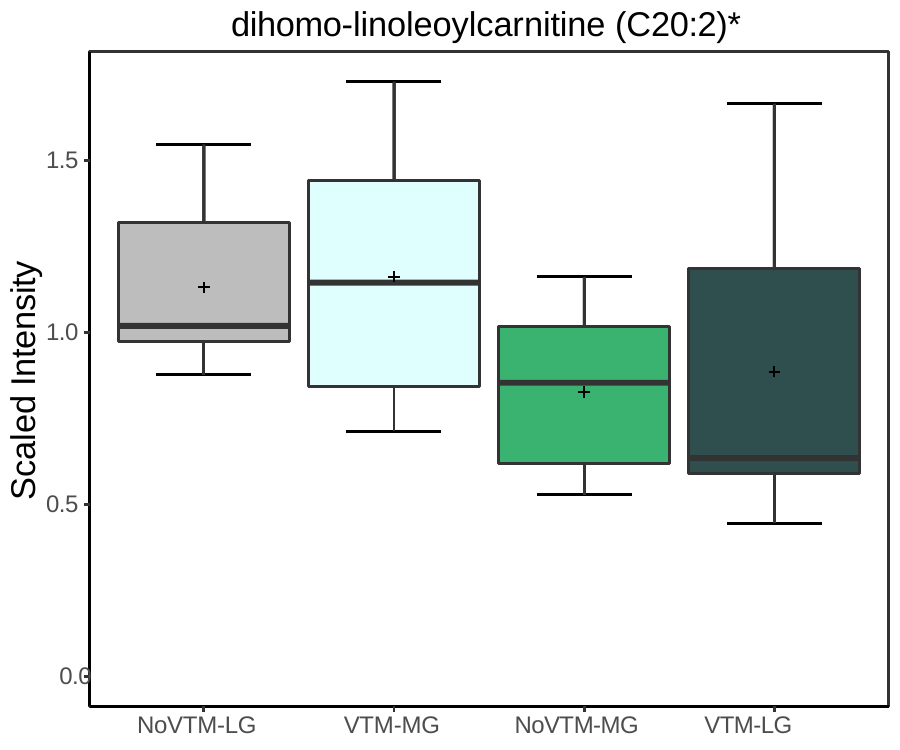

dihomo-linoleoylcarnitine (C20:2)*
1.5
Scaled Intensity
1.0
0.5
0.0
NoVTM-LG
VTM-MG
NoVTM-MG
VTM-LG

## Slide 105
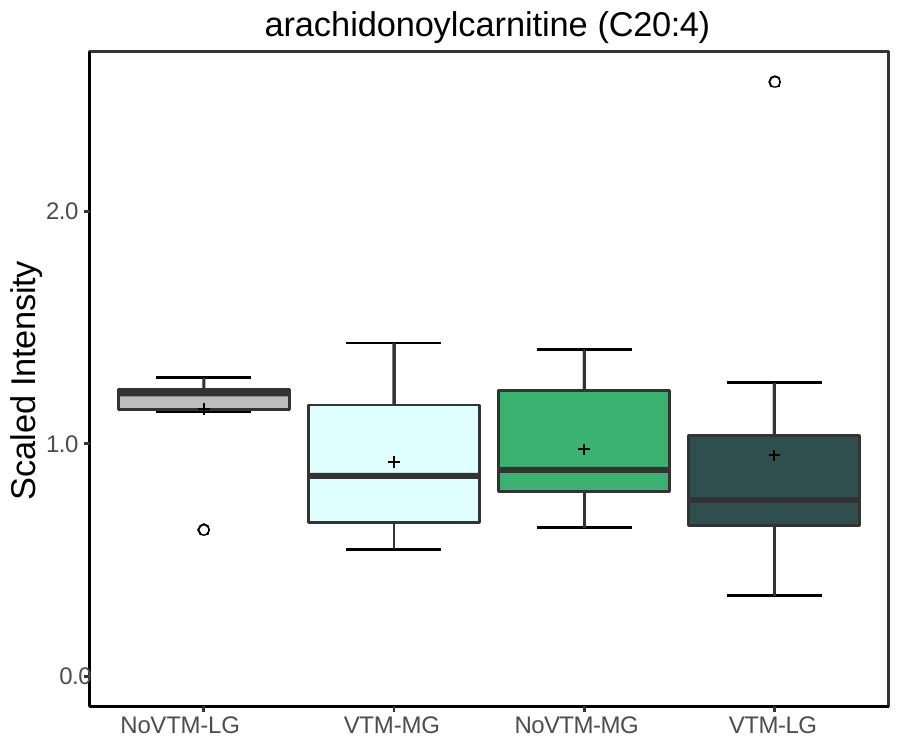

arachidonoylcarnitine (C20:4)
2.0
Scaled Intensity
1.0
0.0
NoVTM-LG
VTM-MG
NoVTM-MG
VTM-LG

## Slide 106
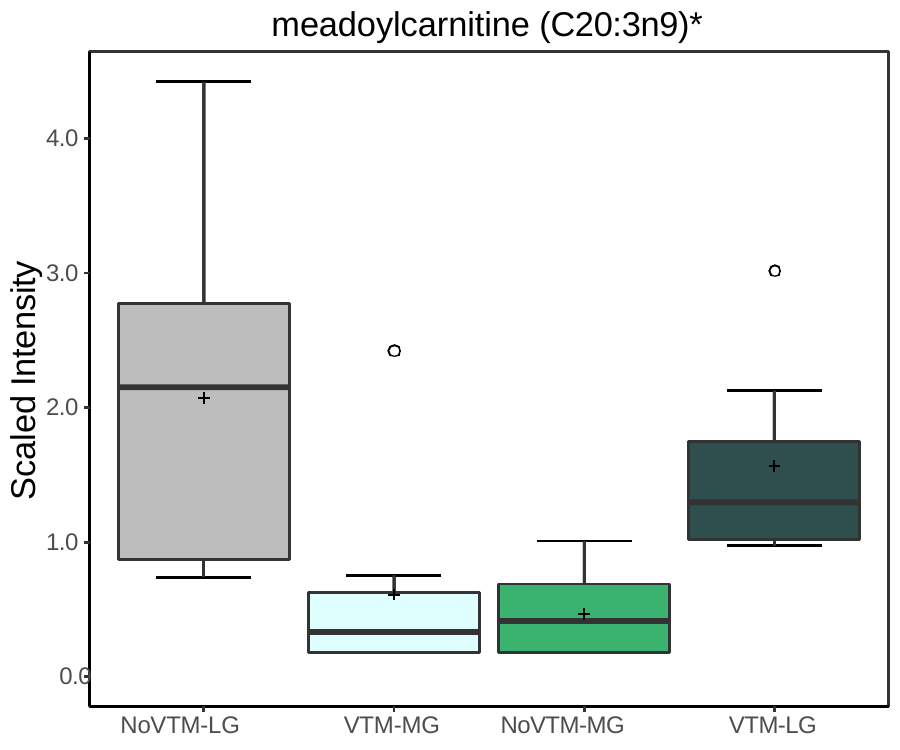

meadoylcarnitine (C20:3n9)*
4.0
3.0
Scaled Intensity
2.0
1.0
0.0
NoVTM-LG
VTM-MG
NoVTM-MG
VTM-LG

## Slide 107
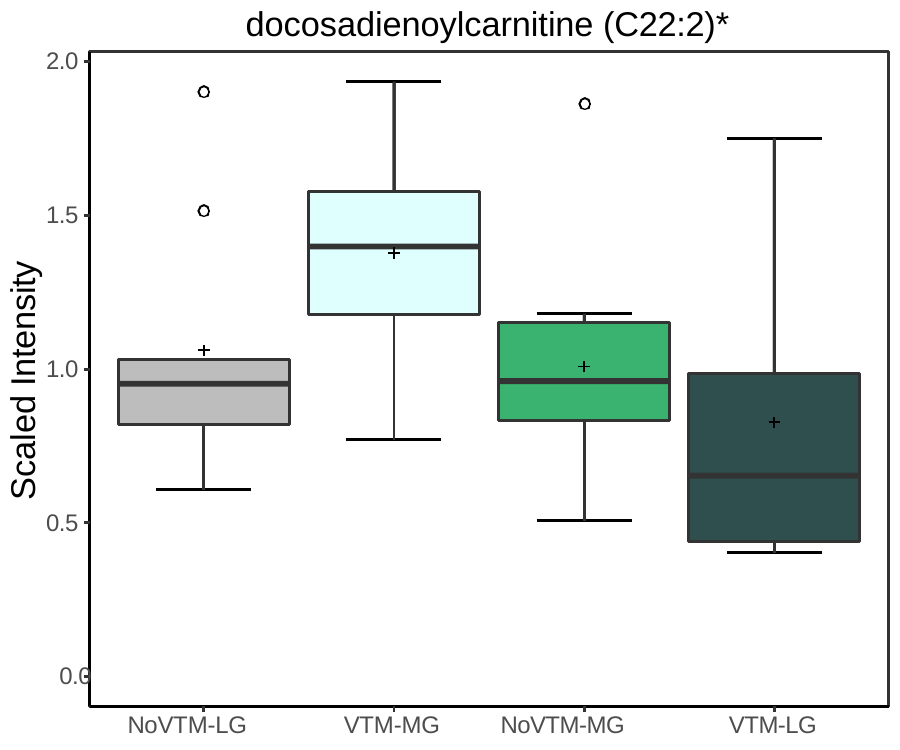

docosadienoylcarnitine (C22:2)*
2.0
1.5
Scaled Intensity
1.0
0.5
0.0
NoVTM-LG
VTM-MG
NoVTM-MG
VTM-LG

## Slide 108
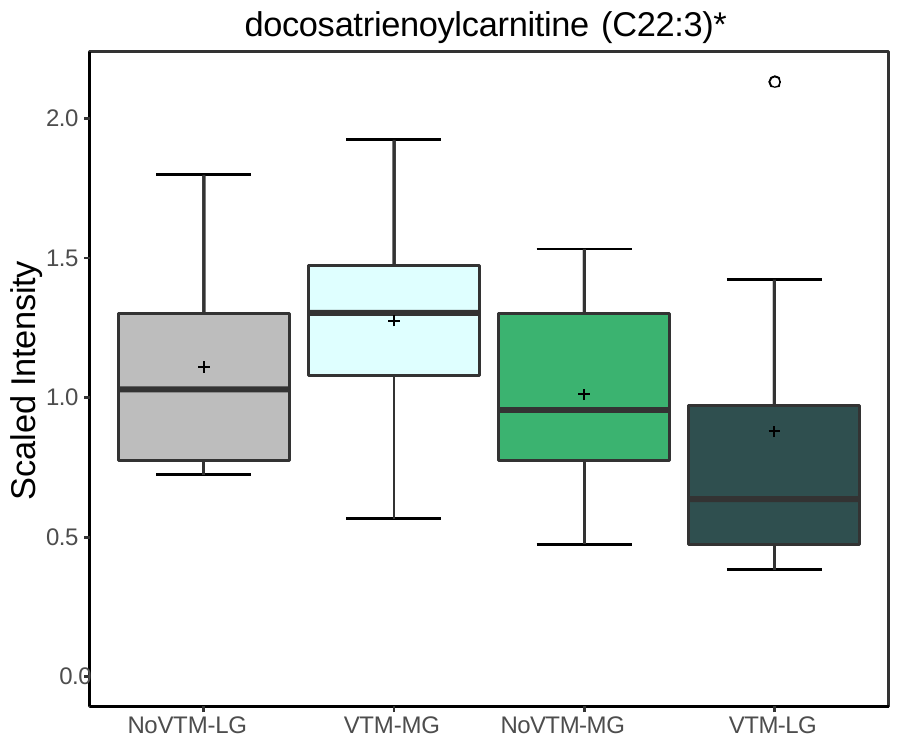

docosatrienoylcarnitine (C22:3)*
2.0
1.5
Scaled Intensity
1.0
0.5
0.0
NoVTM-LG
VTM-MG
NoVTM-MG
VTM-LG

## Slide 109
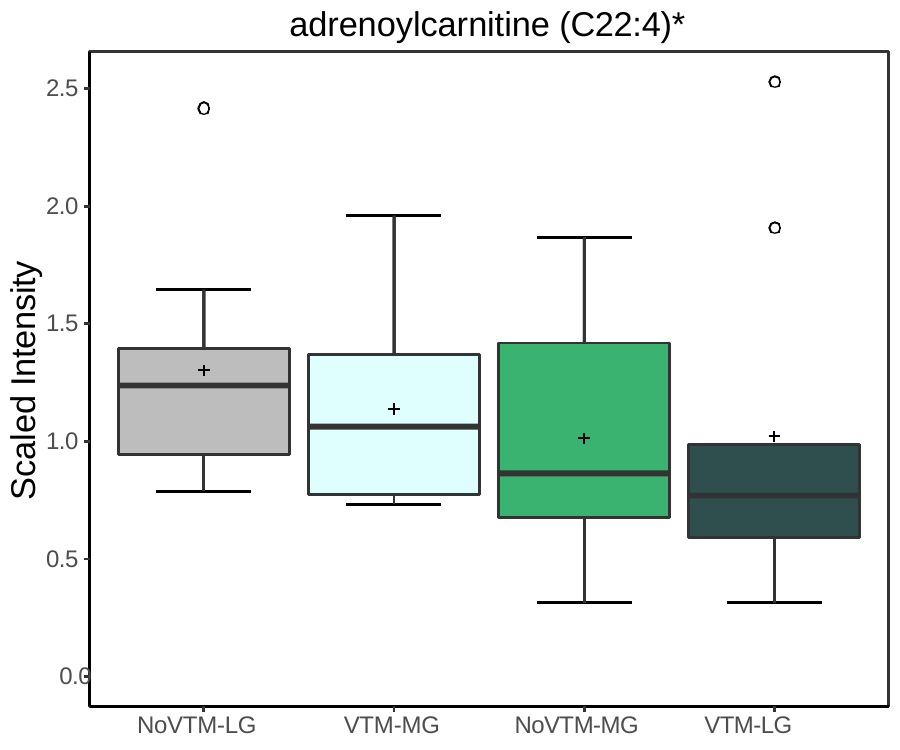

adrenoylcarnitine (C22:4)*
2.5
2.0
Scaled Intensity
1.5
1.0
0.5
0.0
NoVTM-LG
VTM-MG
NoVTM-MG
VTM-LG

## Slide 110
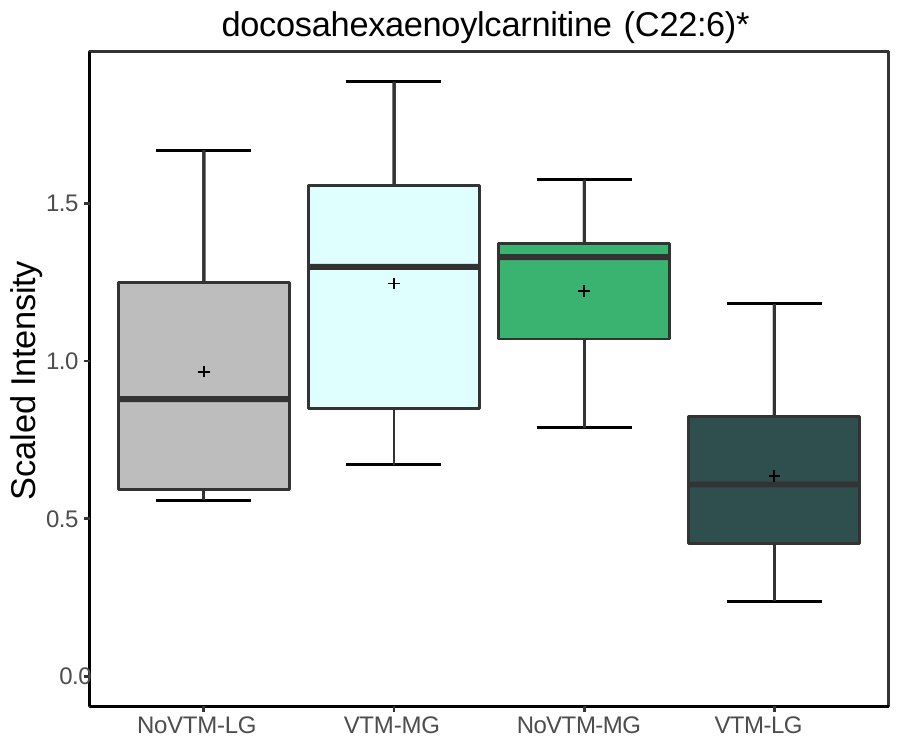

docosahexaenoylcarnitine (C22:6)*
1.5
Scaled Intensity
1.0
0.5
0.0
NoVTM-LG
VTM-MG
NoVTM-MG
VTM-LG

## Slide 111
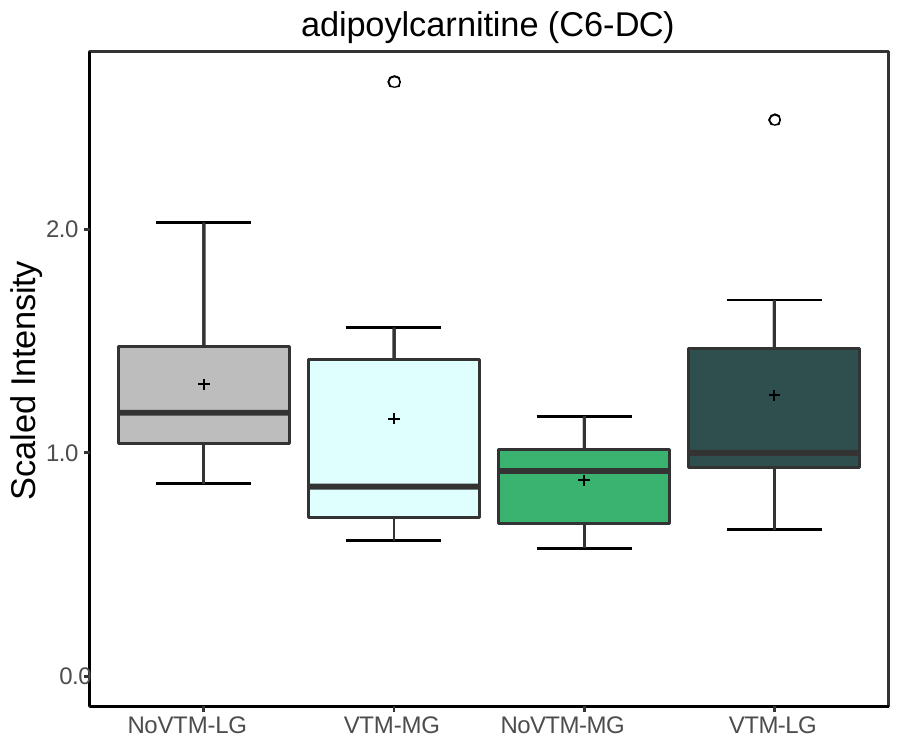

adipoylcarnitine (C6-DC)
2.0
Scaled Intensity
1.0
0.0
NoVTM-LG
VTM-MG
NoVTM-MG
VTM-LG

## Slide 112
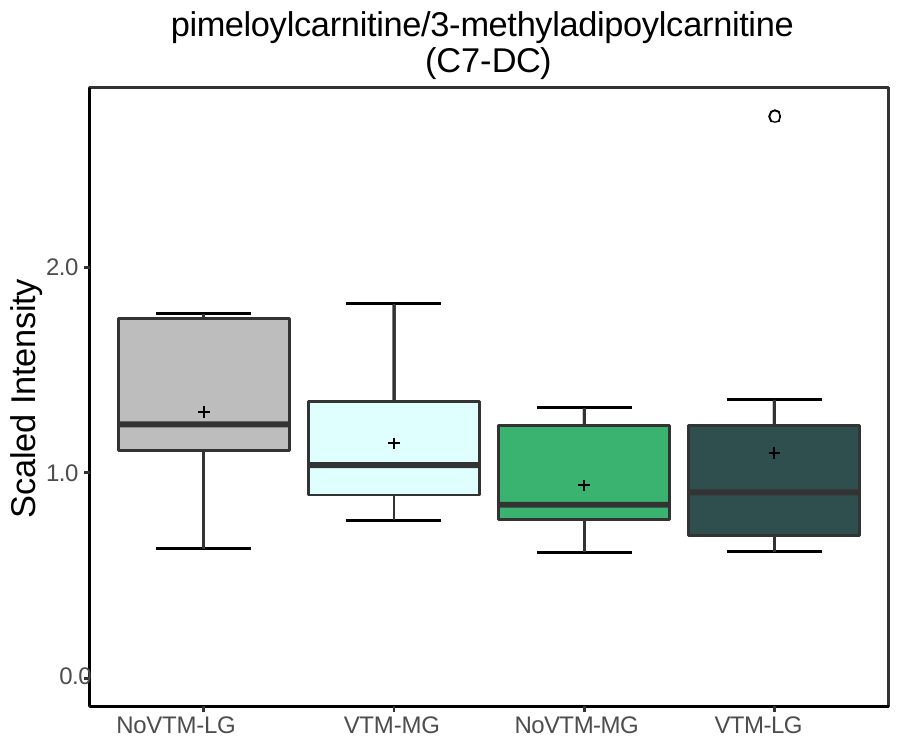

# pimeloylcarnitine/3-methyladipoylcarnitine (C7-DC)
2.0
Scaled Intensity
1.0
0.0
NoVTM-LG
VTM-MG
NoVTM-MG
VTM-LG

## Slide 113
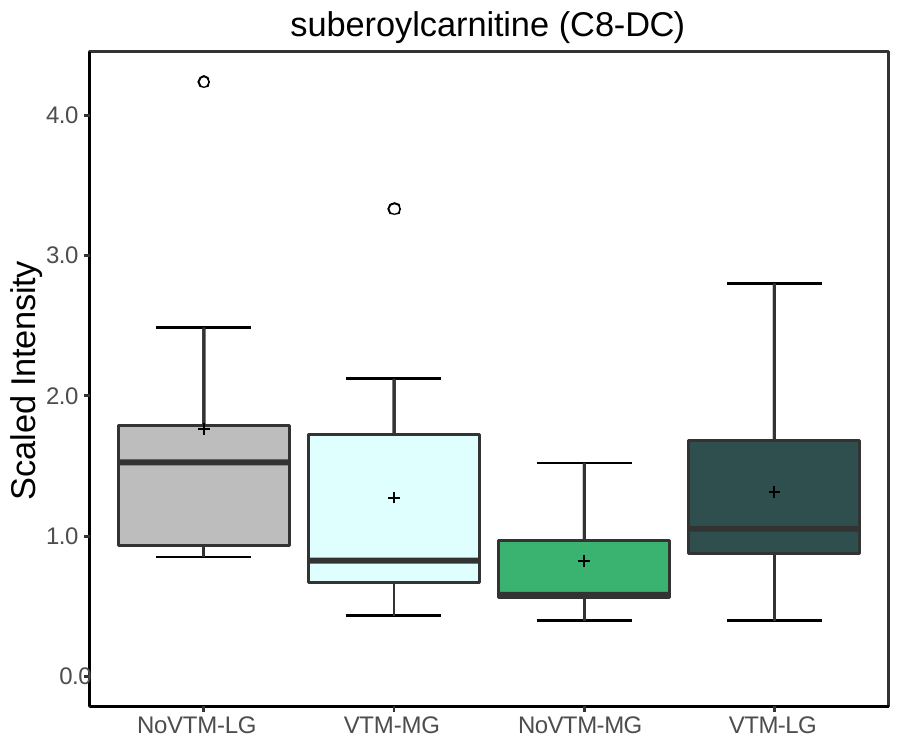

suberoylcarnitine (C8-DC)
4.0
3.0
Scaled Intensity
2.0
1.0
0.0
NoVTM-LG
VTM-MG
NoVTM-MG
VTM-LG

## Slide 114
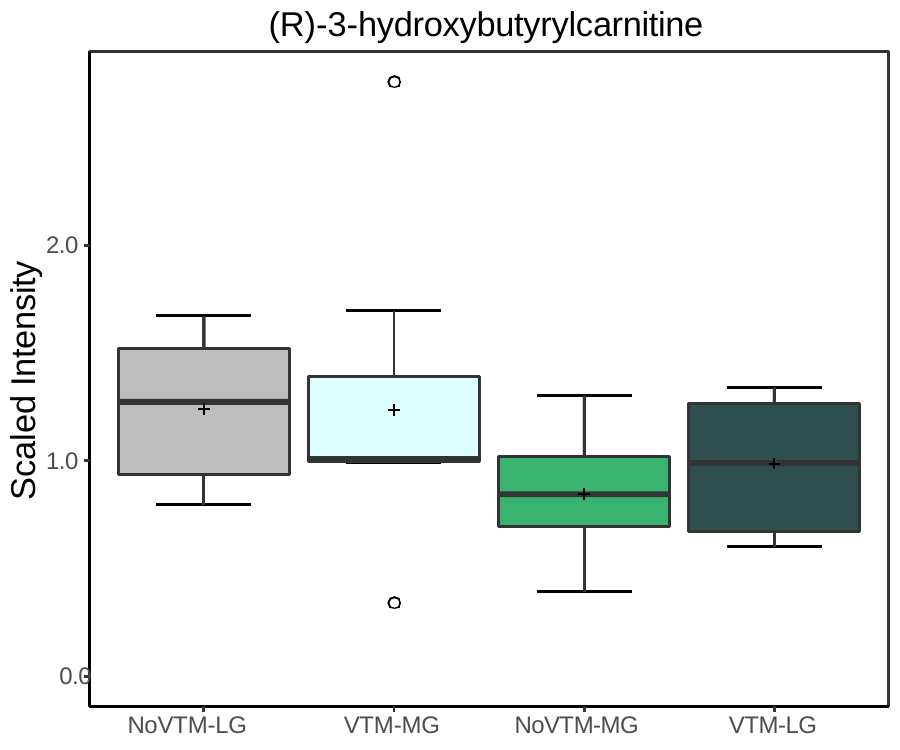

(R)-3-hydroxybutyrylcarnitine
2.0
Scaled Intensity
1.0
0.0
NoVTM-LG
VTM-MG
NoVTM-MG
VTM-LG

## Slide 115
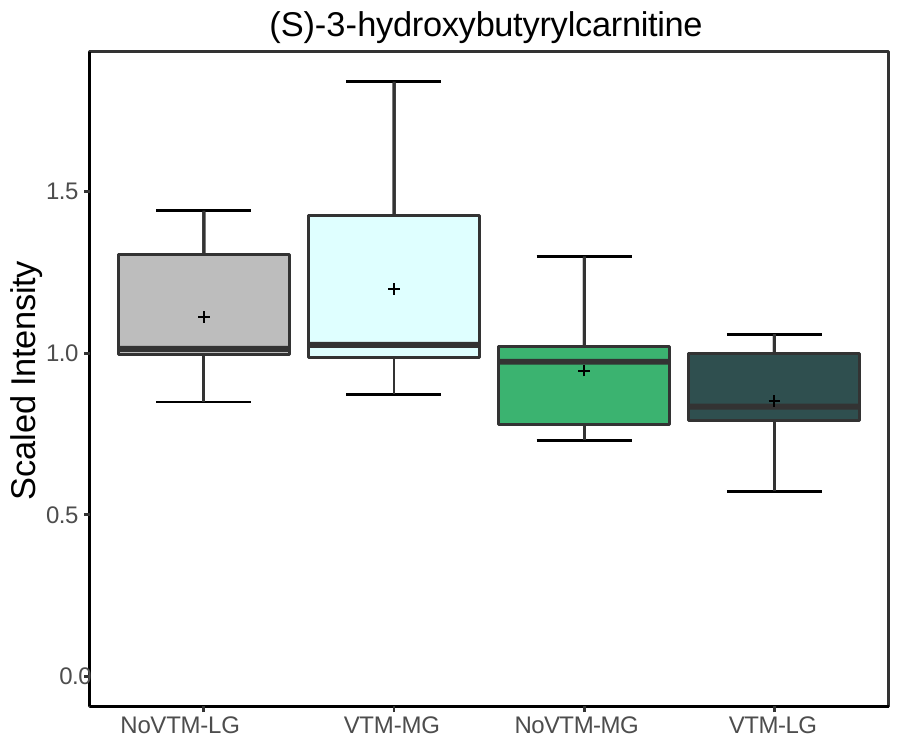

(S)-3-hydroxybutyrylcarnitine
1.5
Scaled Intensity
1.0
0.5
0.0
NoVTM-LG
VTM-MG
NoVTM-MG
VTM-LG

## Slide 116
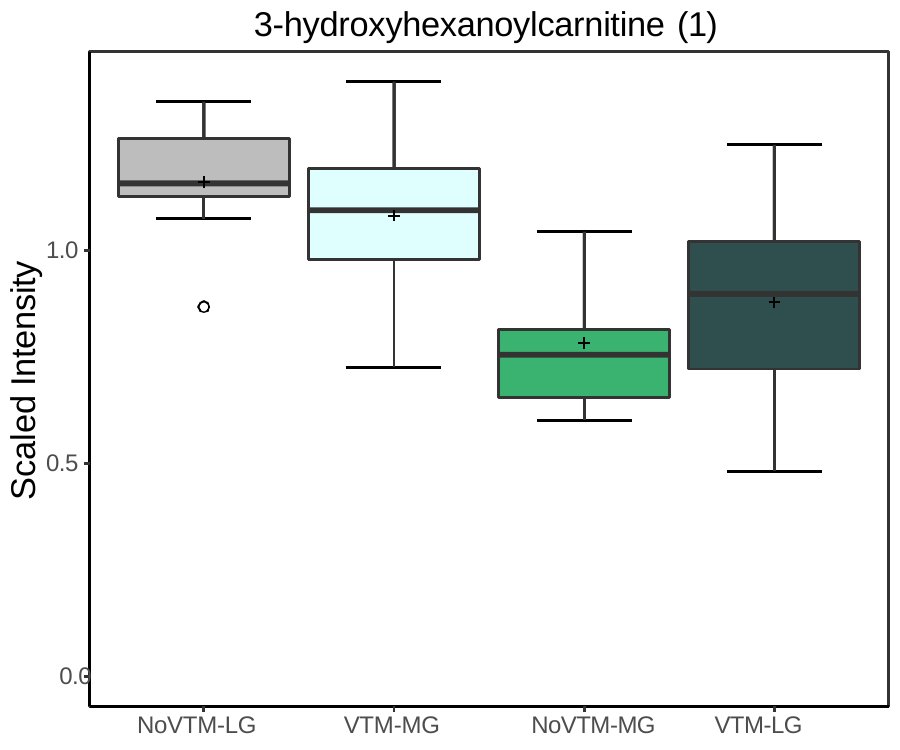

3-hydroxyhexanoylcarnitine (1)
1.0
Scaled Intensity
0.5
0.0
NoVTM-LG
VTM-MG
NoVTM-MG
VTM-LG

## Slide 117
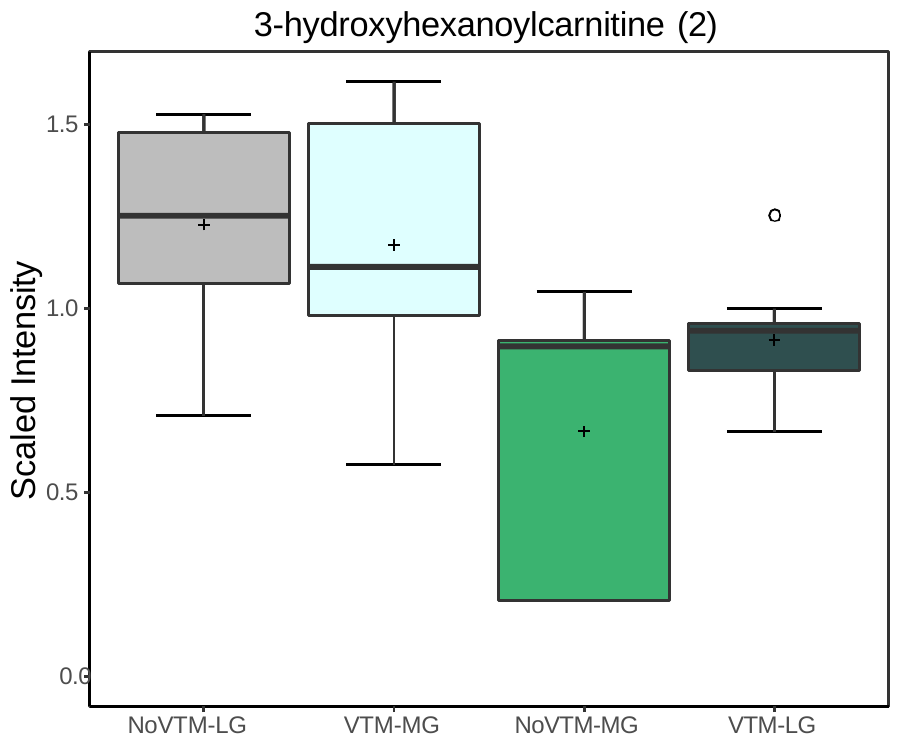

3-hydroxyhexanoylcarnitine (2)
1.5
Scaled Intensity
1.0
0.5
0.0
NoVTM-LG
VTM-MG
NoVTM-MG
VTM-LG

## Slide 118
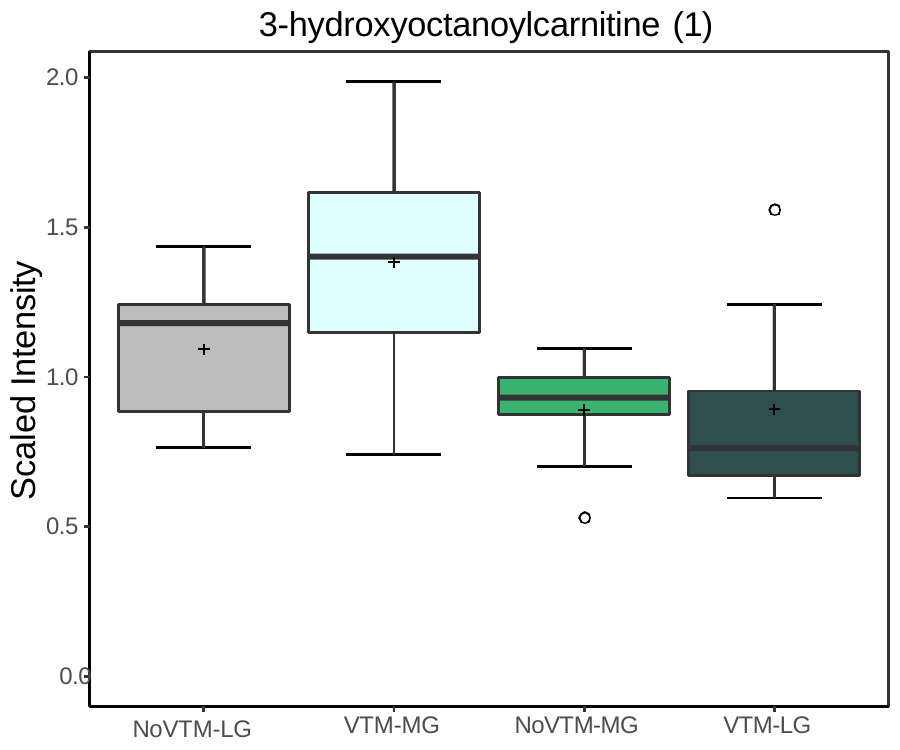

3-hydroxyoctanoylcarnitine (1)
2.0
1.5
Scaled Intensity
1.0
0.5
0.0
VTM-MG
NoVTM-MG
VTM-LG
NoVTM-LG

## Slide 119
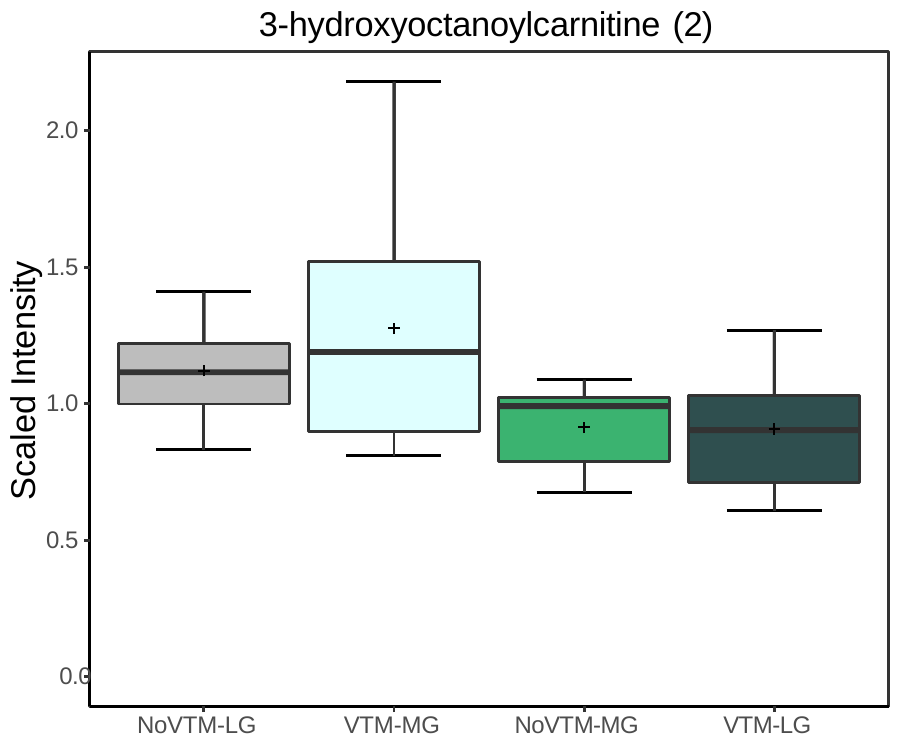

3-hydroxyoctanoylcarnitine (2)
2.0
1.5
Scaled Intensity
1.0
0.5
0.0
NoVTM-LG
VTM-MG
NoVTM-MG
VTM-LG

## Slide 120
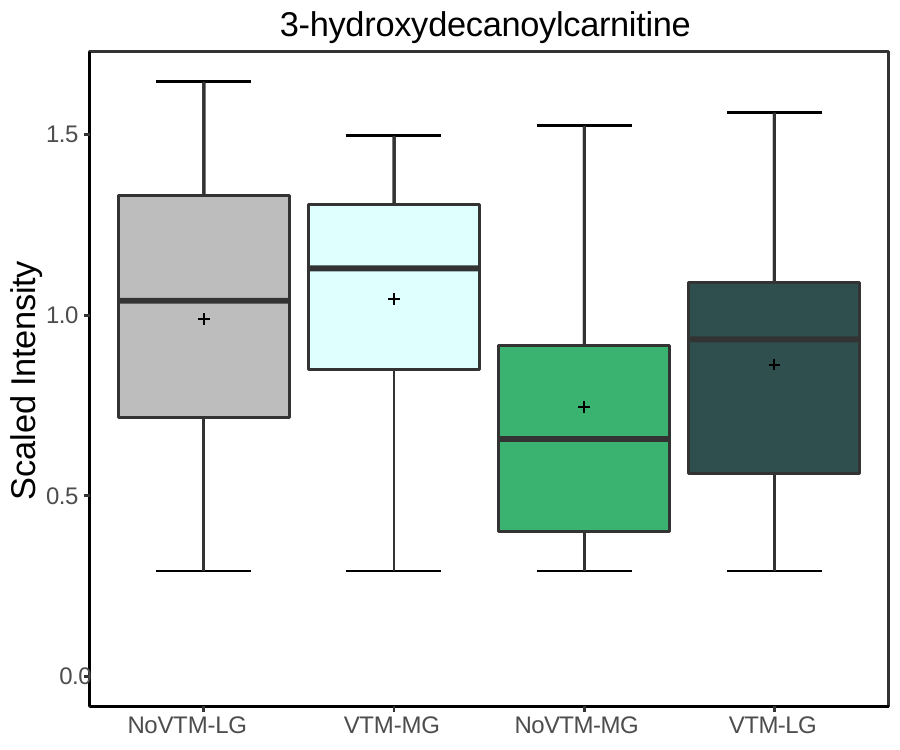

3-hydroxydecanoylcarnitine
1.5
Scaled Intensity
1.0
0.5
0.0
NoVTM-LG
VTM-MG
NoVTM-MG
VTM-LG

## Slide 121
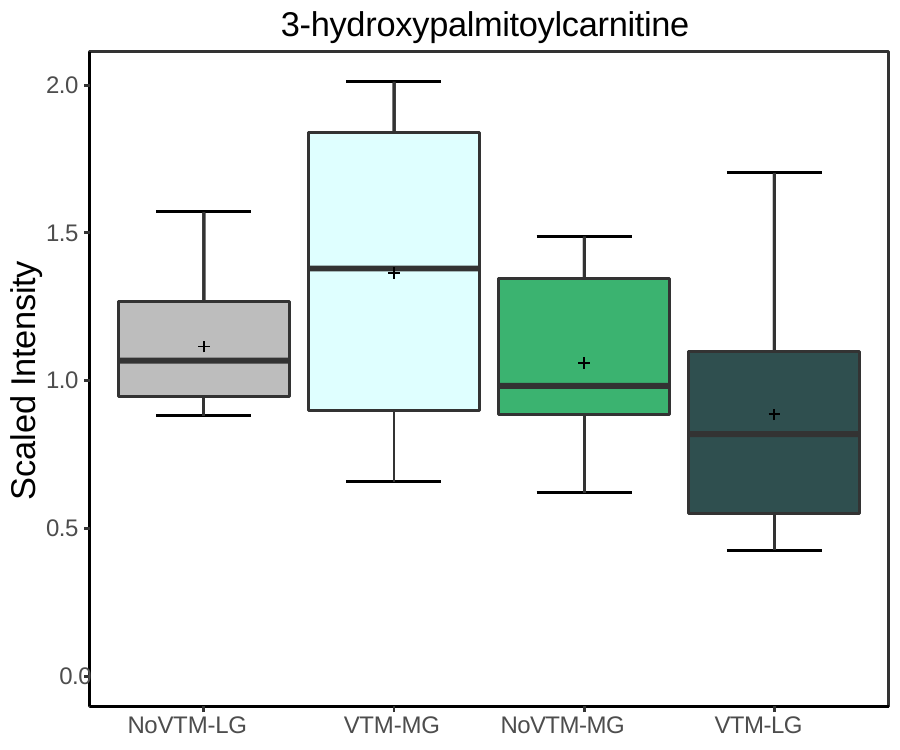

3-hydroxypalmitoylcarnitine
2.0
1.5
Scaled Intensity
1.0
0.5
0.0
NoVTM-LG
VTM-MG
NoVTM-MG
VTM-LG

## Slide 122
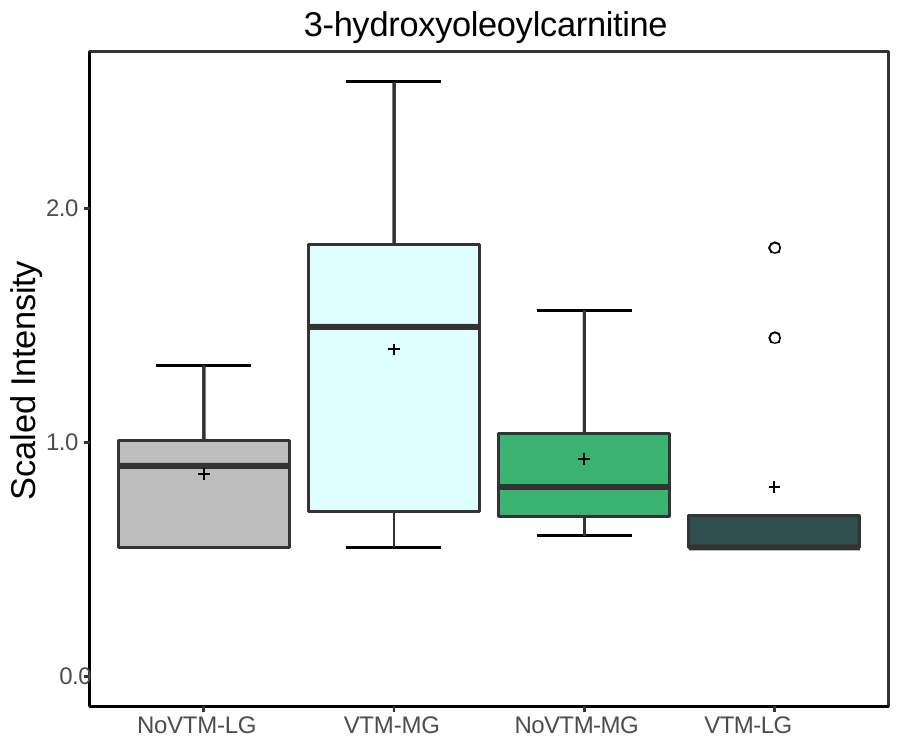

3-hydroxyoleoylcarnitine
2.0
Scaled Intensity
1.0
0.0
NoVTM-LG
VTM-MG
NoVTM-MG
VTM-LG

## Slide 123
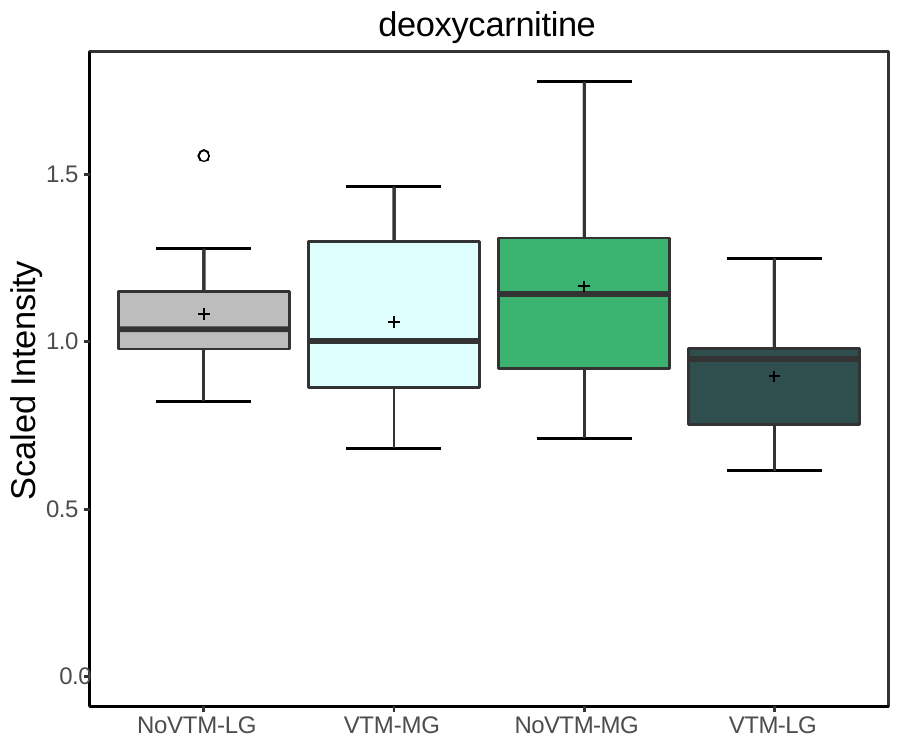

deoxycarnitine
1.5
Scaled Intensity
1.0
0.5
0.0
NoVTM-LG
VTM-MG
NoVTM-MG
VTM-LG

## Slide 124
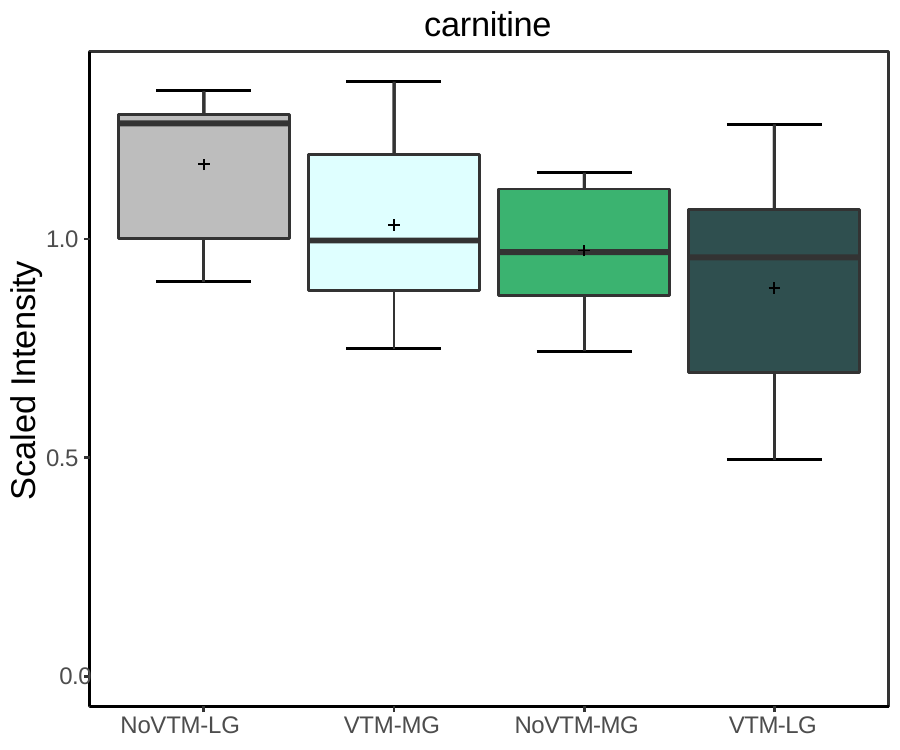

carnitine
1.0
Scaled Intensity
0.5
0.0
NoVTM-LG
VTM-MG
NoVTM-MG
VTM-LG

## Slide 125
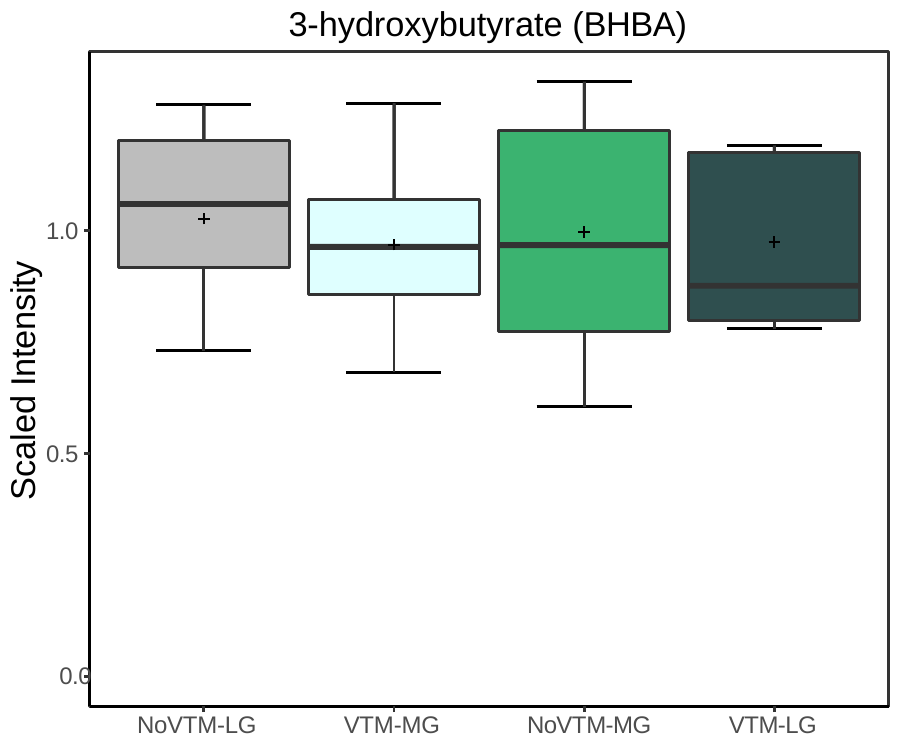

3-hydroxybutyrate (BHBA)
1.0
Scaled Intensity
0.5
0.0
NoVTM-LG
VTM-MG
NoVTM-MG
VTM-LG

## Slide 126
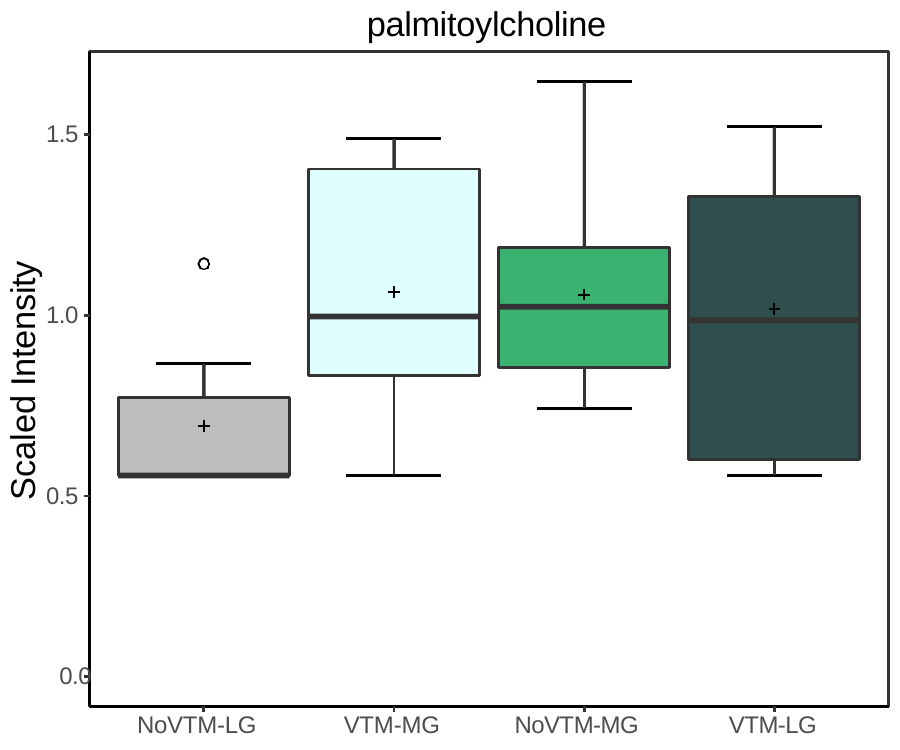

palmitoylcholine
1.5
Scaled Intensity
1.0
0.5
0.0
NoVTM-LG
VTM-MG
NoVTM-MG
VTM-LG

## Slide 127
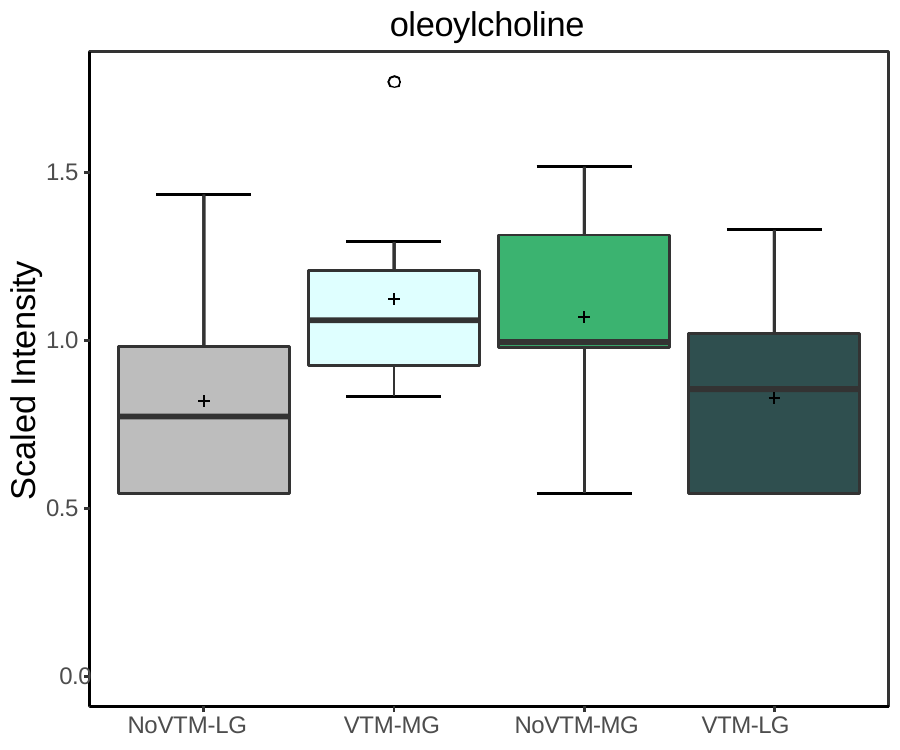

oleoylcholine
1.5
Scaled Intensity
1.0
0.5
0.0
NoVTM-LG
VTM-MG
NoVTM-MG
VTM-LG

## Slide 128
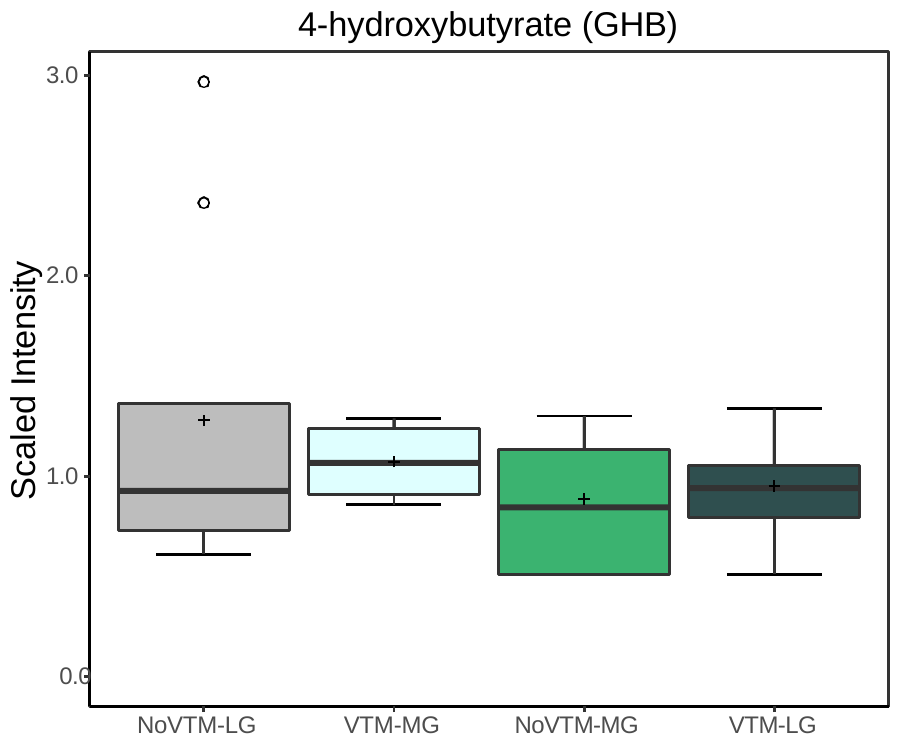

4-hydroxybutyrate (GHB)
3.0
Scaled Intensity
2.0
1.0
0.0
NoVTM-LG
VTM-MG
NoVTM-MG
VTM-LG

## Slide 129
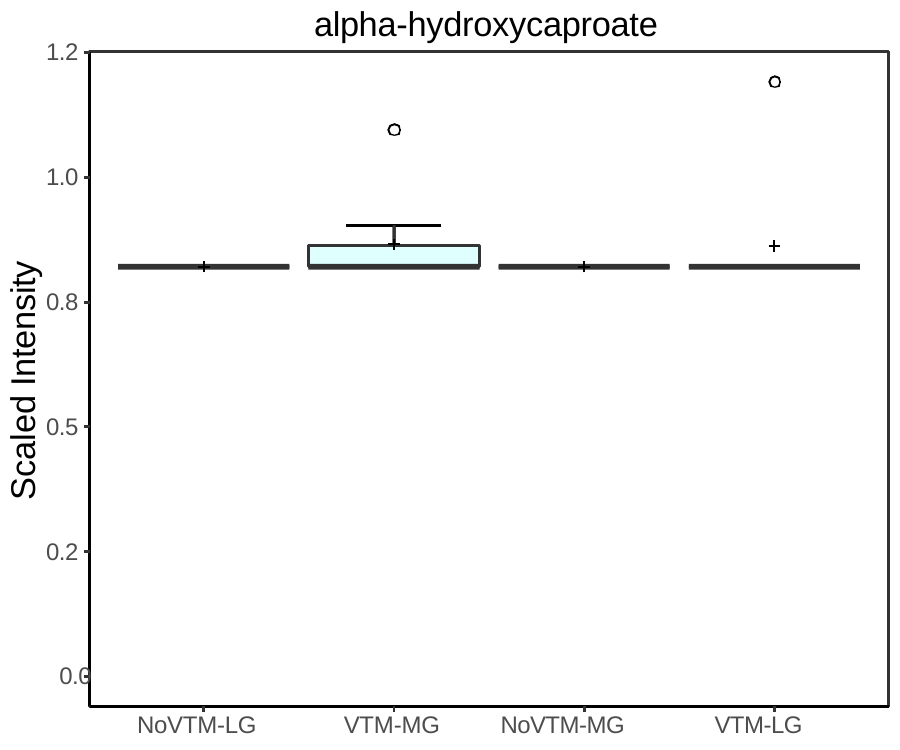

alpha-hydroxycaproate
1.2
1.0
Scaled Intensity
0.8
0.5
0.2
0.0
NoVTM-LG
VTM-MG
NoVTM-MG
VTM-LG

## Slide 130
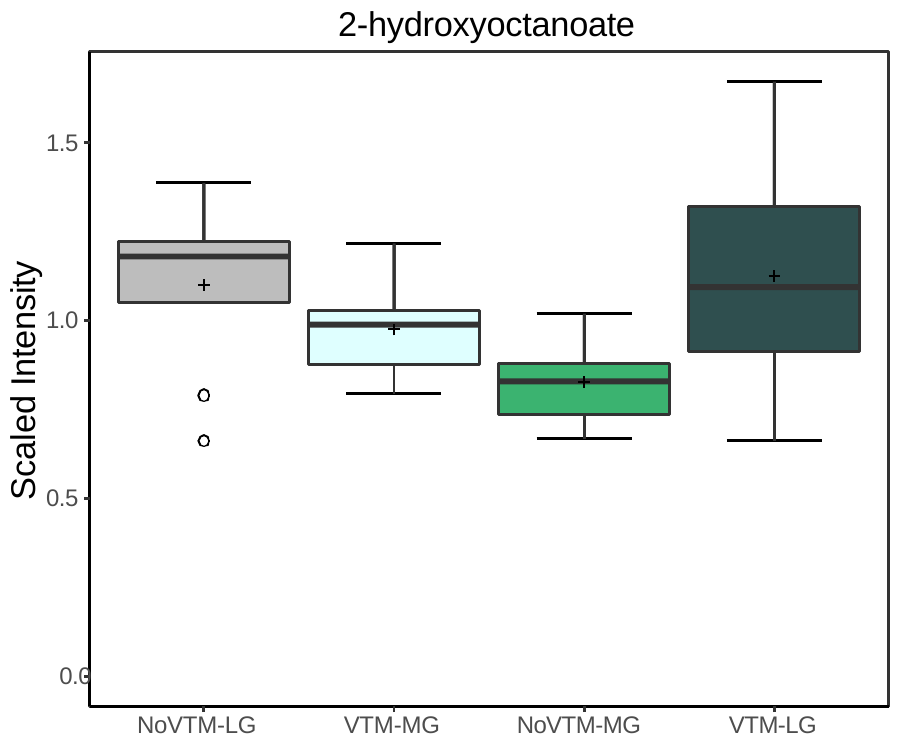

2-hydroxyoctanoate
1.5
Scaled Intensity
1.0
0.5
0.0
NoVTM-LG
VTM-MG
NoVTM-MG
VTM-LG

## Slide 131
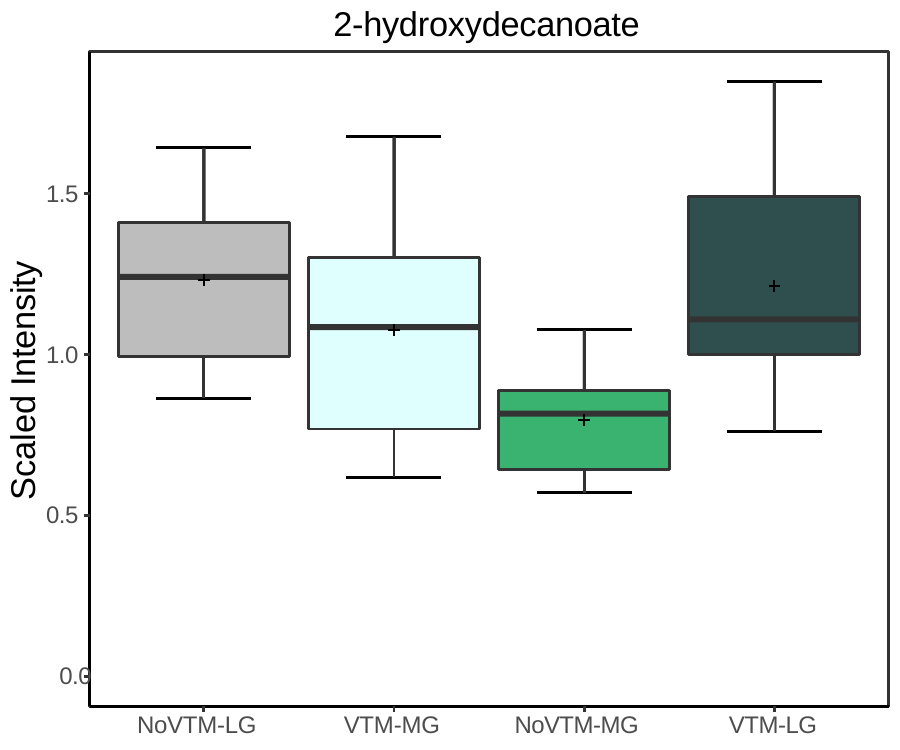

2-hydroxydecanoate
1.5
Scaled Intensity
1.0
0.5
0.0
NoVTM-LG
VTM-MG
NoVTM-MG
VTM-LG

## Slide 132
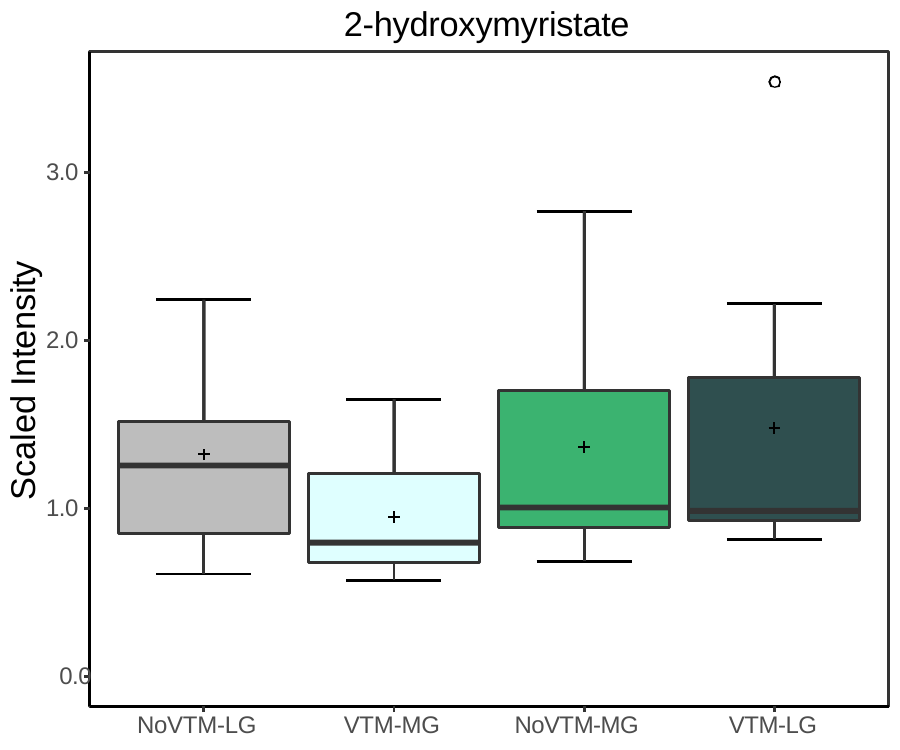

2-hydroxymyristate
3.0
Scaled Intensity
2.0
1.0
0.0
NoVTM-LG
VTM-MG
NoVTM-MG
VTM-LG

## Slide 133
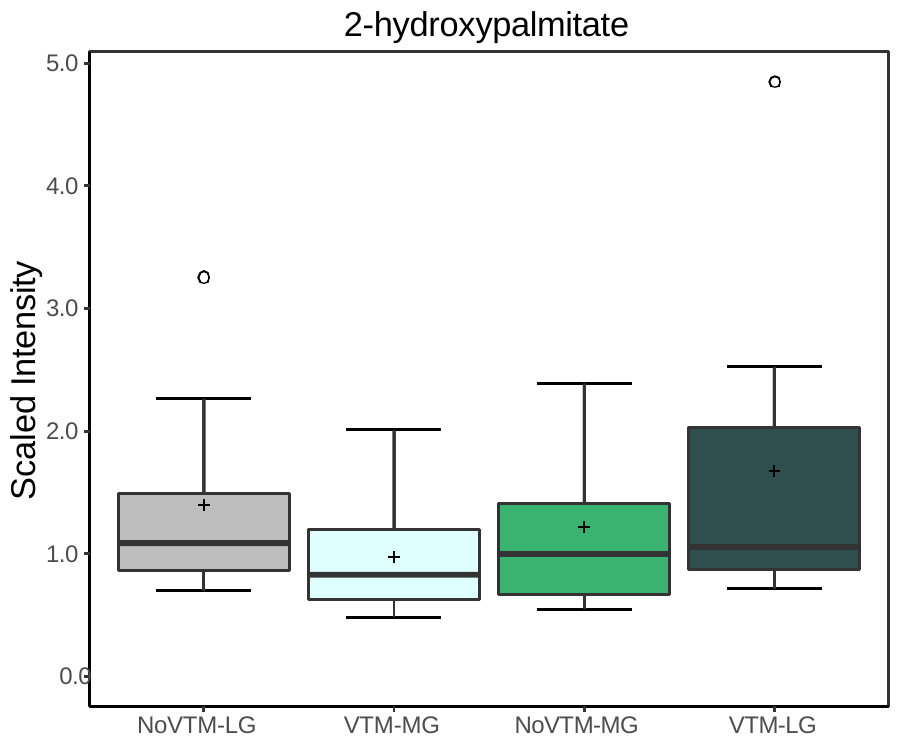

2-hydroxypalmitate
5.0
4.0
Scaled Intensity
3.0
2.0
1.0
0.0
NoVTM-LG
VTM-MG
NoVTM-MG
VTM-LG

## Slide 134
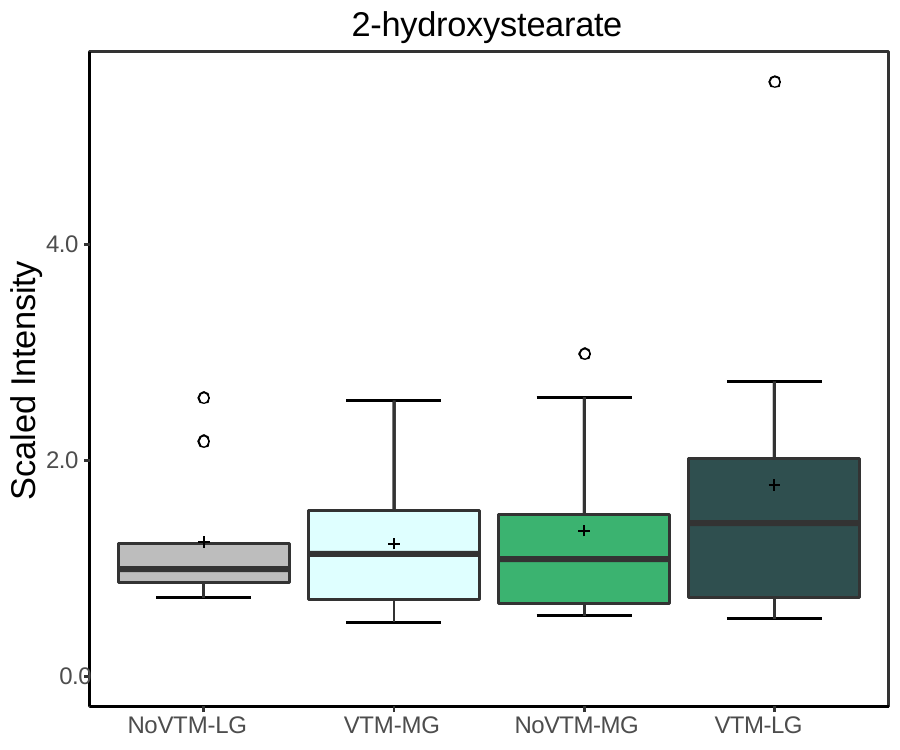

2-hydroxystearate
4.0
Scaled Intensity
2.0
0.0
NoVTM-LG
VTM-MG
NoVTM-MG
VTM-LG

## Slide 135
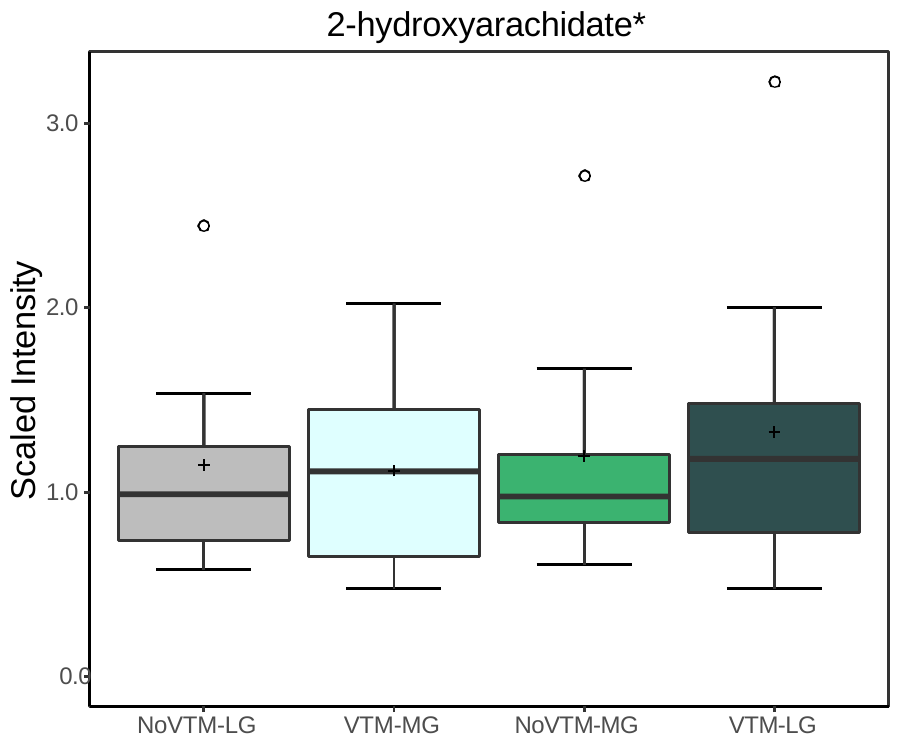

2-hydroxyarachidate*
3.0
Scaled Intensity
2.0
1.0
0.0
NoVTM-LG
VTM-MG
NoVTM-MG
VTM-LG

## Slide 136
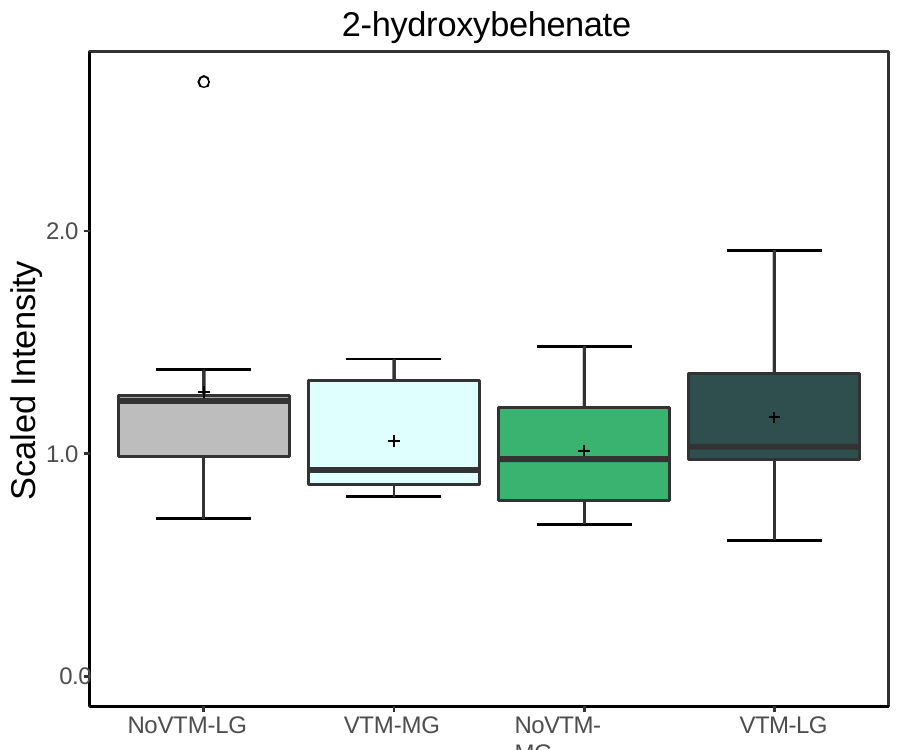

2-hydroxybehenate
2.0
Scaled Intensity
1.0
0.0
NoVTM-LG
VTM-MG
NoVTM-MG
VTM-LG

## Slide 137
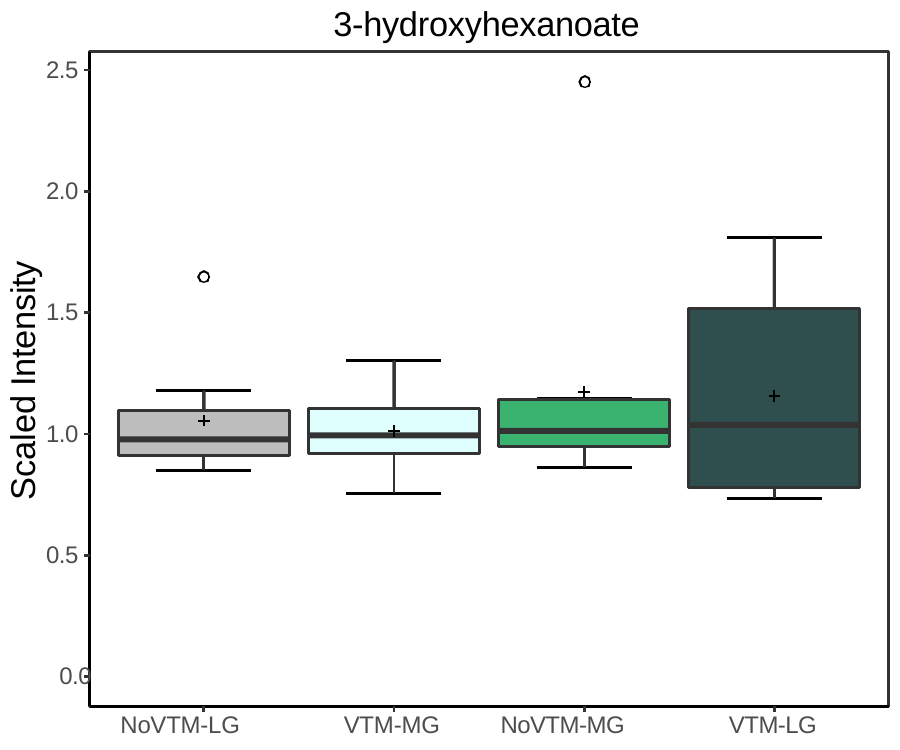

3-hydroxyhexanoate
2.5
2.0
Scaled Intensity
1.5
1.0
0.5
0.0
NoVTM-LG
VTM-MG
NoVTM-MG
VTM-LG

## Slide 138
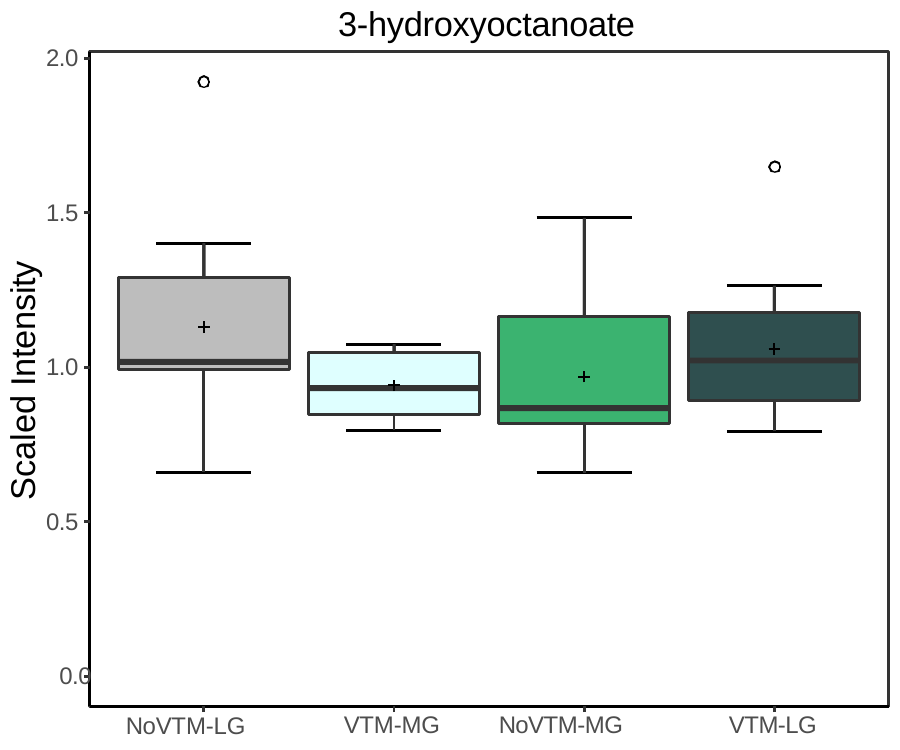

3-hydroxyoctanoate
2.0
1.5
Scaled Intensity
1.0
0.5
0.0
VTM-MG
NoVTM-MG
VTM-LG
NoVTM-LG

## Slide 139
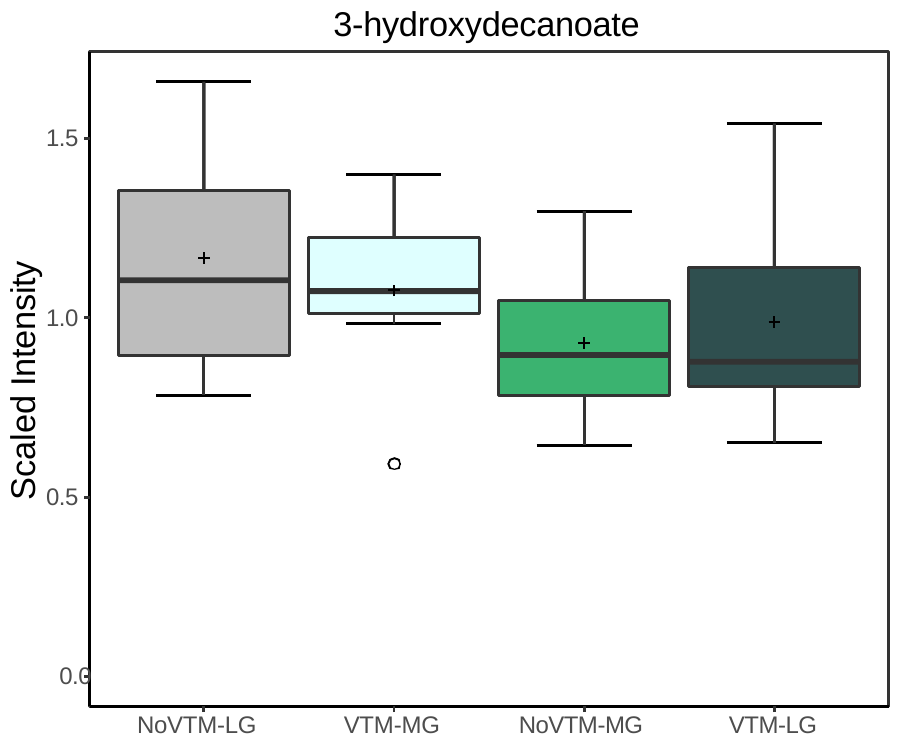

3-hydroxydecanoate
1.5
Scaled Intensity
1.0
0.5
0.0
NoVTM-LG
VTM-MG
NoVTM-MG
VTM-LG

## Slide 140
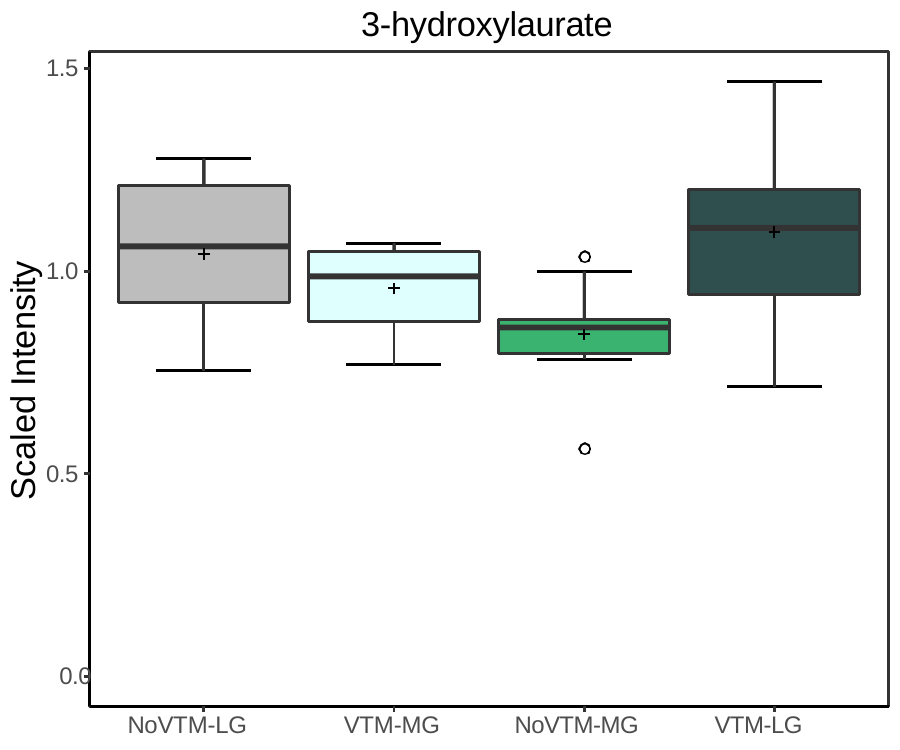

3-hydroxylaurate
1.5
1.0
Scaled Intensity
0.5
0.0
NoVTM-LG
VTM-MG
NoVTM-MG
VTM-LG

## Slide 141
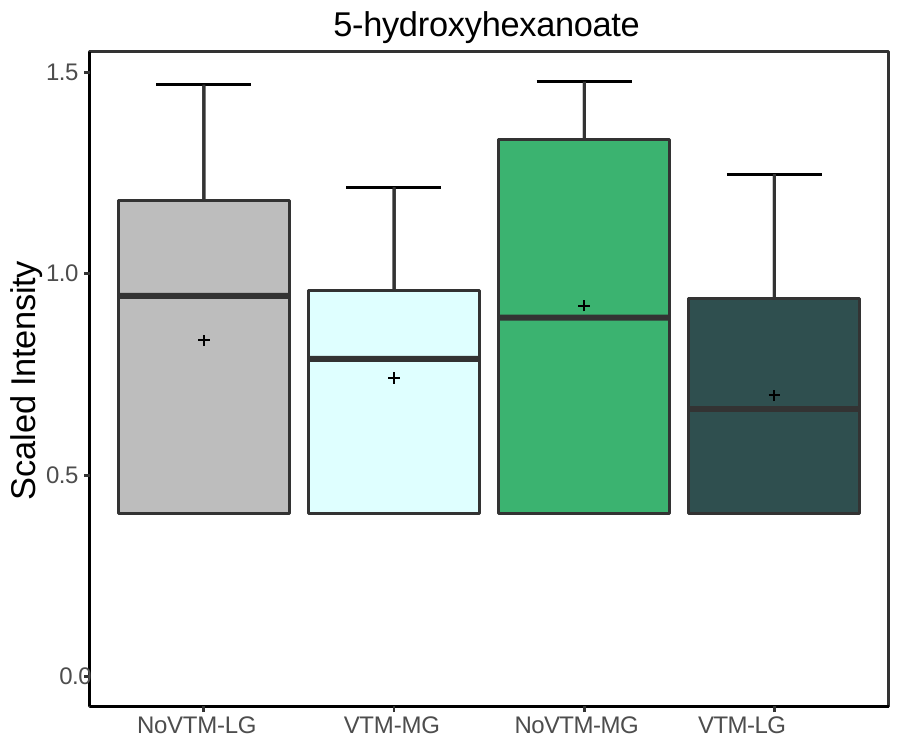

5-hydroxyhexanoate
1.5
1.0
Scaled Intensity
0.5
0.0
NoVTM-LG
VTM-MG
NoVTM-MG
VTM-LG

## Slide 142
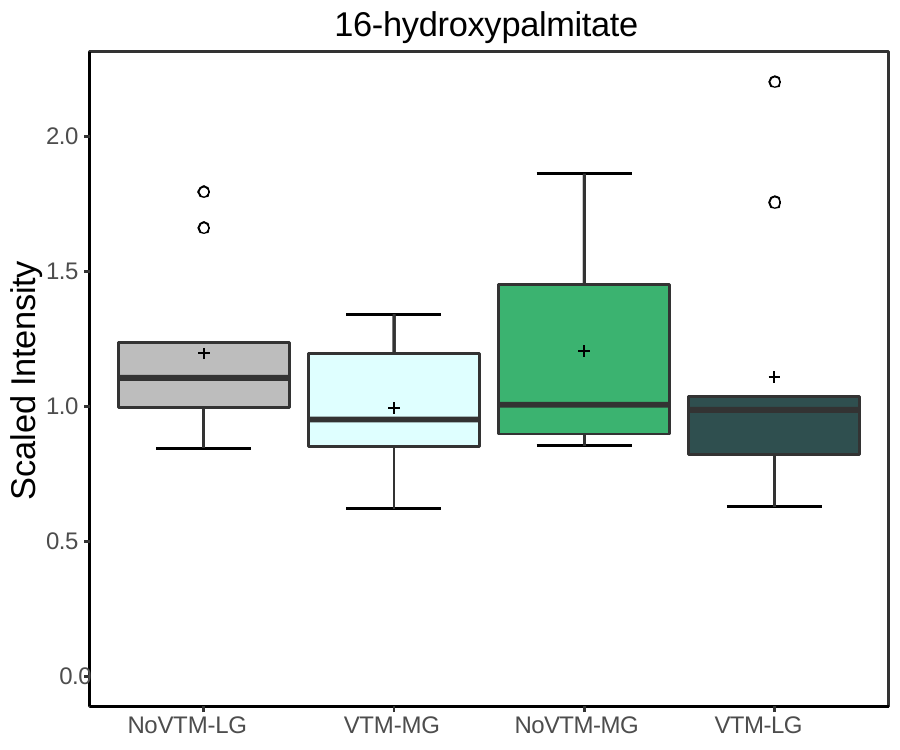

16-hydroxypalmitate
2.0
1.5
Scaled Intensity
1.0
0.5
0.0
NoVTM-LG
VTM-MG
NoVTM-MG
VTM-LG

## Slide 143
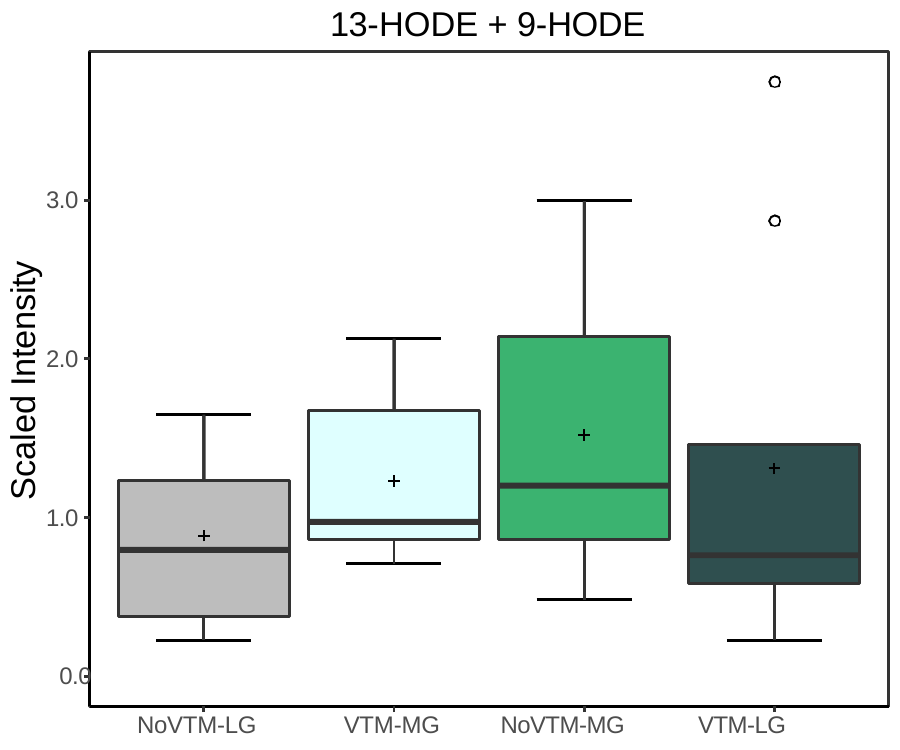

13-HODE + 9-HODE
3.0
Scaled Intensity
2.0
1.0
0.0
NoVTM-LG
VTM-MG
NoVTM-MG
VTM-LG

## Slide 144
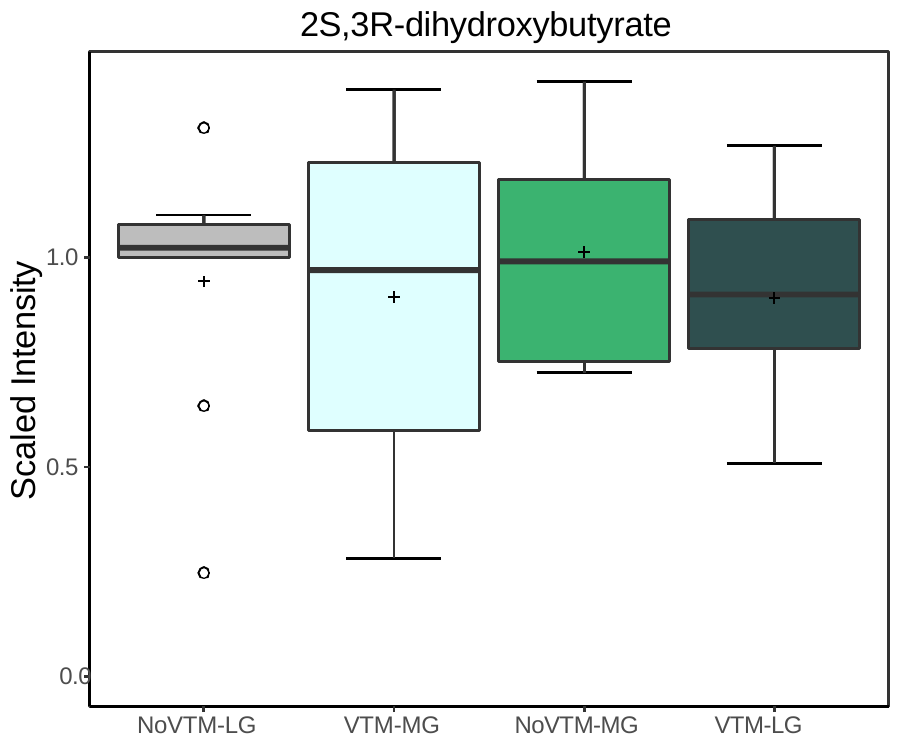

2S,3R-dihydroxybutyrate
1.0
Scaled Intensity
0.5
0.0
NoVTM-LG
VTM-MG
NoVTM-MG
VTM-LG

## Slide 145
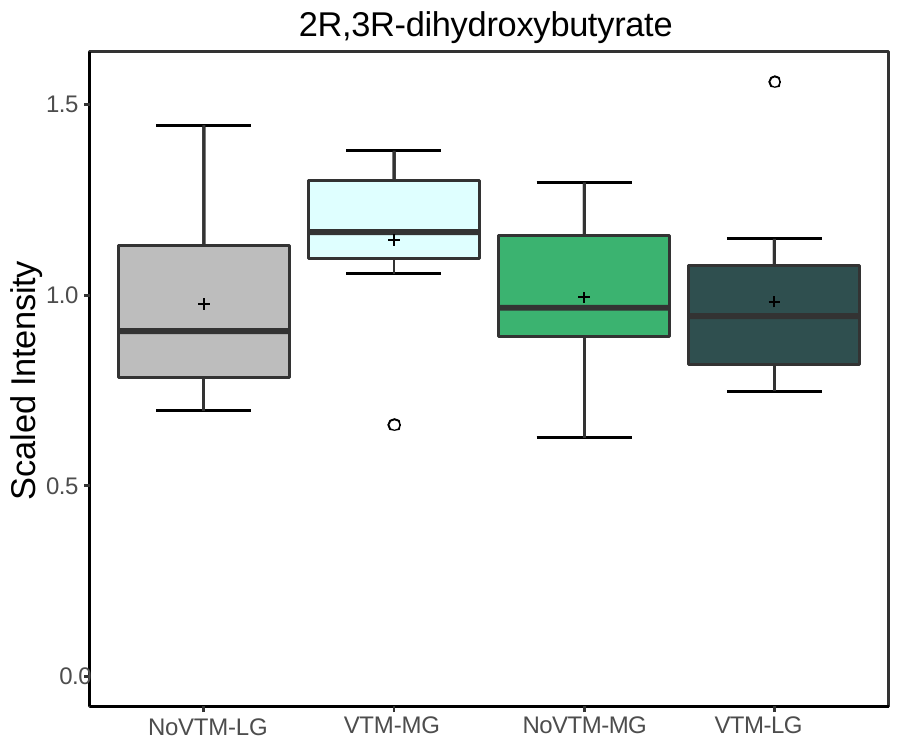

2R,3R-dihydroxybutyrate
1.5
Scaled Intensity
1.0
0.5
0.0
VTM-MG
NoVTM-MG
VTM-LG
NoVTM-LG

## Slide 146
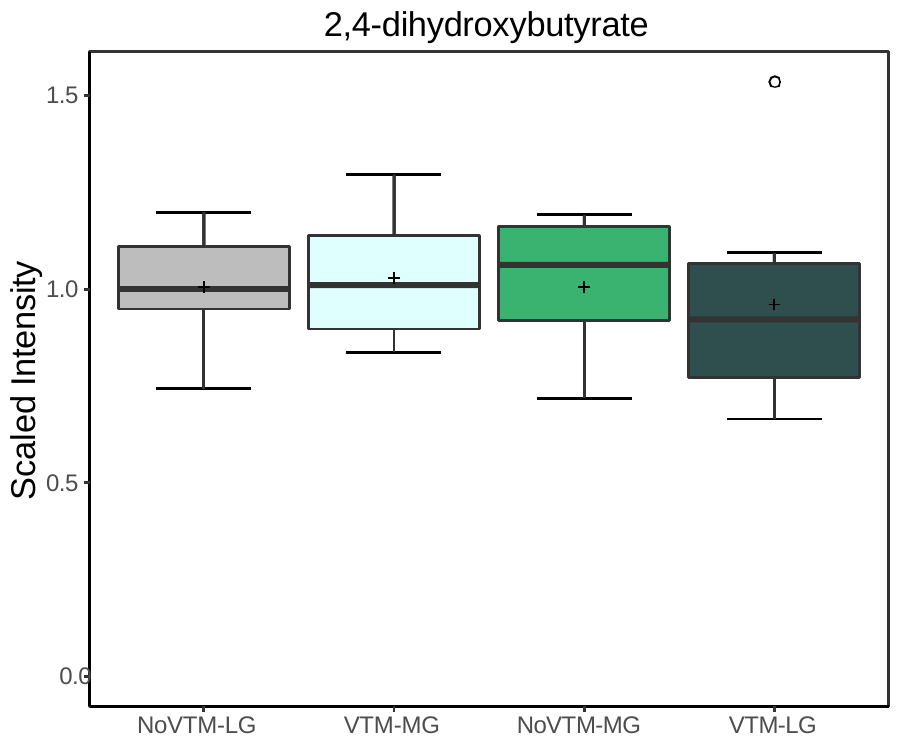

2,4-dihydroxybutyrate
1.5
Scaled Intensity
1.0
0.5
0.0
NoVTM-LG
VTM-MG
NoVTM-MG
VTM-LG

## Slide 147
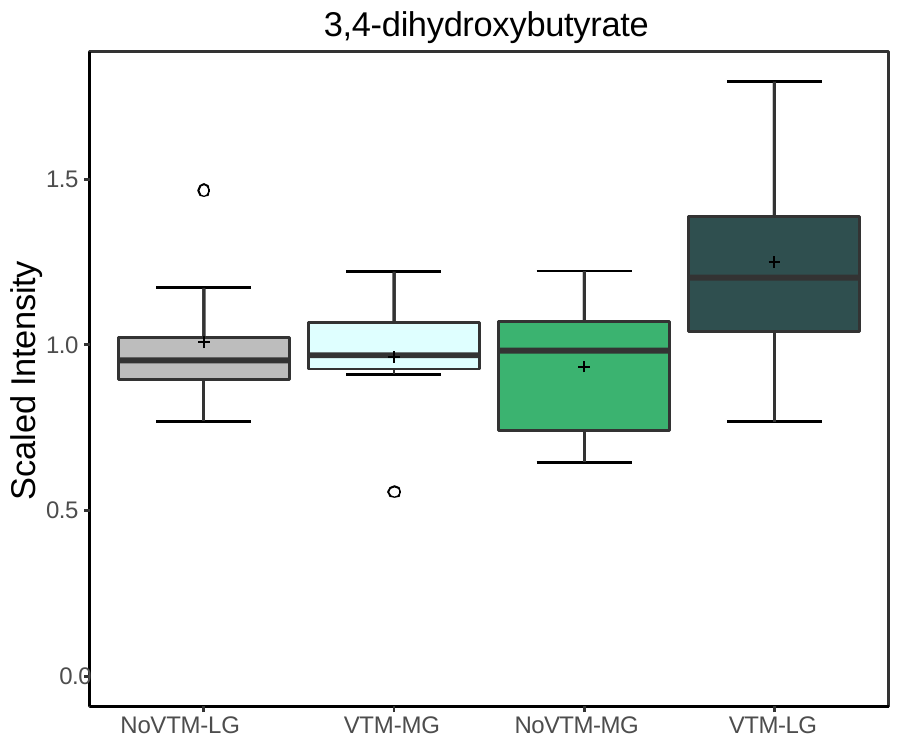

3,4-dihydroxybutyrate
1.5
Scaled Intensity
1.0
0.5
0.0
NoVTM-LG
VTM-MG
NoVTM-MG
VTM-LG

## Slide 148
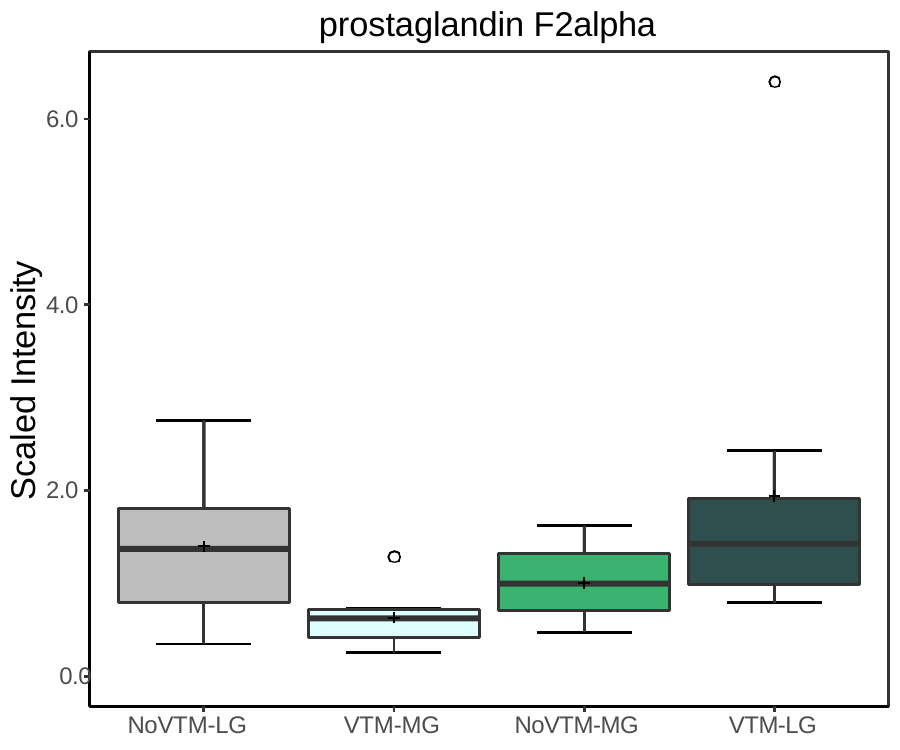

prostaglandin F2alpha
6.0
Scaled Intensity
4.0
2.0
0.0
NoVTM-LG
VTM-MG
NoVTM-MG
VTM-LG

## Slide 149
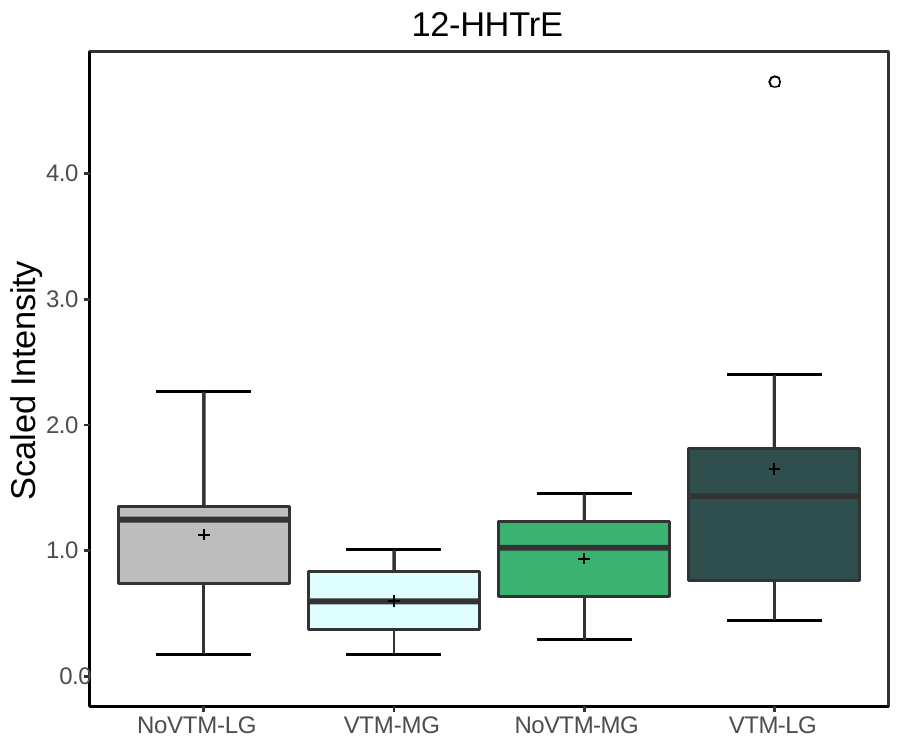

12-HHTrE
4.0
Scaled Intensity
3.0
2.0
1.0
0.0
NoVTM-LG
VTM-MG
NoVTM-MG
VTM-LG

## Slide 150
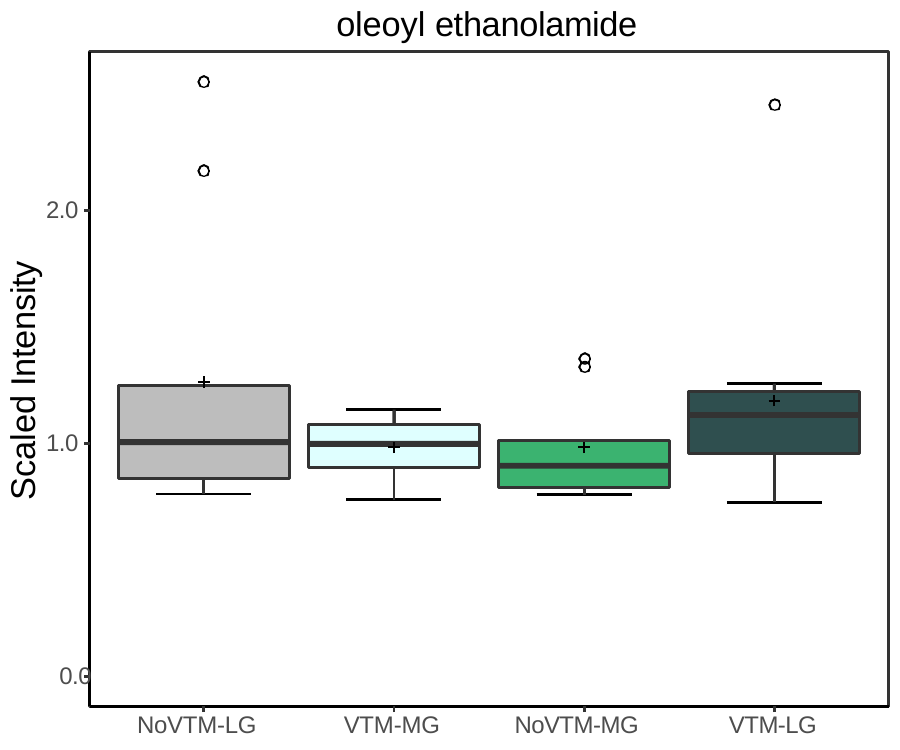

oleoyl ethanolamide
2.0
Scaled Intensity
1.0
0.0
NoVTM-LG
VTM-MG
NoVTM-MG
VTM-LG

## Slide 151
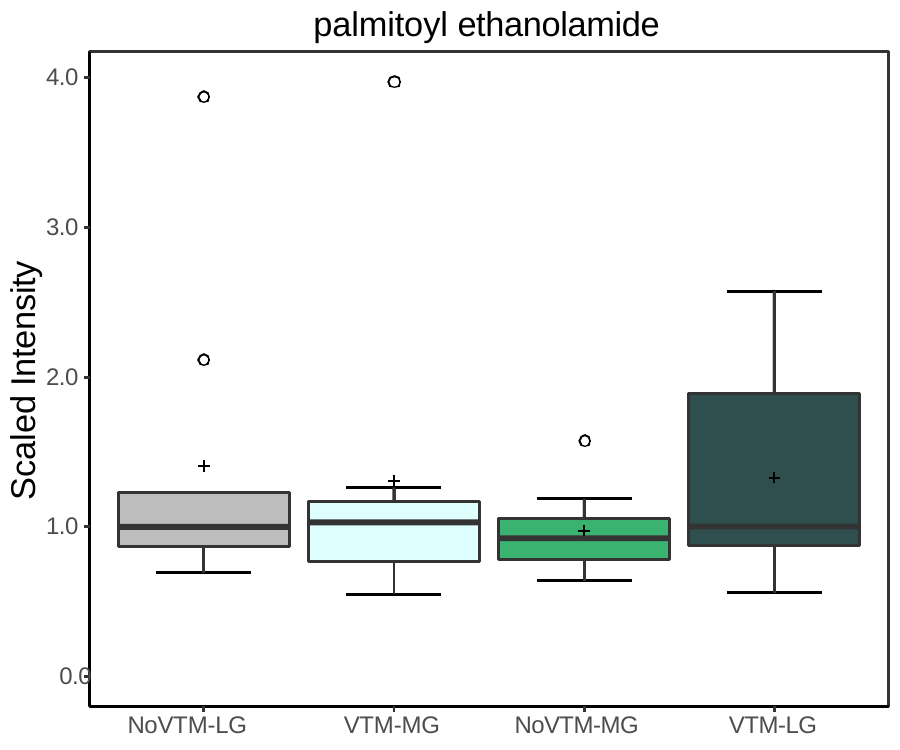

palmitoyl ethanolamide
4.0
3.0
Scaled Intensity
2.0
1.0
0.0
NoVTM-LG
VTM-MG
NoVTM-MG
VTM-LG

## Slide 152
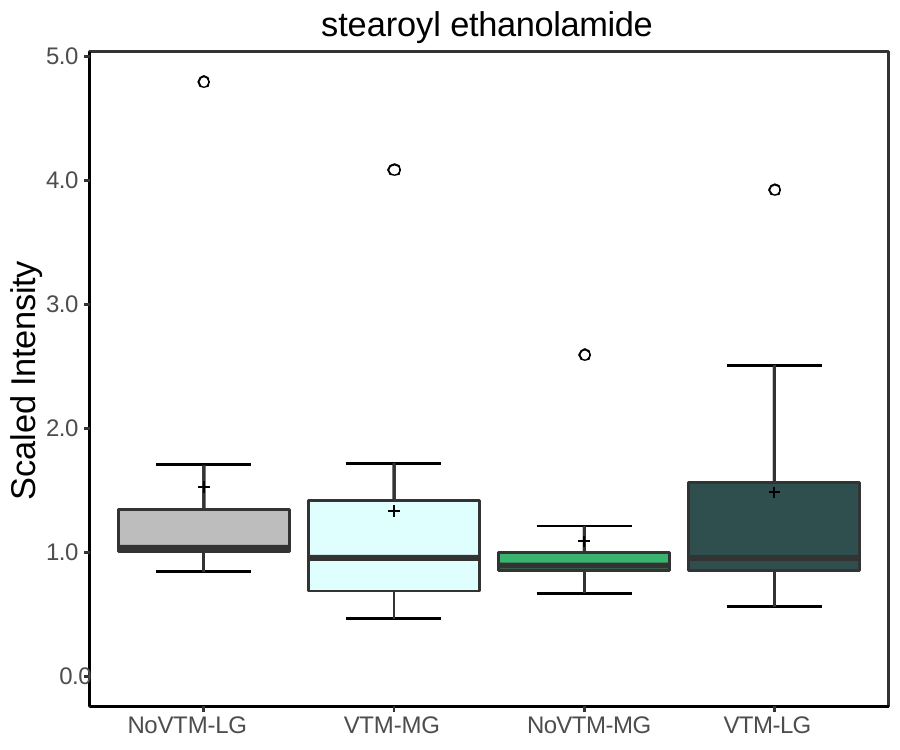

stearoyl ethanolamide
5.0
4.0
Scaled Intensity
3.0
2.0
1.0
0.0
NoVTM-LG
VTM-MG
NoVTM-MG
VTM-LG

## Slide 153
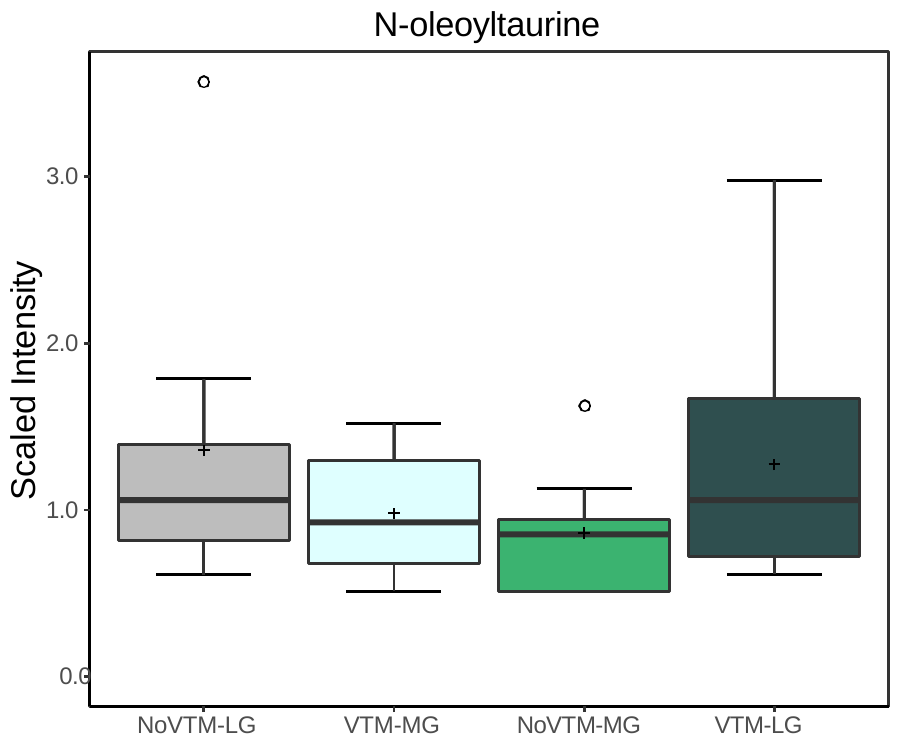

N-oleoyltaurine
3.0
Scaled Intensity
2.0
1.0
0.0
NoVTM-LG
VTM-MG
NoVTM-MG
VTM-LG

## Slide 154
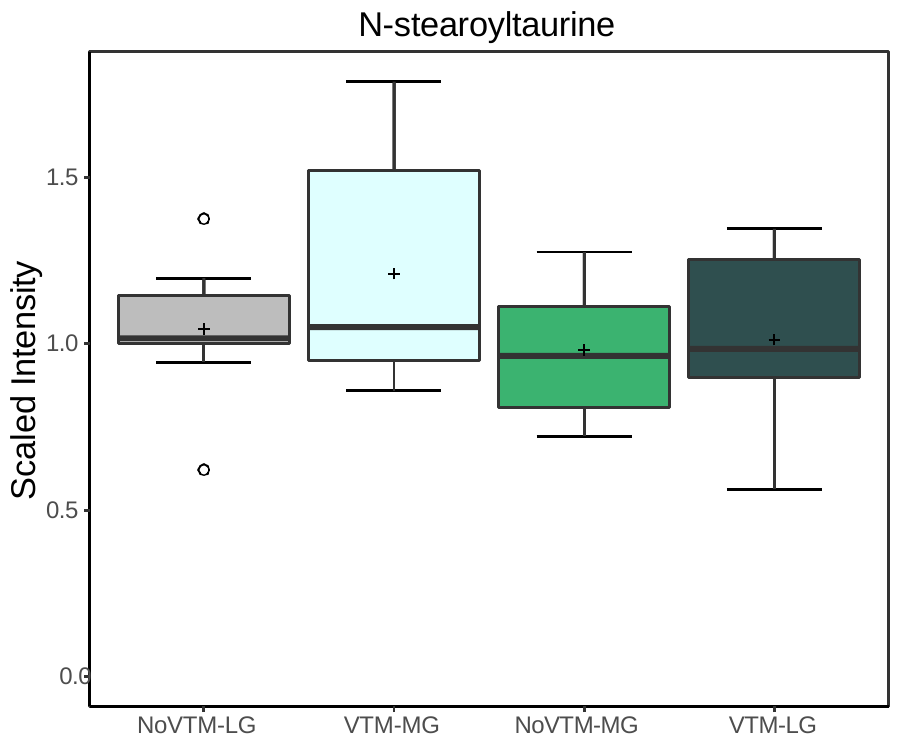

N-stearoyltaurine
1.5
Scaled Intensity
1.0
0.5
0.0
NoVTM-LG
VTM-MG
NoVTM-MG
VTM-LG

## Slide 155
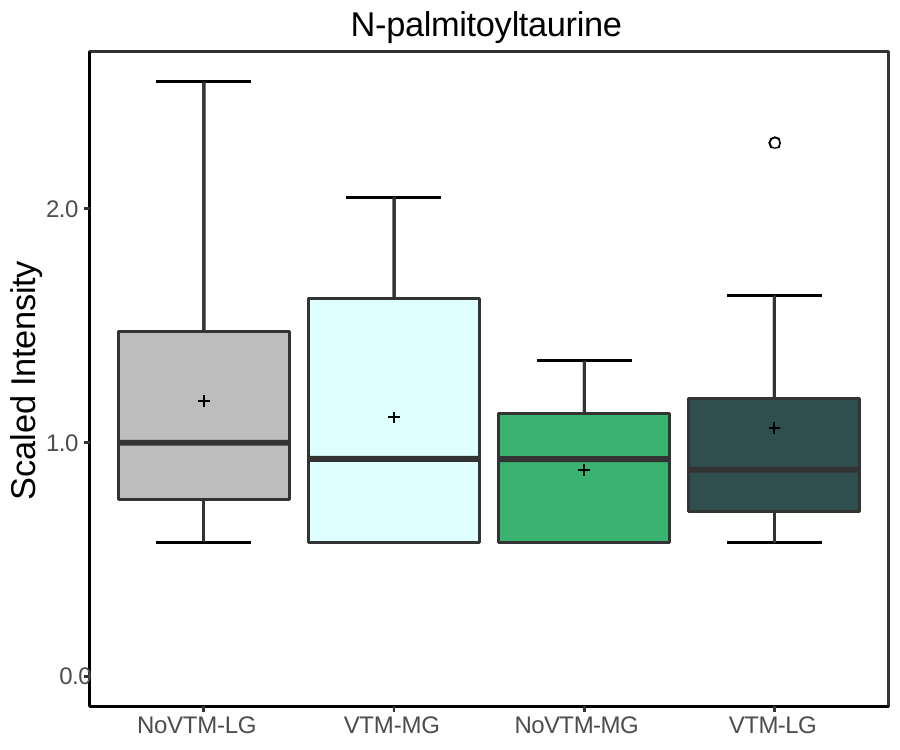

N-palmitoyltaurine
2.0
Scaled Intensity
1.0
0.0
NoVTM-LG
VTM-MG
NoVTM-MG
VTM-LG

## Slide 156
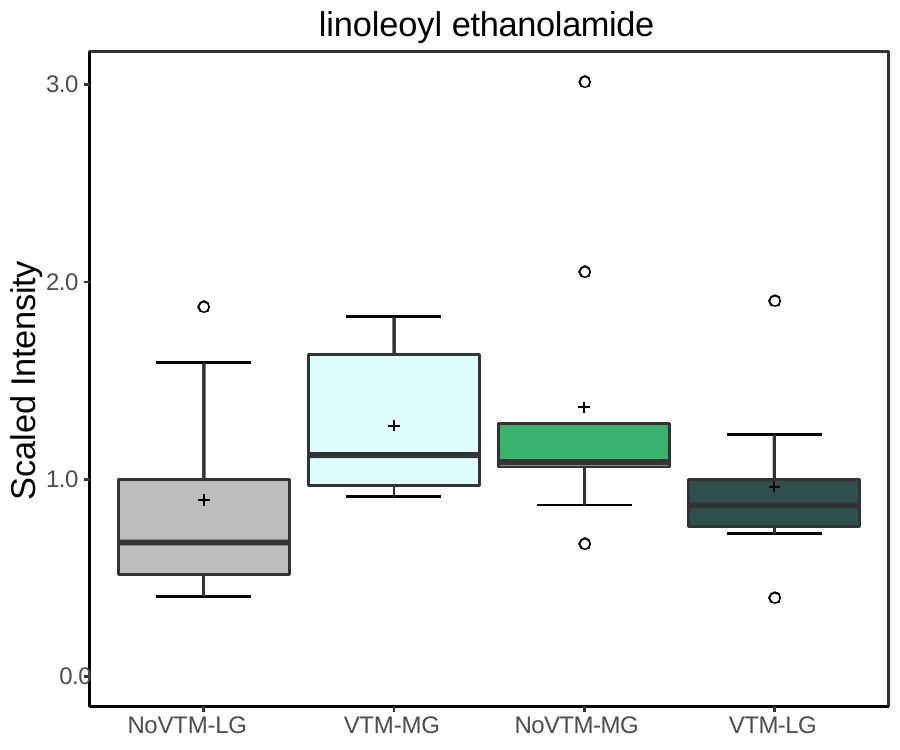

linoleoyl ethanolamide
3.0
Scaled Intensity
2.0
1.0
0.0
NoVTM-LG
VTM-MG
NoVTM-MG
VTM-LG

## Slide 157
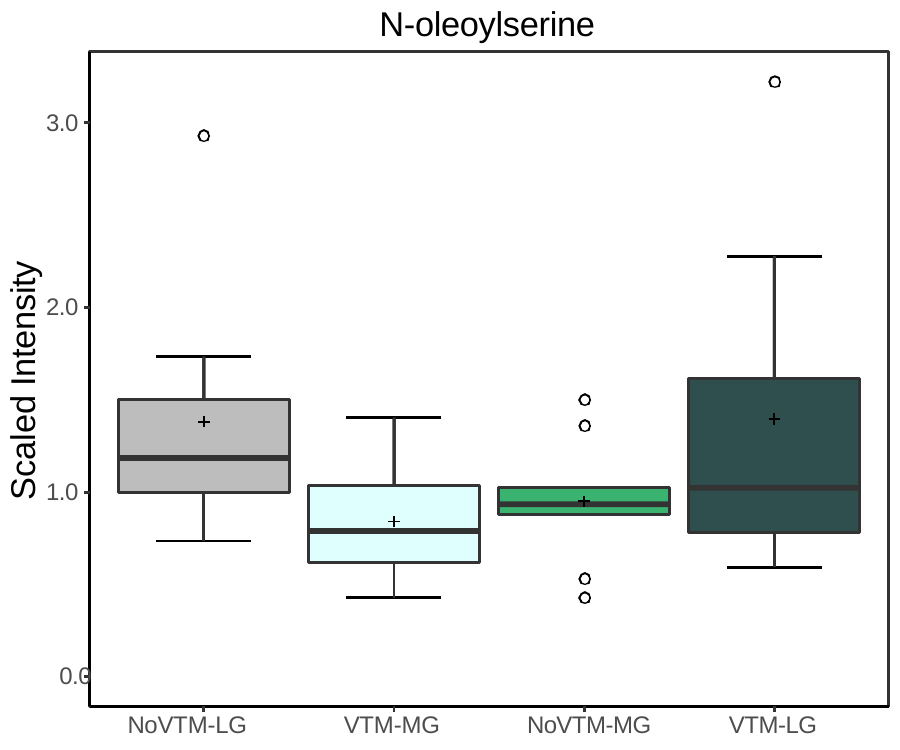

N-oleoylserine
3.0
Scaled Intensity
2.0
1.0
0.0
NoVTM-LG
VTM-MG
NoVTM-MG
VTM-LG

## Slide 158
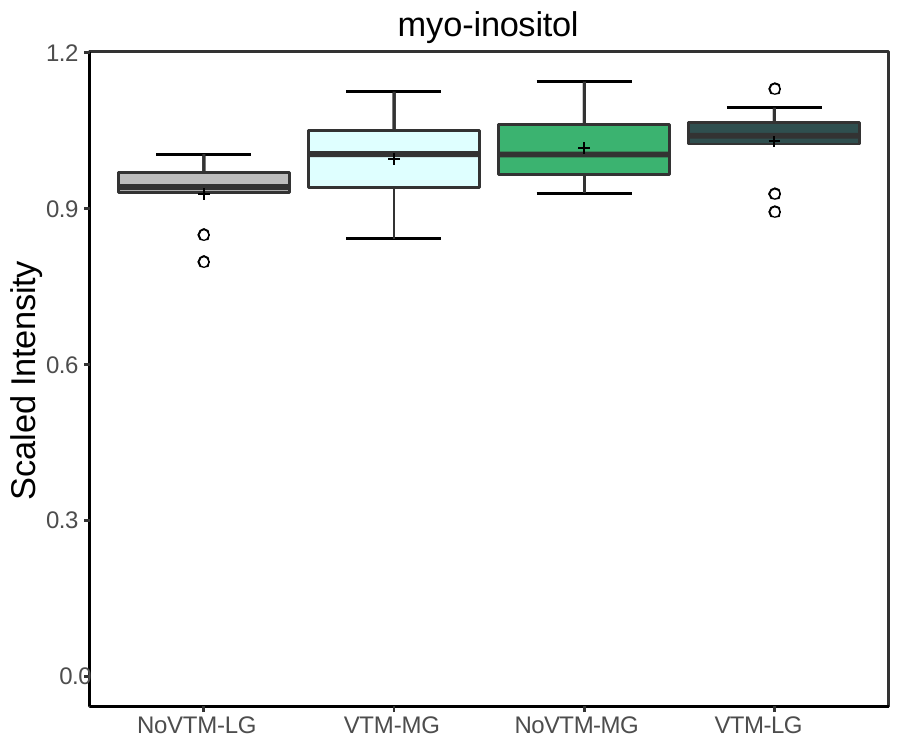

myo-inositol
1.2
0.9
Scaled Intensity
0.6
0.3
0.0
NoVTM-LG
VTM-MG
NoVTM-MG
VTM-LG

## Slide 159
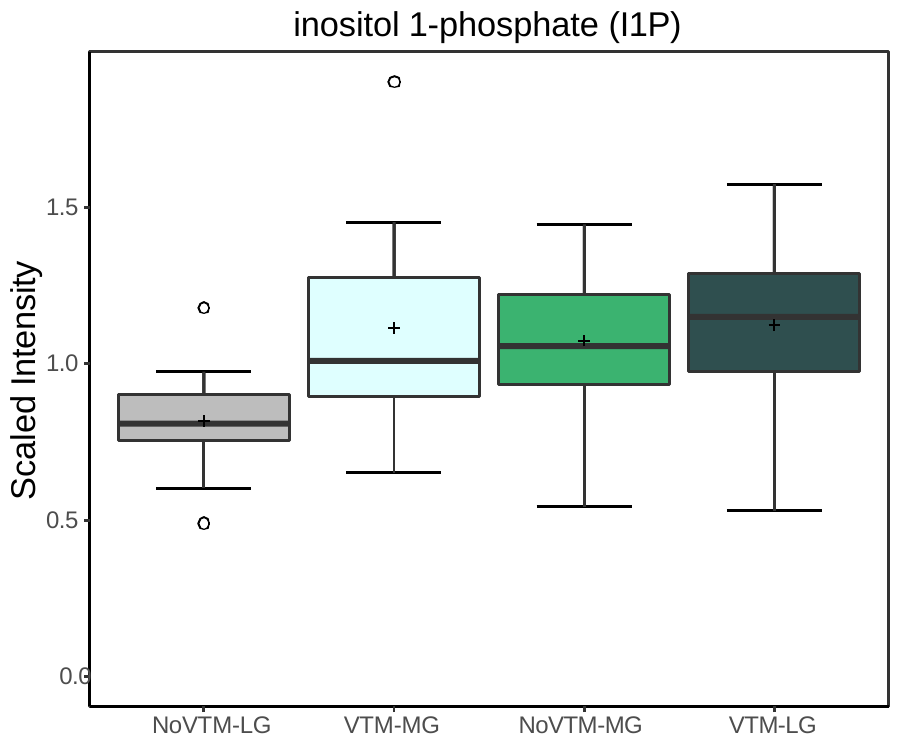

inositol 1-phosphate (I1P)
1.5
Scaled Intensity
1.0
0.5
0.0
NoVTM-LG
VTM-MG
NoVTM-MG
VTM-LG

## Slide 160
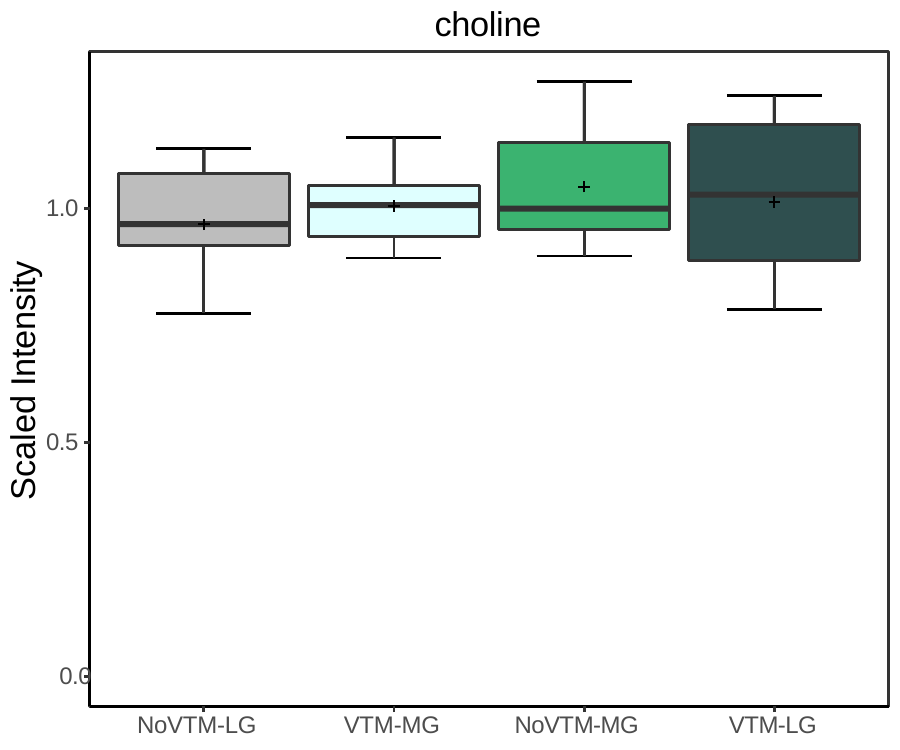

choline
1.0
Scaled Intensity
0.5
0.0
NoVTM-LG
VTM-MG
NoVTM-MG
VTM-LG

## Slide 161
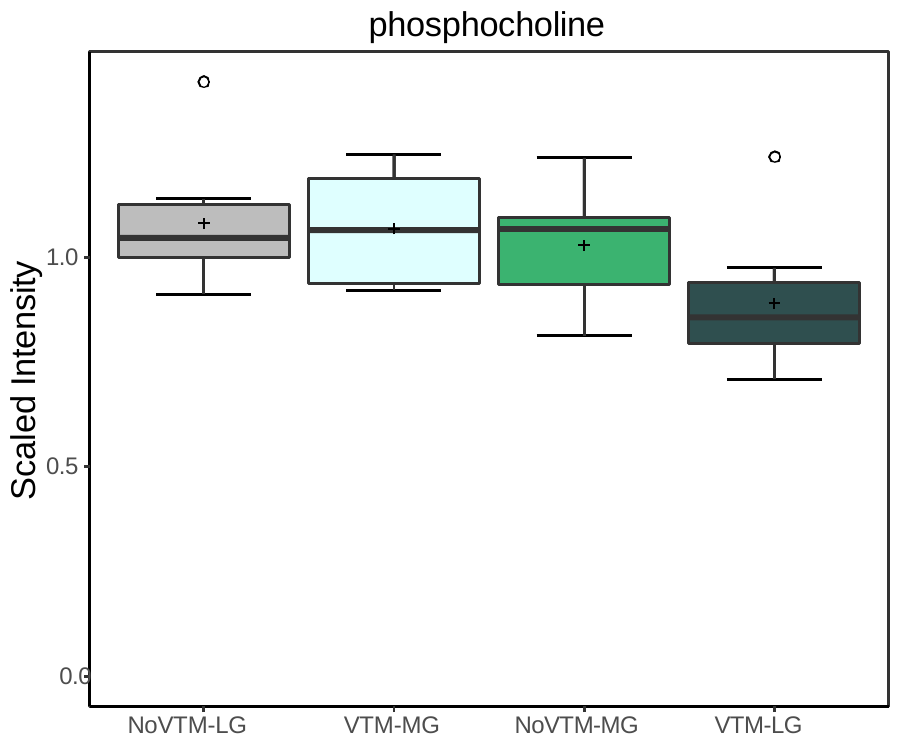

phosphocholine
1.0
Scaled Intensity
0.5
0.0
NoVTM-LG
VTM-MG
NoVTM-MG
VTM-LG

## Slide 162
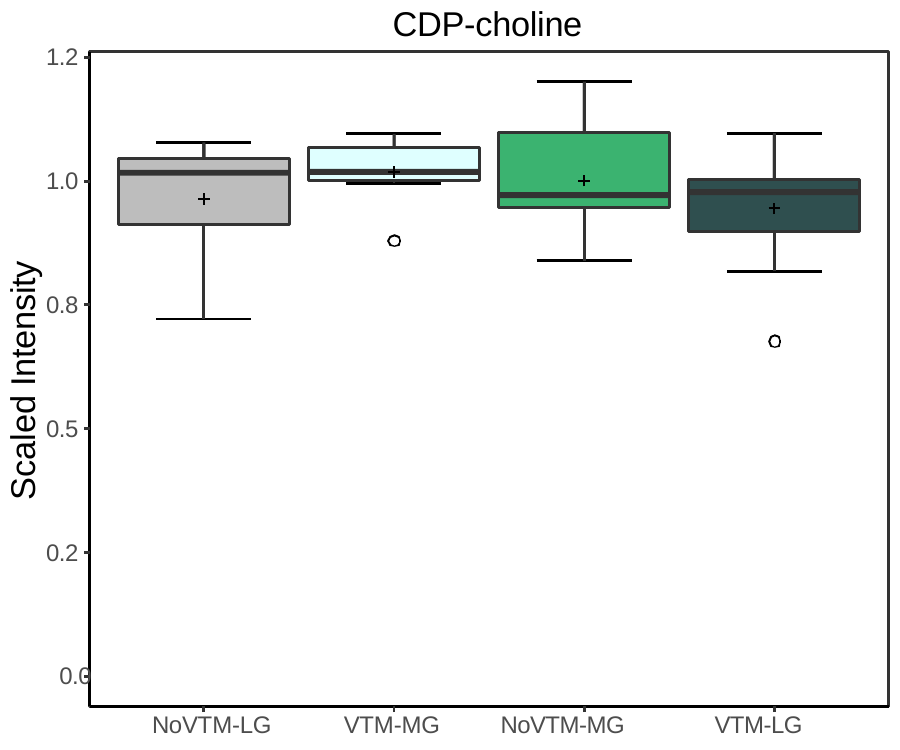

CDP-choline
1.2
1.0
Scaled Intensity
0.8
0.5
0.2
0.0
NoVTM-LG
VTM-MG
NoVTM-MG
VTM-LG

## Slide 163
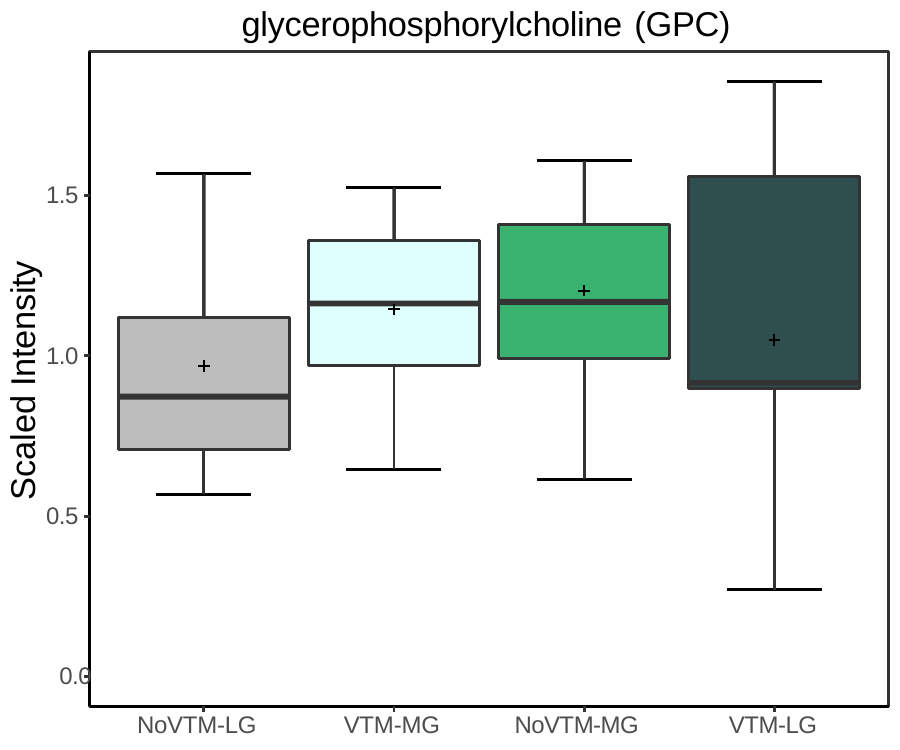

glycerophosphorylcholine (GPC)
1.5
Scaled Intensity
1.0
0.5
0.0
NoVTM-LG
VTM-MG
NoVTM-MG
VTM-LG

## Slide 164
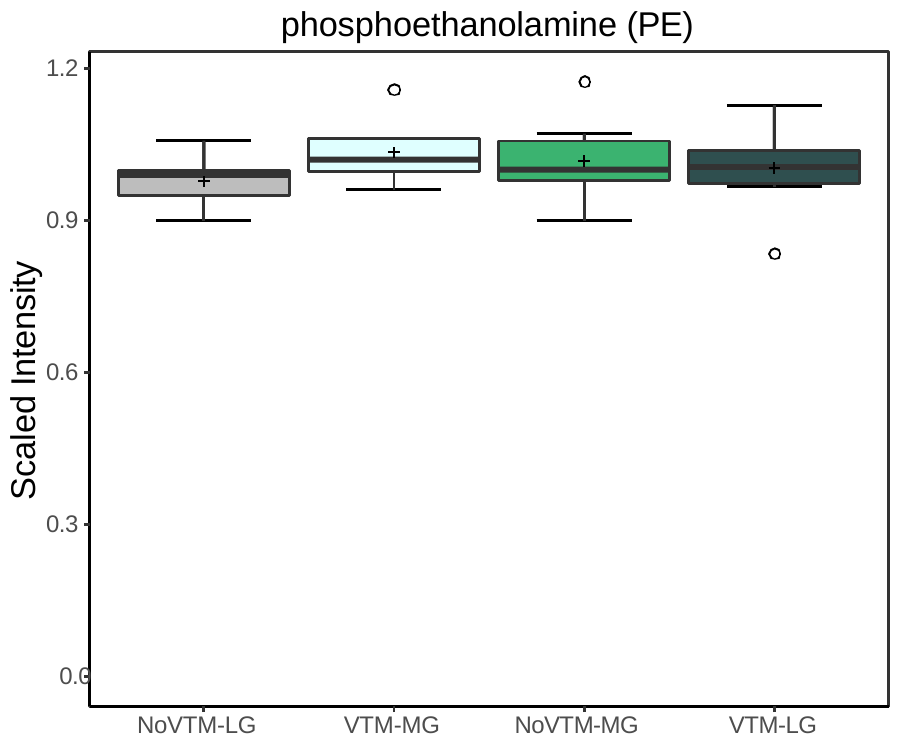

phosphoethanolamine (PE)
1.2
0.9
Scaled Intensity
0.6
0.3
0.0
NoVTM-LG
VTM-MG
NoVTM-MG
VTM-LG

## Slide 165
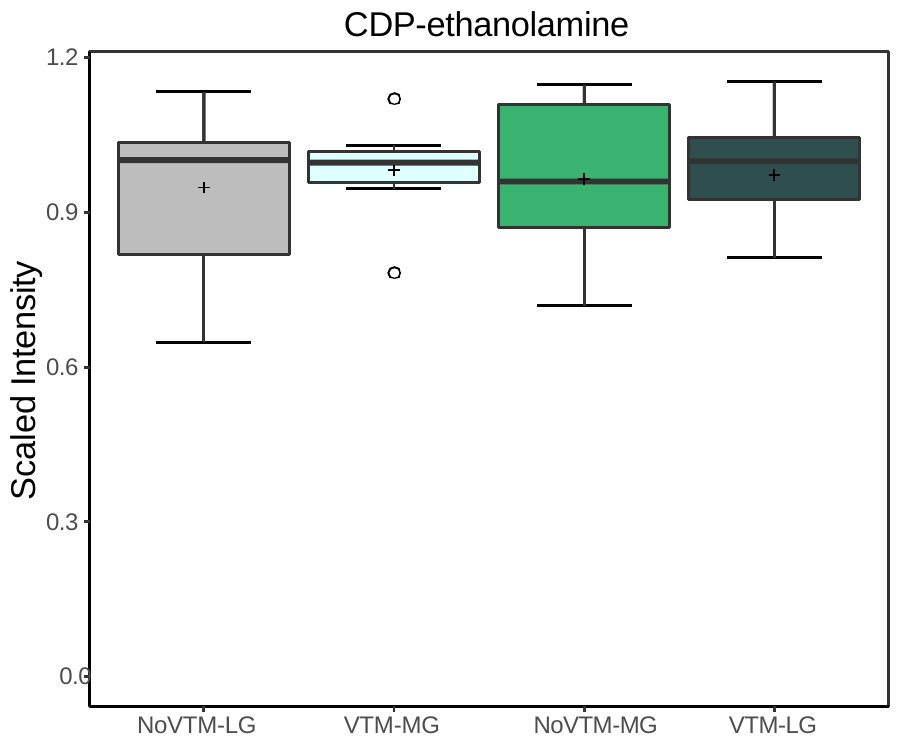

CDP-ethanolamine
1.2
0.9
Scaled Intensity
0.6
0.3
0.0
NoVTM-LG
VTM-MG
NoVTM-MG
VTM-LG

## Slide 166
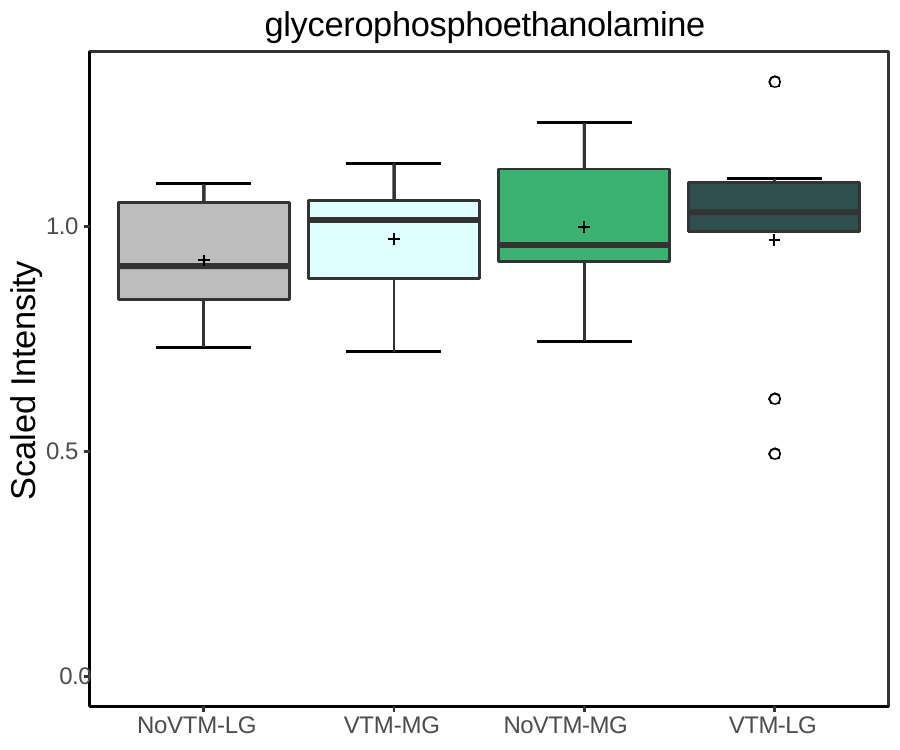

glycerophosphoethanolamine
1.0
Scaled Intensity
0.5
0.0
NoVTM-LG
VTM-MG
NoVTM-MG
VTM-LG

## Slide 167
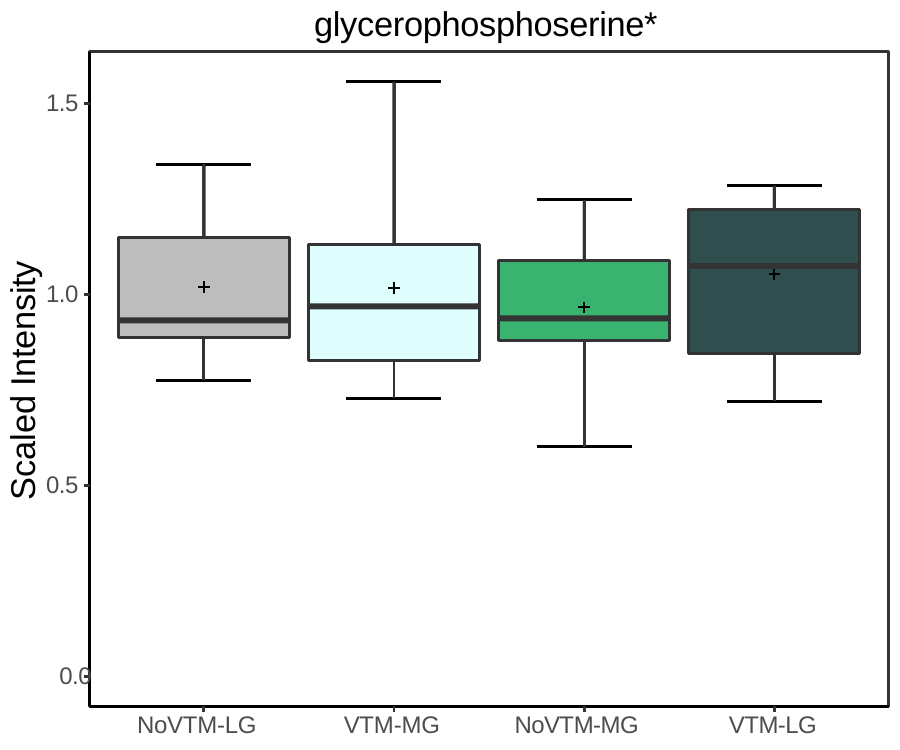

glycerophosphoserine*
1.5
Scaled Intensity
1.0
0.5
0.0
NoVTM-LG
VTM-MG
NoVTM-MG
VTM-LG

## Slide 168
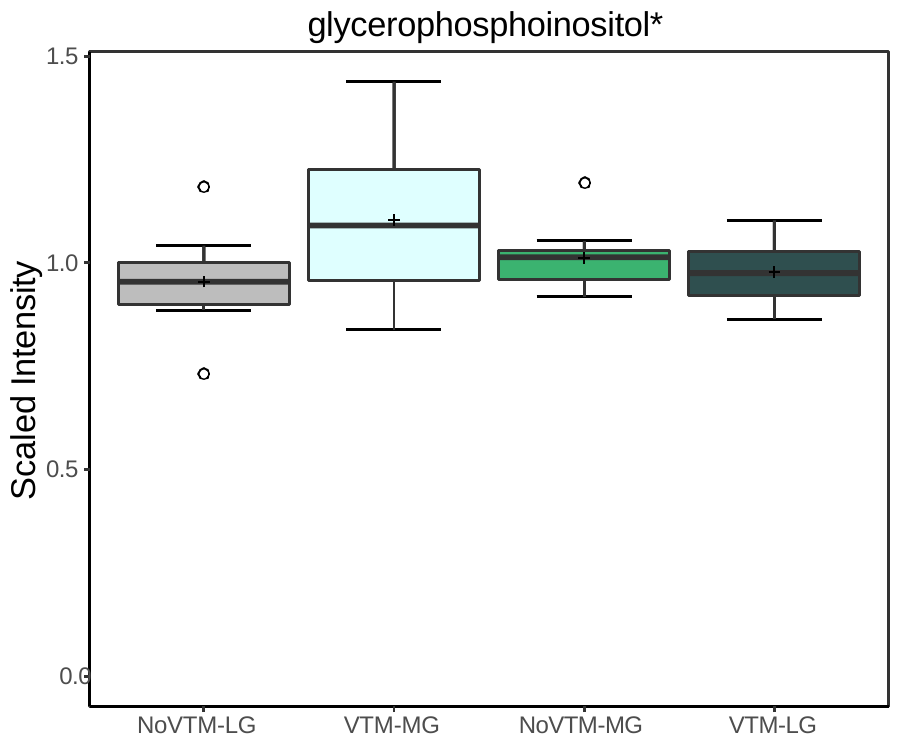

glycerophosphoinositol*
1.5
1.0
Scaled Intensity
0.5
0.0
NoVTM-LG
VTM-MG
NoVTM-MG
VTM-LG

## Slide 169
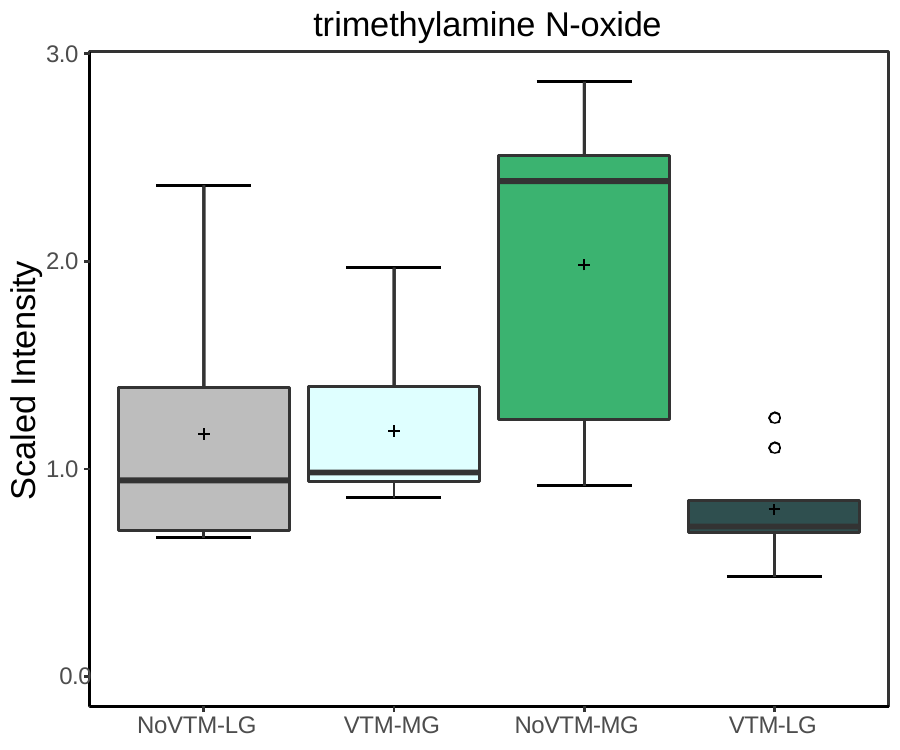

trimethylamine N-oxide
3.0
2.0
Scaled Intensity
1.0
0.0
NoVTM-LG
VTM-MG
NoVTM-MG
VTM-LG

## Slide 170
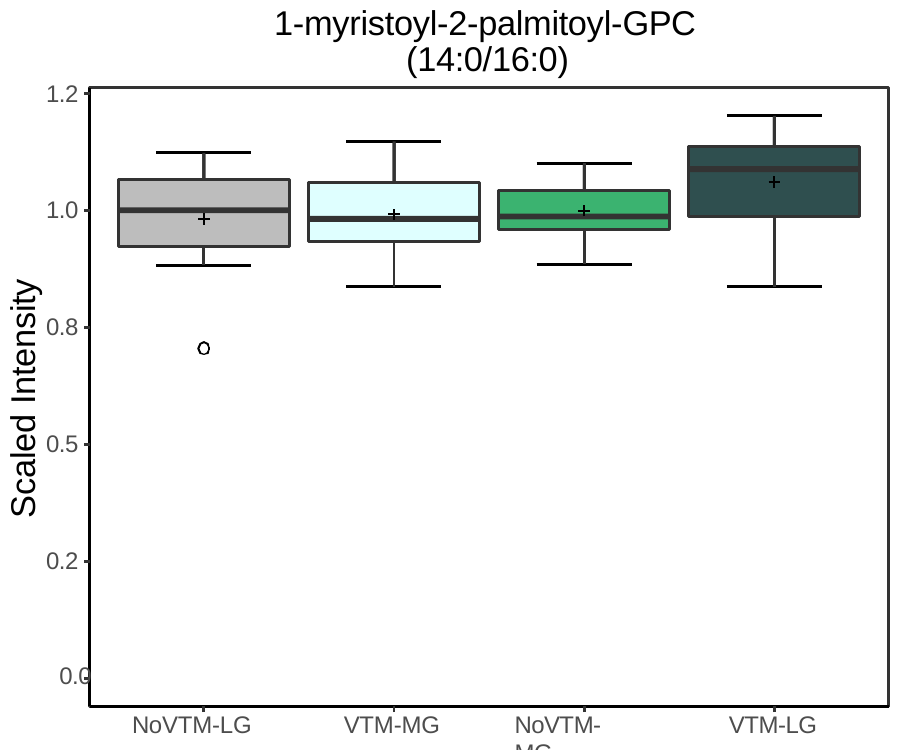

# 1-myristoyl-2-palmitoyl-GPC (14:0/16:0)
1.2
1.0
Scaled Intensity
0.8
0.5
0.2
0.0
NoVTM-LG
VTM-MG
NoVTM-MG
VTM-LG

## Slide 171
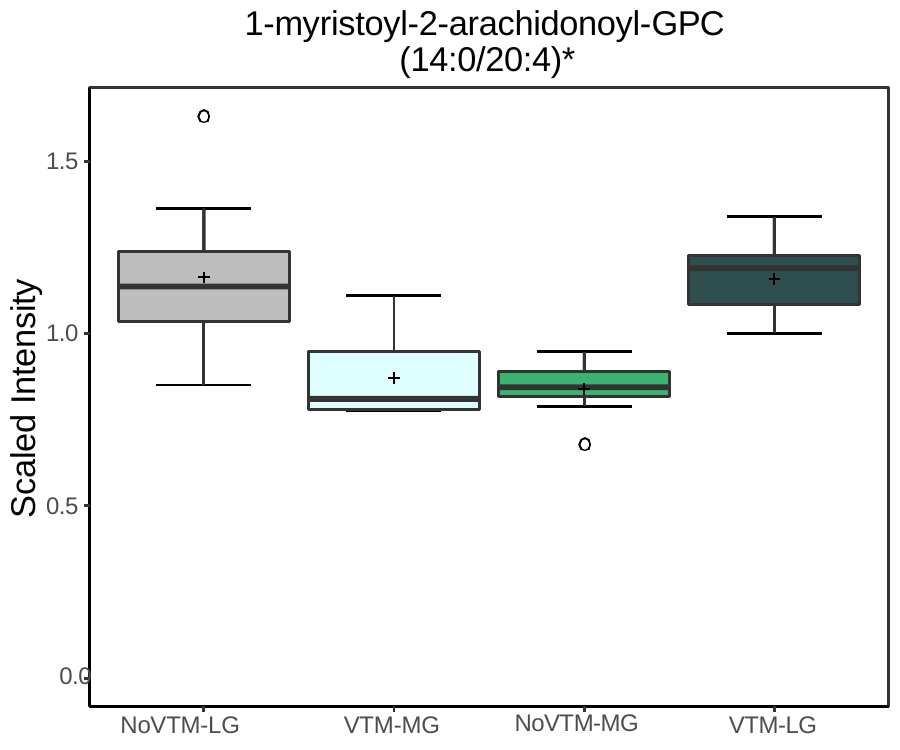

# 1-myristoyl-2-arachidonoyl-GPC (14:0/20:4)*
1.5
Scaled Intensity
1.0
0.5
0.0
NoVTM-MG
NoVTM-LG
VTM-MG
VTM-LG

## Slide 172
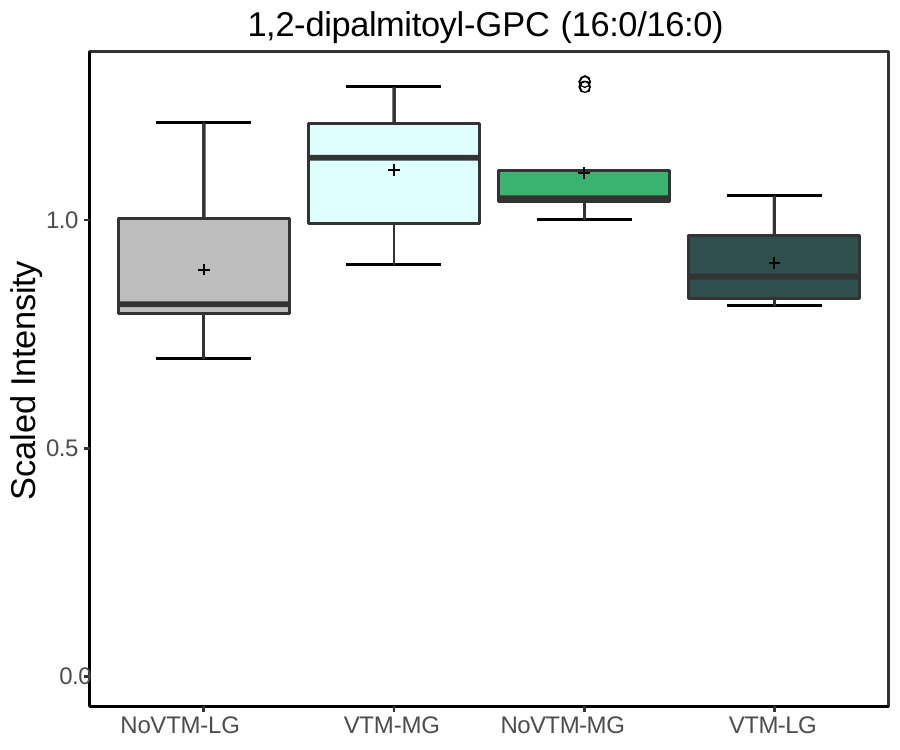

1,2-dipalmitoyl-GPC (16:0/16:0)
1.0
Scaled Intensity
0.5
0.0
NoVTM-LG
VTM-MG
NoVTM-MG
VTM-LG

## Slide 173
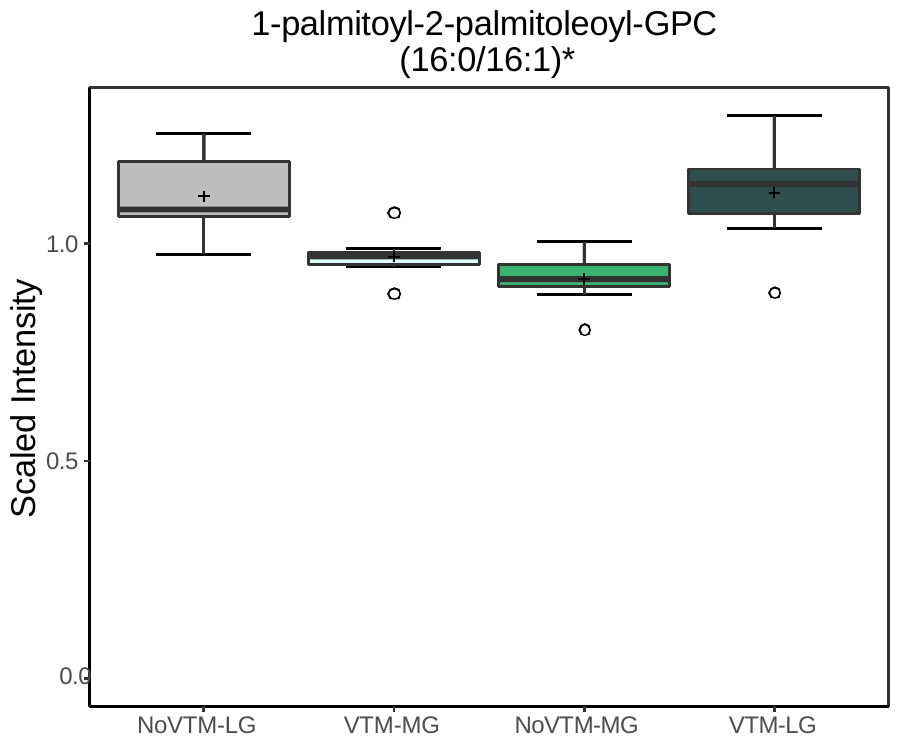

# 1-palmitoyl-2-palmitoleoyl-GPC (16:0/16:1)*
1.0
Scaled Intensity
0.5
0.0
NoVTM-LG
VTM-MG
NoVTM-MG
VTM-LG

## Slide 174
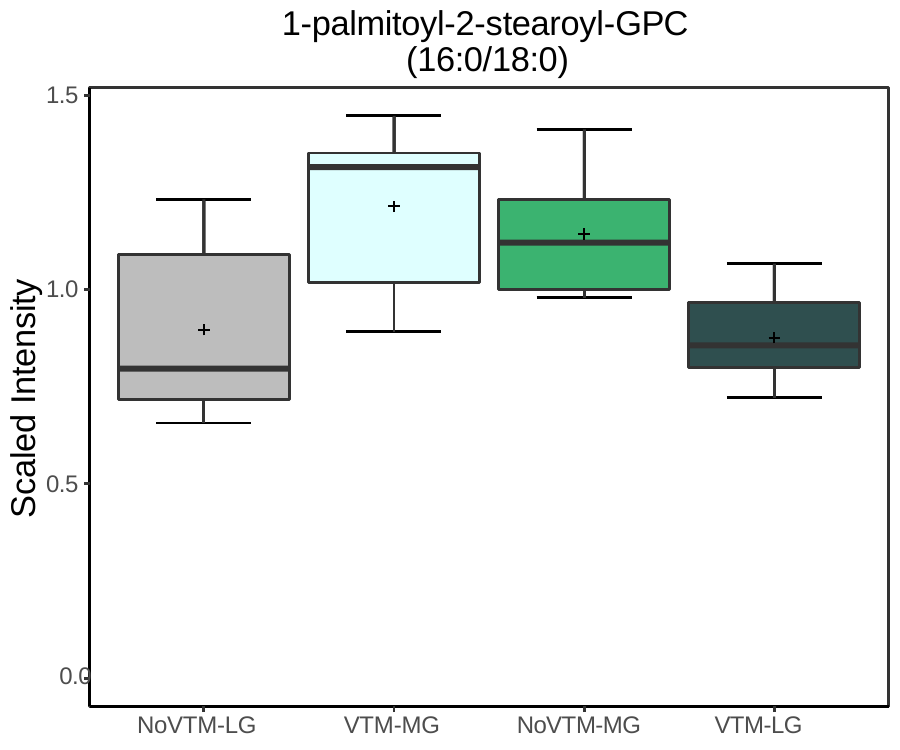

# 1-palmitoyl-2-stearoyl-GPC (16:0/18:0)
1.5
1.0
Scaled Intensity
0.5
0.0
NoVTM-LG
VTM-MG
NoVTM-MG
VTM-LG

## Slide 175
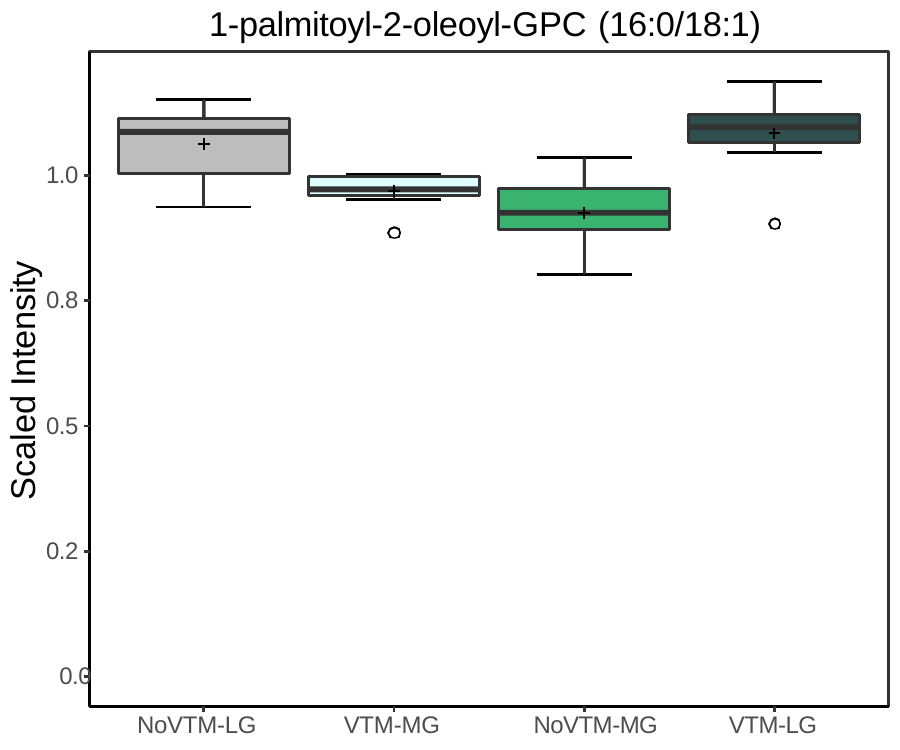

1-palmitoyl-2-oleoyl-GPC (16:0/18:1)
1.0
Scaled Intensity
0.8
0.5
0.2
0.0
NoVTM-LG
VTM-MG
NoVTM-MG
VTM-LG

## Slide 176
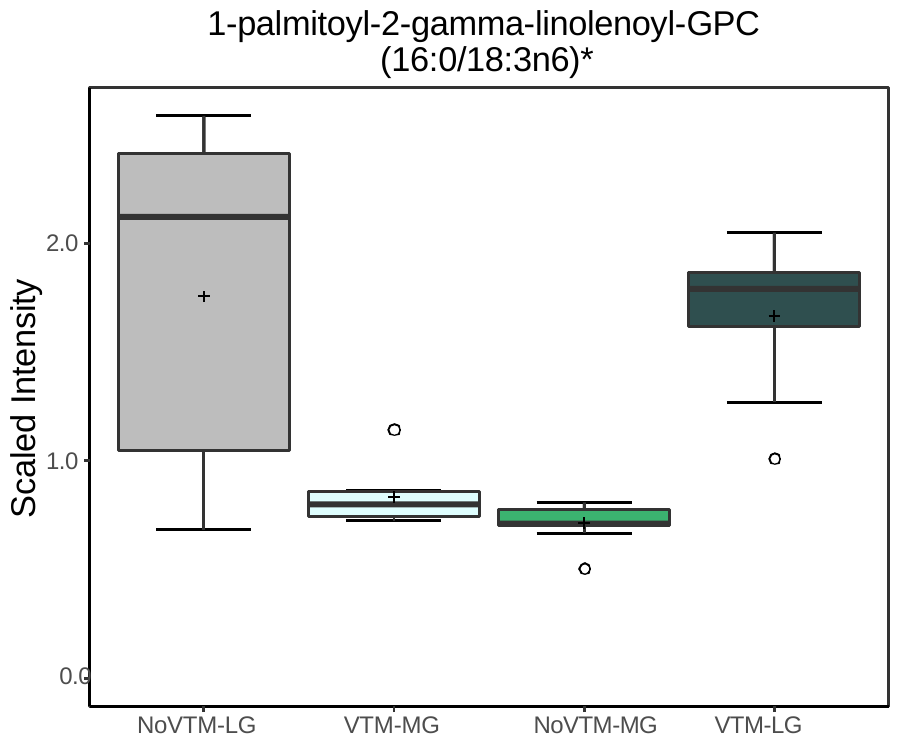

# 1-palmitoyl-2-gamma-linolenoyl-GPC (16:0/18:3n6)*
2.0
Scaled Intensity
1.0
0.0
NoVTM-LG
VTM-MG
NoVTM-MG
VTM-LG

## Slide 177
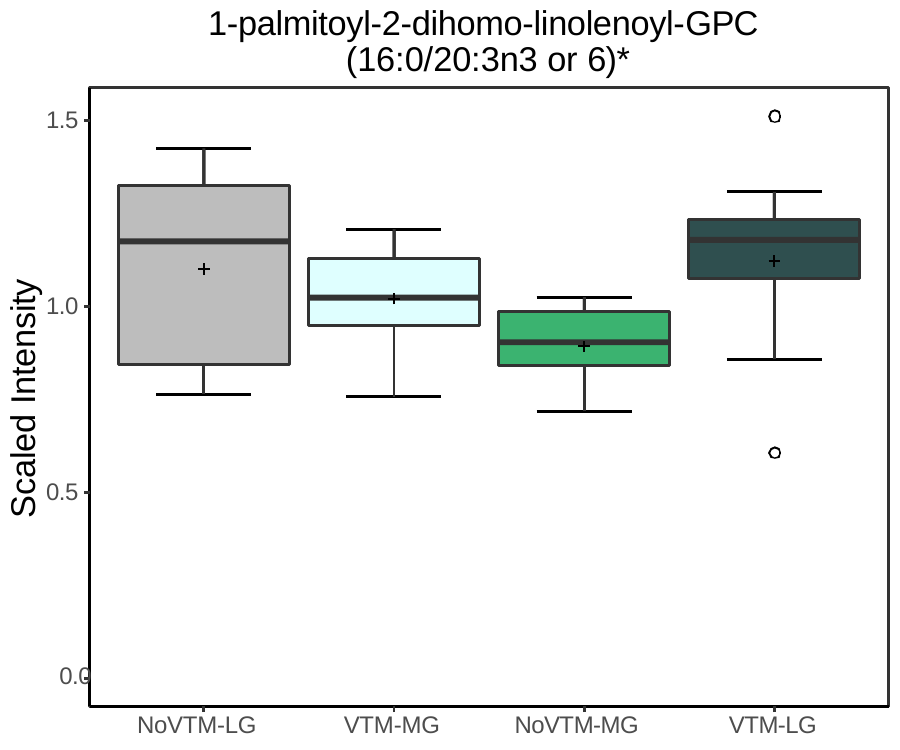

# 1-palmitoyl-2-dihomo-linolenoyl-GPC (16:0/20:3n3 or 6)*
1.5
Scaled Intensity
1.0
0.5
0.0
NoVTM-LG
VTM-MG
NoVTM-MG
VTM-LG

## Slide 178
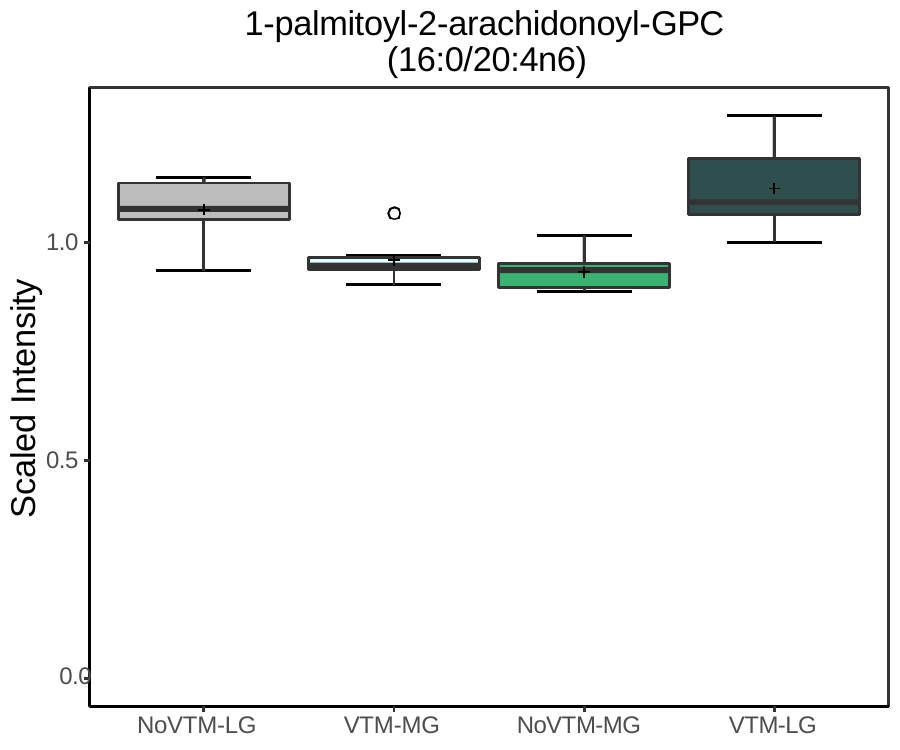

# 1-palmitoyl-2-arachidonoyl-GPC (16:0/20:4n6)
1.0
Scaled Intensity
0.5
0.0
NoVTM-LG
VTM-MG
NoVTM-MG
VTM-LG

## Slide 179
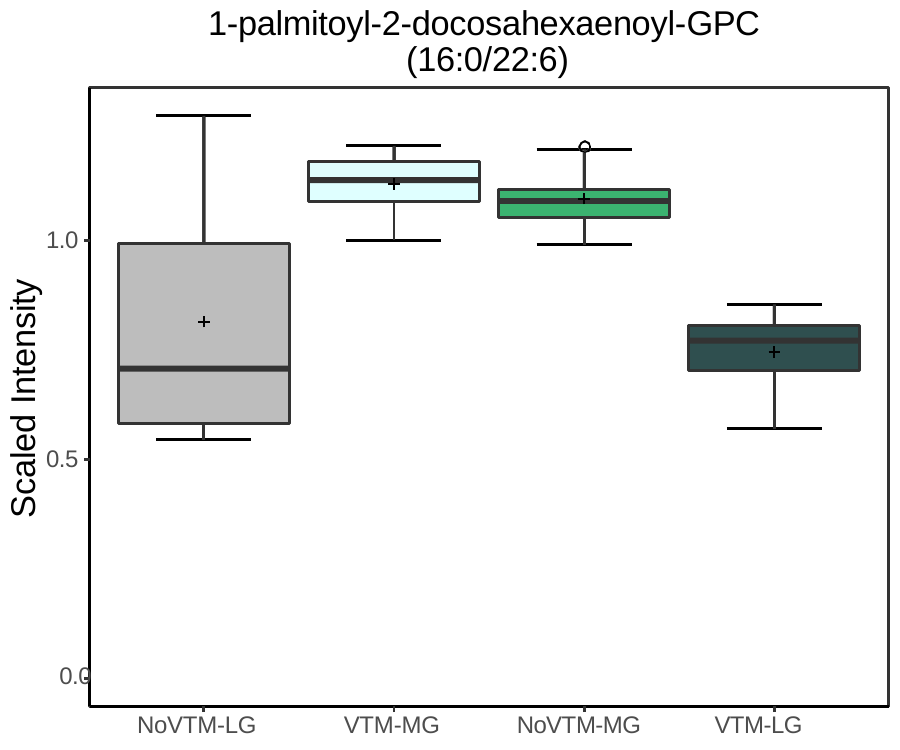

# 1-palmitoyl-2-docosahexaenoyl-GPC (16:0/22:6)
1.0
Scaled Intensity
0.5
0.0
NoVTM-LG
VTM-MG
NoVTM-MG
VTM-LG

## Slide 180
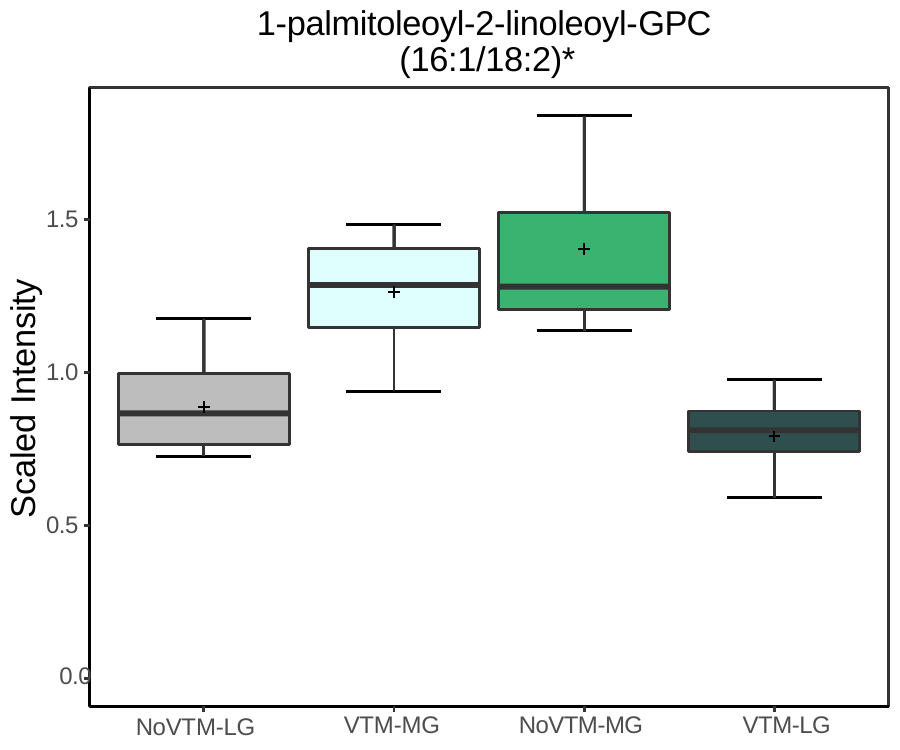

# 1-palmitoleoyl-2-linoleoyl-GPC (16:1/18:2)*
1.5
Scaled Intensity
1.0
0.5
0.0
VTM-LG
VTM-MG
NoVTM-MG
NoVTM-LG

## Slide 181
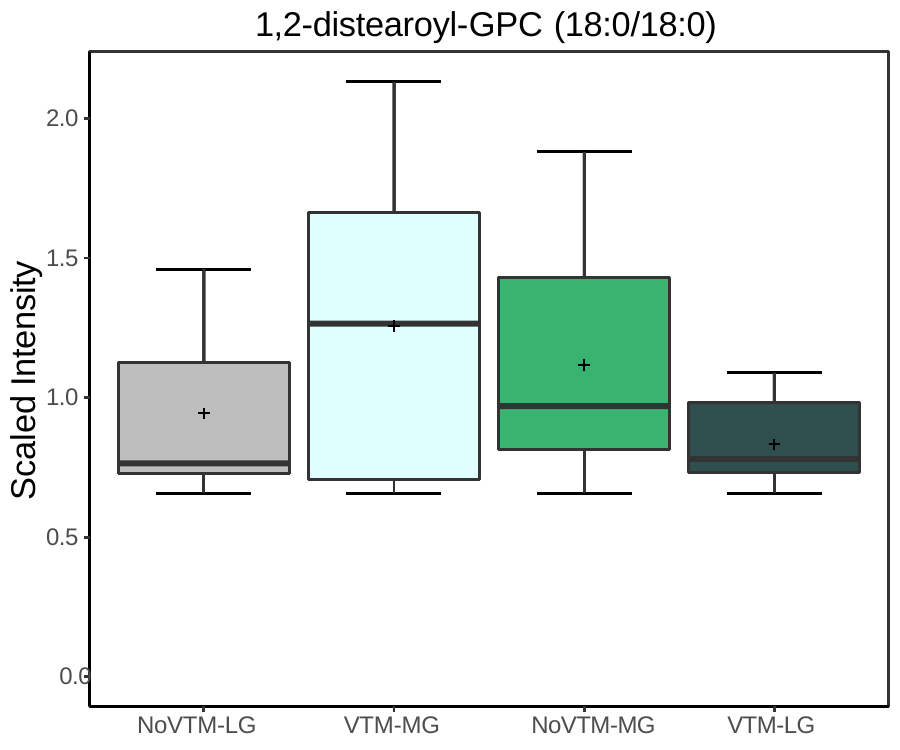

1,2-distearoyl-GPC (18:0/18:0)
2.0
1.5
Scaled Intensity
1.0
0.5
0.0
NoVTM-LG
VTM-MG
NoVTM-MG
VTM-LG

## Slide 182
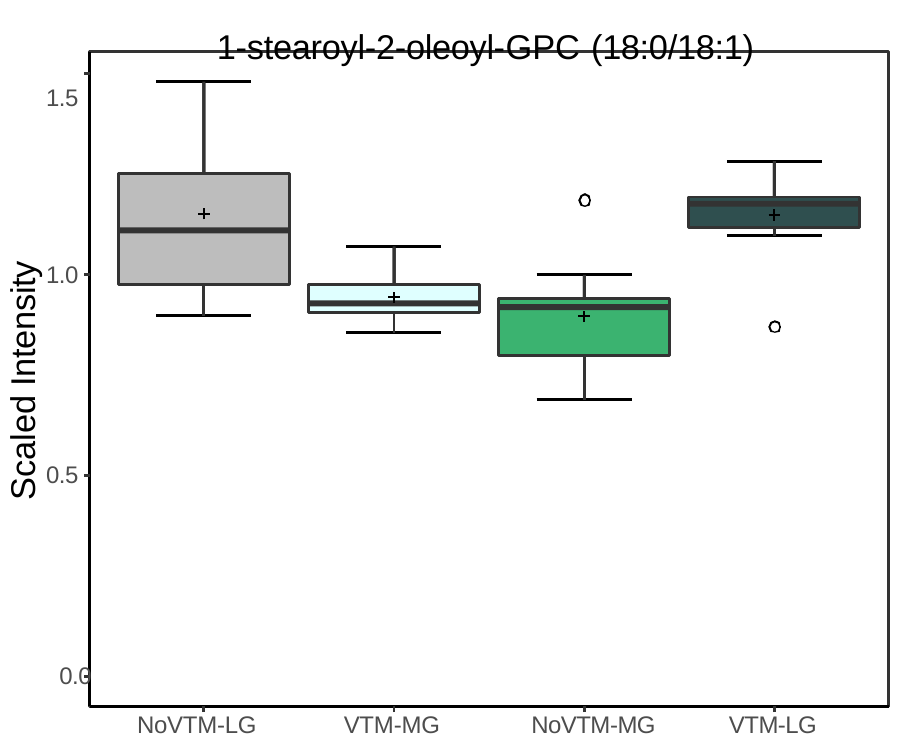

1-stearoyl-2-oleoyl-GPC (18:0/18:1)
1.5
Scaled Intensity
1.0
0.5
0.0
NoVTM-LG
VTM-MG
NoVTM-MG
VTM-LG

## Slide 183
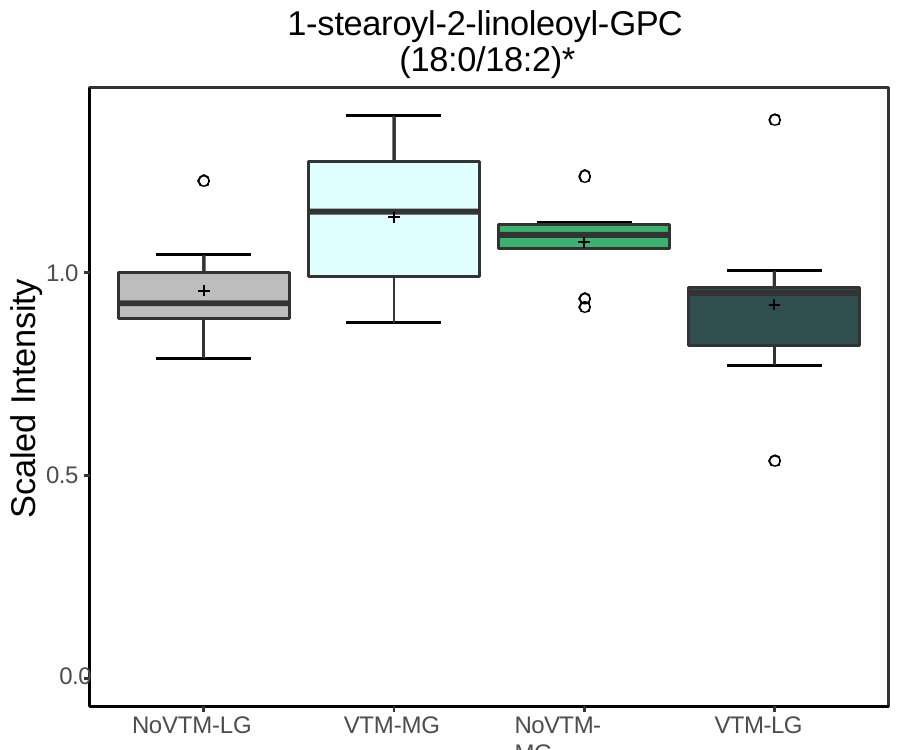

# 1-stearoyl-2-linoleoyl-GPC (18:0/18:2)*
1.0
Scaled Intensity
0.5
0.0
NoVTM-LG
VTM-MG
NoVTM-MG
VTM-LG

## Slide 184
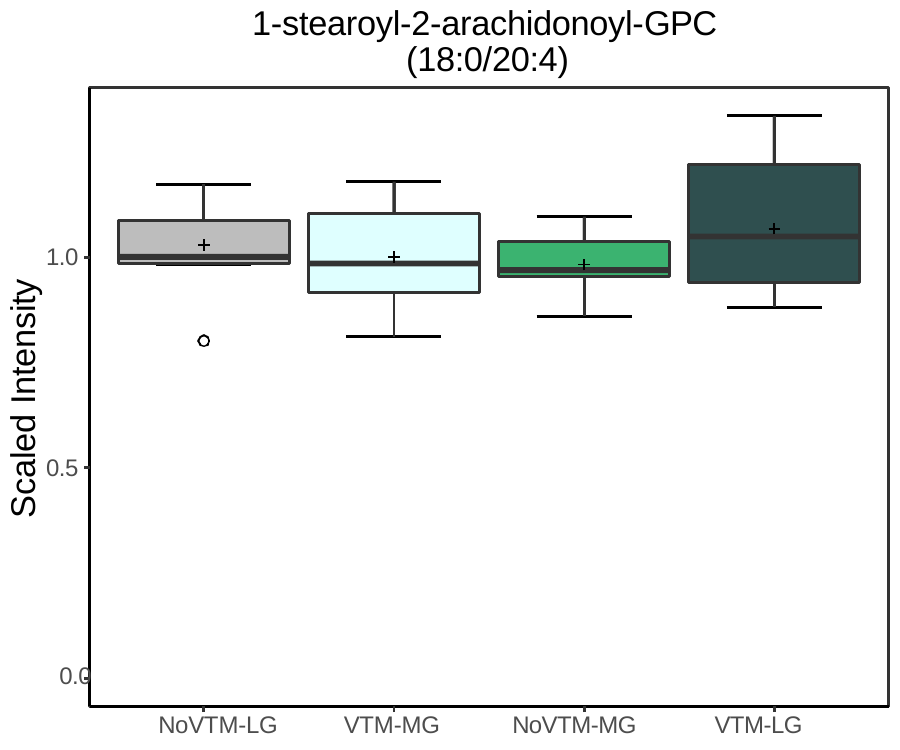

# 1-stearoyl-2-arachidonoyl-GPC (18:0/20:4)
1.0
Scaled Intensity
0.5
0.0
NoVTM-LG
VTM-MG
NoVTM-MG
VTM-LG

## Slide 185
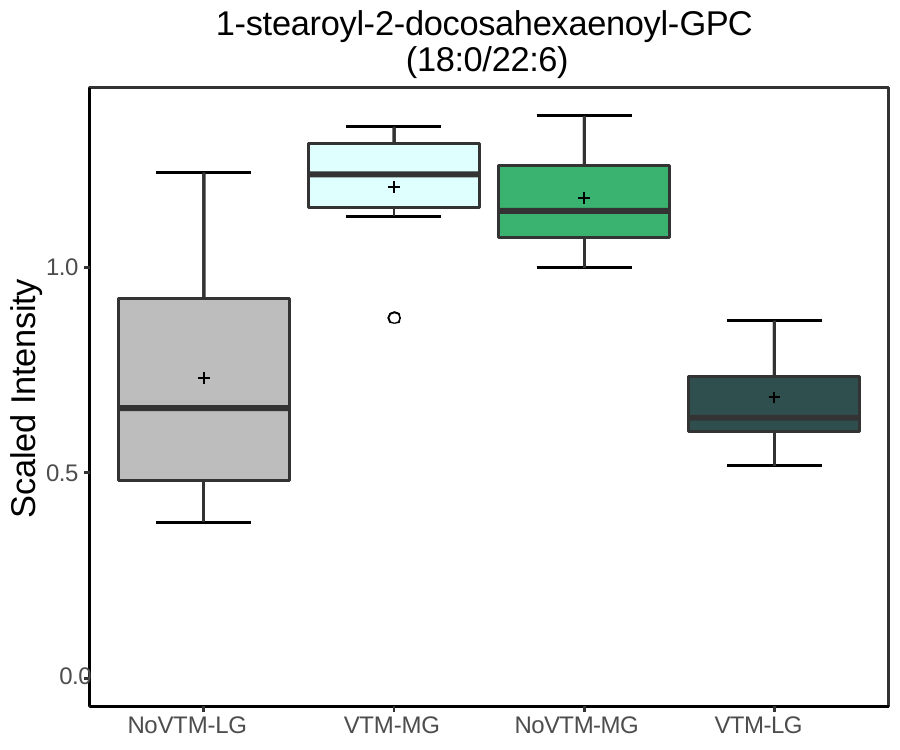

# 1-stearoyl-2-docosahexaenoyl-GPC (18:0/22:6)
1.0
Scaled Intensity
0.5
0.0
NoVTM-LG
VTM-MG
NoVTM-MG
VTM-LG

## Slide 186
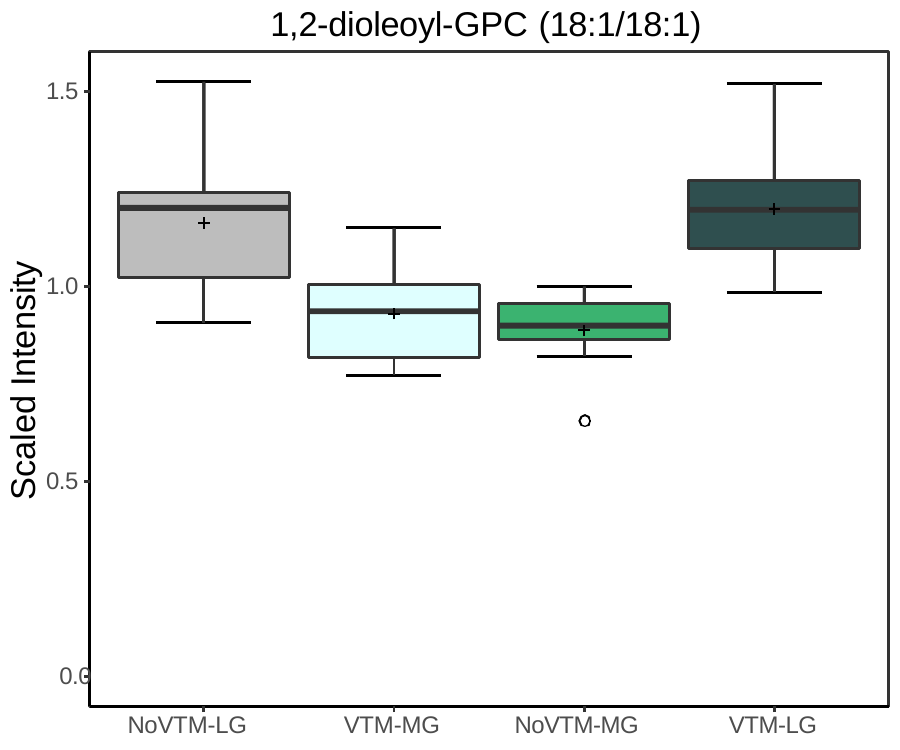

1,2-dioleoyl-GPC (18:1/18:1)
1.5
Scaled Intensity
1.0
0.5
0.0
NoVTM-LG
VTM-MG
NoVTM-MG
VTM-LG

## Slide 187
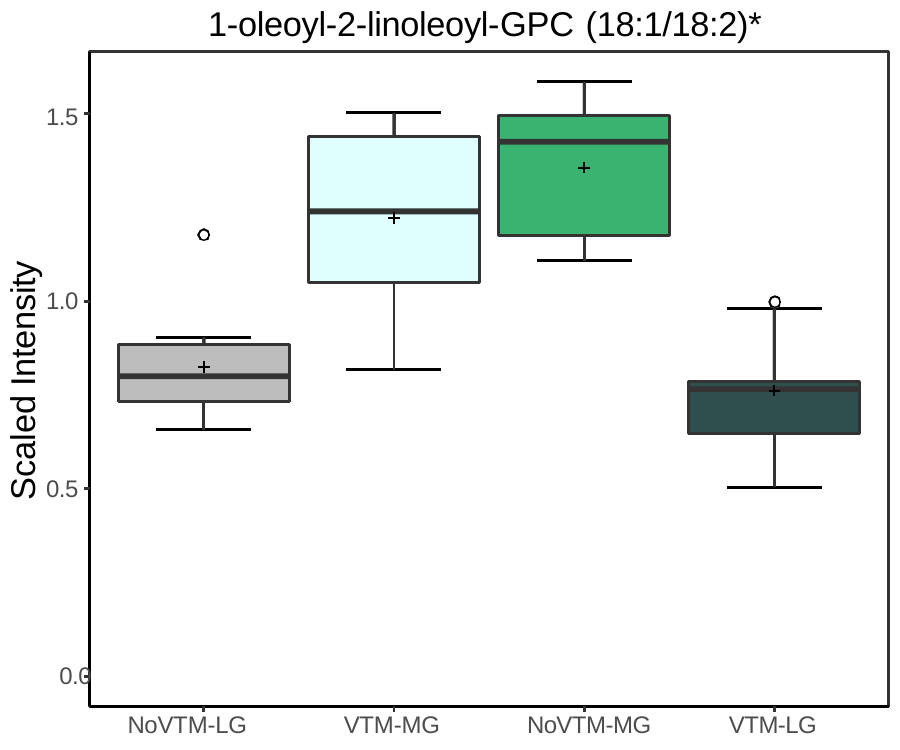

1-oleoyl-2-linoleoyl-GPC (18:1/18:2)*
1.5
Scaled Intensity
1.0
0.5
0.0
NoVTM-LG
VTM-MG
NoVTM-MG
VTM-LG

## Slide 188
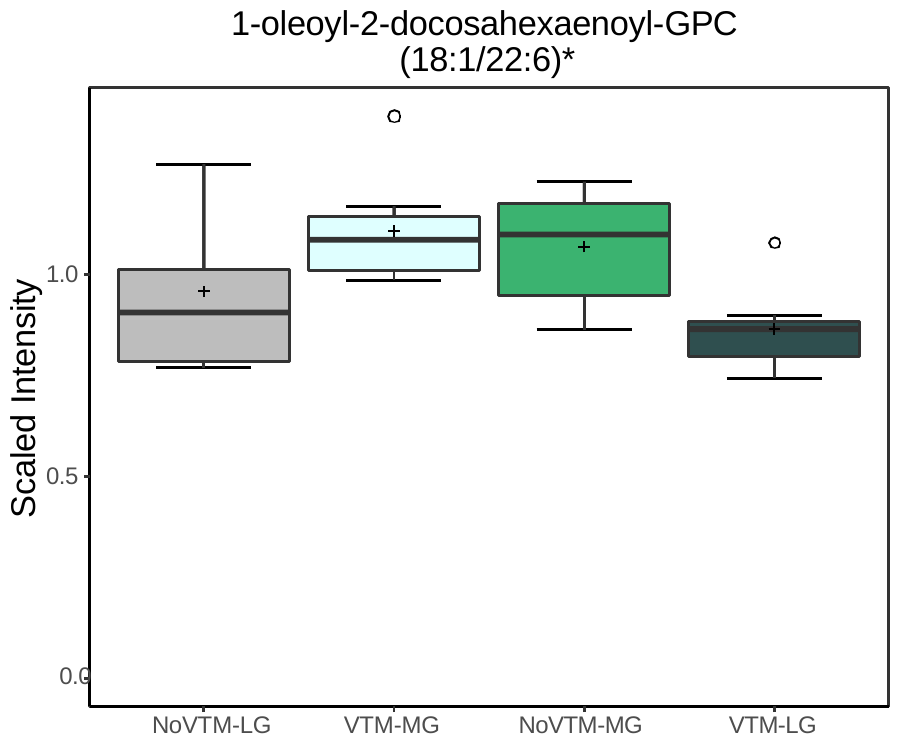

# 1-oleoyl-2-docosahexaenoyl-GPC (18:1/22:6)*
1.0
Scaled Intensity
0.5
0.0
NoVTM-LG
VTM-MG
NoVTM-MG
VTM-LG

## Slide 189
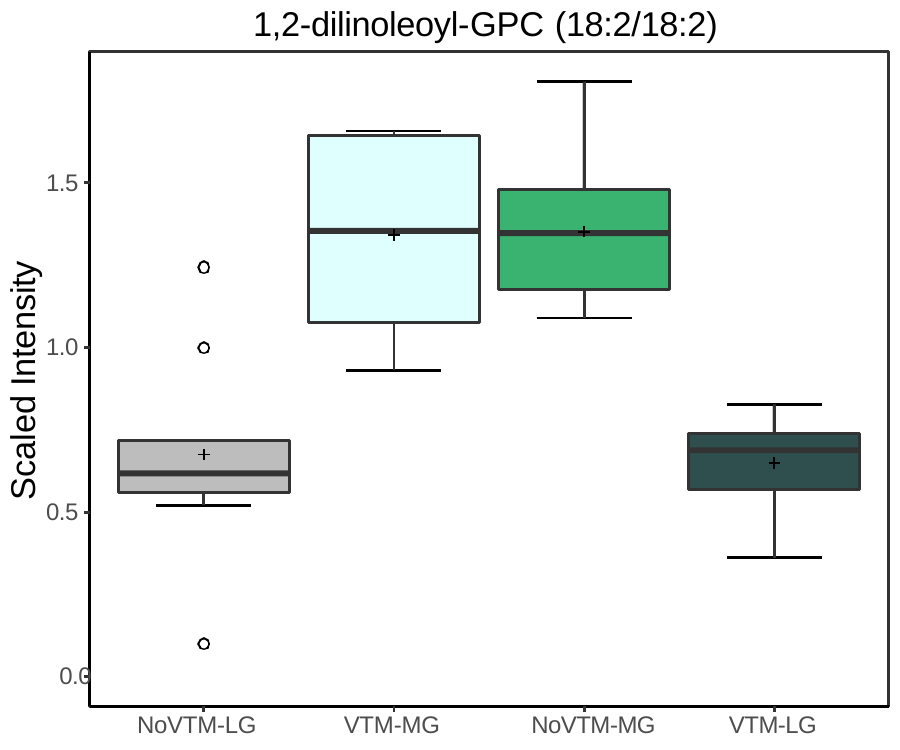

1,2-dilinoleoyl-GPC (18:2/18:2)
1.5
Scaled Intensity
1.0
0.5
0.0
NoVTM-LG
VTM-MG
NoVTM-MG
VTM-LG

## Slide 190
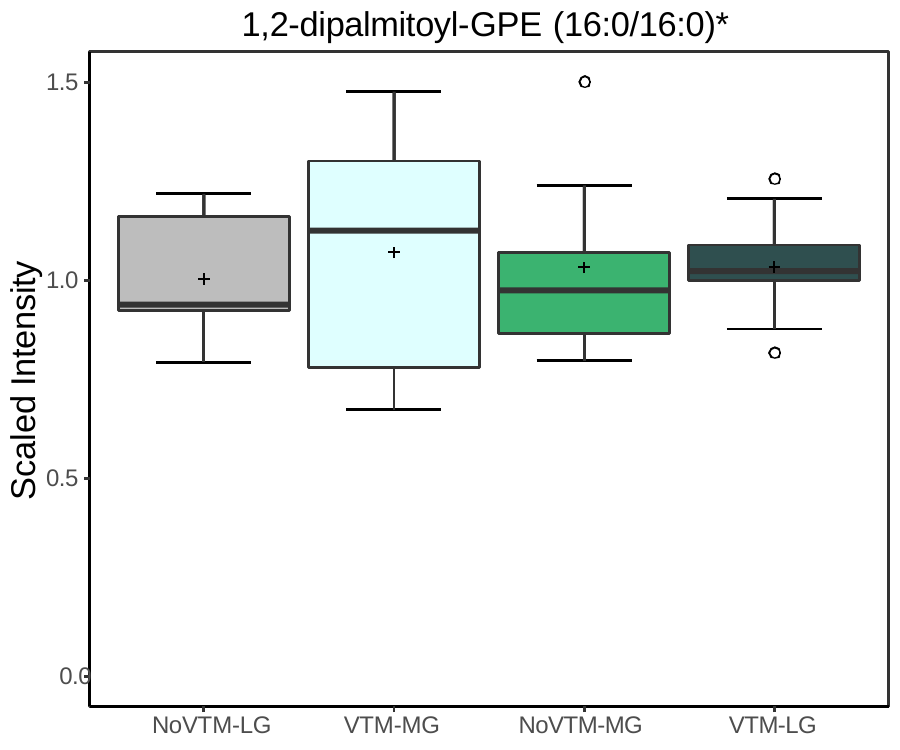

1,2-dipalmitoyl-GPE (16:0/16:0)*
1.5
Scaled Intensity
1.0
0.5
0.0
NoVTM-LG
VTM-MG
NoVTM-MG
VTM-LG

## Slide 191
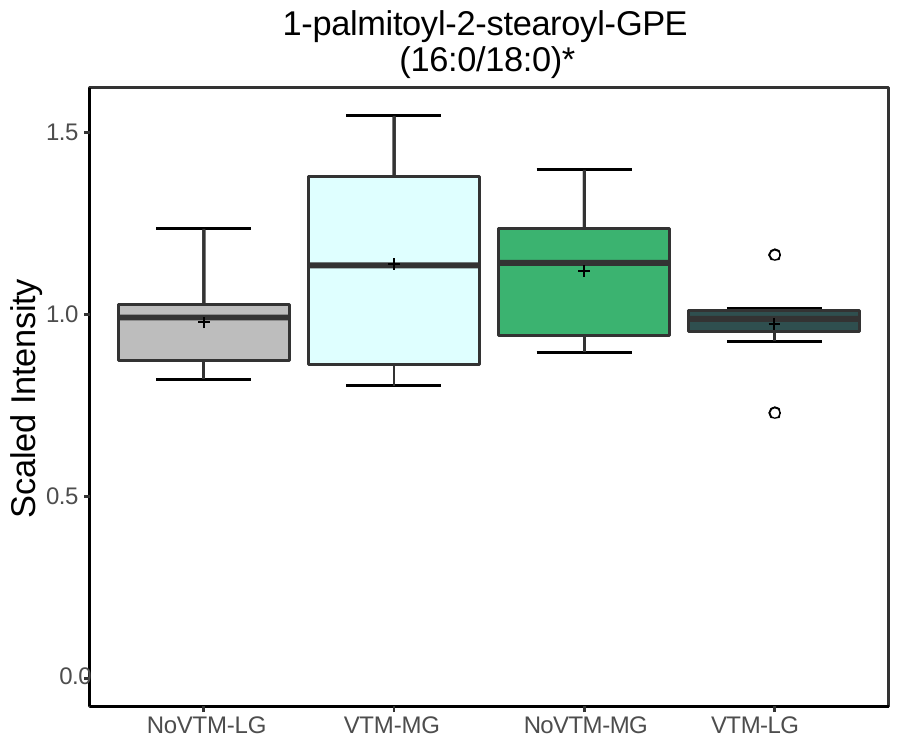

# 1-palmitoyl-2-stearoyl-GPE (16:0/18:0)*
1.5
Scaled Intensity
1.0
0.5
0.0
NoVTM-LG
VTM-MG
NoVTM-MG
VTM-LG

## Slide 192
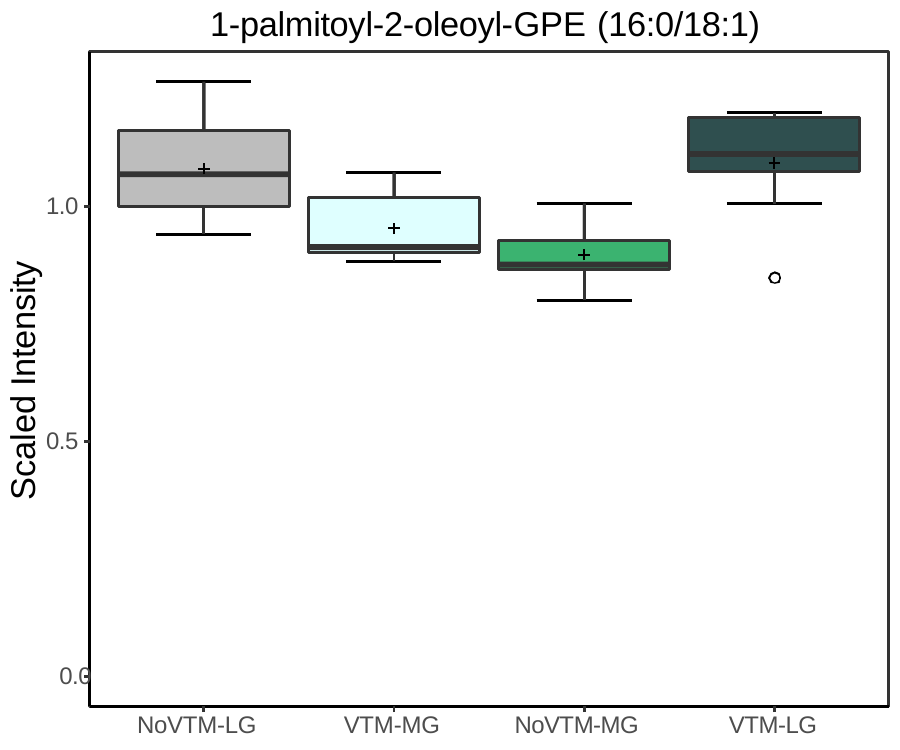

1-palmitoyl-2-oleoyl-GPE (16:0/18:1)
1.0
Scaled Intensity
0.5
0.0
NoVTM-LG
VTM-MG
NoVTM-MG
VTM-LG

## Slide 193
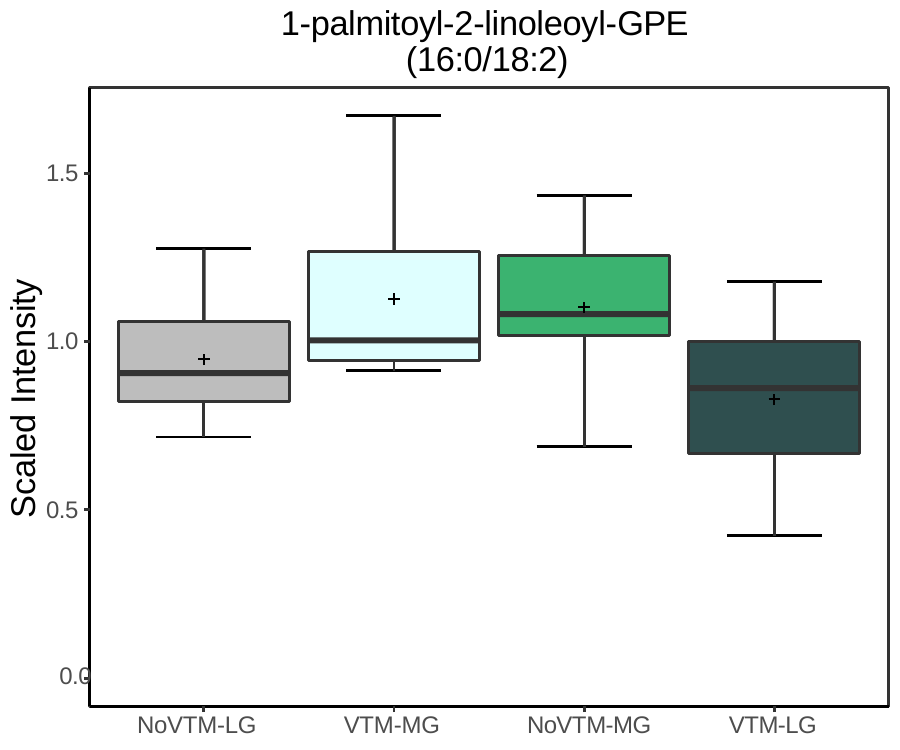

# 1-palmitoyl-2-linoleoyl-GPE (16:0/18:2)
1.5
Scaled Intensity
1.0
0.5
0.0
NoVTM-LG
VTM-MG
NoVTM-MG
VTM-LG

## Slide 194
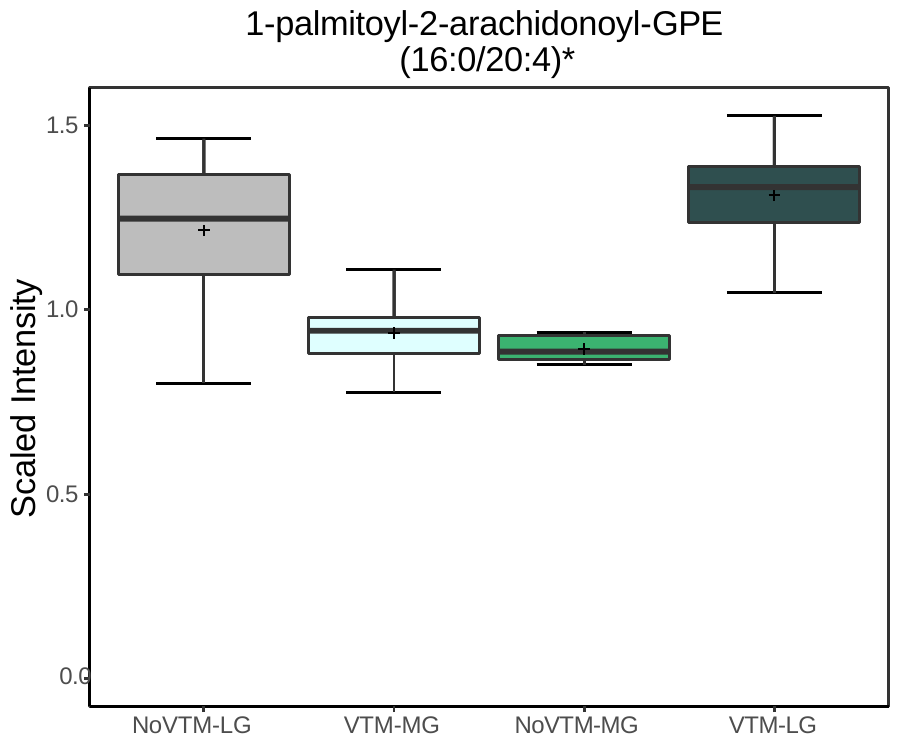

# 1-palmitoyl-2-arachidonoyl-GPE (16:0/20:4)*
1.5
Scaled Intensity
1.0
0.5
0.0
NoVTM-LG
VTM-MG
NoVTM-MG
VTM-LG

## Slide 195
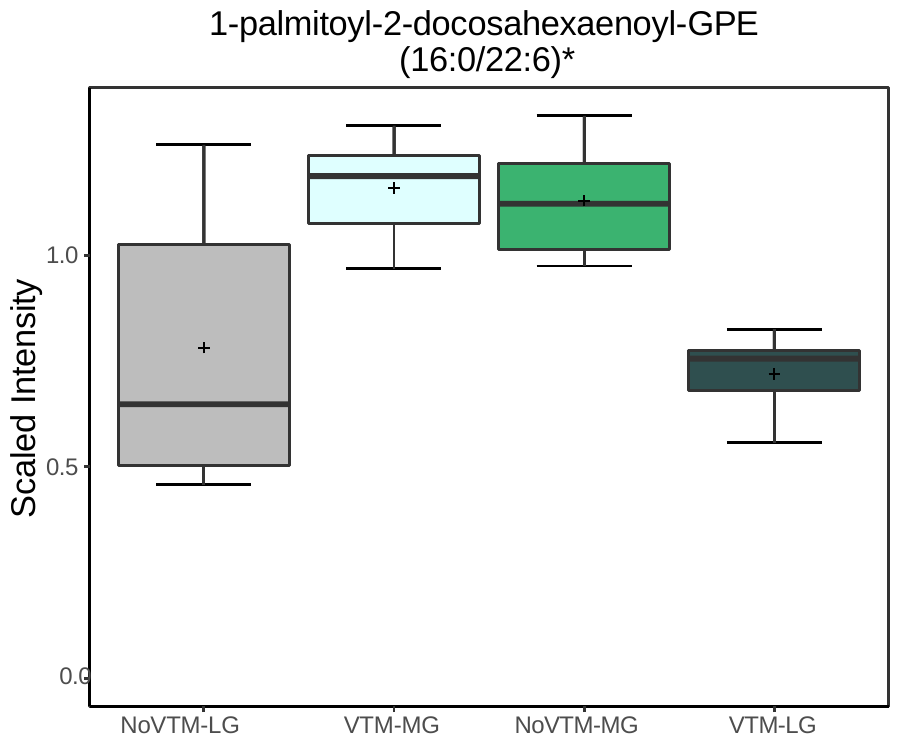

# 1-palmitoyl-2-docosahexaenoyl-GPE (16:0/22:6)*
1.0
Scaled Intensity
0.5
0.0
NoVTM-LG
VTM-MG
NoVTM-MG
VTM-LG

## Slide 196
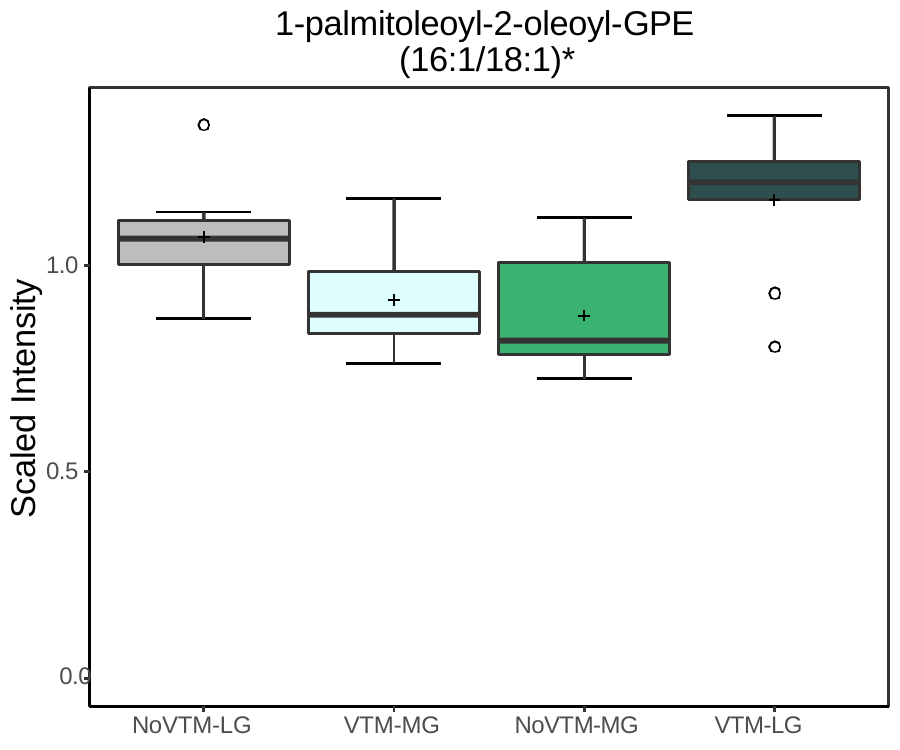

# 1-palmitoleoyl-2-oleoyl-GPE (16:1/18:1)*
1.0
Scaled Intensity
0.5
0.0
NoVTM-LG
VTM-MG
NoVTM-MG
VTM-LG

## Slide 197
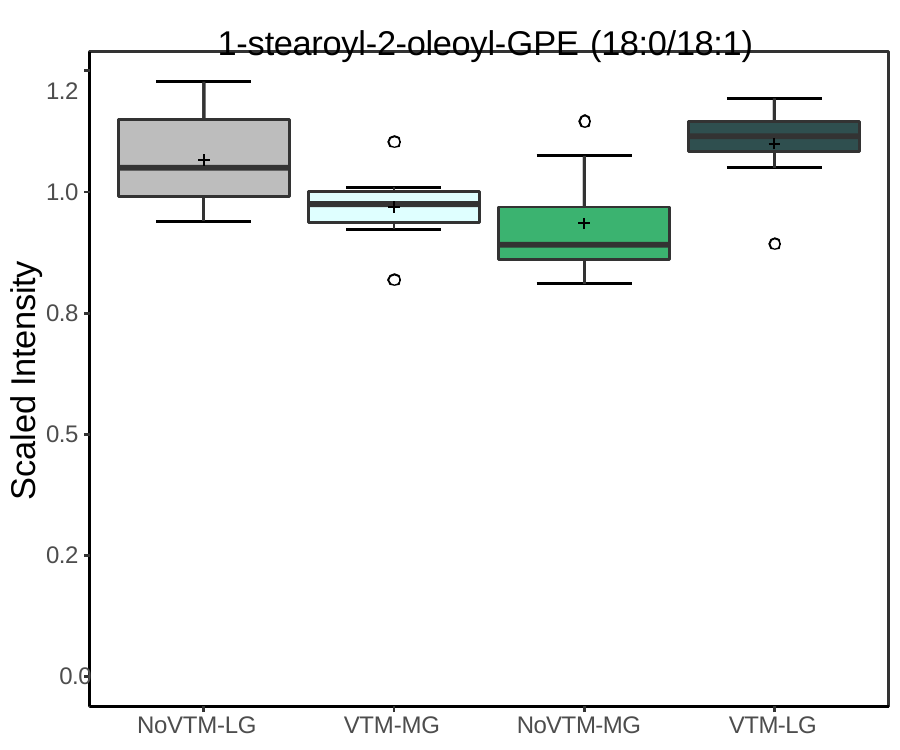

1-stearoyl-2-oleoyl-GPE (18:0/18:1)
1.2
1.0
Scaled Intensity
0.8
0.5
0.2
0.0
NoVTM-LG
VTM-MG
NoVTM-MG
VTM-LG

## Slide 198
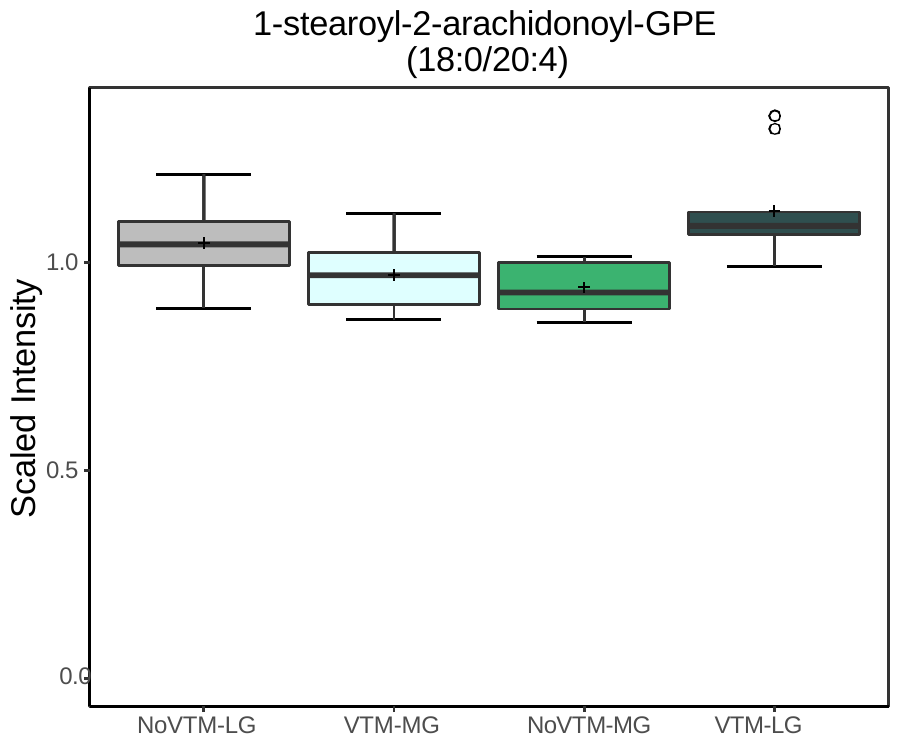

# 1-stearoyl-2-arachidonoyl-GPE (18:0/20:4)
1.0
Scaled Intensity
0.5
0.0
NoVTM-LG
VTM-MG
NoVTM-MG
VTM-LG

## Slide 199
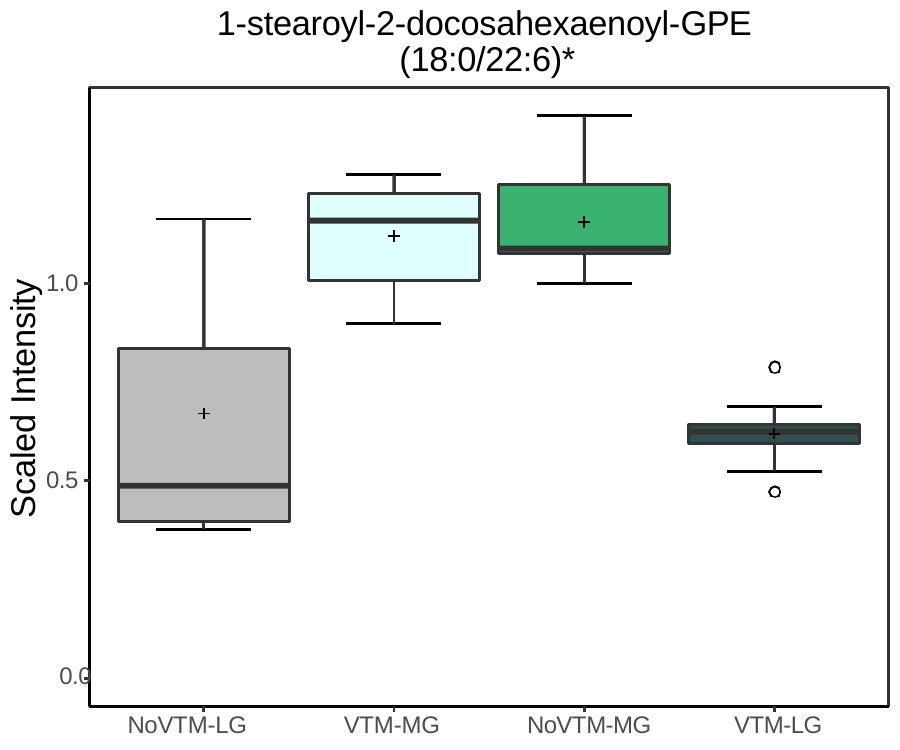

# 1-stearoyl-2-docosahexaenoyl-GPE (18:0/22:6)*
1.0
Scaled Intensity
0.5
0.0
NoVTM-LG
VTM-MG
NoVTM-MG
VTM-LG

## Slide 200
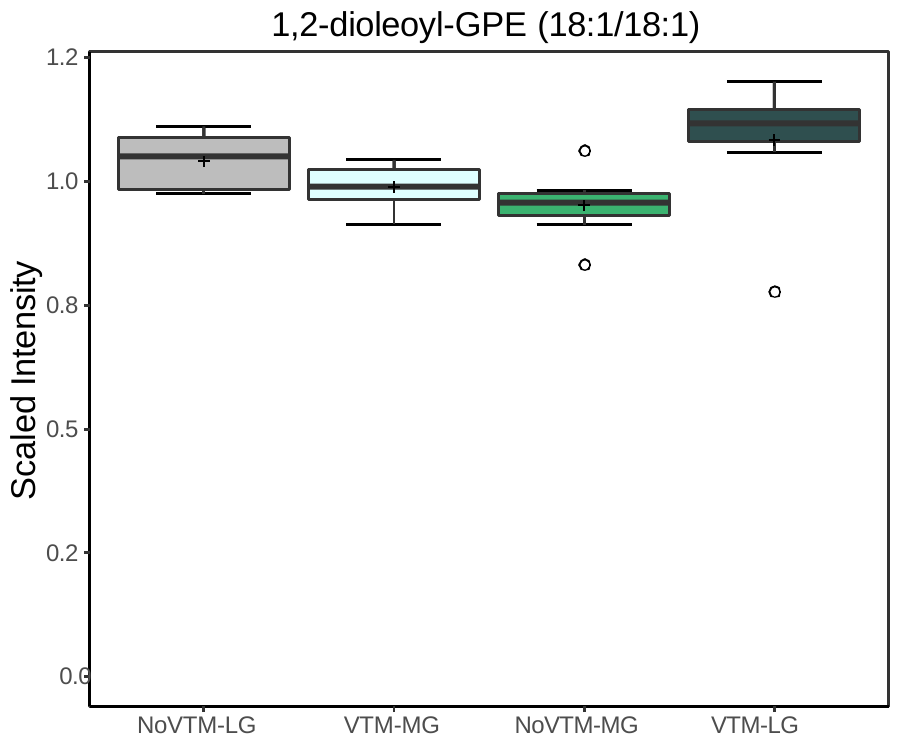

1,2-dioleoyl-GPE (18:1/18:1)
1.2
1.0
Scaled Intensity
0.8
0.5
0.2
0.0
NoVTM-LG
VTM-MG
NoVTM-MG
VTM-LG

## Slide 201
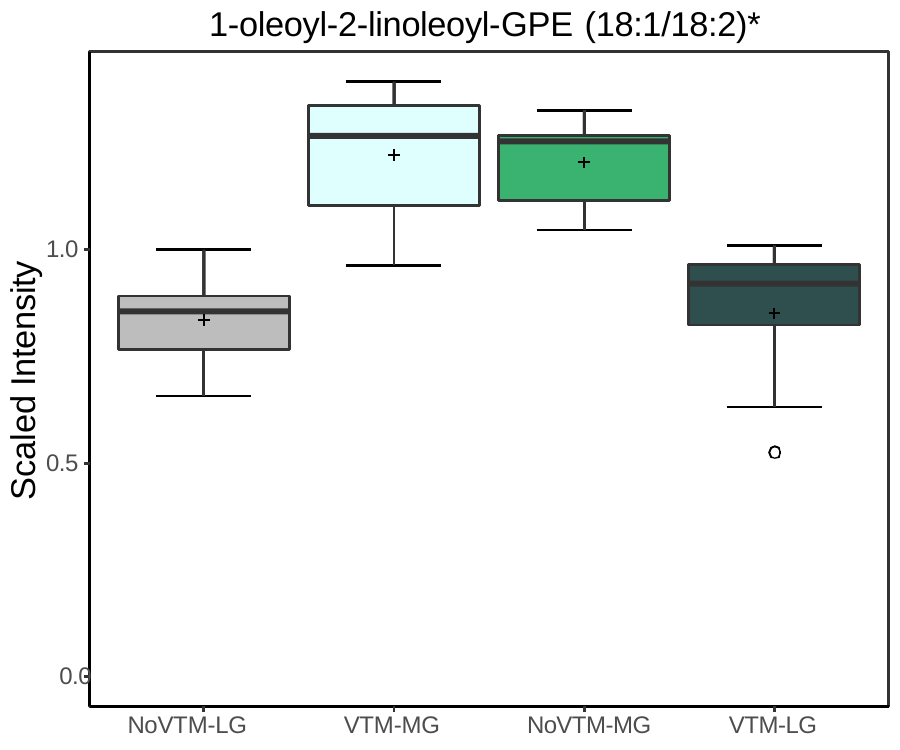

1-oleoyl-2-linoleoyl-GPE (18:1/18:2)*
1.0
Scaled Intensity
0.5
0.0
NoVTM-LG
VTM-MG
NoVTM-MG
VTM-LG

## Slide 202
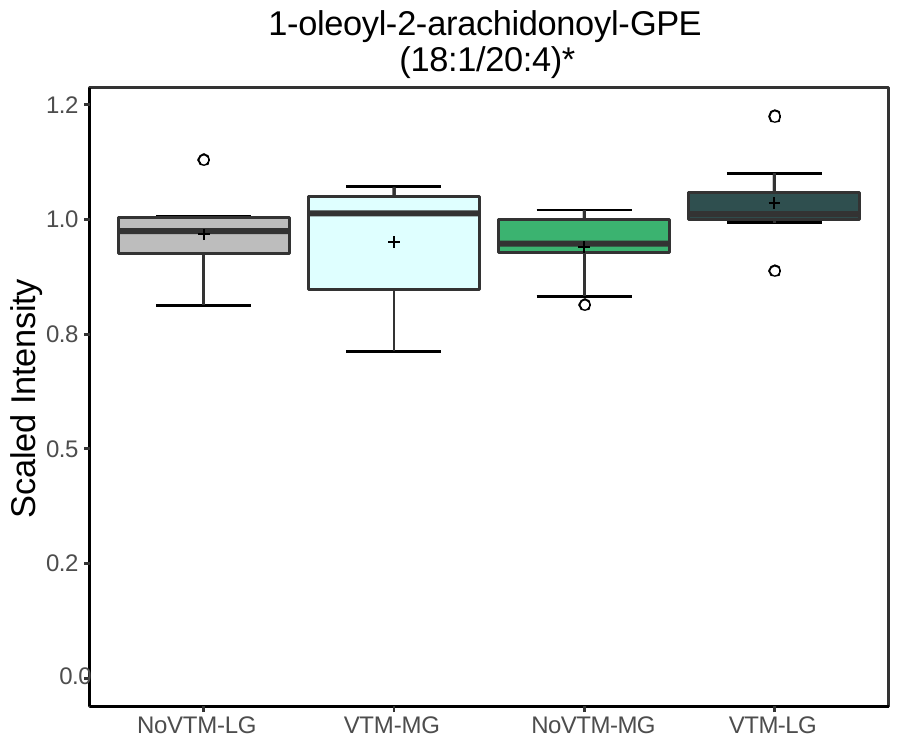

# 1-oleoyl-2-arachidonoyl-GPE (18:1/20:4)*
1.2
1.0
Scaled Intensity
0.8
0.5
0.2
0.0
NoVTM-LG
VTM-MG
NoVTM-MG
VTM-LG

## Slide 203
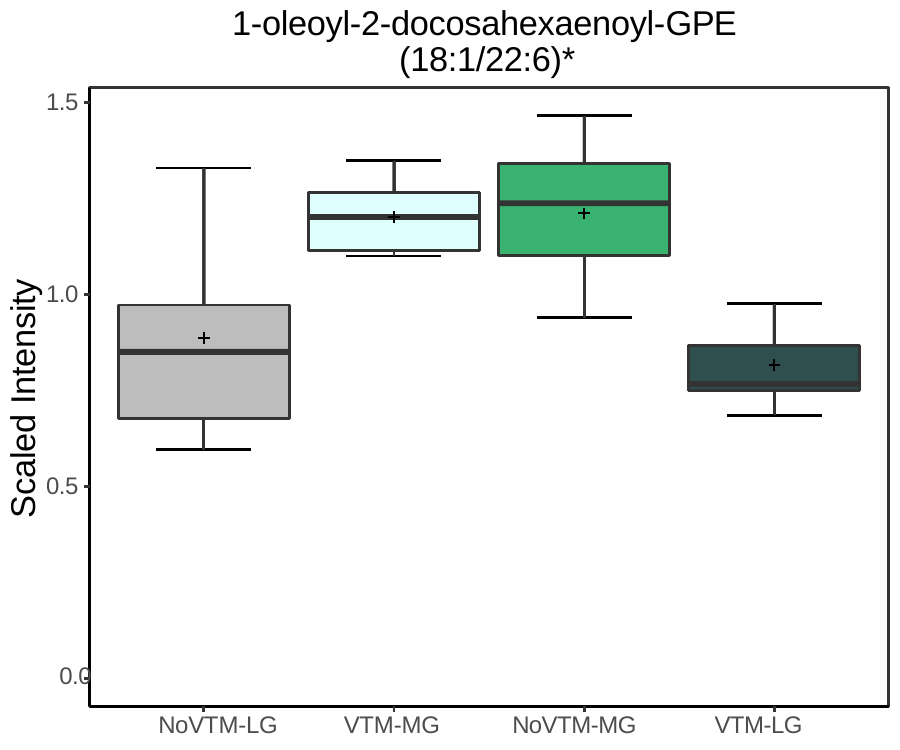

# 1-oleoyl-2-docosahexaenoyl-GPE (18:1/22:6)*
1.5
Scaled Intensity
1.0
0.5
0.0
NoVTM-LG
VTM-MG
NoVTM-MG
VTM-LG

## Slide 204
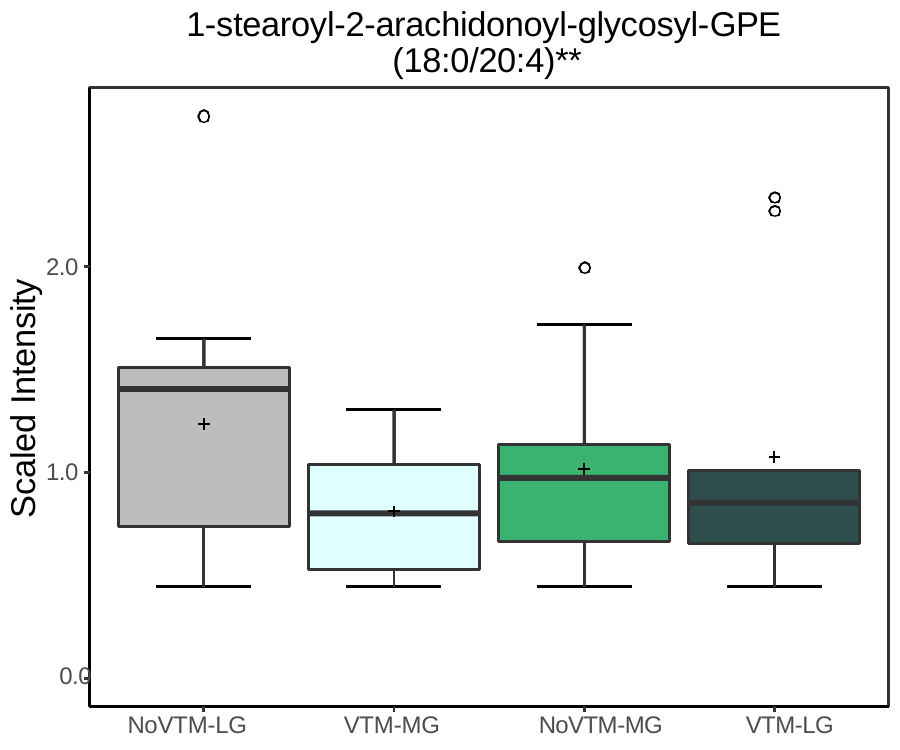

# 1-stearoyl-2-arachidonoyl-glycosyl-GPE (18:0/20:4)**
2.0
Scaled Intensity
1.0
0.0
NoVTM-LG
VTM-MG
NoVTM-MG
VTM-LG

## Slide 205
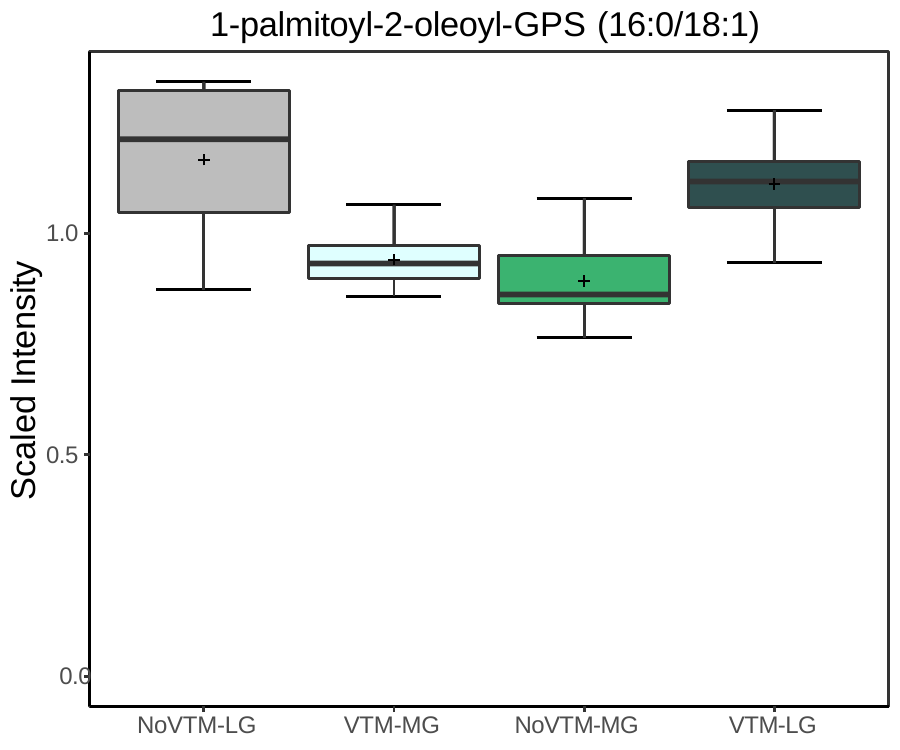

1-palmitoyl-2-oleoyl-GPS (16:0/18:1)
1.0
Scaled Intensity
0.5
0.0
NoVTM-LG
VTM-MG
NoVTM-MG
VTM-LG

## Slide 206
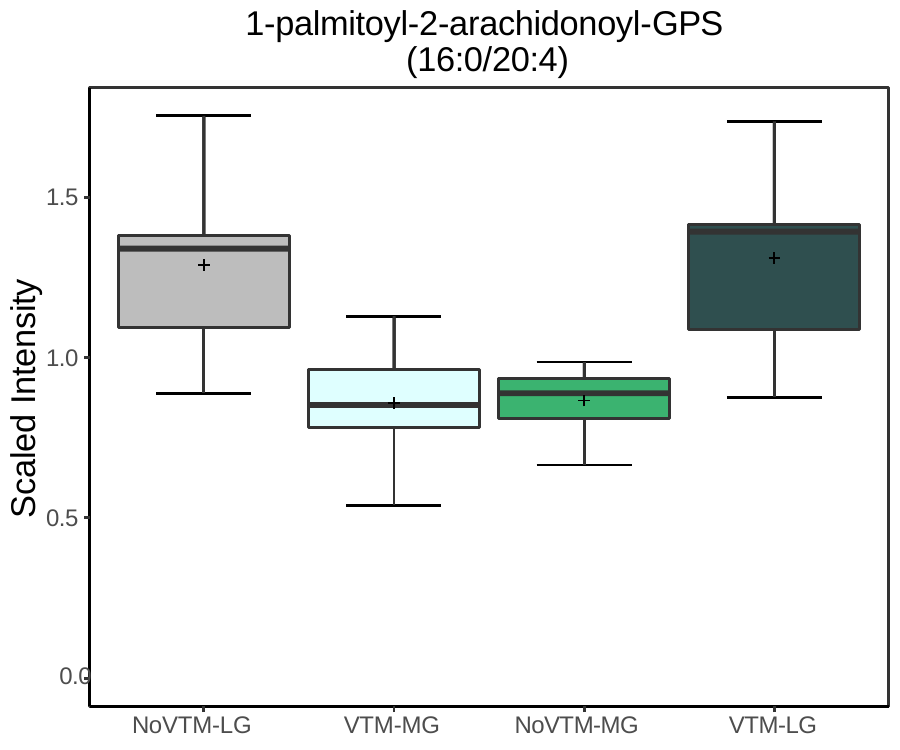

# 1-palmitoyl-2-arachidonoyl-GPS (16:0/20:4)
1.5
Scaled Intensity
1.0
0.5
0.0
NoVTM-LG
VTM-MG
NoVTM-MG
VTM-LG

## Slide 207
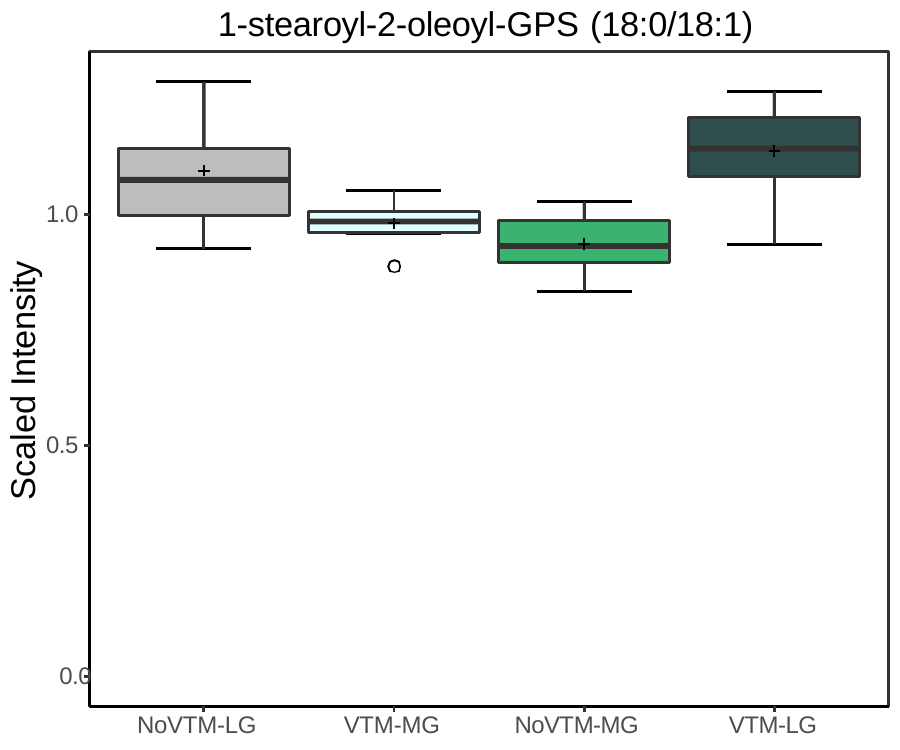

1-stearoyl-2-oleoyl-GPS (18:0/18:1)
1.0
Scaled Intensity
0.5
0.0
NoVTM-LG
VTM-MG
NoVTM-MG
VTM-LG

## Slide 208
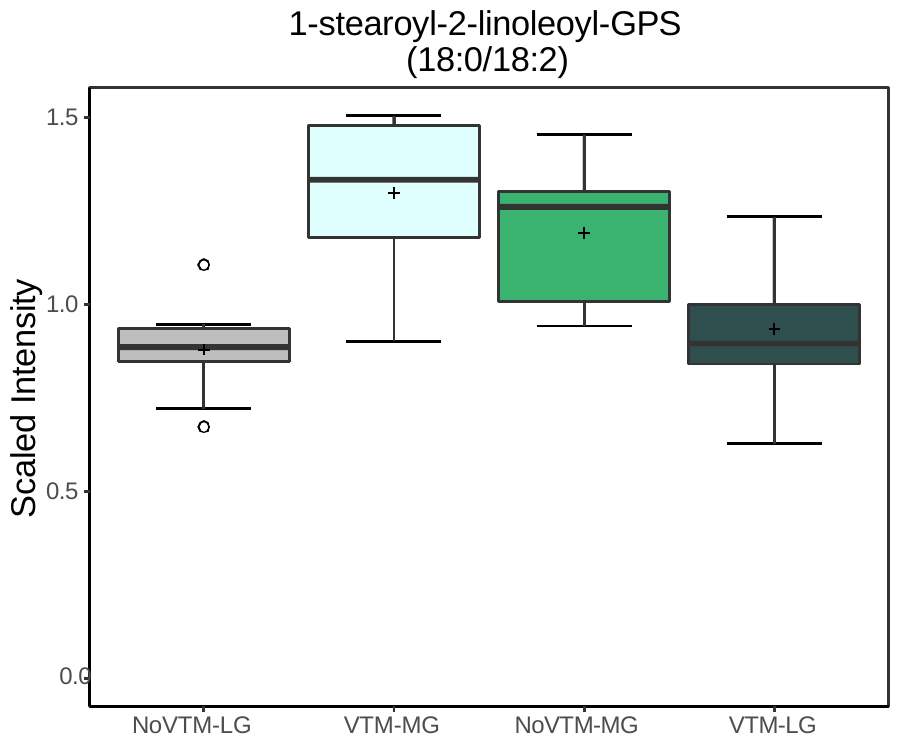

# 1-stearoyl-2-linoleoyl-GPS (18:0/18:2)
1.5
Scaled Intensity
1.0
0.5
0.0
NoVTM-LG
VTM-MG
NoVTM-MG
VTM-LG

## Slide 209
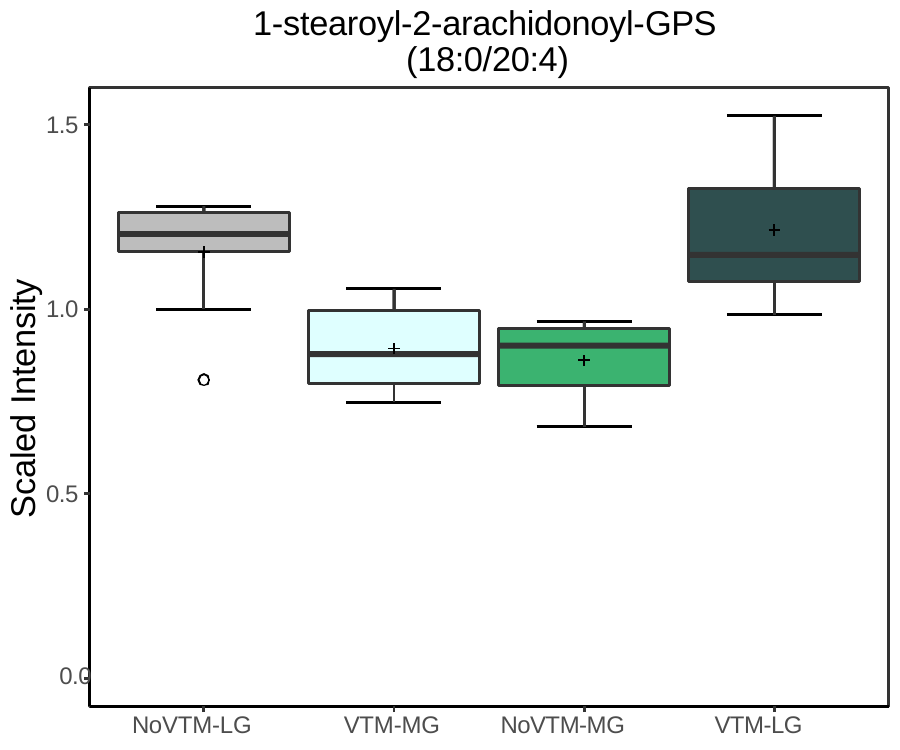

# 1-stearoyl-2-arachidonoyl-GPS (18:0/20:4)
1.5
Scaled Intensity
1.0
0.5
0.0
NoVTM-LG
VTM-MG
NoVTM-MG
VTM-LG

## Slide 210
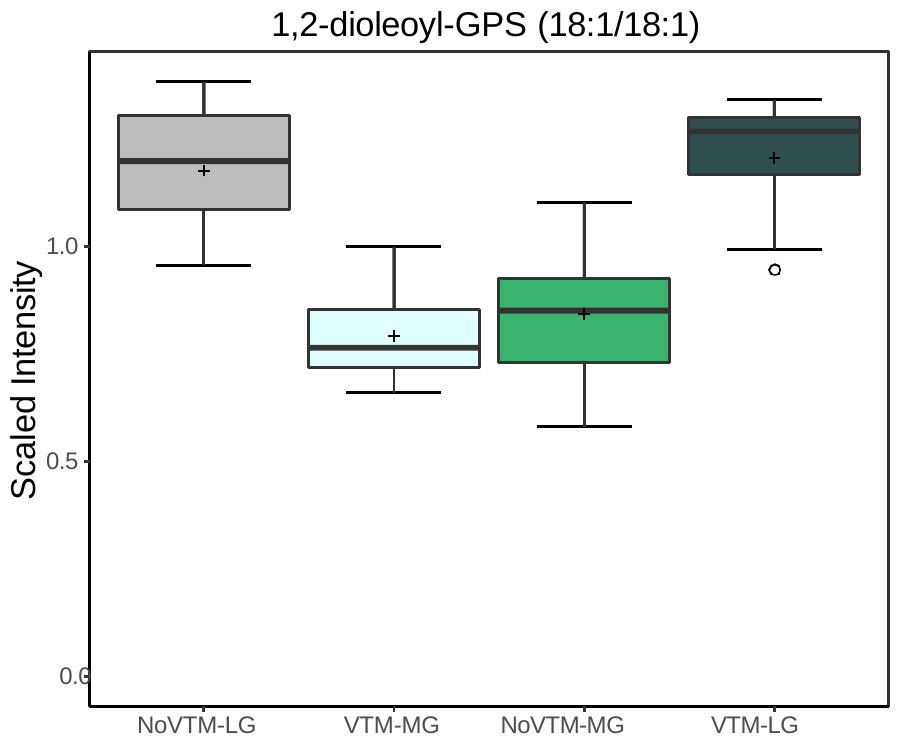

1,2-dioleoyl-GPS (18:1/18:1)
1.0
Scaled Intensity
0.5
0.0
NoVTM-LG
VTM-MG
NoVTM-MG
VTM-LG

## Slide 211
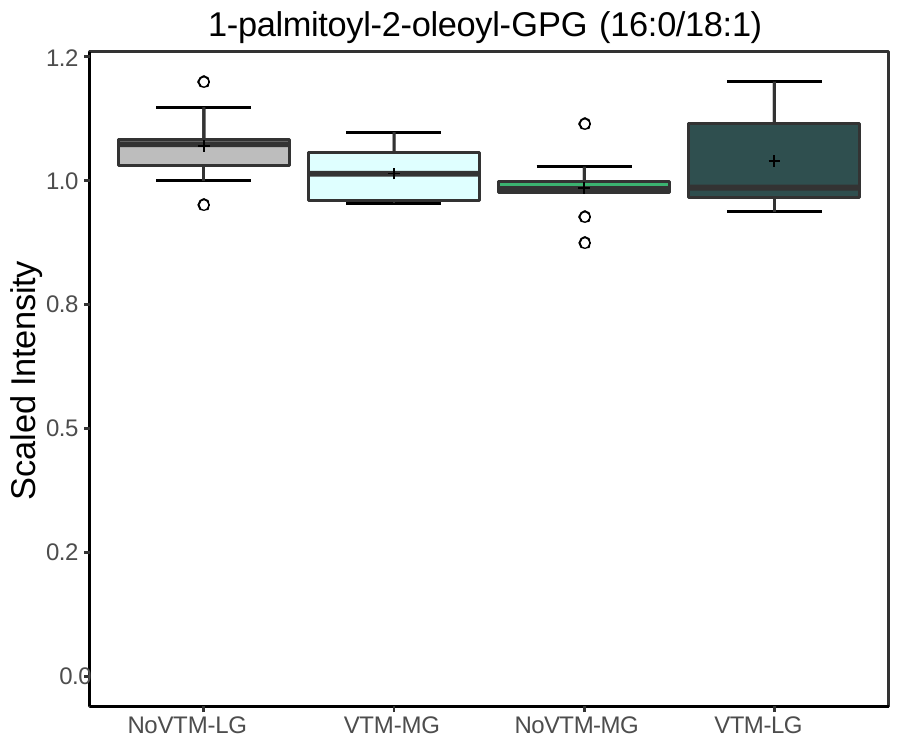

1-palmitoyl-2-oleoyl-GPG (16:0/18:1)
1.2
1.0
Scaled Intensity
0.8
0.5
0.2
0.0
NoVTM-LG
VTM-MG
NoVTM-MG
VTM-LG

## Slide 212
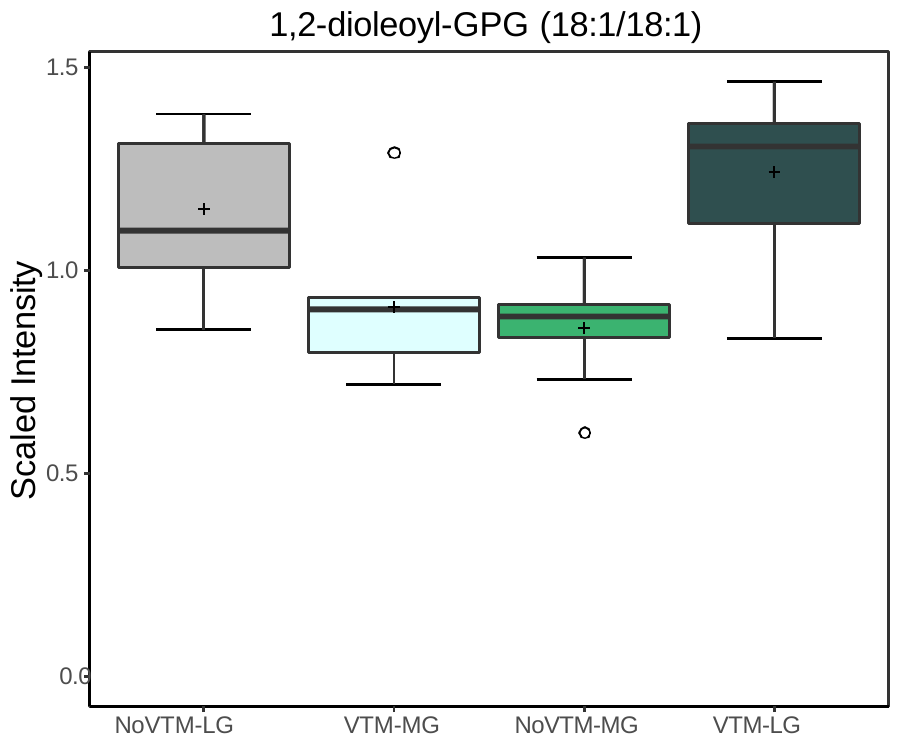

1,2-dioleoyl-GPG (18:1/18:1)
1.5
1.0
Scaled Intensity
0.5
0.0
NoVTM-LG
VTM-MG
NoVTM-MG
VTM-LG

## Slide 213
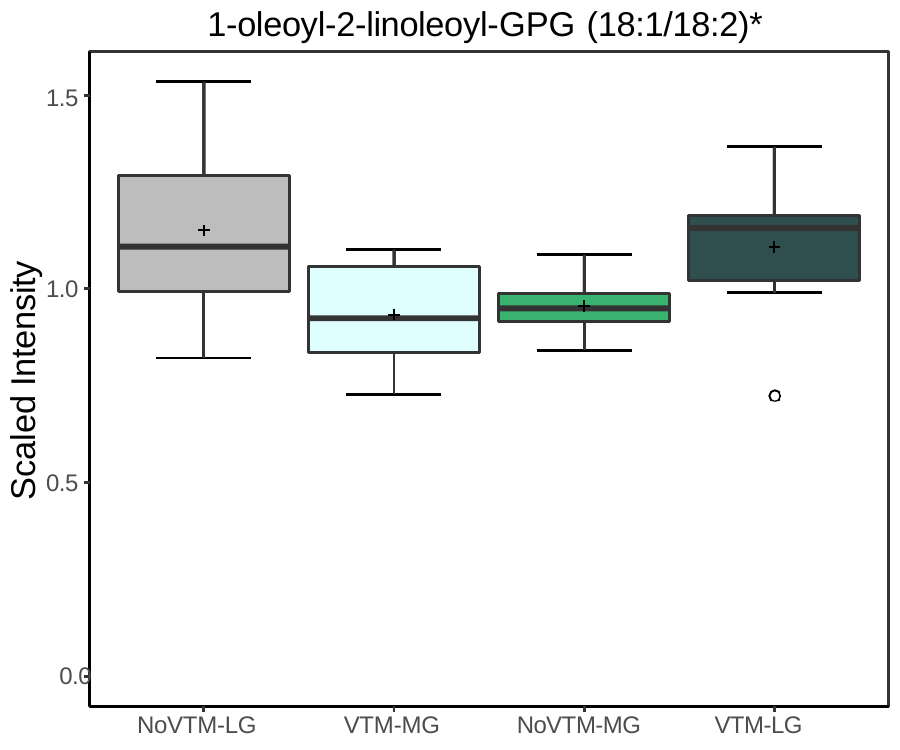

1-oleoyl-2-linoleoyl-GPG (18:1/18:2)*
1.5
Scaled Intensity
1.0
0.5
0.0
NoVTM-LG
VTM-MG
NoVTM-MG
VTM-LG

## Slide 214
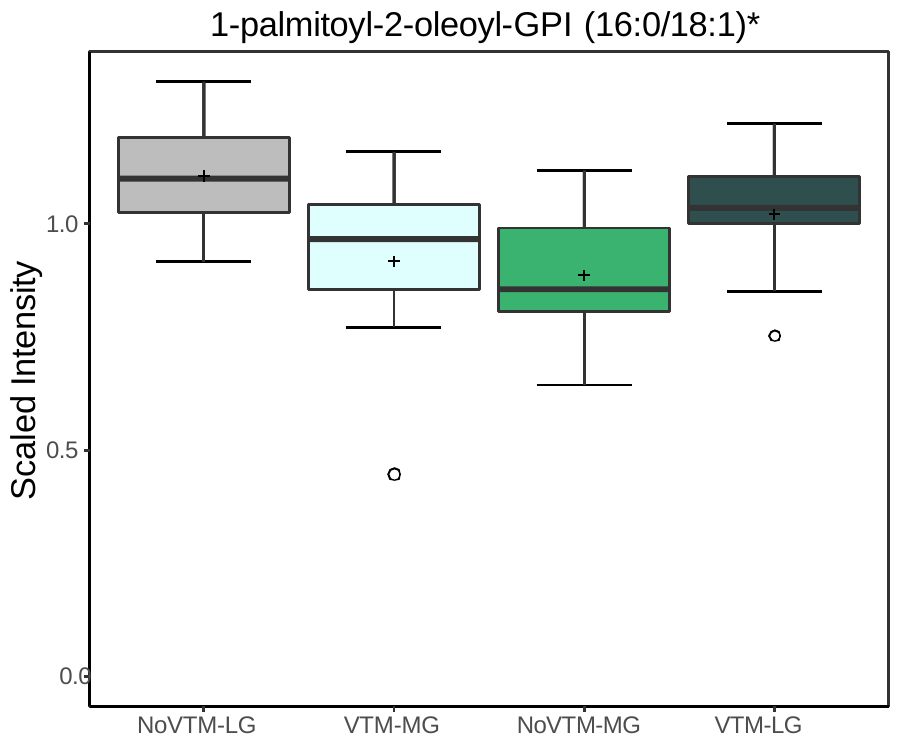

1-palmitoyl-2-oleoyl-GPI (16:0/18:1)*
1.0
Scaled Intensity
0.5
0.0
NoVTM-LG
VTM-MG
NoVTM-MG
VTM-LG

## Slide 215
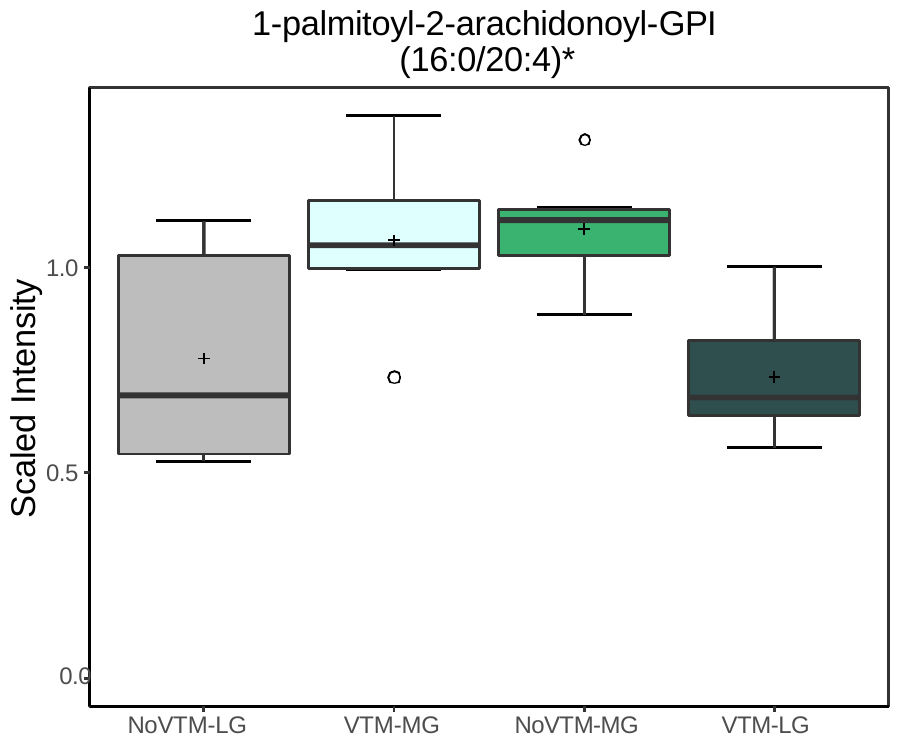

# 1-palmitoyl-2-arachidonoyl-GPI (16:0/20:4)*
1.0
Scaled Intensity
0.5
0.0
NoVTM-LG
VTM-MG
NoVTM-MG
VTM-LG

## Slide 216
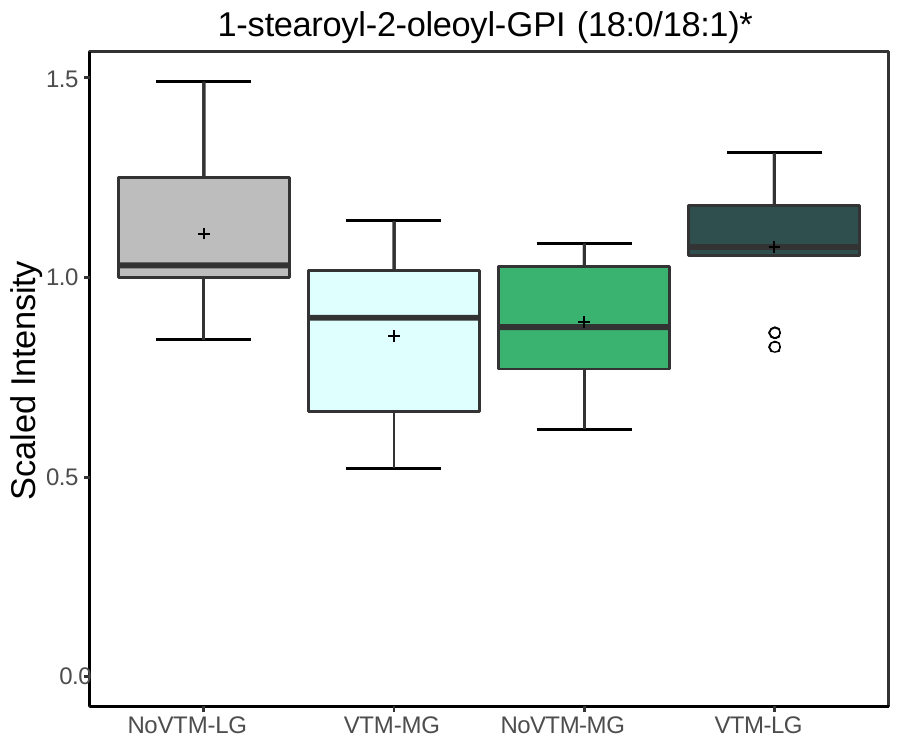

1-stearoyl-2-oleoyl-GPI (18:0/18:1)*
1.5
Scaled Intensity
1.0
0.5
0.0
NoVTM-LG
VTM-MG
NoVTM-MG
VTM-LG

## Slide 217
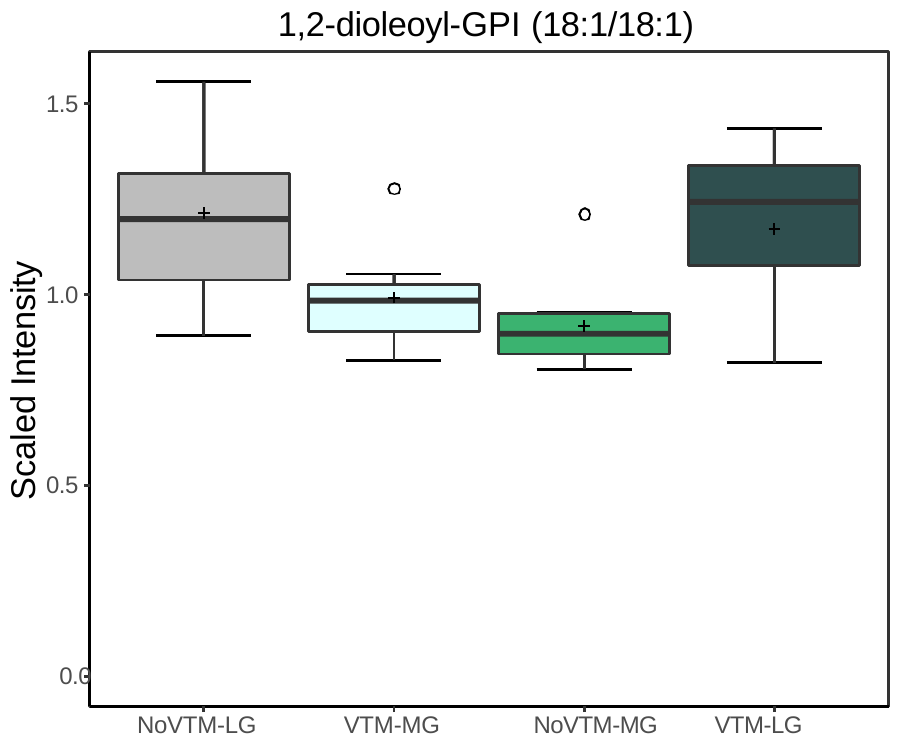

1,2-dioleoyl-GPI (18:1/18:1)
1.5
Scaled Intensity
1.0
0.5
0.0
NoVTM-LG
VTM-MG
NoVTM-MG
VTM-LG

## Slide 218
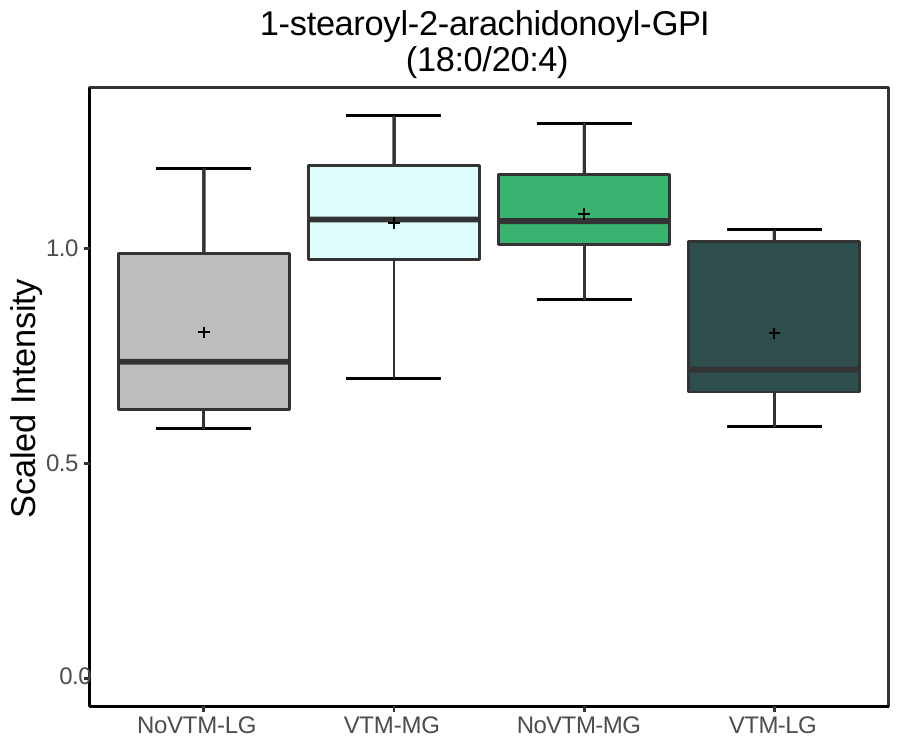

# 1-stearoyl-2-arachidonoyl-GPI (18:0/20:4)
1.0
Scaled Intensity
0.5
0.0
NoVTM-LG
VTM-MG
NoVTM-MG
VTM-LG

## Slide 219
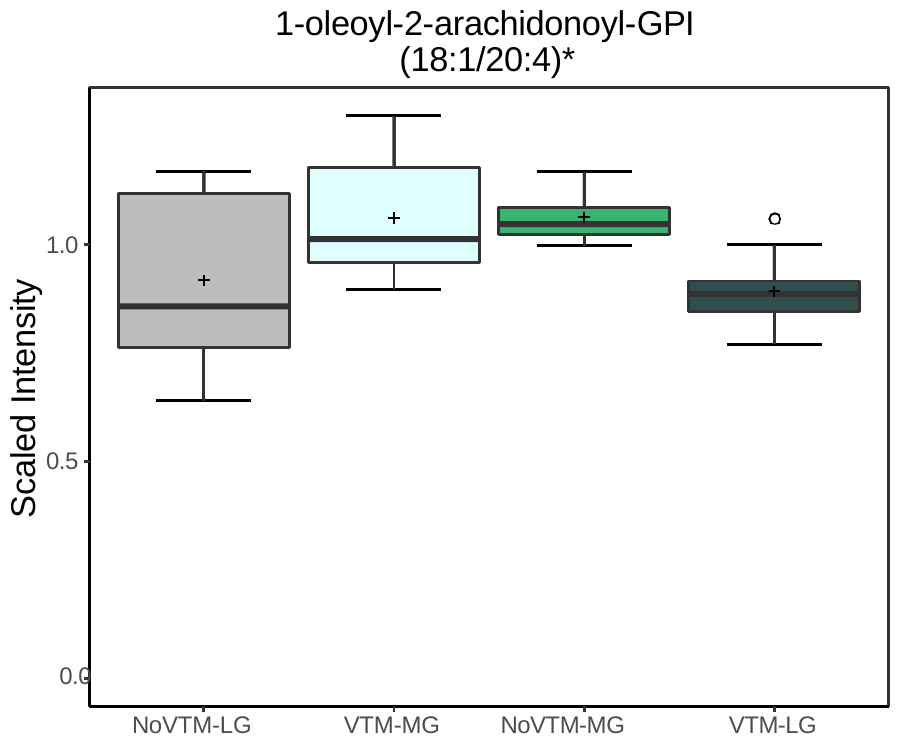

# 1-oleoyl-2-arachidonoyl-GPI (18:1/20:4)*
1.0
Scaled Intensity
0.5
0.0
NoVTM-LG
VTM-MG
NoVTM-MG
VTM-LG

## Slide 220
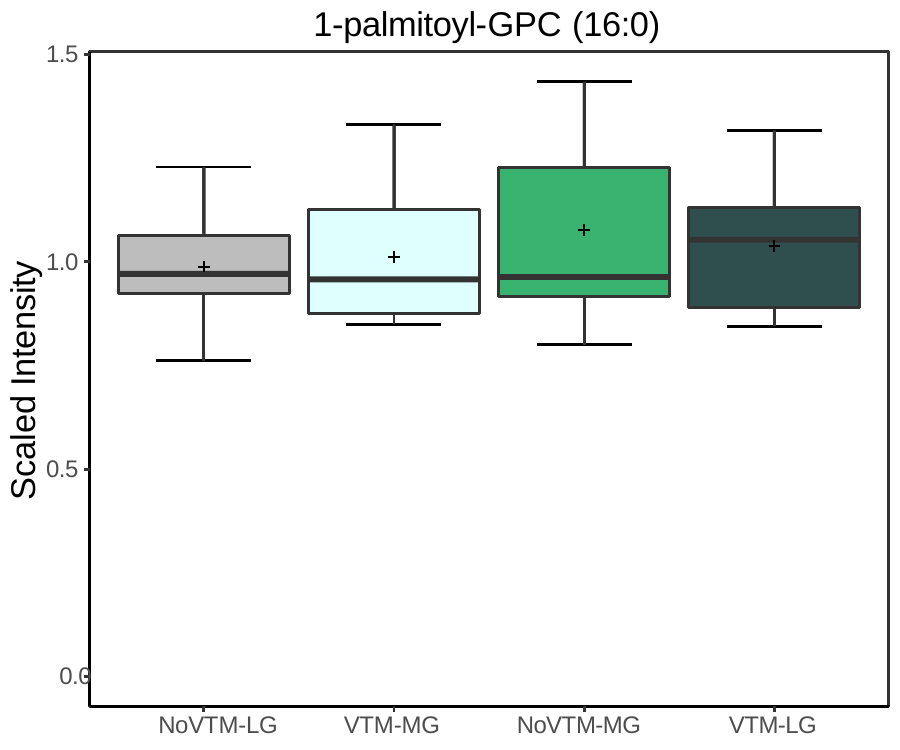

1-palmitoyl-GPC (16:0)
1.5
1.0
Scaled Intensity
0.5
0.0
NoVTM-LG
VTM-MG
NoVTM-MG
VTM-LG

## Slide 221
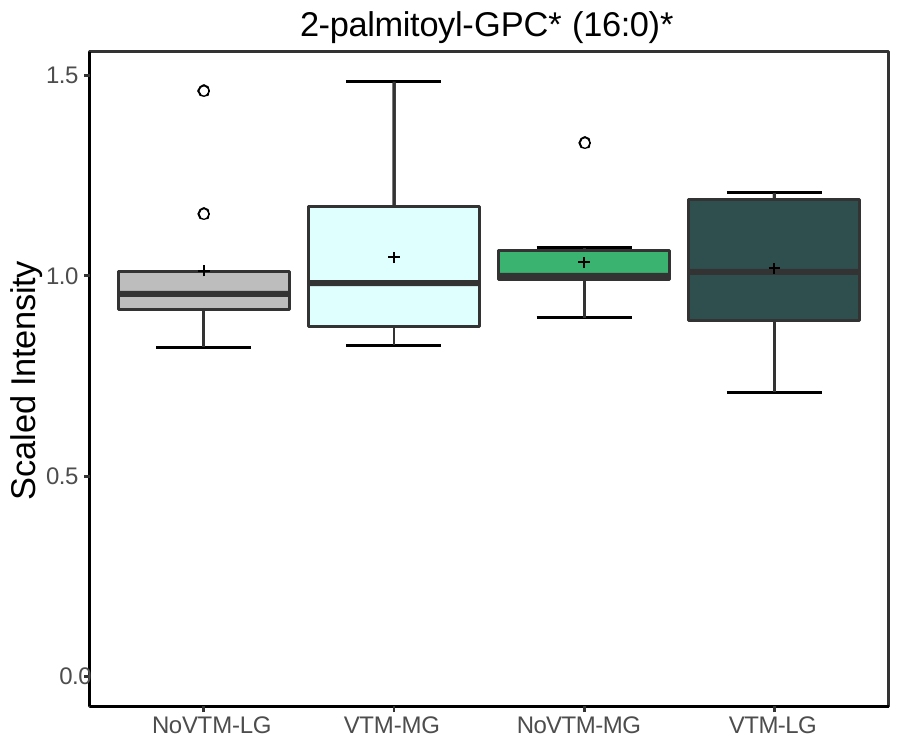

2-palmitoyl-GPC* (16:0)*
1.5
Scaled Intensity
1.0
0.5
0.0
NoVTM-LG
VTM-MG
NoVTM-MG
VTM-LG

## Slide 222
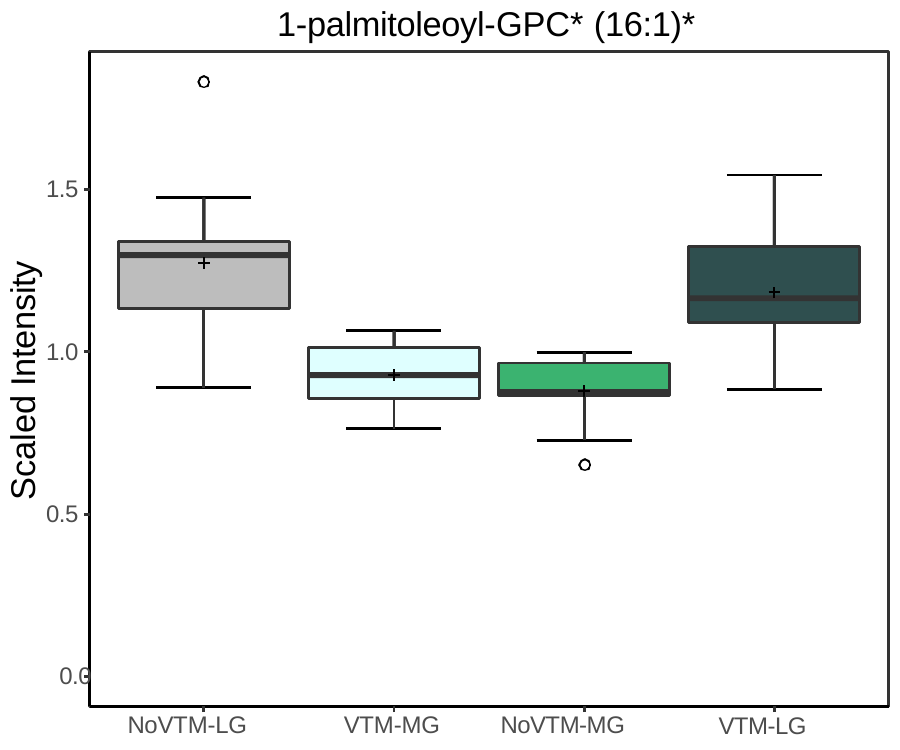

1-palmitoleoyl-GPC* (16:1)*
1.5
Scaled Intensity
1.0
0.5
0.0
NoVTM-LG
VTM-MG
NoVTM-MG
VTM-LG

## Slide 223
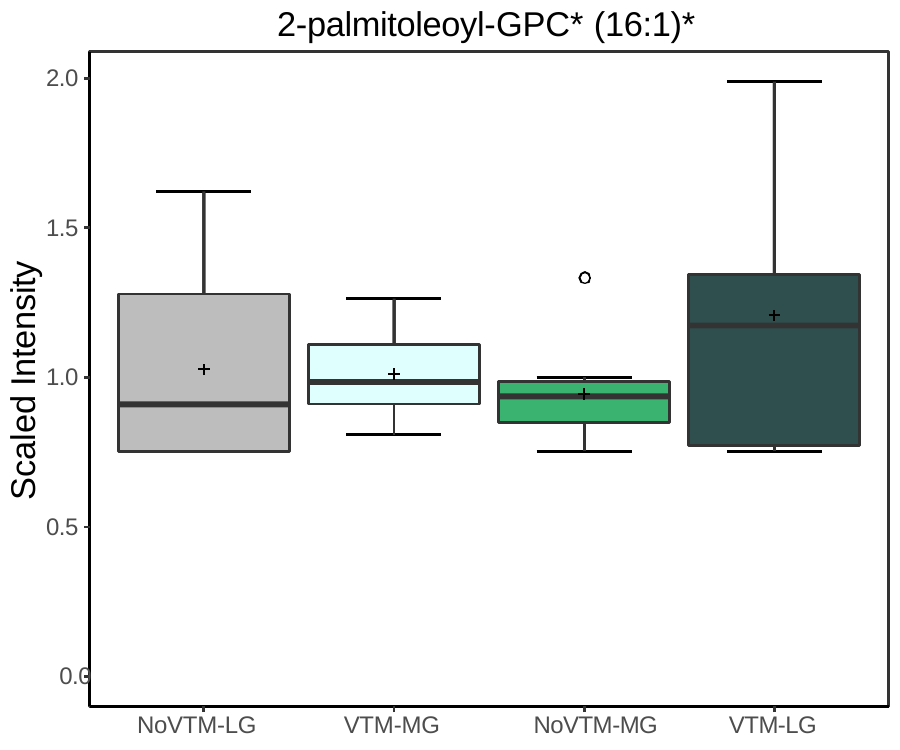

2-palmitoleoyl-GPC* (16:1)*
2.0
1.5
Scaled Intensity
1.0
0.5
0.0
NoVTM-LG
VTM-MG
NoVTM-MG
VTM-LG

## Slide 224
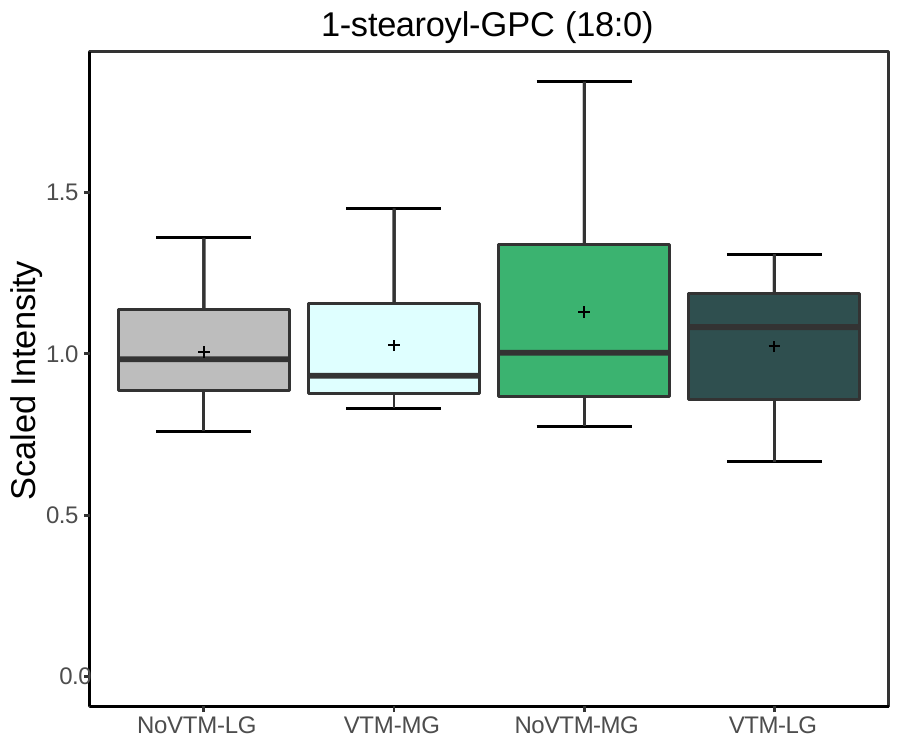

1-stearoyl-GPC (18:0)
1.5
Scaled Intensity
1.0
0.5
0.0
NoVTM-LG
VTM-MG
NoVTM-MG
VTM-LG

## Slide 225
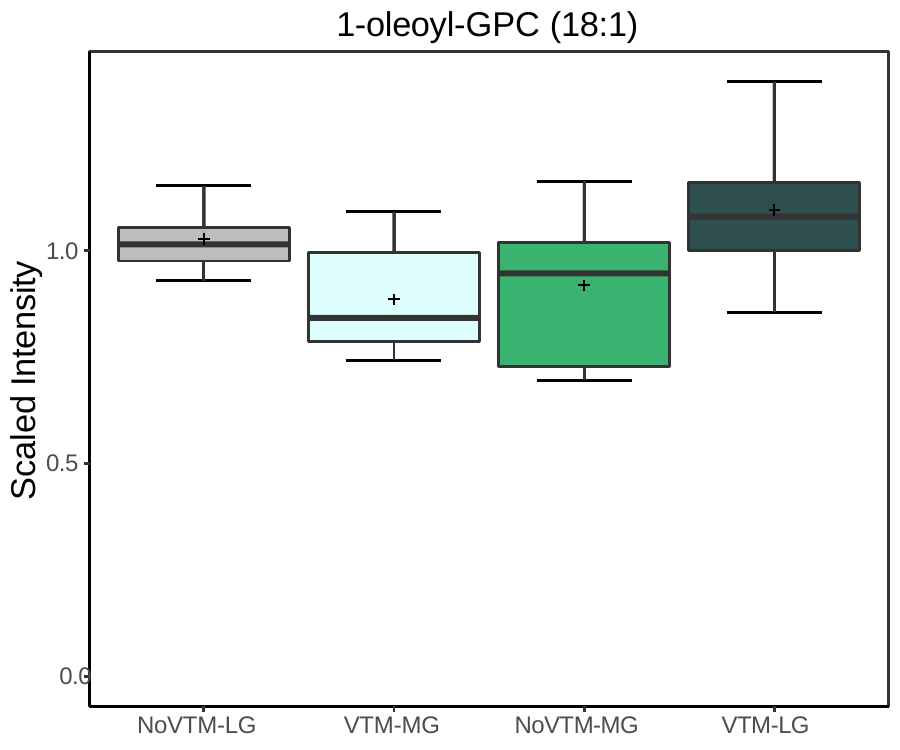

1-oleoyl-GPC (18:1)
1.0
Scaled Intensity
0.5
0.0
NoVTM-LG
VTM-MG
NoVTM-MG
VTM-LG

## Slide 226
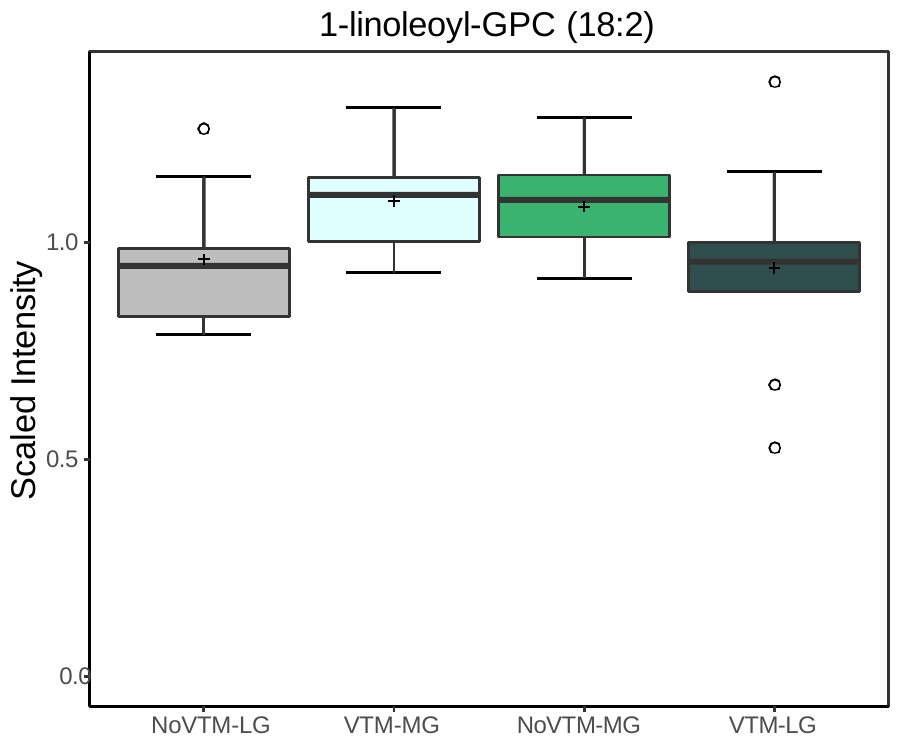

1-linoleoyl-GPC (18:2)
1.0
Scaled Intensity
0.5
0.0
NoVTM-LG
VTM-MG
NoVTM-MG
VTM-LG

## Slide 227
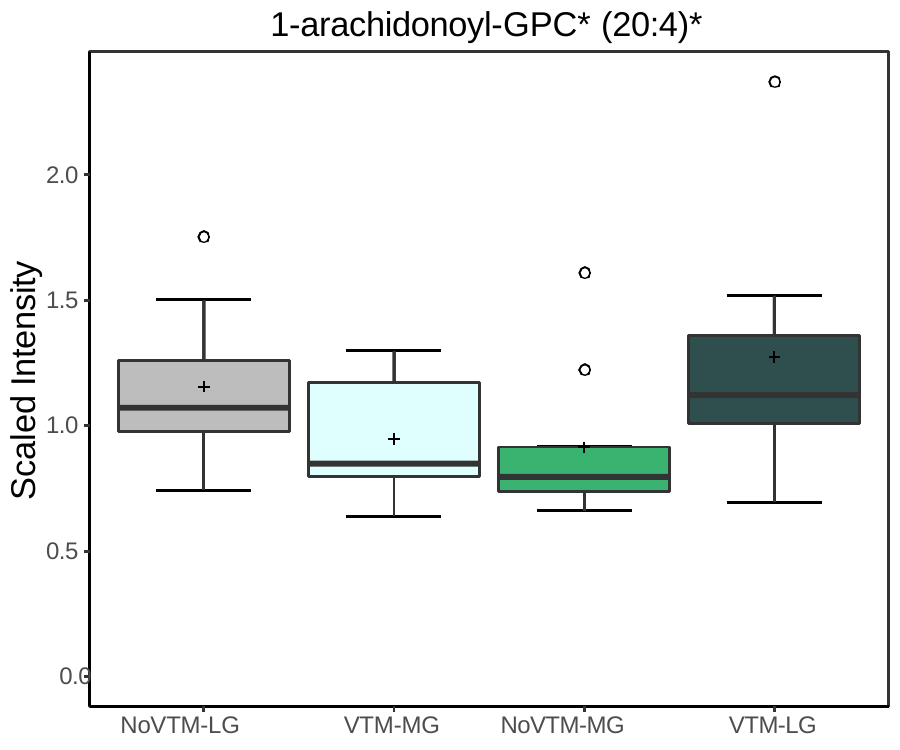

1-arachidonoyl-GPC* (20:4)*
2.0
Scaled Intensity
1.5
1.0
0.5
0.0
NoVTM-LG
VTM-MG
NoVTM-MG
VTM-LG

## Slide 228
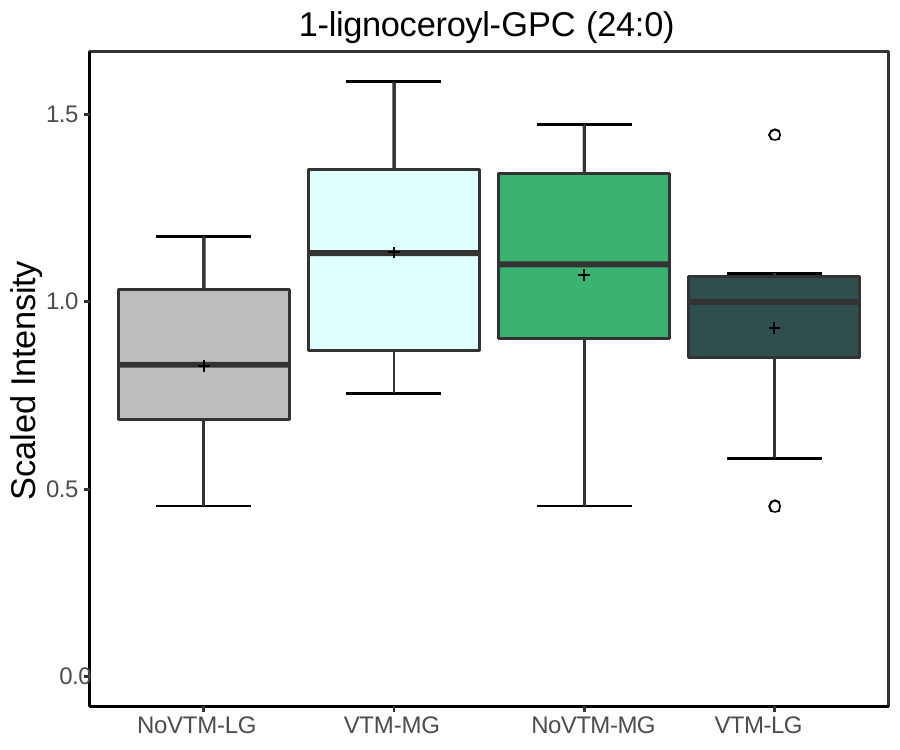

1-lignoceroyl-GPC (24:0)
1.5
Scaled Intensity
1.0
0.5
0.0
NoVTM-LG
VTM-MG
NoVTM-MG
VTM-LG

## Slide 229
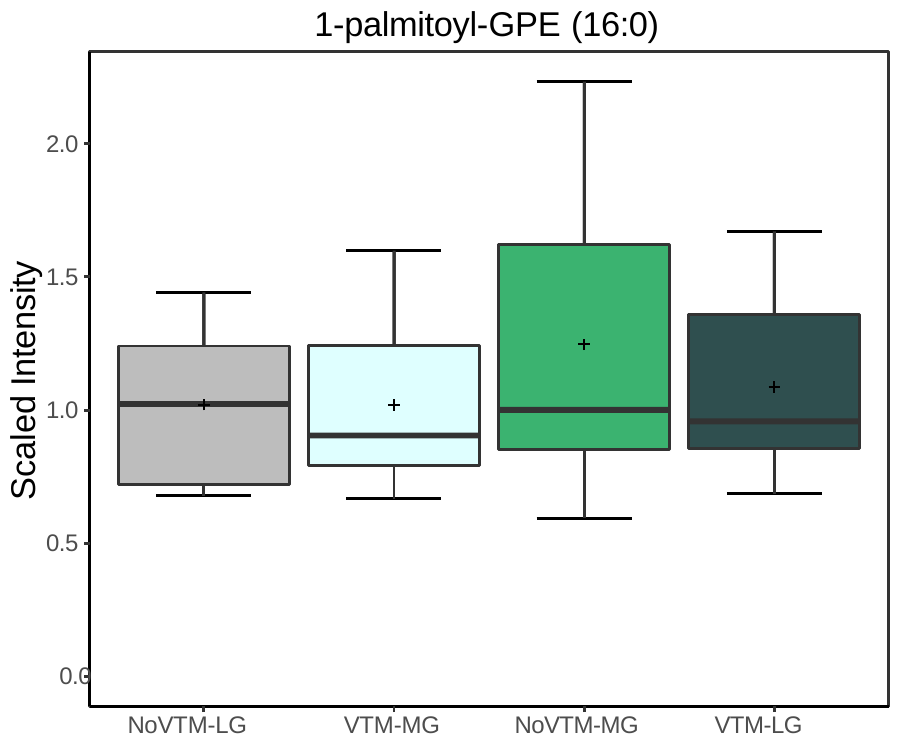

1-palmitoyl-GPE (16:0)
2.0
Scaled Intensity
1.5
1.0
0.5
0.0
NoVTM-LG
VTM-MG
NoVTM-MG
VTM-LG

## Slide 230
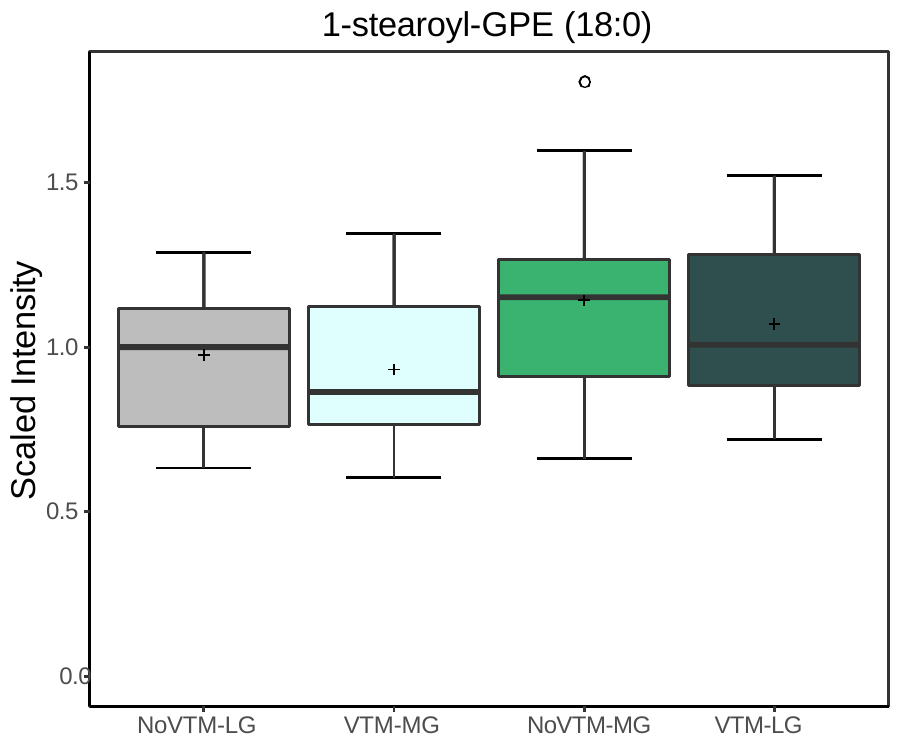

1-stearoyl-GPE (18:0)
1.5
Scaled Intensity
1.0
0.5
0.0
NoVTM-LG
VTM-MG
NoVTM-MG
VTM-LG

## Slide 231
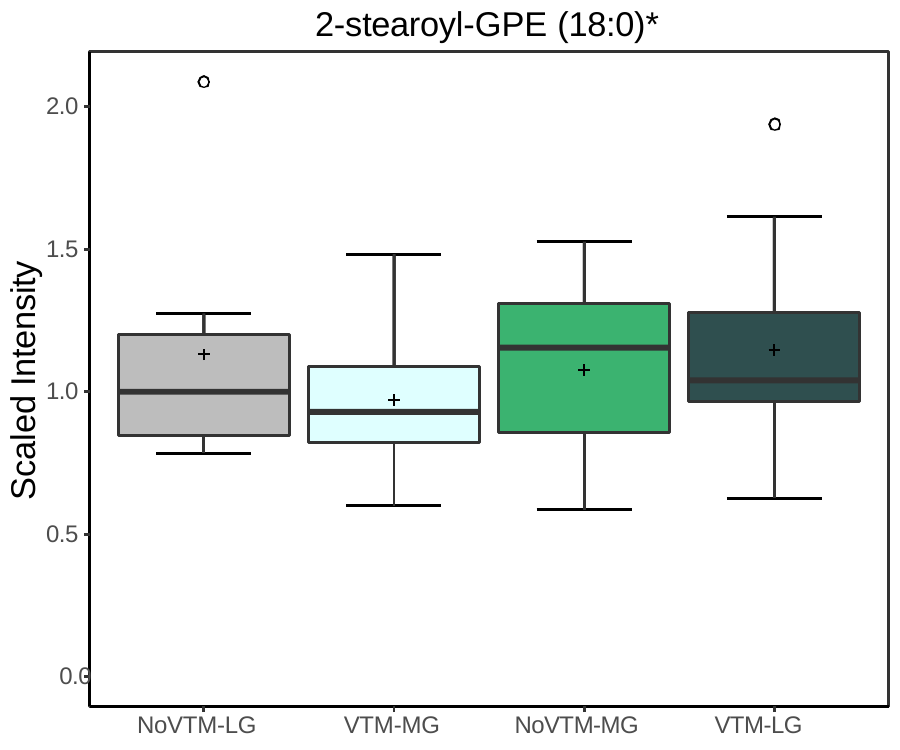

2-stearoyl-GPE (18:0)*
2.0
1.5
Scaled Intensity
1.0
0.5
0.0
NoVTM-LG
VTM-MG
NoVTM-MG
VTM-LG

## Slide 232
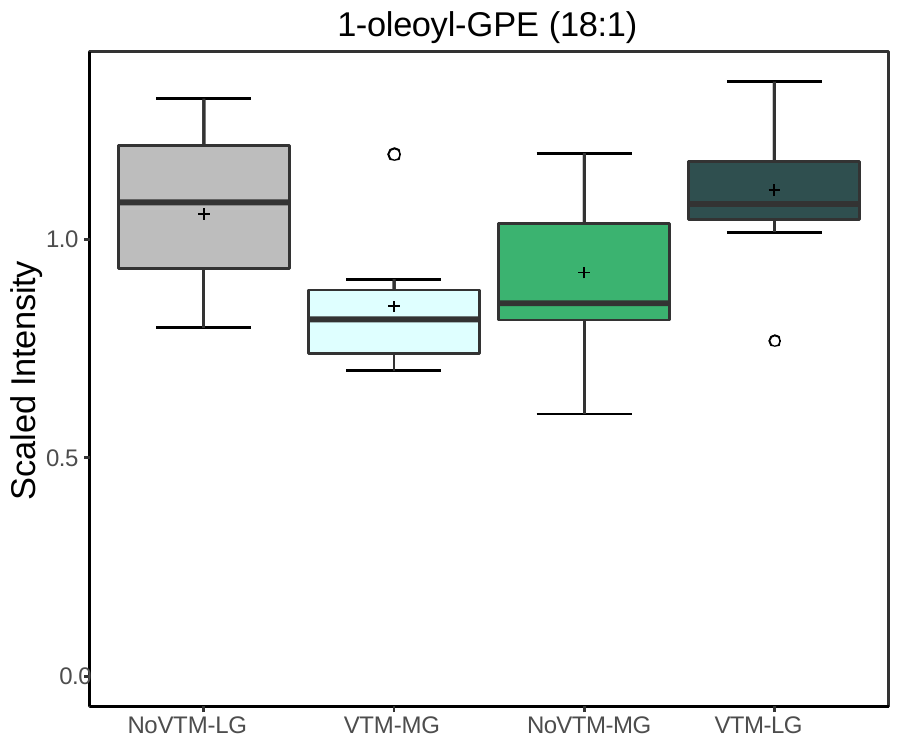

1-oleoyl-GPE (18:1)
1.0
Scaled Intensity
0.5
0.0
NoVTM-LG
VTM-MG
NoVTM-MG
VTM-LG

## Slide 233
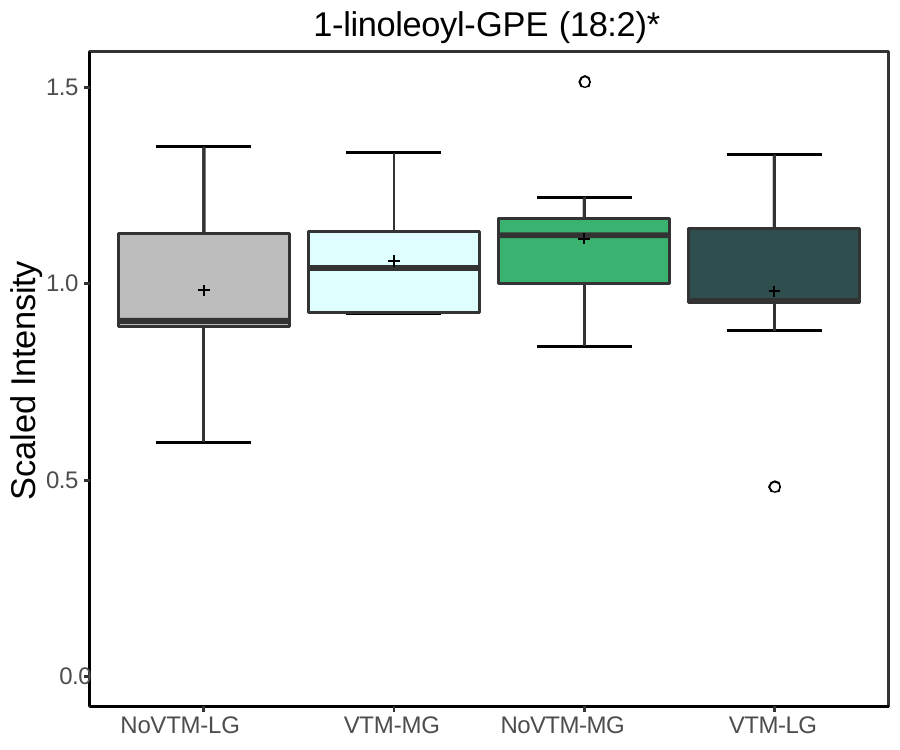

1-linoleoyl-GPE (18:2)*
1.5
Scaled Intensity
1.0
0.5
0.0
NoVTM-LG
VTM-MG
NoVTM-MG
VTM-LG

## Slide 234
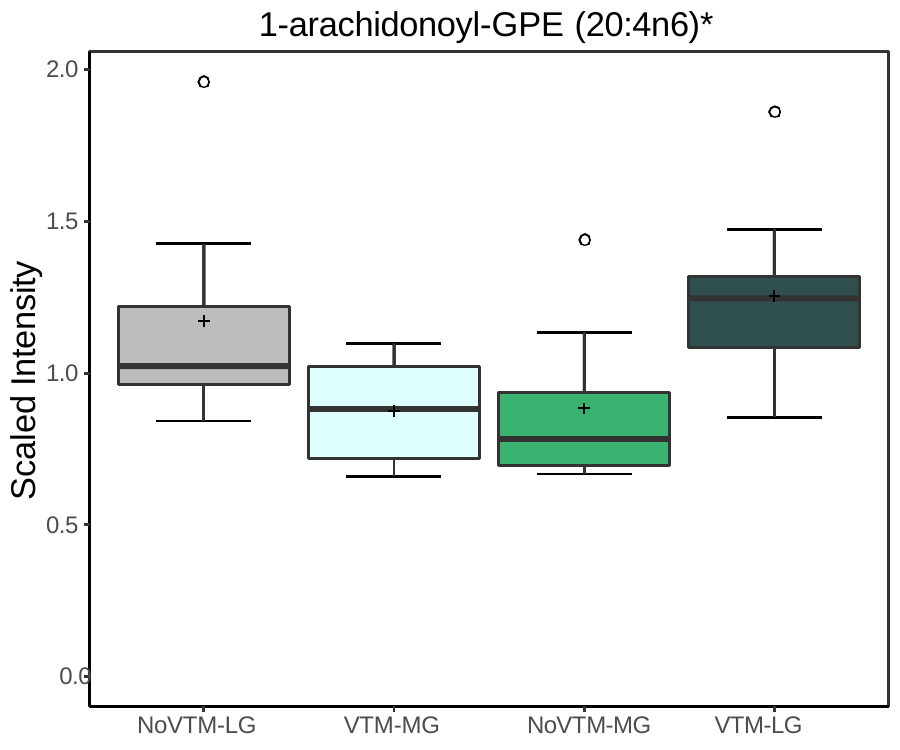

1-arachidonoyl-GPE (20:4n6)*
2.0
1.5
Scaled Intensity
1.0
0.5
0.0
NoVTM-LG
VTM-MG
NoVTM-MG
VTM-LG

## Slide 235
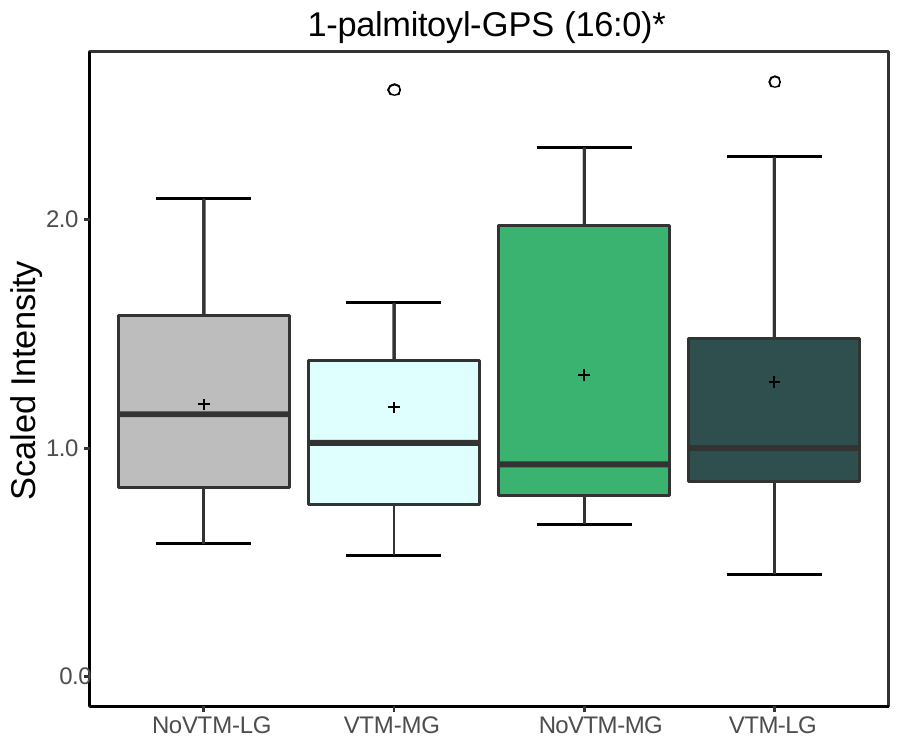

1-palmitoyl-GPS (16:0)*
2.0
Scaled Intensity
1.0
0.0
NoVTM-LG
VTM-MG
NoVTM-MG
VTM-LG

## Slide 236
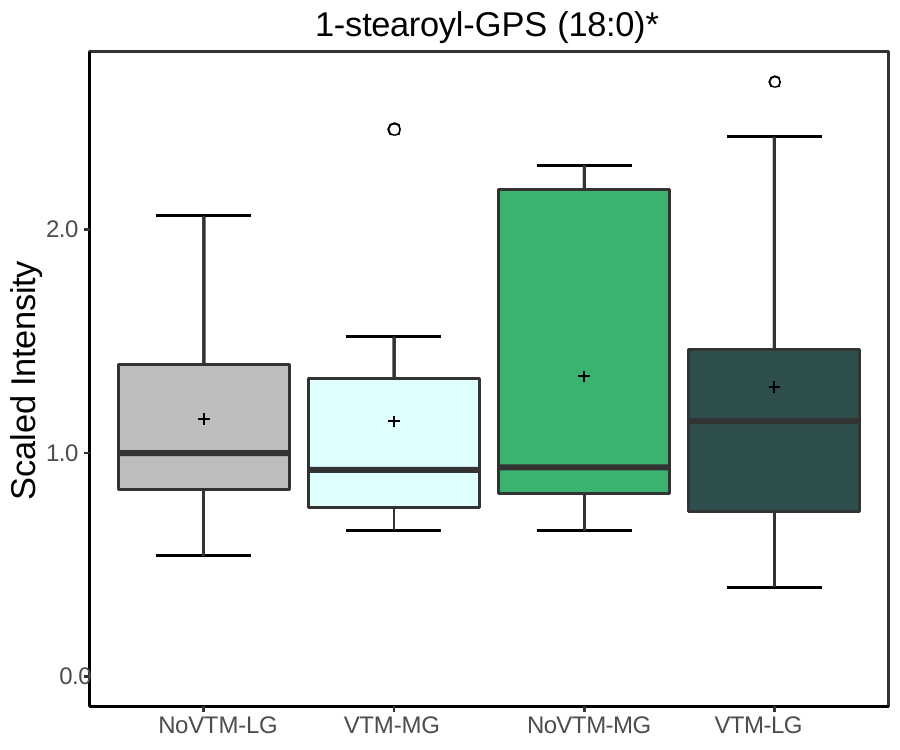

1-stearoyl-GPS (18:0)*
2.0
Scaled Intensity
1.0
0.0
NoVTM-LG
VTM-MG
NoVTM-MG
VTM-LG

## Slide 237
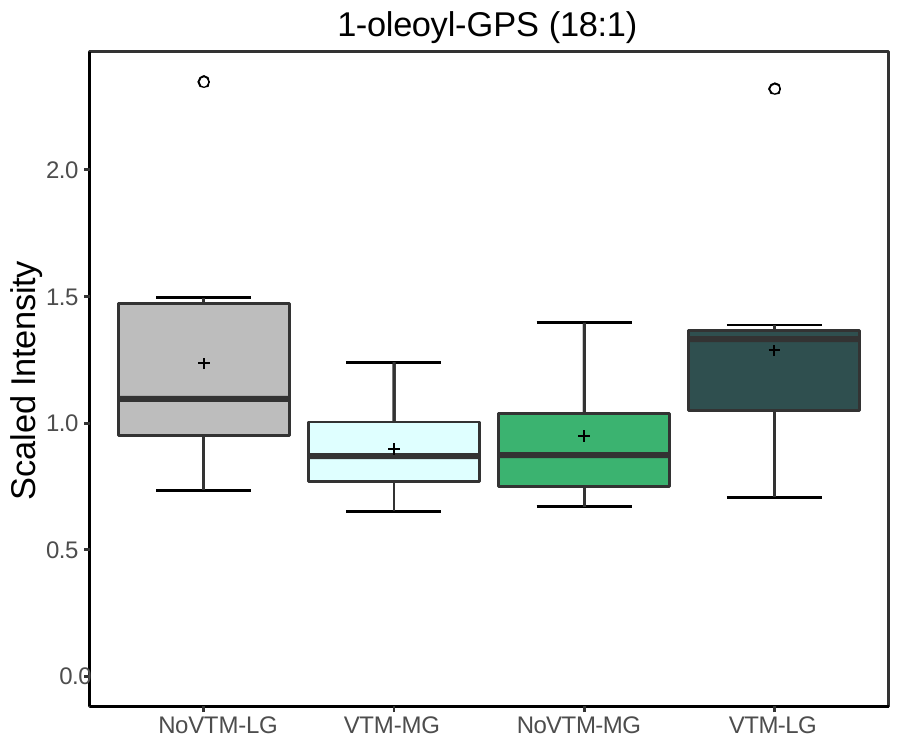

1-oleoyl-GPS (18:1)
2.0
Scaled Intensity
1.5
1.0
0.5
0.0
NoVTM-LG
VTM-MG
NoVTM-MG
VTM-LG

## Slide 238
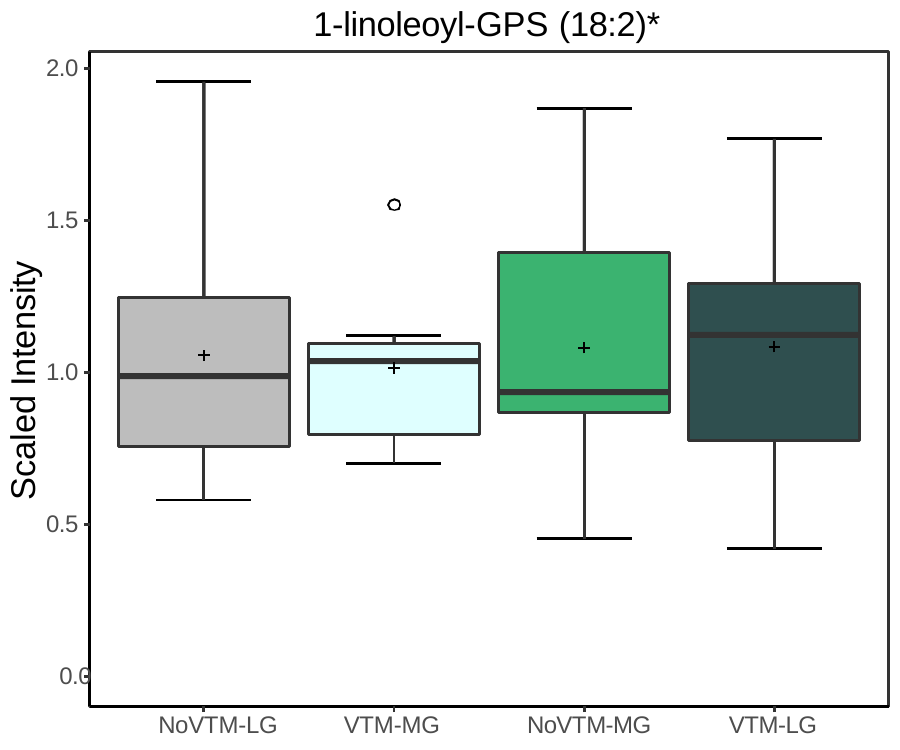

1-linoleoyl-GPS (18:2)*
2.0
1.5
Scaled Intensity
1.0
0.5
0.0
NoVTM-LG
VTM-MG
NoVTM-MG
VTM-LG

## Slide 239
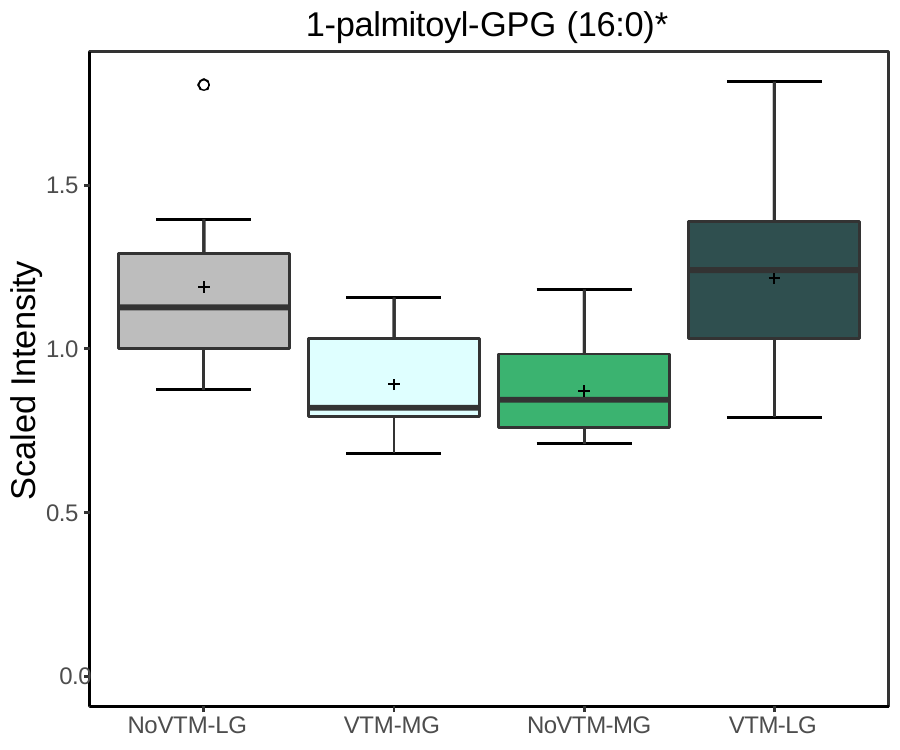

1-palmitoyl-GPG (16:0)*
1.5
Scaled Intensity
1.0
0.5
0.0
NoVTM-LG
VTM-MG
NoVTM-MG
VTM-LG

## Slide 240
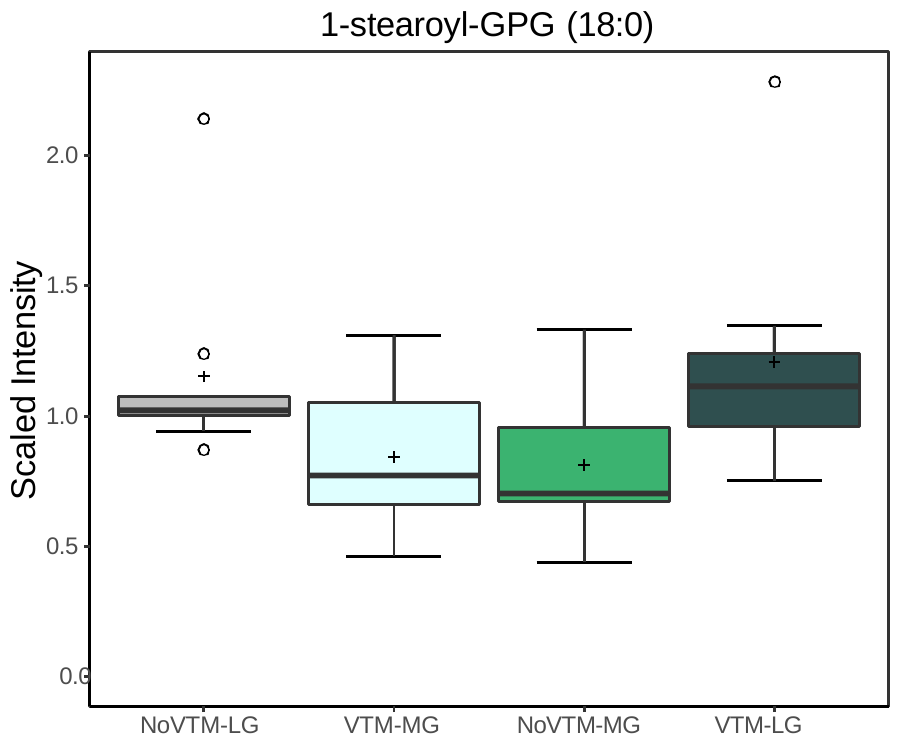

1-stearoyl-GPG (18:0)
2.0
Scaled Intensity
1.5
1.0
0.5
0.0
NoVTM-LG
VTM-MG
NoVTM-MG
VTM-LG

## Slide 241
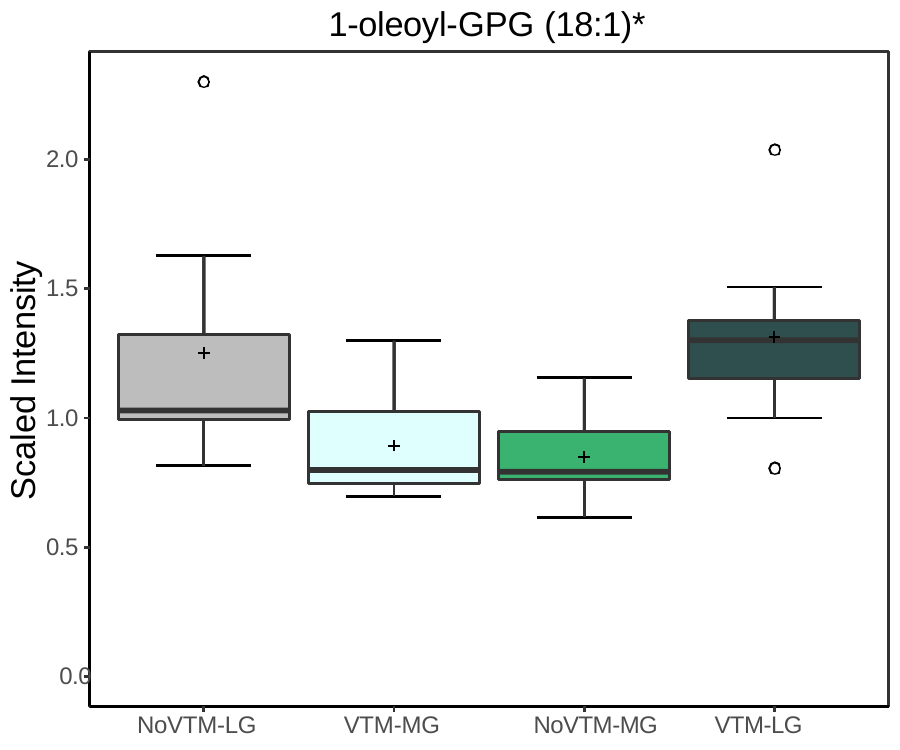

1-oleoyl-GPG (18:1)*
2.0
Scaled Intensity
1.5
1.0
0.5
0.0
NoVTM-LG
VTM-MG
NoVTM-MG
VTM-LG

## Slide 242
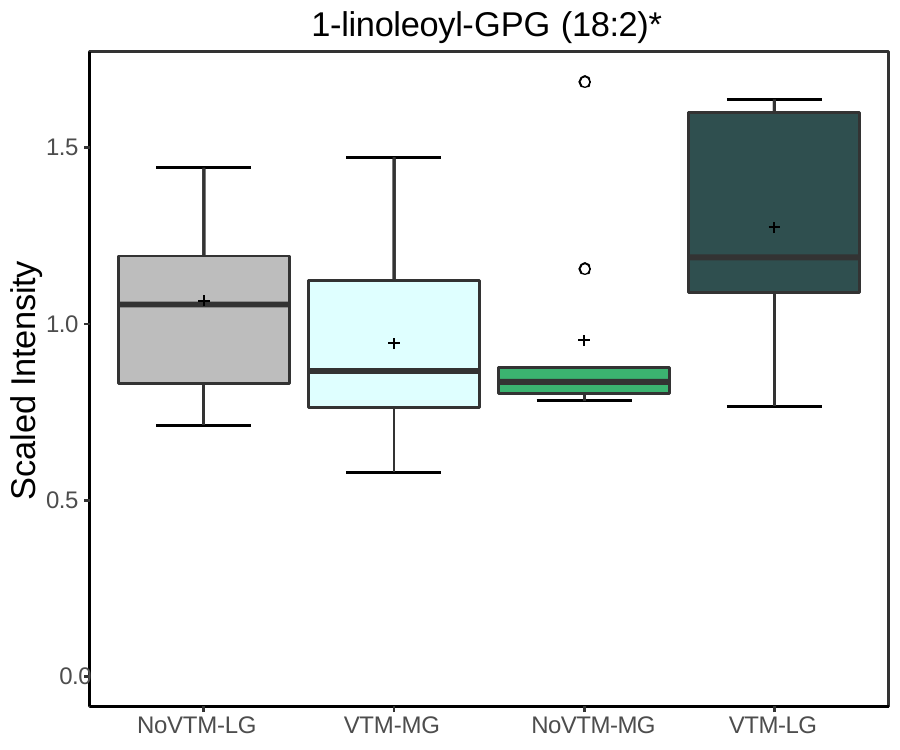

1-linoleoyl-GPG (18:2)*
1.5
Scaled Intensity
1.0
0.5
0.0
NoVTM-LG
VTM-MG
NoVTM-MG
VTM-LG

## Slide 243
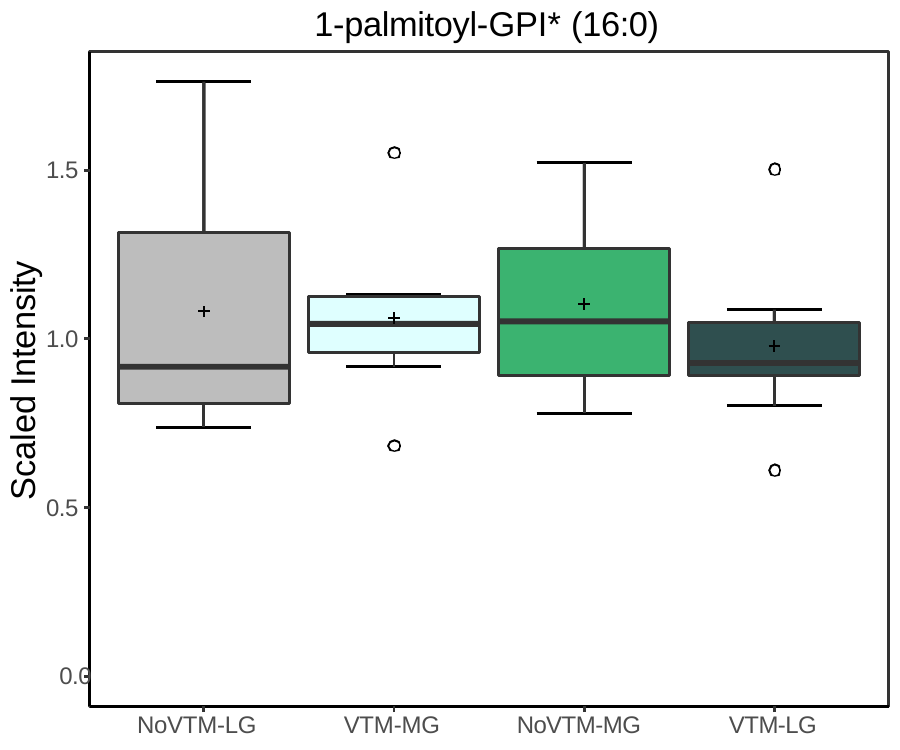

1-palmitoyl-GPI* (16:0)
1.5
Scaled Intensity
1.0
0.5
0.0
NoVTM-LG
VTM-MG
NoVTM-MG
VTM-LG

## Slide 244
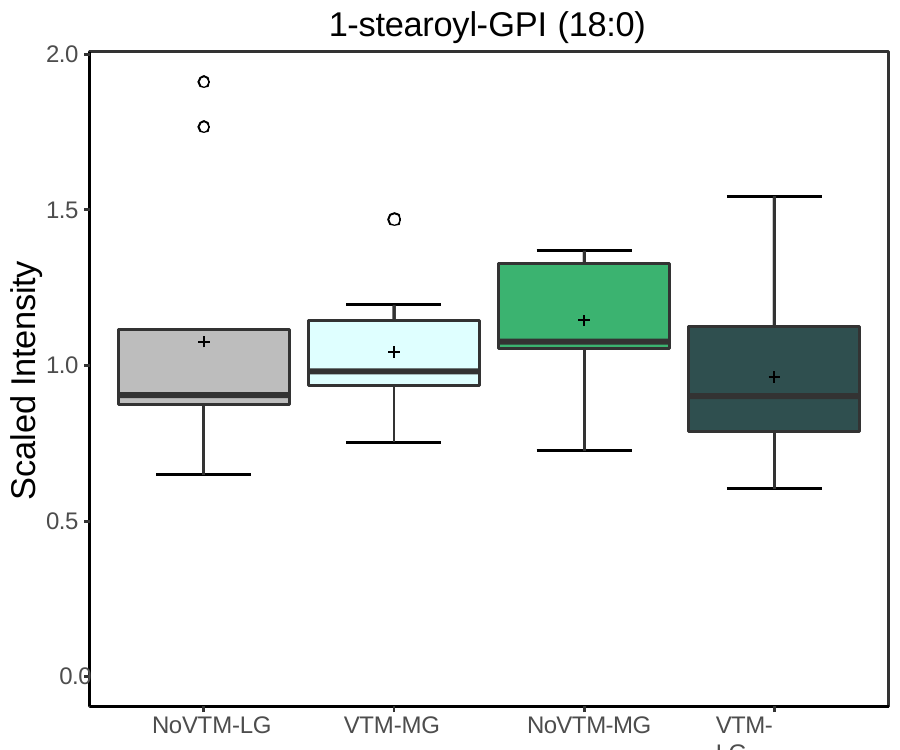

1-stearoyl-GPI (18:0)
2.0
1.5
Scaled Intensity
1.0
0.5
0.0
NoVTM-LG
VTM-MG
NoVTM-MG
VTM-LG

## Slide 245
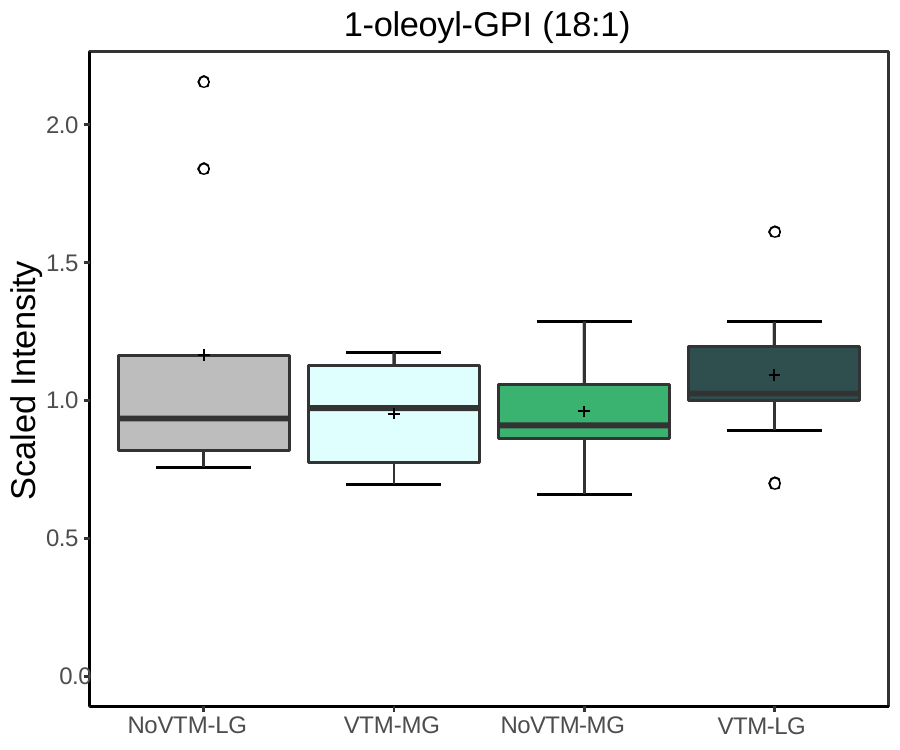

1-oleoyl-GPI (18:1)
2.0
1.5
Scaled Intensity
1.0
0.5
0.0
NoVTM-LG
VTM-MG
NoVTM-MG
VTM-LG

## Slide 246
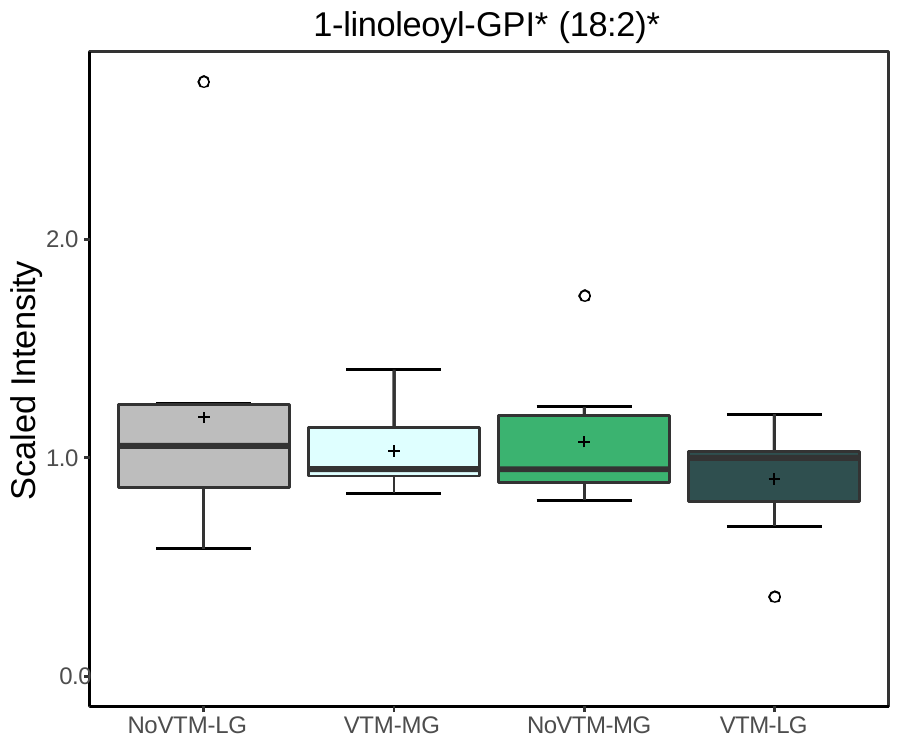

1-linoleoyl-GPI* (18:2)*
2.0
Scaled Intensity
1.0
0.0
NoVTM-LG
VTM-MG
NoVTM-MG
VTM-LG

## Slide 247
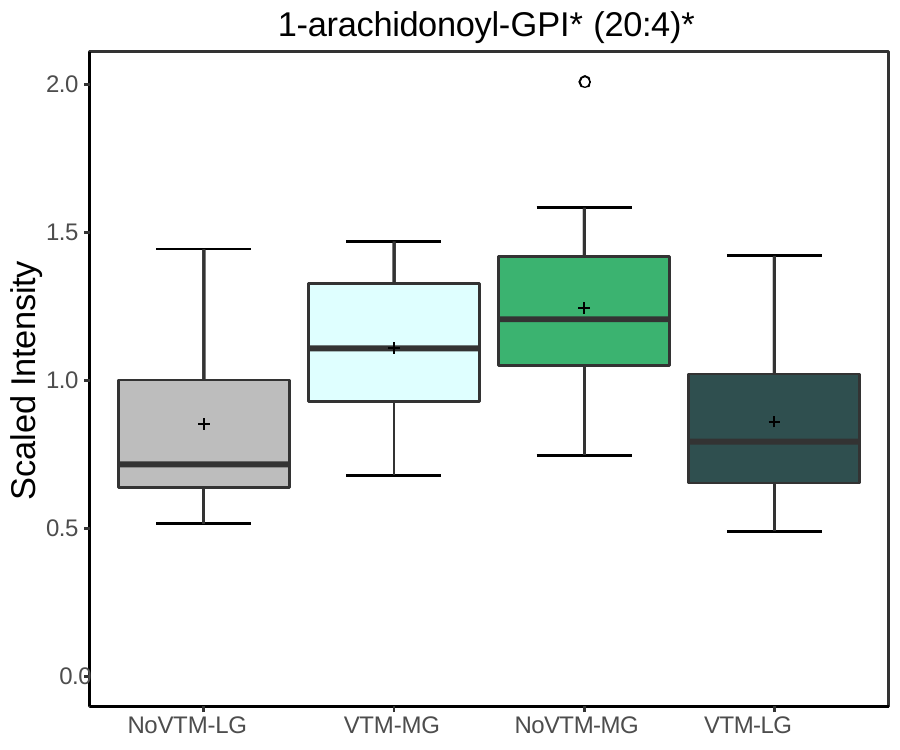

1-arachidonoyl-GPI* (20:4)*
2.0
1.5
Scaled Intensity
1.0
0.5
0.0
NoVTM-LG
VTM-MG
NoVTM-MG
VTM-LG

## Slide 248
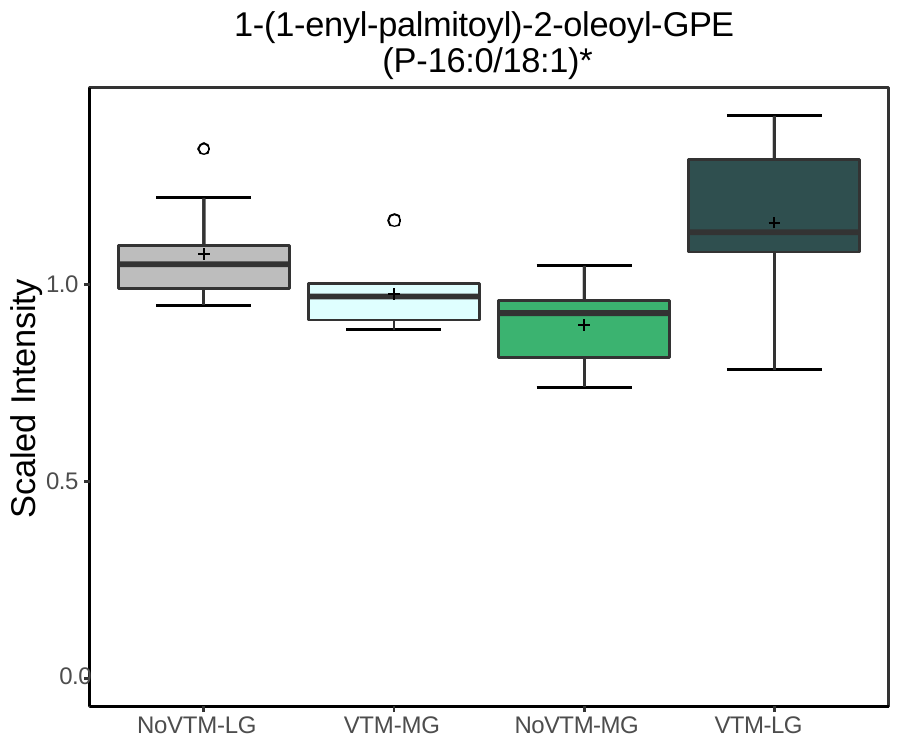

# 1-(1-enyl-palmitoyl)-2-oleoyl-GPE (P-16:0/18:1)*
1.0
Scaled Intensity
0.5
0.0
NoVTM-LG
VTM-MG
NoVTM-MG
VTM-LG

## Slide 249
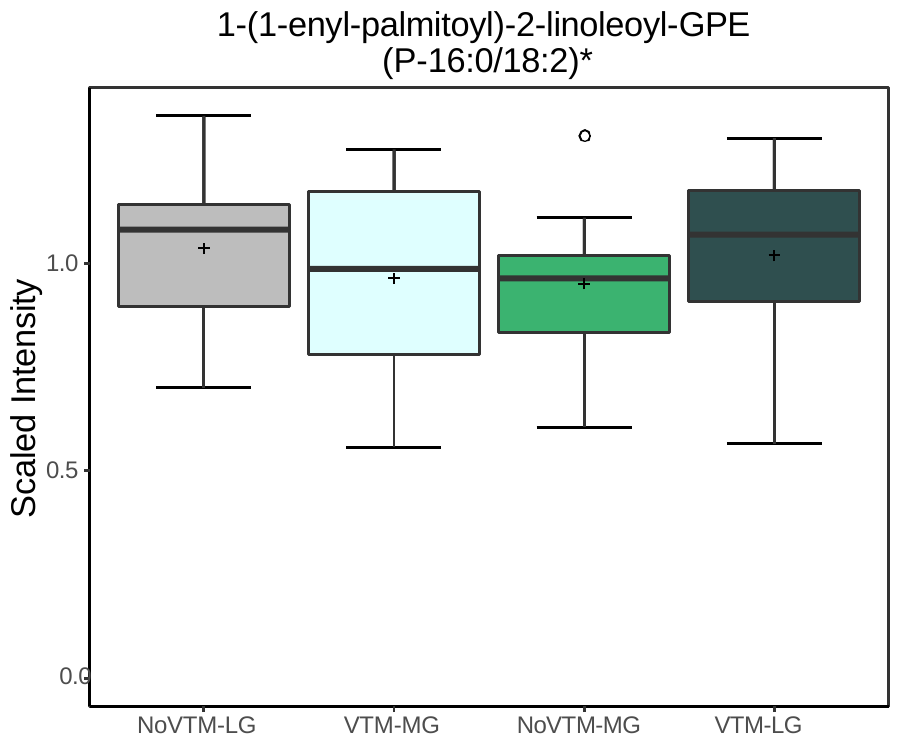

# 1-(1-enyl-palmitoyl)-2-linoleoyl-GPE (P-16:0/18:2)*
1.0
Scaled Intensity
0.5
0.0
NoVTM-LG
VTM-MG
NoVTM-MG
VTM-LG

## Slide 250
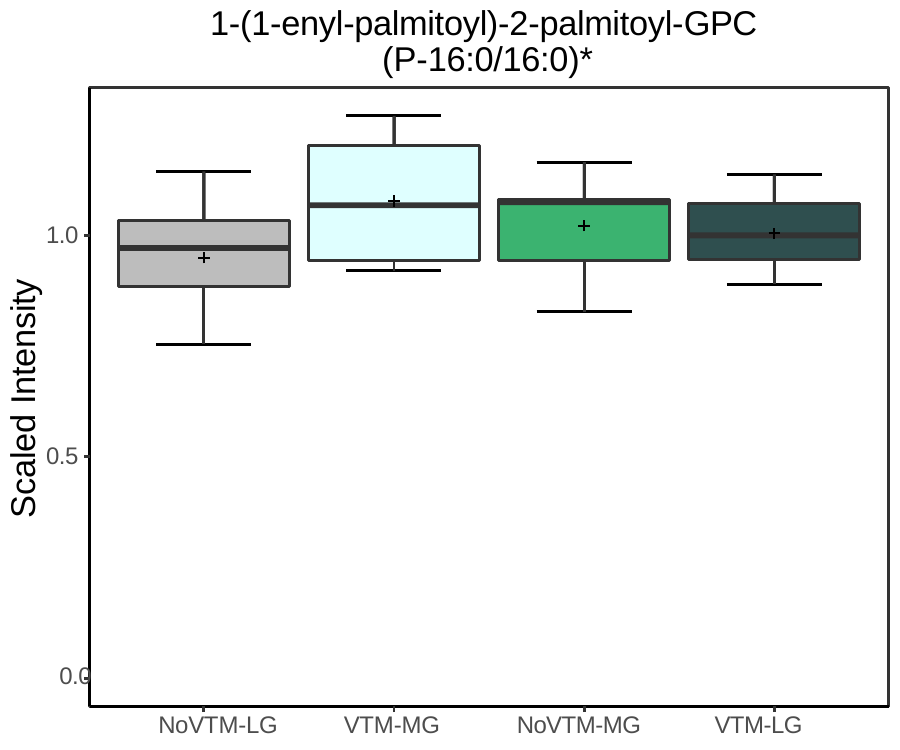

# 1-(1-enyl-palmitoyl)-2-palmitoyl-GPC (P-16:0/16:0)*
1.0
Scaled Intensity
0.5
0.0
NoVTM-LG
VTM-MG
NoVTM-MG
VTM-LG

## Slide 251
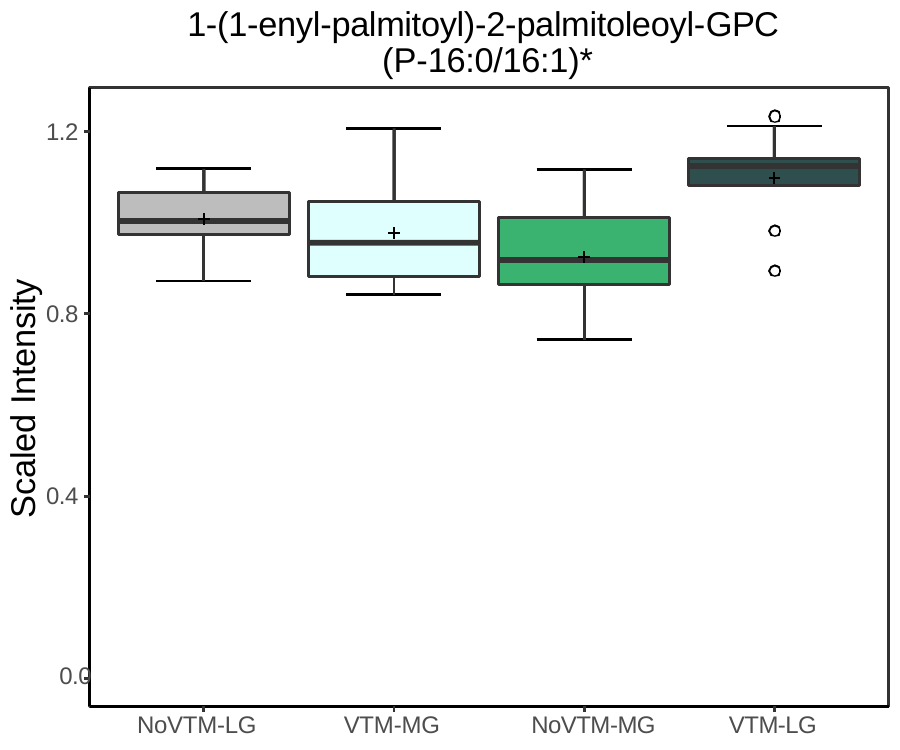

# 1-(1-enyl-palmitoyl)-2-palmitoleoyl-GPC (P-16:0/16:1)*
1.2
Scaled Intensity
0.8
0.4
0.0
NoVTM-LG
VTM-MG
NoVTM-MG
VTM-LG

## Slide 252
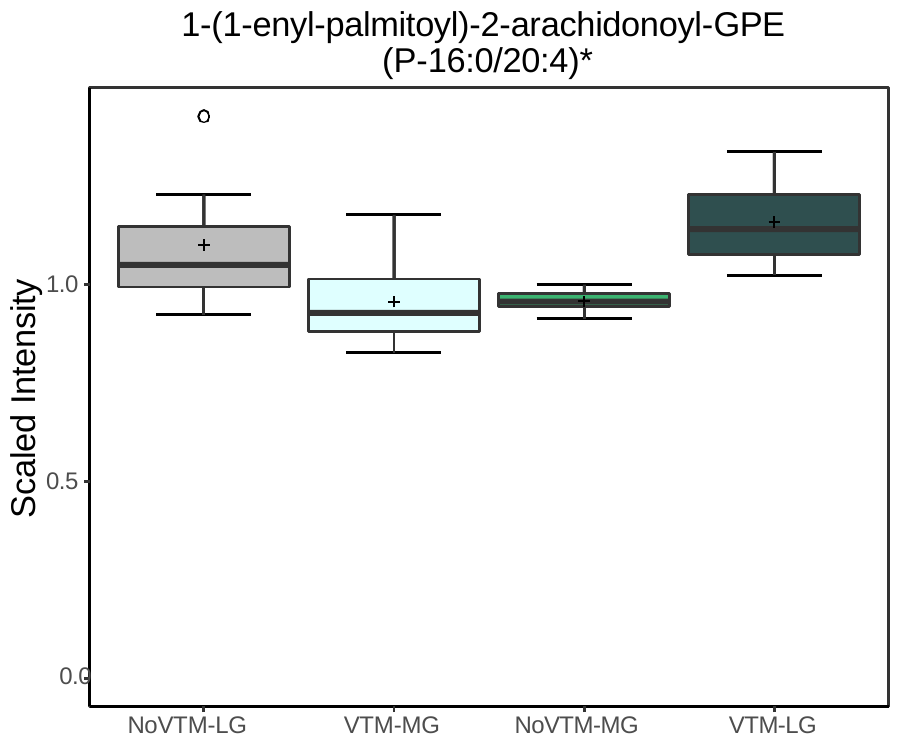

# 1-(1-enyl-palmitoyl)-2-arachidonoyl-GPE (P-16:0/20:4)*
1.0
Scaled Intensity
0.5
0.0
NoVTM-LG
VTM-MG
NoVTM-MG
VTM-LG

## Slide 253
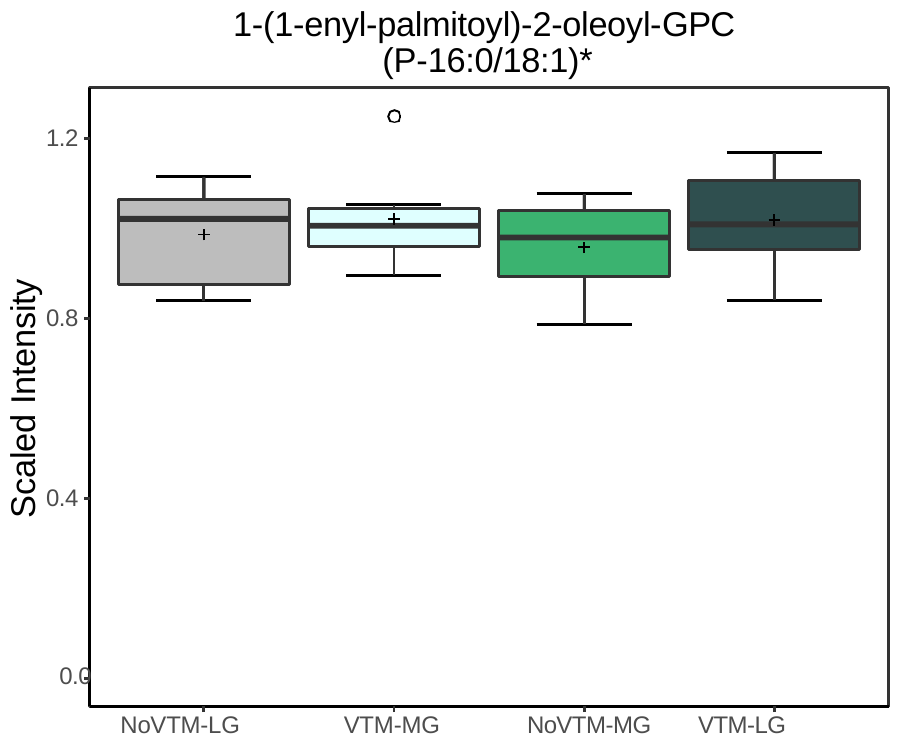

# 1-(1-enyl-palmitoyl)-2-oleoyl-GPC (P-16:0/18:1)*
1.2
Scaled Intensity
0.8
0.4
0.0
NoVTM-LG
VTM-MG
NoVTM-MG
VTM-LG

## Slide 254
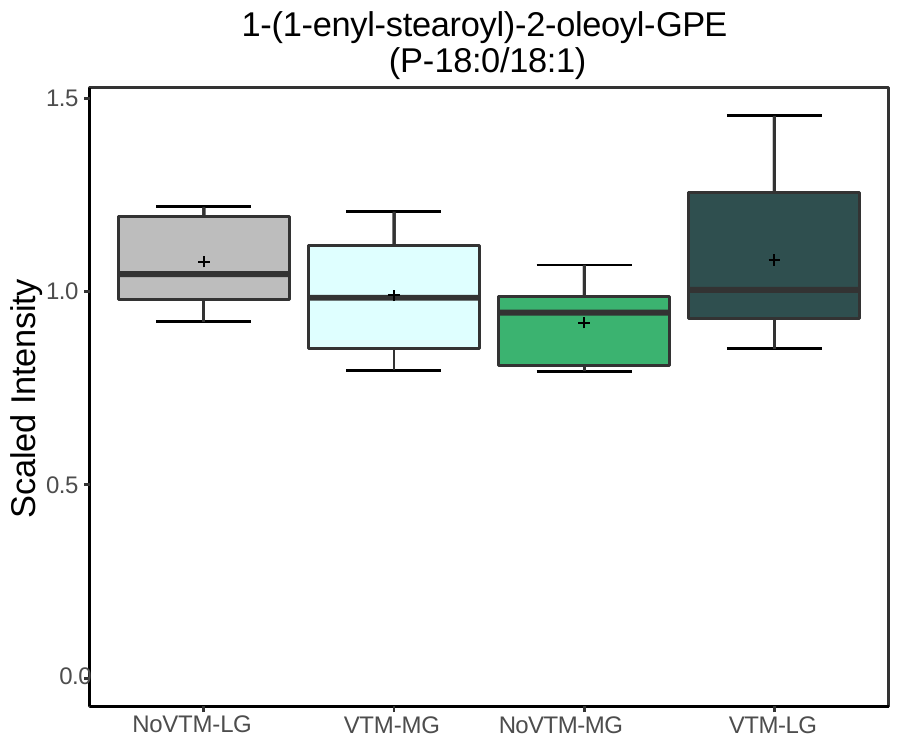

# 1-(1-enyl-stearoyl)-2-oleoyl-GPE (P-18:0/18:1)
1.5
1.0
Scaled Intensity
0.5
0.0
NoVTM-LG
VTM-MG
NoVTM-MG
VTM-LG

## Slide 255
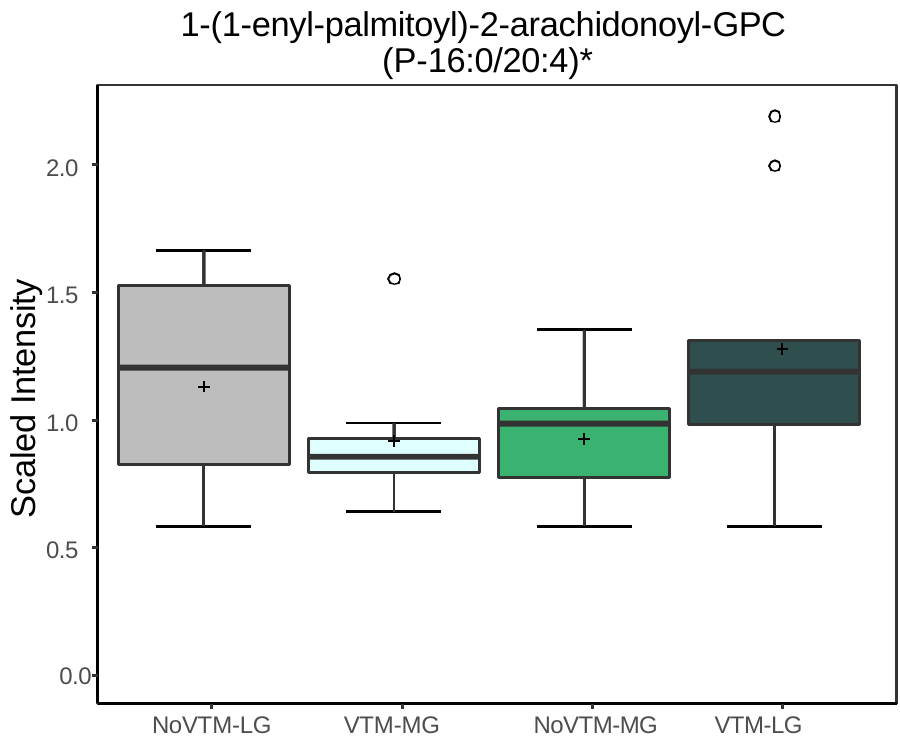

# 1-(1-enyl-palmitoyl)-2-arachidonoyl-GPC (P-16:0/20:4)*
2.0
Scaled Intensity
1.5
1.0
0.5
0.0
NoVTM-MG
NoVTM-LG
VTM-MG
VTM-LG

## Slide 256
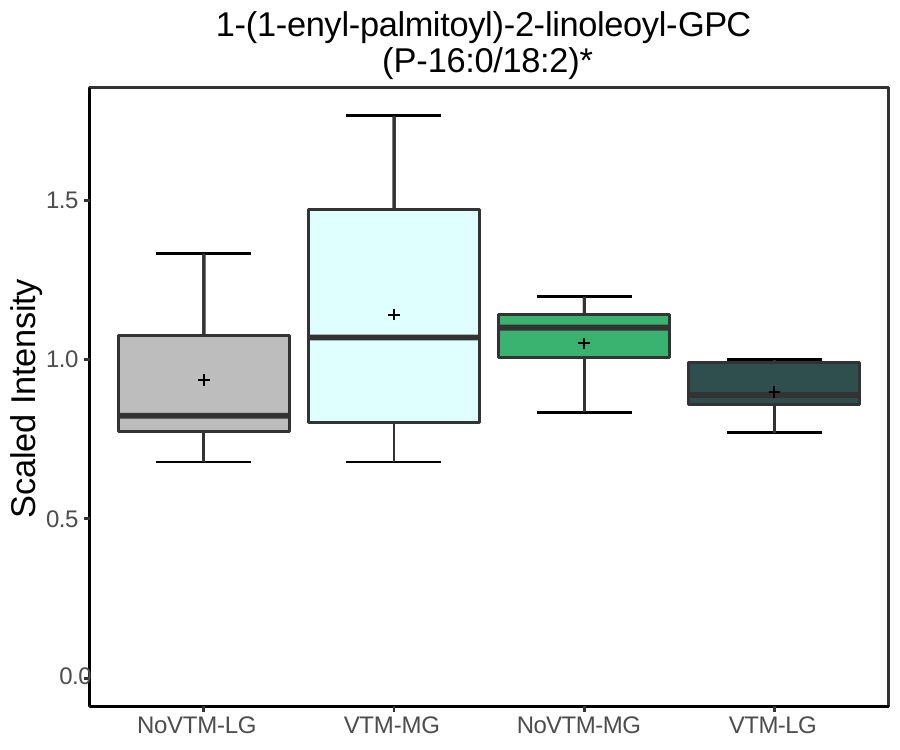

# 1-(1-enyl-palmitoyl)-2-linoleoyl-GPC (P-16:0/18:2)*
1.5
Scaled Intensity
1.0
0.5
0.0
NoVTM-LG
VTM-MG
NoVTM-MG
VTM-LG

## Slide 257
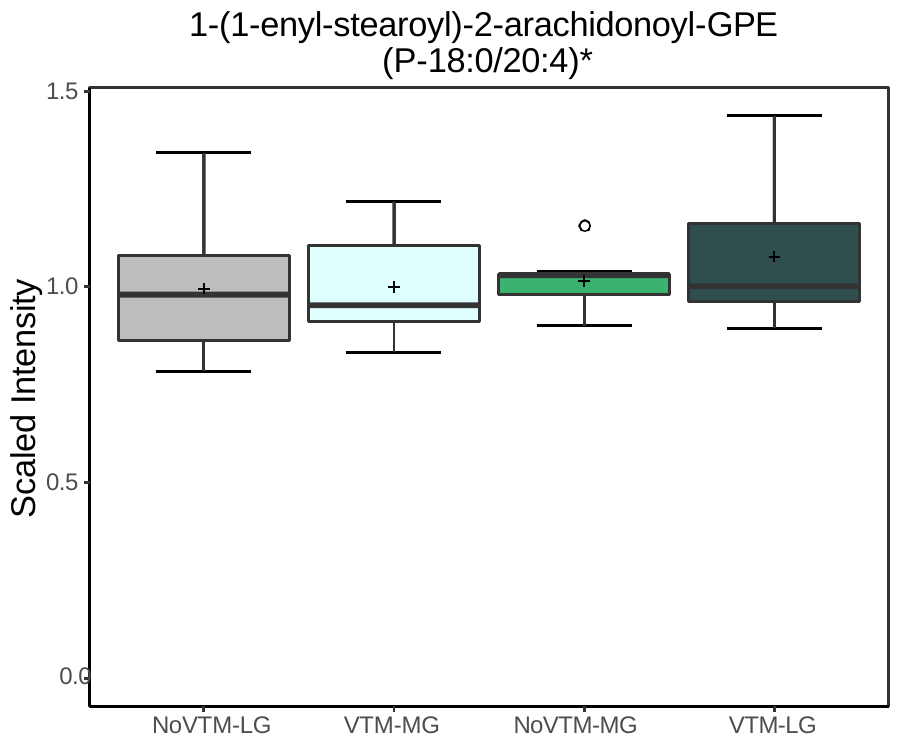

# 1-(1-enyl-stearoyl)-2-arachidonoyl-GPE (P-18:0/20:4)*
1.5
1.0
Scaled Intensity
0.5
0.0
NoVTM-LG
VTM-MG
NoVTM-MG
VTM-LG

## Slide 258
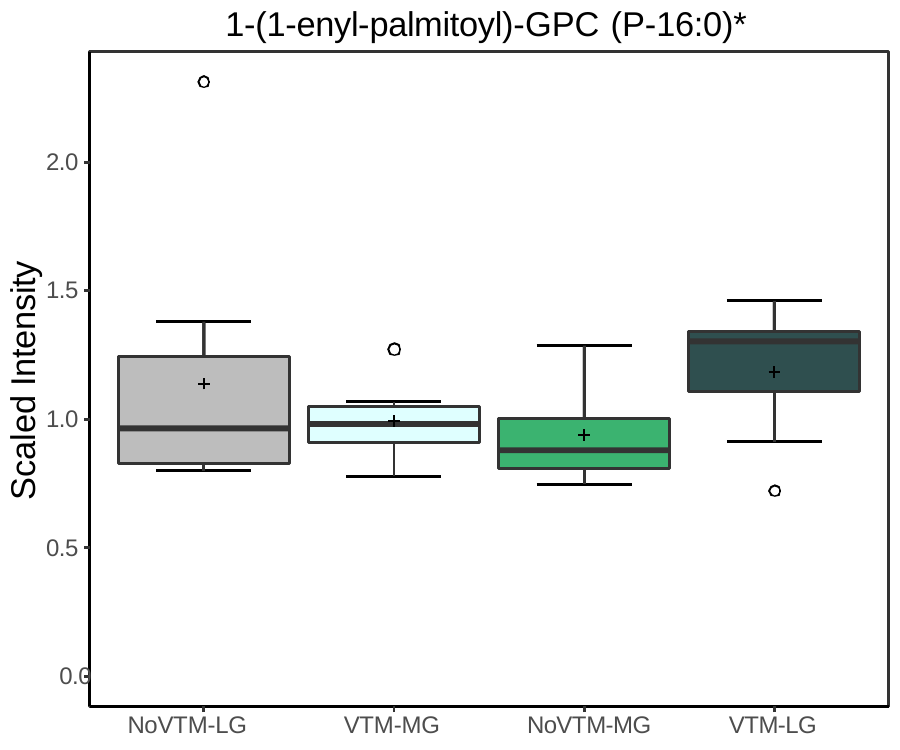

1-(1-enyl-palmitoyl)-GPC (P-16:0)*
2.0
Scaled Intensity
1.5
1.0
0.5
0.0
NoVTM-LG
VTM-MG
NoVTM-MG
VTM-LG

## Slide 259
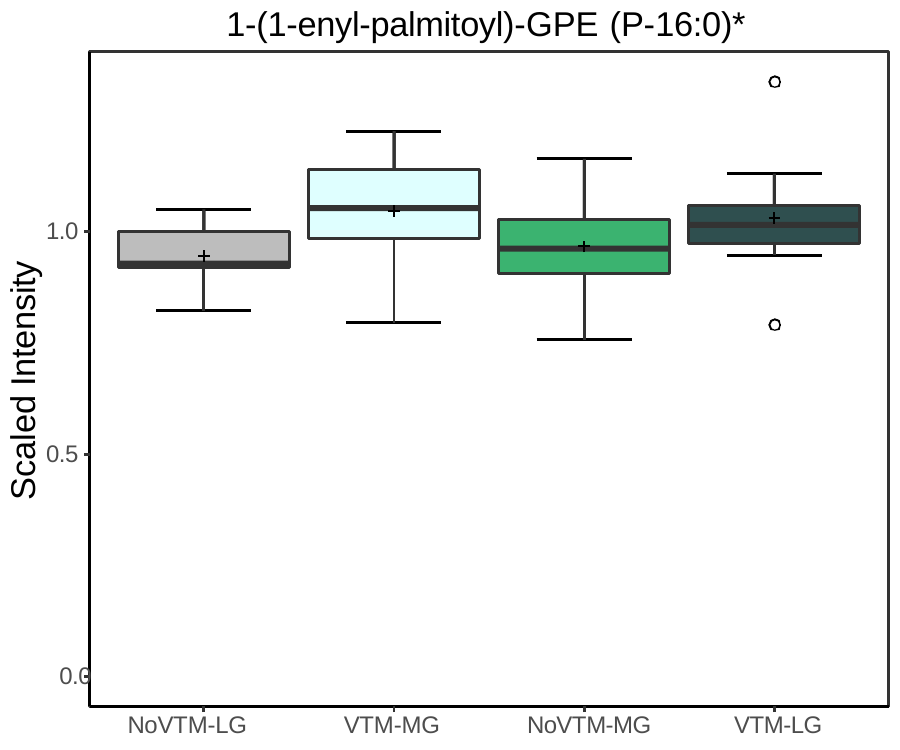

1-(1-enyl-palmitoyl)-GPE (P-16:0)*
1.0
Scaled Intensity
0.5
0.0
NoVTM-LG
VTM-MG
NoVTM-MG
VTM-LG

## Slide 260
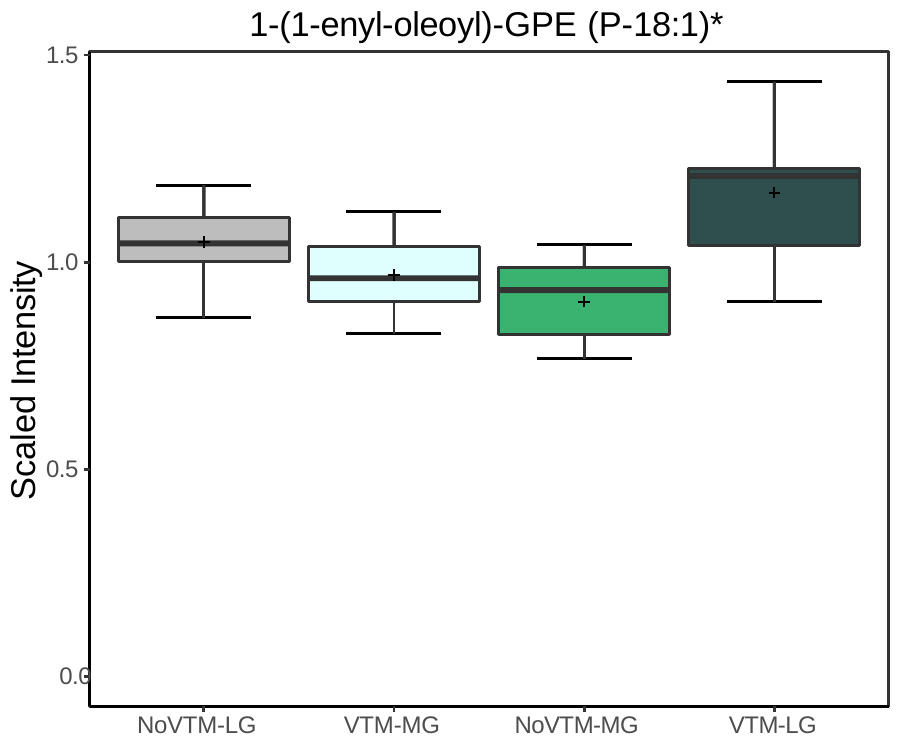

1-(1-enyl-oleoyl)-GPE (P-18:1)*
1.5
1.0
Scaled Intensity
0.5
0.0
NoVTM-LG
VTM-MG
NoVTM-MG
VTM-LG

## Slide 261
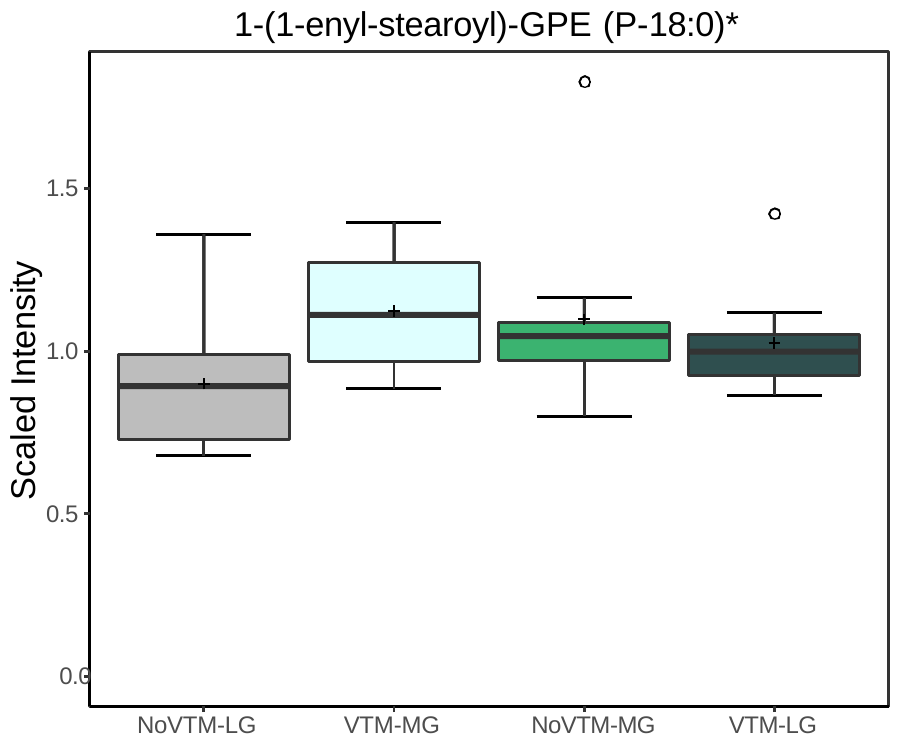

1-(1-enyl-stearoyl)-GPE (P-18:0)*
1.5
Scaled Intensity
1.0
0.5
0.0
NoVTM-LG
VTM-MG
NoVTM-MG
VTM-LG

## Slide 262
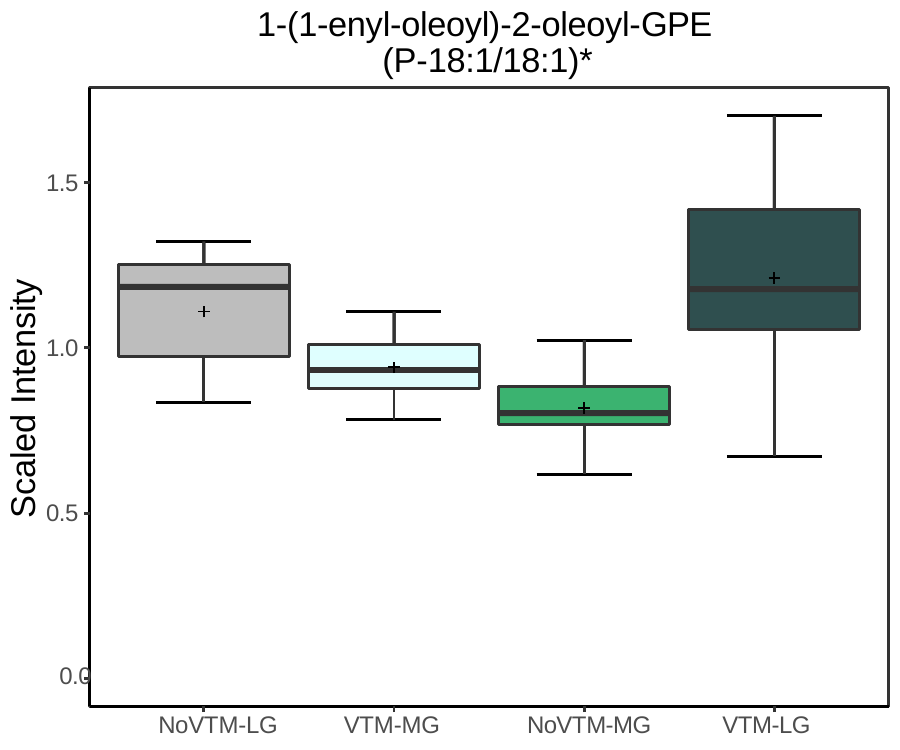

# 1-(1-enyl-oleoyl)-2-oleoyl-GPE (P-18:1/18:1)*
1.5
Scaled Intensity
1.0
0.5
0.0
NoVTM-LG
VTM-MG
NoVTM-MG
VTM-LG

## Slide 263
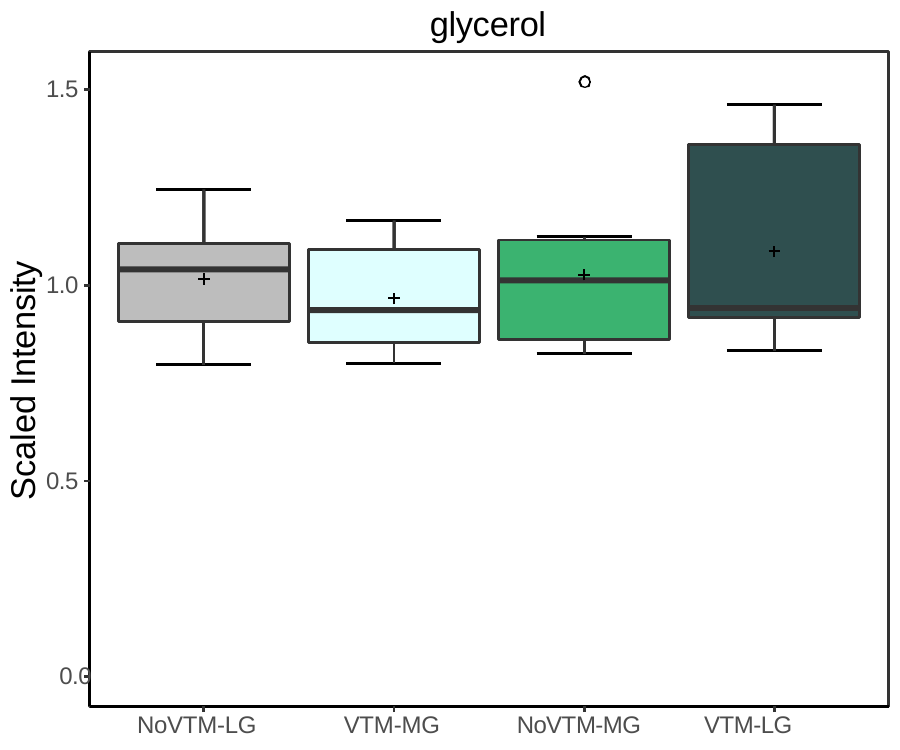

glycerol
1.5
Scaled Intensity
1.0
0.5
0.0
NoVTM-LG
VTM-MG
NoVTM-MG
VTM-LG

## Slide 264
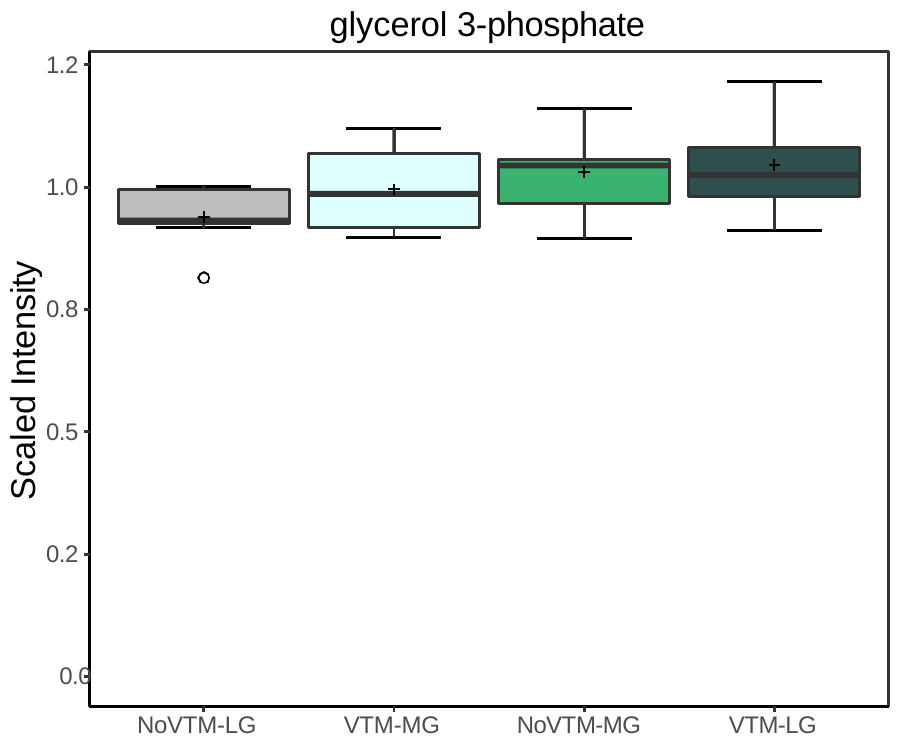

glycerol 3-phosphate
1.2
1.0
Scaled Intensity
0.8
0.5
0.2
0.0
NoVTM-LG
VTM-MG
NoVTM-MG
VTM-LG

## Slide 265
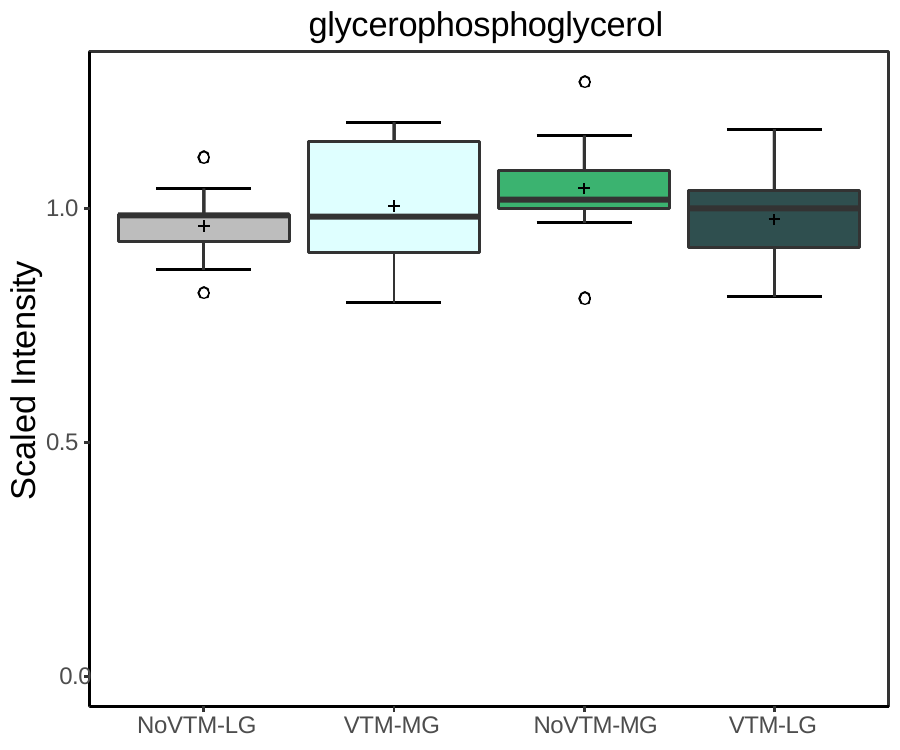

glycerophosphoglycerol
1.0
Scaled Intensity
0.5
0.0
NoVTM-LG
VTM-MG
NoVTM-MG
VTM-LG

## Slide 266
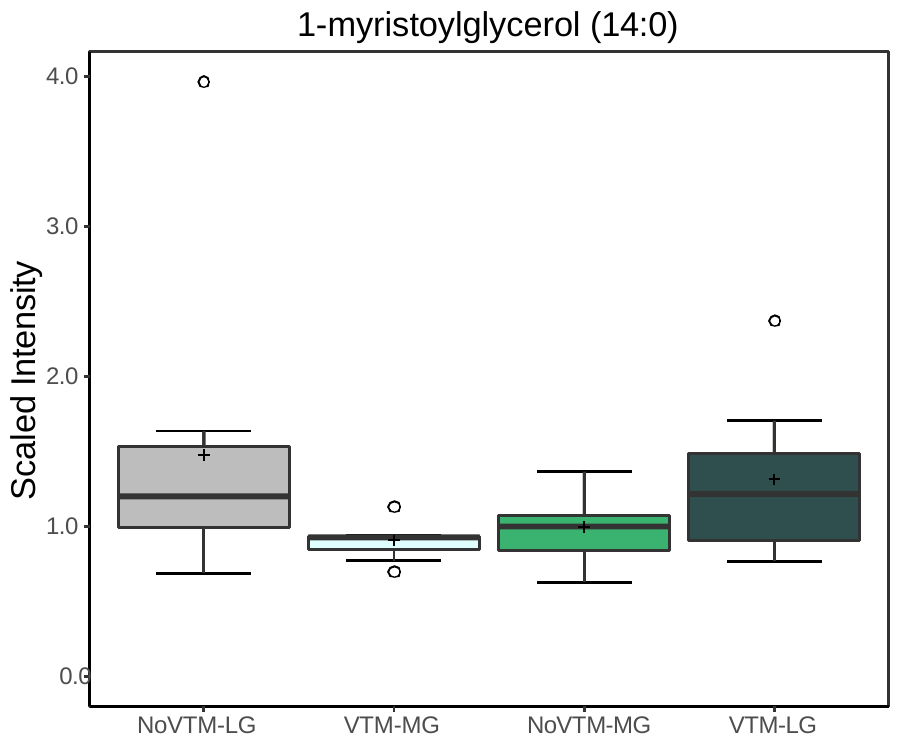

1-myristoylglycerol (14:0)
4.0
3.0
Scaled Intensity
2.0
1.0
0.0
NoVTM-LG
VTM-MG
NoVTM-MG
VTM-LG

## Slide 267
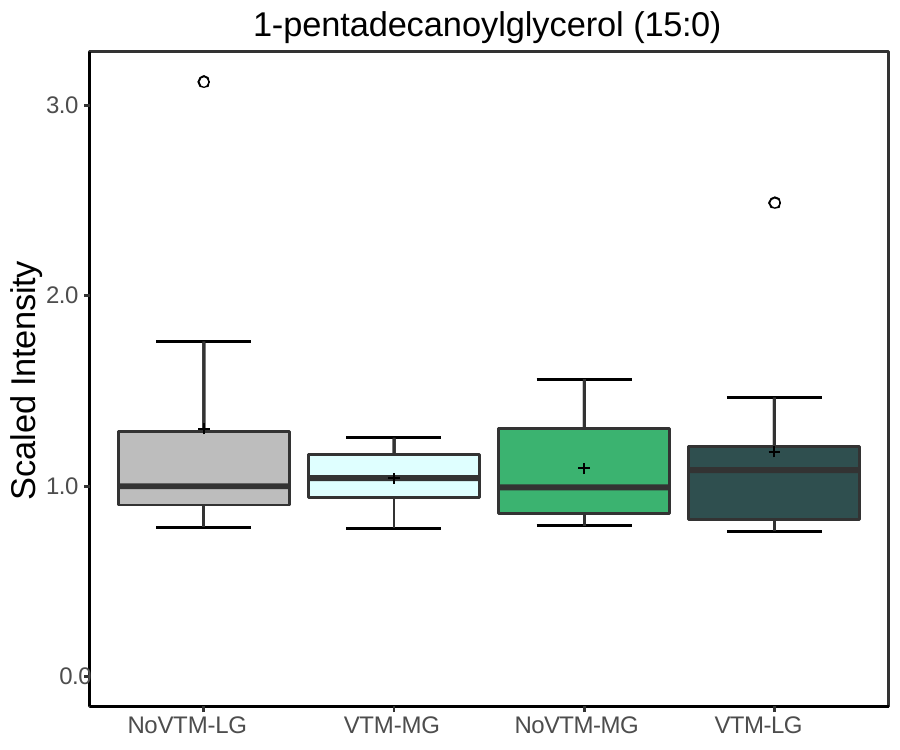

1-pentadecanoylglycerol (15:0)
3.0
Scaled Intensity
2.0
1.0
0.0
NoVTM-LG
VTM-MG
NoVTM-MG
VTM-LG

## Slide 268
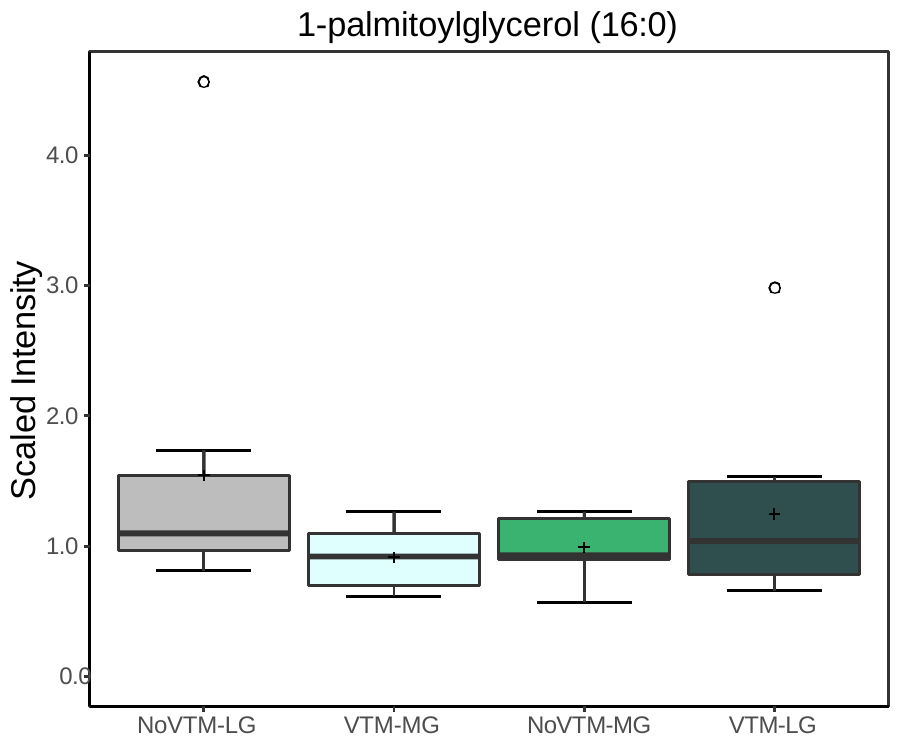

1-palmitoylglycerol (16:0)
4.0
Scaled Intensity
3.0
2.0
1.0
0.0
NoVTM-LG
VTM-MG
NoVTM-MG
VTM-LG

## Slide 269
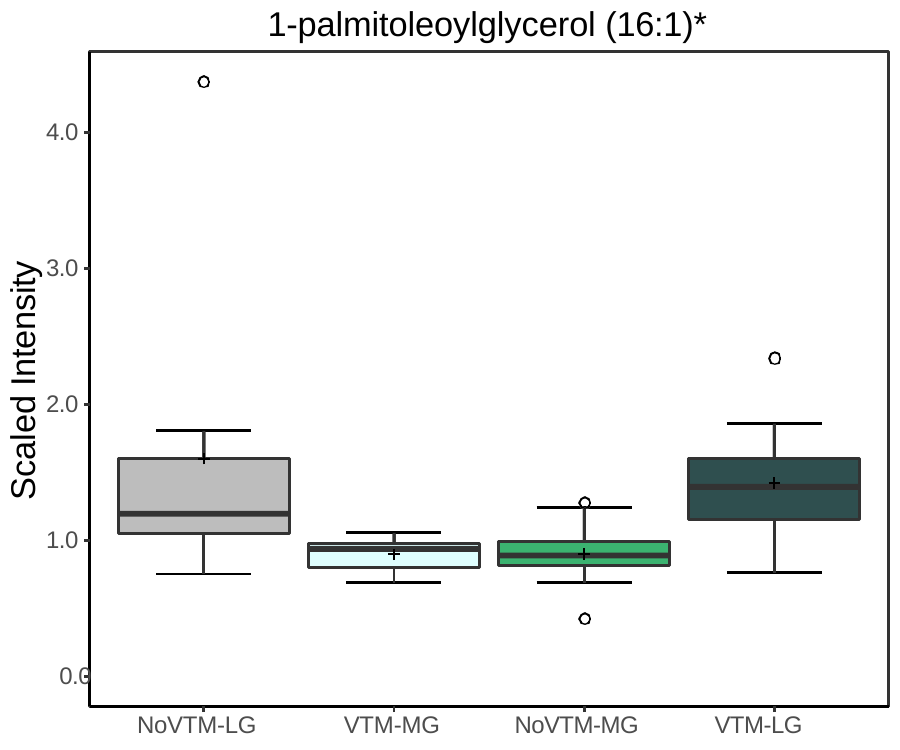

1-palmitoleoylglycerol (16:1)*
4.0
3.0
Scaled Intensity
2.0
1.0
0.0
NoVTM-LG
VTM-MG
NoVTM-MG
VTM-LG

## Slide 270
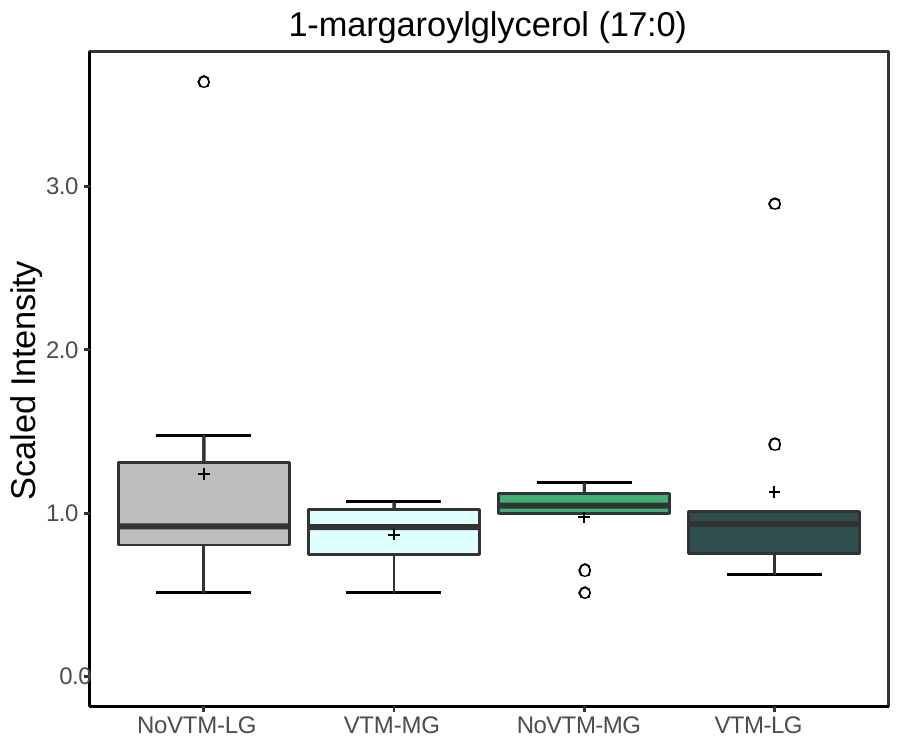

1-margaroylglycerol (17:0)
3.0
Scaled Intensity
2.0
1.0
0.0
NoVTM-LG
VTM-MG
NoVTM-MG
VTM-LG

## Slide 271
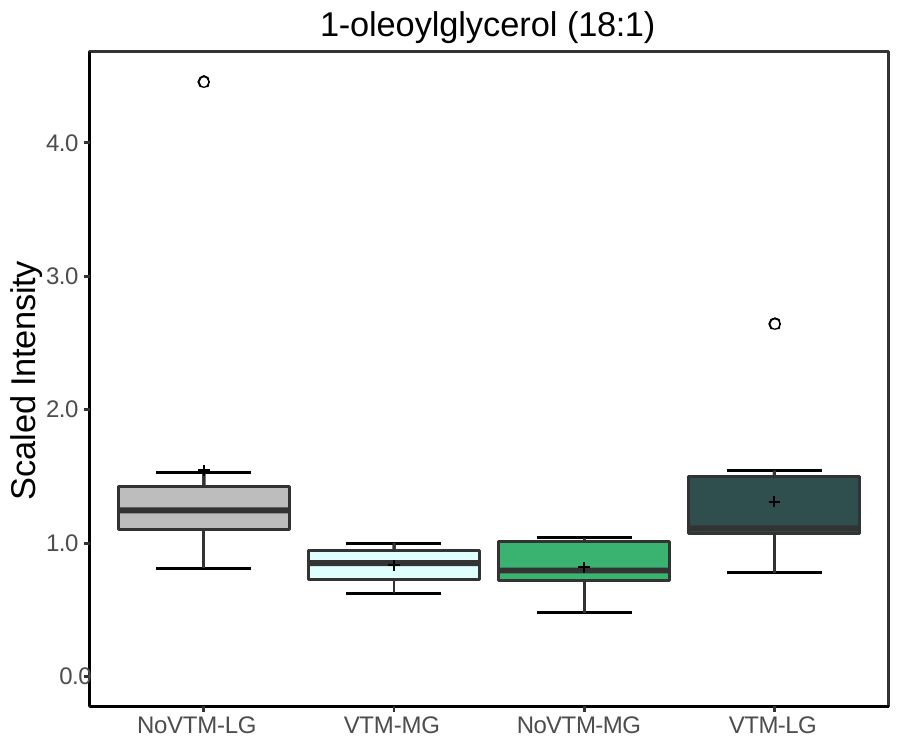

1-oleoylglycerol (18:1)
4.0
Scaled Intensity
3.0
2.0
1.0
0.0
NoVTM-LG
VTM-MG
NoVTM-MG
VTM-LG

## Slide 272
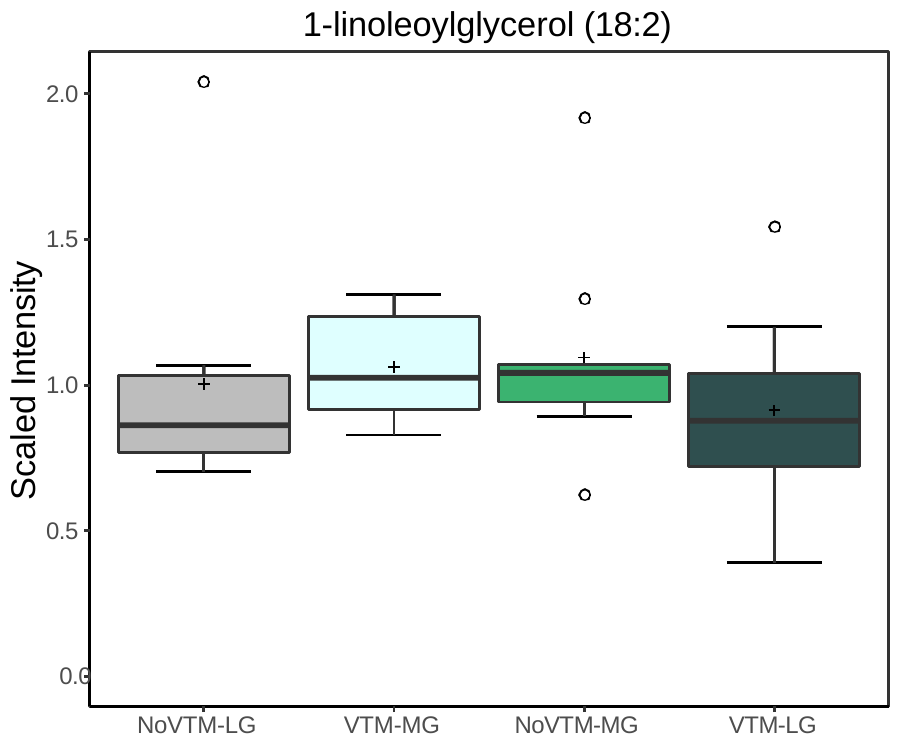

1-linoleoylglycerol (18:2)
2.0
1.5
Scaled Intensity
1.0
0.5
0.0
NoVTM-LG
VTM-MG
NoVTM-MG
VTM-LG

## Slide 273
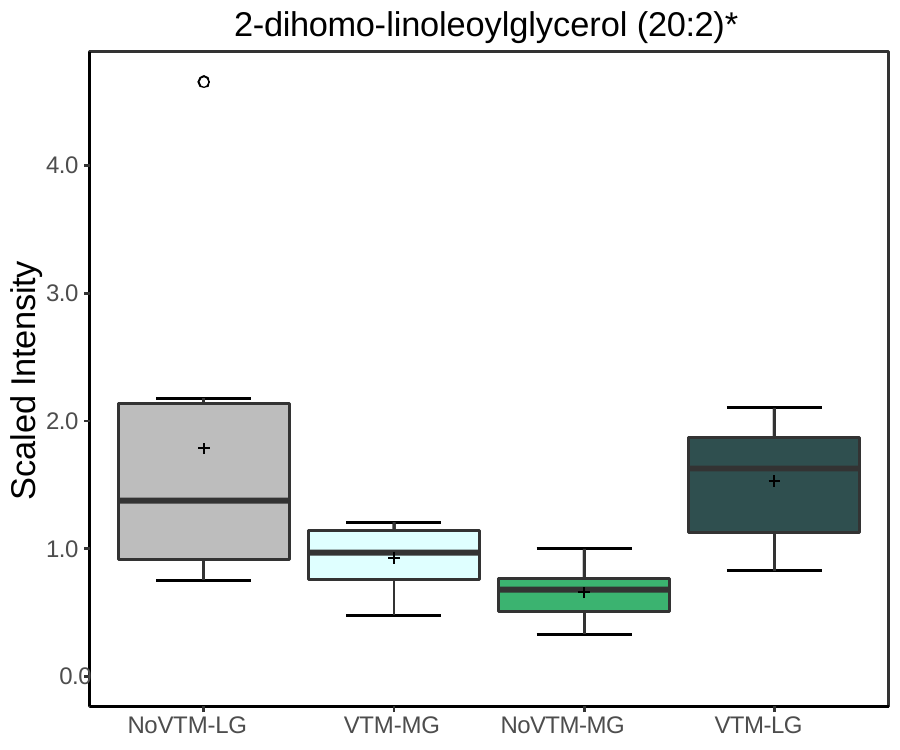

2-dihomo-linoleoylglycerol (20:2)*
4.0
Scaled Intensity
3.0
2.0
1.0
0.0
NoVTM-LG
VTM-MG
NoVTM-MG
VTM-LG

## Slide 274
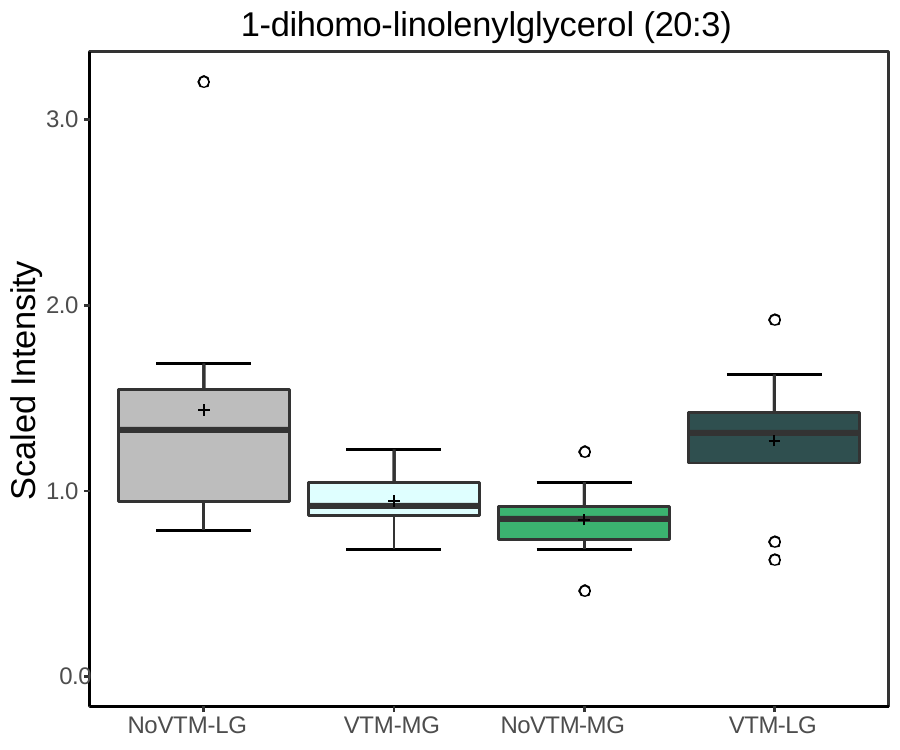

1-dihomo-linolenylglycerol (20:3)
3.0
Scaled Intensity
2.0
1.0
0.0
NoVTM-LG
VTM-MG
NoVTM-MG
VTM-LG

## Slide 275
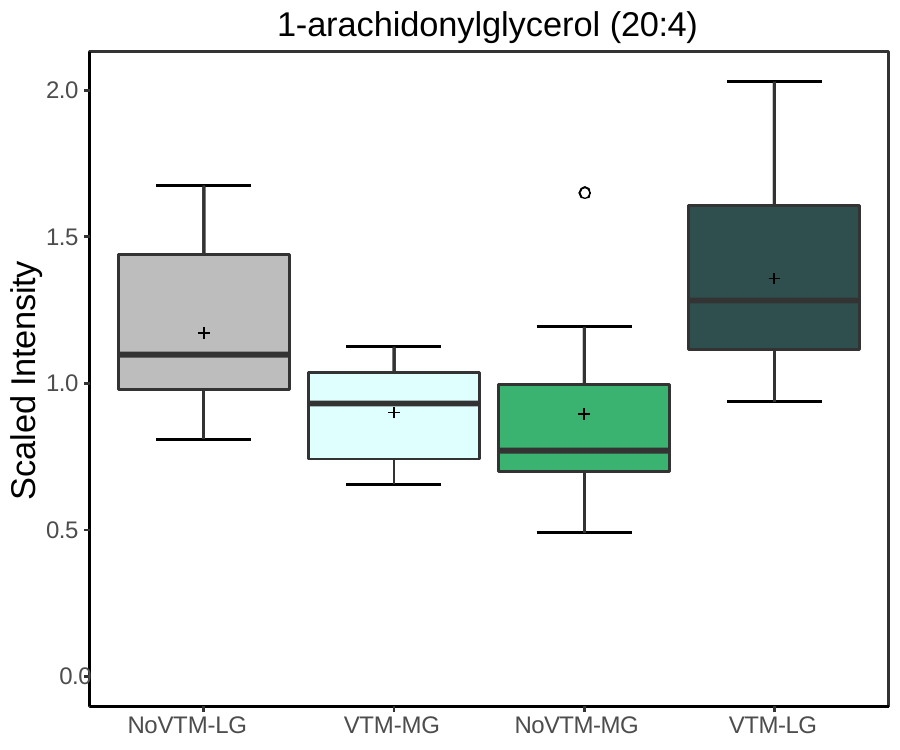

1-arachidonylglycerol (20:4)
2.0
1.5
Scaled Intensity
1.0
0.5
0.0
NoVTM-LG
VTM-MG
NoVTM-MG
VTM-LG

## Slide 276
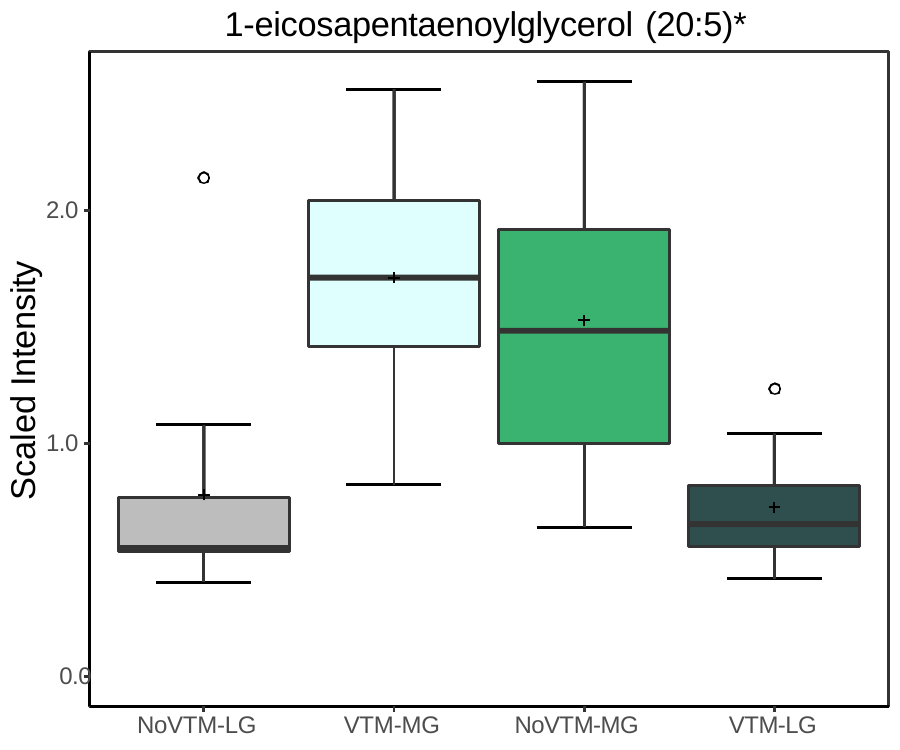

1-eicosapentaenoylglycerol (20:5)*
2.0
Scaled Intensity
1.0
0.0
NoVTM-LG
VTM-MG
NoVTM-MG
VTM-LG

## Slide 277
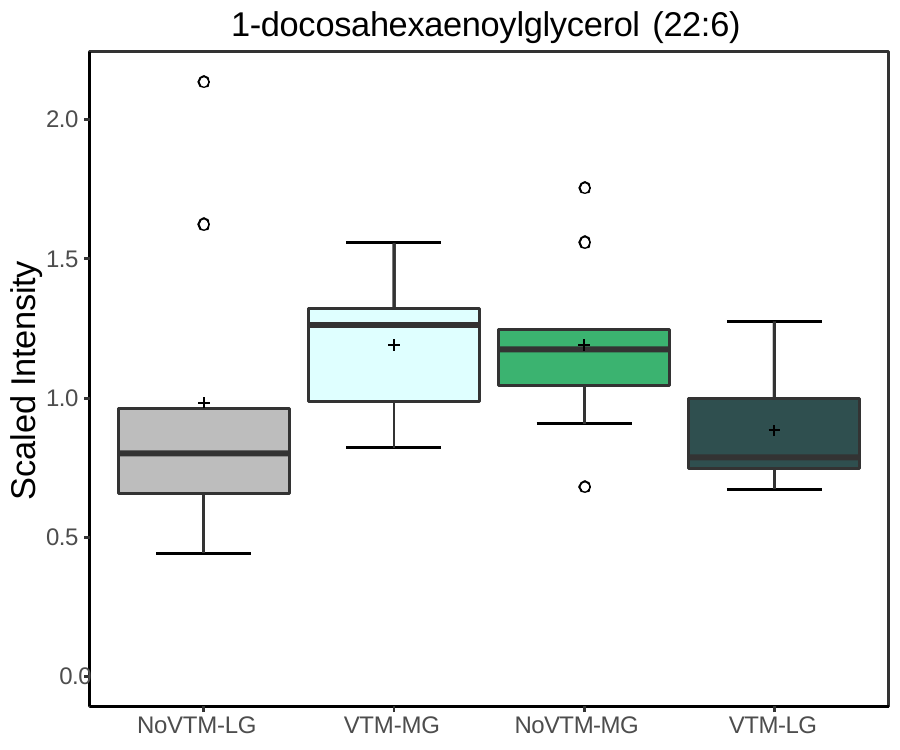

1-docosahexaenoylglycerol (22:6)
2.0
1.5
Scaled Intensity
1.0
0.5
0.0
NoVTM-LG
VTM-MG
NoVTM-MG
VTM-LG

## Slide 278
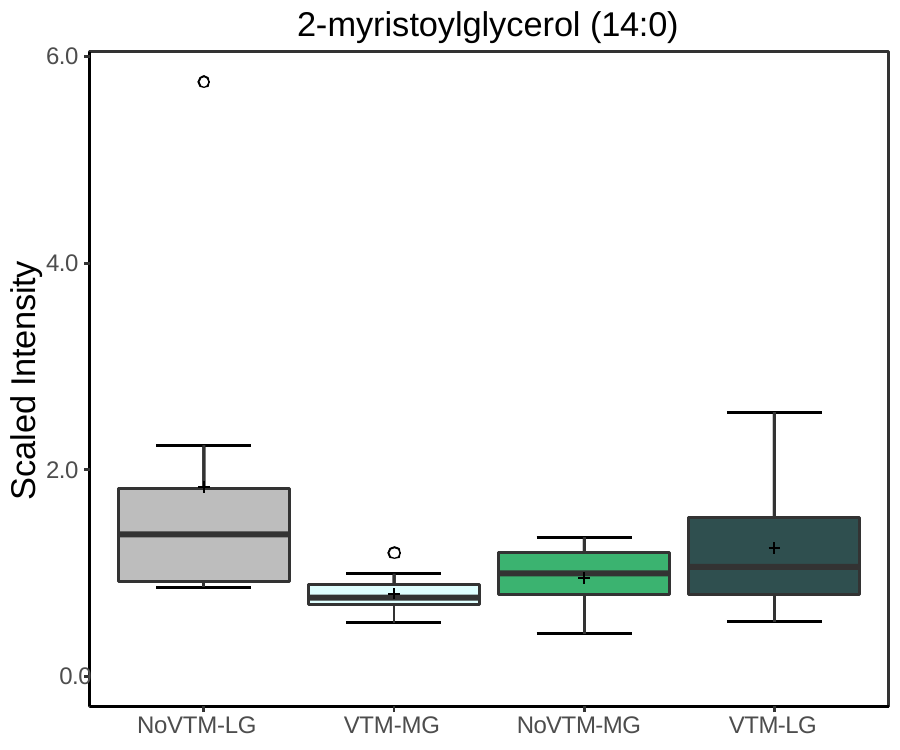

2-myristoylglycerol (14:0)
6.0
4.0
Scaled Intensity
2.0
0.0
NoVTM-LG
VTM-MG
NoVTM-MG
VTM-LG

## Slide 279
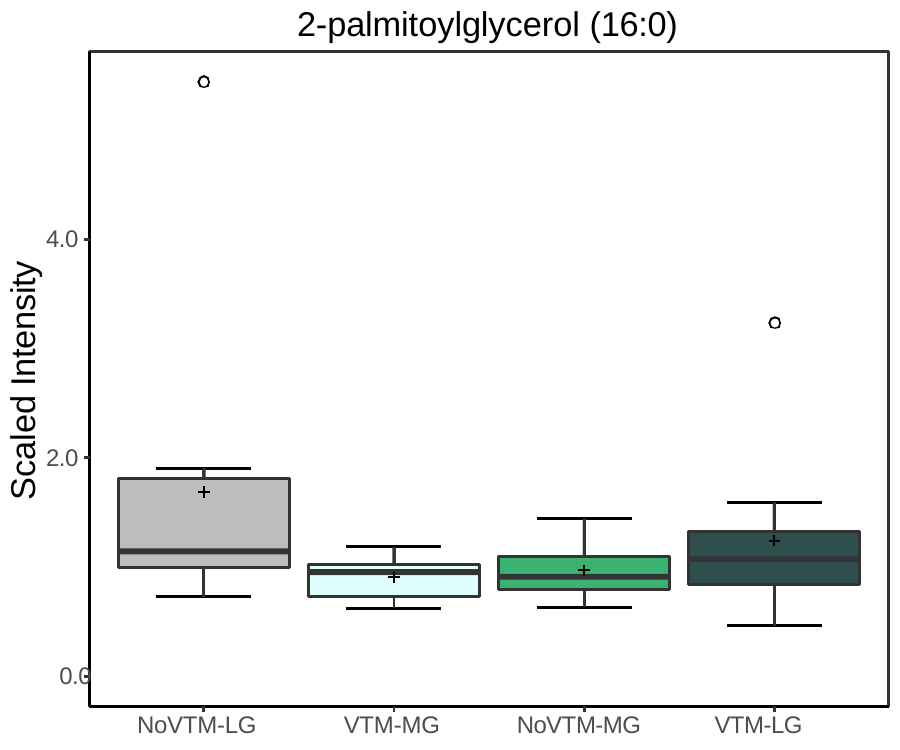

2-palmitoylglycerol (16:0)
4.0
Scaled Intensity
2.0
0.0
NoVTM-LG
VTM-MG
NoVTM-MG
VTM-LG

## Slide 280
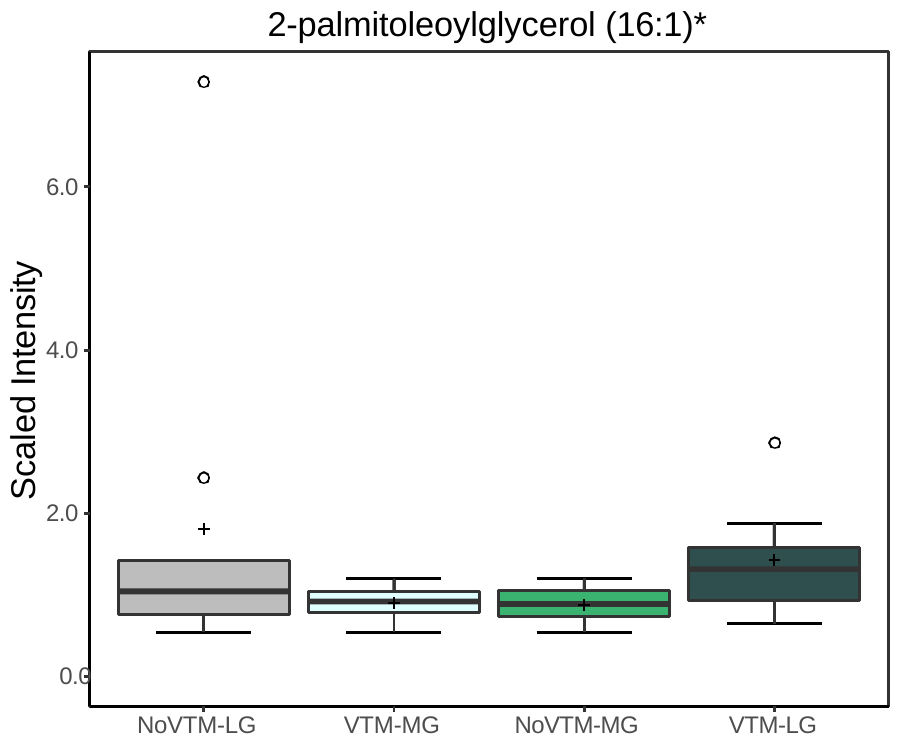

2-palmitoleoylglycerol (16:1)*
6.0
Scaled Intensity
4.0
2.0
0.0
NoVTM-LG
VTM-MG
NoVTM-MG
VTM-LG

## Slide 281
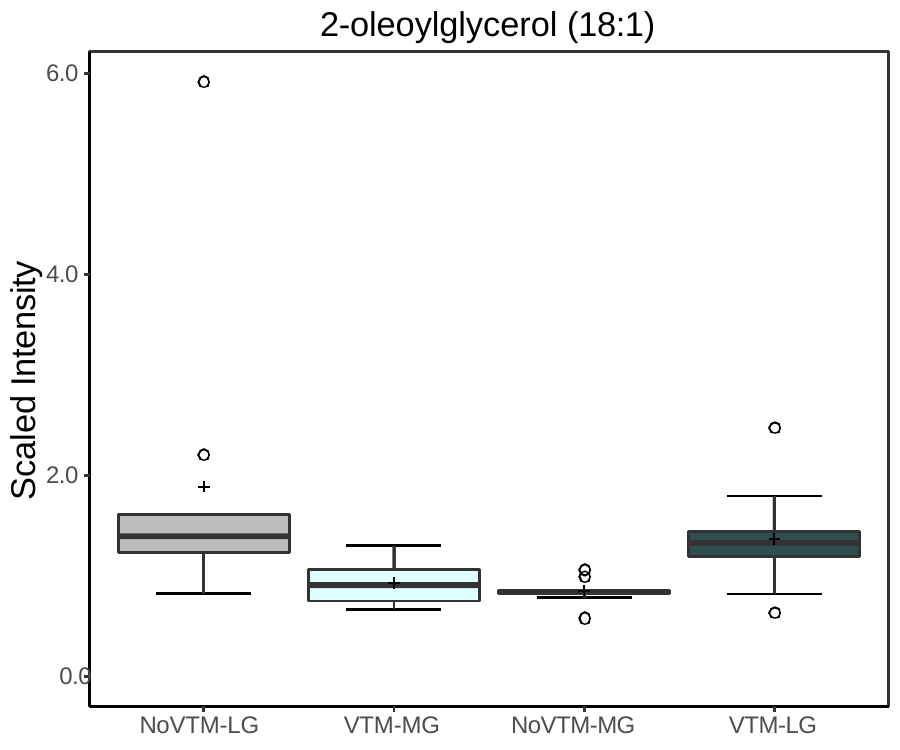

2-oleoylglycerol (18:1)
6.0
Scaled Intensity
4.0
2.0
0.0
NoVTM-LG
VTM-MG
NoVTM-MG
VTM-LG

## Slide 282
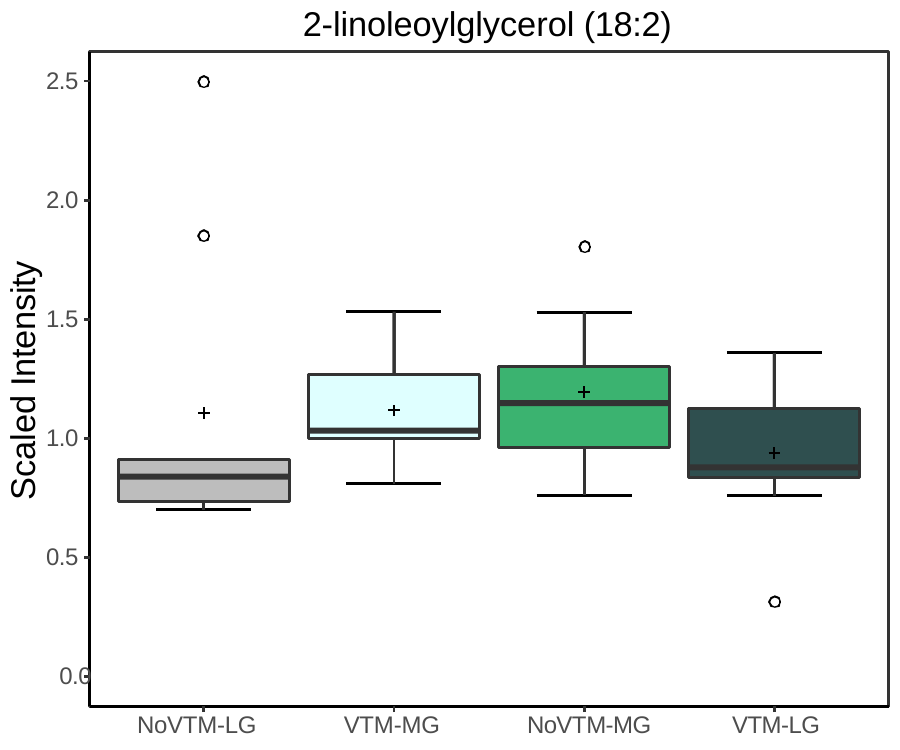

2-linoleoylglycerol (18:2)
2.5
2.0
Scaled Intensity
1.5
1.0
0.5
0.0
NoVTM-LG
VTM-MG
NoVTM-MG
VTM-LG

## Slide 283
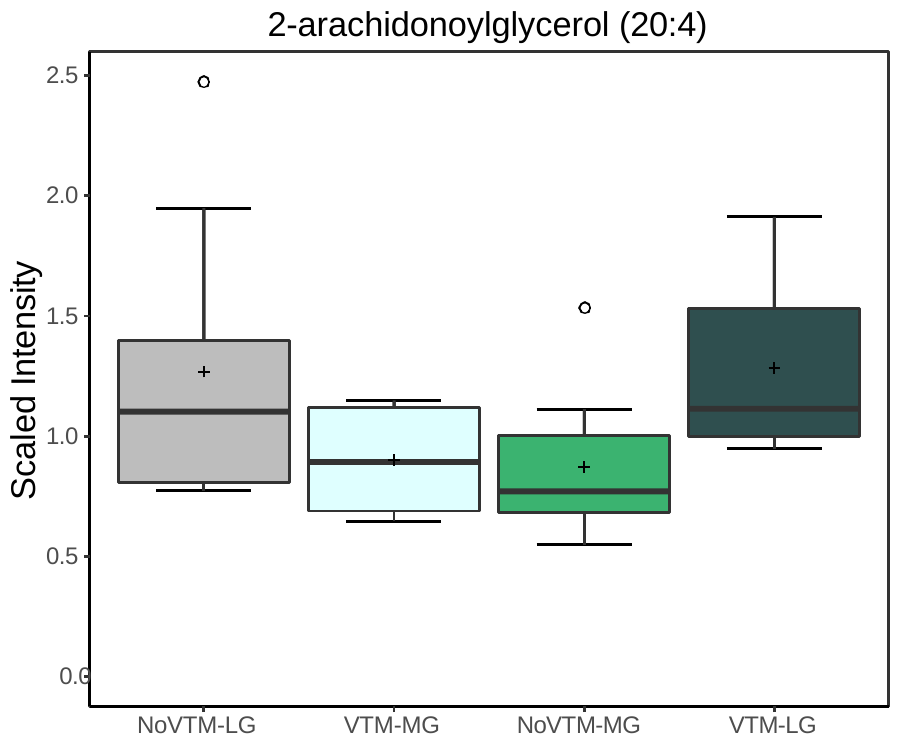

2-arachidonoylglycerol (20:4)
2.5
2.0
Scaled Intensity
1.5
1.0
0.5
0.0
NoVTM-LG
VTM-MG
NoVTM-MG
VTM-LG

## Slide 284
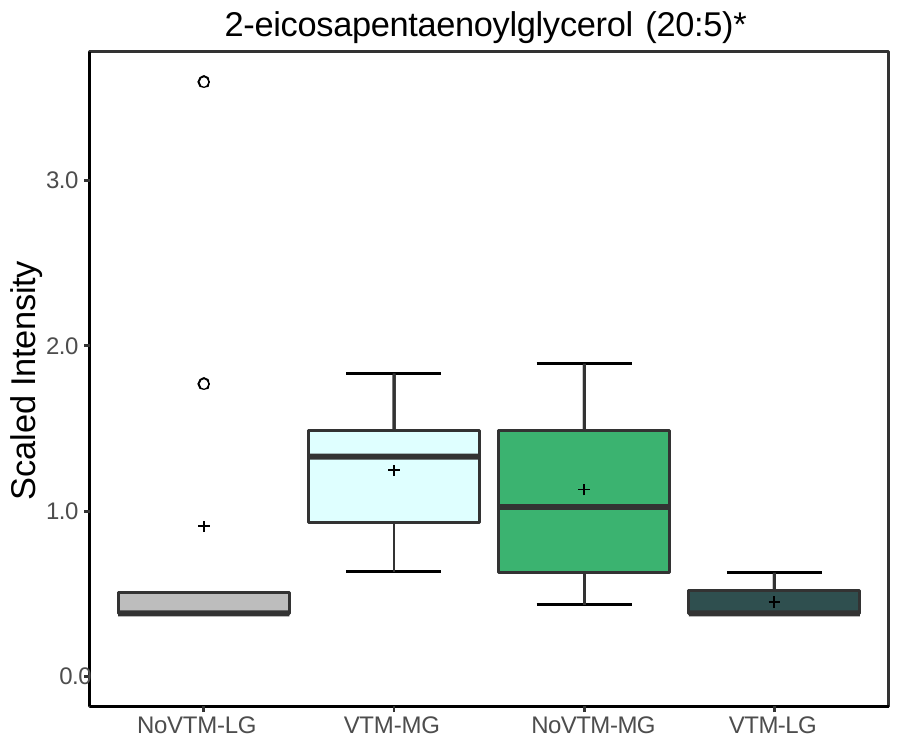

2-eicosapentaenoylglycerol (20:5)*
3.0
Scaled Intensity
2.0
1.0
0.0
NoVTM-LG
VTM-MG
NoVTM-MG
VTM-LG

## Slide 285
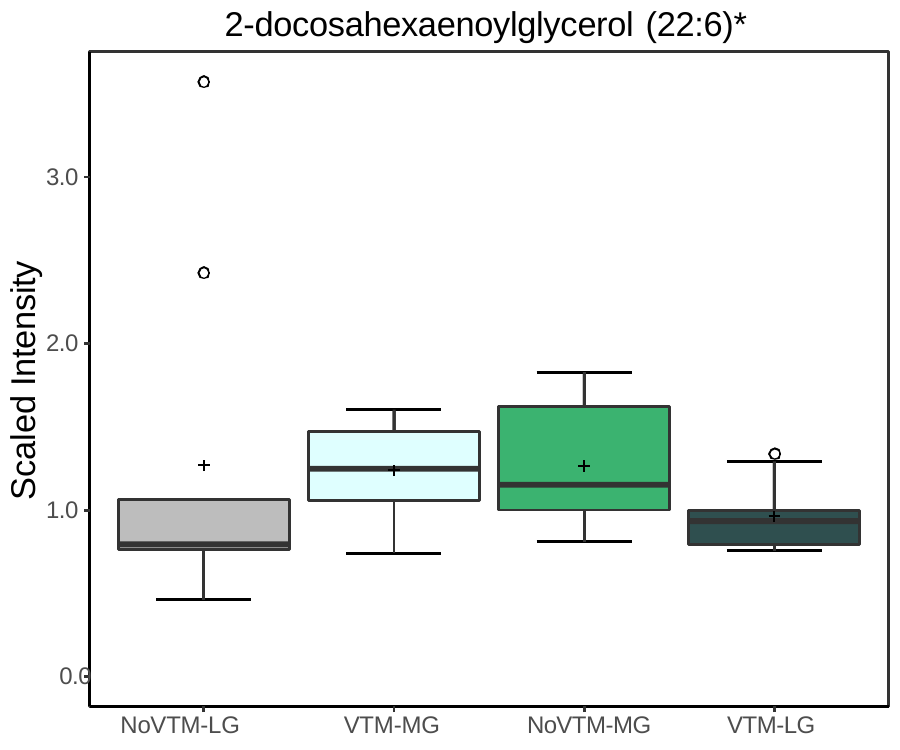

2-docosahexaenoylglycerol (22:6)*
3.0
Scaled Intensity
2.0
1.0
0.0
NoVTM-LG
VTM-MG
NoVTM-MG
VTM-LG

## Slide 286
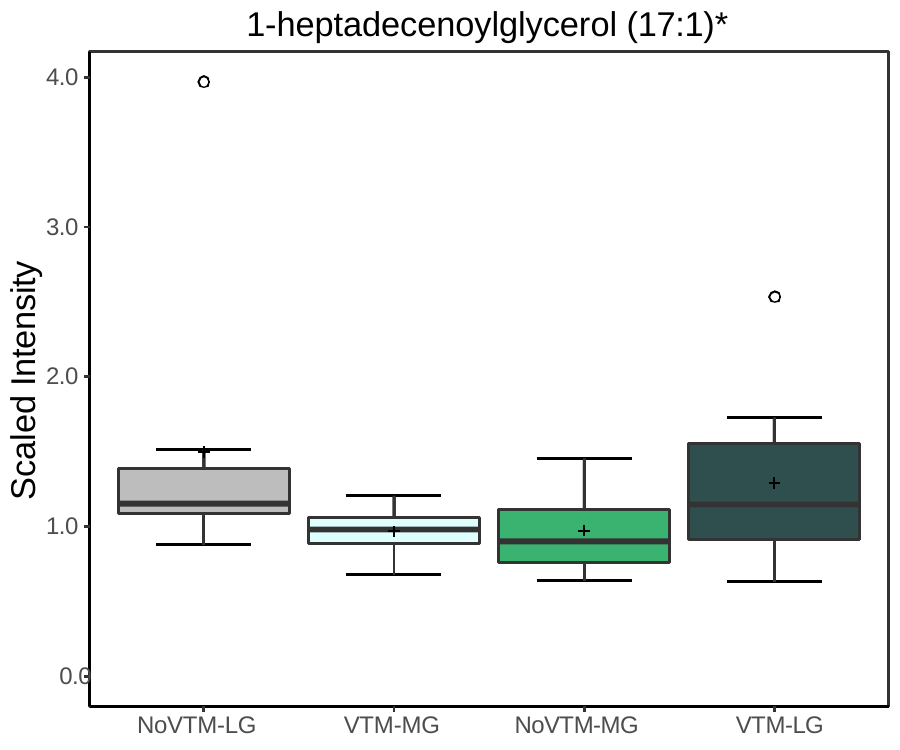

1-heptadecenoylglycerol (17:1)*
4.0
3.0
Scaled Intensity
2.0
1.0
0.0
NoVTM-LG
VTM-MG
NoVTM-MG
VTM-LG

## Slide 287
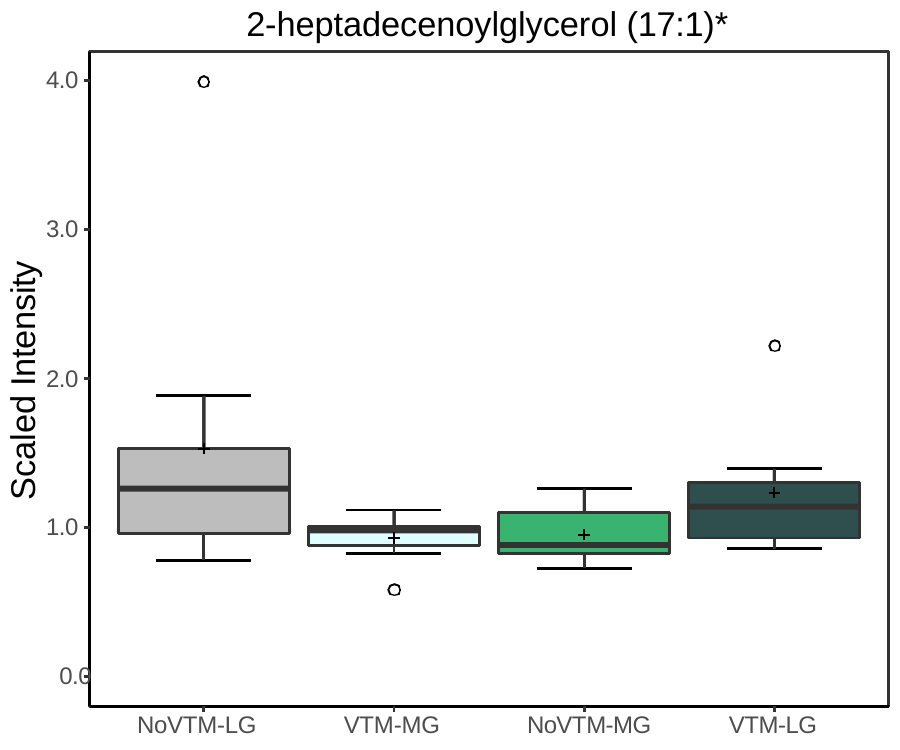

2-heptadecenoylglycerol (17:1)*
4.0
3.0
Scaled Intensity
2.0
1.0
0.0
NoVTM-LG
VTM-MG
NoVTM-MG
VTM-LG

## Slide 288
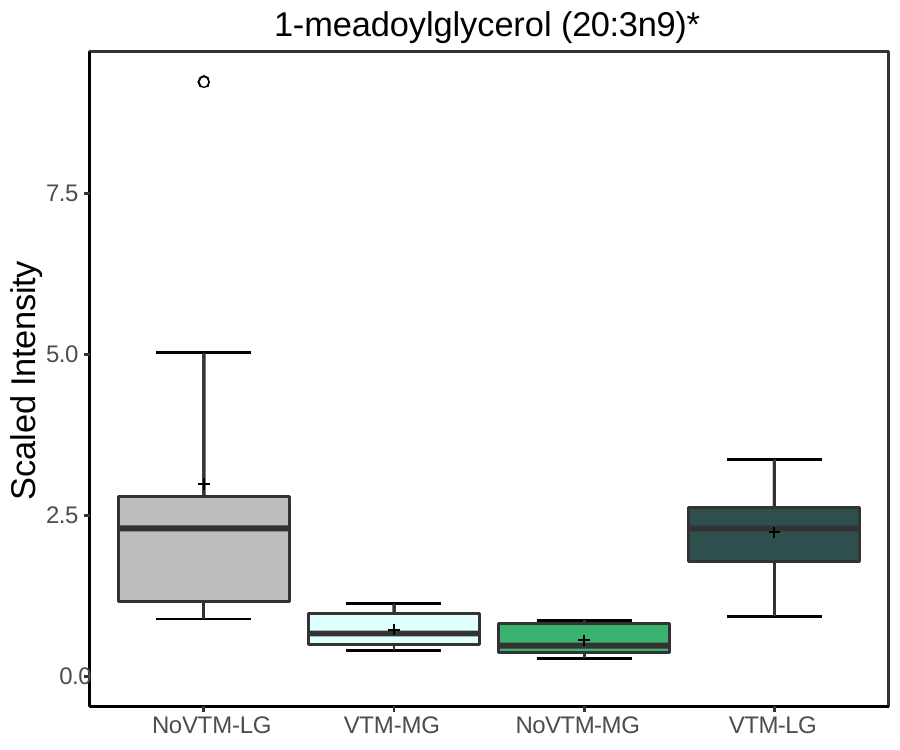

1-meadoylglycerol (20:3n9)*
7.5
Scaled Intensity
5.0
2.5
0.0
NoVTM-LG
VTM-MG
NoVTM-MG
VTM-LG

## Slide 289
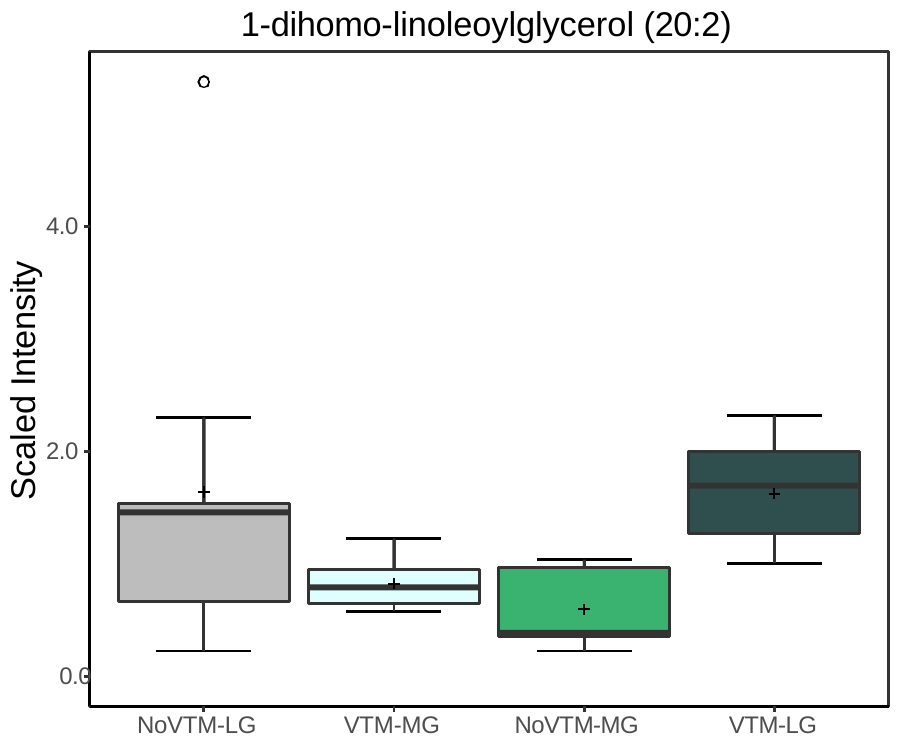

1-dihomo-linoleoylglycerol (20:2)
4.0
Scaled Intensity
2.0
0.0
NoVTM-LG
VTM-MG
NoVTM-MG
VTM-LG

## Slide 290
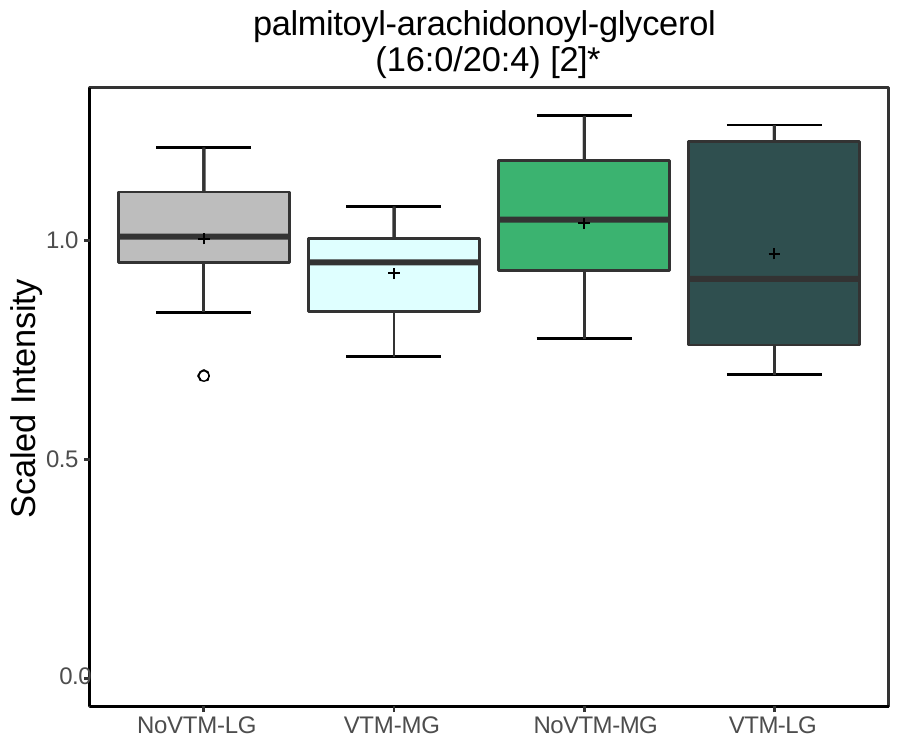

# palmitoyl-arachidonoyl-glycerol (16:0/20:4) [2]*
1.0
Scaled Intensity
0.5
0.0
NoVTM-LG
VTM-MG
NoVTM-MG
VTM-LG

## Slide 291
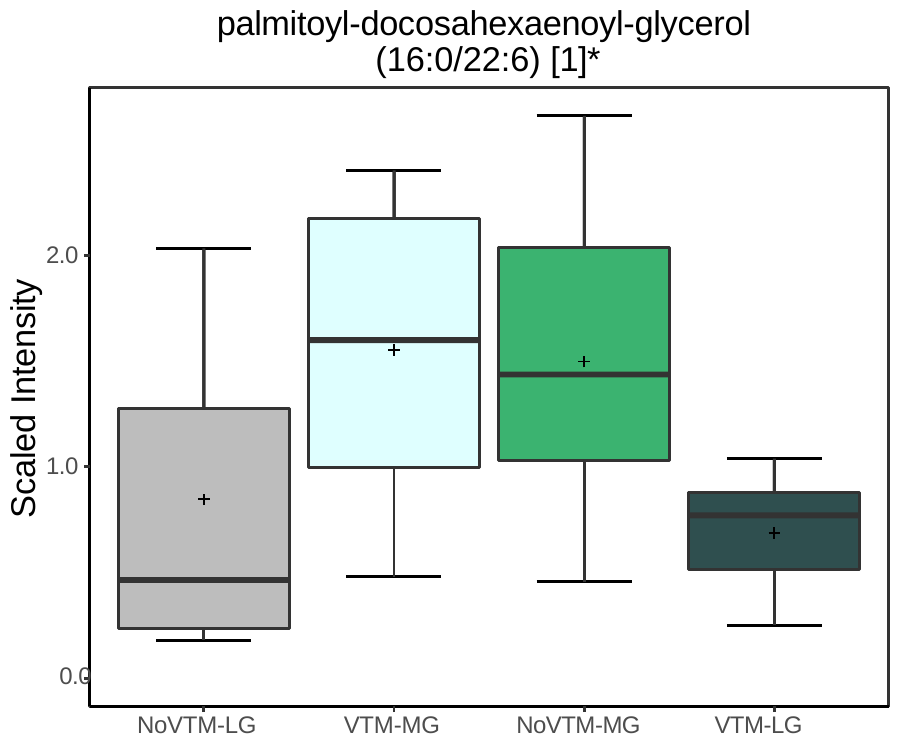

# palmitoyl-docosahexaenoyl-glycerol (16:0/22:6) [1]*
2.0
Scaled Intensity
1.0
0.0
NoVTM-LG
VTM-MG
NoVTM-MG
VTM-LG

## Slide 292
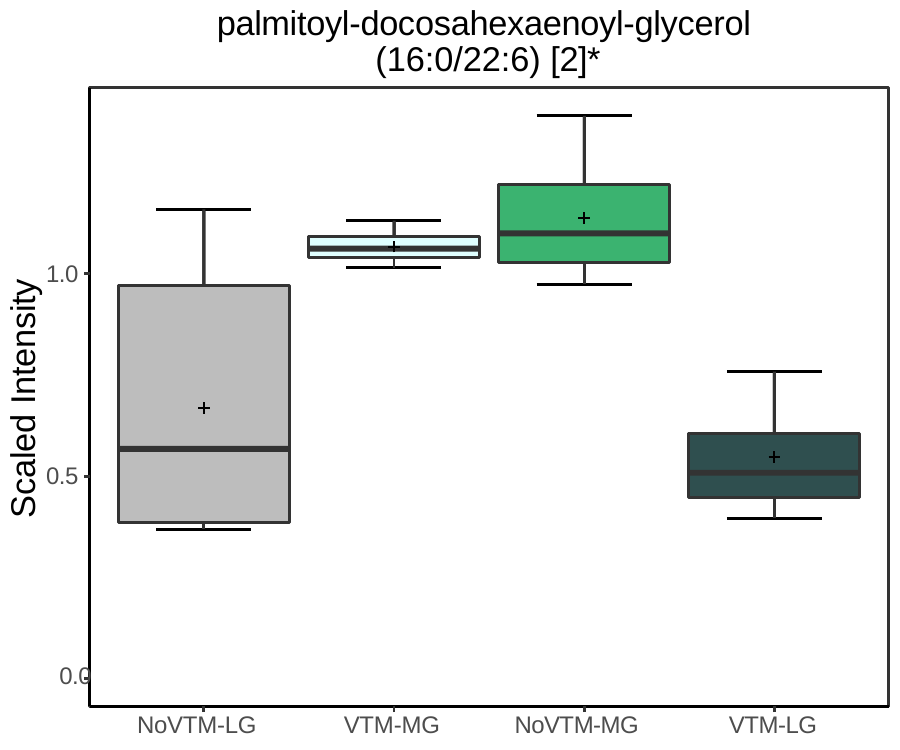

# palmitoyl-docosahexaenoyl-glycerol (16:0/22:6) [2]*
1.0
Scaled Intensity
0.5
0.0
NoVTM-LG
VTM-MG
NoVTM-MG
VTM-LG

## Slide 293
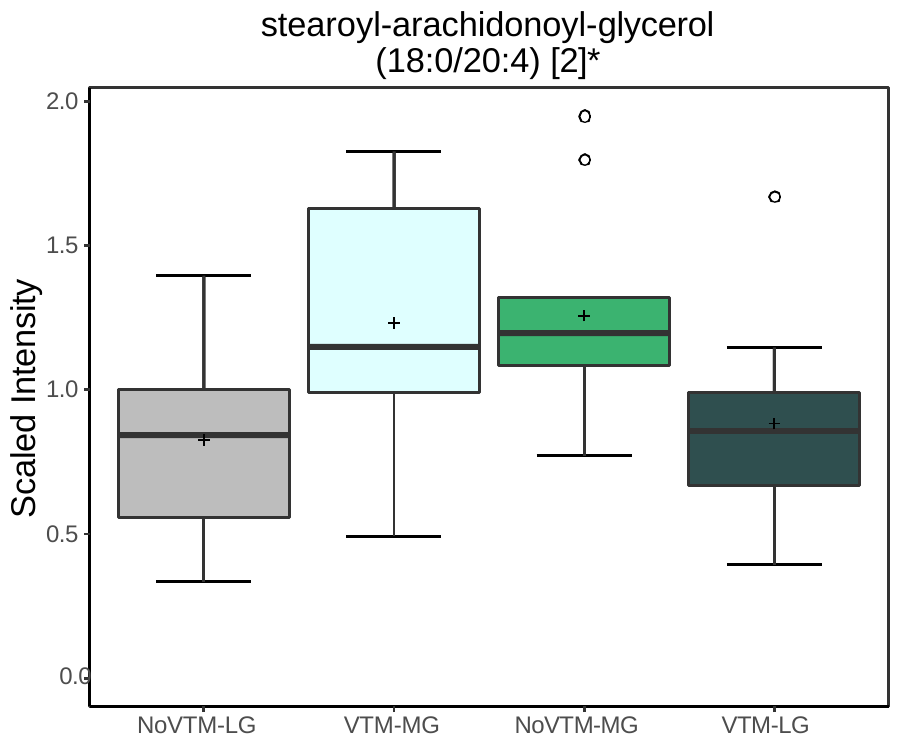

# stearoyl-arachidonoyl-glycerol (18:0/20:4) [2]*
2.0
1.5
Scaled Intensity
1.0
0.5
0.0
NoVTM-LG
VTM-MG
NoVTM-MG
VTM-LG

## Slide 294
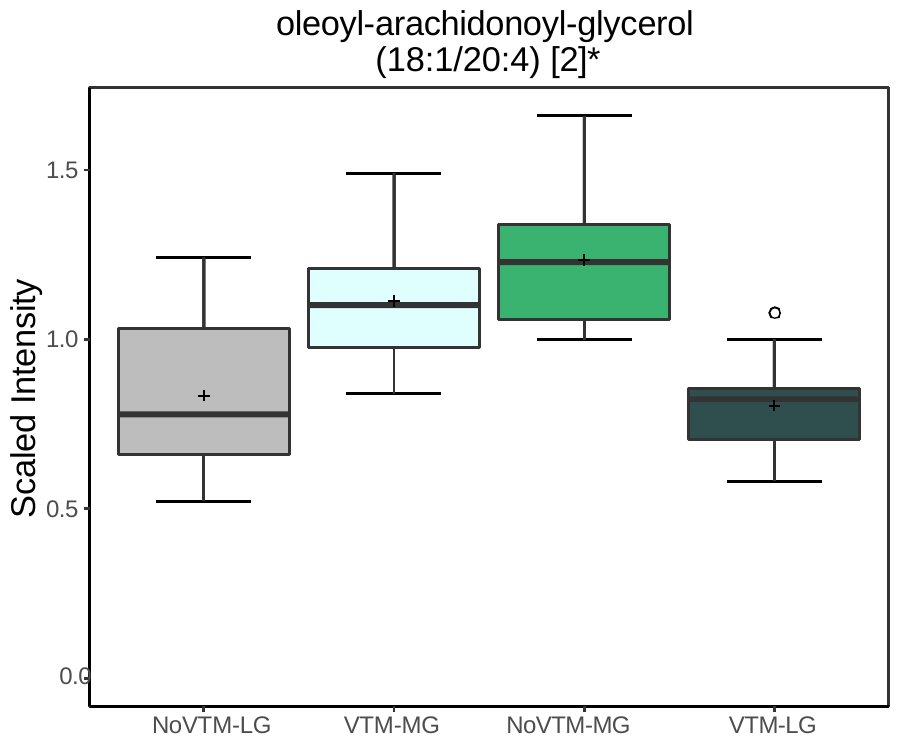

# oleoyl-arachidonoyl-glycerol (18:1/20:4) [2]*
1.5
Scaled Intensity
1.0
0.5
0.0
NoVTM-LG
VTM-MG
NoVTM-MG
VTM-LG

## Slide 295
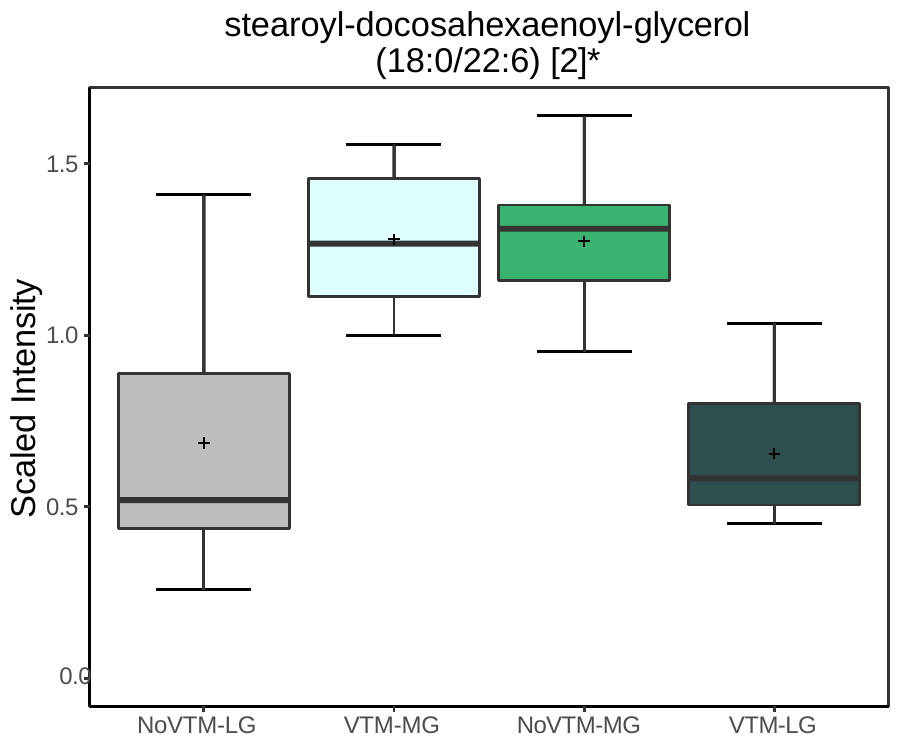

# stearoyl-docosahexaenoyl-glycerol (18:0/22:6) [2]*
1.5
Scaled Intensity
1.0
0.5
0.0
NoVTM-LG
VTM-MG
NoVTM-MG
VTM-LG

## Slide 296
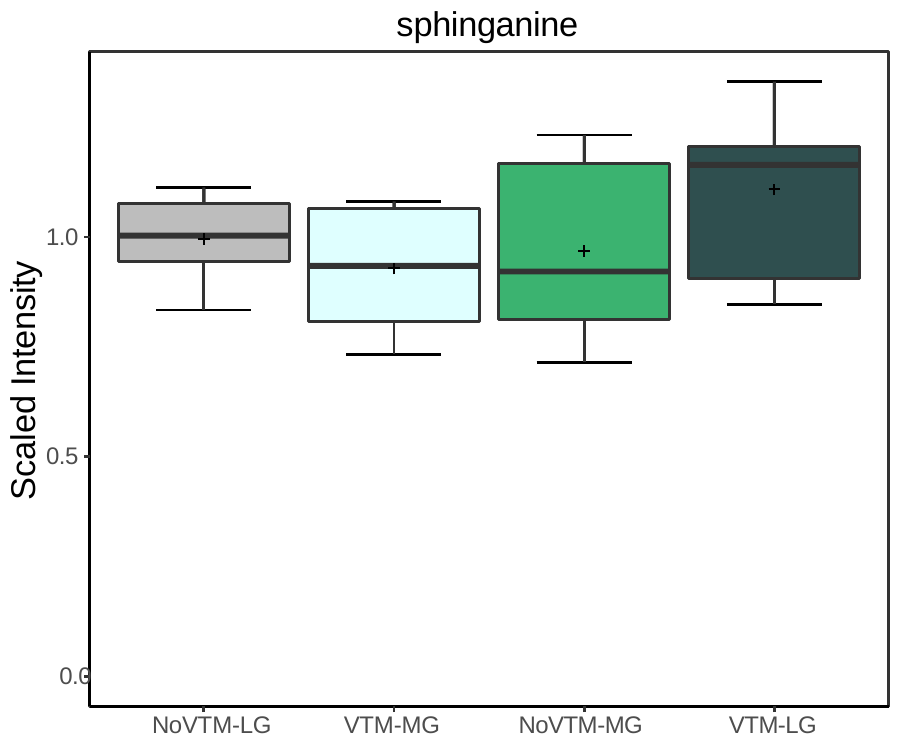

sphinganine
1.0
Scaled Intensity
0.5
0.0
NoVTM-LG
VTM-MG
NoVTM-MG
VTM-LG

## Slide 297
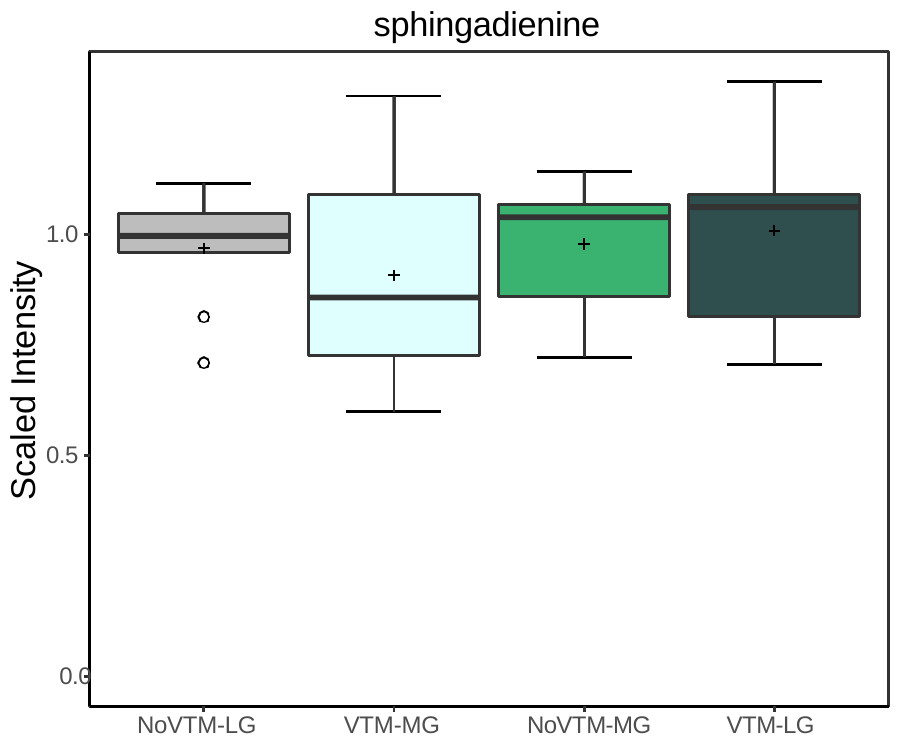

sphingadienine
1.0
Scaled Intensity
0.5
0.0
NoVTM-LG
VTM-MG
NoVTM-MG
VTM-LG

## Slide 298
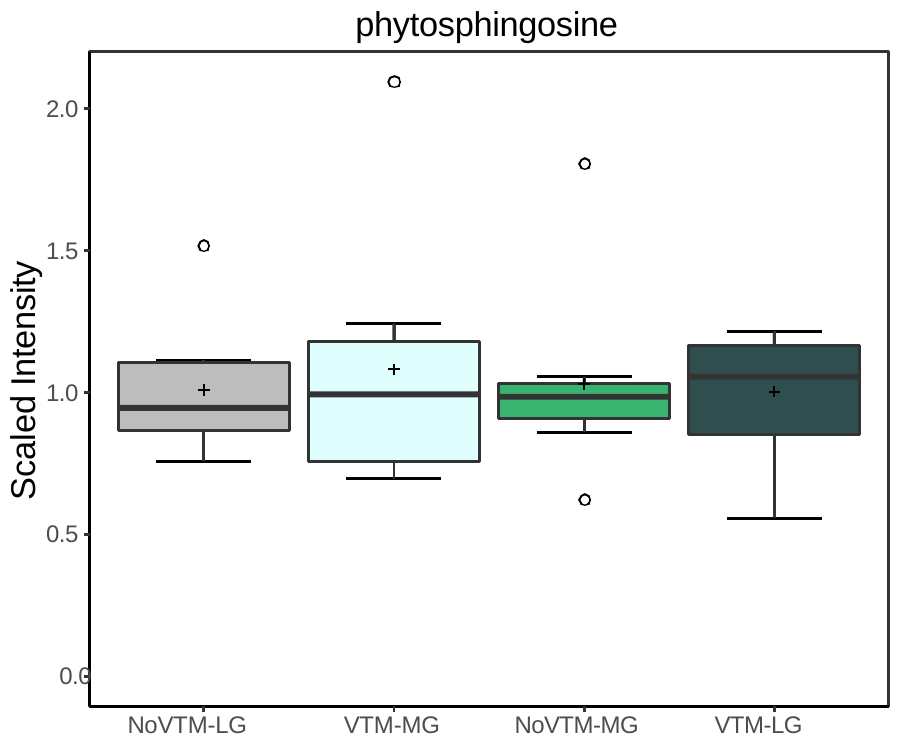

phytosphingosine
2.0
1.5
Scaled Intensity
1.0
0.5
0.0
NoVTM-LG
VTM-MG
NoVTM-MG
VTM-LG

## Slide 299
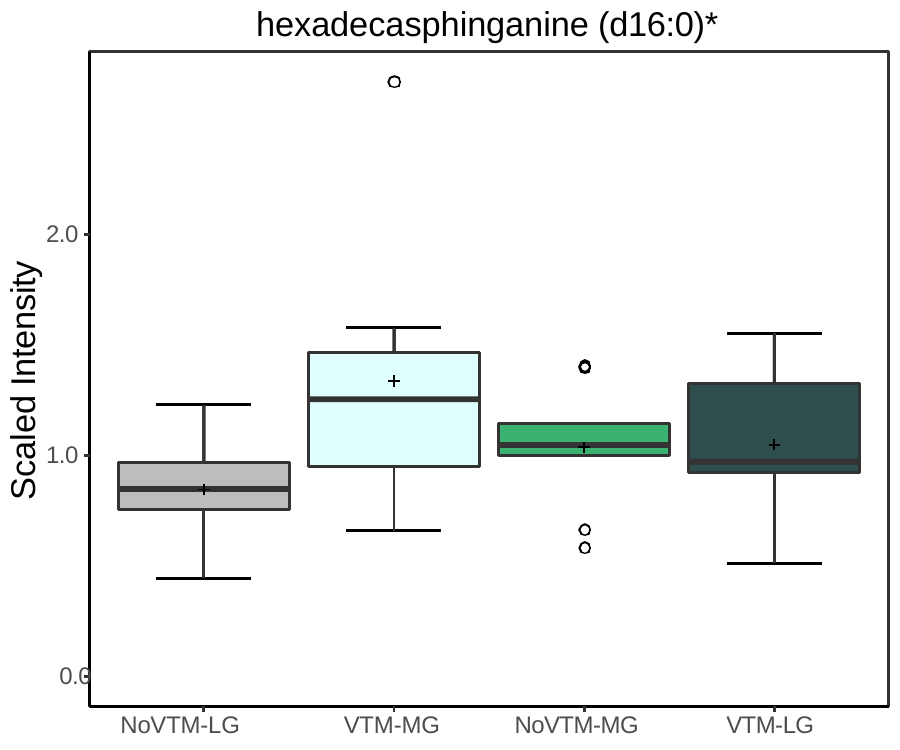

hexadecasphinganine (d16:0)*
2.0
Scaled Intensity
1.0
0.0
NoVTM-LG
VTM-MG
NoVTM-MG
VTM-LG

## Slide 300
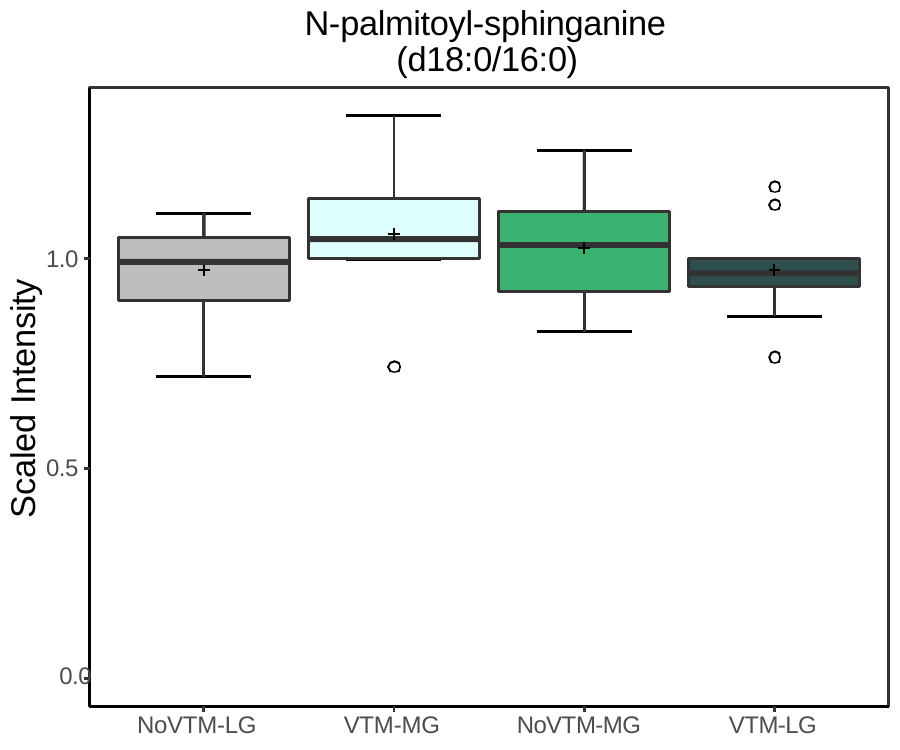

# N-palmitoyl-sphinganine (d18:0/16:0)
1.0
Scaled Intensity
0.5
0.0
NoVTM-LG
VTM-MG
NoVTM-MG
VTM-LG

## Slide 301
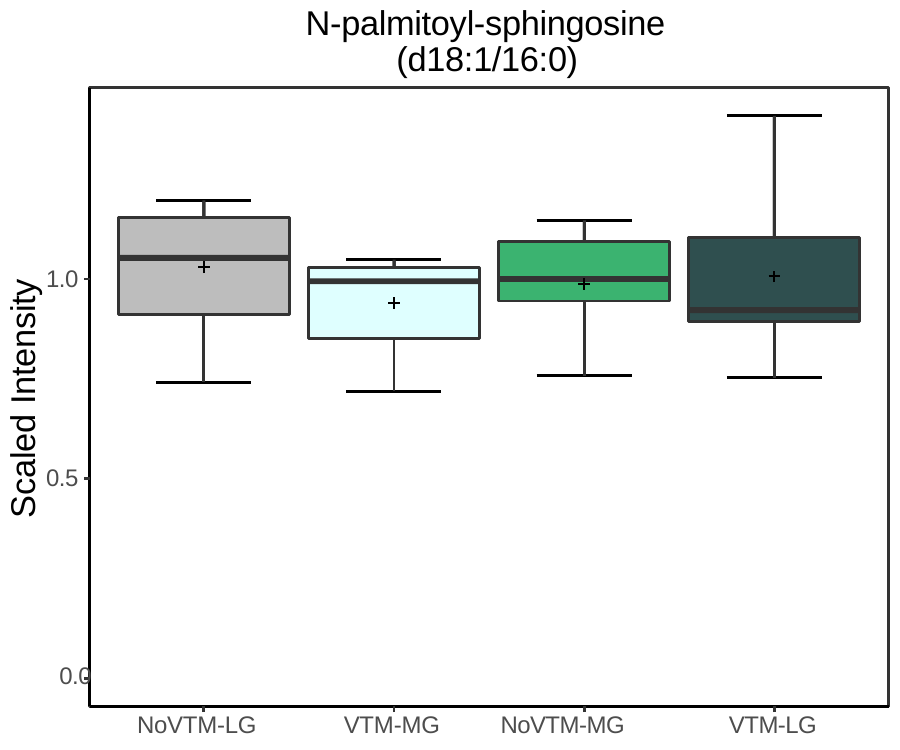

# N-palmitoyl-sphingosine (d18:1/16:0)
1.0
Scaled Intensity
0.5
0.0
NoVTM-LG
VTM-MG
NoVTM-MG
VTM-LG

## Slide 302
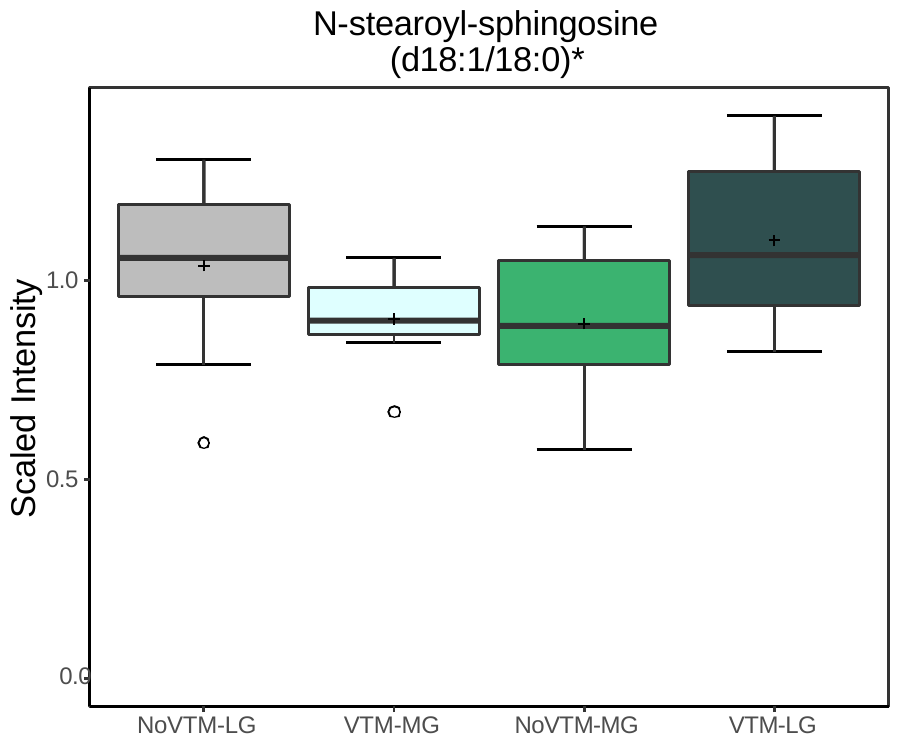

# N-stearoyl-sphingosine (d18:1/18:0)*
1.0
Scaled Intensity
0.5
0.0
NoVTM-LG
VTM-MG
NoVTM-MG
VTM-LG

## Slide 303
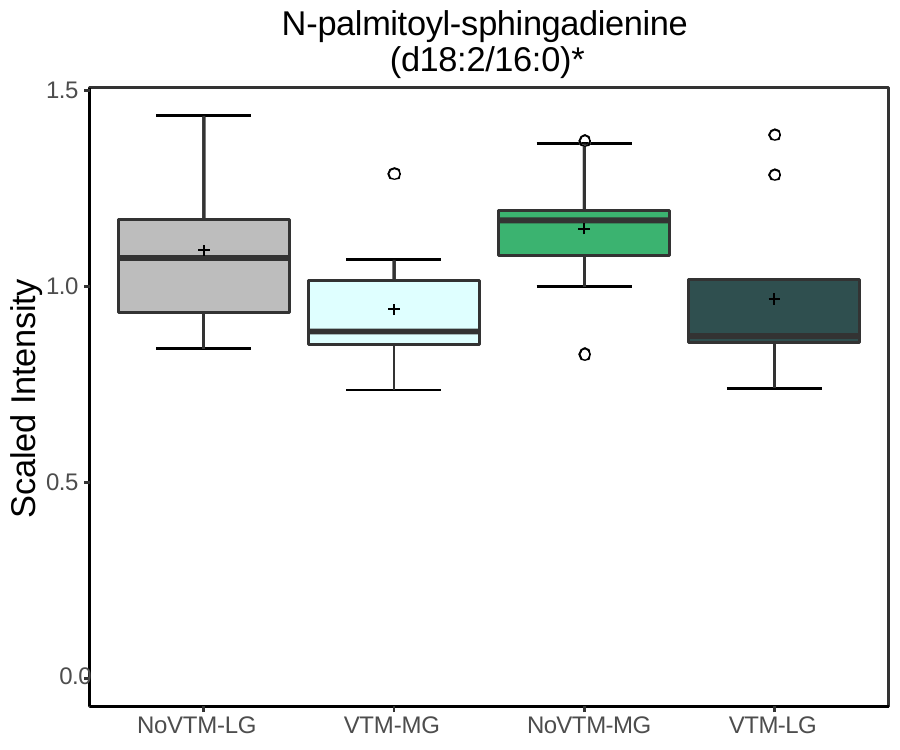

# N-palmitoyl-sphingadienine (d18:2/16:0)*
1.5
1.0
Scaled Intensity
0.5
0.0
NoVTM-LG
VTM-MG
NoVTM-MG
VTM-LG

## Slide 304
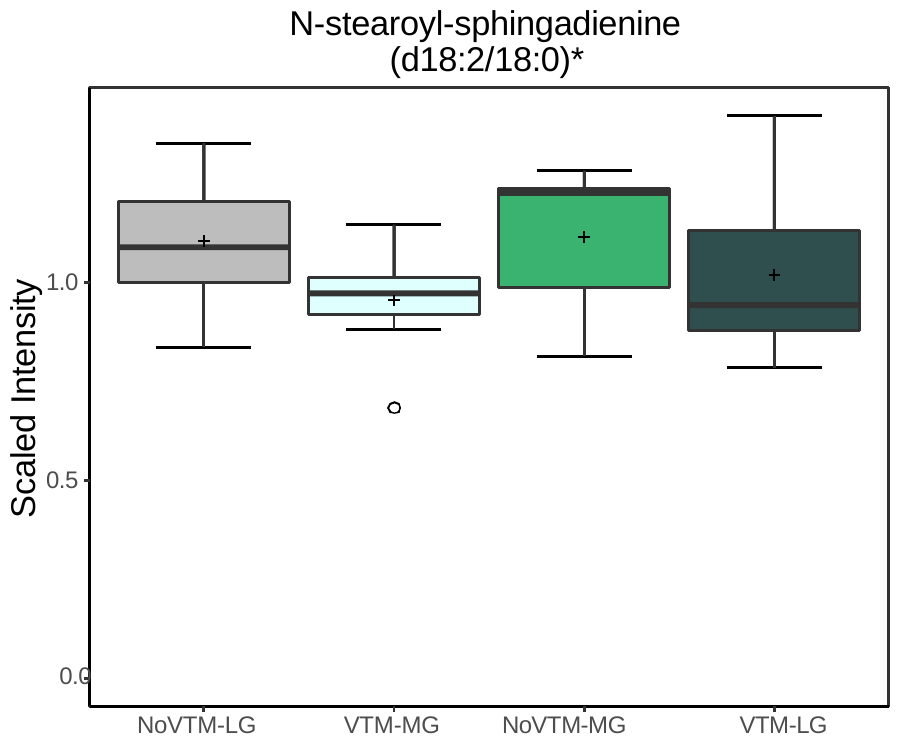

# N-stearoyl-sphingadienine (d18:2/18:0)*
1.0
Scaled Intensity
0.5
0.0
NoVTM-LG
VTM-MG
NoVTM-MG
VTM-LG

## Slide 305
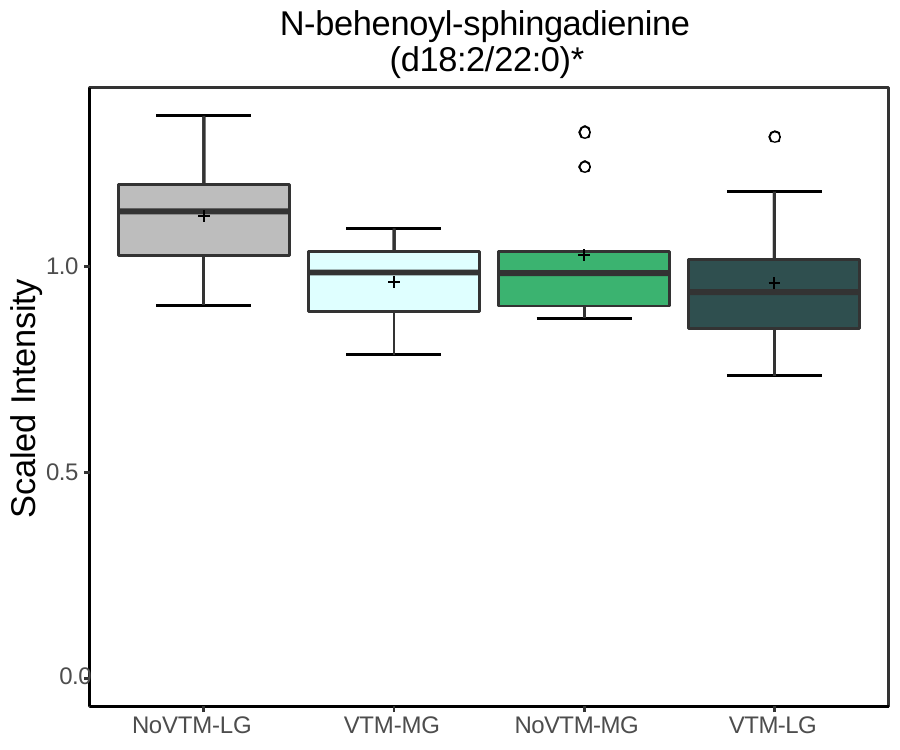

# N-behenoyl-sphingadienine (d18:2/22:0)*
1.0
Scaled Intensity
0.5
0.0
NoVTM-LG
VTM-MG
NoVTM-MG
VTM-LG

## Slide 306
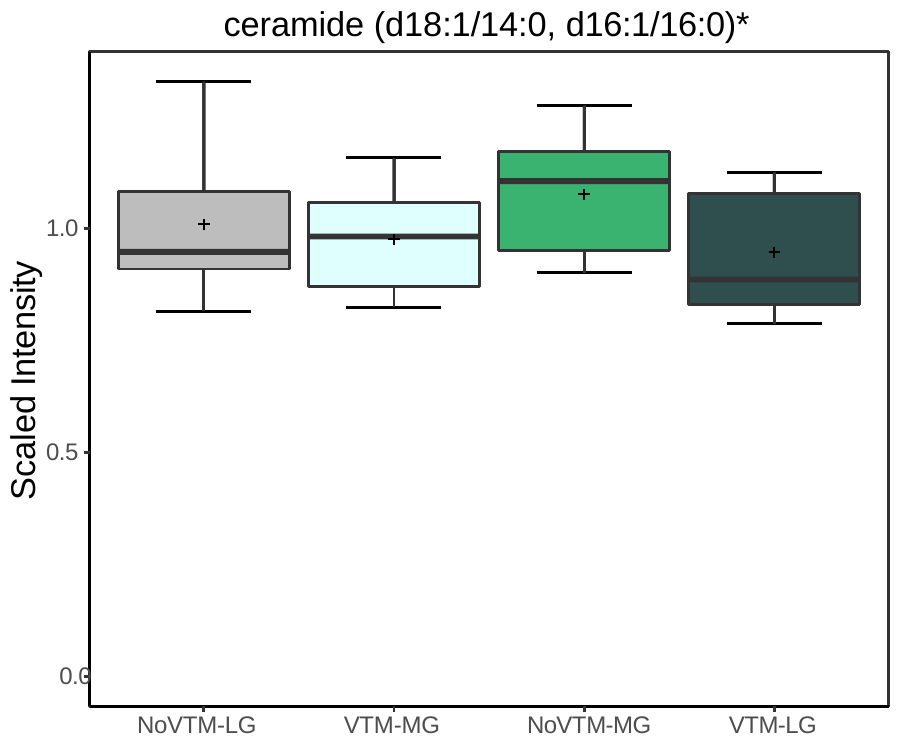

ceramide (d18:1/14:0, d16:1/16:0)*
1.0
Scaled Intensity
0.5
0.0
NoVTM-LG
VTM-MG
NoVTM-MG
VTM-LG

## Slide 307
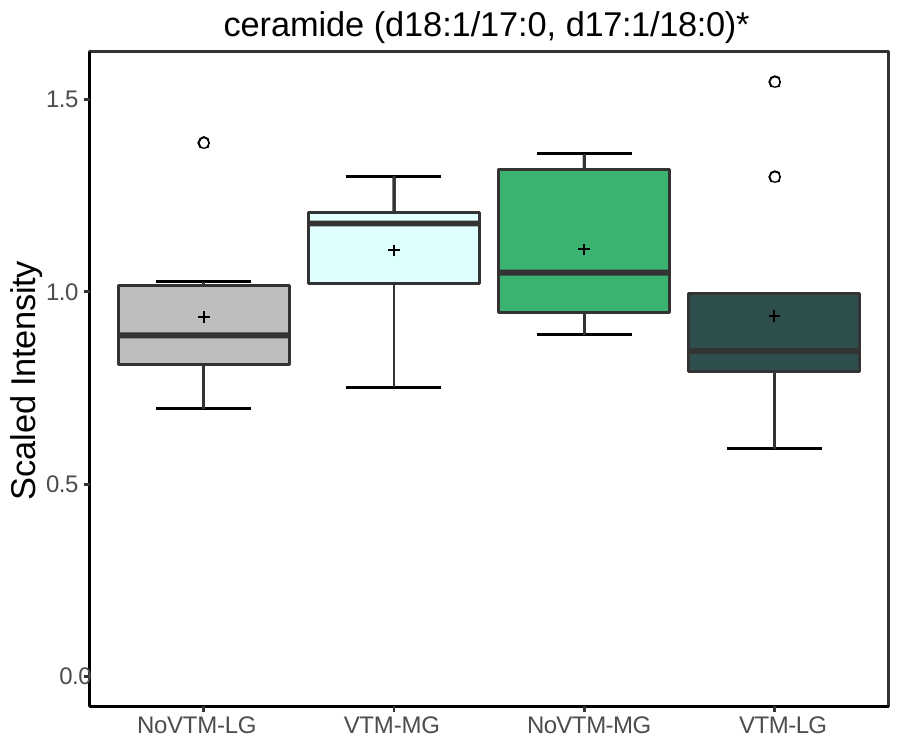

ceramide (d18:1/17:0, d17:1/18:0)*
1.5
Scaled Intensity
1.0
0.5
0.0
NoVTM-LG
VTM-MG
NoVTM-MG
VTM-LG

## Slide 308
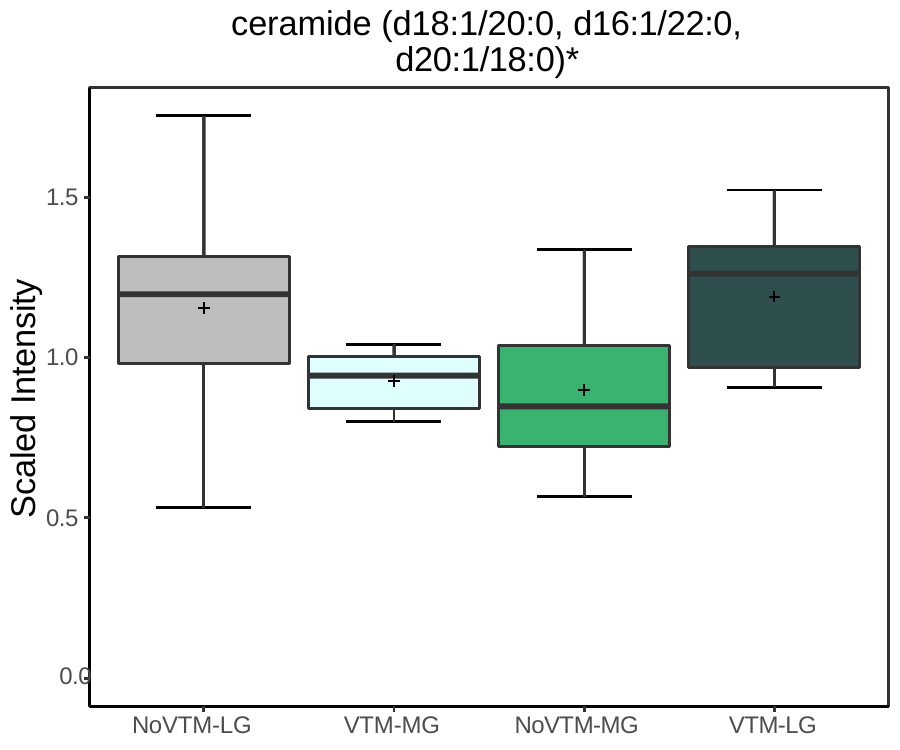

# ceramide (d18:1/20:0, d16:1/22:0, d20:1/18:0)*
1.5
Scaled Intensity
1.0
0.5
0.0
NoVTM-LG
VTM-MG
NoVTM-MG
VTM-LG

## Slide 309
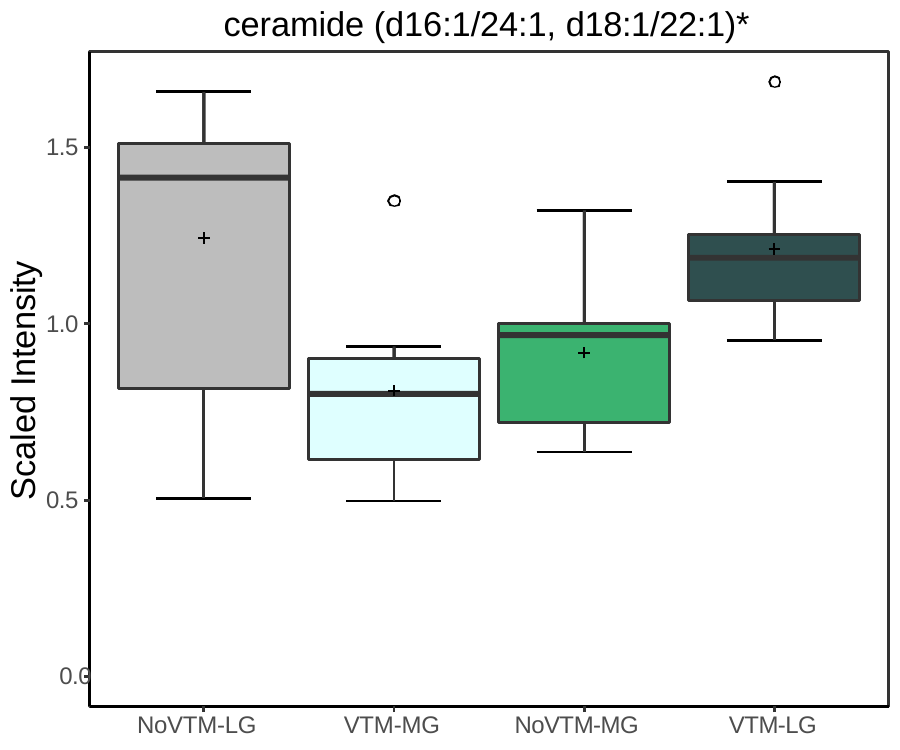

ceramide (d16:1/24:1, d18:1/22:1)*
1.5
Scaled Intensity
1.0
0.5
0.0
NoVTM-LG
VTM-MG
NoVTM-MG
VTM-LG

## Slide 310
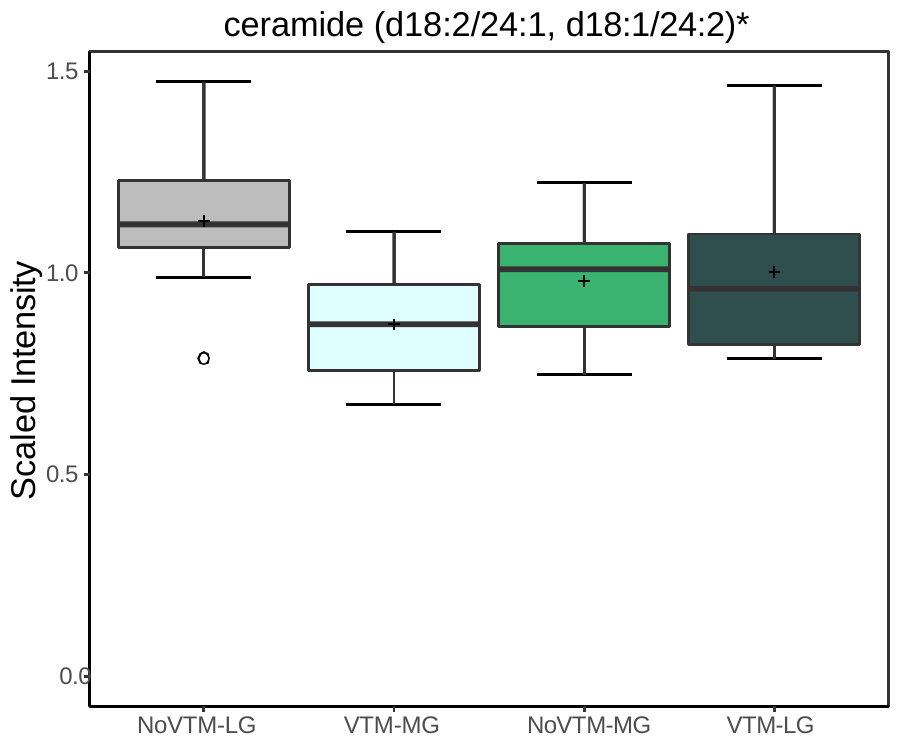

ceramide (d18:2/24:1, d18:1/24:2)*
1.5
1.0
Scaled Intensity
0.5
0.0
NoVTM-LG
VTM-MG
NoVTM-MG
VTM-LG

## Slide 311
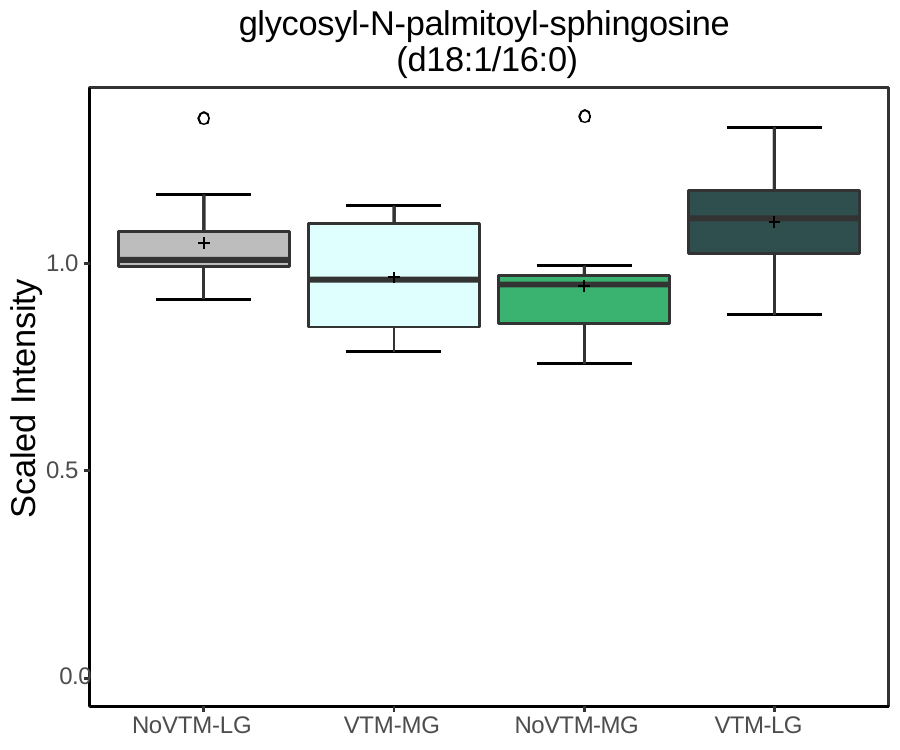

# glycosyl-N-palmitoyl-sphingosine (d18:1/16:0)
1.0
Scaled Intensity
0.5
0.0
NoVTM-LG
VTM-MG
NoVTM-MG
VTM-LG

## Slide 312
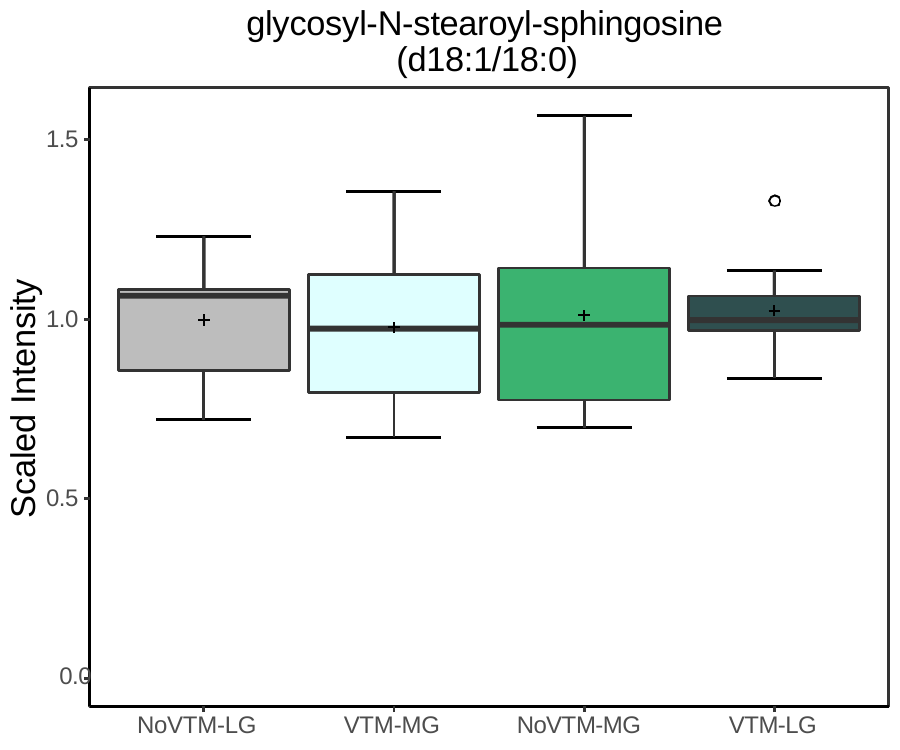

# glycosyl-N-stearoyl-sphingosine (d18:1/18:0)
1.5
Scaled Intensity
1.0
0.5
0.0
NoVTM-LG
VTM-MG
NoVTM-MG
VTM-LG

## Slide 313
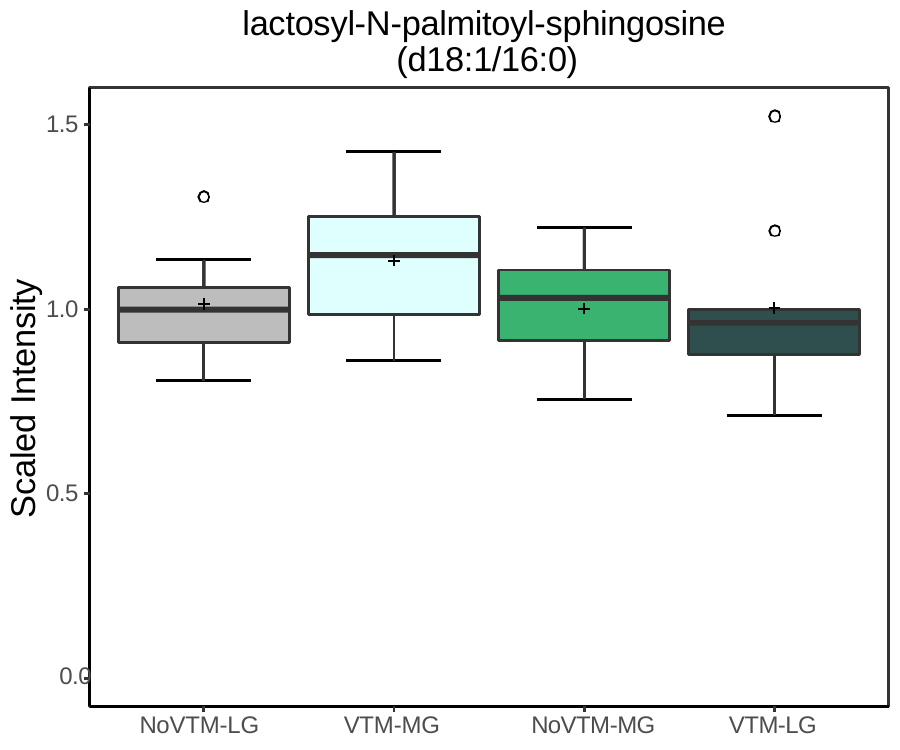

# lactosyl-N-palmitoyl-sphingosine (d18:1/16:0)
1.5
Scaled Intensity
1.0
0.5
0.0
NoVTM-LG
VTM-MG
NoVTM-MG
VTM-LG

## Slide 314
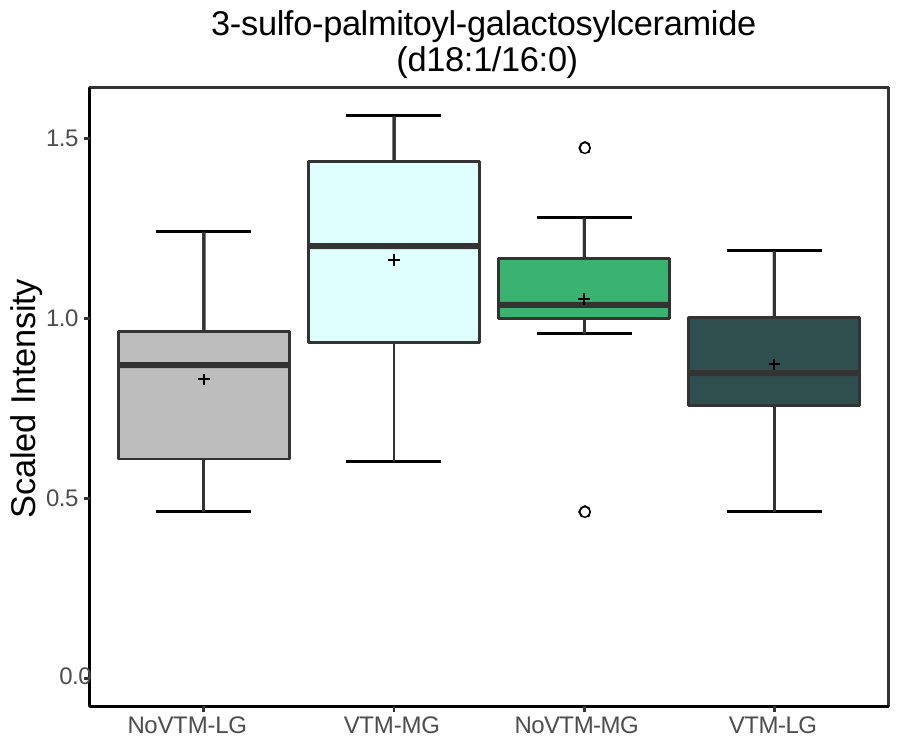

# 3-sulfo-palmitoyl-galactosylceramide (d18:1/16:0)
1.5
Scaled Intensity
1.0
0.5
0.0
NoVTM-LG
VTM-MG
NoVTM-MG
VTM-LG

## Slide 315
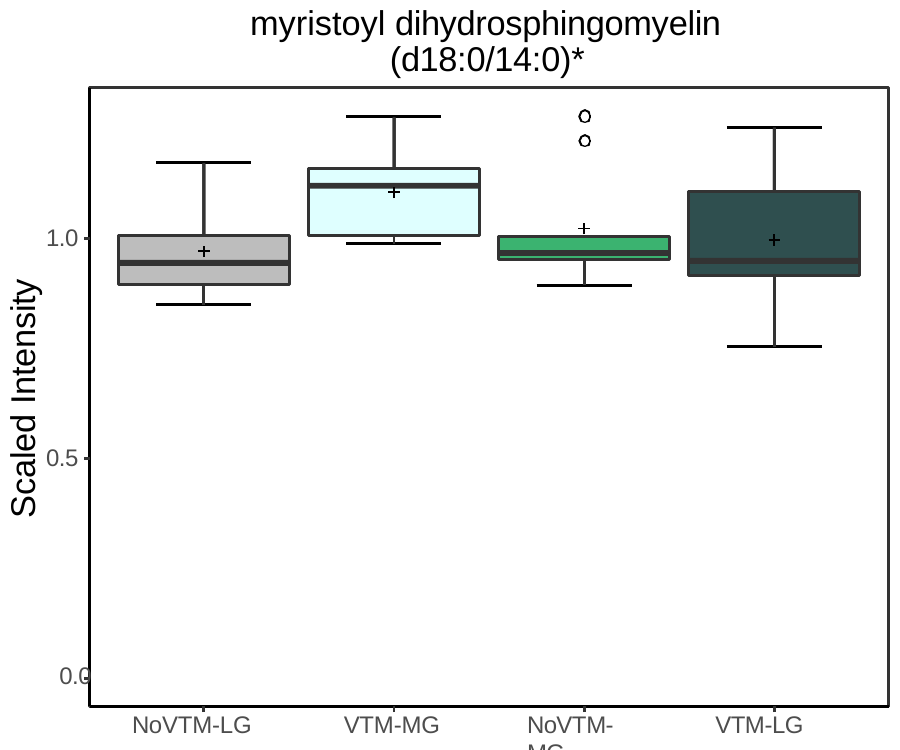

# myristoyl dihydrosphingomyelin (d18:0/14:0)*
1.0
Scaled Intensity
0.5
0.0
NoVTM-LG
VTM-MG
NoVTM-MG
VTM-LG

## Slide 316
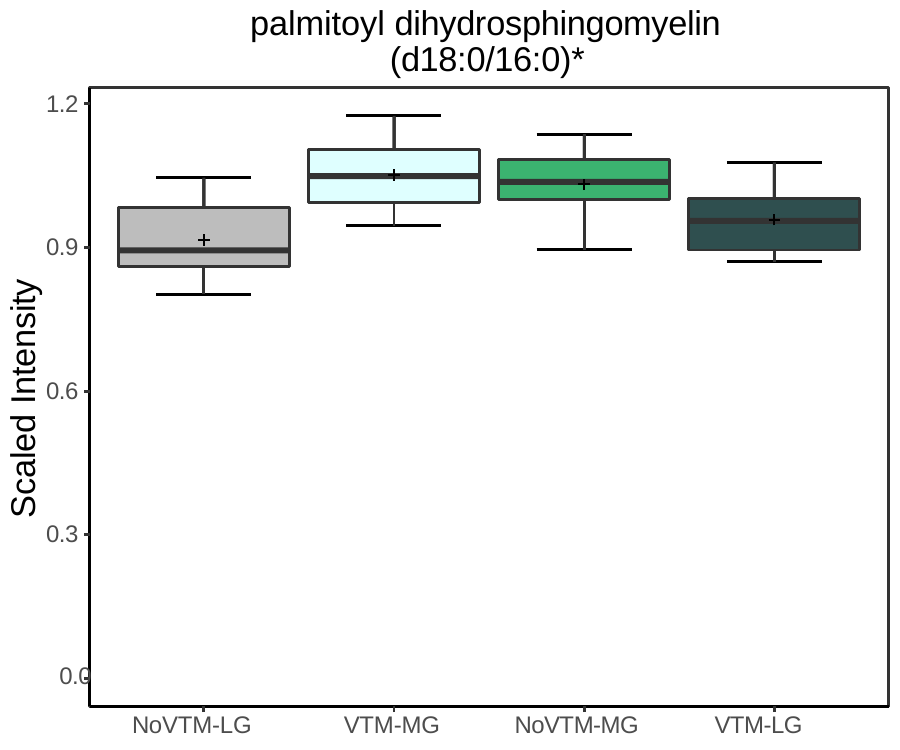

# palmitoyl dihydrosphingomyelin (d18:0/16:0)*
1.2
0.9
Scaled Intensity
0.6
0.3
0.0
NoVTM-LG
VTM-MG
NoVTM-MG
VTM-LG

## Slide 317
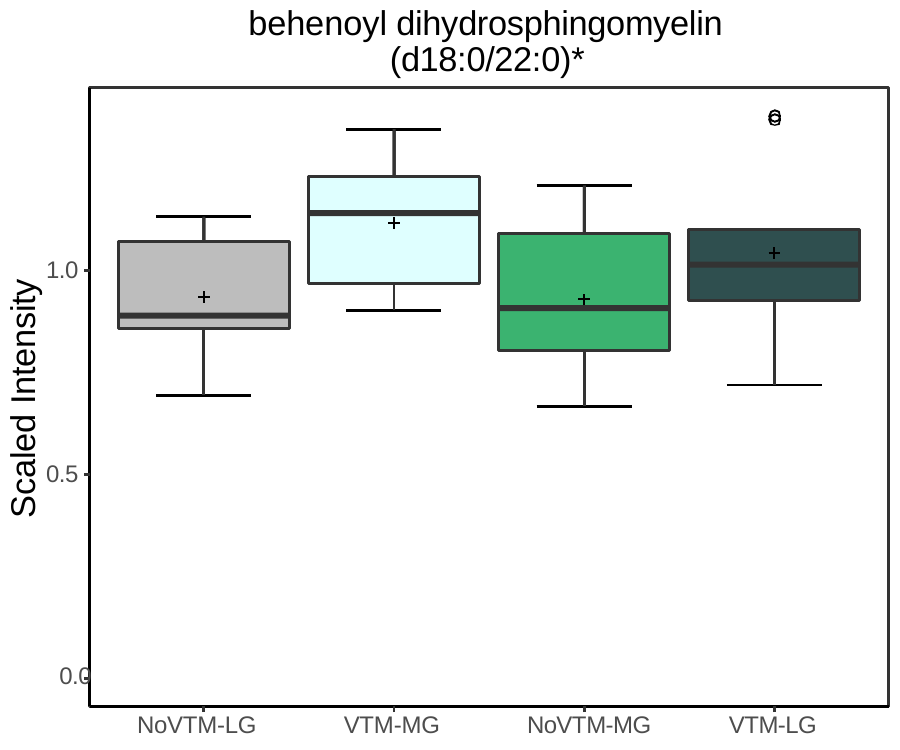

# behenoyl dihydrosphingomyelin (d18:0/22:0)*
1.0
Scaled Intensity
0.5
0.0
NoVTM-LG
VTM-MG
NoVTM-MG
VTM-LG

## Slide 318
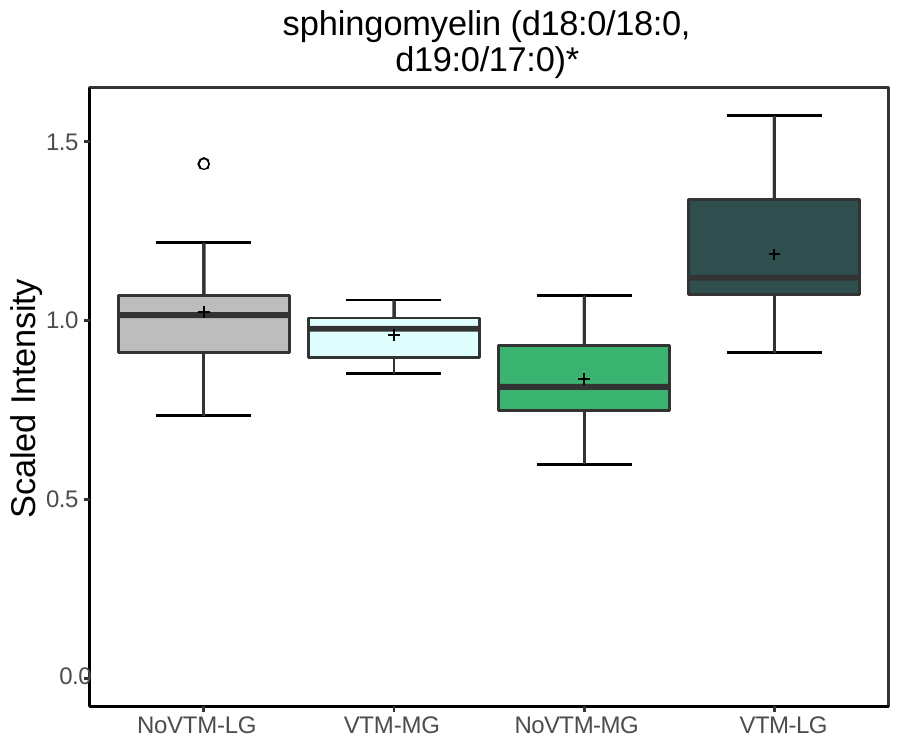

# sphingomyelin (d18:0/18:0, d19:0/17:0)*
1.5
Scaled Intensity
1.0
0.5
0.0
NoVTM-LG
VTM-MG
NoVTM-MG
VTM-LG

## Slide 319
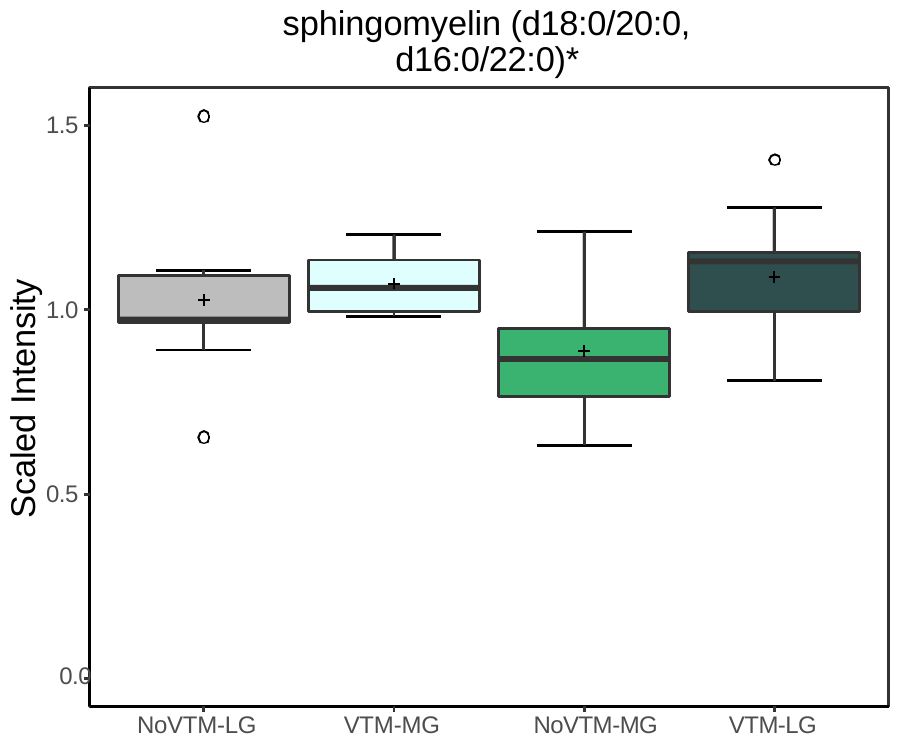

# sphingomyelin (d18:0/20:0, d16:0/22:0)*
1.5
Scaled Intensity
1.0
0.5
0.0
NoVTM-LG
VTM-MG
NoVTM-MG
VTM-LG

## Slide 320
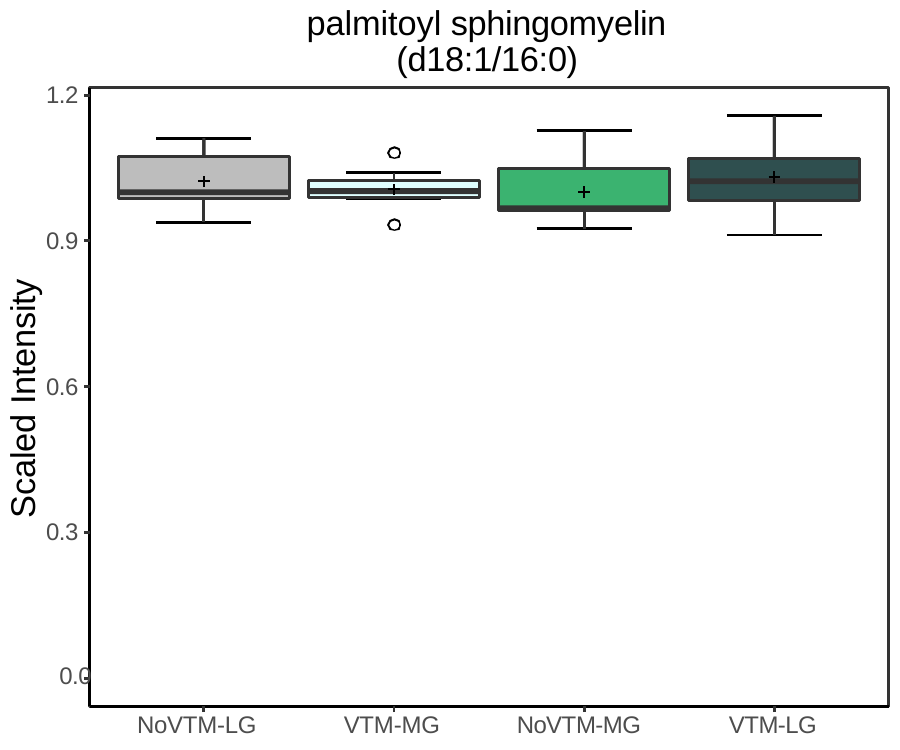

# palmitoyl sphingomyelin (d18:1/16:0)
1.2
0.9
Scaled Intensity
0.6
0.3
0.0
NoVTM-LG
VTM-MG
NoVTM-MG
VTM-LG

## Slide 321
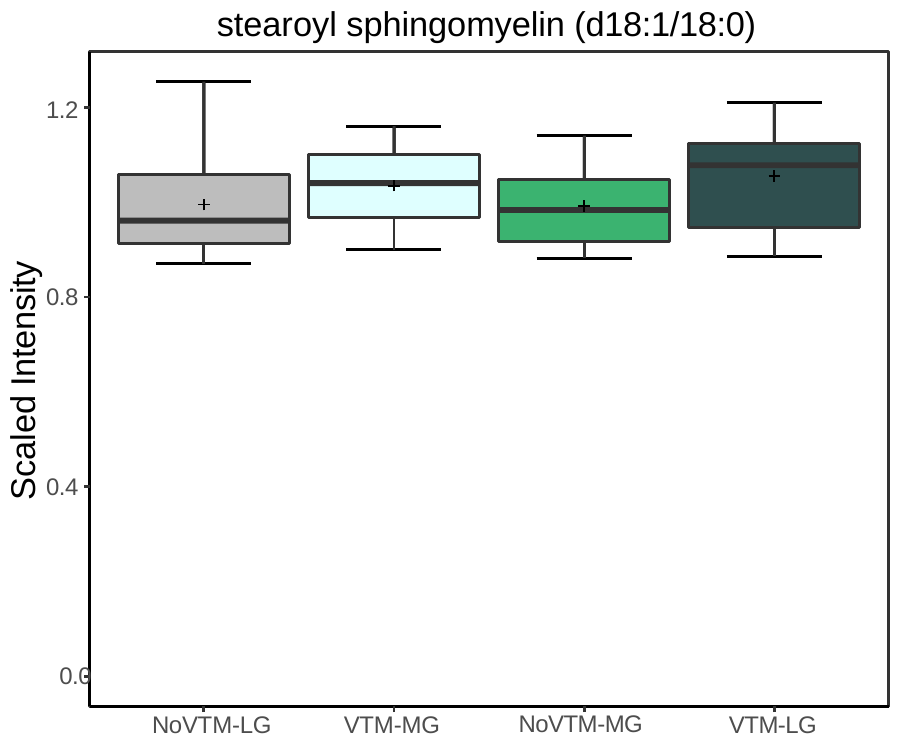

stearoyl sphingomyelin (d18:1/18:0)
1.2
Scaled Intensity
0.8
0.4
0.0
NoVTM-MG
NoVTM-LG
VTM-MG
VTM-LG

## Slide 322
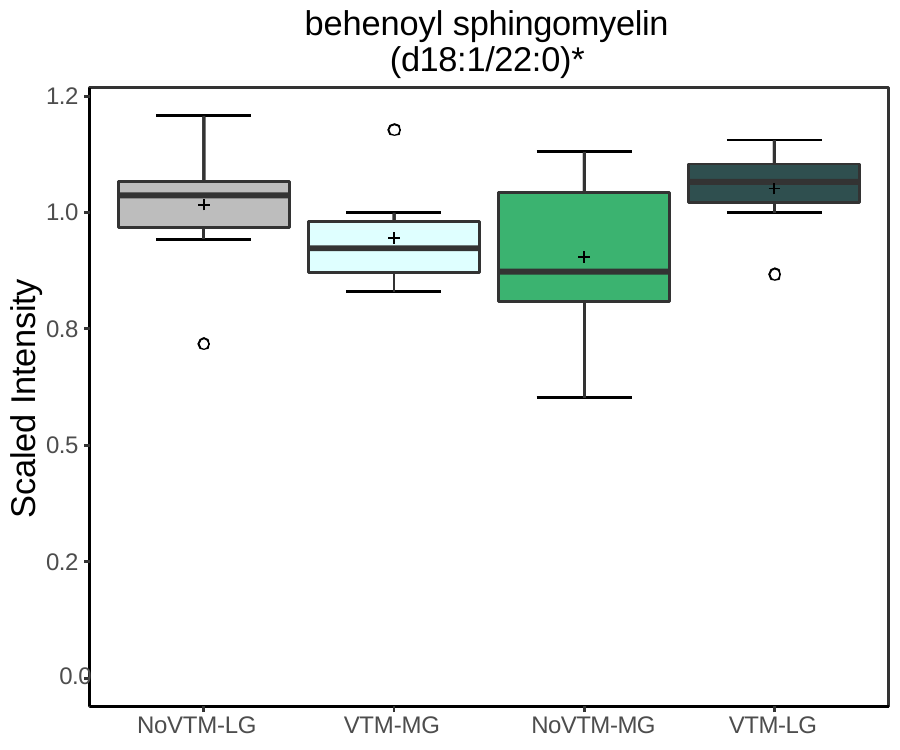

# behenoyl sphingomyelin (d18:1/22:0)*
1.2
1.0
Scaled Intensity
0.8
0.5
0.2
0.0
NoVTM-LG
VTM-MG
NoVTM-MG
VTM-LG

## Slide 323
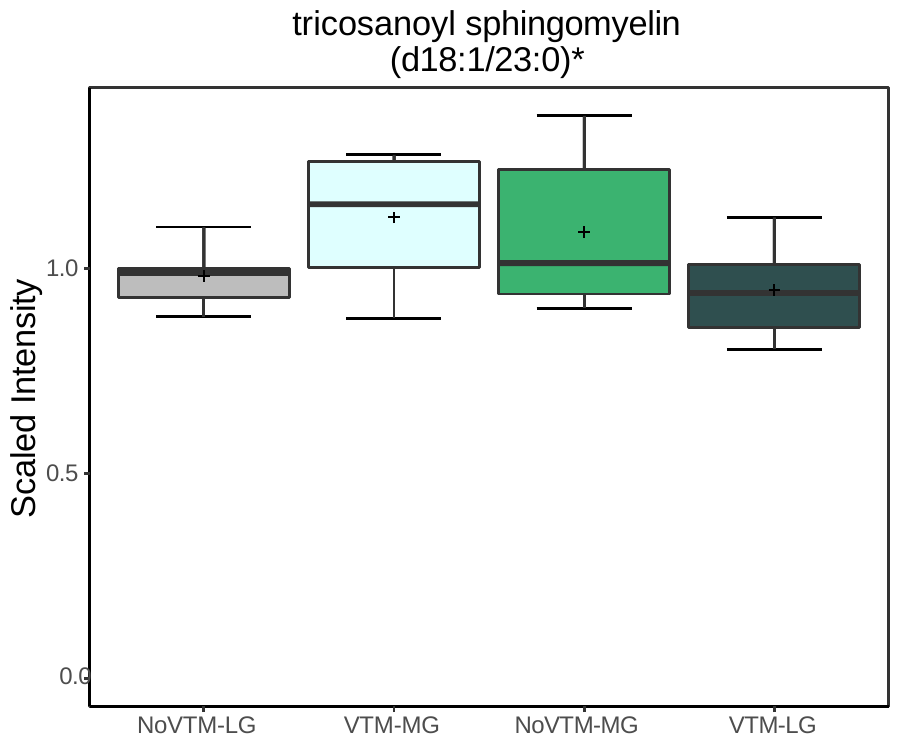

# tricosanoyl sphingomyelin (d18:1/23:0)*
1.0
Scaled Intensity
0.5
0.0
NoVTM-LG
VTM-MG
NoVTM-MG
VTM-LG

## Slide 324
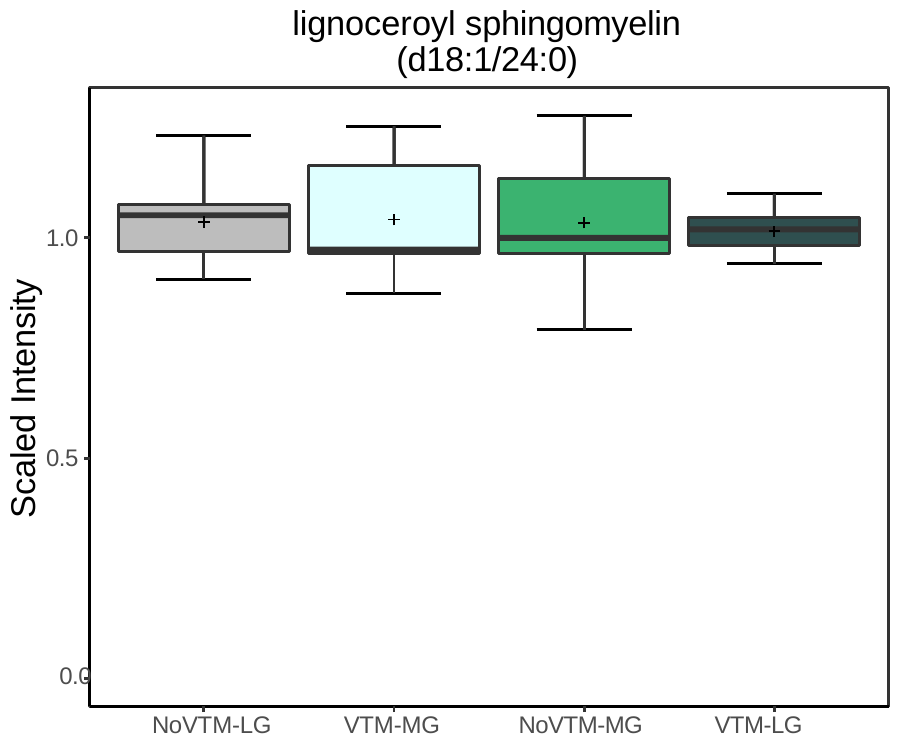

# lignoceroyl sphingomyelin (d18:1/24:0)
1.0
Scaled Intensity
0.5
0.0
NoVTM-LG
VTM-MG
NoVTM-MG
VTM-LG

## Slide 325
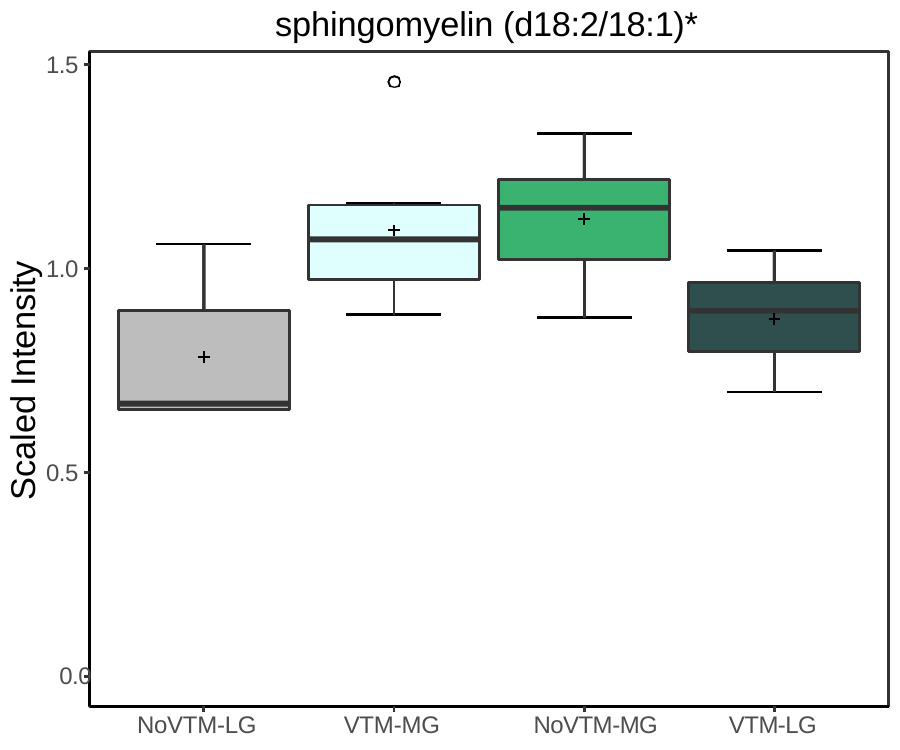

sphingomyelin (d18:2/18:1)*
1.5
1.0
Scaled Intensity
0.5
0.0
NoVTM-LG
VTM-MG
NoVTM-MG
VTM-LG

## Slide 326
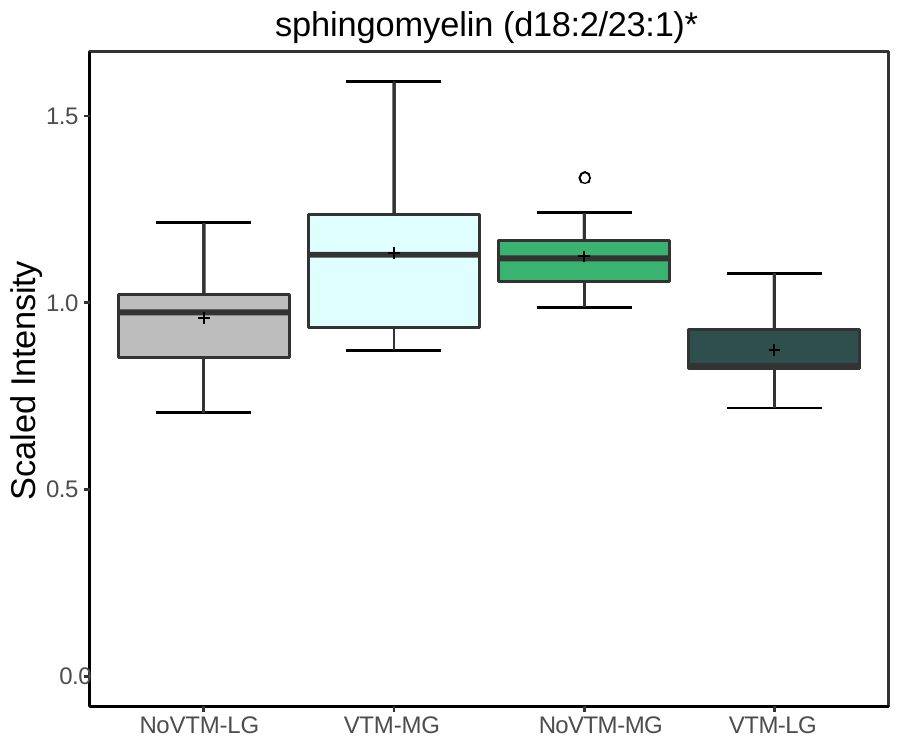

sphingomyelin (d18:2/23:1)*
1.5
Scaled Intensity
1.0
0.5
0.0
NoVTM-LG
VTM-MG
NoVTM-MG
VTM-LG

## Slide 327
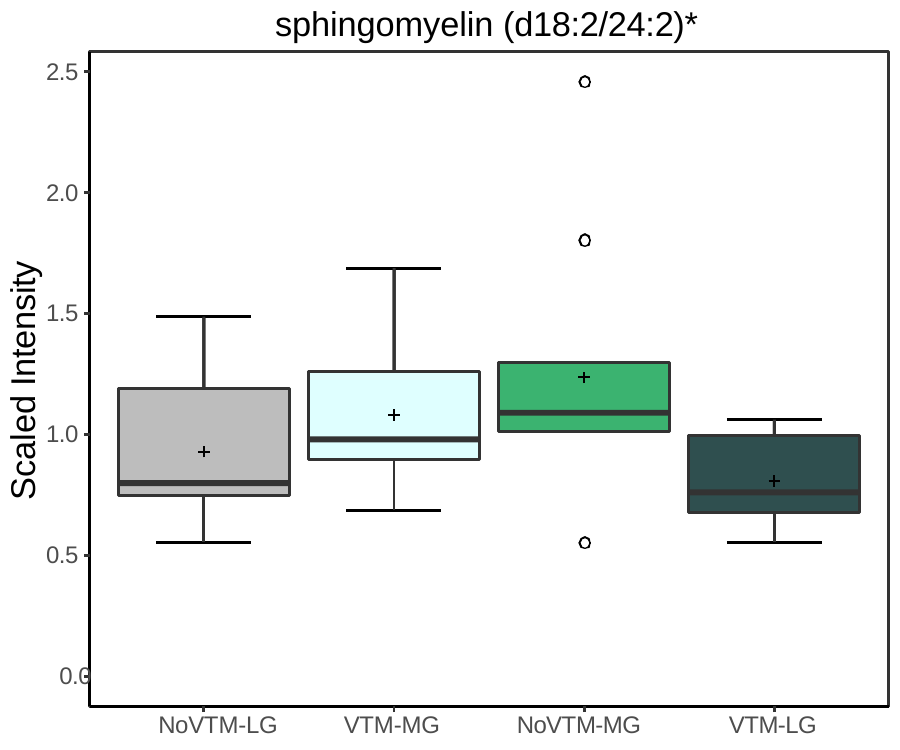

sphingomyelin (d18:2/24:2)*
2.5
2.0
Scaled Intensity
1.5
1.0
0.5
0.0
NoVTM-LG
VTM-MG
NoVTM-MG
VTM-LG

## Slide 328
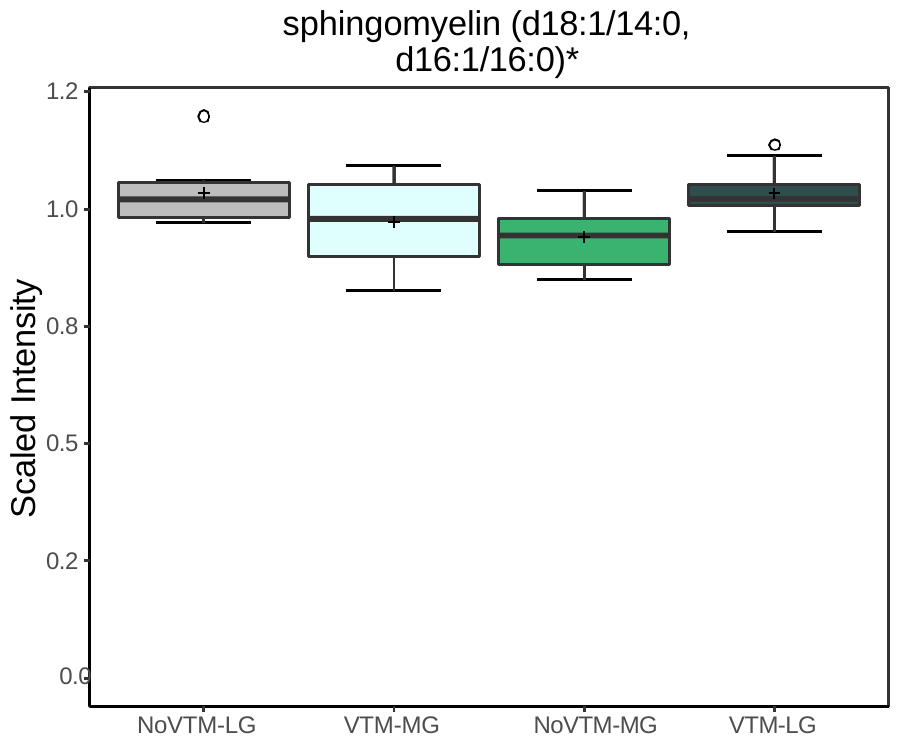

# sphingomyelin (d18:1/14:0, d16:1/16:0)*
1.2
1.0
Scaled Intensity
0.8
0.5
0.2
0.0
NoVTM-LG
VTM-MG
NoVTM-MG
VTM-LG

## Slide 329
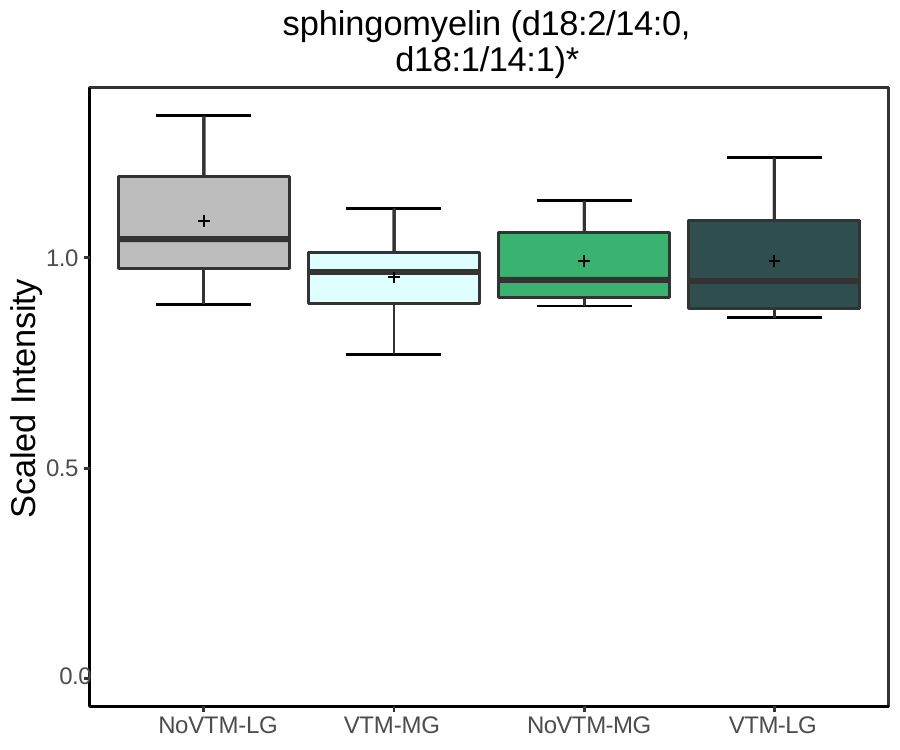

# sphingomyelin (d18:2/14:0, d18:1/14:1)*
1.0
Scaled Intensity
0.5
0.0
NoVTM-LG
VTM-MG
NoVTM-MG
VTM-LG

## Slide 330
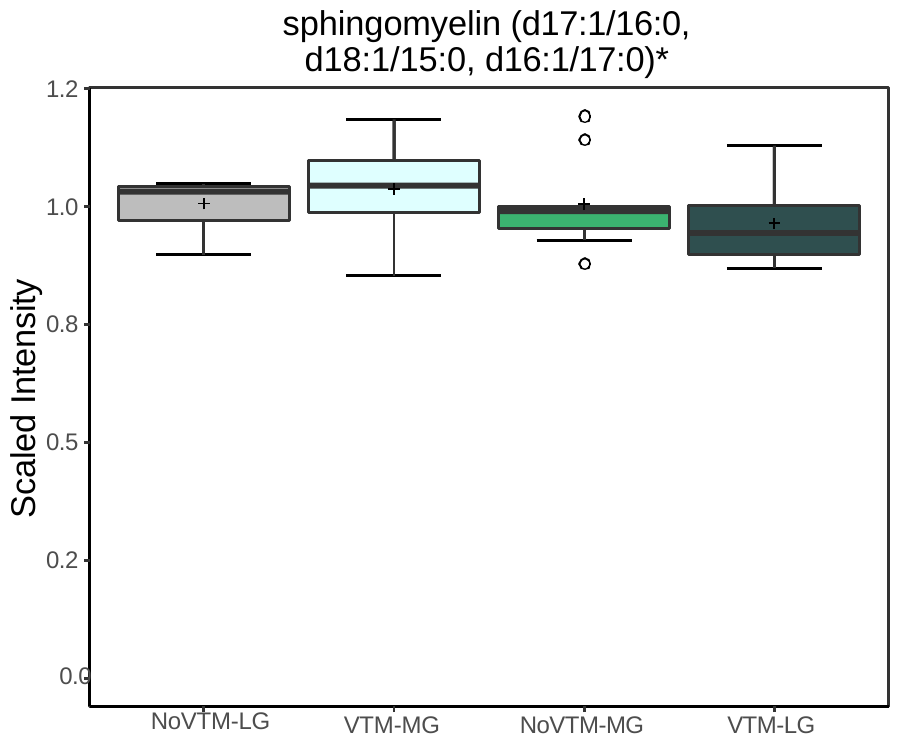

# sphingomyelin (d17:1/16:0, d18:1/15:0, d16:1/17:0)*
1.2
1.0
Scaled Intensity
0.8
0.5
0.2
0.0
NoVTM-LG
VTM-MG
NoVTM-MG
VTM-LG

## Slide 331
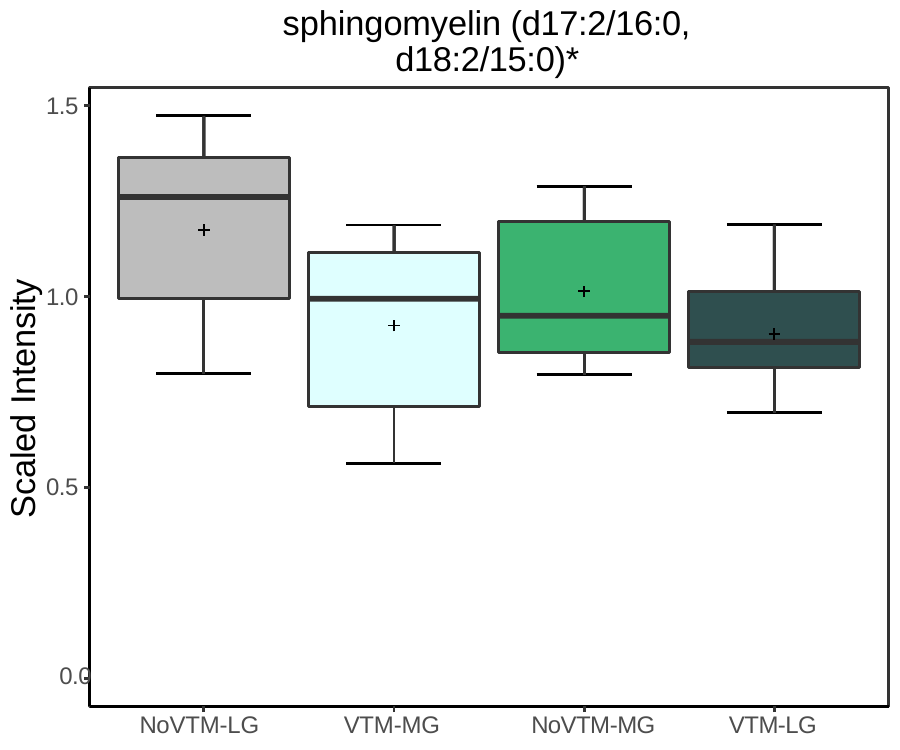

# sphingomyelin (d17:2/16:0, d18:2/15:0)*
1.5
Scaled Intensity
1.0
0.5
0.0
NoVTM-LG
VTM-MG
NoVTM-MG
VTM-LG

## Slide 332
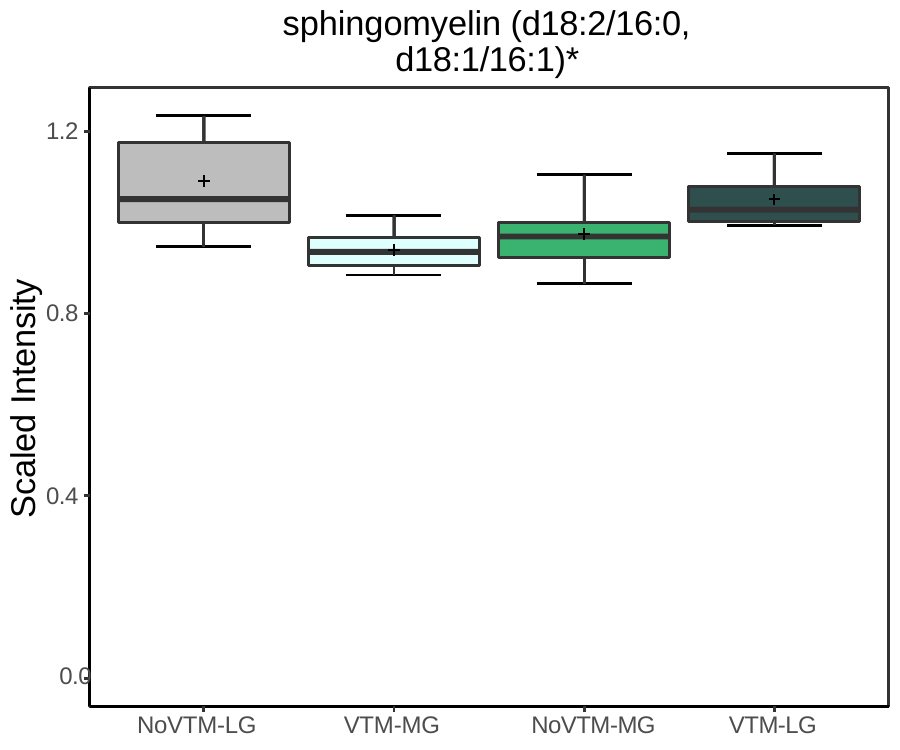

# sphingomyelin (d18:2/16:0, d18:1/16:1)*
1.2
Scaled Intensity
0.8
0.4
0.0
NoVTM-LG
VTM-MG
NoVTM-MG
VTM-LG

## Slide 333
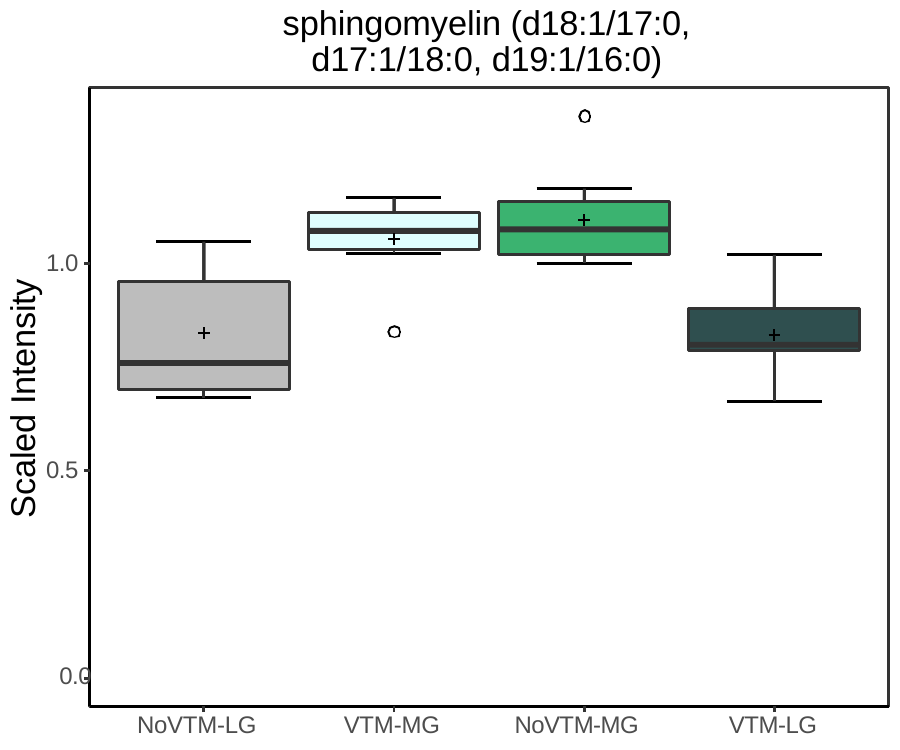

# sphingomyelin (d18:1/17:0, d17:1/18:0, d19:1/16:0)
1.0
Scaled Intensity
0.5
0.0
NoVTM-LG
VTM-MG
NoVTM-MG
VTM-LG

## Slide 334
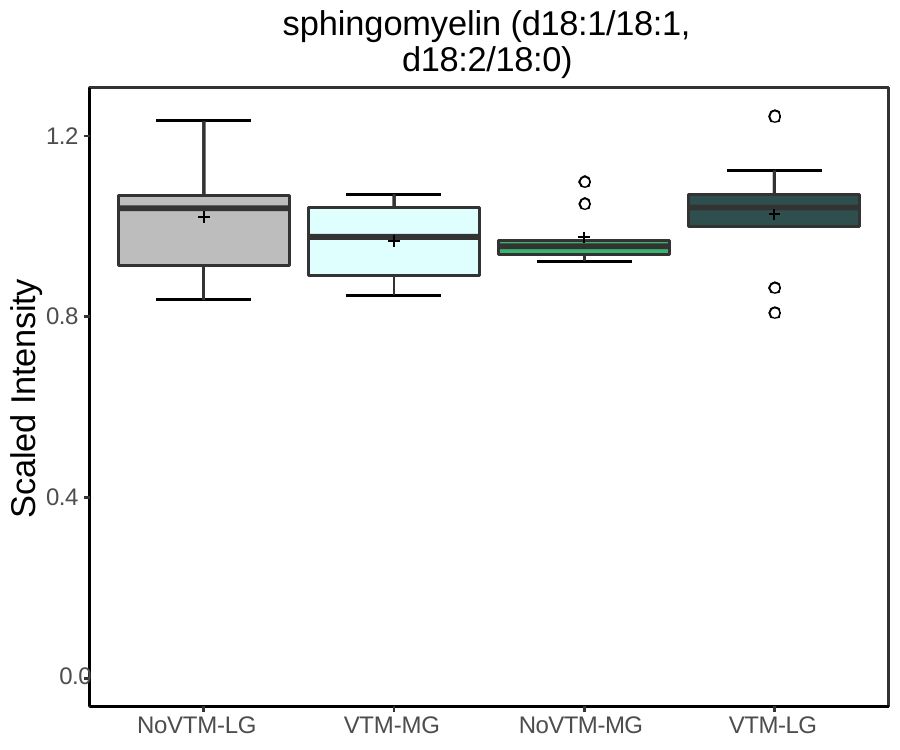

# sphingomyelin (d18:1/18:1, d18:2/18:0)
1.2
Scaled Intensity
0.8
0.4
0.0
NoVTM-LG
VTM-MG
NoVTM-MG
VTM-LG

## Slide 335
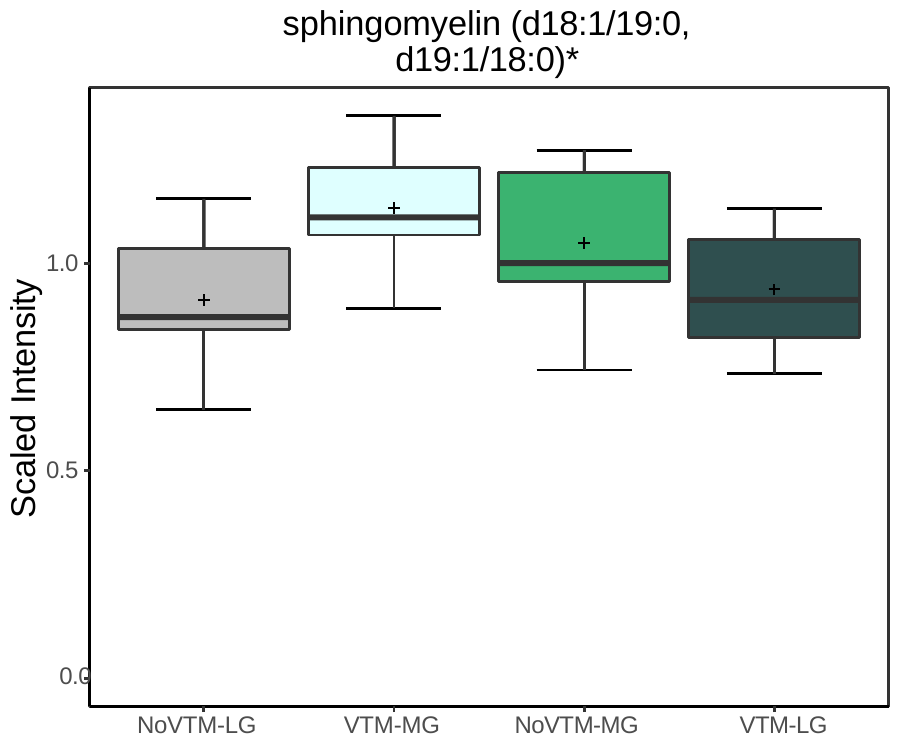

# sphingomyelin (d18:1/19:0, d19:1/18:0)*
1.0
Scaled Intensity
0.5
0.0
NoVTM-LG
VTM-MG
NoVTM-MG
VTM-LG

## Slide 336
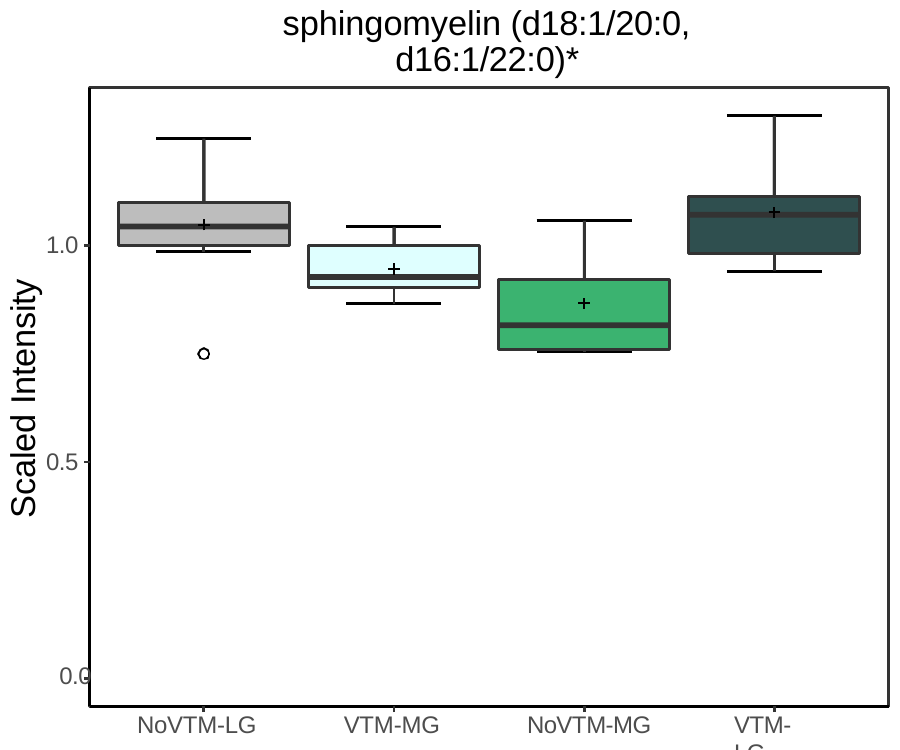

# sphingomyelin (d18:1/20:0, d16:1/22:0)*
1.0
Scaled Intensity
0.5
0.0
NoVTM-LG
VTM-MG
NoVTM-MG
VTM-LG

## Slide 337
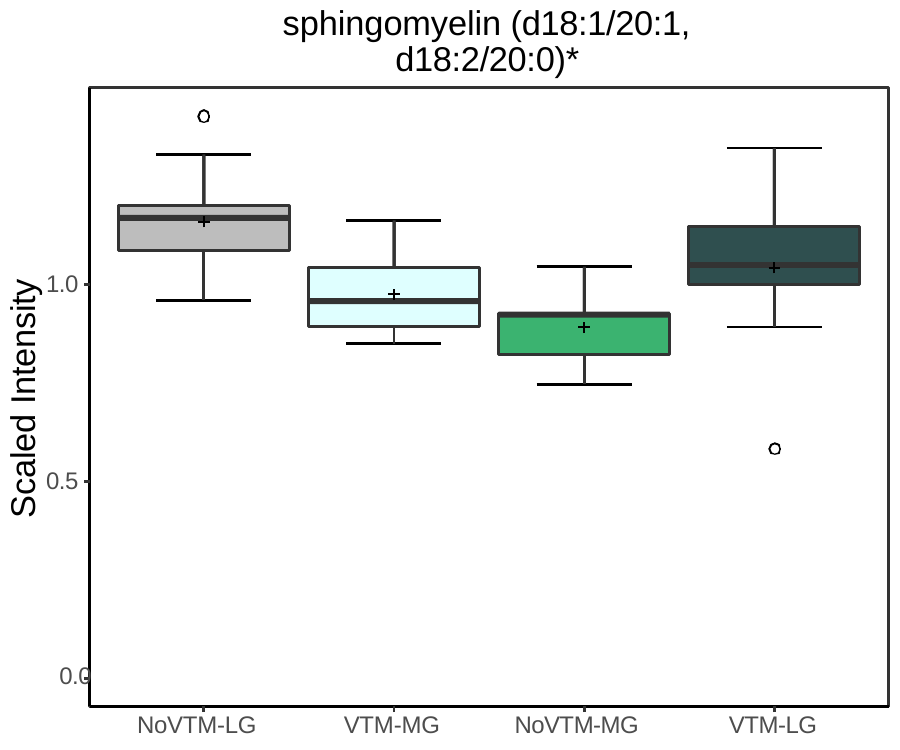

# sphingomyelin (d18:1/20:1, d18:2/20:0)*
1.0
Scaled Intensity
0.5
0.0
NoVTM-LG
VTM-MG
NoVTM-MG
VTM-LG

## Slide 338
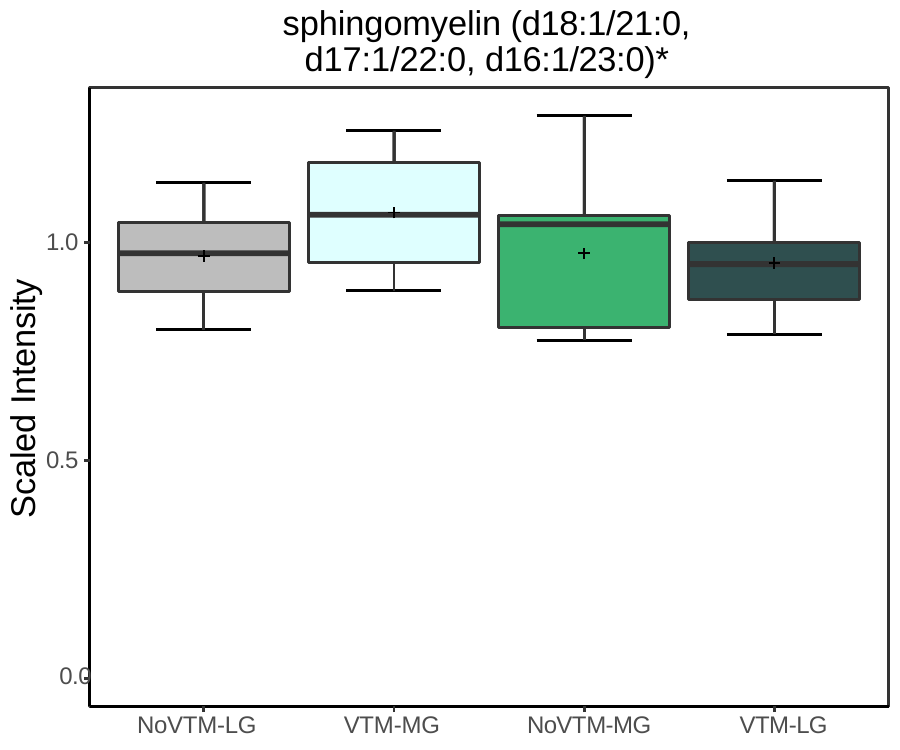

# sphingomyelin (d18:1/21:0, d17:1/22:0, d16:1/23:0)*
1.0
Scaled Intensity
0.5
0.0
NoVTM-LG
VTM-MG
NoVTM-MG
VTM-LG

## Slide 339
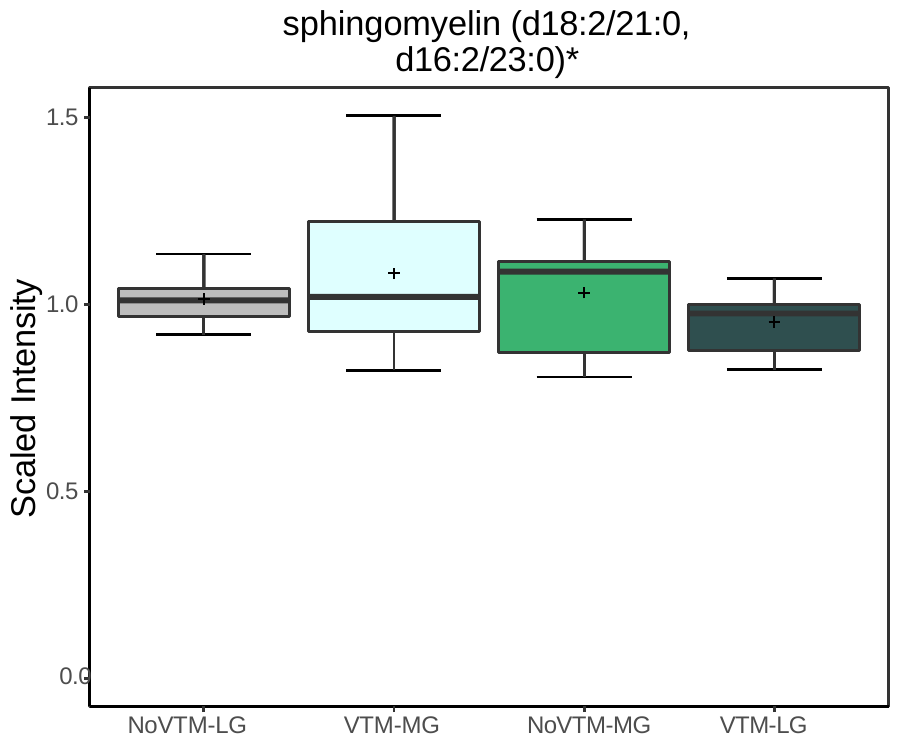

# sphingomyelin (d18:2/21:0, d16:2/23:0)*
1.5
Scaled Intensity
1.0
0.5
0.0
NoVTM-LG
VTM-MG
NoVTM-MG
VTM-LG

## Slide 340
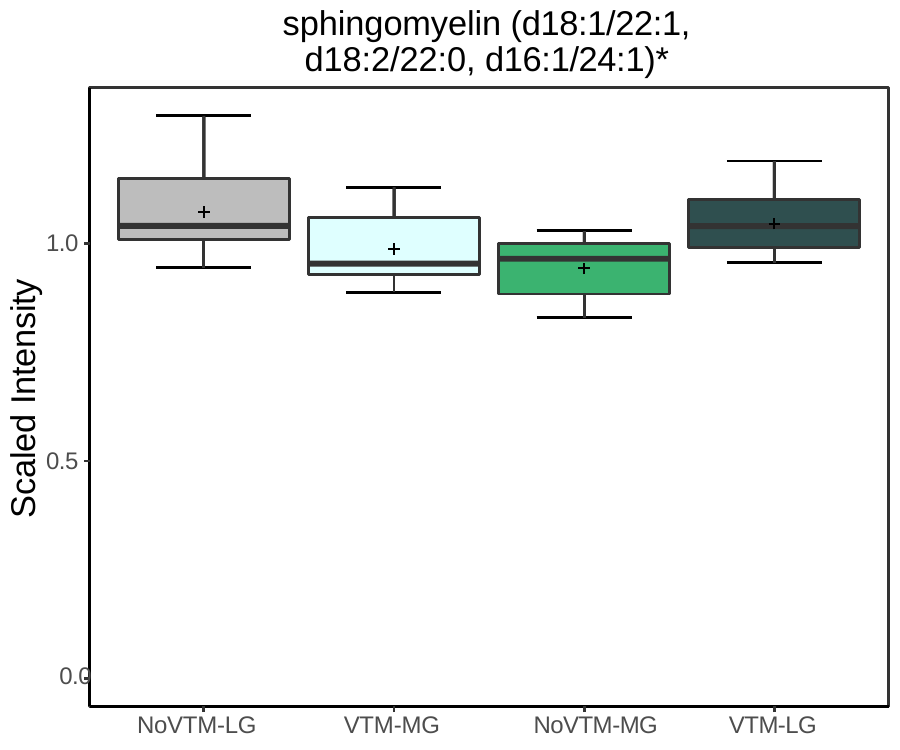

# sphingomyelin (d18:1/22:1, d18:2/22:0, d16:1/24:1)*
1.0
Scaled Intensity
0.5
0.0
NoVTM-LG
VTM-MG
NoVTM-MG
VTM-LG

## Slide 341
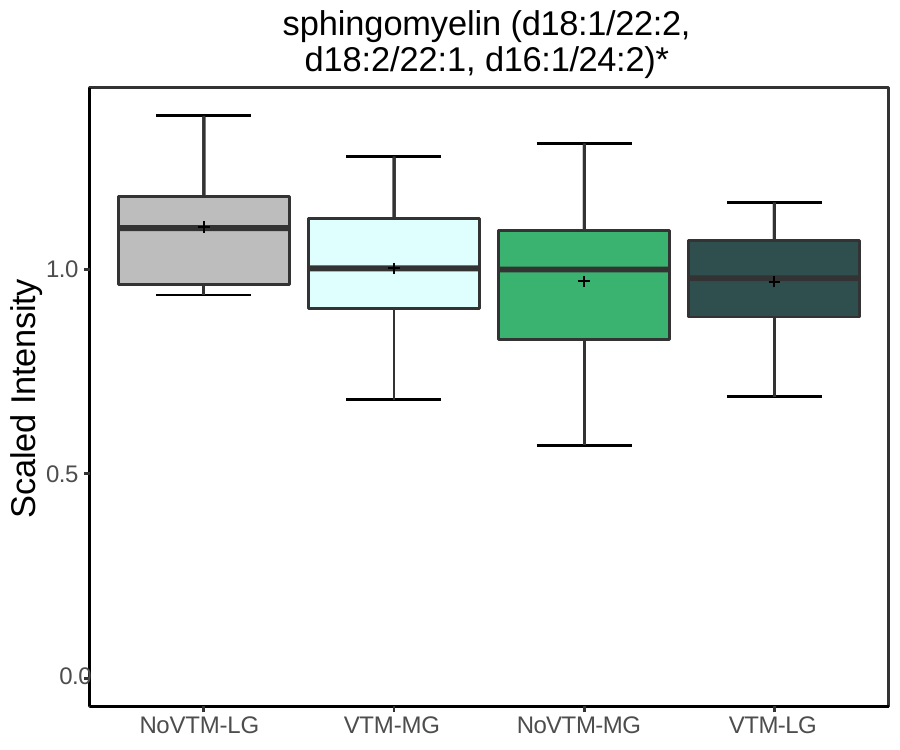

# sphingomyelin (d18:1/22:2, d18:2/22:1, d16:1/24:2)*
1.0
Scaled Intensity
0.5
0.0
NoVTM-LG
VTM-MG
NoVTM-MG
VTM-LG

## Slide 342
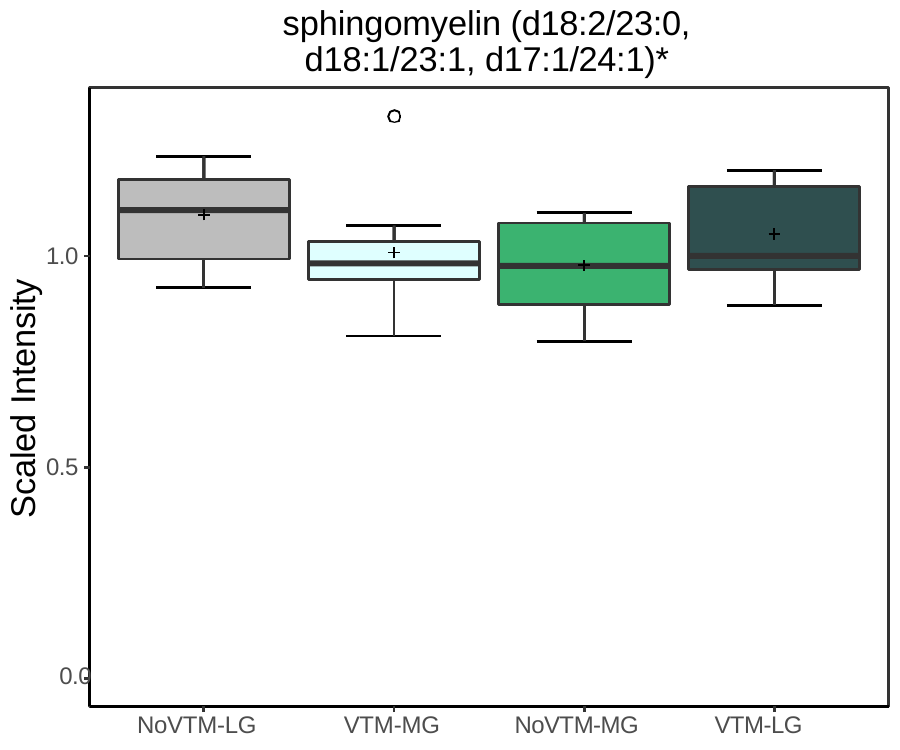

# sphingomyelin (d18:2/23:0, d18:1/23:1, d17:1/24:1)*
1.0
Scaled Intensity
0.5
0.0
NoVTM-LG
VTM-MG
NoVTM-MG
VTM-LG

## Slide 343
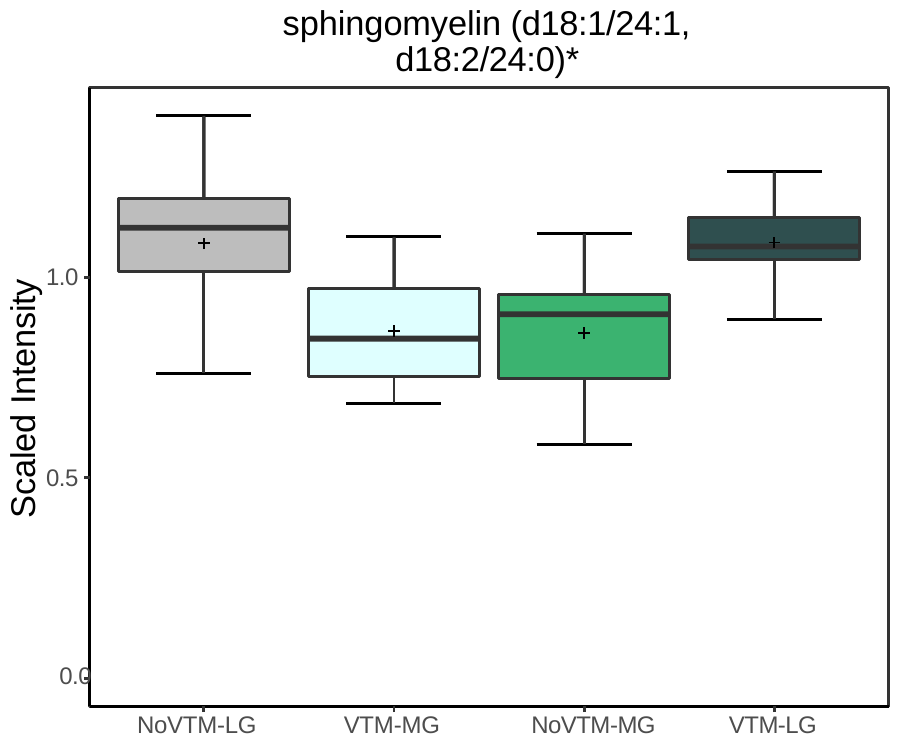

# sphingomyelin (d18:1/24:1, d18:2/24:0)*
1.0
Scaled Intensity
0.5
0.0
NoVTM-LG
VTM-MG
NoVTM-MG
VTM-LG

## Slide 344
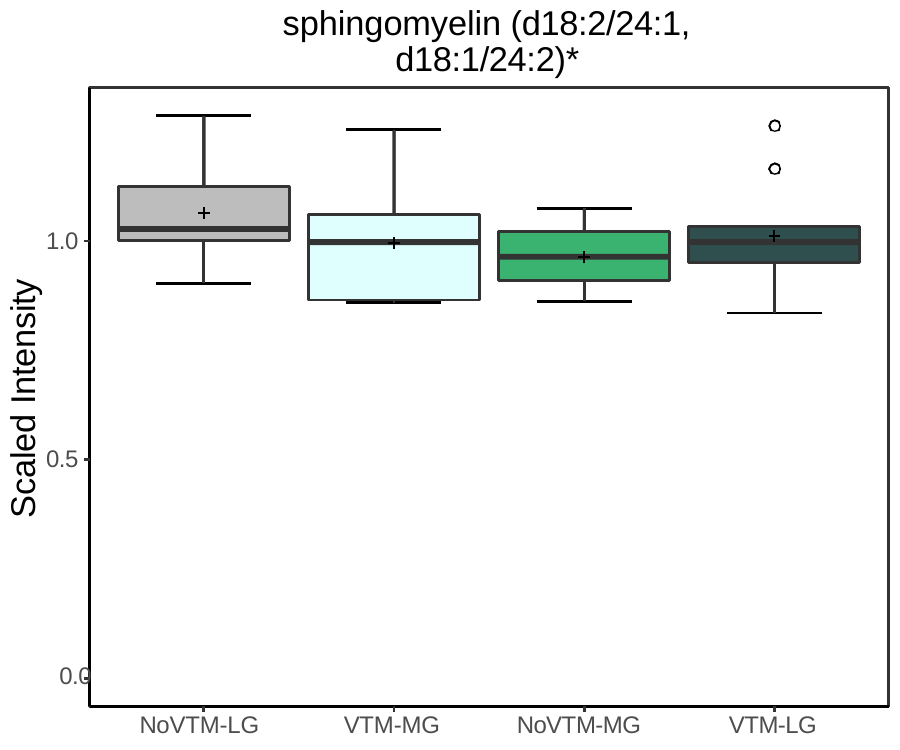

# sphingomyelin (d18:2/24:1, d18:1/24:2)*
1.0
Scaled Intensity
0.5
0.0
NoVTM-LG
VTM-MG
NoVTM-MG
VTM-LG

## Slide 345
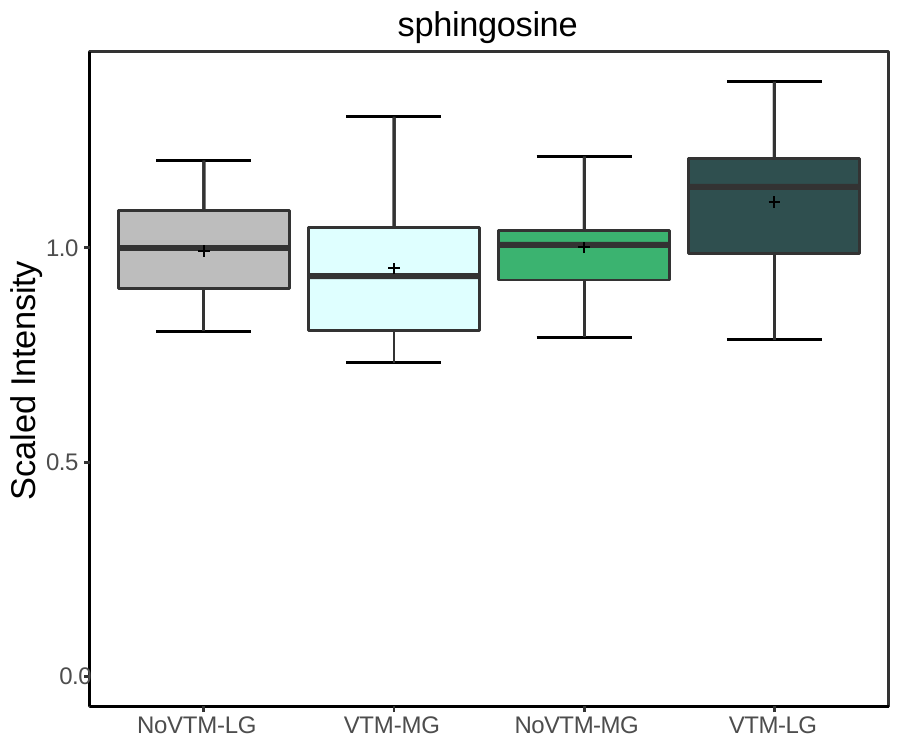

sphingosine
1.0
Scaled Intensity
0.5
0.0
NoVTM-LG
VTM-MG
NoVTM-MG
VTM-LG

## Slide 346
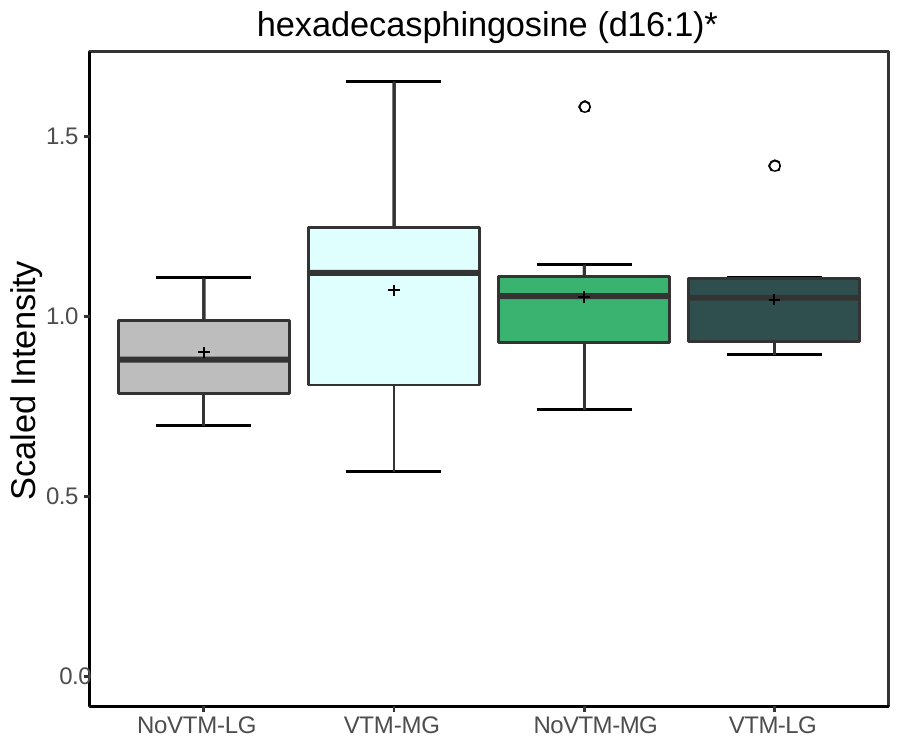

hexadecasphingosine (d16:1)*
1.5
Scaled Intensity
1.0
0.5
0.0
NoVTM-LG
VTM-MG
NoVTM-MG
VTM-LG

## Slide 347
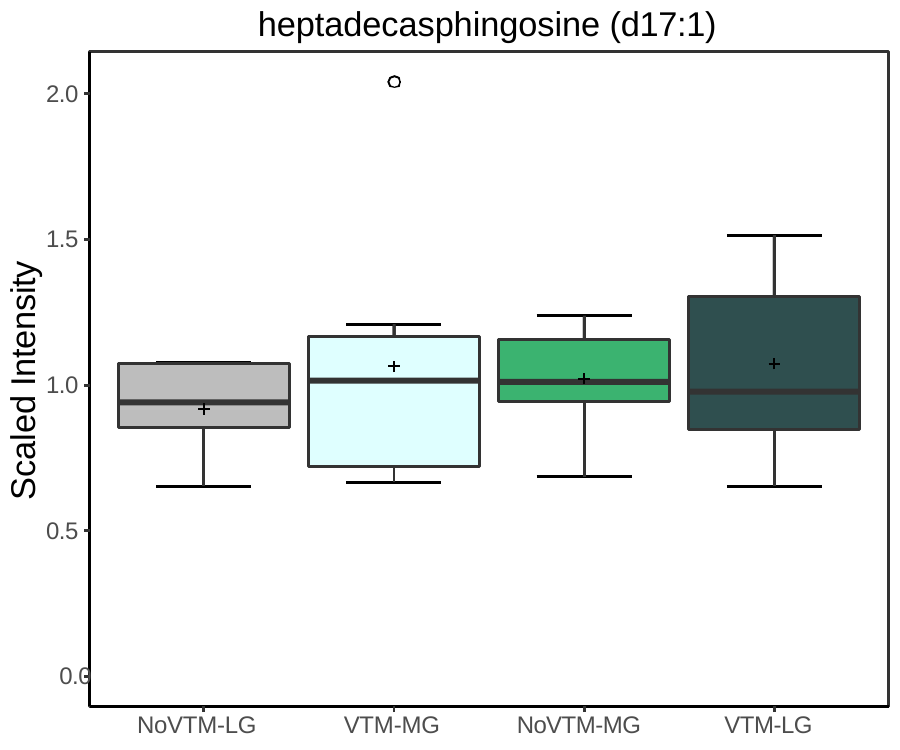

heptadecasphingosine (d17:1)
2.0
1.5
Scaled Intensity
1.0
0.5
0.0
NoVTM-LG
VTM-MG
NoVTM-MG
VTM-LG

## Slide 348
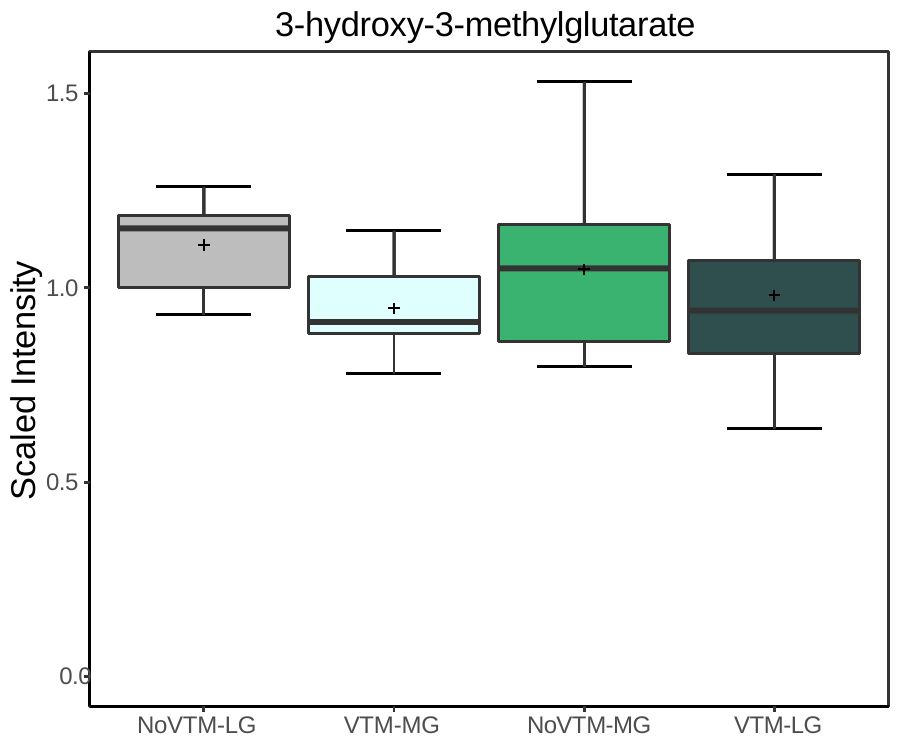

3-hydroxy-3-methylglutarate
1.5
Scaled Intensity
1.0
0.5
0.0
NoVTM-LG
VTM-MG
NoVTM-MG
VTM-LG

## Slide 349
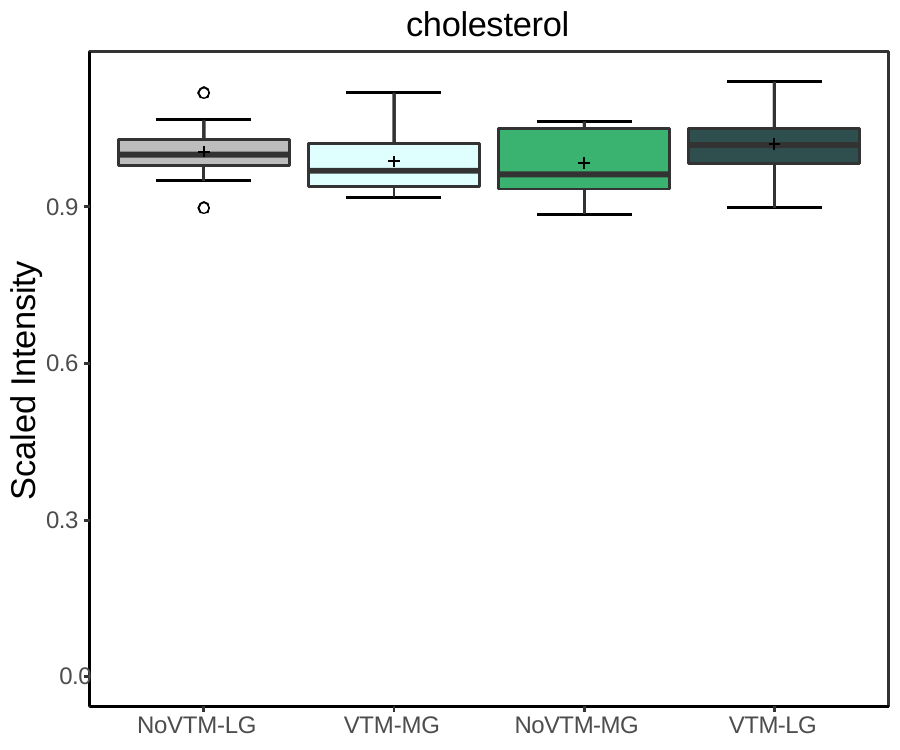

cholesterol
0.9
Scaled Intensity
0.6
0.3
0.0
NoVTM-LG
VTM-MG
NoVTM-MG
VTM-LG

## Slide 350
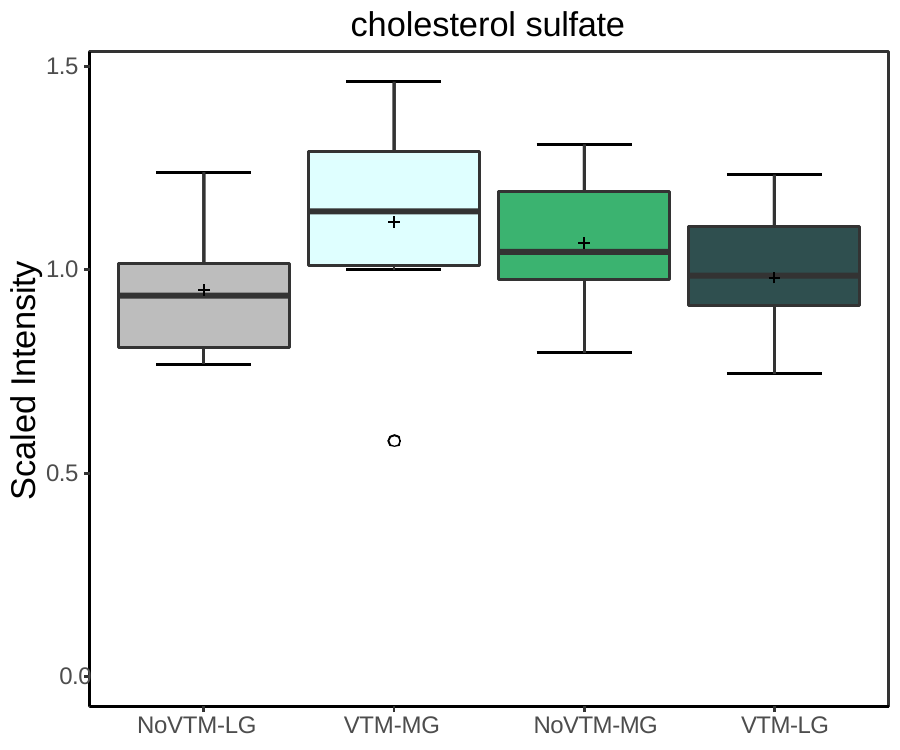

cholesterol sulfate
1.5
1.0
Scaled Intensity
0.5
0.0
NoVTM-LG
VTM-MG
NoVTM-MG
VTM-LG

## Slide 351
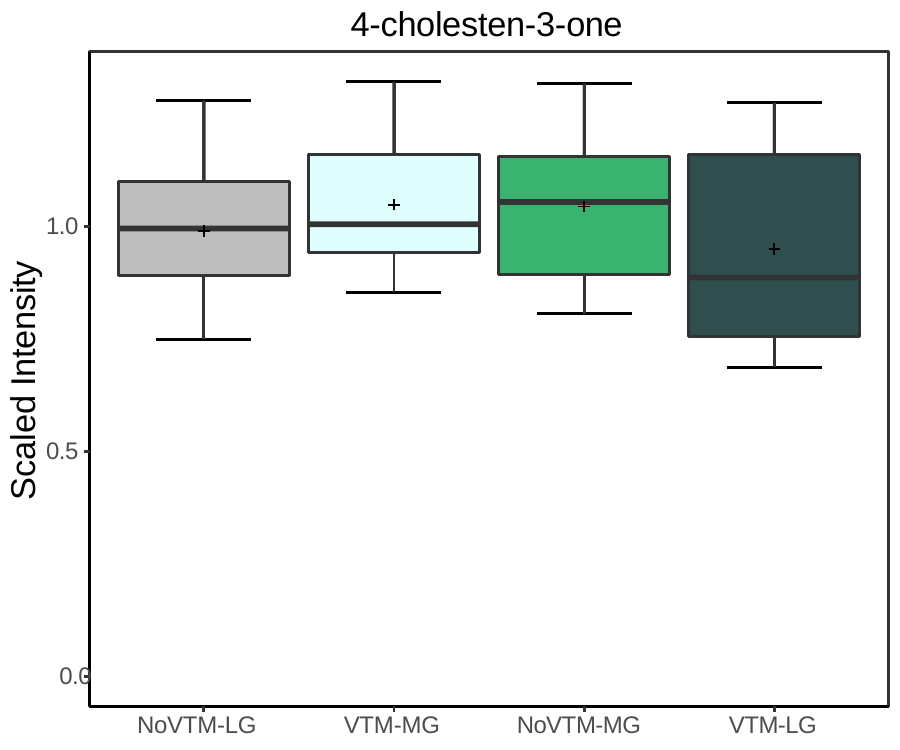

4-cholesten-3-one
1.0
Scaled Intensity
0.5
0.0
NoVTM-LG
VTM-MG
NoVTM-MG
VTM-LG

## Slide 352
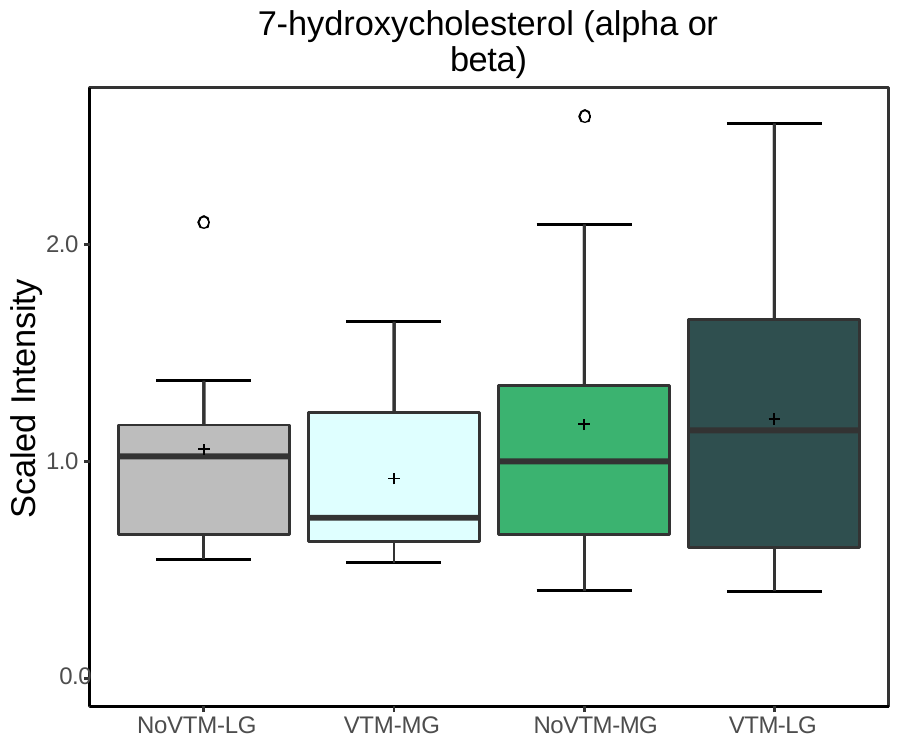

# 7-hydroxycholesterol (alpha or beta)
2.0
Scaled Intensity
1.0
0.0
NoVTM-LG
VTM-MG
NoVTM-MG
VTM-LG

## Slide 353
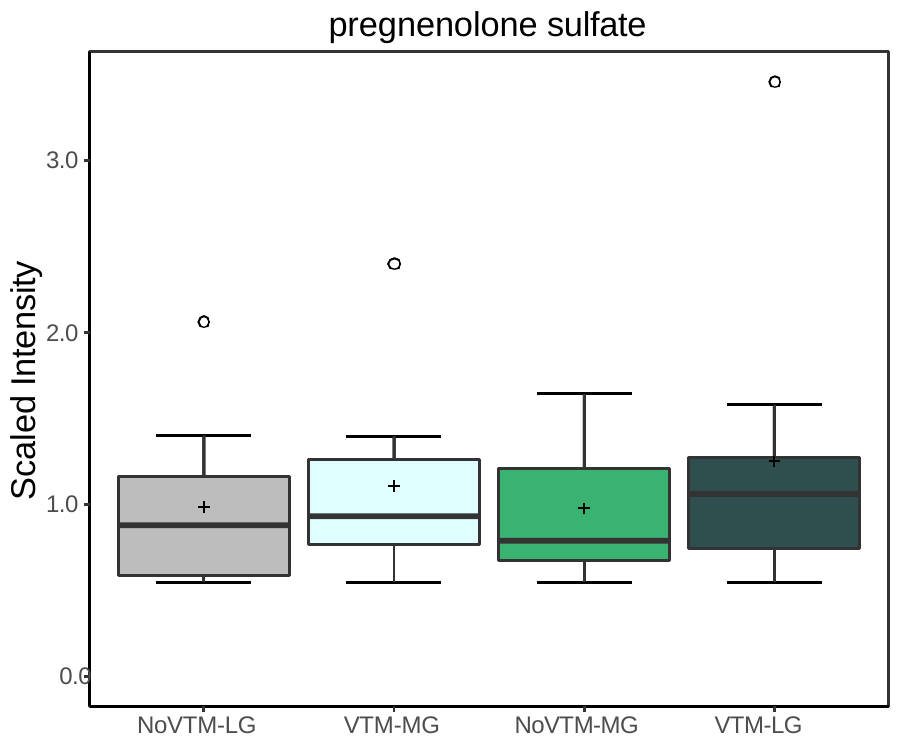

pregnenolone sulfate
3.0
Scaled Intensity
2.0
1.0
0.0
NoVTM-LG
VTM-MG
NoVTM-MG
VTM-LG

## Slide 354
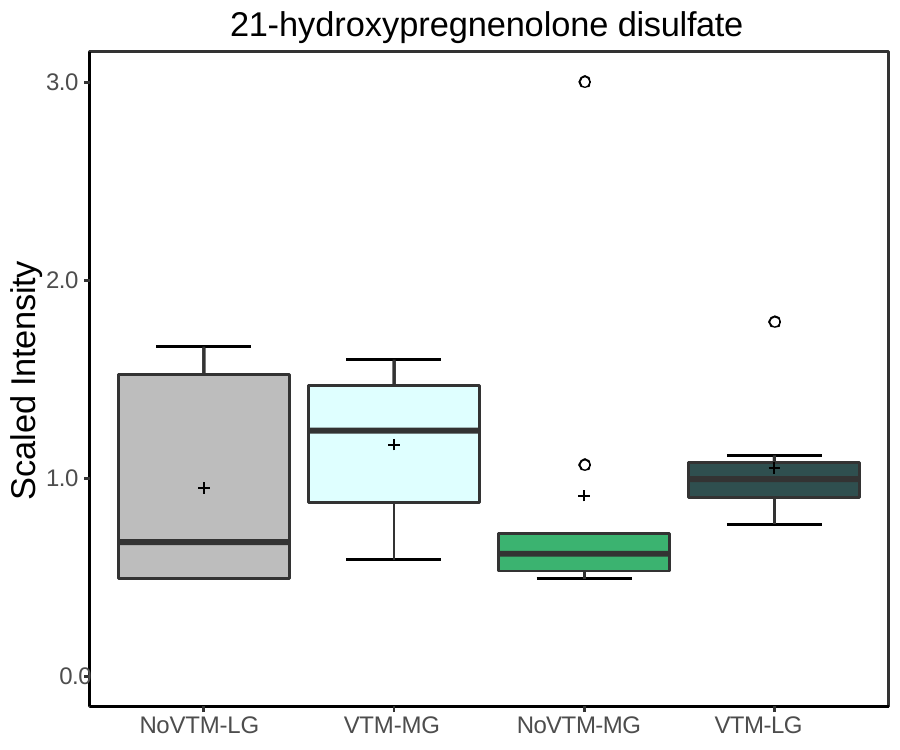

21-hydroxypregnenolone disulfate
3.0
Scaled Intensity
2.0
1.0
0.0
NoVTM-LG
VTM-MG
NoVTM-MG
VTM-LG

## Slide 355
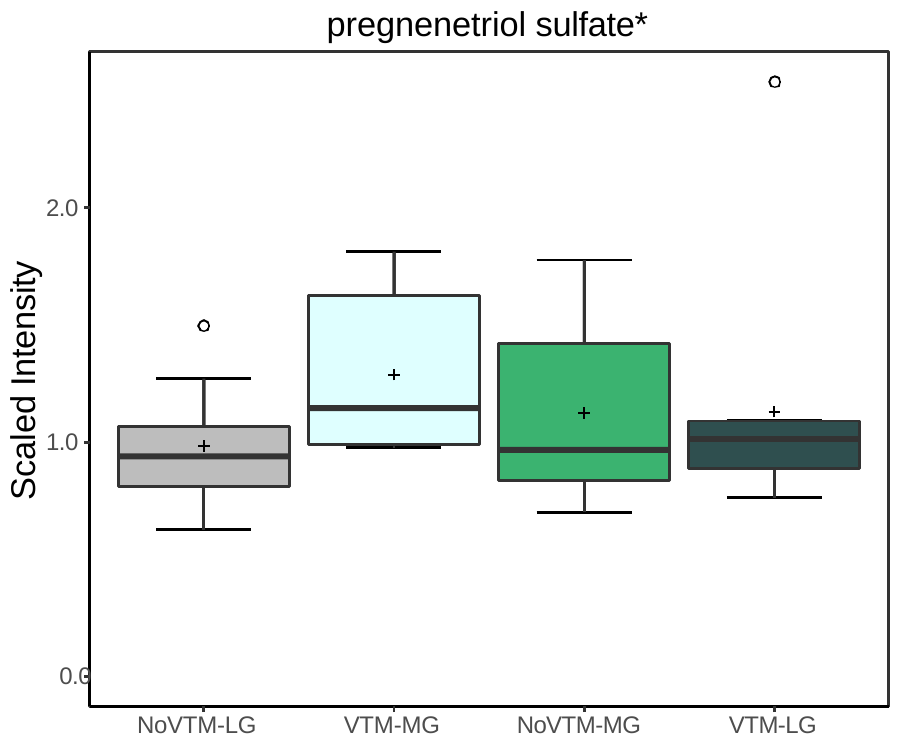

pregnenetriol sulfate*
2.0
Scaled Intensity
1.0
0.0
NoVTM-LG
VTM-MG
NoVTM-MG
VTM-LG

## Slide 356
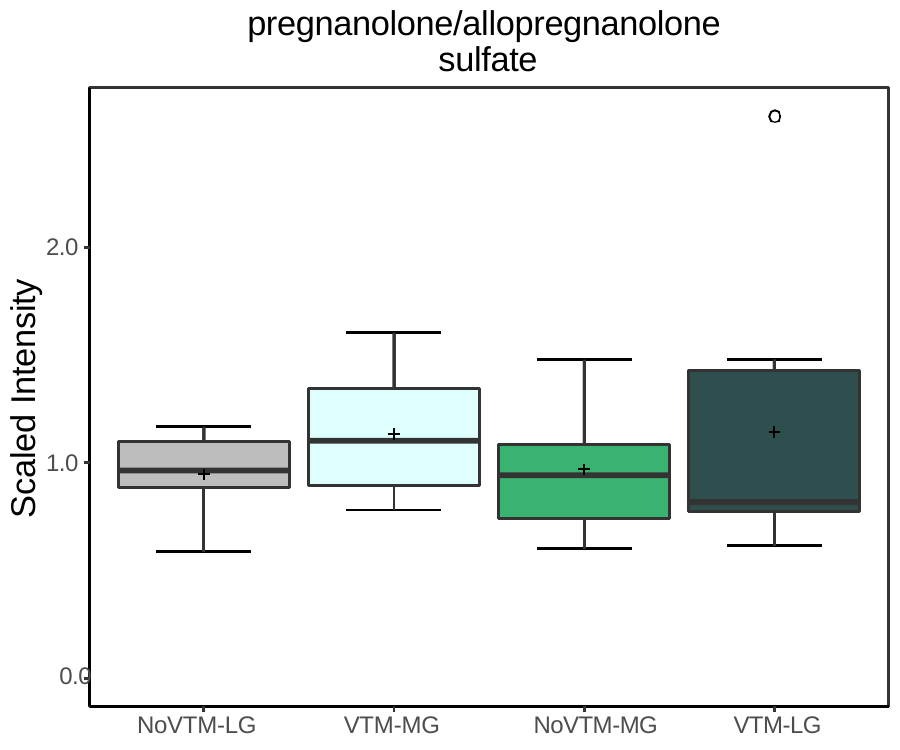

# pregnanolone/allopregnanolone sulfate
2.0
Scaled Intensity
1.0
0.0
NoVTM-LG
VTM-MG
NoVTM-MG
VTM-LG

## Slide 357
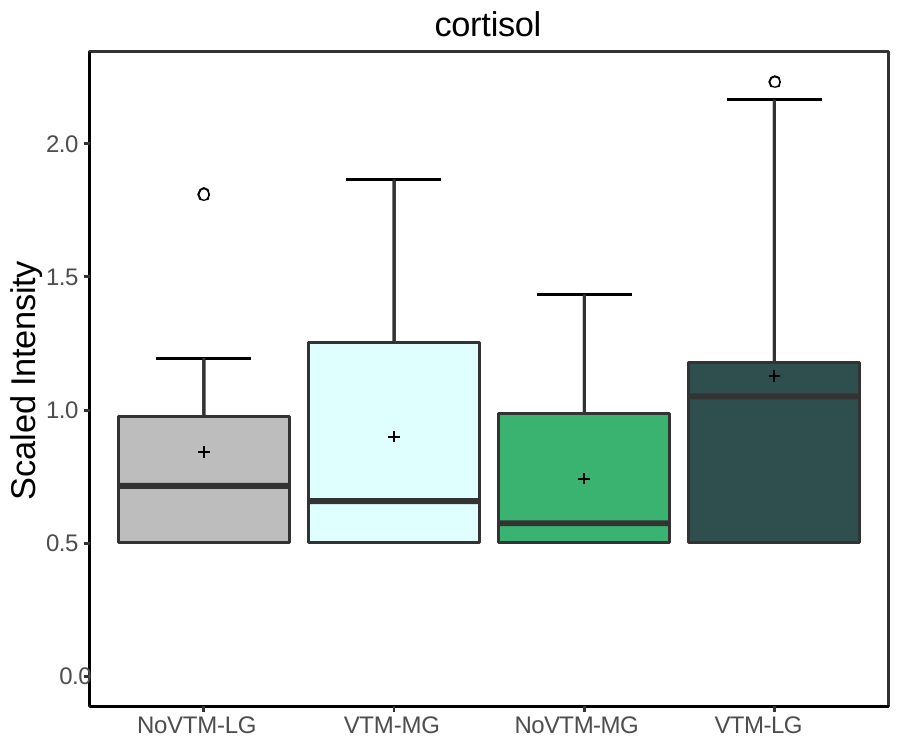

cortisol
2.0
Scaled Intensity
1.5
1.0
0.5
0.0
NoVTM-LG
VTM-MG
NoVTM-MG
VTM-LG

## Slide 358
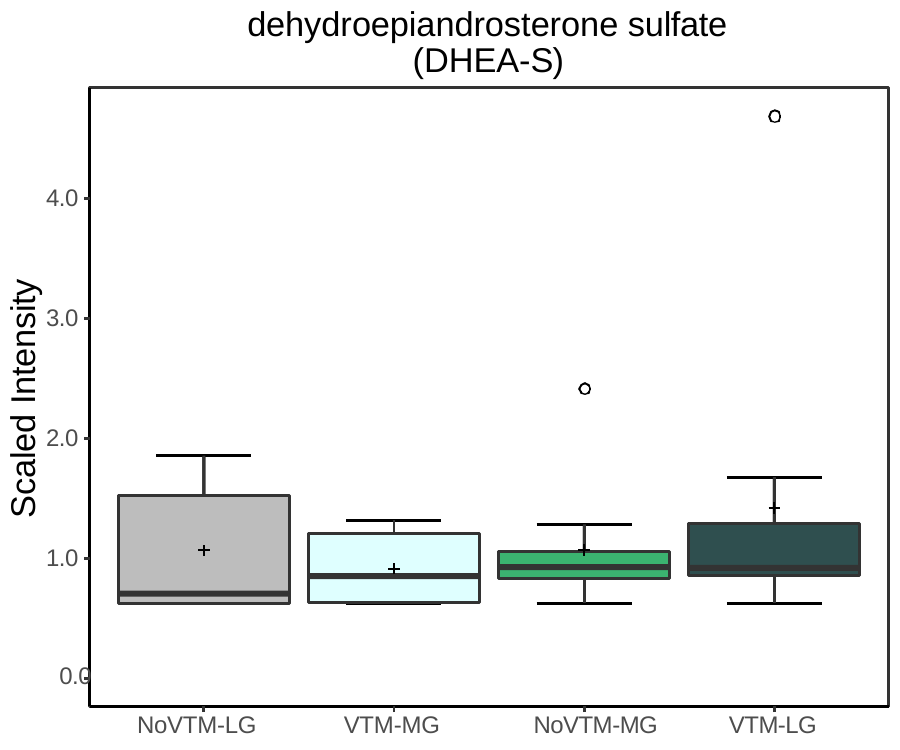

# dehydroepiandrosterone sulfate (DHEA-S)
4.0
Scaled Intensity
3.0
2.0
1.0
0.0
NoVTM-LG
VTM-MG
NoVTM-MG
VTM-LG

## Slide 359
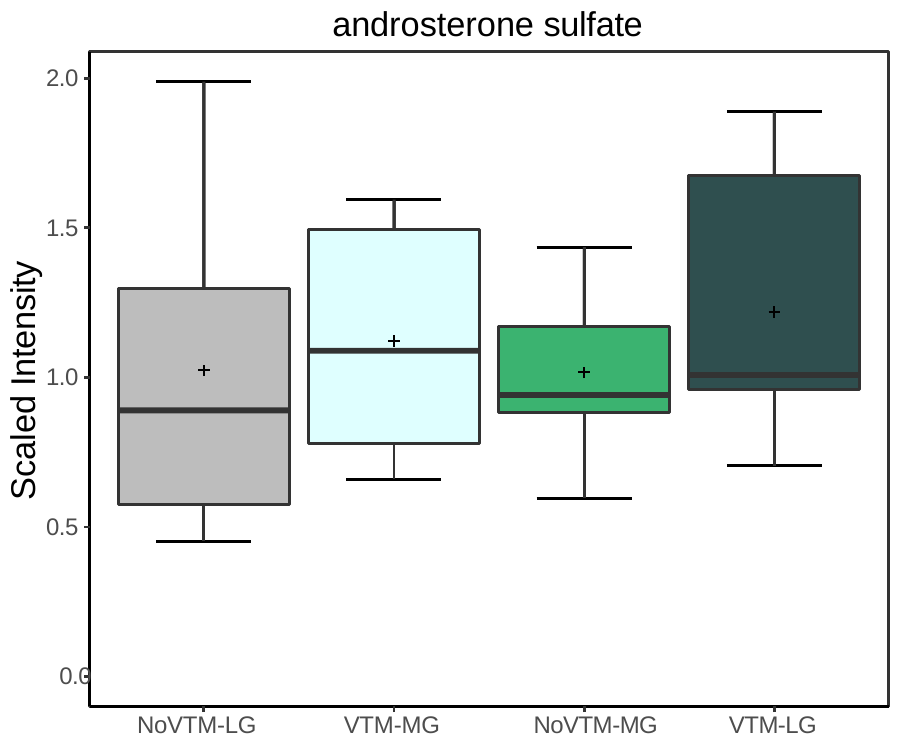

androsterone sulfate
2.0
1.5
Scaled Intensity
1.0
0.5
0.0
NoVTM-LG
VTM-MG
NoVTM-MG
VTM-LG

## Slide 360
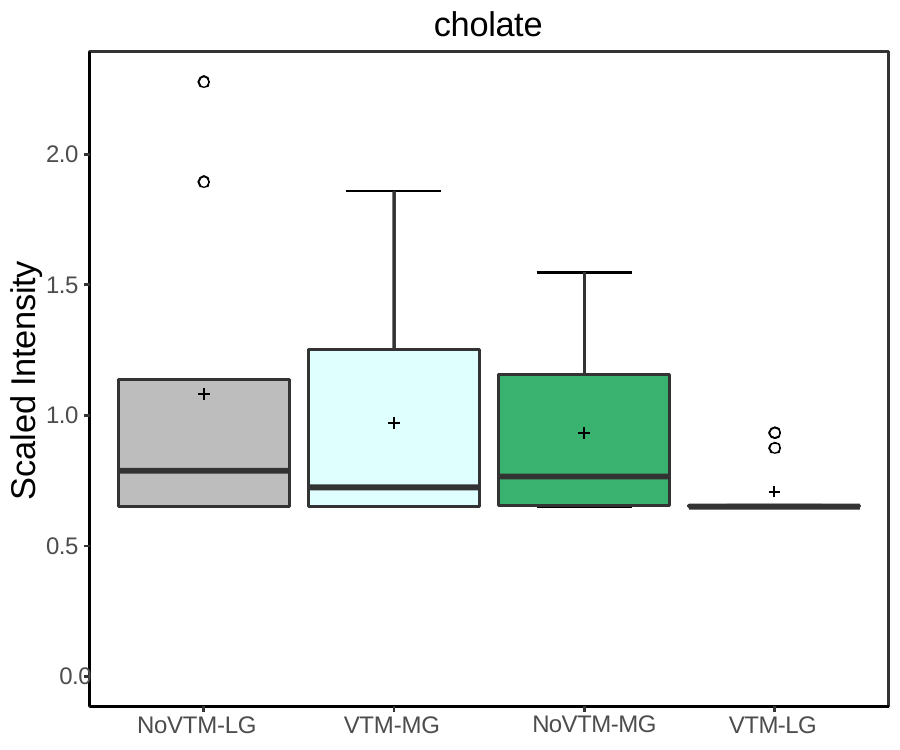

cholate
2.0
Scaled Intensity
1.5
1.0
0.5
0.0
NoVTM-MG
NoVTM-LG
VTM-MG
VTM-LG

## Slide 361
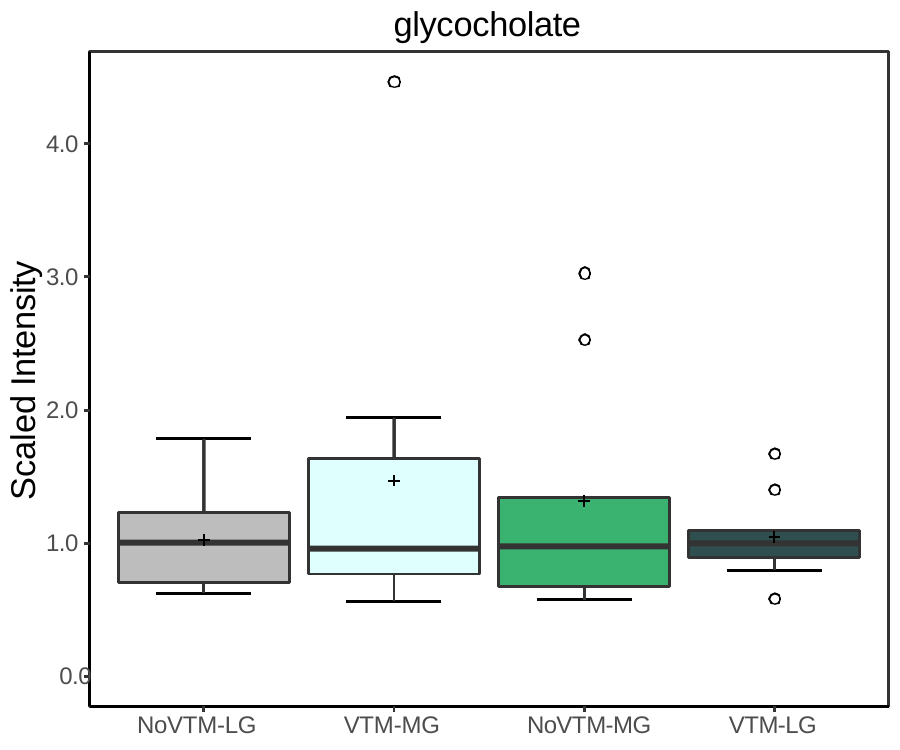

glycocholate
4.0
Scaled Intensity
3.0
2.0
1.0
0.0
NoVTM-LG
VTM-MG
NoVTM-MG
VTM-LG

## Slide 362
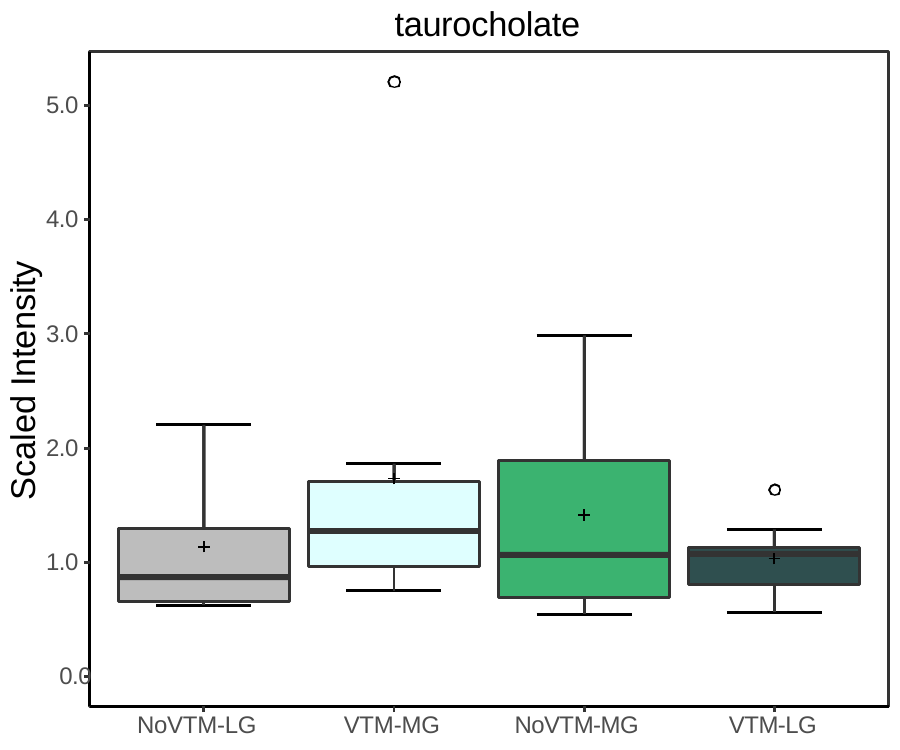

taurocholate
5.0
4.0
Scaled Intensity
3.0
2.0
1.0
0.0
NoVTM-LG
VTM-MG
NoVTM-MG
VTM-LG

## Slide 363
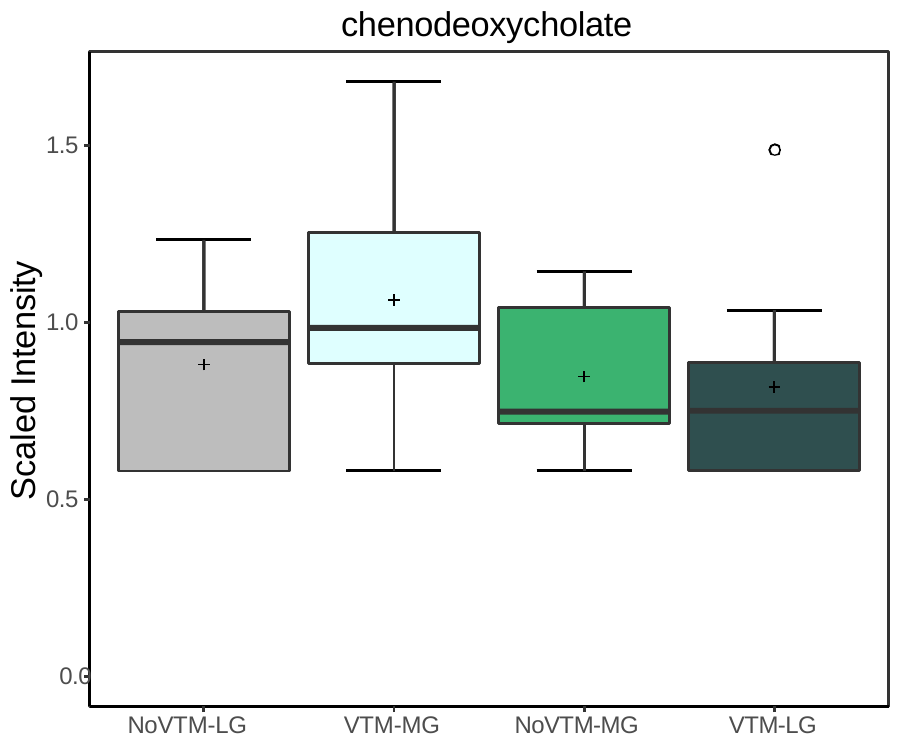

chenodeoxycholate
1.5
Scaled Intensity
1.0
0.5
0.0
NoVTM-LG
VTM-MG
NoVTM-MG
VTM-LG

## Slide 364
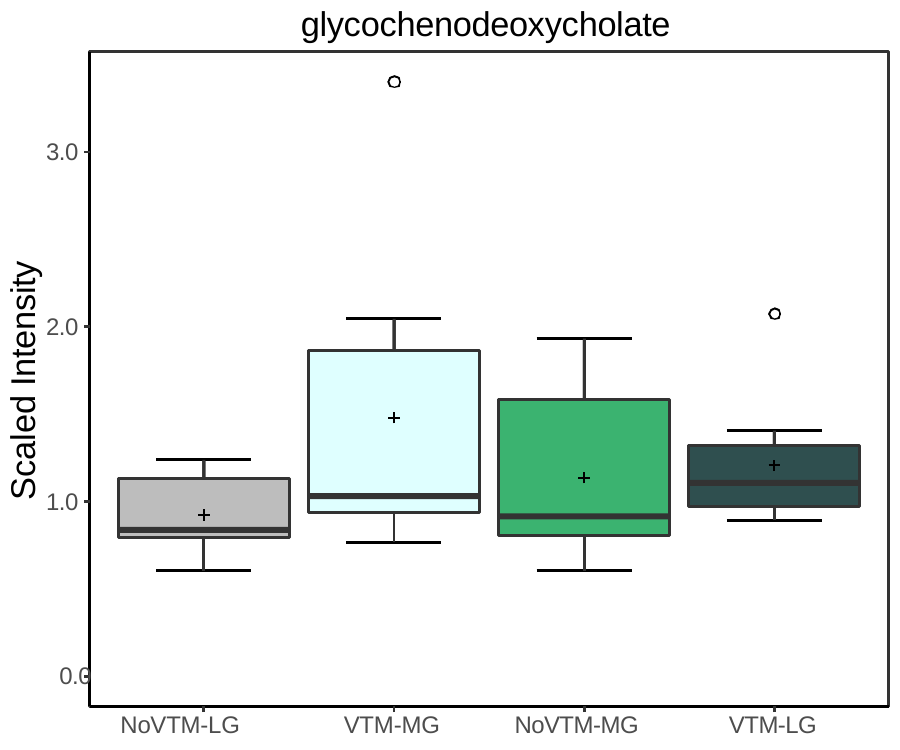

glycochenodeoxycholate
3.0
Scaled Intensity
2.0
1.0
0.0
NoVTM-LG
VTM-MG
NoVTM-MG
VTM-LG

## Slide 365
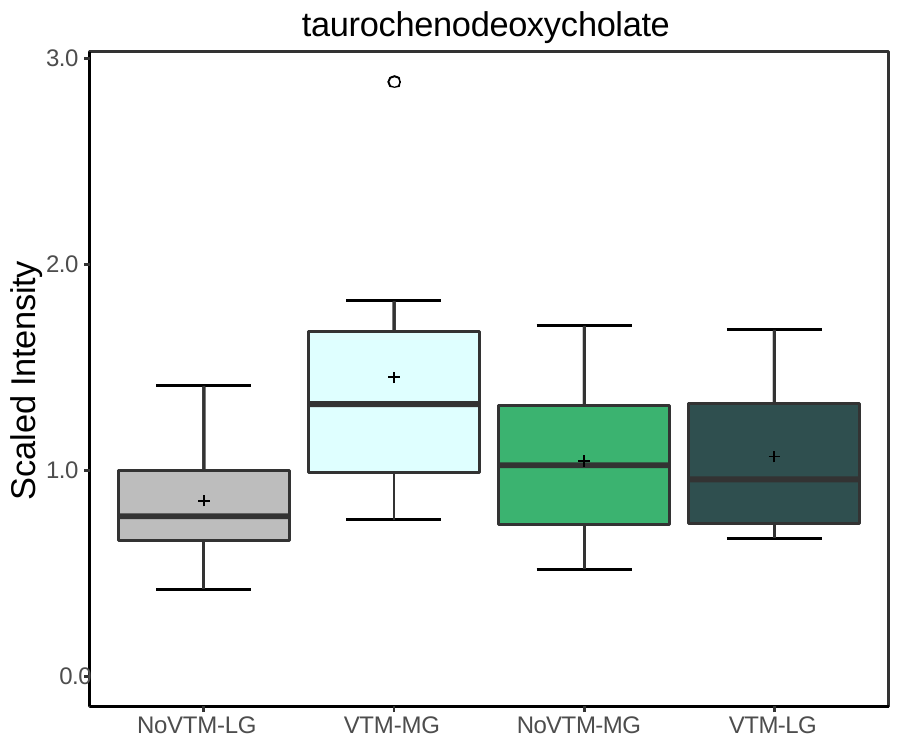

taurochenodeoxycholate
3.0
2.0
Scaled Intensity
1.0
0.0
NoVTM-LG
VTM-MG
NoVTM-MG
VTM-LG

## Slide 366
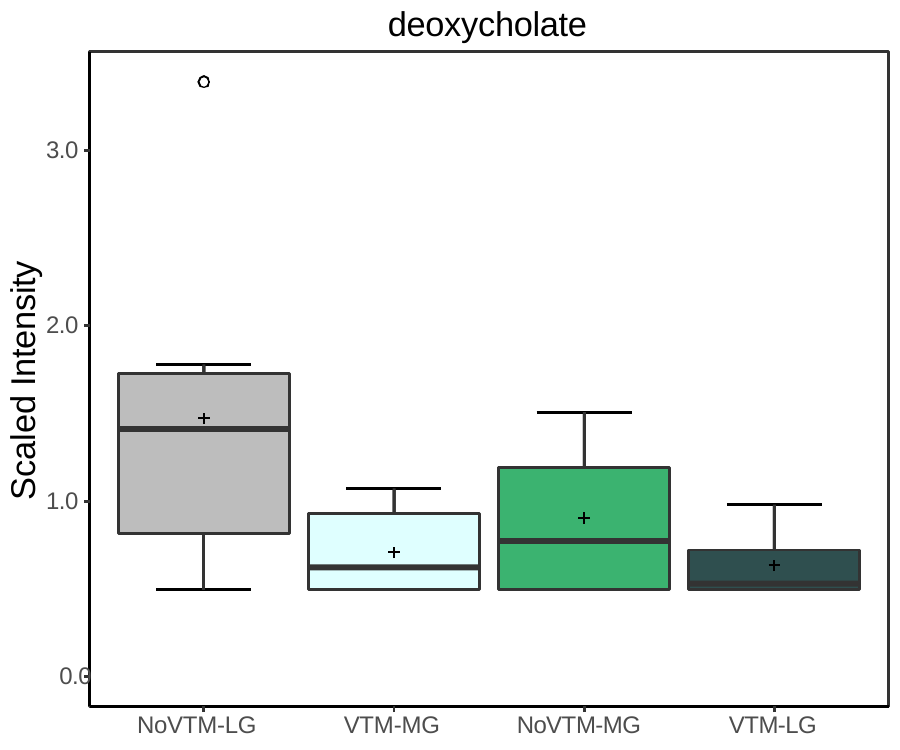

deoxycholate
3.0
Scaled Intensity
2.0
1.0
0.0
NoVTM-LG
VTM-MG
NoVTM-MG
VTM-LG

## Slide 367
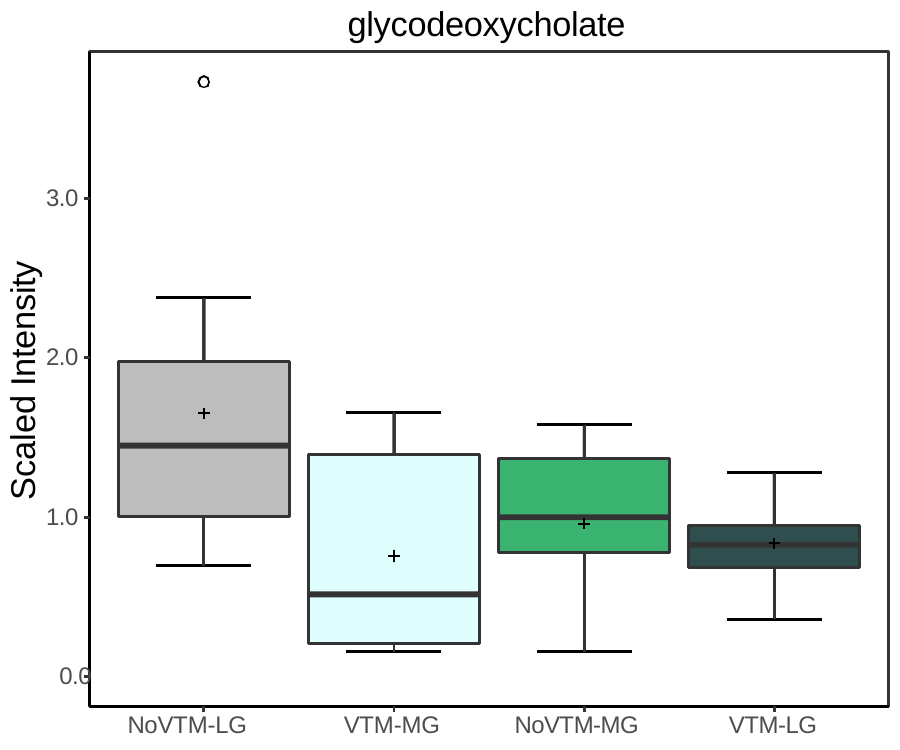

glycodeoxycholate
3.0
Scaled Intensity
2.0
1.0
0.0
NoVTM-LG
VTM-MG
NoVTM-MG
VTM-LG

## Slide 368
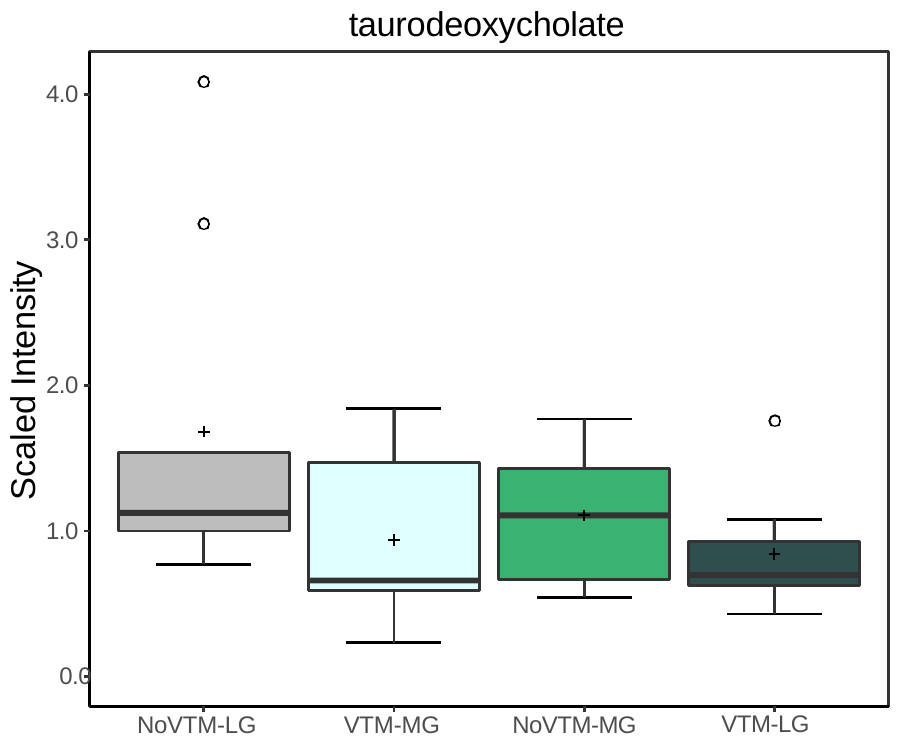

taurodeoxycholate
4.0
3.0
Scaled Intensity
2.0
1.0
0.0
VTM-LG
NoVTM-LG
VTM-MG
NoVTM-MG

## Slide 369
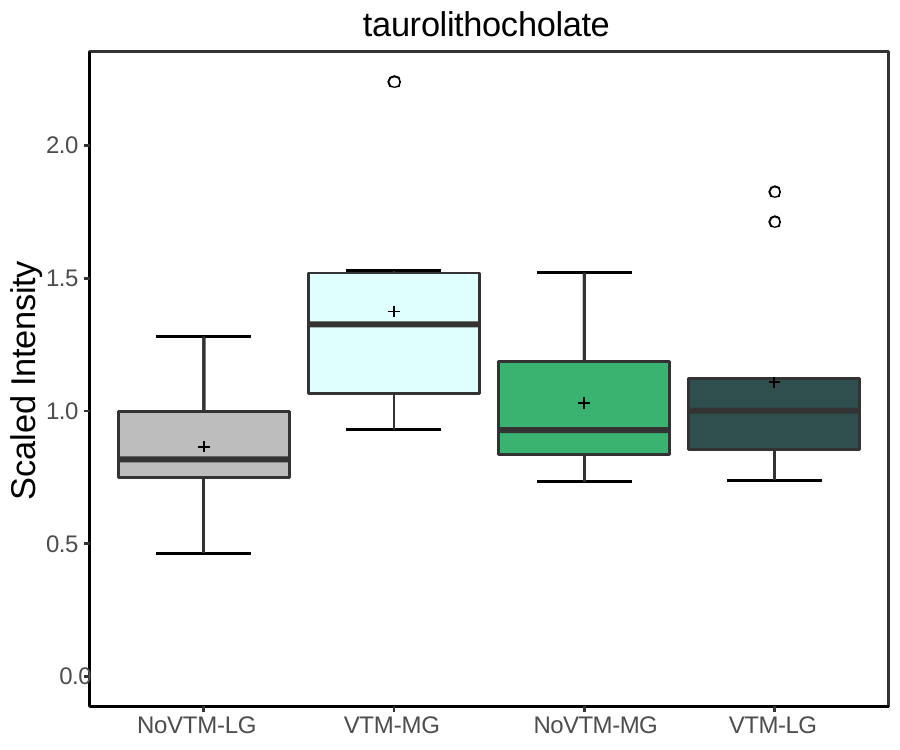

taurolithocholate
2.0
Scaled Intensity
1.5
1.0
0.5
0.0
NoVTM-LG
VTM-MG
NoVTM-MG
VTM-LG

## Slide 370
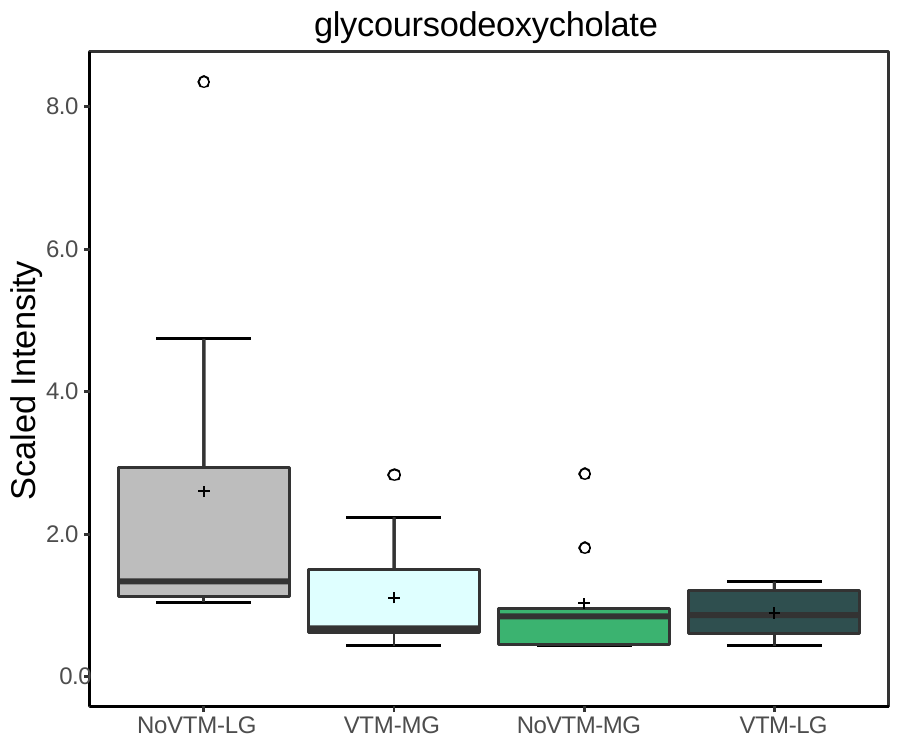

glycoursodeoxycholate
8.0
6.0
Scaled Intensity
4.0
2.0
0.0
NoVTM-LG
VTM-MG
NoVTM-MG
VTM-LG

## Slide 371
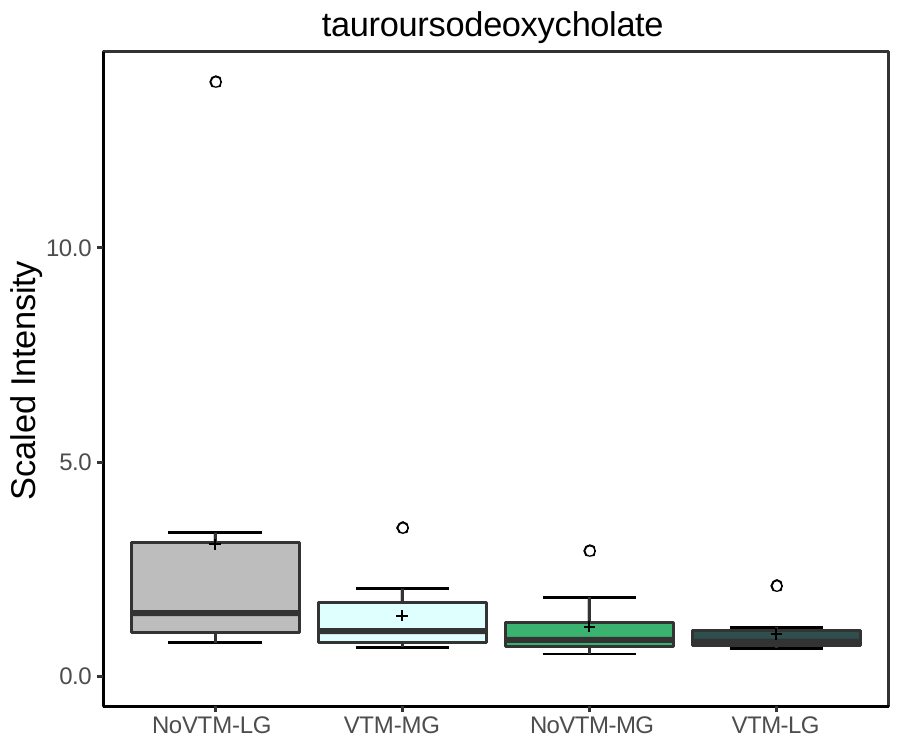

tauroursodeoxycholate
10.0
Scaled Intensity
5.0
0.0
NoVTM-LG
VTM-MG
NoVTM-MG
VTM-LG

## Slide 372
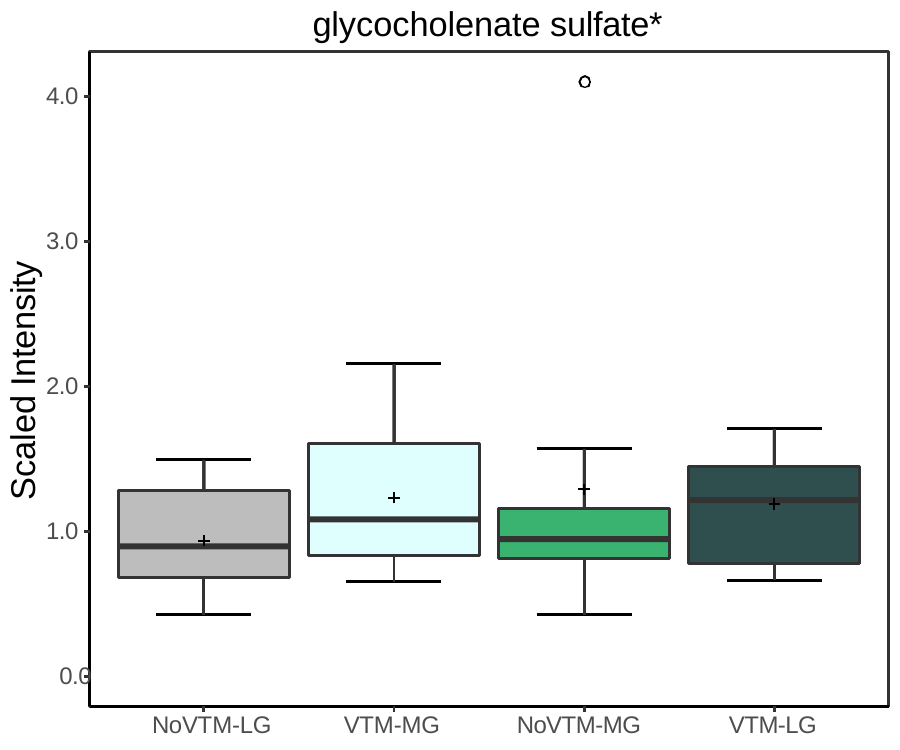

glycocholenate sulfate*
4.0
3.0
Scaled Intensity
2.0
1.0
0.0
NoVTM-LG
VTM-MG
NoVTM-MG
VTM-LG

## Slide 373
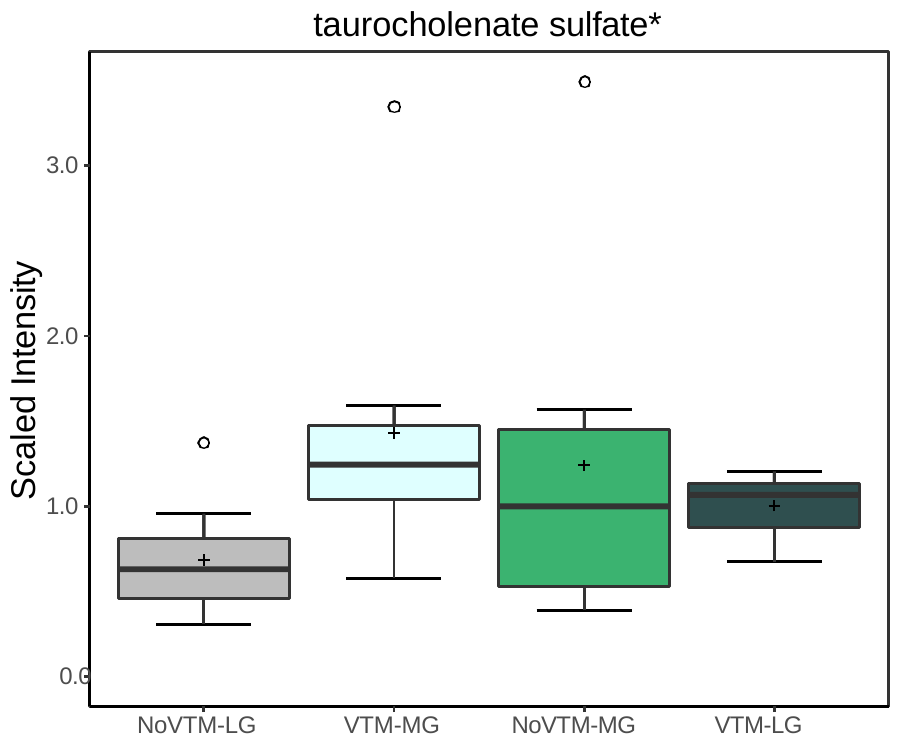

taurocholenate sulfate*
3.0
Scaled Intensity
2.0
1.0
0.0
NoVTM-LG
VTM-MG
NoVTM-MG
VTM-LG

## Slide 374
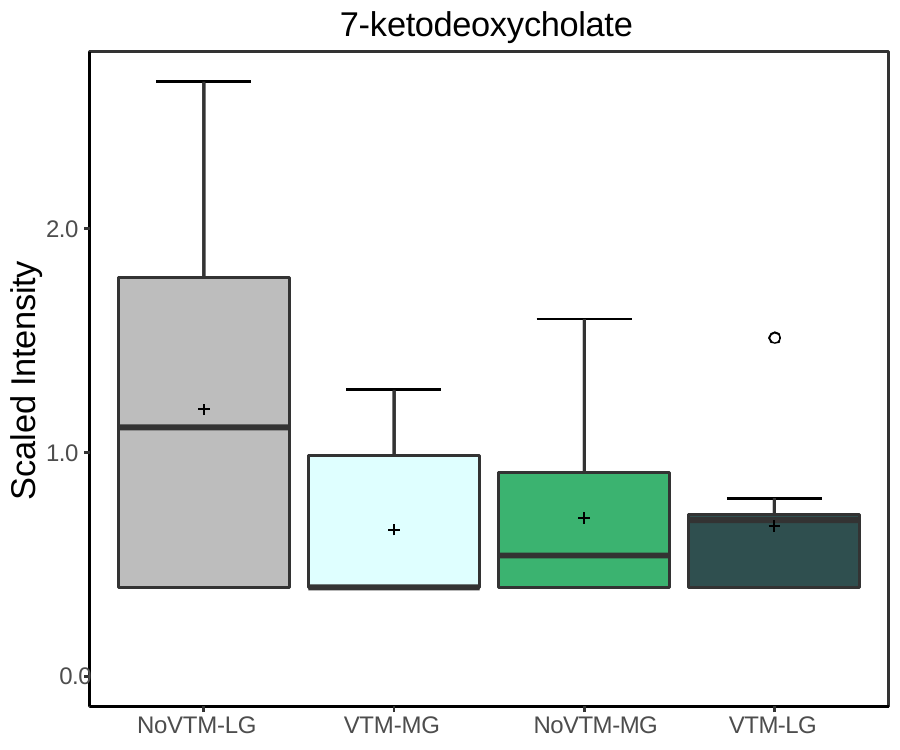

7-ketodeoxycholate
2.0
Scaled Intensity
1.0
0.0
NoVTM-LG
VTM-MG
NoVTM-MG
VTM-LG
